# Health Care Providers and Human Trafficking: What do They Know, What do They Need to Know? Findings from the Middle East, the Caribbean, and Central America

**DOI:** 10.3389/fpubh.2015.00006

**Published:** 2015-01-29

**Authors:** Roderik F. Viergever, Haley West, Rosilyne Borland, Cathy Zimmerman

**Affiliations:** ^1^Department of Health Services Research and Policy, London School of Hygiene and Tropical Medicine, London, UK; ^2^Radboud Institute for Health Sciences, Radboud University Medical Center, Nijmegen, Netherlands; ^3^International Organization for Migration (IOM) Mission in South Sudan, Juba, South Sudan; ^4^International Organization for Migration (IOM) Regional Office for South America, Buenos Aires, Argentina; ^5^Department of Global Health and Development, London School of Hygiene and Tropical Medicine, London, UK

**Keywords:** human trafficking, trafficked persons, training, health care providers, violence, crime, program evaluation

## Abstract

**Background:** Human trafficking is a crime that commonly results in acute and chronic physical and psychological harm. To foster more informed health sector responses to human trafficking, training sessions for health care providers were developed and pilot-tested in the Middle East, Central America, and the Caribbean. This study presents the results of an investigation into what health care providers knew and needed to know about human trafficking as part of that training program.

**Methods:** Participants attended one of seven two-day training courses in Antigua and Barbuda, Belize, Costa Rica, Egypt, El Salvador, Guyana, and Jordan. We assessed participants’ knowledge about human trafficking and opinions about appropriate responses in trafficking cases via questionnaires pre-training, and considered participant feedback about the training post-training.

**Results:** 178 participants attended the trainings. Pre-training questionnaires were completed by 165 participants (93%) and post-training questionnaires by 156 participants (88%). Pre-training knowledge about health and human trafficking appeared generally high for topics such as the international nature of trafficking and the likelihood of poor mental health outcomes among survivors. However, many participants had misconceptions about the characteristics of trafficked persons and a provider’s role in responding to cases of trafficking. The most valued training components included the “Role of the Health Provider,” “Basic Definitions and Concepts,” and “Health Consequences of Trafficking.”

**Discussion:** Training health care providers on caring for trafficked persons has the potential to improve practitioners’ knowledge about human trafficking and its health consequences, and to increase safe practices when responding in cases of trafficking. This study provides lessons for the design of training programs on human trafficking that aim to help health care providers identify and refer victims, and provide care for survivors.

## Introduction

It is estimated that there are approximately 21 million adults and children in situations of forced labor, bonded labor, and forced prostitution around the world as a result of human trafficking (1). Trafficking is frequently associated with repetitive physical and psychological violence, rape, confinement, and deprivation, which have been associated with a range of mental and physical health problems (2–12). The nature of these trafficking-related abuses makes it likely that many, if not most survivors will emerge with multiple health, social and legal service needs. Yet, providing care for trafficked persons can be complicated by, for example, ongoing threats from traffickers, insecure immigration status, or a victim’s participation in judicial processes. Care of trafficked persons often poses further challenges such as: cultural and language barriers (13); unwanted pregnancy; and care for the children of trafficking survivors (14). Additionally, in many trafficking cases, survivors are also struggling with various social and economic challenges including poor social or financial support, feelings of shame and guilt, mistrust of officials and support persons, and social stigma (6, 14). Given the growing identification of trafficking survivors around the world and the potential complexities associated with healthcare provision, it is time to raise practitioners’ awareness of human trafficking and foster service approaches that are well-suited to the care needs of trafficked persons.

### Background and rationale

While there has been greater acknowledgment of the essential role that health care providers can play in the identification and referral of possible victims of trafficking and as part of the support network for trafficking survivors (15), there is still little evidence on providers’ knowledge about human trafficking or their ability to respond to suspected trafficking cases in safe and appropriate ways. Indeed, to date, the role of the health sector in the counter-trafficking response in most countries is extremely limited. Ministries of health or health departments are often not part of formal counter-trafficking national coordination mechanisms, and where they are included, in practice, they are frequently less active than other public sector services, such as shelter providers, police, immigration, and social services. With the growing identification and referral of trafficking survivors into recovery services, there is a pressing need for specialized guidance for health professionals, especially for care providers in areas with high numbers of migrant workers and exploitative labor sectors who might also come into contact with vulnerable populations that could include victims of trafficking (16–18).

The *Caring for Trafficked Persons* handbook was developed to offer guidance to health care providers in responding to cases of human trafficking or suspected situations of trafficking or similar forms of exploitation. (19) This handbook was subsequently adapted into a set of training materials for health care providers (see Supplementary Material) (20), which were piloted in three languages and in seven countries in the Middle East, Caribbean, and Central America. At each training event, participants were asked to complete a survey before and after the 2-day training.

In this article, through an analysis of these surveys, we describe what health care providers know and need to know about human trafficking and appropriate responses in trafficking cases, highlight provider knowledge gaps, and describe user feedback on the *Caring for Trafficked Persons* training program. In doing so, we aim to provide lessons for the design of training programs on human trafficking that aim to help health care providers identify and refer victims, and provide care for survivors.

## Methods

### Handbook

The *Caring for Trafficked Persons* handbook aims “to provide practical, non-clinical guidance to help concerned health care providers understand the phenomenon of human trafficking, recognize some of the health problems associated with trafficking and consider safe and appropriate approaches to providing health care for trafficked persons” (19). This guidance tool was developed by the International Organization of Migration (IOM) and the London School for Hygiene and Tropical Medicine (LSHTM), with the input of a group of international experts, and funding from the United Nations Global Initiative to Fight Trafficking in Persons (UN.GIFT) in 2009.

### Training materials and events

The *Caring for Trafficked Persons* handbook was adapted into a set of training materials for health care providers by IOM and LSHTM, with the input of experienced trainers from both organizations (see Supplementary Material) (20), and funded in part by the United Kingdom’s Economic and Social Research Council. Two-day training courses were conducted as part of the validation and piloting process in seven countries: Antigua and Barbuda, Belize, Costa Rica, Egypt, El Salvador, Guyana, and Jordan. Countries were chosen based on IOM presence and ongoing counter-trafficking projects, as well as the possible interest of counter-trafficking and health partners to organize training courses. In addition, language was a selection criterion, as the training was purposely validated in three different languages.

In each setting, local IOM counter-trafficking specialists coordinated with the international training team to adapt the materials for the local context. Local counter-trafficking trainers provided input to ensure that the training materials were locally relevant and to include information about local resources available to support trafficked persons. Trainings were carried out in English, Spanish, and Arabic. A total of 178 participants attended the two-day training course, which aimed to increase health care provider capacity to respond to cases of human trafficking. Specifically, the course included sessions on basic concepts of trafficking in persons, health consequences for victims/survivors, trauma-informed care approaches, patient and provider safety, and the opportunities and limitations of the health care provider role.

### Selection of training participants

Participants were primarily health care providers and were identified and invited by the IOM and the local counter-trafficking and health authorities in each country. To the extent possible, invitations were extended to health professionals who would be likely to encounter a trafficked person (and benefit directly from the training), and those who were actively working within existing counter-trafficking referral networks (formal or informal), or with other victims of violence. Ministry of Health staff members were frequently involved in the coordination of the training and attended the course. Invitations were also extended to a limited number of other services offering assistance for trafficked people, policy-makers from different ministries and program managers from non-governmental organizations (NGOs) and inter-governmental organizations (IGOs).

### Surveys and data collection

At the start and completion of the course in each training site, participants were asked to complete self-assessment, pre-, and post-training questionnaires. The pre-training questionnaire consisted of 42 questions and collected information about participants’ characteristics, knowledge about human trafficking, and their opinions about appropriate care responses in cases of trafficking. Questions were designed to gather general knowledge about trafficking, explore common misconceptions, and determine health care providers’ understanding of their role in the network of support services. The post-training questionnaire consisted of 17 questions and mainly solicited feedback on the training and its main topics.

The survey questionnaire drew on a tool developed to assess physician readiness to manage intimate partner violence (21) and on the training team’s experience in the field of health and human trafficking. The final pre-training questionnaire for the *Caring for Trafficked Persons* training assessment was comprised of eleven questions about demographics and previous experience, 23 multiple choice questions, seven true-false questions, and 1 open ended question. The post-training questionnaire for the *Caring for Trafficked Persons* training assessment was comprised of five questions about demographics and previous experience, four multiple choice questions, two true-false questions, and six open ended questions. Questionnaires were written in the language of the training and participants were informed that all surveys would remain anonymous.

These evaluations were initially undertaken to acquire participant feedback on the trainings with a view to improving and further developing the training programs. Over the course of assessing the participants’ responses to the course knowledge and evaluation forms it was determined that the results of the trainings were of academic interest, and merited scientific publication.

### Data analysis

Data entry was completed directly after receipt of the participants’ surveys and was separated according to training site. Once collected, the data from both the pre- and post-training surveys were entered into a Microsoft Excel database (MS Office 2009). The integrity of the data was ensured by cleaning the data in Excel and then verifying the entered data with that of the original questionnaires.

Analysis combined quantitative and qualitative methods. Quantitative analysis comprised descriptive analyses of pre-specified or categorized responses to pre- and post-training surveys. Qualitative analysis consisted of thematic analyses used to develop categorizations of free-text responses by participants.

Data analysis was conducted using Microsoft Excel for quantitative data and NVIVO for qualitative data.

## Results

### Pre-training questionnaires

#### Participants characteristics

Of the 178 people who participated in the training events, 165 participants (93%) completed the pre-training questionnaire and 156 participants (88%) completed the post-training questionnaire. An approximately equal number of participants from each of the seven countries completed the questionnaires: the pre-training questionnaires were completed by 29 people in Antigua, 19 people in Belize, 18 people in Costa Rica, 27 people in Egypt, 22 people in El Salvador, 23 people in Guyana, and 27 people in Jordan (participation was slightly less for the post-training questionnaires).

Participants were mostly health professionals and social workers (71%), with nurses particularly well-represented (24%) (Table 1). Several other types of professionals also participated, namely policy-makers from different ministries (8%) and NGO or IGO project managers (7%). Participants were mainly women (72%) of varying ages. The years of professional experience varied too, but there was a comparatively large group of participants with fewer than 5 years of practice experience (26%).

**Table 1:** **Demographic characteristics of participants**.

Categories	*N*	%
**Sex**
Male	45	27.3
Female	119	72.1
No response	1	0.6
**Age**
20–29	31	18.8
30–39	48	29.1
40–49	40	24.2
50–59	31	18.8
60–69	6	3.6
70–79	1	0.6
No response	8	4.8
**Profession**
Nurse	40	24.2
Social worker	23	13.9
Medical Doctor	20	12.1
Psychologist	15	9.1
Policymaker	13	7.9
NGO/IGO project manager	11	6.7
Health educator	6	3.6
Counselor	5	3.0
Researcher	4	2.4
Hospital manager	4	2.4
Detective or inspector	3	1.8
Administrative support worker	2	1.2
Other health care provider	2	1.2
Lawyer	1	0.6
Interpreter	1	0.6
Physiotherapist	1	0.6
Volunteer	1	0.6
No response	13	7.9
**Years of experience**
0	1	0.6
1–5	42	25.5
6–10	23	13.9
11–15	12	7.3
16–20	17	10.3
21–30	19	11.5
31–40	6	3.6
No response	45	27.3
**Total**	165	100.0

*NGO, non-governmental organization; IGO, inter-governmental organization*.

*Nurses included midwives, specialized nurses, and nurse practitioners. Policy-makers worked in Ministries of Health, Social Work, Education, Labor, and Social Development*.

#### Knowledge about human trafficking

Before the training, most participants demonstrated a general awareness of key issues related to human trafficking (Table 2). Only 5% of participants thought trafficking was a very rare occurrence. Most participants were aware that men also become victims of trafficking. However, there was more uncertainty about other characteristics of trafficked persons, with many participants incorrectly speculating that: trafficking must involve movement across an international border; trafficking does *not* include exploitation of children by relatives in domestic work; and that trafficking only happens to people with little education. In addition, almost half of the participants incorrectly believed that there will generally be “obvious signs” if someone is trafficked. Over 20% of participants perceived that getting involved in cases of trafficking was outside the remit of health care providers. There were also considerable misconceptions about appropriate courses of action in terms of contacting the police immediately in suspected cases of trafficking and whether care providers should ask an individual escorting a person they suspect of being trafficked to provide language interpretation for that person.

**Table 2:** **Participants’ knowledge about human trafficking before the training (*N* = 165)**.

Knowledge about human trafficking	Correct answer	Participants’ responses					
		Agree		Disagree		No opinion or no response	
		*N*	%	*N*	%	*N*	%
**General**
Human trafficking is a very rare occurrence	[false]	8	4.8	145	87.9	12	7.3
**Characteristics of trafficked persons**
Men cannot be trafficked, or it is very rare	[false]	11	6.7	137	83.0	17	10.3
All trafficked persons cross an international border	[false]	28	16.9	122	73.9	15	9.1
Children working for relatives in domestic work cannot be considered “trafficked”	[false]	36	21.8	101	61.2	28	17.0
Most people who try to migrate for work will be trafficked	[false]	65	39.4	74	44.9	26	15.8
Being trafficked only happens to low education persons	[false]	23	13.9	136	82.4	6	3.6
**Signals**
There will be obvious signs that a person has been trafficked	[false]	75	45.5	67	40.6	23	13.9
People who are being exploited have difficulty reporting these situations to outsiders, especially professionals	[true]	149	90.3	7	4.2	9	5.5
**Health providers’ response**
Healthcare workers should stay within the confines of diagnosing medical problems and not get involved in cases of trafficking	[false]	35	21.2	122	73.9	8	4.8
It is not a good idea to immediately call the police if you suspect a person has been trafficked	[true]	54	32.7	83	50.3	28	17.0
I believe it is useful to ask a friend of the suspected trafficked person to interpret for him or her, if needed	[false]	70	42.4	69	41.8	26	15.8

*To some questions, the answer is categorically [false] or [true]. To other questions, the answer is more complex, and to answer [true] or [false] is a simplification of the true situation. Such questions were purposefully included to solicit discussion*.

Participants were asked to provide a free-text response to the question “What are the most important health symptoms or indicators that a person may have been trafficked?” Most (82%) of the 131 participants who responded to this question listed mental health problems, in particular anxiety and depression (Table 3). Physical sequelae (49%) were also listed, in particular, sexually transmitted infections (STIs). One-third of participants mentioned signs of abuse (30%). Participants also noted non-health indicators, such as demographic characteristics (20%), indicators related to social interaction with the person (12%), and other non-health indicators (27%) [e.g., deprivation of freedoms (7%); when someone else is translating, accompanying or answering for the person (5%); or when someone is not in the possession of an identification-card (5%)].

**Table 3:** **Participants’ free-text responses before the training to the question “What are the most important health symptoms or indicators that a person may have been trafficked?” (*N* = 131)**.

Health problems and indicators	*N*	%
**Mental health symptoms**	**107**	**81.7**
Fear/anxiety/being afraid/distrust	58	44.3
Depression	53	40.5
Psychological/mental disorders/distress in general	24	18.3
Post-traumatic stress disorder (PTSD)	18	13.7
Other	31	23.7
**Physical health symptoms**	**64**	**48.9**
Sexually transmitted infections (STIs)	38	29.0
Poor nutritional status/dehydration/starvation/hypothermia/malnourishment	16	12.2
Headaches	5	3.8
Unexplained/inconsistent health problems	5	3.8
Somatic complaints related to trafficking/physical indicators of trafficking	5	3.8
Other	4	3.1
**Signs of abuse**	**39**	**29.8**
Signs of abuse/physical trauma/bruising	32	24.4
Signs or history of sexual assault/sexual abuse	8	6.1
Burns	5	3.8
Signs of torture	3	2.3
Other	2	1.5
**Demographics**	**26**	**19.8**
Age (underage)	5	3.8
Person has traveled far away from home and family	4	3.1
Type of work/salary	4	3.1
Migrant/refugee status	3	2.3
Other	3	2.3
**Social interaction with person**	**16**	**12.2**
Keeping eyes down/withdrawn from others	11	8.4
Unwillingness to answer questions	6	4.6
Confusion when taking a history – incoherent/contradictory/distorted information	5	3.8
Not friendly/no friends/no support network	3	2.3
Low self esteem	3	2.3
Other	5	3.8
**Other non-health indicators**	**35**	**26.7**
Not free to leave job or move around/being isolated or guarded from other people/no control over life	9	6.9
Someone else translating/someone else accompanying and answering	7	5.3
Passport confiscated/not in the possession of an ID-card	6	4.6
Poor health care/not sought medical care when should have/signs of neglect	4	3.1
Unable to provide basic info (address/Tel no)/not having a home address	4	3.1
Other	16	12.2

*For each category four subcategories (or more in case of equal scores) are reported in this Table and remaining responses were collated under “other.” Participants’ responses do not add up to 100%, since categories were not mutually exclusive*.

#### Opinions about responding to trafficked persons

Participants’ opinions about how they would respond in their setting to trafficked persons were also surveyed as part of the pre-training questionnaire. Most participants believed they were likely to encounter a trafficked person in their practice, as only 15% thought it was very unlikely (Table 4). When asked whether they felt confident they would know what to do if they encountered a trafficked person in their workplace, 22% stated that they did not feel confident. Reasons for feeling less confident included: feeling that the workplace was not safe enough to discuss trafficking (21%); there would not be enough time to ask someone about trafficking (20%); and that they would not know where to refer a person who reports having been trafficked (18%).

**Table 4:** **Participants’ opinions before the training about responding to trafficked persons (*N* = 165)**.

Participants’ opinions about responding to trafficked persons	Agree		Disagree		No opinion or no response	
	*N*	%	*N*	%	*N*	%
**General**
I am very unlikely to encounter a patient who has been trafficked	25	15.2	124	75.2	16	9.7
I am confident that I know what to do if I encounter a trafficked person in my workplace	100	60.6	36	21.8	29	17.6
**Enquiry**
My workplace is safe enough to discuss confidentially about human trafficking and exploitative situations with my patients	106	64.2	34	20.6	25	15.2
In my practice there is not enough time to ask about trafficking if I suspect someone might be trafficked	33	20.0	113	68.5	19	11.5
I would feel comfortable asking a person if they were in danger from an employer or in very bad or hazardous working conditions	135	81.8	19	11.5	11	6.7
**Referral**
I would know where to refer a person who reports having been trafficked	111	67.3	30	18.2	24	14.5
I am confident I can make the necessary referrals for women who have been trafficked or exploited	117	70.9	24	14.5	24	14.5
I am confident I can make the necessary referrals for children who have been trafficked or exploited	119	72.1	24	14.5	22	13.3
I am confident I can make the necessary referrals for men who have been trafficked or exploited	108	65.5	31	18.8	26	15.8
I am confident I can document trafficking or other abuse accurately and confidentially	120	72.7	23	13.9	22	13.3

### Post-training questionnaires

After the training, the majority of health care providers (86%) believed that they were going to apply in their practice what they learned in the training. Only three participants (2%) did not believe so [no response from (12%)]. Participants reported that certain training sessions were most useful for their work, including: “Role of the Health Care Provider in Caring for Trafficked Persons,” “Basic Definitions and Concepts about Human Trafficking,” and “Health Consequences of Trafficking” (Table 5). Participants indicated that the following sessions were less useful: “Culturally Sensitive Care,” “Comprehensive Assistance,” and “Children and Adolescents.”

**Table 5:** **Participants’ feedback after the training on which topic was the *most* useful for their work (*N* = 156)**.

Topics	%
Role of the Health Providers in Caring for Trafficked Persons	37.8
Basic Definitions and Concepts about Human Trafficking	19.2
Health Consequences of Trafficking	13.5
All topics equally useful	12.2
Comprehensive Assistance	9.0
Features of “Trauma-Informed Care”	7.1
Mental health and trafficking	6.8
Children and Adolescents	4.1
Culturally Sensitive Care	2.6
Other Topic(s) Considered Useful	0.6
No response	3.2

*The total number of responses in this table is larger than 100%, because some participants gave multiple answers, which were counted separately*.

Participants were also asked about the need or desire for additional follow-up training. Of the 156 participants completing the post-training survey, 144 participants (92%) stated they would like follow-up training and suggested that topics of interest included: the role of the health care provider in caring for trafficked person; mental health, trauma-informed care and dealing with the psychological consequences of trafficking; and participating in a service referral network.

## Discussion

Health care is an essential component of a multi-sector response to human trafficking. Health care providers can play a critical role in identifying and referring people who may have been trafficked, and are integral to post-trafficking care. Yet, to date, there has been limited attention to the information and training needs of individuals in the health sector to support their participation in the network of services for survivors or their participation in the broader counter-trafficking response.

Although there is little evidence on what health care providers know about human trafficking and how to respond appropriately, there are strong indications that providers have not been sufficiently included in national or international dialogues on human trafficking or integrated into the network of post-trafficking referral services (14, 16, 17, 22–30). The subject of guidance for health care providers on human trafficking has just started to emerge in the literature, but often appears solely as short opinion articles. For example, Ahn et al. showed recently that there are approximately 27 fairly substantial educational resources on this topic that are publicly available, however, none have been rigorously evaluated (16). This study makes a contribution to this small knowledge base and provides lessons for the design of training programs for health care providers on human trafficking, by assessing health care provider knowledge about human trafficking and appropriate responses in trafficking cases and by soliciting user feedback as part of a training program that was implemented in seven countries in three regions.

There are several important limitations to this analysis, however. First, while we have assessed health care provider knowledge about human trafficking and participant feedback on the *Caring for Trafficked Persons* training program, we have not assessed whether there were increases in knowledge as a result of the training program. This constitutes an important area for future research. Second, the surveys in this study assessed knowledge among participants who were purposively selected based on their likelihood of encountering trafficked persons (or related vulnerable populations, including migrants, workers in informal or exploitative sectors, and victims of violence and exploitation) or providing related services. This targeted selection is likely to mean that this group was more well-informed about human trafficking than an average health care provider. Yet, at the same time, for future training activities, we strongly recommend that participants are selected based on their likelihood of putting the knowledge to use in cases of human trafficking and similar forms of exploitation. Additionally, as these training sessions were carried out in middle- and low-resource country-settings, we are uncertain to what extent these results might be transferable to professionals in a high-resource country.

Despite these limitations, we believe our findings provide useful input for the development of future training programs on human trafficking for health care providers. While many health care providers in these training sessions initially demonstrated a generally good level of knowledge about human trafficking prior to the training, knowledge was not consistent across all participants and there were important misconceptions about important care approaches in cases of human trafficking. Knowledge gaps and misconceptions among a more general population of care providers are likely to be even greater than among this specifically selected participant group.

Particularly complex decisions often arise around contacting police in cases where a provider suspects a patient may have been trafficked. While a provider’s instinct may be to urgently contact the police, experience suggests that people who are trafficked often have well-founded reasons for avoiding contact with law enforcement (e.g., to protect children who are known to the trafficker) (31). Generally, the safest course of action is to first discuss the option of contacting law enforcement with the patient privately and learn his or her preferred assistance option to avoid putting the individual or his or her family in further danger. While this concept can be challenging for health care providers, it resonated for those providers with experience in gender-based violence, and the lessons learned for safe and appropriate responses that do not put the patient or provider at risk.

Another common challenge for health care providers arises around language interpretation, especially given the links between trafficking in persons and international and internal migration. When treating patients who do not speak the local language, it is not uncommon for care providers to rely on bilingual members of the patient’s family for translation, or on other individuals who escort the patient, because of high costs and limited availability of interpreters. Lessons learned from past trafficking cases highlight the risk that the accompanying individual may be involved in the trafficking situation and therefore provide misleading information and/or react badly to suspicious health care providers (e.g., block care, punish the victim) (32). The training emphasizes the importance of identifying a neutral interpreter either at the time of the appointment or scheduling a future follow-up appointment when an independent interpreter can be available. Health care providers are often in the fortunate position of insisting on the need for further treatment.

Because a comprehensive response to human trafficking frequently requires services from a wide range of providers (e.g., shelter services, law enforcement, legal aid, migration and consular services, social and child protection services, etc.), it can often be confusing for providers to determine their obligations and the limits of their responsibilities (14). Those providing health care for trafficked people may pose questions such as: Is it my responsibility to report this? Can I break confidentiality to do so? Who do I need to contact? Should I take any steps before I do so? What can and should I tell my patient about my suspicions? Do I need to consult him or her before I take steps? What about my own safety? These are basic questions that can have serious implications for the safety and well-being of both patients and care providers. This perhaps explains training participants’ strong interest in learning about their role in cases of trafficking or suspected trafficking, e.g., their responsibility to assist beyond clinical treatment.

For example, during training sessions, participants expressed concerns about their limited knowledge of immigration laws when dealing with irregular migrants. While it may be useful for health care providers who are likely to encounter trafficked persons to be familiar with general rules related to immigration, they do not need to know this legislation and, in fact, should be strongly discouraged from offering legal advice to individuals. Instead, the training emphasized the need for health care providers to be able to make appropriate referrals to the proper experts.

While there are clearly additional challenges to treating trafficked persons compared to other vulnerable populations, health care providers can also draw on current knowledge about caring for victims of other types of traumatic events, especially among migrant populations, such as refugees. Serving these populations requires similar familiarity with the effects of current immigration policies, legal aid options, multicultural counseling competencies, and an appreciation for the comprehensive spectrum of services needed by marginalized, vulnerable groups (14, 33, 34). Lessons learned in caring for other violence survivors are also extremely valuable when caring for trafficked persons, because support in the recovery process requires the care provider to recognize the prior trauma exposures and methods of control used by perpetrators. The training sessions focused on how to create safe spaces and use trust-building approaches that facilitate the active participation and decision-making by survivors who were denied agency by those that exploited and abused them.

In planning for future training events, to ensure the efficient use of resources and engagement of the participating care providers, it is important to prioritize (or require) invitations to individuals who are most likely to encounter a trafficked person and related populations. For example, individuals working in emergency services, sexual and reproductive health clinics that may have contact with sex workers, clinics, or mobile teams that have contact with migrants or workers in specific sectors are more likely to encounter trafficked people than, for instance, anesthetists. Future guidance may also aim to be more precise about the varying needs of trafficked persons based on the type of occupational exploitation they experienced. Much of the research to date has focused on sexual exploitation of women and children, and not enough is known about the health consequences of other types of trafficking, such as for labor exploitation, and the health care needs of men, women, boys, and girls trafficked for this purpose. Training sessions for health care providers will also benefit from the participation of representatives from other sectors to foster links with those sectors and build a trusted network of support. At a minimum, clear contact details for locally available resources that can be used for referral in cases of trafficking should be provided.

While more in-depth training should generally be targeted to those likely to care for trafficking survivors, the wider health sector will undoubtedly benefit from broader awareness-raising programs to alert them to the possibility that they may encounter a trafficked person and how to safely refer a suspected victim. Succinct guidance documents and local protocols on possible “red flags” and indicators for identifying trafficked persons, steps to follow in cases of suspicion, and who to contact for additional advice, would also be useful and have previously proven effective for sensitizing the broader health care provider community to domestic violence and child abuse (35, 36).

## Conclusion

Health policy-makers have recently begun to recognize human trafficking as a fundamental health concern. (37) Because of the great likelihood that trafficked persons will require health services both while they are in a trafficking situation and once they have been released, there is every reason to invest in capacity-building of health care providers as a means to improve the well-being and safety of trafficked persons and related populations. It is likely that tested resources such as the training program based on the *Caring for Trafficked Persons* handbook (19) will be an important tool in the arsenal to build active health sector participation in a multi-sector response to trafficking. To improve the ability of training programs to help health care providers identify and refer trafficking victims, and provide care for survivors, it is important that these training programs are evaluated, and the results of those evaluations made publicly available (16). In this article, we have aimed to contribute to knowledge development in this area by describing what health care providers knew and what they needed to know about human trafficking as part of a training program across seven countries in the Middle East, the Caribbean, and Central America.

## Author Contributions

Cathy Zimmerman and Rosilyne Borland led the development of handbook. Cathy Zimmerman, Rosilyne Borland, and Haley West developed the training materials and developed the study methods; Cathy Zimmerman, Rosilyne Borland, and Haley West conducted the trainings with a range of local IOM partners; Haley West led the data collection and entry process; Roderik F. Viergever conducted a literature review that provided the background for the article; Roderik F. Viergever analyzed all results; Roderik F. Viergever wrote the first draft of this article; Cathy Zimmerman, Haley West, and Rosilyne Borland contributed to writing the article; all approve the article as submitted.

## Ethics Statement

This article reports on evaluations that were undertaken of trainings on human trafficking for health care providers. These evaluations were initially undertaken to acquire participant feedback on the trainings with a view to improving and further developing the training programs. Over the course of assessing the outcomes of the evaluations, it was felt that the results of the trainings were of academic interest, and merited scientific publication. At this point, we decided to use the data from the evaluations for a scientific article. All data were anonymized and non-identifiable. Because these evaluations did not start out as a research study, ethics approval was not obtained before the evaluations. Prior to publication, a statement from the LSHTM ethics committee was requested and received that noted that pre-evaluation ethics approval was not required because this project started as an audit project.

## Conflict of Interest Statement

The authors declare that the research was conducted in the absence of any commercial or financial relationships that could be construed as a potential conflict of interest.

## Supplementary Material

The Supplementary Material for this article can be found online at http://www.frontiersin.org/Journal/10.3389/fpubh.2015.00006/abstract

Supplementary file 1: Caring for Trafficked Persons training Presentation.zip

Description: This zip-file provides the training materials used in the Caring for Trafficked Persons training, including the Caring for Trafficked Persons handbook, the Caring for Trafficked Persons training guide and presentations for five training sessions.
