# Supplementary material for: Health Care Providers and Human Trafficking: What do They Know, What do They Need to Know? Findings from the Middle East, the Caribbean, and Central America
Source: Front Public Health. 2015 Jan 29;3:6. doi: 10.3389/fpubh.2015.00006 (PMC4310216; doi:10.3389/fpubh.2015.00006)
Supplement: Supplementary file 1 [file Presentation_1.ZIP › IOM_LSHTM_Caring for Trafficked Persons_2009.pdf]

CARING FOR TRAFFICKED PERSONS

GUIDANCE FOR HEALTH PROVIDERS

IOM International Organization for Migration

UN.GIFT
Global Initiative to Fight Human Trafficking

LONDON SCHOOL of HYGIENE & TROPICAL MEDICINE

The opinions expressed in the report are those of the authors and do not necessarily reflect the views of the International Organization for Migration (IOM). The designations employed and the presentation of material throughout the report do not imply the expression of any opinion whatsoever on the part of IOM concerning the legal status of any country, territory, city or area, or of its authorities, or concerning its frontiers or boundaries.

IOM is committed to the principle that humane and orderly migration benefits migrants and society. As an intergovernmental organization, IOM acts with its partners in the international community to: assist in meeting the operational challenges of migration; advance understanding of migration issues; encourage social and economic development through migration; and uphold the human dignity and well-being of migrants.

Editors: Cathy Zimmerman
London School of Hygiene & Tropical Medicine
Gender Violence & Health Centre

Rosilyne Borland
International Organization for Migration
Migration Health Division

Publisher: International Organization for Migration
17 route des Morillons
1211 Geneva 19
Switzerland
Tel: +41.22.717 91 11
Fax: +41.22.798 61 50
E-mail: hq@iom.int
Internet: http://www.iom.int

ISBN 978-92-9068-466-4
© 2009 International Organization for Migration (IOM)
© London School for Hygiene and Tropical Medicine (LSHTM)
© United Nations Global Initiative to Fight Trafficking in Persons (UN.GIFT)

All rights reserved. No part of this publication may be reproduced, stored in a retrieval system, or transmitted in any form or by any means, electronic, mechanical, photocopying, recording, or otherwise without the prior written permission of the publisher.

07_09

CARING FOR TRAFFICKED PERSONS

GUIDANCE FOR HEALTH PROVIDERS

IOM International Organization for Migration

UN.GIFT
Global Initiative to Fight Human Trafficking

LONDON SCHOOL of HYGIENE &TROPICAL MEDICINE

Acknowledgements

This handbook was made possible through the generous support of the United Nations Global Initiative to Fight Trafficking in Persons. The development of this handbook was coordinated by the International Organization for Migration and the Gender Violence & Health Centre of the London School for Hygiene & Tropical Medicine.

We were privileged to facilitate a broad group of health and human trafficking experts from around the world in the development of *Caring for Trafficked Persons: Guidelines for Health Providers*. Principal authors and contributors included Dr. Melanie Abas (Institute of Psychiatry, Kings College London), Dr. Idit Albert (Centre for Anxiety, Disorders and Trauma, South London & Maudsley NHS Foundation Trust), Dr. Islene Araujo (Migration Health Department, International Organization for Migration), Hedia Belhadj-El Ghouayel (United Nations Population Fund), Rosilyne Borland (Migration Health Department, International Organization for Migration), Jenny Butler (United Nations Population Fund), Sarah Craggs (Counter-Trafficking Division, International Organization for Migration), Dr. Michele Decker (Harvard School of Public Health), Dr. Sean Devine (Independent Consultant), Riet Groenen (United Nations Population Fund), Takashi Izutsu (United Nations Population Fund), Dr. Elizabeth Miller (UC Davis School of Medicine), Dr. Nenette Motus (Regional Office for Southeast Asia, International Organization for Migration), Tina Nebe (United Nations Population Fund), Dr. Anula Nikapota (Institute of Psychiatry, UK-Sri Lanka Trauma Group), Marija Nikolovska (Regional Office for Southern Africa, International Organization for Migration), Siân Oram (doctoral candidate, LSHTM), Donka Petrova (Animus Foundation), Dr. Clydette Powell (Bureau for Global Health, US Agency for International Development), Kate Ramsey (United Nations Population Fund), Timothy Ross (Fundación Social Fénix), Dr. Jesus Sarol (Migration Health Department, International Organization for Migration), Maria Tchomarova (Animus Foundation), Leyla Sharafi (United Nations Population Fund), Dr. Amara Soonthorndhada (Institute

for Population and Social Research, Mahidol University), Aminata Toure (United Nations Population Fund), Jacqueline Weekers (Migration Health Department, International Organization for Migration), Dr. Katherine Welch (Global Health Promise), Dr. Brian Willis (Global Health Promise), Dr. David Wells (Victorian Institute of Forensic Medicine), Dr. Teresa Zakaria (IOM Jakarta, International Organization for Migration), and Dr. Cathy Zimmerman (London School for Hygiene & Tropical Medicine).

Many peer reviewers from around the world supported our authors. Special thanks to Dr. Jane Cottingham, Dr. Claudia Garcia Moreno, Dr. Jason Sigurdson and Dr. Susan Timberlake for ensuring we had detailed inputs from a range of colleagues at the World Health Organization and the United Nations Joint Programme on HIV/AIDS. The handbook would not have been possible without the ongoing support and guidance of Dr. Davide Mosca, Director of the Migration Health Department of the International Organization for Migration and Richard Danziger, Director of the International Organization for Migration's Counter-Trafficking Division.

Table of Contents

Acknowledgements i
Introduction 1
Chapter 1: Human trafficking 7
Chapter 2: The health consequences of human trafficking 15
Chapter 3: Guiding principles 27
Action Sheet 1: Trauma-informed care 33
Action Sheet 2: Culturally appropriate, individualized care 41
Action Sheet 3: Working with interpreters 49
Action Sheet 4: Comprehensive health assessment 57
Action Sheet 5: Special considerations when examining children and adolescents 71
Action Sheet 6: What to do if you suspect trafficking 79
Action Sheet 7: Protection and security 89
Action Sheet 8: Self-care 97
Action Sheet 9: Patient data and files 107
Action Sheet 10: Safe referrals 117
Action Sheet 11: Urgent care 129
Action Sheet 12: Mental health care 137
Action Sheet 13: Sexual and reproductive health 149
Action Sheet 14: Disability 159
Action Sheet 15: Infectious diseases 169
Action Sheet 16: Medico-legal considerations 181
Action Sheet 17: Interactions with law enforcement 191
Conclusion 199
Bibliography 203

INTRO

Introduction

DUC

TION

Introduction

Human trafficking is a harmful and sometimes deadly practice whereby individuals are enticed by jobs and hopes for a better future into a cycle of migration and exploitation. Trafficking of persons has been called:

- a criminal act
- a human rights violation
- a form of exploitation
- an act of violence.

For health care providers, trafficking in persons is best understood as a very serious health risk, because trafficking, like other forms of violence, is associated with physical and psychological harm.

Evidence on human trafficking and exploitation indicates that no region of the world is free of the practice: Trafficking patterns exist in South, Central and North America, Africa, Europe, Asia and in the Pacific. The widespread nature of trafficking suggests that a health provider may at some point come into contact with a person who has been trafficked.

A trafficked person may be referred to a health care provider; a patient may disclose a trafficking experience; or a provider may detect signs that suggest an individual has been trafficked. The informed and attentive health care provider can play an important role in assisting and treating individuals who may have suffered unspeakable and repeated abuse. In fact, health care is a central form of prevention and support in the network of anti-trafficking assistance measures.

Purpose of the guidance

This document aims to provide practical, non-clinical guidance to help concerned health providers understand the phenomenon of human

trafficking, recognize some of the health problems associated with trafficking and consider safe and appropriate approaches to providing health care for trafficked persons. It outlines the health provider's role in providing care and describes some of the limitations of his or her responsibility to assist.

This resource attempts to respond to questions such as: "What special approaches are required for diagnosis and treatment of a patient who has been trafficked?" and "What can I do if I know or suspect someone has been trafficked?"

Victims of trafficking, like victims of other forms of abuse, sustain injuries and illnesses that frequently fall to the health sector to address in a safe and confidential way. For a trafficked person, contact with someone in the health sector may be the first – or only – opportunity to explain what has happened or ask for help.

Special note: Human trafficking is a crime that can be easily confused with other high-risk situations of migrants, including people smuggling and labour exploitation. Although there are legal distinctions between trafficking, smuggling and abusive labour conditions, there are often commonalities between the health risks and needs of people in these different circumstances. For health providers, distinctions in category should not affect the level of care they provide but may be important in determining which referral options they can use. All persons deserve and are entitled to health support and assistance based on human rights and humanitarian principles.

Although this document focuses on trafficked persons, its guidance is designed to be inclusive, with information that may be useful for meeting the health needs of other marginalized or abused populations. The aim is achieving the best health for all.

Target audience

These recommendations are written for health providers who may now or in the future provide direct health care services for individuals who have been trafficked. They are designed to accommodate varying degrees of contact with and involvement in the care and referral of people who have been trafficked. The intended audience includes the following:

- general practitioners and primary care providers
- private and public health providers
- emergency room staff

- health centre staff, such as receptionists or technical staff
- clinicians, e.g., gynaecologists, neurologists, infectious disease specialists
- outreach care providers in fields such as sexual health or refugee/migrant health
- mental health care professionals, e.g., psychologists or psychiatrists.

These guidelines should be made accessible to all providers involved with direct care of trafficked persons. The care approaches described should, to the extent possible, be supported by training and sensitization to ensure appropriate and consistent implementation. Additionally, while this document offers guidance on good practice, different settings will undoubtedly have varying health care contexts and available resources. Recommendations should be adapted to local contexts.

Chapters and action sheets: what they are and how to use them

To encourage the use of these documents by busy health providers, this resource offers the main points of required knowledge and recommended approaches in a succinct manner. This document can be read in sequence or by topic of interest, therefore some concepts are repeated in different action sheets, where relevant. However, if you or your colleagues are unfamiliar with the phenomenon of trafficking or its health risks and consequences for trafficked persons, it is recommended that you read the introductory chapters first.

The guidelines begin with three chapters that provide:

- background information on human trafficking
- current knowledge on the health risks and consequences of trafficking
- guiding principles in the care of trafficked persons.

These chapters are followed by 17 action sheets covering the following general areas:

- tools for the patient encounter, such as trauma-informed care and culturally and linguistically responsive care;
- approaches to various aspects of medical care, such as comprehensive health assessment, acute care, communicable diseases, and sexual and reproductive health;
- strategies for referral, security and case file management, and coordination with law enforcement.

Each action sheet begins with a rationale offering a basic description of the subject and its significance. This is followed by an outline of required actions providing guidance on the particular area of care or strategy.

At the end of most chapters and action sheets is a section on references and further resources to complement and support the information provided. Overall, this guidance document draws from many sources, including such items as other guidelines, tools and standards; research and background materials; and other resources developed by the World Health Organization, the United Nations, non-governmental organizations and academic sources. It is heavily informed by years of collective experience in addressing the consequences of human trafficking of the expert group that compiled the document. The principles and recommendations in this document are grounded in international norms and United Nations conventions. A complete list of all references is provided at the end of the book.

Current evidence on trafficking encompasses primarily the most extreme cases of trafficking, which generally involve severe abuse. The recommendations in this document tend to offer suggestions for treating those who are most affected by a trafficking experience. However, in reality, not all trafficking cases involve extreme abuse and not all trafficked persons experience profound post-trauma reactions. As more trafficking cases come to light in the coming years and individuals feel safer to disclose trafficking experiences, increasingly, cases that are less severe will be reported. Providers should readily adapt the advice in this document to meet the varying level of needs of their patients.

Making a difference

The abuses involved in human trafficking can pose many health risks. In many cases, individuals experience physical and psychological damage and fears that seem overwhelming. The health provider who encounters a trafficked person or other exploited individual has a unique opportunity to provide essential medical care and vital referral options that may be an individual's first step towards safety and recovery.

Chapter 1:
Human Trafficking

Chapter 1:
Human trafficking

For health providers to provide safe and appropriate care to persons who have been trafficked, it is useful to understand the nature of human trafficking and the context of individuals who have been trafficked. This chapter offers basic information about human trafficking and gives a sense of the characteristics and dynamics of what is, in reality, a very complex and diverse phenomenon.

What is the definition of trafficking? The most widely accepted definition of 'trafficking in persons' is found in the *Protocol to Prevent, Suppress, and Punish Trafficking in Persons, Especially Women and Children, Supplementing the United Nations Convention against Transnational Organized Crime*:

> (a) "Trafficking in persons" shall mean the recruitment, transportation, transfer, harbouring or receipt of persons, by means of the threat or use of force or other forms of coercion, of abduction, of fraud, of deception, of the abuse of power or of a position of vulnerability or of the giving or receiving of payments or benefits to achieve the consent of a person having control over another person, for the purpose of exploitation. Exploitation shall include, at a minimum, the exploitation of the prostitution of others or other forms of sexual exploitation, forced labour or services, slavery or practices similar to slavery, servitude or the removal of organs;
>
> (b) The consent of a victim of trafficking in persons to the intended exploitation set forth in subparagraph (a) of this article shall be irrelevant where any of the means set forth in subparagraph (a) have been used;
>
> (c) The recruitment, transportation, transfer, harbouring or receipt of a child for the purpose of exploitation shall be considered "trafficking in persons" even if this does not involve any of the means set forth in subparagraph (a) of this article;
>
> (d) "Child" shall mean any person under eighteen years of age.[1]

[1] Protocol to Prevent, Suppress and Punish Trafficking in Persons, Especially Women and Children, Supplementing the United Nations Convention against Transnational Organized Crime, United Nations, New York, 2000, Article 3. See http://untreaty.un.org/English/TreatyEvent2003/Texts/treaty2E.pdf

Key features of the crime include movement or confinement of an individual, accompanied by coercion and exploitation, usually for the financial profit of the trafficker.

How many people are trafficked? Although human trafficking is recognized as a global phenomenon, there are no reliable statistics on how many people are trafficked. It is clear, however, that this is a global crime that is not abating, because it is both profitable and difficult to detect.

What types of trafficking-related exploitation are common? Individuals may be trafficked and exploited in a variety of ways. The trafficking of women and children for sexual exploitation has been the most commonly recognized form of trafficking. Yet, many, if not more, men, women and children are trafficked for such forms of labour exploitation as work in factories, in the agriculture, construction, fisheries, textile, and mining industries, and for domestic servitude and care services. Traffickers frequently target children for begging, domestic servitude, adoption and petty street theft.

Who are traffickers? There is no single profile of those who traffic and exploit others. A trafficker may be female or male, a member of an organized crime network, part of a small family business or an amateur operator who assists, for example, in the transportation, documentation or logistics of a trafficking operation. Family members, friends and acquaintances of the person who has been trafficked may have participated in or lead the recruitment or other stages of the trafficking and exploitation process. Some traffickers are former victims of trafficking who now recruit and control other victims.

Who do traffickers recruit? Poverty, unemployment, war, natural disasters and desperation are good predictors of vulnerability to being trafficked. However, those who fall prey to traffickers may also be relatively wealthy, relatively educated and come from urban centres.

Where are people trafficked to and from? While international trafficking is often in the spotlight, people who are trafficked may be transported internationally, regionally or, as is the case with many trafficked persons, within their own national borders. Each region has common routes, many of which lead trafficked persons from areas of relative poverty to locations of relative wealth.

What types of abuse do trafficked persons suffer? Trafficking is a crime that is not always evident, its victims not always readily identifiable. It is a form of violence that occurs on a spectrum. Some individuals will suffer extreme physical abuse or torture-like violence, such as beating, burning,

rape and confinement, while others may be subjected to less obvious – but nonetheless coercive and menacing – tactics, including physical and verbal threats to themselves or their families (particularly those with children). Coercive measures may include blackmail, extortion, lies about an individual's ability to gain help from police or others, warnings about being imprisoned for immigration or other crimes and confiscation of such vital identity documents as passports and personal identity cards.

Why do trafficked persons stay in exploitative situations? People who are trafficked very often perceive no other option than to remain under the control of traffickers. Traffickers use common control tactics to force, manipulate and manage victims. Tactics may include: physical, sexual and psychological violence; debt-bondage; threats against family members; lies and deceit; withholding documents, maintaining victims in unpredictable or uncontrollable conditions; and emotional manipulation. When individuals are transported to unfamiliar locations, particularly to places where they do not speak the local language, it is extremely difficult for them to know where to go for help, whom to trust (many come from places where law enforcement is corrupt or indifferent) or how to ask for assistance, and how to navigate an unfamiliar city or remote area. Individuals may fear reprisals for escape attempts or feel afraid of being arrested and imprisoned. Those trafficked outside their home country may fear deportation and returning indebted and without the income that may have been promised them. Paradoxically, many individuals often, therefore, place their hopes for returning home safely in the hands of the very individuals who are exploiting them.

What is the trafficking cycle? Human trafficking is best understood as a process rather than a single act (*see figure 1*).[2] The trafficking cycle begins at the pre-departure or recruitment stage, followed by the travel and destination/exploitation stages. Upon release or escape from the period of exploitation, individuals are often received and/or detained by authorities after which they enter the integration (if remaining at destination) or re-integration (if returned home) stage. Each stage of this cycle poses risks to an individual's health, as well as opportunities for health care professionals and others to intervene with information and assistance.

2 This section is based on the conceptual models and study findings developed in Zimmerman, C. et al., *The Health Risks and Consequences of Trafficking in Women and Adolescents: Findings from a European study*, London School of Hygiene and Tropical Medicine, London, 2003.

Figure 1: Stages of the trafficking cycle[3]

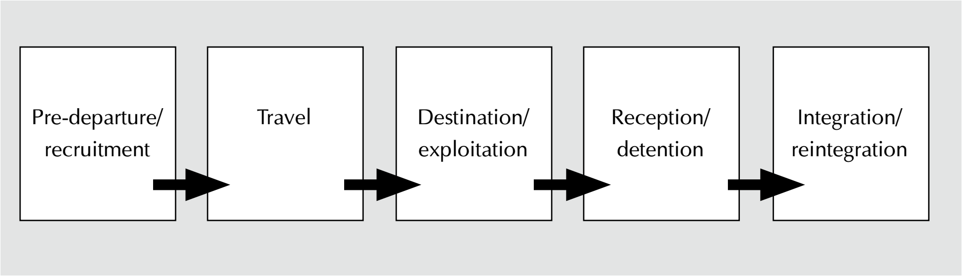

The complex circumstances of trafficked persons

The situation of individuals who are trafficked is almost always complicated. Whether still under a trafficker's control, trying to leave or already out of the trafficking environment, trafficked persons are generally mired in difficult physical, psychological, social, legal and, not least, financial circumstances. To care for individuals who have been trafficked, it is helpful to try to imagine how the world might look through their eyes.

Individuals who are **still in** a trafficking situation may:[4]

- not know or understand what 'trafficking' is.
- have limited personal freedom and feel trapped with no way out.
- work under pressure to pay off debts or feel the burden of a family financial crisis.
- fear reprisals from traffickers or 'employers'.
- work in an informal sector or illicit industry, or in unhealthy, hazardous or dangerous conditions.
- worry about their legal status.
- be moved regularly from place to place, venue to venue.
- be susceptible to penalties, fines or punishment by traffickers or employers.
- lie about legal status, age, country of origin, family or relationship with trafficker.
- worry about the safety and well-being of family at home, especially when traffickers know the location of their family.

---

[3] Based on the conceptual models and study findings developed in Zimmerman, C. et al., *The Health Risks and Consequences of Trafficking in Women and Adolescents: Findings from a European study*, London School of Hygiene and Tropical Medicine, London, 2003.

[4] Adapted from Zimmerman, C. and C. Watts, *WHO Ethical and Safety Recommendations for Interviewing Trafficked Women*, World Health Organization, Geneva, 2003.

- suffer traumatic reactions that affect their ability to remember, trust others, react appropriately, estimate risk and seek or accept assistance.
- view the situation as only temporary and envision a future time when they will eventually earn sufficient income.
- be wary of officials of any type, including health providers.
- seek assurances that they are not to blame for what happened to them.

Individuals who are **out of** a trafficking situation may:[5]

- experience many of the same concerns of individuals who are still in a trafficking situation (see above).
- continue to feel (or be) watched, followed and vulnerable to retribution against themselves or family members.
- have outstanding debts and/or few financial resources.
- have an unstable living situation, have temporary residency, fear imminent removal or deportation, or remain undocumented.
- wish to return home, but not have the means.
- not wish to return home because of abusive, deprived or dangerous past circumstances.
- keep their experience secret from friends, family and others.
- feel ashamed and stigmatized.
- feel independent and empowered by the experience and not wish to be treated like a victim.
- feel pressured to participate in a legal proceeding against traffickers, or feel in danger because of such participation.
- envision no alternative but to return to the traffickers.
- continue to experience extreme stress reactions that affect physical, sexual, psychological and social functioning.
- feel that talking about the past is reliving it.
- be unable to use health or other resources because of financial circumstances, legal status, language barriers, logistics concerns or alienation.

Any one of these reactions can make seeking help difficult for a trafficked person. Once an individual is able to access care, these sensitivities, particularly shame, can make disclosing concerns, posing questions and expressing frustrations stressful. People who have been trafficked need assurance that they are not to blame for what happened to them. They also need to regain a sense of being respected and accepted.

5 Ibid.

Particular complexities

Some trafficker-victim situations are particularly complex, and may pose special challenges in work with trafficked persons. There are many reasons that may prevent a person who has been trafficked from disclosing information or cause them to alter details about themselves, their situation, their intentions or their family.

- **Victims with family members at potential risk:** Individuals often have family members, especially children, who may have a major impact on their decision-making, including decisions about their own safety and well-being.
- **Victims with an intimate or familial relationship with their trafficker:** In some cases, victims may have a personal relationship with the trafficker. Women may currently have, or have had in the past, a romantic relationship with their abuser. Children may be exploited by a family member or someone they look up to as a parental figure.
- **Victim-perpetrator:** Victims may have 'moved up' from being a victim of trafficking to being a recruiter or manager of other trafficked persons.

Each of these situations can complicate the provision and acceptance of assistance. For the trafficked person, these circumstances may pose problems of dual loyalty, ongoing fear or intimidation and wavering intentions about the future. Health care providers may find that patients do not keep appointments and are not able to adhere to treatment, or that patients' health care needs become intertwined with other support needs.

Responses to human trafficking

Responses to human trafficking usually focus on three broad areas known as the three 'Ps': Prevention, Protection and Prosecution. Prevention activities include awareness-raising and education to warn potential victims about trafficking, for example, and activities to prevent exploitation of migrant workers. Protection encompasses the support mechanisms and resources aimed at assisting victims and ensuring their safety. Prosecution actions are associated with law enforcement and aimed at identifying, arresting and criminally prosecuting perpetrators of trafficking.

The following chapter describes some of the evidence around the health risks and consequences associated with human trafficking and discusses the implications for providing health care.

Chapter 2: The health consequences of human trafficking

Chapter 2: The health consequences of human trafficking

Trafficked persons may have health problems that are minor or severe, but few individuals are unscathed. Many will experience injuries and illnesses that are severe, debilitating and often enduring. Abuse, deprivation and stress-filled or terrifying circumstances are all hallmarks of human trafficking.

The *Protocol to Prevent, Suppress, and Punish Trafficking in Persons, Especially Women and Children, Supplementing the United Nations Convention against Transnational Organized Crime* establishes the basis of the assistance measures that should be provided for trafficked persons [Article 6 (3)]:

"Each State Party shall consider implementing measures to provide for the physical, psychological and social recovery of victims of trafficking in persons, including, in appropriate cases, in cooperation with non-governmental organizations, other relevant organizations and other elements of civil society, and, in particular, the provision of:

(a) appropriate housing;
(b) counselling and information, in particular as regards their legal rights, in a language that the victims of trafficking in persons can understand;
(c) medical, psychological and material assistance; and
(d) employment, educational and training opportunities." [6]

[6] Protocol to Prevent, Suppress and Punish Trafficking in Persons, Especially Women and Children, Supplementing the United Nations Convention against Transnational Organized Crime, United Nations, New York, 2000. See http://untreaty.un.org/English/TreatyEvent2003/Texts/treaty2E.pdf

Additionally, Article 6 (4) also recognizes the needs of particularly vulnerable groups:

> "Each State Party shall take into account, in applying the provisions of this article, the age, gender and special needs of victims of trafficking in persons, in particular the special needs of children, including appropriate housing, education and care."[7]

In meeting the requirements of this Protocol, medical and other health providers are a vital link in the chain of care required by persons who are trafficked.

Diagnosing the health needs of trafficked persons is often complex because their symptoms generally reflect the cumulative effects of the health risks they face throughout the trafficking process (*see chapter 1, figure 1*).

Current knowledge indicates that most people who are trafficked are exposed to health risks before, during, and even after the period of exploitation, such as when they are held in detention centres or prisons, or when they are on the streets, often left isolated from assistance. It is worth noting that health care for trafficked persons may also be challenging because of what might be termed 'hostile environments', e.g., when patients are in a closed facility, or in locales where medical care is not state-supported, or in places where law enforcement cannot be trusted,[8] or when deportation or relocation procedures are uncertain.

Table 1 summarizes some of the basic categories of health risks to trafficked persons, as well as their consequences. Many of these overlap, particularly psychological morbidity, which is linked to most physical, sexual and social health risks.

---

[7] Ibid.

[8] Although police and other members of law enforcement are essential counter-trafficking partners, sometimes individual police officers are involved with the criminal networks that traffic human beings. See action sheet 17 for more information.

Table 1: Summary of the health risks and consequences of being trafficked[9]

HEALTH RISKS	POTENTIAL CONSEQUENCES
**Physical abuse, deprivation**	Physical health problems, including death, contusions, cuts, burns, broken bones
**Threats, intimidation, abuse**	Mental health problems including suicidal ideation and attempts, depression, anxiety, hostility, flashbacks and re-experiencing symptoms
**Sexual abuse**	Sexually transmitted infections (including HIV), pelvic inflammatory disease, infertility, vaginal fistula, unwanted pregnancy, unsafe abortion, poor reproductive health
**Substance misuse**<br>Drugs (legal & illegal), alcohol	Overdose, drug or alcohol addiction
**Social restrictions & manipulation & emotional abuse**	Psychological distress, inability to access care
**Economic exploitation**<br>Debt bondage, deceptive accounting	Insufficient food or liquid, climate control, poor hygiene, risk-taking to repay debts, insufficient funds to pay for care
**Legal insecurity**<br>Forced illegal activities, confiscation of documents	Restriction from or hesitancy to access services resulting in deterioration of health and exacerbation of conditions
**Occupational hazards** (see Table 2)<br>Dangerous working conditions, poor training or equipment, exposure to chemical, bacterial or physical dangers	Dehydration, physical injury, bacterial infections, heat or cold overexposure, cut or amputated limbs
**Marginalization**<br>Structural and social barriers, including isolation, discrimination, linguistic and cultural barriers, difficult logistics, e.g., transport systems, administrative procedures	Unattended injuries or infections, debilitating conditions, psycho-social health problems

Influences on a patient's health may include pre-existing chronic or genetic conditions, exposure to infectious diseases, repetitive physical, sexual and psychological violence, chronic deprivation, hazards related to various forms of labour exploitation, and deterioration of conditions resulting from lack of diagnosis and care. As is the case with victims of torture, individuals who have been trafficked are likely to sustain multiple physical or psychological injuries and illnesses and report a complex set of symptoms.

[9] This section is based on the conceptual models and study findings developed in Zimmerman, C. et al., *The Health Risks and Consequences of Trafficking in Women and Adolescents: Findings from a European study*, London School of Hygiene and Tropical Medicine, London, 2003.

Trafficked and exploited persons often live and work at the margins of society. They frequently experience discrimination and neglect and their access to health and safety resources is restricted. Caring for these groups that are nearly invisible to mainstream services requires concerted efforts on the part of health care providers.

Evidence on the health of trafficked persons

To date, there is limited research-based data on the health of trafficked persons. Most existing evidence on health is based on individuals attending post-trafficking services, and primarily applies to women and girls trafficked for sexual exploitation.

Current knowledge suggests, for example, that, prior to being recruited, more than half of trafficked women and girls may have been exposed to physical and/or sexual abuse. This history of violence may, in fact, have influenced individuals' vulnerability to recruitment and is likely to contribute to post-trafficking health problems.

Evidence from women and girls suggests that common post-trafficking symptoms and problems include the following:

- headaches (among the most prevalent and enduring physical symptoms)
- fatigue
- dizziness
- memory loss
- sexually transmitted infections
- abdominal pain
- back pain
- dental problems.

Additional health problems frequently noted include weight loss, eating disorders, sleep disturbance and insomnia.

For individuals trafficked into sectors other than sexual exploitation, there is currently little research-based data. Accounts from assistance organizations around the world suggest that individuals are forced into high-risk settings that pose numerous hazards related to their jobs and to their living conditions. Health and safety standards in such exploitative settings are generally extremely low.

Those caught in situations of exploitation may remain silent about their conditions because they often do not know where to seek help and may try

to endure, in hopes of eventually gaining promised income. Table 2 gives examples of common industries for labour exploitation and general labour-related health risks and consequences.

Table 2: Industries, health risks and consequences

Industries	Labour-exploitation health risks	Health consequences
• Construction<br>• Manufacturing (e.g., textile, metal, wood)<br>• Industrial fishing and fisheries<br>• Agriculture<br>• Domestic servitude<br>• Mining, quarrying<br>• Food processing<br>• Forestry<br>• Leather & tanning<br>• Carpet-weaving<br>• Livestock	• Poor ventilation, sanitation and nutrition<br>• Sleep deprivation, long hours<br>• Repetitive-motion activities, e.g., back-bending, lifting<br>• Poor training on heavy or high-risk equipment<br>• Chemical hazards<br>• Poor/no personal protective equipment (PPE), e.g., hats, helmets, gloves, goggles<br>• Heat or cold stress<br>• Airborne contaminants, e.g., fumes, dust, particles<br>• Bacterial contaminants, e.g., water, food, soil	• Exhaustion<br>• Malnutrition<br>• Dehydration<br>• Repetitive-motion syndromes, strains<br>• Heat stroke or stress, hypothermia, frostbite<br>• Repetitive syndromes, e.g., back, neck and joint problems<br>• Accidental injuries, e.g., severed limbs, broken bones, concussions<br>• Respiratory problems, lung cancer, endotoxin or asbestos contamination<br>• Skin infections, diseases, cancer, occupational dermatosis<br>• Gastro-intestinal infection (water- and food-related)

Psychological trauma and stress

In the most extreme cases, trafficking-related abuses and post-trafficking psychological symptoms can be compared to the violence, restrictions and psychological reactions identified in torture victims.[10] Characteristic features of torture and trafficking situations are life-threatening events and persistent stress and repetitive or chronic danger. Studies on torture have shown that the 'unpredictability' and 'uncontrollability' of traumatic events are features highly predictive of an intense or prolonged psychological reaction.[11] Common post-trauma responses include such post-traumatic stress symptoms as post-traumatic stress disorder, depression, anxiety and hostility or irritability. Where sexual abuse has occurred, these symptoms may be particularly acute. Suicidal ideation and suicide attempts are not uncommon. Action sheet 12

[10] Zimmerman, C. et al., 2003.

[11] Basoğlu, M. and S. Mineka, "The role of uncontrollable and unpredictable stress in post-traumatic stress responses in torture survivors" in *Torture and Its Consequences: Current Treatment Approaches*, M. Basoğlu, Ed., Cambridge University Press, New York, 1992.

provides detailed information on post-trafficking mental health and offers suggestions for addressing the psychological health of trafficked persons.

Detecting and responding to a person who may have been trafficked

There are no definitive symptoms by which to identify a person who has been trafficked. However, a health provider may suspect that an individual has been trafficked or has suffered extreme forms of exploitation when she or he presents with industry-related morbidity and post-trauma reactions and reports having migrated for a trafficking-related form of labour.

If a provider suspects or learns that an individual has been trafficked, it is important to be prepared with appropriate and up-to-date referral information and to offer care in a sensitive, confidential way. Caring for persons who have been trafficked may pose a number of challenges, but if the health provider is informed and attentive, assistance can be provided safely and effectively. Providing good health care requires adopting approaches that take into account, among other things, past or current risk of being subject to violence, post-trauma reactions, social or cultural differences, economic circumstances associated with debts and legal status (*see action sheets 1, 2, and 3*).[12]

Providers should take time to gain trust and learn about the individual's risks and restrictions. They should also make the effort to act in ways that assure individuals that they are respected and not held responsible for the crimes that occurred.

The role of the health care provider

While trafficking often appears to be an elusive and impenetrable phenomenon, health providers have multiple opportunities to intervene with information and care. As countries, law enforcement and non-governmental organizations give greater attention to the industry of human trafficking, more trafficked persons are likely to be identified.

Although many of the medical needs of trafficked persons may be treated through standard clinical practices, persons who have been trafficked are often hidden or alienated from services and exposed to multiple dangers which can pose diagnostic and treatment challenges. Intervention approaches may be similar or linked to activities designed to reach marginalized or vulnerable

---

[12] International Organization for Migration, *The IOM Handbook on Direct Assistance for Victims of Trafficking*, IOM, Geneva, 2007.

groups, such as migrants, refugees or mobile populations, minorities and low-wage or irregular labourers.

Based on growing information about the abuses and health problems suffered by victims of trafficking, and the lessons learned about protecting hard-to-reach populations, the health care community should now try to become equipped with well-considered approaches to address the diverse – and often highly sensitive – needs of people who have been trafficked.

REFERENCES AND RESOURCES

Anderson, B. and B. Rogaly
2005 *Forced Labour and Migration to the UK*, Oxford: Centre for Migration, Policy and Society (COMPAS), in association with the Trades Union Congress, TUC, London, 2005.

Anti-Slavery International
2006 *Trafficking in Women, Forced Labour and Domestic Work in the Context of the Middle East and Gulf*, working paper, Anti-Slavery International, London, 2006.

Anti Slavery International and International Confederation of Free Trade Unions (ICTFU)
2001 *Forced Labour in the 21st Century*, Anti-Slavery International, London, 2001.

Basoğlu, M. and Ş. Mineka
1992 "The role of uncontrollable and unpredictable stress in post-traumatic stress responses in torture survivors" in T*orture and Its Consequences: Current Treatment Approaches*, M. Basoğlu, Ed., Cambridge University Press, New York, 1992.

Canadian Centre for Occupational Health and Safety
2008 "Extreme hot or cold temperature conditions", web information, available at « http://www.ccohs.ca/oshanswers/phys_agents/hot_cold.html » CCOHS, Hamilton, Ontario, Canada, page last updated 20 October 2008 (accessed 2 January 2009).

Hossain, M. et al.
2005 *Recommendations for Reproductive and Sexual Health Care of Trafficked Women in Ukraine: Focus on STI/RTI Care, First Edition*, London School of Hygiene & Tropical Medicine and the International Organization for Migration, Kiev, 2005.

International Labour Organization

2005 *A Global Alliance Against Forced Labour: Global report under the follow-up to the ILO Declaration on Fundamental Principles and Rights at Work*, International Labour Conference, 93rd Session 2005, Report I (B), International Labour Office, Geneva, 2005.

International Organization for Migration

2007 *The IOM Handbook on Direct Assistance for Victims of Trafficking*, IOM, Geneva, 2007.

2006 Breaking the Cycle of Vulnerability: Responding to the Health Needs of Trafficked Women in East and Southern Africa, IOM, Pretoria, South Africa, September 2006.

Rende Taylor, L.

2008 Guide to ethics and human rights in counter-trafficking. Ethical standards for counter-trafficking research and programming. United Nations Inter-agency Project on Human Trafficking

Stellman, J. M. (Editor-in-chief)

1998 *Encyclopaedia of Occupational Health and Safety, Fourth Edition,* International Labour Organization, Geneva, 1998.

United Nations

2000 Protocol to Prevent, Suppress and Punish Trafficking in Persons, Especially Women and Children, Supplementing the United Nations Convention against Transnational Organized Crime, United Nations, New York, 2000.

United Nations Office of the High Commissioner for Human Rights

2002 *Recommended Principles and Guidelines on Human Rights and Human Trafficking*, Report of the United Nations High Commissioner for Human Rights to the Economic and Social Council (E/2002/68/Add.1), United Nations Economic and Social Council, New York, 20 May 2002.

United States Centers for Disease Control and Prevention
Electronic library of construction occupational safety and health
http://www.cdc.gov/elcosh/

United States Department of Health & Human Services

2008 *Fact Sheet: Human Trafficking*, United States Department of Health and Human Services Administration of Children &

Families, Campaign to Rescue and Restore Victims of Human Trafficking, Washington, DC, USA, January 2008.

2007 *Common Health Issues Seen in Victims of Human Trafficking*, web information available at « http://www.acf.hhs.gov/trafficking/campaign_kits/tool_kit_health/health_problems.html » United States Department of Health and Human Services Administration of Children & Families, Campaign to Rescue and Restore Victims of Human Trafficking, Washington, DC, Campaign to Rescue and Restore Victims of Human Trafficking, October 2007.

United States Department of State

2007 *Health Consequences of Trafficking in Persons*, fact sheet, Department of State Office to Monitor and Combat Trafficking in Persons, Washington, DC, USA, 8 August 2007.

University of California at Davis

"A guide to agricultural heat stress", newsletter, Agricultural Personnel Management Program, Davis, California, USA, undated.

Zimmerman, C. et al.

2006 *Stolen Smiles: The physical and psychological health consequences of women and adolescents trafficked in Europe,* London School of Hygiene and Tropical Medicine, London, 2006.

Zimmerman, C. et al.

2003 *The Health Risks and Consequences of Trafficking in Women and Adolescents: Findings from a European study*, London School of Hygiene and Tropical Medicine, London, 2003.

Zimmerman, C. and C. Watts,

2003 *WHO Ethical and Safety Recommendations for Interviewing Trafficked Women*, World Health Organization, Geneva, 2003.

CHAPTER THREE

Chapter 3:
Guiding principles

Chapter 3:
Guiding principles

Caring for persons who have been trafficked requires special attention to an individual's health, safety and well-being that often goes well beyond the basic medical principle of 'do no harm'. Individuals who have been through traumatic events need to regain a sense of safety, dignity and control over their bodies and actions. They need to be encouraged to seek information, question their options and assert their choices. Health care providers can help foster feelings of security, self-esteem and self-determination by adopting approaches to care that emphasize confidentiality, information-giving, informed consent and respect for individual decision-making. Practitioners may help protect patients from present and future harm by ensuring that their services and staff are sensitive to the vulnerabilities of trafficked persons and that referral options are safe, appropriate and convenient.

The following guiding principles are considered good practice for all professionals involved with persons who have been trafficked. Health providers should integrate these ethical and human rights standards into all aspects of health care for trafficked and exploited persons.

1. **Adhere to existing recommendations** in the *WHO Ethical and Safety Recommendations for Interviewing Trafficked Women*[13] (see Figure 2 at the end of this chapter).
2. **Treat all contact with trafficked persons as a potential step towards improving their health.** Each encounter with a trafficked person can have positive or negative effects on their health and well-being.
3. **Prioritize the safety of trafficked persons, self and staff** by assessing risks and making consultative and well-informed decisions. Be aware of the safety concerns of trafficked persons and potential dangers to them or their family members.

[13] Zimmerman, C. and C. Watts, *WHO Ethical and Safety Recommendations for Interviewing Trafficked Women*, World Health Organization, Geneva, 2003.

4. **Provide respectful, equitable care that does not discriminate** based on gender, age, social class, religion, race or ethnicity. Health care should respect the rights and dignity of those who are vulnerable, particularly women, children, the poor and minorities.
5. **Be prepared with referral information and contact details for trusted support persons** for a range of assistance, including shelter, social services, counselling, legal advocacy and law enforcement. If providing information to persons who are suspected or known victims who may still be in contact with traffickers, this must be done discretely, e.g., with small pieces of paper that can be hidden.
6. **Collaborate with other support services** to implement prevention activities and response strategies that are cooperative and appropriate to the differing needs of trafficked persons.
7. **Ensure the confidentiality and privacy of trafficked persons and their families.** Put measures into place to make sure all communications with and about trafficked persons are dealt with confidentially and that each trafficked person is assured that his or her privacy will be respected.
8. **Provide information in a way that each trafficked person can understand.** Communicate care plans, purposes and procedures with linguistically and age-appropriate descriptions, taking the time necessary to be sure that each individual understands what is being said and has the opportunity to ask questions. This is an essential step prior to requesting informed consent.
9. **Obtain voluntary, informed consent**. Before sharing or transferring information about patients, and before beginning procedures to diagnose, treat or make referrals, it is necessary to obtain the patient's voluntary informed consent. If an individual agrees that information about them or others may be shared, provide only that which is necessary to assist the individual (e.g., when making a referral to another service) or to assist others (e.g., other trafficked persons).
10. **Respect the rights, choices, and dignity of each individual by:**
    - Conducting interviews in private settings.
    - Offering the patient the option of interacting with male or female staff or interpreters. For interviews and clinical examinations of trafficked women and girls, it is of particular importance to make certain female staff and interpreters are available.

- Maintaining a non-judgmental and sympathetic manner and showing respect for and acceptance of each individual and his or her culture and situation.
- Showing patience. Do not press for information if individuals do not appear ready or willing to speak about their situation or experience.
- Asking only relevant questions that are necessary for the assistance being provided. Do not ask questions out of simple curiosity, e.g., about the person's virginity, money paid or earned, etc.
- Avoiding repeated requests for the same information through multiple interviews. When possible, ask for the individual's consent to transfer necessary information to other key service providers.
- Do not offer access to media, journalists or others seeking interviews with trafficked persons without their express permission. Do not coerce individuals to participate. Individuals in 'fragile' health conditions or risky circumstances should be warned against participating.

11. **Avoid calling authorities, such as police or immigration services, unless given the consent of the trafficked person.** Trafficked persons may have well-founded reasons to avoid authorities. Attempts should be made to discuss viable options and gain consent for actions.[14]
12. **Maintain all information about trafficked persons in secure facilities.** Data and case files on trafficked persons should be coded whenever possible and kept in locked files. Electronic information should be protected by passwords.

Special note regarding children: Care for children who have been abused or exploited requires special attention. Apply the above principles to children, including their right to participate in decisions that will affect them. If a decision is made on behalf of a child, the best interests of the child should be the overriding consideration, and appropriate procedures should be followed. UNICEF's *Reference Guide on Protecting the Rights of Child Victims of Trafficking in Europe* provides some guidance on these issues and offers additional resources that can be consulted (*see references section for more details)*.

These principles may serve as the basis for rights-based care strategies that recognize the vulnerability of individuals who are in or have emerged from

[14] Please see action sheet 16 for special considerations related to competence, capacity, and guardianship.

trafficking situations. They are only fully beneficial if they are communicated to all health personnel who might come into contact with trafficked persons and if adherence to the principles is monitored on a regular basis.

Figure 2: Ten guiding principles for ethical and safe interviews[15]

1	Do no harm
2	Know your subject and assess the risks
3	Prepare referral information – do not make promises that you cannot fulfil
4	Adequately select and prepare interpreters and co-workers
5	Ensure anonymity and confidentiality
6	Get informed consent
7	Listen to and respect each person's assessment of their situation and risks to their safety
8	Do not re-traumatize individuals
9	Be prepared for emergency intervention
10	Put information collected to good use

REFERENCES AND RESOURCES

United Nations Children's Fund

2004 *Reference Guide on Protecting the Rights of Child Victims of Trafficking in Europe*, UNICEF, 2006.

United Nations High Commissioner for Refugees

2003 *Sexual and Gender-Based Violence against Refugees, Returnees, and Internally Displaced Persons: Guidelines for prevention and response*, UNHCR, Geneva, May 2003.

Zimmerman, C. and C. Watts,

2003 *WHO Ethical and Safety Recommendations for Interviewing Trafficked Women*, World Health Organization, Geneva 2003.

2005 *Guidelines for Gender-Based Violence Interventions in Humanitarian Settings: Focusing on Prevention of and Response to Sexual Violence in Emergencies*, IASC, Geneva, September 2005.

[15] Adapted from Zimmerman, C. and C. Watts, W*HO Ethical and Safety Recommendations for Interviewing Trafficked Women*, World Health Organization, Geneva 2003.

Action Sheet 1:
Trauma-informed care

Action Sheet 1: Trauma-informed care

RATIONALE

Many individuals who have been trafficked will have experienced life-threatening or traumatic events prior to, during and sometimes after the trafficking experience. In addition to suffering from violence that is obvious because of physical injuries, trafficked persons may also sustain less evident health problems resulting from abuse.

This action sheet focuses on a central framework for the care of trafficked persons that acknowledges the impact of these traumatic experiences. The goal in caring for trafficked persons is to ensure that all care is:

- adapted to the individual's needs;
- supportive and avoids judgmental statements or actions;
- integrated and holistic, treating the trafficked person as a whole person, not just a list of clinical symptoms;
- empowering, ensuring that the patient's rights to information, privacy, bodily integrity and participation in decision-making are respected;
- supportive of healing and recovery through a patient-centred treatment plan.

Trauma-informed care involves recognizing the impact of traumatic experiences (specifically, a range of violence that may include abuse prior to the actual trafficking experience) on an individual's life and behaviour, and on their perceptions of themselves and their bodies.[16] Trafficked persons often present with a constellation of symptoms and disease conditions (*see*

[16] Harris, M. and R.D. Fallot, "Envisioning a trauma-informed service system: a vital paradigm shift", *New Directions for Mental Health Services*, vol. 89, Spring 2001, pp. 3-22 and Elliott, D. et al., "Trauma-informed or trauma-denied: Principles and implementation of trauma-informed services for women ", *Journal of Community Psychology*, vol. 33, no. 4 (special issue on 'Serving the needs of women with co-occurring disorders and a history of trauma'), July 2005, pp. 461-477.

action sheets 4, 5, 11, 12, 13 and 15) that are influenced by these traumatic experiences. Hypervigilance around being examined, mistrust of health care providers, anxiety about sitting in a waiting room full of other people and fear of medical procedures may all be related to the abuses experienced while being trafficked. Providers of trauma-informed care incorporate into their routine clinical practice an appreciation of how traumatic experiences may affect their patients' behaviours and perceptions of their bodies and health. For providers who may only see these patients for brief clinical encounters (e.g., the individual is in transit), a non-judgmental, comforting approach helps to reinforce for the patient that no one deserves to be hurt, and that everyone deserves to be treated with respect. For those providers who have the opportunity to work for more extended periods with trafficked persons, the trauma-informed approach, which acknowledges exposure to violence, can serve to build trust with patients and may facilitate discussing abuse and the trafficking experience.[17]

Cultural norms, age, education, gender and personal histories influence how trafficked persons express reactions to traumatic experiences; such reactions may include anger, hostility, irritability, self-harm and withdrawal, as well as numbing or dissociative states (*see action sheet 12*). Notably, reactions to traumatic experiences, presenting symptoms in the clinical setting, and how patients talk about what has happened vary considerably. This means that there is no simple 'right way' to approach all trafficked persons.

Yet, experience suggests that it is useful for providers to empower patients by encouraging their participation and offering information and support throughout the clinical encounter.[18] Providers who recognize the intersection of physical and psychological problems in trafficked persons can address medical and mental health needs in an integrated way.

Related to trauma-informed care is the concept of **patient-centred care**, i.e., care that makes patients central to the decision-making process at all stages of the clinical encounter. The defining features of the trafficking experience are often unpredictability and lack of control over events – particularly abuse and neglect. The loss of personal control over one's body and actions can have a significant influence on psychological health. It is

[17] Chang, J. C. et al., "Asking about intimate partner violence: advice from female survivors to health care providers", *Patient Education and Counseling*, vol. 59, no. 2, November 2005, pp. 141-147.

[18] Elliott, D. et al. (2005); Morrissey, J.P. et al., "Twelve-month outcomes of trauma-informed interventions for women with co-occurring disorders", *Psychiatric Services*, vol. 56, no. 10, October 2005, pp. 1213-1222; Huntington, N. et al., "Developing and implementing a comprehensive approach to serving women with co-occurring disorders and histories of trauma", *Journal of Community Psychology,* vol. 33, no. 4 (special issue on 'Serving the needs of women with co-occurring disorders and a history of trauma'), July 2005, pp. 395-410.

important for health providers to restore decision-making power to each individual as quickly and supportively as possible. Encouraging patients to participate in decisions throughout the clinical encounter helps prevent disempowering or re-traumatizing individuals who have been trafficked.

To accomplish this, it is necessary to train not only health care providers but also front-desk staff and medical assistants. They should be able to provide empathetic attention to patients' needs; they should also engage patients as partners in the health care delivery process in an inclusive and respectful manner. When providers encourage patients to participate in the development of the treatment plan, patients are more likely to feel that they are actively participating in their health care and may more likely to adhere to the prescribed treatment.

REQUIRED ACTIONS

Creating a clinical 'safe space'

- Aim to provide care in a **rights-based environment**:
    - The clinical environment is welcoming (trained staff and literature available in multiple languages).
    - Patients' rights are communicated clearly, verbally and in writing.
    - Patients' rights are respected at all times (for example, by ensuring systems are in place for protecting the confidentiality of patient files and providing private spaces for taking histories and for the physical exam).
- In order to approach patients from a consistently supportive and empowering stance, staff and provider training should focus on describing the impact trauma may have on people's behaviours, including ways in which patient's post-trauma reactions may manifest as anger, irritability and belligerence, or withdrawal and avoidance.
- Every encounter – even with clerical or medical support staff – can have a positive or negative impact on a trafficked person's health.
- Always strive to do no harm – inadvertent disclosures of trafficking history, breaches of confidentiality, judgmental comments or probing unnecessarily or in an insensitive manner about the patient's abuse history may contribute to individuals' mistrust and fear of health care settings. Providers can minimize the potential for re-traumatizing trafficked persons by having well-trained personnel and clear protocols

for supporting patients through acute and ongoing care. For example, it may be helpful to show patients how their records will remain in locked filing cabinets or access-coded computer files and to explain that a professional code of ethics prevents others from seeing these files without the patient's permission or a court order.

- Communicate slowly and clearly throughout the visit – this includes knowing how to respectfully assess patients' level of literacy and language comprehension, and how to use visual aids to ensure that an individual understands what is happening. This may also involve working with interpreters (*see action sheet 3*).
- Provide accurate and easy-to-understand information to patients regarding what will happen during the exam – before it happens – is crucial to keep patients informed and empower them to make well-considered decisions. This is particularly important given trafficked persons' lack of information and control during trafficking experiences.
- Be prepared to discuss **informed consent** using verbal, visual and written tools. Throughout the visit, providers should reiterate the voluntary nature of the clinical history-taking, exam and other services or treatment. Provide information both verbally and in writing; offer multiple opportunities for patients to ask questions.
- Always empower patients – clinical services are voluntary and patients have the right to decide what they are comfortable with (or not) based on a clear explanation of the procedures, exam or treatment beforehand. The right to refuse should be reiterated at regular and appropriate stages during complicated, lengthy or stressful procedures.
- Providers and office staff must understand the limits of confidentiality. Clinical settings have different mandates for reporting certain behaviours or situations, including suicidal or homicidal tendencies or reports of sexual abuse. Patients should be made aware of these limits to confidentiality prior to any delivery of clinical services. Additional information regarding confidentiality is provided in action sheets 9 and 16.
- Promote access to a network of resources to support patients' various needs. Providers should be familiar with the established procedures for contacting other health services and support organizations to address needs such as food, housing, shelter, education, legal aid and job-skills development (*see action sheet 10*).

Trauma-informed care relies on the above principles and actions, which aim to recognise and respond to the potential impact that patients' past experiences of abuse may have on their health and on their interactions in a health care setting.

REFERENCES AND RESOURCES

Chang, J. C. et al.
2005 "Asking about intimate partner violence: advice from female survivors to health care providers", *Patient Education and Counseling*, vol. 59, no. 2, November 2005, pp. 141–147.

Clark, H. and A. Power
2005 "Women, co-occurring disorders, and violence study: a case for trauma-informed care", *Journal of Substance Abuse Treatment*, vol. 28, no. 2, March 2005, pp. 145–146.

Elliott, D. et al.
2005 "Trauma-informed or trauma-denied: principles and implementation of trauma-informed services for women ", *Journal of Community Psychology*, vol. 33, no. 4 (special issue on 'Serving the needs of women with co-occurring disorders and a history of trauma'), July 2005, pp. 461–477.

Harris, M. and R.D. Fallot
2001 "Envisioning a trauma-informed service system: a vital paradigm shift", *New Directions for Mental Health Services,* vol. 89, Spring 2001, pp. 3–22.

Huntington, N. et al.
2005 "Developing and implementing a comprehensive approach to serving women with co-occurring disorders and histories of trauma", *Journal of Community Psychology,* vol. 33, no. 4 (special issue on 'Serving the needs of women with co-occurring disorders and a history of trauma'), July 2005, pp. 395–410.

Morrissey, J.P. et al.
2005 "Twelve-month outcomes of trauma-informed interventions for women with co-occurring disorders", *Psychiatric Services*, vol. 56, no. 10, October 2005, pp. 1213–1222.

Salasin, S.
2005 "Evolution of women's trauma-integrated services at the Substance Abuse and Mental Health Services Administration", *Journal of Community Psychology*, vol. 3, no. 4 (special issue on 'Serving the needs of women with co-occurring disorders and a history of trauma'), July 2005, pp. 379–393.

Action Sheet 2:
Culturally appropriate, individualized care

ACTION SHEET TWO

Action Sheet 2: Culturally appropriate, individualized care

RATIONALE

Men, women and children who have been trafficked are likely to have highly diverse backgrounds, and many social, cultural, economic, ethnic, or linguistic differences. With trafficking occurring on a global scale, people may have been trafficked from various countries and distant regions from where they receive care. Having been isolated and dominated by traffickers, individuals may have little understanding of where they have been while they were exploited and, once in a clinical setting, they may still not know where they are.

Culturally responsive care (also called 'cultural sensitivity' or 'cultural competence') refers to the provision of care that is attentive to the various ways people from diverse backgrounds experience and express illness and how they respond to care. In addition to language and literacy barriers, styles of communication, levels of mistrust, differing expectations of the health care system, gender roles and traditions and spiritual beliefs all contribute to how a person experiences illness and responds to care.

This action sheet builds on action sheet 1 on trauma-informed care to highlight the particular importance of individualized care that recognises the potential cross-cultural differences in patients' health and care needs. The guidance provided on culturally-sensitive care should also be considered together with action sheet 3, which focuses on the use of interpreters. Special approaches required for care of children and adolescents are described in action sheet 5.

People's personal, cultural and socio-economic background, level of education and events that happened to them while in the trafficking situation are very likely to influence their experience of illness and health and their expectations for health care. Individuals who have been trafficked may, for

example, have a profound fear of any formal setting, including the health care system. They may also have feelings of shame about being trafficked. Ignorance about the health care system in the destination location – about their rights to health services and patient confidentiality – commonly compounds people's fears and reluctance.

Language barriers and limited literacy levels are among the most difficult challenges of patient-provider communication. Language barriers, in particular must be quickly addressed through appropriate interpreting support, because misunderstandings and poor assumptions may contribute to misdiagnoses, poor adherence to treatment and poor outcomes overall (*see action sheet 3*). When there are significant differences in the backgrounds and knowledge levels of the patients and providers, it may be difficult for patients to relate their concerns and for providers to assess symptoms and needs. In particular, for women who might come from situations where abuse is 'normalised', the patient may tend to minimize the severity of her experiences.

Whenever possible, health care staff should receive training in trauma-informed care (*see action sheet 1*) to be able to provide support that recognises that while people may have different expressions of illness and need, they each require care that is non-judgmental, holistic, and patient-centred. As part of this approach, providers – and the clinical settings in which they work – must also be able to provide culturally and linguistically responsive care. Culturally appropriate communication requires adjusting one's practice and identifying appropriate resources, such as interpreters, to ensure that the patient can communicate needs and have those needs understood. Select interpreters carefully. Do not use casual bystanders, minors or those who have accompanied the trafficked person as interpreters (*see action sheets 3, 6 and 10*).

REQUIRED ACTIONS

To provide care that is culturally responsive

- Provide access to interpreters if there are language barriers (*see action sheet 3*). Have a system for identifying a patient's language needs, including in-person or telephone access to interpreters.
- Assess an individual's literacy level to ensure that information is conveyed in understandable ways. Some people respond well to information conveyed using visual aids.
- Don't make rapid or negative assumptions about individuals' reactions or behaviour. Consider possible cultural, social or personal reasons for individual reactions.

- Identify current social stressors, including housing, legal and financial constraints, and making appropriate and timely referrals to social services (*see action sheet 10*).
- Explore the patient's own understanding of their illness. See section on the explanatory model, below.
- Ensure that the patient's wishes for a provider of the same sex is respected when possible, as well as the patient's choices about whom they would permit to examine them.
- Recognize the importance of religious beliefs in a patient's recovery, as well as their understanding of the trafficking experience in the context of their religion and cultural beliefs.

Explanatory model (EM)

Exploring the patient's meaning of illness is important, particularly while evaluating the range of symptoms. The 'explanatory model' is a practical approach to assessing this that can help providers avoid cultural stereotyping. Because it is not possible to know everything about every culture, it is important to learn how to ask trafficked persons about their expectations for care. Suggested questions for eliciting the patient's perspective on their symptoms include:

1. What is the problem? How do you describe what is going on?
2. What do you think has caused your problem? How?
3. Why do you think the problem started when it did?
4. How does it affect you?
5. What worries you most? (Severity? Duration?)
6. What kind of treatment do you think you should receive? (Expectations?)

Provider-patient negotiation

Once you have explored the patient's concerns and elicited the patient's perspectives on their illness (their explanatory model), utilizing the support of interpreters and visual aids as necessary, work with the patient to develop a treatment plan that is understandable, feasible and sustainable.

Creating a mutually acceptable plan that acknowledges the background and individuality of each patient for next steps requires shifting paradigms from 'making a patient *comply* with treatment' to '*supporting* patients in their healing and recovery'. Planning treatment in cooperation with the trafficked person conveys responsive support and a sense of empowerment: it is a signal to the patient that you have listened and understood the patient's concerns.

A mutually acceptable treatment plan is more likely to be embraced by the patient and result in improved outcomes. Such negotiations may be constrained when clinical encounters are brief (e.g., if the patient is in detention or in transit) or by a patient's lack of health insurance or resources to pay for care.[19]

While you may be concerned about the additional time involved in communicating and negotiating with patients, particularly those from diverse backgrounds, evidence suggests that using these strategies actually shortens the amount of time ultimately required to diagnose and treat. Patients are more engaged and open to communication when providers offer culturally responsive care. Using these techniques to take medical histories from those who may express their illnesses differently and to plan treatment for individuals familiar with other medical practices is likely to take less time and be more effective than guessing, missing diagnoses and failing to get adherence to care.

REFERENCES AND RESOURCES

Brach, C. and I. Fraser

2000 "Can cultural competency reduce racial and ethnic disparities? A review and conceptual model", *Medical Care Research and Review*, vol. 57, no. 4 suppl., December 2000, pp. 181–217.

Carrillo, J. E. et al.

1999 "Cross-cultural primary care: a patient-based approach", *Annals of Internal Medicine*, vol. 130, no. 10, 18 May 1999, pp. 829–834.

Culhane-Pera, K.A. et al.

1997 "A curriculum for multicultural education in family medicine", *Family Medicine*, vol. 29, no. 10, November-December 1997, pp. 719–723.

[19] Health is a recognized human right and many countries strive to provide universal access to health care for victims of violence, those in need of emergency care and vulnerable groups. However, in reality it can be very complicated to provide the necessary medical and mental health care for a victim of trafficking who cannot pay and may not have the identity documents required to access existing social services. Coordination with other service providers, particularly those with experience in meeting the many non-health needs of trafficked persons, is essential (*see action sheet 10*).

Davis, D.A. et al.
1995 "Changing physician performance: a systematic review of the effect of continuing medical education strategies", *Journal of the American Medical Association*, vol. 274, no. 9, 6 September 1995, pp. 700–705.

Day, J.H. et al.
2006 *Risking Connection in Faith Communities: A training curriculum for faith leaders supporting trauma survivors*, Sidran Institute Press, Baltimore, Maryland, USA, 2006.

Denoba, D. L. et al.
1998 "Reducing health disparities through cultural competence", *American Journal of Health Education*, vol. 29 (5 Suppl.), pp. S47–S58.

Joos, S. K. et al.
1996 "Effects of a physician communication intervention on patient care outcomes", *Journal of General Internal Medicine,* vol. 11, no. 3, pp. 147–155.

Action Sheet 3: Working with interpreters

ACTION SHEET THREE

Action Sheet 3:
Working with interpreters

RATIONALE

Accurate communication is essential to providing quality, rights-based health care to trafficked persons. Due to the nature of trafficking, many trafficked persons are offered care when they are away from their home country by health care providers who do not speak their language. (Persons trafficked within a country may also face language barriers.) In these situations, care is often facilitated by interpreters. This action sheet focuses on communication via interpreters, while acknowledging that there are also cultural, gender, age-related and other sensitivities (e.g., literacy, disability, culture or capacity) that may affect communication with trafficked persons. (*See action sheets 1, 2, 5, 9, and 10 for more information on other topics related to communication.*)

Working with interpreters in health care settings can be enjoyable as well as challenging. Such topics as abuse and psychiatric symptoms may be more difficult to discuss through interpreters. Some health care providers report greater detachment from their patients and feel less effective in their work or frustrated because treatments take longer. However, in many cases, care strategies can be enhanced by working with an interpreter who fosters better communication and engagement with the trafficked person, and who helps the provider obtain accurate information within a cultural understanding of the individual (*see action sheet 2*).

Interpreters in health care settings can have various roles. When working with an interpreter it is worth considering the potential roles that he or she might take. This role will depend on the care setting, the patient's needs and the professional experience of the interpreter and the clinician. The following roles have been identified for interpreters:

- **Interpreter** who provides a neutral and impartial service.
- **Cultural broker** who explains and gives cultural and contextual understanding to the health care provider and the client.
- **Advocate** who represents the patient's interests.
- **Intermediary or conciliator** who resolves conflicts between the patient and the health care provider.
- **Link worker** who helps the clinician identify unmet needs of the patients and provides support to the patient.
- **Bilingual co-worker** who takes on a more involved therapeutic role in addition to providing translation.

REQUIRED ACTIONS

Before beginning work with an interpreter there are some preparations that you might wish to consider to facilitate your work with the trafficked person and the interpreter.

Selecting an interpreter

Selecting an interpreter is an extremely important step that is crucial to the safety and well-being of the trafficked person as well as that of the health provider. The following basic cautionary notes should be followed:

- Do not permit individuals who state that they are friends, family, employers or associates of the trafficked person, or have accompanied them, to interpret for them. These persons may be part of the trafficking situation or may provide information to the traffickers.
- Do not use minors or children of the trafficked person as interpreters.
- Do not permit someone from the same village or local community (either in the home or destination location) to interpret for the individual. This may inhibit the trafficked person from speaking freely; information provided may turn into 'gossip' or otherwise stigmatize the patient.
- Do not permit someone to interpret who may discriminate against the ethnic group or social class of the individual or who may be disgusted by potential past events (e.g., sexual abuse or prostitution).
- Use special precautions when interviewing children, giving special consideration to the possibility that the individual acting as their guardian may be involved in their trafficking.

For the patient encounter

- Find out the trafficked person's first language and dialect and if he or she speaks any other languages.
- Take into account the nationality, religion and gender of the patient (*see action sheet 2*).
- Book longer sessions that will allow time for interpretation.
- Try to find a way to explain to the trafficked person that you are seeking interpreting services.
- Try to find a way to encourage the trafficked person to let you know if he or she becomes uncomfortable with the selected interpreter for any reason.

For the interpreter

- Notify the interpreter or his or her agency of the subject matter (e.g., confidential medical discussions that may cover violence, rape, etc.). Be aware that if you don't do that you might find that the interpreter is reluctant or feels uncomfortable with the subject matter.
- Because confidentially is extremely important when working with trafficked persons (*see action sheets 7 and 9*), you may want to consider establishing a formal mechanism which makes clear the role of the interpreter and their responsibilities to maintain confidentiality. An example of this could be a standard operating procedure agreed to with the agency or individual.
- In order to establish a relationship in which the trafficked person feels safe working with you and the interpreter, it is important to carry out all consultations with the same interpreter if more than one meeting is possible. When arranging for an interpreter it is important to ensure that the agency and the individual interpreter know that you are planning to use the same interpreter in your future sessions.
- Warn the interpreter not to give out personal contact details and against disclosing any information to others about the trafficked person.

During the consultation

- Allow some time for the interpreter and the service user to introduce themselves.
- Introduce the interpreter; confirm that the interpreter is a professional bound by the agency's policy for confidentiality.
- Try to ask the trafficked person whether there are any safety or cultural issues that might inhibit.
- Try to avoid specialist terminology.
- Do not expect all meanings and thoughts to be conveyed perfectly.
- Avoid using proverbs and sayings because these are often culturally influenced and tend to lose their meanings in translation.
- You may need to slow down your pace. If your sentence is long, summarize it for the interpreter. Check that the interpreter is clear about your meaning.
- Provide sufficient breaks during the interview to allow the interpreter to carry out his or her job.
- Try not to show too much collegiality with the interpreter and avoid discussing issues with the interpreter that do not require interpretation in front of the individual that may make the patient feel isolated, left out or fearful.
- Try not to leave the interpreter alone with the patient. Interpreters may be put under pressure in these circumstances to assist the client in matters for which they were not contracted.
- Try to observe whether the interpreter is showing sympathy or condescension.
- Observe whether the interpreter is permitting the individual to speak for him or herself or whether the interpreter is taking control and **explaining for** the individual.

After the consultation

- Ask the interpreter to clarify cultural issues and seek meanings for points that were not clear during the meeting.
- Remember that the interpreter is not necessarily trained to work with traumatized individuals and is unlikely to have supervision or peer support. It is important to encourage the interpreter to discuss emotions and difficulties he or she encountered with regard to the session.

- If you have serious doubts about the interpreter's conduct, discuss this with the interpreter. If the individual is employed by a service, provide feedback to the employer.
- Reflect on how to maintain good working alliances with interpreters who have demonstrated that they can be trusted.

REFERENCES AND RESOURCES

Raval, H.

2005 "Being heard and understood in the context of seeking asylum and refuge: communicating with the help of bilingual co-workers", *Clinical Child Psychology and Psychiatry*, vol. 10, no. 2, 1 April 2005, pp. 197–216.

Tribe, R. and H. Raval (Eds.)

2003 *Working with Interpreters in Mental Health*, Brunner-Routledge, Hove, United Kingdom and New York, NY, USA, 2003.

Action Sheet 4: Comprehensive health assessment

Action Sheet 4: Comprehensive health assessment

RATIONALE

A comprehensive health assessment is important because many trafficked persons will experience a range of abuses and other health risks that result in co-occurring conditions.[20] Although patients may present with specific symptoms that seem to be the focus of the clinical encounter, patients are likely to require a comprehensive medical and mental health assessment because the high likelihood of co-occurring disorders. Knowing the multiple health problems that trafficked persons face may assist you in using a systematic care approach to address patients whose medical problems are often complex. Depending on the trafficking context, trafficked persons may have experienced a range of abuse, violence and other health risks. For example, a child exploited sexually will also likely have experienced physical abuse, poor nutrition and sleep deprivation. Careful 'head-to-toe' assessments should be developmentally appropriate. Examination of children should be conducted by providers who are comfortable with the care of abused children and with forensic examination whenever possible (*see action sheets 5 and 12*). Infectious diseases are discussed in detail in action sheet 15.

Many trafficked persons may have had poor health prior to being trafficked, because factors such as poverty and poor living conditions, which increase susceptibility to being trafficked, also predict baseline poor health.[21],[22] Many patients may have additional multiple active medical problems (e.g., asthma, diabetes, anaemia and parasitosis) that are likely to have been exacerbated in the context of trafficking. Living in overcrowded

---

[20] This action sheet assumes that health care providers are aware of the basic principles of trauma-informed care (*see action sheet 1)* and cross-cultural care (*see action sheet 2*), appropriate identification and safety assessments have been conducted (*see action sheet 7*), and referral options for legal advocacy and social services have been identified (*see action sheet 10*).

[21] Beyrer, C., "Is trafficking a health issue?" The Lancet, vol. 363, no. 9408, 14 February 2004, p. 564.

[22] Zimmerman, C. et al. (2003).

situations, with poor nutrition, restricted lifestyles, and limited access to health care all contribute to poor health and greater severity of symptoms.[23] Providers should try to learn about the local epidemiology of disease patterns in a patient's country of origin, the transit environment and the trafficking destination to ensure that such common disease conditions as dengue, malaria, tuberculosis and others are not missed.

Physical and mental health symptoms in a trafficked person are often intertwined, in particular during the acute period during or immediately after the trafficking situation. Somatic symptoms without a clear organic cause are not uncommon but require thorough assessment to ensure that underlying organic causes are not missed. In trafficked persons, their many symptoms often intersect with – and are exacerbated by – post-traumatic reactions. Somatic and behavioural symptoms related to a history of trauma may include anorexia, chronic fatigue, chronic headaches, chronic pain, dizziness, emotional numbness, hostility, hyper-arousal, hyper-vigilance, irritability, lack of motivation, memory problems, poor concentration, re-experiencing traumatic events and sleep disorders. These post-traumatic symptoms contribute to the overall poor health status of trafficked persons (*see action sheets 1 and 12*).

Medical assessments of trafficked persons should be conducted based on the guiding principles (*see chapter 3*) and using the techniques described for trauma-informed and patient-centred care (*see action sheets 1 and 2*). Confidentiality and privacy are of the utmost importance in cases involving trafficking. The history and exam should take place alone with the patient (i.e., with health provider and chaperone when necessary, but with no other accompanying persons). Note that in cases where a referral has been made from a support service to a medical clinic or where a clinic has 'patient advocates' or 'cultural mediators[24]' available, it is desirable to have these type of support persons at health encounters, particularly first encounters. As discussed, persons who have been trafficked are likely to mistrust others and find it difficult to express their concerns with new professionals. While some patients may state that they prefer their partner or family member to stay, it is crucial to have some private time with the patient, who may be scared to ask the accompanying party to leave the room. It may be necessary to state that it is the clinic's policy to see all patients individually and in private at some point during the visit, in order to afford everyone the same kind of privacy.

---

[23] Wolffers, I. et al., "Migration, human rights, and health", *The Lancet*, vol. 362, no. 9400, 13 December 2003, pp. 2019-2020.

[24] Hjermov, Birgit, *Cultural Mediation at the Workplace – an Introduction*, 2004.

REQUIRED ACTIONS

Goals for the clinical encounter

- Create a safe space for the patient where care is individualized, supportive, non-judgmental and integrated (*see action sheets 1 and 2*).
- Describe to the patient the reasons for the exam, how the exam will be conducted, how the results will be communicated and who will have access to the results.
- Conduct a comprehensive health assessment, because this clinical encounter may be the only contact the trafficked person has with the health care system (e.g., individual may return to the trafficking situation, may be in detention or in transit). This includes a thorough and systematic review of symptoms, a careful 'head to toe' exam and appropriate laboratory testing, recognizing that trafficked persons present with conditions that are co-morbid with other complex and chronic disorders (*see action sheet 5 for specific considerations when examining children and adolescents*).
- Focus the clinical encounter as much as possible on those medical problems identified by the patient. Questions that simply serve the curiosity of the provider are not appropriate.
- If possible, receive training in mental health assessment and/or have access to a mental health provider to offer a detailed assessment necessary to identify specific mental health diagnoses and treatment needs. The impact of traumatic experiences on patients' symptoms, adherence to treatment and outcomes cannot be overstated (*see action sheets 1 and 12*).
- Try to ensure that there is a consistent, certain and secure communications mechanism to inform patients of the results of any testing and a convenient means for patients to receive ongoing care, including preventive care.
- Confirm that patients are connected to resources and services to address multiple needs whenever possible, including food, shelter, legal advocacy, mental health support, education and job skills development, which are all crucial to the health, safety and well-being of trafficked persons (*see action sheet 10*).

The thorough, systematic review of symptoms

While many vague somatic concerns, chronic pain and fatigue may not have an underlying organic cause, when conducting an initial medical assessment you must have a high degree of suspicion that organic conditions exist. Resist the urge for premature closure of the patient encounter. This means avoiding quick conclusions about the causes of a patient's complaints without conducting a thorough evaluation of presenting symptoms. For example, a headache may be connected to distress or depression, or may be a result of a blow to the head.

You may become frustrated with patients who have been trafficked, because their reporting of events or symptoms seems vague. In the context of multiple traumatic experiences, poor recall of details is not uncommon. Recognize that an unclear or inconsistent history does not mean that the patient is being obstructionist or difficult, but may instead reflect the patient's reactions to abuse and violence.

In addition to the review of symptoms that is a standard of care among providers of Western medicine, the following outlines some **additional** history questions to include in the assessment:

Head/eyes/ears/nose/throat

- Any history of head trauma? Examine the skull for bruises, depressions or healed lacerations
- Exposure to loud noises?
- Frequent headaches?
- Any pharyngeal trauma (lacerations, tears)?
- Dental or gingival pain?[25]
- Any visual changes? Sudden or gradual?

Neck

- Any history of strangulation?

Cardiovascular

- Any trauma to the chest?

Respiratory

- Any exposure to chemicals, fumes, asbestos or other occupational exposures?
- Possible exposure to TB? (Living conditions? Number of people sharing one bedroom? Ventilation?)

Gastrointestinal

- Abdominal trauma?

---

[25] Poor oral health is a major co-morbid factor in poor general health and malnutrition.

- Chronic diarrhoea? Constipation? Visible parasites in stool? The first two symptoms may be related to mental health (*see action sheet 12*).

Genitourinary

- Forced sex, or sexual trauma that includes foreign objects?
- Enuresis or encopresis (a potential result of sexual abuse)?

Musculoskeletal

- Repetitive and non-repetitive work-related injuries?
- Fractures?
- History of physical abuse such as burns? Contractures?
- Vitamin D deficiency?

Neurological/behavioural

- Seizure activity (may also need to consider pseudo-seizures)?
- Sleep disorders (inability to fall asleep, frequent awakenings, nightmares)?
- Any history of head trauma?

Nutrition

- Any nutritional deficiencies (food intake, content)?
- Disordered eating (e.g., anorexia or bulimic behaviour)?

Dermatological

- Scabies, lice, scant or fine hair (may indicate a nutritional deficiency)?
- Burns (e.g. cigarette burns, scalds from hot water)?
- Impetigo and fungal infections?

Conducting a patient-centred physical exam

Conduct a careful, complete physical exam from head to toe. All physical injuries must be documented, and when appropriate, through photo documentation. As is the case with all victims of violence and torture, describe the exam that will be conducted before a patient undresses and then explain each step of the exam as you carry it out, always giving the patient the option to refuse at any point. It is useful to warn the patient about procedures that may be invasive or potentially painful.

Patients may not always tell you all of their complaints or respond to questions honestly out of fear, mistrust or shame. Be vigilant to look for signs of other medical conditions that were not mentioned in the medical history.

In addition, be aware that the physical exam may trigger flashbacks in some patients. This may involve the patient 'zoning out' (appearing to be in a different place and not responding to questions), hyperventilation

and near-syncope. It may be helpful to explain before proceeding with the examination that it may cause the patient to recall prior victimization, and then to check regularly about the well-being of the patient throughout the exam. For example, some trafficked persons may have been exploited through pornography, so particular care should be taken if it is necessary to photograph lesions.

Forensic exam

If the medical assessment might be used for prosecution, and in particular, if evidence collection is necessary to prove assault (particularly rape), a specially trained health provider should carry out a forensic exam. Given the difficulties of good evidence collection and the challenges of avoiding re-traumatizing patients, a clinic with several providers may want to designate one provider to receive additional training in counselling and examining victims of sexual assault and collection of forensic evidence, depending on the procedures and laws of your country. In particular, providers examining children should have additional training in child abuse evaluations, including strategies for taking a child's medical history and documentation (*see action sheets 5 and 12*). When appropriate, and according to local laws and procedures, collect minimum forensic evidence:

- Local legal requirements and laboratory capabilities determine if and what evidence should be collected for use in criminal prosecution, and by whom. If available, it is best to have forensic evidence collected by specially trained forensic professionals. Health workers should not collect evidence that cannot be processed or that will not be used.
- Counsel the survivor about taking evidence for criminal prosecution. It is essential that you know in advance where to refer victims of sexual violence safely (*see action sheet 10*). Assure the trafficked person that the information will only be released to the authorities with their consent.
- For cases of sexual violence, a careful written record should be kept of all findings of the medical examination, including the state of the patient's clothes. The medical chart may be part of the legal record and in most locations can be submitted as evidence in a court case.

Sexual trauma and reproductive health (*see action sheet 13 for more details*)

While human trafficking does not always involve sexual exploitation, in many locations women and children, who are commonly subject to sexual violence when trafficked, make up a significant portion of trafficking cases.

- Where medically indicated, perform a thorough pelvic exam for women who have been trafficked and a genital (including anal) exam for men, if the patient consents. Offer patients the option of having a provider of the same sex if they prefer and of having a professional chaperone in the exam room. In some cases, the patient may never have received an internal exam and it is therefore especially important to explain procedures, step-by-step.
- Include detailed questions about reproductive and sexual health in the medical history.
- Follow up any external or internal evidence of trauma found during the physical exam with laboratory testing. Collect samples (urine, cervical, anal) to test for sexually transmitted infections.
- Offer testing for pregnancy as well as HIV and other sexually transmitted infections when laboratory capability allows. Offer appropriate pre-test counselling and a specific follow-up plan for notifying and counselling patients about results. This should include treatment planning and appropriate referrals if testing is positive. Offer presumptive treatment for sexually transmitted infections as indicated.

Nutritional deficiencies

Trafficked individuals are often subject to severe restrictions in their movement and constraints in their access to food. Their access to fresh produce, intake of adequate protein and minerals and exposure to the sun (for vitamin D) may be severely limited, depending on the type of exploitation. Histories of substance abuse, often co-occurring with other clinical problems, compound the problem of poor nutrition. It is therefore necessary to take a detailed history of nutritional intake as well as look for evidence of nutritional deficiencies (e.g., gum disease, tongue and skin changes) during the physical exam.

Laboratory testing should include at a minimum a complete blood cell count with mean corpuscular volume. When available, iron count, total iron binding capacity and vitamin B_{12}, folic acid, calcium, phosphorous, and 25-hydroxyvitamin D (25-OH vitamin D) levels may help guide treatment.

Oral health

Poor oral health (including caries, gingival disease and abscesses) is a common and often very painful co-morbid factor in the poor health of trafficked persons. Pharyngeal trauma from forced oral sex can further complicate this picture. Poor oral health can contribute to poor nutrition, chronic headaches, disturbed sleep and gastrointestinal problems.

Head injury

Among the most common symptoms reported by individuals who have been trafficked are chronic headaches, with many patients describing multiple head traumas associated with loss of consciousness and relaying stories suggestive of concussion and post-concussion syndromes. With chronic headaches, the question of the need for neuro-imaging arises. If resources exist, and when history and exam suggest an intracranial process, head imaging is appropriate. However, a careful history which looks at migraine characteristics – aura, unilateral pain, nausea, photophobia, phono-phobia – and visual changes, seizure activity, loss of coordination and imbalance, as well as a thorough neurological exam are generally sufficient to rule out such significant underlying pathologies as chronic subdural haematomas.

Seizure disorders, pseudo-seizures and dissociation

In addition to non-specific symptoms of headaches and dizziness, trafficked persons also report experiences of falling, passing out and not remembering things. In light of the traumatic head injuries that some patients may have experienced, a thorough history and neurological examination are crucial. Specifically, asymmetries in exam, ataxia (e.g., loss of balance or disordered gait) and proprioceptive dysfunction may indicate a serious underlying cause. More often, however, the overall neurologic examination is normal, without evidence of deficits, but the patient continues to have near-syncope or actual falling, sometimes with what appears to be seizure activity (*see action sheet 12*). Apparent dissociative states and pseudo-seizures should first be evaluated for organic causes before assuming that these are post-traumatic reactions.

Occupational health

Trafficked persons may have been exploited for labour in a variety of industries, such as garment factories, meat processing plants, construction, agriculture and domestic servitude.

- Document the types of work performed to help guide diagnosis of injuries.
- Consider the possibility of repetitive work injuries if a patient is suffering from persistent pain.
- Examine skin carefully for burns, wounds and other occupational injuries.
- Consider the possibility of exposure to toxins and other dangerous materials in the workplace that may contribute to headaches or breathing difficulties.
- Check vision because eyesight may be compromised from working in dimly lit workplaces.
- Ask about ventilation in the work environment; poor ventilation may lead to respiratory problems.

Common infections (*see action sheet 15 for more details*)

With the emphasis on the patient's presenting symptoms, you may inadvertently overlook other common communicable diseases associated with the patient's travel history and exposures, including airborne, waterborne and mosquito-borne illnesses. You should have easily accessible information on local epidemiology for all the places in which a trafficked person has been. Websites such as http://www.cdc.gov/travel/default.aspx offer up-to-date information on outbreaks, changes in antibiotic resistance and endemic areas.

Preventive care, immunizations and presumptive care

While the scope of the physical and mental health needs of trafficked persons is often overwhelming, this systematic approach offers a methodical way to assess patients with complex medical problems. Because of the multitude of acute concerns, preventive care – including blood pressure screening, eye exams, immunizations and cancer screening (testicular and cervical, as well as breast and colorectal for older patients) – may be overlooked.

The question of presumptive care, or the treatment of diseases without test results, is a controversial area in the care of persons who have been

trafficked. If it is unlikely that the trafficked person can be contacted after the initial visit to discuss test results, some providers will opt to treat patients presumptively for common diseases such as chlamydia, in particular if the treatment costs less than the laboratory test. Consider having policies and procedures in place to determine under what circumstances such presumptive care would be delivered, if at all.[26]

Because this health assessment may be the only clinical encounter for the patient, consider offering comprehensive preventive care if resources exist for this. There should be a protocol in place for patients to receive results and be directed to follow-up care as needed.

REFERENCES AND RESOURCES

Beyrer, C.
2004 "Is trafficking a health issue?" *The Lancet,* vol. 363, no. 9408, 14 February 2004, p. 564.

Hjermov, B.
2004 *Cultural Mediation at the Workplace – an Introduction*, 2004.

United Nations High Commissioner for Refugees
1999 *Reproductive Health in Refugee Situations: An inter-agency field manual*, UNHCR, Geneva, 1999.

Wolffers, I. et al.
2003 "Migration, human rights, and health", *The Lancet*, vol. 362, no. 9400, 13 December 2003, pp. 2019-2020.

World Health Organization
2005 *Sexually Transmitted and Other Reproductive Tract Infections: A guide to essential practice*, WHO, Geneva, 2005.
1997 *Syndromic Case Management of Sexually Transmitted Diseases: A guide for decision-makers, health care workers and communicators*, WHO Regional Office for the Western Pacific, Manila, 1997.

[26] See World Health Organization, *Syndromic Case Management of Sexually Transmitted Diseases: A guide for decision-makers, health care workers and communicators*, WHO Regional Office for the Western Pacific, Manila, 1997 and United Nations High Commissioner for Refugees, Reproductive Health in Refugee Situations: An inter-agency field manual, UNHCR, Geneva, 1999.

Zimmerman, C. et al.
2003 *The Health Risks and Consequences of Trafficking in Women and Adolescents: Findings from a European study*, London School of Hygiene and Tropical Medicine, London, 2003.

Action Sheet 5: Special considerations when examining children and adolescents

ACTION SHEET FIVE

Action Sheet 5: Special considerations when examining children and adolescents

RATIONALE

Children and adolescents are frequent targets of traffickers; they may have been trafficked by a family member or acquaintance or may have been indirectly trafficked when they accompany parents who are trafficked or when entire families are trafficked together. Children and adolescents require physical and mental health assessments according to their developmental stage. At the same time, the developmental age of a child may not be congruent with that child's physical age, for example, due to chronic abuse or deprivation. While providers must obviously assess children to determine urgent health needs, they must also pay particular attention to health consequences of trafficking that may affect a child's long-term health and development.

Children who have been exposed to the abuse, trauma and deprivation of trafficking are likely to have a wide range of care needs. They may have been physically and sexually abused; they may have experienced or witnessed traumatic events or been forced into forms of labour exploitation subjecting them to dangerous or life-threatening health hazards.

In child trafficking cases, a young person's mental and emotional health is of particular concern. Severe and prolonged stress can cause cognitive and emotional developmental delays and possibly developmental regression. If forced to participate in adult activities, young persons may also have adopted behaviours, perceptions or language seemingly beyond their age. Early psychological trauma or syndromes may be predictors of long-term psychological morbidity and future risk-taking (*see action sheet 12*). Children who have been chronically undernourished may also risk long-term cognitive and behavioural problems. Children's mental, emotional and social health needs will require age-appropriate health care and various other forms of support designed for young persons.

The health provider may be the first person to identify a child or adolescent as possibly trafficked – and therefore their first and potentially only advocate until the child is referred to a broader system of care.

REQUIRED ACTIONS

Providing care for young persons requires careful consideration of their age, capacity and developmental needs. The following are key aspects of care that should be considered when a young person has been trafficked or exposed to a trafficking situation:

- Refer to general information on the comprehensive health examination (*see action sheet 4*).
- Refer to guidance on abused or deprived children (*see reference list at the end of this action sheet*).
- Give every child and adolescent an age-appropriate medical and developmental assessment. If a paediatric specialist is not available and it is likely that children and adolescents will be patients, consider accessing the services of a paediatrician or designating one person in the health structure to receive special training in caring for children.
- Encourage children, adolescents and family members, as appropriate, to participate in decision-making as much as possible. Explain to them each step of the evaluation. However, remember that family members or individuals seen as parental figures by a child may be involved in trafficking the child. This should always be kept in mind and assessed when working with trafficked children and adolescents.
- Designate a care coordinator or lead case manager for each child or group of children, if possible. The care coordinator can bring together the different forms of care a child requires and help prevent redundant investigations. An individual identified as a lead case manager can establish a rapport with a child and act as her or his advocate.
- Establish a short- and long-term plan to ensure that there is continuity of care based on the child's medical and social needs (*see action sheets 12 and 14*).
- Keep detailed and up-to-date records for child patients. This is particularly important because children's medical needs can change rapidly. Keeping detailed records can help prevent unnecessary or dangerous duplication of medical interventions or prescriptions (e.g., x-rays, vaccinations, medications that may be counter-indicated).

- Coordinate with other service providers to address the multiple special needs of separated and orphaned children (*see action sheet 10*). Make identification bands for each child. He or she needs to be placed immediately in a safe, non-threatening environment, such as a foster home, to ensure protection from further abuse. Every effort should be made to reunite children with their family if this is appropriate, safe and possible. If not, children should be paired with appropriate adult caregivers who are well-informed about the child's needs in order to establish continuity of mental and emotional support. This caregiver, teamed with the medical care coordinator, helps serve as an advocate for the child. In many countries, child services have established mechanisms to place children.
- Be proactive to ascertain whether family members or self-designated guardians have been involved in the trafficking or any abuse or neglect of the young person before involving these persons in their care.
- Keep together families that have been rescued together when safe and possible.

Examination and review of systems[27]

In addition to a standard paediatric review of systems and physical examination, there are trafficking-specific issues that require attention. Please see action sheet 4 for general information on review of the following systems in any trafficked person: oral health, ear/nose, neurological, respiratory, gastrointestinal, genitourinary, reproductive health, musculoskeletal, dermatological, nutrition, laboratory studies, and forensic examination. The following is *additional* information for children and adolescent trafficked persons.

Neurological

Infants should have an ophthalmological exam for retinal haemorrhages, which may indicate physical abuse or 'shaken baby' syndrome.

Reproductive health

Assess every child using a focal examination for trauma and infection, and testing for specific sexually transmitted infections. Girls should receive assessments related to their reproductive health, including menstrual history and possible pregnancy. Boys should be examined for genital and anal trauma. Care must be taken not to re-traumatize the adolescent; their refusal

---

[27] See action sheet 4.

must be respected. Every examination should be done by a practitioner of the gender requested by the patient. The practitioner should talk the patient through every step with appropriate explanations and expectations. Requests by a child or adolescent to delay or suspend an examination should be responded to appropriately and with patience, even if this means delaying or terminating the exam (*see action sheet 12*).

Nutrition

Children are more dependent than adults for security of food and safe water. Since food is often restricted in a trafficking situation, vitamin, protein and mineral deficiencies are common. Poor nutrition impacts the growth and development of children more dramatically than that of adults. Therefore, health professionals should assess each child's nutritional status against international standards (e.g., World Health Organization standards) and immediately start providing for nutritional needs. Assessment includes weight-for-height measurements or a mid-upper arm circumference. Nutritional deficiencies put children at greater risk of contracting certain infectious diseases (e.g., vitamin A deficiency). Hair color and presence of oedema should also be noted.

Infectious diseases

Infectious diseases are very common among children and can present differently from similar diseases in adults. Every practitioner must have a high degree of suspicion for infectious diseases in every child and adolescent (*see action sheet 15*).

Substance abuse

This must not be overlooked in children and adolescents. A toxicology screen can help identify drugs of abuse and poisons that are either intentionally taken or administered to a child. It is important to rule out toxic substances as an organic cause of altered mental status, behavioural abnormalities and other medical conditions. Withdrawal from some substances can present as a medical emergency (*see action sheet 11*).

Forensic examination

Health professionals should have specific training in evaluation of violence towards children that is appropriate for the patient's age and developmental stage. Sexual assault is common among trafficked children and therefore needs to be addressed urgently, but in a delicate way. If any evidence is likely to be used in any legal proceedings, a professional trained in collection of forensic evidence should perform the examination. Appropriate national or local guidelines for collection, reporting and chain of custody of forensic evidence need to be followed. It is also essential that sexually

assaulted or exploited children and adolescents receive mental health care as soon as possible (*see action sheet 12*).

Treatment

- Administer immunizations according to each country's expanded protocol for immunizations. You may consider other preventive measures including hepatitis B immunoglobulin (HBIG), post-exposure prophylaxis for HIV and emergency contraception (*see action sheets 13 and 15*).
- Consider prescribing a vitamin and mineral supplement for all children in the acute care setting with continued supplementation for those who demonstrate deficiencies. Infants with mothers should continue to breastfeed, unless contraindicated.
- Referral to a dentist, if necessary, should be done rapidly to reduce potentially serious complications.
- When dosing medication, it is important to remember that medicine is often dosed according to weight. If paediatric dosages are not available, it is important to determine the correct dosage of adult medication. Children metabolize medicines differently from adults and some medicines are contraindicated in children because they are harmful to a child's physical development.
- Treatment protocols for such infections as HIV and tuberculosis are highly specific to children; such protocols should be reviewed, and a paediatric infectious disease specialist consulted if possible.

Legal considerations (*see action sheet 16 for more details*)

- Be aware of local laws pertaining to the treatment of children, consent to test for and/or treat specific conditions in children (e.g., the requirements to obtain consent for treatment of a minor), as well as legal requirements that may cover reporting of certain conditions (e.g., child abuse) and diseases in children.
- Adolescents in some places may be regarded as emancipated minors, which means that despite being younger than the legal age of adulthood, they have complete autonomy to make decisions regarding consent, refusal and direction of care.

REFERENCES AND RESOURCES

Delaney, S. and C. Coterrill

2005 *The Psychosocial Rehabilitation of Children who have been Commercially Sexually Exploited: A training guide*, ECPAT International, Bangkok, Thailand, 2005.

International Organization for Migration

2007 *The IOM Handbook on Direct Assistance for Victims of Trafficking*, IOM, Geneva, 2007, pp. 206–213.

The National Child Traumatic Stress Network

www.nctsn.org

United Nations

1989 Convention on the Rights of the Child, adopted on 20 November 1989 by General Assembly Resolution 44/25, entry into force 2 September 1990.

United Nations Children's Fund

2005 *Manual for Medical Officers Dealing with Child Victims of Trafficking and Commercial Sexual Exploitation (Manual for Medical Officers Dealing with Medico-Legal Cases of Victims of Trafficking for Commercial Sexual Exploitation and Child Sexual Abuse)*, UNICEF and the Department of Women and Child Development, Government of India, New Delhi, 2005.

2006 *Reference Guide on Protecting the Rights of Child Victims of Trafficking in Europe*, UNICEF, 2006.

World Health Organization

2004 *Antiretroviral Drugs for Treating Pregnant Women and Preventing HIV Infection in Infants: Guidelines on care, treatment and support for women living with HIV/AIDS and their children in resource-constrained settings*, WHO, Geneva, 2004.

2002 *Guidelines for Medico-Legal Care for Victims of Sexual Violence*, WHO, Geneva, 2003.

Action Sheet 6:
What to do if you suspect trafficking

ACTION SHEET SIX

Action Sheet 6: What to do if you suspect trafficking

RATIONALE

The health care system is in a unique position to provide protection to trafficked persons. Health providers may treat persons who have already been identified by assistance organizations or police; however, there may be times when a provider receives a patient that has not been formally identified as a trafficked person. A provider may detect signs that the individual has been trafficked in the past or may still be in a trafficking situation.

Situations where trafficked persons have not yet been identified could happen in settings such as a general clinic or private practice; through care for individuals in immigration detention, at a reception facility or in prison; or via outreach services for sex workers or migrant populations. In each case, it is important to safely assess the situation and decide on appropriate response options (*see action sheet 17*).

Reacting to a person who is still in the trafficking situation requires well-considered responses that prioritize the safety of the individual, the health provider and possibly those near to them (*see action sheet 7*). Although trafficked persons caught in the trafficking situation are generally kept away from potential sources of assistance, given the nature of the abuse and exploitation associated with trafficking, it is not unusual for trafficked persons to become ill or injured, limiting their usefulness and decreasing their profitability to the trafficker. Because of someone's need for medical attention, and because traffickers may consider health providers to be less of a threat compared to other service providers, traffickers may seek medical care for those they have trafficked.

This action sheet focuses on what the health provider needs to consider in a clinical setting when encountering patients whom they suspect may have been trafficked or otherwise caught in an abusive or exploitative situation

(e.g., smuggling or abusive labour exploitation), but who are not yet linked to any protection services. It outlines ways to recognize signs of trafficking and offers information on intervening in a safe, well-thought-out and professional way.

Seeing the clues, spotting red flags

Although no single set of symptoms or signs indicates definitively that a person has been trafficked, human trafficking situations are associated with common features that, if linked together, may suggest that a person has been trafficked. Figure 3 lists key factors that might suggest a person has been trafficked.

Figure 3: Linking clues that a patient may have been trafficked

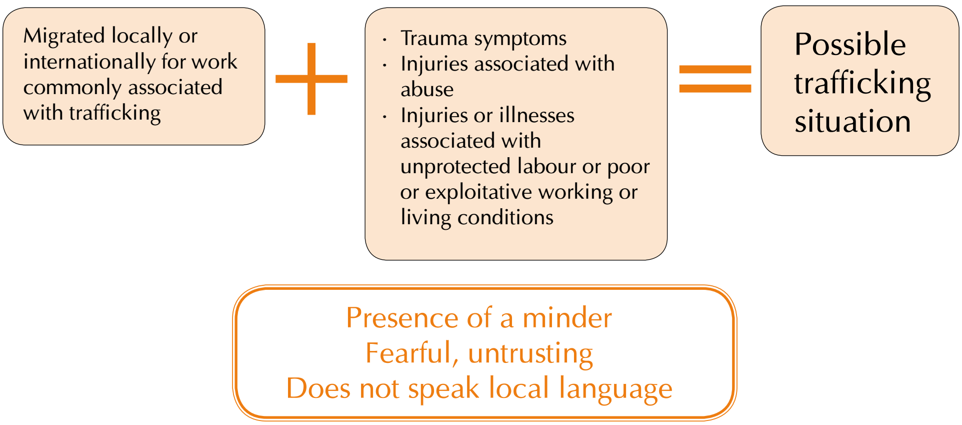

These indicators are particularly relevant when a patient is an international migrant or appears to have migrated within a country for work. Suspicions about trafficking should increase if an individual discloses or is identified as having worked or currently working in a job sector commonly associated with trafficking; if he or she shows signs or symptoms of trauma reactions, injuries, illnesses or infections that suggest that abuse has occurred or that the individual has been working in exploitative conditions; or if the patients shows physical manifestations of poor nutrition, hygiene and lack of self-care (*see chapters 1 and 2*).

Trafficked persons may also appear fearful, mistrustful and anxious about their surroundings. They may have difficulties in articulating their medical complaints; sometimes they may not speak the local language. They may have legal problems that add to their fear and mistrust, including problems

related to immigration status or possibly criminal status. In the more obvious trafficking cases, a 'minder' will accompany the trafficked person to serve as a translator, monitor behaviour or provide transportation. Intimidation is not always evident – but often the signs of quiet coercion can be discerned.

Finally, it is not uncommon for a person who has been trafficked to be unaware that he or she is a victim of a crime. Many trafficked persons will not have heard of the crime of 'trafficking' and may consider the exploitation or abuse that occurred to be a matter of bad luck, or the result of poor judgment.

REQUIRED ACTIONS

Picking up on the red flags described above, you should consider the actions outlined below.

If you suspect or find out that your patient has been trafficked

The most important part of the response to a suspected trafficking situation is the work you do **before** you react. Learn about the protection options available for trafficked persons or similar vulnerable groups in your country and local setting. Keep referral information in various languages if possible, including names and phone numbers of contact persons and know whether these referral options are competent to support the needs of trafficked persons – and specifically your patient (*see action sheet 10*).

Bear in mind that you may not be able to rescue your patient due to security risks to you and/or your patient, and that the responsibility is not yours alone. There are other options available to maximize the benefits to your patient, even if you only have one single encounter. Consider the following important points:

- **Do not** try to rescue your patient if you are not yet linked to the protection system available for trafficked persons in your country or area, and do not have proper information on existing referral networks and available services (*see action sheet 10*).
- Ensure the safety of your patient, yourself and your health facility first (*see action sheet 7*).
    - o Find ways to talk to the patient alone. **Do not** inquire about trafficking-related circumstances in front of others, including your patient's companion. To gain privacy with the patient, you could, for example, suggest that a private examination is required.

   - Ask the patient whether he or she feels safe to talk about things that may be bothering him or her at this time.
   - **Do not** disclose your personal address to your patient or attempt to shelter him or her in your own home.
- Do not contact the authorities (e.g., police, immigration) without explaining that this is an option and gaining the patient's permission (see below).
- The following are possible questions to ask that may give a better understanding of your patient's situation. Always ask your questions in relation to your patient's health, and in the simplest way possible. React supportively to whatever is reported. Select those questions that are relevant or adapt any of the following symptoms according to the individual's health conditions:
   - You look *very pale*. Can you tell me about your diet? What have you eaten this week? Over the last month?
   - You are *coughing a lot*. I need to know about your home situation. Can you tell me about your home and your bedroom? Are you sharing your room with others? Do you have a window in your room? Can you open it easily?
   - I think you may be suffering from a disease that is not common here. Where are you originally from? How long have you been here?
   - Were you *injured* while working? Can you tell me about your work and how you were injured? Is this the first time that you were injured or do you have other injuries elsewhere? I need to make sure that you are okay everywhere.
- **Do not** ask anyone accompanying the individual to assist with interpreting or an examination, even if you do not speak the same language as the patient and you have no immediate access to interpreting services (*see action sheet 3*).
- Do not make promises you cannot keep. When trying to give your patient hope, only offer what you are sure can be delivered.
- Your patient may not be able to pay for necessary health services; you need to assess funding support for basic social services in your country or area *(see action sheet 10*).

After the initial assessment

Various scenarios are possible after the initial assessment.

- You may be able to refer your patient to another organization for protection and further assistance.

- You are not able to provide a referral, but you feel that you can ensure your patient's compliance to come to you again for follow-up medication and examination.
- You feel that this particular encounter with your patient will likely be the only one for whatever reason.
- There is clear presence of imminent danger and urgent assistance is required.

When referral is possible and you are aware of services available and who to contact, consider the following (*see action sheet 10*):

- What protection services are available in your country or area for trafficked persons? Is there a specific agency appointed as first point of service? Are you compelled by local laws to report cases of trafficking and/or violence?
- What service(s) does your patient most need at the time of your encounter? Shelter and food? More comprehensive health and psychological care? Legal or immigration assistance? Translation services? The trafficked person should have a voice in making this decision whenever possible (*see action sheets 1 and 16*).
- **Do not** contact any support organization or the police without first explaining to your patient how this communication will affect him or her. Discuss available options, and explain how they work and what the benefits are. **Act only with your patient's consent.**[28]
- Different contexts may apply to your patient in terms of his or her rights, depending on his or her immigration and legal status, presence of proper documents, and the type of work engaged in (whether legal or illegal). All of these circumstances may have an impact on the patient's safety. Explain the situation to your patient and help him or her make the best decision.

When you cannot refer your patient but feel confident about your patient's compliance for follow-up assessment and treatment, consider the following:

- Maintain your professional role as health provider. Provide comprehensive management for your patient, including arranging follow-up care and visits.
- You may face different circumstances in your next encounter with your patient. Your patient may develop trust and request different assistance.

[28] Please see action sheet 16 for special considerations related to competence, capacity, and guardianship.

- Be prepared at this time with appropriate referral information that can be offered in a safe way.

When referral is not possible because the patient does not desire it, the situation is too unsafe to make a referral, or the patient is subject to deportation or immediate return home, maximize the encounter with the patient because it may be the only chance you have to help improve your patient's condition:

- Offer the patient as much information as possible related to his or her medical condition, treatment and necessary follow-up. Provide information about the crime of trafficking, available support services including hotline phone numbers, details on where your patient can go, and information on whom to call in the future if the patient should wish to take advantage of services at a later time.
- Communicate this information carefully. Be attentive to the form of documentation you provide your patient because there will usually be safety risks involved. Documents may be traced back to you or your health facility; therefore you may want to put this important information on a piece of paper small enough for your patient to hide under clothing (e.g., to tuck into undergarments).
- Provide a complete regimen of prescribed medication in that single encounter, if applicable and possible. Assume that the patient will not be able to come back for follow-up treatment and assessment, or for additional diagnostic examinations. Use single dose therapy whenever possible and provide your patient with a medical summary and referral documents as appropriate.

When urgent assistance is required or imminent danger is present, the following are important points to consider:

- Ensure your own safety first.
- If emergency medical referral is needed, you might have to persuade your patient and any companion or minder about this. Focus on the health status of your patient and **do not** elaborate on the causes of your patient's health deterioration, especially if they may be related to abuse.
- You may face situations where emergency medical assistance will need to be provided in your facility (*see action sheet 11*).
- If your patient is alone and contact with police is desired by the patient or seems necessary to the individual's immediate safety, discuss this slowly and clearly with your patient, making certain that this is the preferred course of action. Refer

to a specific trusted police focal point whenever possible (*see action sheet 10*).

REFERENCES AND RESOURCES

International Organization for Migration

2007 *The IOM Handbook on Direct Assistance for Victims of Trafficking*, IOM, Geneva, 2007.

2006 2004 *The Mental Health Aspects of Trafficking in Human Beings: Training manual*, IOM, Budapest, Hungary, 2004.

2004 *The Mental Health Aspects of Trafficking in Human Beings: A set of minimum standards*, IOM, Budapest, Hungary, 2004.

2001 *Medical Manual, 2001 Edition*, IOM, Geneva, 2001.

Pan American Health Organization, Women Health and Development Program and Organization for American States, Inter-American Commission on Women

2001 "Trafficking for Sexual Exploitation", Fact Sheet of the Program on Women, Health and Development, Washington, DC, July 2001.

Zimmerman, C. and C. Watts,

2003 *WHO Ethical and Safety Recommendations for Interviewing Trafficked Women*, World Health Organization, Geneva 2003.

Zimmerman, C. et al.

2008 "The health of trafficked women: a survey of women entering posttrafficking services in Europe", *American Journal of Public Health*, vol. 98, no. 1, January 2008, pp. 55–59.

Action Sheet 7:
Protection and security

AC
TION
SHEET
SEVEN

Action Sheet 7: Protection and security

RATIONALE

All trafficked persons have the right to physical safety and protection. According to international standards, "States have a responsibility under international law to act with due diligence to prevent trafficking, to investigate and prosecute traffickers and to assist and protect trafficked persons."[29]

Protection is one of the three 'Ps' of counter-trafficking activities – prevention, protection and prosecution – and is an essential element of an assistance package to a trafficked person. For a healthcare provider, 'protection' means meeting individuals' health needs and helping to keep them safe from harm.[30]

Human trafficking is a criminal activity that frequently involves organized crime networks. The security and physical safety of trafficked persons—and of health care providers—must always be among the highest priorities. For traffickers, the loss of a trafficked person is the loss of income and a symbol of their loss of control. In some cases, individuals who have escaped traffickers may be pursued by them or their co-conspirators, especially if the trafficked person is participating in a criminal investigation against the trafficker.

Although health care providers are not ultimately responsible for the security of a trafficked person, they are obligated to contribute in every way

[29] United Nations Office of the High Commissioner for Human Rights, *Recommended Principles and Guidelines on Human Rights and Human Trafficking*, Report of the United Nations High Commissioner for Human Rights to the Economic and Social Council (E/2002/68/Add.1), United Nations Economic and Social Council, New York, 20 May 2002. Article 2.

[30] In a broader sense, protection also means creating a social, political and legal environment that fosters the protection of victims of trafficking. For states that receive persons trafficked internationally, this may mean offering special residence permits or visas to allow victims from other countries to remain in a country legally, for example. Protection may also involve assisting trafficking survivors to return to their country or region of origin in safe ways that respect their human rights. See: *IOM Counter-Trafficking Training Modules*.

possible to the health and safety of individuals in their care, and to refer them into services where they may be safe. And, while cases of trafficking do not always pose a threat to persons providing health care and support, health professionals should still be attentive to potential security risks to themselves and other staff members. This action sheet describes some of the measures that a health facility may take to ensure the safety of a trafficked person and of the staff.

REQUIRED ACTIONS

A key principle of providing assistance to trafficked persons is that actions and services should be conducted within what is known as a 'sphere of protection'.[31] This means prioritising safety and security during the assistance and referral process, including making an effort to protect victims from harm, threats or intimidation by traffickers or their associates.[32]

Maintaining **strict** confidentiality about trafficking cases is a basic security measure that health professionals can take. Staff of medical facilities that assist trafficked persons should have well-defined and well-communicated rules regarding confidentiality about trafficking cases (*see action sheet 9*). Information regarding a trafficked person's identity, whereabouts and circumstances must be protected at all times (*see action sheets 7, 9 and 10*). This is essential not only to protect you and the trafficked person from reprisals by traffickers, but also to protect trafficked persons from potential stigma within their families or communities. Protection also includes shielding individuals from the attention or abuses, of the media. Health professionals are strongly discouraged from facilitating journalist, filmmaking and other media interviews with trafficked persons. Health facilities must be viewed as safe and anonymous locations of care and support by individuals who are vulnerable. Clear policies on media should be established and communicated.

Careful listening is an essential security tool in cases of trafficking. Health providers must take the time to learn about any security risks associated with their patient. Trafficked persons are often in the best position to know of and interpret any dangers they may face. However, providers must never coerce or pressure individuals to divulge details they are not ready to discuss. Information-gathering must happen in a non-coercive way. In cases where other service providers or police are involved, health

---

31 International Organization for Migration, *IOM Counter-Trafficking Training Modules: Return and reintegration*, IOM, Geneva, 2005.

32 United Nations Office of the High Commissioner for Human Rights (2002). Article 2.

providers should enquire about potential safety risks to the trafficked person and to themselves. They should seek advice from competent individuals or agencies on how address possible risks.

Well-functioning referral networks and procedures are crucial (*see action sheet 10*). Health providers should be prepared, in advance, to know who to call in situations that feel unsafe. In some countries a special counter-trafficking police unit exists; in some places, a counter-trafficking hotline can link a health provider to specialists who can help in a crisis situation (*see action sheet 10*).

Having in place a specific security protocol or code of conduct for dealing with patients who have been trafficked can help maintain a safe environment for staff and patients. Health facilities may wish to institute a special security plan for trafficking cases and to alert staff and patients about safety procedures and key contacts, if potential problems arise.

It is important to be aware that:

- Security is a priority in trafficking cases, but total security may not be possible.
- Personal security can be enhanced by assessing and managing risks, being aware of the immediate surroundings and situation and listening carefully to the patient's assessments and concerns.
- Although there is the potential to cause staff anxiety by overstating potential risks, everyone who interacts with trafficked persons should be informed of possible risks.

Health structures

Conduct a risk assessment to identify potential security problems for trafficked persons and staff. Depending on the local context, a medical facility may want to take specific security precautions, such as alarms or, in extreme circumstances, security personnel, depending on the potential dangers associated with assisting trafficked persons.

Health providers

Take responsibility for your own safety and adhere to any established security procedures:

- Do not give out personal contact details or take trafficked persons into your home.
- When interpreters are involved, take time to warn them against disclosure of case details (*see action sheet 2*).
- Do not discuss health conditions or give statements about a trafficked person to uninvolved third parties or to the media.
- Do not discuss a trafficked person's health-related matters in public areas or with family or friends.

Medical staff providing health outreach services in settings where trafficked persons may be exploited must take additional safety precautions. Security measures may vary slightly among outreach teams, but general principles include:

- Never work alone. Outreach health providers should always work in teams of two or more.
- Establish check-in and check-out procedures for outreach work so that office staff know when you are on your shift **and** when you have returned.
- Show extra caution in isolated areas.
- Be vigilant for yourself and encourage outreach partners to do the same.
- When conducting outreach work, always keep interactions safe and do not engage in personal conversations that may reveal personal details, residence or information about loved ones. Don't give out personal phone numbers or addresses.
- Prepare in advance an alert system linked to your office, other assistance organizations or to the local police station to trigger an immediate emergency response in the event of an incident or serious security threat.

Trafficked persons

- Make individuals who have been trafficked aware of efforts to protect them and encourage them to be alert to their surroundings and to communicate concerns. Explain that they should also play a role in preserving their safety and the security of those assisting them.
- Whenever possible, try to arrange for trafficked persons to be accompanied when they are making their first and, in some cases, subsequent visits to other support service locations.

REFERENCES AND RESOURCES

International Organization for Migration

2007 *The IOM Handbook on Direct Assistance for Victims of Trafficking*, IOM, Geneva, 2007.

2005 *IOM Counter-Trafficking Training Modules: Return and reintegration*, IOM, Geneva, 2005.

United Nations Office of the High Commissioner for Human Rights

2002 *Recommended Principles and Guidelines on Human Rights and Human Trafficking*, Report of the United Nations High Commissioner for Human Rights to the Economic and Social Council (E/2002/68/Add.1), United Nations Economic and Social Council, New York, 20 May 2002.

Action Sheet 8:
Self-care

AC
TION
SHEET
EIGHT

Action Sheet 8: Self-care

RATIONALE

Providing support to people who have been trafficked is usually a highly rewarding experience. However, exposure to graphic details of abuse and to the ways in which abuse affects the lives of those who have been trafficked can be stressful for health providers.

It is not unusual for people working with trafficked persons to experience emotions of anger, pain, frustration, sadness, shock, horror and distress. The work could also affect energy levels and cause disturbed sleep, somatic complaints and hyper-arousal. Some health providers may experience intrusive images, thoughts and nightmares about their patients' distressing experiences. These symptoms have been described as 'secondary traumatic stress' and are similar to post traumatic stress disorder, except that the exposure is to knowledge about a traumatizing event experienced by someone else. Health care providers re-experiencing a patient's traumatic event often wish to avoid both the patient and reminders of his or her trauma. They may also have increased concerns over their own safety and the safety of the people close to them.

Hearing about extreme trauma can change a health provider's view of the world, of other people and of him- or herself. The provider may see the world as a more dangerous place, lose trust in other people and experience feelings of personal helplessness and hopelessness. Health care providers may react to such changes by distancing themselves from their patients and experiencing what is known as 'compassion fatigue', a reduced capacity or interest in being empathic or in bearing the suffering of patients. Compassion fatigue among health care providers also encompasses symptoms of job burnout. This is characterized by emotional exhaustion and reduced personal accomplishment in response to prolonged exposure to demanding interpersonal situations without adequate support.

Research into the effects of supporting traumatized patients on health care providers suggests that a provider's own beliefs about his or her role might be associated with secondary trauma and compassion fatigue.[33] Common beliefs might include such thoughts as 'I can help everyone and can help everyone immediately'; 'the more time I give, the more I can help my patients'; and 'I can know everything. I can love everyone'.

Systemic issues such as size of caseload, level of organizational support, training, case supervision and peer support are also associated with the effects of trauma work on health care providers.

REQUIRED ACTIONS

If you are worried about your safety because of the nature of your work please see action sheet 7, which provides recommendations for physical protection. Be aware, however, that increased concerns over danger may also be a symptom of secondary traumatic stress. The following recommendations provide information on how to put in place systems to minimize potential negative effects of providing care for trafficked persons.

Recommendations for organizations and managers

A basic requirement for the psychological well-being of personnel is a sense that their employer is taking all the necessary steps to ensure their security. First, it is important to ensure that personnel have and *feel* that they have the necessary level of training to carry out the duties the job requires.

- Make certain health care staff have job descriptions that clearly define the goals and limitations of their work and options for professional support and stress management. Make sure these are reviewed and appraised regularly. Clearly defined roles and available resources can prevent staff from feeling overwhelmed or helpless.
- Give staff copies of these guidelines and make them aware that they could potentially experience secondary trauma, job burnout and chronic fatigue.
- Schedule regular clinical supervision. Clinical supervision is essential to ensuring quality of care and minimizing the risk of compassion fatigue. The availability of such supervision will vary in different settings. Clinical supervision, which should be scheduled at regular times when possible, can be carried out in a group, but staff should also have individual

[33] See references at the end of this action sheet for more information.

supervision. Separating management issues from clinical supervision in important when the supervision is done by a manager.

- Establish clear guidelines regarding the size and complexity of the clinician's caseload; this can help minimize the risk of job burnout. Caseloads should be reviewed regularly with supervisors to ensure a balanced caseload. When possible, a health care provider's caseload should be varied. In a centre dealing only with trafficked persons, for example, direct patient care can be combined with work that does not involve direct contact with patients; it may also include having patients at different stages of rehabilitation.
- Discourage attitudes of controlled emotions and of bravado. Where possible, services should foster a culture of peer support and an environment open to discussions of health care providers' emotions in relation to their work.
- Establish a procedure for health care providers to request that personally challenging cases be transferred to other colleagues.
- Encourage health care providers to keep a healthy work-life balance. Health care providers should be discouraged from neglecting their own leisure and social life in order to help their patients.

Recommendations for health care providers

- Work with patients on agreed goals – goals that are derived directly from a treatment plan and based on a shared understanding of the difficulties facing the patient. This will help both you and your patient to have realistic expectations and avoid feelings of helplessness and hopelessness; it will also help define the boundaries of your work together.
- Discuss your cases regularly with your clinical supervisor or colleagues to ensure good practice and self care.
- If you are struggling with your caseload, bring this to the attention of your supervisor and colleagues. Not doing so could be harmful to your patients and yourself.
- Demonstrate caring attitudes towards your colleagues; this will help create and maintain a supportive working environment in your service.
- Use your social support network and leisure activities as a way of looking after yourself. This could include such self-care behaviours as taking holiday leave, relaxing after work, and getting regular exercise.

- Work with traumatized individuals could potentially trigger unresolved emotional difficulties in the service provider. As a first step, discuss this with someone you trust who will maintain confidentiality. Patients' confidentiality is important in all clinical settings but it has additional importance when working with trafficked persons because of the forensic aspects of the work.
- Engage in psychological therapy if emotional difficulties remain unresolved.

Identifying signs of fatigue or burn out

Below are some early signs that can help you recognize whether you are affected by your clinical work with trafficked persons:

- Chronic fatigue – exhaustion, tiredness and a sense of being physically run-down
- Anger at those making demands
- Self-criticism for putting up with the demands
- Cynicism, negativity and irritability
- A sense of being besieged
- Exploding easily at seemingly inconsequential things
- Frequent headaches and gastrointestinal problems
- Weight loss or gain
- Sleeplessness and depression
- Shortness of breath
- Feelings of suspicion
- Feelings of helplessness
- Increased degree of risk-taking

If you have some of these symptoms, you might wish to fill in the compassion fatigue scale (following). You can use it to assess compassion fatigue, secondary trauma and job burnout.

How to address compassion fatigue

If you think that you are affected by your work and suffer from compassion fatigue, secondary trauma or job burnout, discuss it with your supervisor. Do not ignore the signs of these difficulties, because they will not go away unless you address them. The same applies if you are a manager or clinical supervisor who suspects that a staff member is experiencing symptoms; these should not be ignored. A meeting between the health care provider and supervisor should be arranged as soon as possible. The aim of the meeting is to assess the health care provider's needs and how those could

be met, while considering how his or her clinical caseload could be covered. The discussion in the meeting should cover following issues:

- If an occupational nurse or doctor is available, discuss referring the health care provider.
- If the organization does not have occupational support persons, review the health provider's current responsibilities and agree on if – and to what extent – he or she can continue to carry out responsibilities towards patients.
- Discuss who could provide appropriate support and counselling to the health care provider.
- Agree on who to notify of the difficulties that the health care provider is encountering.
- Arrange a review meeting to discuss the health care provider's progress and his or her care plan and job description.

Compassion fatigue scale[34]

Consider the following thoughts related to your work/life situation. Write the number that best reflects your experiences using a scale of 1 through 10:

**Never/rarely**									**Very often**
1	2	3	4	5	6	7	8	9	10

____ a. I have felt trapped by my work.
____ b. I have thoughts that I am not succeeding in achieving my life goals.
____ c. I have had flashbacks connected to my clients.
____ d. I feel that I am a 'failure' in my work.
____ e. I experience troubling dreams similar to those of a client of mine.
____ f. I have felt a sense of hopelessness associated with working with clients/patients.
____ g. I have frequently felt weak, tired or rundown as a result of my work.
____ h. I have experienced intrusive thoughts after working with especially difficult client/patients.
____ i. I have felt depressed as a result of my work.
____ j. I have suddenly and involuntarily recalled a frightening experience while working with a client/patient.
____ k. I feel I am unsuccessful at separating work from my personal life.
____ l. I am losing sleep over a client's traumatic experiences.
____ m. I have a sense of worthlessness, disillusionment or resentment associated with my work.

The scale has three subscales: secondary trauma (items c, e, h, j and l); job burnout (a, b, d, f, g, i, k and m); and chronic fatigue (all items). There are currently no cut-off scores for the scale but high scores are suggestive of the indicated conditions. The scale can provide a monitoring tool.

[34] Adams, R. E. et al., "Compassion fatigue and psychological distress among social workers: a validation study", *American Journal of Orthopsychiatry*, vol. 76, no. 1, January 2006, pp. 103–108.

REFERENCES AND RESOURCES

Adams, R. E. et al.

2006 "Compassion fatigue and psychological distress among social workers: a validation study", *American Journal of Orthopsychiatry*, vol. 76, no. 1, January 2006, pp. 103–108.

Figley, C.R. (Ed.)

2002 *Treating Compassion Fatigue*, part of Psychological Stress Series, Brunner-Routledge Press, New York, NY, USA, 2002.

Action Sheet 9:
Patient data and files

Action Sheet 9: Patient data and files

RATIONALE

Although most health providers have in place certain security precautions for patient medical files and rules around patient confidentiality, providers caring for persons who have been trafficked will need to institute extra safeguards for written, electronic and verbally communicated information on trafficked persons.

> "Health data include all records pertaining to the physical, mental and social health of the trafficked person. A health information system is the way in which health data are collected, organized, stored and communicated."[35]

As with all patient data, the way information is collected, stored and transferred between providers is important to accurate diagnosis and treatment.[36] Because trafficked persons are particularly likely to face security risks, be referred to other providers and/or transferred from the original care location (e.g., internationally) or participate in one or more legal proceedings, it is especially important to follow good data management procedures to ensure the safety of each individual and the quality of follow-up and future care. Trafficking cases may involve organised criminal groups. Health data may be used in court or may be used to support or undermine an asylum claim. Notably, patient files may also identify health care staff involved in providing care.

Central to managing the health data of trafficked persons are: **privacy, confidentiality** and **security** (*see chapter 3*). These concepts are fundamental principles in handling trafficking-related data in general and certain sensitive

[35] International Organization for Migration (2007), p. 256.
[36] Ibid., p. 255.

health information (e.g., HIV) in particular.[37] **Privacy** refers to the patients' right to control how they provide information, the use of this information and their access to it. **Confidentiality** indicates the right of patients to determine who has or does not have access to their patient information and for trafficked persons, suggests the importance of anonymity. **Security** suggests the need to safeguard patient files against security breaches during data collection, storage, transfer and use.

In managing data on trafficked persons it is important to recognize and balance the patients' rights to protect and access their personal data **and** the need for health care providers to collect, use and disclose personal data in the course of providing care. The framework for protecting the confidentiality and security of HIV information provides a good example of mechanisms for managing the health data of trafficked persons.[38]

This action sheet focuses on the collection, storage, transfer and security of data management in cases involving or potentially involving trafficking and outlines actions to undertake in managing the health information of trafficked persons. It should be read alongside action sheets 7 and 10.

REQUIRED ACTIONS[39]

In addition to adhering to the overarching principles for managing data, health care providers must also take specific actions throughout, and even after, a patient is in their care. Protecting patient information will often involve a number of individuals in a health care setting (e.g., reception staff, nurses, doctors, consultants, managers, data-entry clerks, etc.). All who have a potential role in communicating with trafficked persons, managing information or transferring file data should be made aware of the rules and procedures for patient information.

There are several phases in the management of patient information. Many of the precautions are common for all patient information. In cases of trafficking or potential trafficking, health providers must take care to carry out these steps with the utmost care and attention to the immediate and future safety and well-being of the patient—and health care staff.

---

[37] International Organization for Migration (2007); UNAIDS, *Guidelines on Protecting the Confidentiality and Security of HIV Information: Proceedings from a workshop 15-17 May 2006 Geneva, Switzerland, interim guidelines*, UNAIDS, Geneva, 15 May 2007.

[38] Health care providers should also refer to how various forms of sensitive health data are managed within their respective organization.

[39] The following sections are adapted from the *IOM Handbook on Direct Assistance for Victims of Trafficking* (2007). The *Guidelines on Protecting the Confidentiality and Security of HIV Information: Proceedings from a Workshop 15-17 May 2006 Geneva, Switzerland, Interim Guidelines* (UNAIDS, Geneva, 15 May 2007) in particular were also consulted as an important source.

Data collection

Personal information, including medical history-taking, must be collected in a private setting. Both the patient and the provider must feel safe to speak freely. It is a good idea to ask individuals if they feel comfortable and ready to discuss their health and care needs. Consider, for example, whether the individual wants the door open or closed, discourage other staff from interrupting, and turn off your mobile phone.

- **Obtain informed consent**: The fundamental element of 'informed consent' is informing, by providing clear and accurate information. At the first health consultation, providers should offer information about the scope and purpose of the consultation, services that are and are not available and the measures in place to ensure patient privacy and confidentiality. Once information on data use is provided, an individual may be asked for consent to proceed. Only information covered in the scope of the consent may be requested. If information is to be used for research purposes, this must be disclosed and separate consent may be required. Informed consent is when patients are able to consider the relevant facts (purposes, procedures, uses, risks and benefits) associated with data collection and **then** agree. If consent cannot be obtained because, for example, the trafficked person is a minor, is in a state of trauma or has a physical or psychological disability that would prevent him or her from giving truly informed consent, the health care provider should, at a minimum, ensure that the patient understands sufficiently and appreciates the specified purpose for which personal data are collected and processed. The condition and legal capacity (e.g., if the patient is a minor) of the patient to give consent should be taken into account. The patient must also be allowed to give, withhold and withdraw consent at any time without negative consequences (*see action sheet 16*).
- **Collect pertinent data.** Collect only information that is required to assess and respond to care needs, not simply for curiosity's sake. Refrain from asking trafficked persons about the non-health related consequences of the trafficking process. This may put you in a risky situation and may cause the trafficked person to relive stressful experiences, which may have a negative impact on recovery. Many victims of sexual abuse feel stigmatised by their experience and by certain health problems (e.g., sexually transmitted infections or psychological disorders).

Data file storage

- **Ensure case files are coded.**[40]All health data of trafficked persons must be considered highly sensitive. In trafficking cases, data should be classified as confidential and coded, rather than using the individual's name. Coding is a particularly useful way to protect patients, especially when data is shared. Patient health files should immediately be assigned a unique identification number. Master files connecting individual names matching them to identification code numbers must be kept in a secured location, with access restricted to the key health care provider or designated support person. Refer to individuals by a designated code number on all other documents.[41] Do not use identifiable patient case details and characteristics for published research or reports.
- **Secure paper files.** It is likely that during the course of providing health care, **paper copies of confidential health data** will be produced. All paper case files:
  - must be kept secure and under lock and key safe from unauthorized entry at the health care practice.
  - must never be left unattended on desks, in common areas, etc.
  - must be disposed of by shredding or through similar disposal method when no longer needed.
- **Secure electronic files.** In addition, **electronic heath data files** should be:
- stored in a secure health information system.
- protected by individual passwords with access limited to key staff.
- never stored on personal computers or in such data storage devices as USB flash drives, compact discs, etc. Personal information should never be disclosed via email.

Caution! Even when coding is used, individual patients often can be readily identified by their basic data (demographic background, ethnicity, nationality, date of birth, family data, description of elements of the trafficking process, etc.). Only key health care providers and support staff should have access to a patient's primary case file.[42]

---

[40] Adapted from *The IOM Handbook on Direct Assistance for Victims of Trafficking*, IOM (2007).
[41] Ibid.
[42] Ibid.

- **Conduct a data risk analysis.** Providers caring for trafficked persons may wish to undertake a data risk analysis to consider the implications of what is written in a file, the potential uses and misuses of patient data and to develop strategies to avoid mishandling of data and to deal with information requests. File information may, for example, be required by law enforcement in relation to a court case against an alleged trafficker or needed for an asylum claim. In more sinister situations, traffickers might try to obtain the trafficked person's file information to locate individuals or learn about their health conditions. It is equally important to remember that personal data (name, work location, phone number, etc.) of the health care provider could also be misused. It is therefore important to follow well-designed data security procedures (please see below).

Information communication to patient

All trafficked persons have the right to be fully informed of their medical conditions and health needs and should receive copies of their complete medical and health records.

- **Full and clear communication of information to patient.** Trafficked persons should be fully informed of their medical conditions, diagnoses, test results, health needs and proposed follow-up procedures, which are also recorded in their file. They should be offered copies of their complete medical records. Patients should be given an opportunity to verify and rectify their personal data.

Information communication to others

Sharing case information among health professionals is often necessary for good case management. Health files, electronic data and verbal case information must be transferred to other health practitioners in an efficient and careful manner carefully. All health care providers and support staff, including interpreters, should adhere to the following:

- **Health files and information should not be disclosed to third persons without the prior consent of the patient.**
- **Only 'need-to-know' information should be transferred to others.** Only information that is pertinent to an individual's safety and care should be disclosed to other internal or external parties, on a 'need-to-know' basis and with the consent of

the trafficked person. Issues regarding legal obligations and consent are discussed in action sheet 16.

- **Discuss case information in private and in confidence.** Never communicate about a trafficked person's health records in a public or open environment. When health care providers discuss details of a patient's case during peer supervision or in staff meetings, all information shared must be kept strictly confidential.
- **Do not discuss case information with family members unless person is legally designated guardian.** A trafficked person's case history should never be discussed with family members, friends, other trafficked persons or third parties without the explicit consent of the individual.[43]
- **Conduct ethical and lawful data collection.** Health care providers must be aware of relevant domestic and international laws or guidelines on data protection and mandatory disclosure laws. In certain circumstances, to protect public health or public security you may need to comply with national regulations regarding reporting related to trafficking incidents. In these situations, be certain to learn about your legal rights and obligations as a practitioner and the rights of your patient before divulging private files or information.

What do I do if the patient's health data is requested by a law enforcement agency in relation to a criminal investigation against an alleged trafficker?
Your cooperation might be requested by law enforcement officials for a criminal proceeding against an alleged trafficker, or in relation to an asylum or temporary residency claim by a trafficked person. You may be required to submit a written statement or appear in court. To be certain of your compliance requirements, seek legal counsel.

Procedures and training in data collection and security

Providers caring for trafficked persons must design and document special procedures for collecting, storing and communicating patient information in high-risk cases. Staff should all be made aware of the procedures and particularly the limits of communication of patient information. All staff should be made aware of file security procedures and of procedures to follow if they become aware of safety risks or security breaches.

[43] In certain cases or for therapeutic reasons, it may be useful to offer examples from a case similar to the trafficked person's experience. If this is done, it is necessary to change names and sufficiently alter personal details so that the case being discussed cannot be identified. From The *IOM Handbook on Direct Assistance for Victims of Trafficking*, IOM (2007).

REFERENCES AND RESOURCES

Council of Europe

2005 Council of Europe Convention on Action Against Trafficking in Human Beings, treaty open for signature by the member states, the non-member states which have participated in its elaboration, and by the European Community, and for accession by other non-member states, CETS no. 197, Warsaw, Poland, 16 May 2005.

European Parliament and the Council of the European Union

1995 "Directive 95/46/EC of the European Parliament and of the Council of 24 October 1995, on the protection of individuals with regard to the processing of personal data and on the free movement of such data", *Official Journal of the European Communities*, no. L 231/81, 23 November 1995.

EuroSOCAP Project

2005 European Standards on Confidentiality and Privacy in Health Care, EuroSOCAP and Queen's University, Belfast, November 2005.

International Organization for Migration

2008 Data Protection Principles: Information Bulletin IB/00047

2007 *The IOM Handbook on Direct Assistance for Victims of Trafficking*, IOM, Geneva, 2007, section 5.17.1.

Joint United Nations Programme on HIV/AIDS (UNAIDS)

2007 *Guidelines on Protecting the Confidentiality and Security of HIV Information: Proceedings from a workshop 15–17 May 2006 Geneva, Switzerland, interim guidelines*, UNAIDS, Geneva, 15 May 2007.

United Nations Children's Fund

2006 *Guidelines on the Protection of Child Victims of Trafficking*, UNICEF Technical Notes, provisional version 2.1, UNICEF, New York, September 2006.

United Nations Office of the High Commissioner for Human Rights

2002 *Recommended Principles and Guidelines on Human Rights and Human Trafficking*, Report of the United Nations High Commissioner for Human Rights to the Economic and Social Council (E/2002/68/Add.1), United Nations Economic and Social Council, New York, 20 May 2002.

1990 Guidelines for the Regulation of Computerized Personal Data Files, adopted by General Assembly Resolution 45/95 of 14 December 1990.

Action Sheet 10: Safe referrals

Action Sheet 10: Safe referrals

RATIONALE

The needs of trafficked individuals often go well beyond medical needs to include emergency food and shelter, as well as legal support to deal with immigration, criminal or guardianship matters. A strong referral network among medical professionals and other service providers is necessary to address these needs; referral is not simply a means of transferring an individual from one service to another, but is a fundamental part of providing care. To make a safe referral is to ensure that provision of assistance is handed over to other support services in a way that does not jeopardize the health or safety of the individual.[44] This action sheet covers safe referrals and includes a form for 'mapping' possible referral partners in the community.

It is essential for health care providers to know **where** to refer a trafficked person for help **before** encountering the patient. At the same time, referral of trafficked persons is more complex than for other referrals, because of the range of services needed and the related security risks (*see action sheets 6 and 7*). It is not the responsibility of the health care provider to manage all the needs of the patient; yet the referral process does have the potential to either benefit or worsen a patient's well-being. When referrals are well done, all care providers and the trafficked person feel informed and secure. Poorly thought out referrals, on the other hand, can put patients at risk and break the chain of care. This action sheet is designed to help health care providers develop and implement well-planned and well-executed referrals. It should be read together with action sheet 6.

[44] National referral mechanisms are a key component of counter-trafficking activities. A national referral mechanism can be defined as "a co-operative framework through which state actors fulfil their obligations to protect and promote the human rights of trafficked persons, co-ordinating their efforts in a strategic partnership with civil society." Organisation for Security and Co-operation in Europe (OSCE) / Office for Democratic Institutions and Human Rights (ODIHR), *National Referral Mechanisms*, 2004.

REQUIRED ACTIONS

In some countries a functioning referral system for trafficked persons may exist. Such systems link governmental and non-governmental organizations to coordinate the comprehensive assistance and protection needed by a trafficked person. Where such systems do not exist, it is essential to take the necessary steps to identify and assess available services.

Identify and assess services

Learn about the availability and quality of potential providers of the various services the might be needed by a trafficked person, so that you are prepared to refer your patient safely. These might include organizations providing social services, housing and legal aid; and government contacts in agencies such as law enforcement, consular services, and migration. See the mapping form at the end of this action sheet for more details. Whenever possible, try to assess (and record) the following qualities of each provider or service to help inform future referrals:

- Professionalism and quality of care
- Non-discriminatory, sensitive treatment
- Confidentiality regulations, including patient file and specimen code numbers, locked file facilities, anonymous data transfer capacity (see action sheet 9)
- Procedures for obtaining informed consent
- Security, such as locked facilities, safe location, security staff (see action sheet 7)
- Private rooms for interviewing, examination and treatment
- Language capacity.
- Experience supporting victims of violence or with trauma-informed care (see action sheet 1)
- Cultural and religious aspects, potential implications of cultural or religious characteristics (see action sheet 2)
- Regulations regarding payment
- Location and accessibility (see action sheet 14)

Referral options should be reputable and well-established. It is beneficial to patients and to the referring organization to know whether the staff and key individuals at the referral organization provide non-discriminatory, supportive care to such marginalized groups as migrants, sex workers and minority populations. It can also be helpful to know which service providers have multi-lingual staff.

Develop inter-organizational referral arrangements

Whenever possible, determine agreed referral and information-sharing procedures **before** a referral takes place. Key components of a good referral process include:

- Services that are provided by organization: Details of services that can and cannot be offered by service providers.
- How information and data will be transferred: Details about client information that will (and will not) be transferred to referral organization; how files and other information will be transferred; and how informed consent will be obtained (see action sheet 9).
- How information about services will be provided and patient consent requested: Referral options, arrangements and consent procedures must be clear.
- How the first contact will be arranged: Details about the first point of contact at each referring agency, including main contact person(s), times available, response times for getting called back, if required, and case data required at first contact.
- How the trafficked person will be released and received: Arrangements about transfer to another agency or organization should include details about who has authority to arrange and confirm the referral and release any necessary file information; who has the authority to accept referrals and make reception and support arrangements; and how to ensure that reception staff at receiving organization are aware of and prepared to receive the referred person.
- Minimizing unnecessary movement: Consider whether it is possible to provide treatment and care on site, possibly arranging for the referral institution to come to the patient.
- Escorting or accompanying a referral: Determine when inter-agency escorts should be used and how this might be arranged (see action sheet 14).
- Follow-up and continuity of care: Referring partners should agree on what further care might be required by each organization and arrangements should be agreed for post-appointment information-sharing, including, for example, passing on information about prescriptions and treatment regimen, potential health risks (particularly mental health risks) and security risks.

Inform patient and obtain consent

Inform patient about prescribed treatment and recommended medical or other assistance options, and obtain consent before arranging any referral. It is important that individuals are counselled on all care being offered, including medical procedures, reasons for any prescribed test or treatment; they must also understand referral options and services available. Individuals reserve the right to refuse treatment as well as disclosure of their diagnosis to others. If necessary, use a reliable translation service to ensure the patient's understanding (*see action sheet 3*).

Communicate only information that is required for care and security

Exchange only information that is required for effective care. Personal privacy – in case files and in conversations – is of the utmost importance in sensitive cases. Limit information transferred to other support among support organizations to details that are needed to ensure adequate care for the patient. Do not communicate additional information. Caution staff not to gossip or freely discuss patients' stories or case notes with others at work or at home (*see action sheets 7 and 9*).

Special note: With the patient's permission, it may be possible to transfer basic case file information to help the individual avoid repeating the same information multiple times. Information should be limited to what is agreed upon by the patient and should be done in the most secure way possible.

Make safety and security arrangements for referral

It is important to assess any potential security risks to the patient before arranging a referral. Depending on the level of risk, individuals may have to be moved between service venues covertly or with security personnel. Take into consideration risks to staff of being seen with or associated with a trafficked person. Where appropriate and with the consent of the patient, it can be helpful to discuss options for such secure transfer with police or other trained security personnel (*see action sheet 7*).

Escorting to referrals

Particularly when a trafficked person is not in his or her home country, does not speak the local language or experiences security risks, arranging an escort to a referral site is important. A staff member or support worker may accompany an individual during the first, and sometimes subsequent, visit to a referral partner agency. Particularly when referred to a government agency, law enforcement body or immigration or consular services, an escort can make a significant difference in how a patient is treated.

Feedback between organizations

Feedback between agencies about a trafficked person's case can facilitate follow-up and coordination of care. Ask patients for permission to share case notes; fully inform them about information that is discussed between care providers.

When referral is not possible

Sometimes it is not possible to refer your patient because the situation is too unsafe to do so or because the patient refuses to be referred or is subject to deportation or immediate return home. Maximize the encounter you have with the patient to have as positive an impact on their health as possible (*see action sheet 6*). If possible, share information (e.g., hotline phone numbers) in case the patient decides to make contact at a later time. Such information should be communicated in private, and in a way that will not put the trafficked person at risk (*see action sheet 6*).

Mapping potential partners

See the mapping form on the following page for possible counter-trafficking partners that might exist in your community. See the list of resources and references at the end of this book for additional resources.

REFERENCES AND RESOURCES

Organisation for Security and Co-operation in Europe / Office for Democratic Institutions and Human Rights

2004 National Referral Mechanisms: Joining Efforts to Protect the Rights of Trafficked Persons: A Practical Handbook, OSCE / ODIHR, Warsaw, Poland, 2004.

International Organization for Migration

2007 The IOM Handbook on Direct Assistance for Victims of Trafficking, IOM, Geneva, 2007.

2004 The Mental Health Aspects of Trafficking in Human Beings: Training manual, IOM, Budapest, Hungary, 2004.

2004 The Mental Health Aspects of Trafficking in Human Beings: A set of minimum standards, IOM, Budapest, Hungary, 2004.

2001 Medical Manual, 2001 Edition, IOM, Geneva, 2001.

Zimmerman, C. and C. Watts,
2003 WHO Ethical and Safety Recommendations for Interviewing Trafficked Women, World Health Organization, Geneva 2003.

Zimmerman, C. et al.
2008 "The health of trafficked women: a survey of women entering posttrafficking services in Europe", American Journal of Public Health, vol. 98, no. 1, January 2008, pp. 55–59.

Counter-trafficking protection and assistance referral mapping form

Instructions: This form is to assist you in identifying and keeping a record of support services in your community that may serve as counter-trafficking partners. Whenever possible, **counter-trafficking specialists** are the preferred contact; however, many communities do not have specialized services for trafficked persons. When no formal counter-trafficking services are available to provide a particular service, it is important to identify **other related service providers** who can assist. Suitable groups often include those responding to violence (e.g., domestic violence or torture) or those assisting marginalized groups (e.g., migrants, refugees or the homeless).

The following list offers examples to help you think about what types of support might be available in your community. Note down telephone numbers and contact names. Whenever possible, find out about the quality and reliability of services. **The research you do now to identify possible partners will be essential when you are caring for a trafficked person and need to know whom to call.**

This form is intended as a guide and should be adapted to suit local resources.

SERVICE	CONTACT DETAILS
**Local counter-trafficking organizations**	
**Telephone hotlines**	
Counter-trafficking hotline	
Family violence hotline	
Child services hotline	
Suicide hotline	
Missing persons hotline	

**Shelters & housing services[1]**	
Counter-trafficking shelter	
Domestic violence shelter	
Children & adolescent shelter	
Migrant & refugee shelter	
Homeless shelter	
Shelters run by religious or community-based organizations	
**Health services**	
Sexual health clinics and outreach services	
Reproductive health services, including (where legal) pregnancy termination services	
General practitioners	
Alcohol or drug clinics	
Mobile clinics or outreach services	
Free health services	
**Mental health and counselling services**	
Psychologists or therapists	
Specialists in violence-related counselling	
Mental health/psychiatric clinics	
**Non-governmental and community organizations[2]**	
Counter-trafficking	
Family violence	
Rights organizations (e.g., human rights, women's or children's rights, labour)	

Refugee or immigrant services	
Social support services	
Religious or community-based organizations	
**Legal services**	
Independent lawyers (immigration and criminal)	
Community legal aid services	
**Police, law enforcement services**[3]	
Local police contacts	
Sexual and domestic violence focal point	
Children's focal point	
**Local government contacts**	
National anti-trafficking centre	
Children's offices or services	
Women's offices or services	
Immigration services	
Housing and social services	
**Embassy and consular offices**	
Embassies & consular services for most common migrant or trafficked populations	
**International Organizations**	
International Organization for Migration	

International Labour Organization	
Office of the High Commissioner for Refugees	
Office of the High Commissioner for Human Rights	
United Nations Children's Fund	
United Nations Office on Drugs and Crime	
United Nations Population Fund	
World Health Organization	
Other international agencies	
**Non-governmental organizations in other countries**	
Counter-trafficking organizations based in common countries of origin	
**Interpreters**[4]	
List likely languages required	

1 Shelters are sometimes managed by the government; other times they are managed by local or international organizations.

2 These can include local community organizations as well as international non-governmental organizations. See the UN.GIFT (Global Initiative to Fight Human Trafficking) directory of civil society partners for links to some large coalitions of organizations working in counter-trafficking: http://www.ungift.org/ungift/en/partners/civil.html

3 Officials, including police, may be involved in criminal networks that traffic human beings. Whenever possible, identify specific focal persons who work closely with and are trusted by others working on human trafficking.

4 It can also be helpful to note whether trusted colleagues speak a particular language, because formal interpreters are not always available. Use caution when selecting interpreters (*see action sheet 3*).

Action Sheet 11: Urgent care

ACTION SHEET ELEVEN

Action Sheet 11: Urgent care

RATIONALE

Urgent care includes the actions taken during a trafficked person's initial encounter with a health care provider. These actions may occur under several different scenarios. A person who has been trafficked may present to the health care provider while still in a trafficking situation – brought by the trafficker, either alone or with another trafficked person. Or the patient may seek health care independently after leaving a trafficking situation, or an individual may be rescued from the trafficking situation and referred by the police, shelter, or social service agency or organization.

Someone who has been trafficked may have acute and life-threatening injuries or illnesses, such as a badly infected wound, injuries from physical or sexual attacks or illness resulting from ingestion of medicines or poisons related to a suicide attempt. The patient may also have such chronic health problems as diabetes or asthma, which still require immediate medical attention.

During the period of acute care, particularly post-rescue, there are myriad extremely important concerns for an individual who has been trafficked, but it is important to highlight those problems that are truly medically urgent. In an acute situation, it is essential to coordinate services across various responsibilities (e.g., medical, legal, psychological) to achieve maximum benefit for the trafficked person.

If a police operation to free trafficking victims is planned, where possible police should inform the contact persons at a shelter or medical facility so that appropriate health staff is available to triage and care for trafficked persons. If a health care provider is not immediately available at an after-care facility, social services supporting the trafficked person should make a referral for medical consultation as soon as possible. Individuals

with obvious life-threatening conditions should proceed immediately to an emergency department or urgent care facility.

The actions suggested for the urgent care of trafficked persons are divided into three sections: medical treatment; mental health assessment and care; and reproductive health services.[45]

REQUIRED ACTIONS

General

- It is essential to understand trauma-informed care (*see action sheet 1*). Keep questions focused on medical issues to avoid re-traumatizing the patient. **Do**, however, listen patiently if the individual volunteers other information. Establishing a rapport with trafficked persons is the beginning of their trust and empowerment and facilitates cooperation.
- If a patient needs immediate, life-saving treatment and is not conscious or competent to give consent, it is the health care provider's duty to save the life, as is the case in any emergency situation.
- Establishing the patient's competency and capacity to make decisions is crucial and you may, for example, need to act as the patient's advocate to law enforcement. Obtaining informed consent for all treatments and procedures is important and necessary. However, there are cases when issues of competency and consent are not clear (*see action sheet 16*).
- Due to the psychological harm and possible gender-based or other forms of violence experienced by many persons who have been trafficked, it is recommended that a provider of the same sex be responsible for emergency treatment whenever possible. However, in the case of life-threatening conditions, the first priority is to stabilize the patient.
- The acute setting may be extremely stressful for someone who has been trafficked, therefore coordination of services with efficiency and compassion is of utmost importance. Keeping the patient's needs as the highest priority is paramount.
- Knowing the type of work undertaken by the trafficked person and having a sense of their living conditions may provide an important guide for the health assessment and point the way to certain diagnostic tests and procedures.

[45] See action sheets 4, 5, 12 and 15.

- Be aware of the legal implications of what is recorded in the medical file and understand local and national reporting requirements related to your specialty (*see action sheets 9 and 16*).
- You may not know ahead of time whether a patient is a trafficked person. If during the medical encounter you suspect a patient has been trafficked, there are certain actions you may consider taking (*see action sheet 6*). For example, you may have small cards or other items with phone numbers or other information for the trafficked person to take away. It is also important to prepare for potential referrals (*see action sheet 10*).

Urgent medical care

- Life-threatening injuries or conditions common in trafficked persons include dehydration, severe malnutrition, sepsis, wounds (which may be occult), head injuries (intracranial bleeding), neck and spinal injuries, exposure to toxins, altered mental status and other traumatic injuries.
- Conduct a thorough examination and screen for other injuries or conditions that require urgent care, e.g., dental trauma or infection, seizure disorders and asthma. Obtaining medications for particularly severe conditions is a priority.
- A toxicology screen is recommended because acute drug or alcohol withdrawal can be a medical emergency.
- Knowledge about the type of work the trafficked person was doing may provide clues about hazardous exposures.
- Post-exposure prophylaxis for HIV infection should be offered within 72 hours of the high-risk exposure to those patients who have had unsafe needle injections, experienced sexual abuse (including rape) or had other sexual exposure. In cases of sexual assault, consider offering emergency contraception (*see action sheet 13*).

Urgent mental health care[46]

- Assess each patient for any acute psychiatric condition that might cause them to harm themselves or staff. Examples of mental health emergencies include suicide, psychosis and drug withdrawal.
- Rule out organic causes of altered mental status (e.g., head trauma or drug withdrawal) so that treatment for the mental health condition can begin immediately.

---

[46] See action sheets 12 and 16.

- When available, consult with a qualified mental health specialist to facilitate best-practice care with appropriate medications.
- The fragile mental and emotional status often demonstrated by trafficked persons in the acute setting can confuse the clinical picture and diagnosis of any underlying clinical psychiatric condition. Trafficked people can be combative, uncooperative and even threatening as a result of their traumatic experiences. It is important to support the trafficked person's decision-making as much as possible (*see action sheets 1, 12 and 16*).

Urgent reproductive health care[47]

- Offer pregnancy tests to all females of reproductive age. Many medical interventions must be modified in light of pregnancy.
- Make emergency contraception available wherever possible to all females of reproductive age.
- Assess all trafficked persons for injuries resulting from sexual abuse and assault.
- Syndromic care of sexually transmitted infections may be appropriate where good laboratory testing is unavailable. If you are unsure whether a patient can or will follow up for care, medicine needed to treat such suspected infection should be given to the patient, along with detailed instructions.

Collect minimum forensic evidence

- Local legal requirements and laboratory facilities determine what, if any evidence should be collected for use in criminal prosecution, and who may collect it. In most countries, specially trained health professionals who work with law enforcement collect forensic evidence. Health workers should not collect evidence that cannot be processed or that will not be used.
- Counsel the survivor about taking evidence for criminal prosecution. It is essential that health providers know in advance where to refer victims of sexual violence (*see action sheet 10*).
- Assure the patient that the information will only be released to the authorities with his or her consent or by legal order.

[47] See action sheet 13.

- Keep a careful written record of all findings of the medical examination. This may assist the medical management of the survivor as well as any subsequent legal investigation. The medical chart may be part of the legal record and in some locations may be submitted as evidence in a court case.

REFERENCES AND RESOURCES

Inter-Agency Standing Committee

2005 *Guidelines for Gender-Based Violence Interventions in Humanitarian Settings: Focusing on prevention of and response to sexual violence in emergencies*, IASC, Geneva, September 2005.

United Nations Children's Fund

2005 *Manual for Medical Officers Dealing with Child Victims of Trafficking and Commercial Sexual Exploitation (Manual for Medical Officers Dealing with Medico-Legal Cases of Victims of Trafficking for Commercial Sexual Exploitation and Child Sexual Abuse)*, UNICEF and the Department of Women and Child Development, Government of India, New Delhi, 2005.

United Nations High Commissioner for Refugees

1999 *Reproductive Health in Refugee Situations: An inter-agency field manual*, UNHCR, Geneva, 1999.

World Health Organization

2005 *Sexually Transmitted and Other Reproductive Tract Infections: A guide to essential practice*, WHO, Geneva, 2005.

1997 *Syndromic Case Management of Sexually Transmitted Diseases: A guide for decision-makers, health care workers and communicators*, WHO Regional Office for the Western Pacific, Manila, 1997.

World Health Organization and International Labour Organization

2007 Post-Exposure Prophylaxis to Prevent HIV Infection: Joint WHO/ILO guidelines on post-exposure prophylaxis (PEP) to prevent HIV infection, WHO, Geneva, 2007.

Zimmerman, C. and C. Watts,

2003 *WHO Ethical and Safety Recommendations for Interviewing Trafficked Women*, World Health Organization, Geneva 2003.

Action Sheet 12:
Mental health care

ACTION SHEET TWELVE

Action Sheet 12: Mental health care

RATIONALE

Mental health care includes the range of support and treatment that enables a person to achieve a state of positive emotional well-being. Cultural variations, professional differences and perceptions of stigma all affect how 'mental health' is defined (diagnostic criteria for most mental disorders were developed in industrialized countries). Internationally, there are similarities and differences in symptoms of mental health problems, cultural attitudes towards their causes and priorities for treatment. In some settings it may be particularly important to emphasize that someone needing mental health care has symptoms that are an understandable reaction to severe stressors and that will likely improve with time and support: that is, having mental health needs does not simply equate with the individual 'losing their mind' or with having a condition that might stigmatize them in their community.

Health care providers may encounter a trafficked person with mental health problems at any stage of the trafficking cycle. For example, a trafficked person who has returned to the country of origin may be referred for depression. Alcohol dependency may be detected when someone suspected of being in a trafficking situation consults for a physical illness. A patient who attempted suicide or suffers acute psychosis may reveal that she or he is in a trafficking situation.

In some situations it is necessary to provide crisis care with very little background information. In this scenario, the health care provider's responsibility is to maximize the benefits of the sole encounter by providing the best possible treatment, taking into account that the person may be unable or unwilling to return for follow-up. For example, a health care provider can give as much information as possible about symptoms and prescribed treatment and tell the patient how to access support. At other times it may be possible to provide longer-term care and advocacy support. This could

include giving advice to the trafficked person, and to any trusted individual or agency supporting them, about monitoring recovery and about opportunities for integration and rehabilitation.

Supporting psychological recovery often includes three stages, although there is no clear timeline for these stages in the recovery of trafficked persons and an individual may move between them multiple times. In the first stage, it is essential to restore an individual's sense of safety and personal control over decision and events. At some point, those able to enter the second stage begin to address their traumatic experience and its impact on their mental health. The last stage includes receiving support reintegrating into their original or adopted community. However, mental health status may fluctuate during reintegration, e.g., if the trafficked person takes part in prosecution proceedings or has a difficult encounter with a family member. Severe mental distress may appear or reappear years later.

In some cases, someone who has been trafficked may experience multiple debilitating psychological symptoms that meet the criteria for a mental disorder. Such mental disorders may have developed prior to the trafficking experience or might have been induced or exacerbated by trafficking. This action sheet covers non-specialist support for mental health problems and also refers to assessment and treatment of mental disorders.

REQUIRED ACTIONS

Essential actions for providing first-line support

- Develop trust and rapport. Show empathy by listening to the trafficked person in an accepting and non-judgmental way. Show genuine compassion. Provide care in a manner that recognizes their individual, cultural and religious needs.
- Provide care in a manner that recognizes their individual cultural and religious needs (see action sheet 2).
- Ask about psychological symptoms likely to be common in persons who are in or who have been in a trafficking situation. These may include fearfulness, worry, sadness, guilt, shame, anger, bereavement, memory loss, hopelessness, reliving experiences (through flashbacks or nightmares), loss, emotional numbing, feelings of being cut-off from others, being 'jumpy' or easily startled and suicidal ideation. Find out how severe these symptoms are by asking how often they occur and how much they impair the person's well-being and functioning.

- Ask about recent misuse of alcohol or other substances. These are common problems for trafficked persons and are also found in combination with other problems such as depression and physical complaints. In many settings, trafficked persons are provided with alcohol and drugs during their abuse to reduce their resistance and increase their dependence on traffickers. Their use may have become a way of coping with intense, painful emotions.
- Assess whether the trafficked person has access to suitable, secure support. This may be available through existing community or religious support systems or, if appropriate and safe, through some members of the patient's family. Some patients will not need or want emotional support, but they should be informed about how to access such support.
- Avoid inappropriate pathologization. Be a good listener and provide other basic psychological support including information about symptoms being (in most cases) a normal reaction to stress, information on the likelihood of recovery and advice about the benefits of safe support from trusted persons. Show respect for how the individual has coped thus far, even if their coping mechanisms were maladaptive, e.g., substance abuse and self-harm.
- Discuss how to re-establish basic routines that have been denied, e.g., mealtime and sleep routines. Encourage use of positive means of coping (e.g., culturally appropriate relaxation methods). Advise the patient that if symptoms persist he or she will be referred for further help.
- Use non-drug approaches for mental health symptoms whenever possible, for instance for medically unexplained somatic symptoms. Avoid benzodiazepines as they quickly lead to dependence; however, their use may be justified with caution for certain clinical problems such as severe insomnia or during alcohol withdrawal. On the other hand, if the patient has many severe disabling psychological symptoms and/or indicates an intention to commit suicide, refer to the section below on access to care for mental disorders and consider referring the patient quickly to a clinician trained in mental health care.
- Give non-judgemental, supportive advice about alcohol and substance misuse. Help patients set goals for ceasing consumption and encourage them to express their own motivations for reducing consumption. When someone

is physically dependent on alcohol they need referral to a trained specialist for support in abstaining completely.[48] Coming off illicit drugs and alcohol abruptly can be very uncomfortable and be associated with cravings, agitation, fatigue, poor sleep, anxiety and muscle pain.

- Describe in a straightforward and honest way the available facilities and services. If the patient declares that they do not want to use the available facilities, or you suspect this to be so, still provide contact information covering agencies/ organizations assisting trafficked persons, shelter options if appropriate and emergency medical and social services in case the trafficked person wishes to seek help at a future time.
- Expect a trafficked person to show strong negative emotions, including anger, and do not expect the patient to necessarily show appreciation at this early stage. Anger is a common and normal reaction to having been trafficked, and may be turned on the health care provider. Additionally, some trafficked persons will have long-standing vulnerabilities that pre-date the trafficking experience. Treat the trafficked person fairly without responding emotionally to their anger or if they appear unappreciative or excessively needy. Maintain professional boundaries (see action sheet 8). Acknowledge that the person seems angry; be willing to reflect on this but do not take this personally or react by being angry or dismissive in return.
- Be prepared to listen to details of abuse if the patient chooses to confide in you. Acknowledge the person's distress and their concerns. The traumatic account is often confused or inconsistent, which may be due to emotional distress and trauma rather than lying. Convey the message that the trafficked person was a victim of a crime that was not their fault. If the patient expresses self-blame, encourage them to see that they were not to blame (e.g., through such questions as 'Were you deceived?' or 'Were threats used?'). Emphasize that it is the perpetrator who is to blame.
- Do not initiate a brief single session of talking that focuses on traumatic experiences (a debriefing) unless you have been trained in cognitive behaviour treatment. This is unhelpful and can in fact be harmful. Instead ask only relevant, non-judgemental and non-intrusive questions that seek essential

[48] "Screening and brief intervention for alcohol problems in primary health care", web information available at « www.who.int/substance_abuse/activities/sbi/en/index.html » WHO, Geneva, undated, accessed 3 January 2009.

clarification. Do not force the trafficked person to reveal more details than he or she wishes to disclose at the time.

- Use problem-solving approaches. Empower the trafficked person rather than tell them what choices you think they should make. Discuss their problems to aid clarification, brainstorm together, helping them identify their own options for coping or developing solutions. Discuss the advantages, disadvantages and potential consequences of the different options. Respect the patient's choices about decisions, including about seeking services. In collaboration with the trafficked person, agree on short- and long-term goals for rehabilitation. Discuss their strengths as well as their needs.
- Involve the trafficked person in decision-making as much as possible (see action sheet 16 for a discussion of capacity).
- Involve the trafficked person in the decision to make referrals to appropriate professionals and agencies. This should preferably be to services with which your organization has established procedures for cooperation and a common understanding. Accompany the patient if he or she would like you to do so. With the trafficked person's permission, follow up the work of outside agencies. Maintain confidentiality (see action sheet 9).

Essential training for providers working regularly with trafficked persons

Training in approaches to psychosocial support and mental health care should be offered to staff and integrated into their daily practice. Seminars for staff should cover the following: the phenomenon of trafficking and its effects on victims; the importance of treating trafficked persons with dignity; interview techniques; basic knowledge about how to deal with persons who are very anxious, angry, suicidal, withdrawn or psychotic; empathic listening and rapport-building skills; providing care while using interpreters; problem-solving skills; setting goals; assessing the mental state of a trafficked person; developing confidence in asking about a range of psychological symptoms; recognizing and diagnosing mental disorders and providing initial management using essential drugs; education about symptoms; non-pharmacological management of medically unexplained somatic complaints; organizing small group activities; the roles and responsibilities of other professionals; and how to refer to these other professionals while maintaining confidentiality and upholding the patient's security. Useful resources include *Where There is No Psychiatrist: A mental health care manual* (*see reference list*).

Short theoretical training is not sufficient for those who will be counselling trafficked persons. Instead, provide practical training and follow this up with extensive on-the-job supervision. Staff should be informed about appropriate available health and social services (see *Mental Health Atlas 2005*, issued by the World Health Organization, for mental health resources in all countries) and to document mental health problems in their clients using simple categories as part of routine data collection.

Providing further psychosocial support as recovery proceeds

- Provide ongoing support and address issues raised by the patient.
- Follow up and review the care plan and the goals that you set together, discussing pros and cons for change; aim to encourage the trafficked person to express her or his own motivations for change. Work on building self-esteem and on assuaging feelings of guilt and shame over the abuse. Support groups, if available, could also help address these issues.
- If contact with family and friends remains an unresolved issue, it may become an area to discuss as the patient begins to overcome difficult emotions. Keep in mind the potential dangers if any close contacts of the patient were perpetrators of the trafficking.
- Provide information about appropriate activities, support groups, religious organizations, housing, relevant agencies, employment and education.

Access to care for mental disorders

- Mental disorders may have developed before the trafficking experience or have been induced or exacerbated by the trauma. These disorders include disabling forms of post-traumatic stress disorder, depression and anxiety; mental disorders due to harmful use of alcohol or other substances; psychoses; severe behavioural and emotional disorders in children and adolescents (see below); and any other severe mental health problem or serious risk behaviour such as suicidal intent.
- Recognize serious symptoms. The patient may reveal distress directly by talking about it, or through altered behaviours such as agitation, inability to sleep or to concentrate, confused speech, withdrawal, self-neglect, self-harm or – rarely – threats or actions to harm others (see action sheet 16 for information on capacity).

- Assess risk. When working with persons with severe social difficulties and/or mental health problems, particularly where mood is affected, always ask if things have gotten so bad that they think life is not worth living. If they indicate this is the case, ask about any current intentions or plans to commit suicide. Also ask how they are coping with caring for any children. If they indicate current difficulties, ask if they think there is any risk they may harm their children. Refer to specialist care and other locally existing social services those with suicidal intent or those who you consider at risk of harming others because of a mental disorder. Patients may express intense anger and desire for revenge; in these cases, assess risk of violent, homicidal or illegal actions.
- Refer patients for treatment beyond medication. Interventions for mental disorders will commonly combine skilled psychological treatment with social support (e.g., for instance assistance with housing, employment or childcare, or working with family issues) and sometime medication. Services for trafficked persons need to be set up to link with such specialized mental health providers as psychologists and psychiatrists and with existing social and informal systems.
- Refer to specialists who are aware of evidenced-based treatment for mental disorders in settings where these resources are available. For instance, disabling post-traumatic stress disorder should be treated by a mental health professional. Drugs should not be used as a routine first-line treatment for severe post-traumatic stress disorder. However, antidepressant drugs should be used if the individual cannot start psychological treatment or if psychological treatment has not led to improvement, ideally in combination with psychological treatment. Treatment for mild depression (i.e., with several symptoms of depression but where the patient is still able to cope reasonably with some activities) may be support, information about symptoms and problem-solving approaches by a trained general health provider, or a mental health care provider.
- Encourage adherence if the patient has started taking psychotropic medication, and advise the patient to avoid combining this medication with such potentially dangerous substances as alcohol. If the patient has had thoughts of suicide, monitor these as the person receives mental health treatment.

- To optimize recovery in trafficked persons who are being treated for a mental disorder, continue to offer support using the general approaches outlined in the 'essential actions' section, above and continue to aid integration into the community. A trafficked person with a psychosis or other disabling mental disorder should be provided with a reintegration programme or other rehabilitation for trafficked persons that is tailored to their needs.

Mental Health and Trafficked Children and Adolescents

Children and adolescents may be trafficked for a variety of purposes, including forced labour, sexual exploitation, as child brides or child soldiers. For many children, being trafficked may not be their first encounter with abuse. It is not uncommon for trafficked children to come from dysfunctional situations including violence, alcohol abuse, or to have witnessed violence prior to being trafficked. During the trafficking situation a child or adolescent may have been physically or sexually abused or seen family members injured, raped or killed.

How a trafficking experience will affect each child varies depending on, for example, the age at which the child was trafficked, how long the child remained in the abusive situation, the child's circumstances prior to being trafficked, the many interactions and events that occurred along the way and each child's own resilience. Some of the most serious psychological consequences result from multiple or chronic exposure to abuse.

When children are separated from family and exposed to violence, developmental difficulties or regression may occur. A child's speech may regress or the child may begin to stammer when anxious. Sleep may be disturbed by nightmares. Children may become irritable or withdrawn; they may cry frequently or show behaviour such as rocking movements, thumb-sucking or hair-pulling or appetite loss. Younger children may begin to be incontinent at night (enuresis). They may seem confused and not understand past or present events. Older children may be sad, tearful, and irritable, demanding, aggressive or sexualized.

Sexual abuse has particularly profound and long-term psychological effects, including loss of self-esteem, learned helplessness, confusion about identity – particularly sexual identity. Like adults, a child's reactions may include depression, emotional disorders or anxiety and post-trauma reactions. Prominent post-trauma symptoms in younger children are re-enacting traumatic events through drawing or playing, displaying agitated behaviour and having temper tantrums. In older children, post-trauma reactions are

similar to those of adults and include nightmares, flashbacks and distress when traumatic memories are triggered by current events, avoidance of traumatic memories and hyper-arousal (poor concentration, irritability). Teenagers, particularly girls who have been sexually exploited or who were trafficked to become child brides, may develop eating disorders. Children who are sexually abused are also at increased risk for substance abuse and suicide.

For children and adolescents in particular, it is **extremely** important that all services (including health care but also those related to shelter and protection) be guided by the need for **consistent care to enable recovery from the traumatic experience**. Close coordination with others providing assistance to trafficked children (*see action sheet 10*) is essential to ensure that mental health care begins **immediately** and that other parts of the comprehensive care package do not have an adverse impact on the mental health work being done with the child. In the case of sexual abuse and sexual exploitation in particular, the less time that passes between the sexual trauma and the initiation of therapy, the better for the child.

Supporting a child's mental health depends significantly on being able to provide consistent psychological care that is accompanied by other forms of child-appropriate support that 'normalises' their lifestyle, including shelter, protection, health care and education. Dependable, stable environments are the basis of a child's psychological (and physical) recovery from distressing or traumatic experiences.

Children should be assured that what happened to them is not their fault; that they are now free from their abusers; and that every attempt will be made to keep them safe. Identifying secure housing where the child feels safe is a priority for trafficked children – as for any abused child.

REFERENCES AND RESOURCES

Day, J.H. et al.

2006 *Risking Connection in Faith Communities: A training curriculum for faith leaders supporting trauma survivors*, Sidran Institute Press, Baltimore, Maryland, USA, 2006.

Inter-Agency Standing Committee

2007 *IASC Guidelines on Mental Health and Psychosocial Support in Emergency Settings*, IASC, Geneva, 2007, pp. 116–131. Section 6.1: Include specific psychological and social considerations in provision of general health care. Section 6.2: Provide access to care for people with severe mental disorders.

2005 *Guidelines for Gender-Based Violence Interventions in Humanitarian Settings: Focusing on prevention of and response to sexual violence in emergencies*, IASC, Geneva, September 2005.

International Labour Organization
2005 *A Global Alliance Against Forced Labour: Global report under the follow-up to the ILO Declaration on Fundamental Principles and Rights at Work,* International Labour Conference, 93rd Session 2005, Report I (B), International Labour Office, Geneva, 2005.

Nikapota, A. and D. Samarasinghe
1991 *Manual for Helping Children Traumatized by Conflict*, UNICEF, Sri Lanka, 1991.

Patel, V.
2003 *Where There is No Psychiatrist: A mental health care manual,* Gaskell, London, 2003.

Smith P. et al.
1999 *A Manual for Teaching Survival Techniques to Child Survivors of Wars and Major Disasters*, Children and War Foundation, Bergen, Norway, 1999.

United Nations Population Fund
2008 *Mental, Sexual & Reproductive Health: UNFPA Emerging Issues*, UNFPA, New York, USA, 2008.

World Health Organization
2005 *Mental Health Atlas, Revised* Edition, WHO, Geneva, 2005.
2005 *Child and Adolescent Mental Health Policies and Plans*, mental health policy and service guidance package, WHO, Geneva, 2005.
2005 *WHO Resource Book on Mental Health, Human Rights, and Legislation*, WHO, Geneva, 2005.
1998 *Mental Disorders in Primary* Care, A WHO Educational Package, WHO, Geneva, 1998.

"Screening and brief intervention for alcohol problems in primary health care", web information available at « www.who.int/substance_abuse/activities/sbi/en/index.html » WHO, Geneva, undated, accessed 3 January 2009.

Action Sheet 13: Sexual and reproductive health

ACTION SHEET THIRTEEN

Action Sheet 13: Sexual and reproductive health

RATIONALE

Many people are trafficked for purposes of sexual exploitation; trafficked persons in other types of exploitation may also be sexually abused as a form of coercion and control. As a consequence, trafficked persons, regardless of gender or age, are at risk of developing complications relating to sexual and reproductive health. Addressing sexual and reproductive health issues is therefore an important component of caring for someone who has been trafficked. It is essential that every trafficked person receive timely, competent and comprehensive sexual and reproductive health services even if they were not trafficked explicitly for sexual exploitation.[49] A trafficked person's visit to obtain sexual and reproductive health services may be his or her only chance to receive support, care and information regarding health and safety.

The sexual and reproductive health of men, women and children who have been trafficked, particularly those who have been sexually exploited, is a highly sensitive area of health assessment and care. The examination of the reproductive system can be difficult and can possibly re-traumatize someone who has been sexually abused. The ability to have a normal reproductive life, including a family and children if desired, is often a concern of many sexually exploited persons. Sexual abuse can be very stigmatizing for the victim. Health care providers must be supportive of all persons who have experienced such abuse and liaise closely with mental health care providers.

Even when health care providers offer explanations about medically necessary tests and treatments, trafficked persons may or may not choose to comply with recommended tests and treatments related to their sexual and reproductive health. Having some control over what happens to their bodies

[49] When children or adolescents have been sexually abused or exploited, mental health care should begin as soon as possible (*see action sheet 12*).

can be an empowering experience for people who have been trafficked, and trauma-informed care recognizes the tension between immediate benefit to the patient of a particular intervention and the long-term benefit of the patient's autonomy and empowerment (*see action sheet 1*).

Persons who have been trafficked sometimes have very little knowledge about human anatomy and physiology. For example, they may not know how antibiotics or contraception work, or how much damage a sexually transmitted infection can do to their bodies, or that some are curable and others are not. Therefore, explanations about medical problems and procedures should be clear and comprehensible.

Finally, people who have been trafficked, like many vulnerable populations, may be unaware of their sexual and reproductive rights and the health risks they face. Providers should be prepared to offer related information.

REQUIRED ACTIONS

General

- Follow recommendations for trauma-informed care (*see action sheet 1*) and patient-centred care (*see action sheet 2*).
- Interview individuals who have been trafficked regarding sexual and reproductive health services they have already received and any specific concerns they have about reproductive and sexual health.
- Make information of the potential benefits of all procedures and tests as well as their possible complications available to patients in a manner that is both clear and respectful. Explain to patients the potential complications of **not** addressing sexual and reproductive health issues.
- Carry out interviews and examinations in a patient's own language, via interpreters if necessary (*see action sheet 3*).
- Introduce yourself and explain all procedures before, during and after they occur. Be clear about what you expect from the patient. Make clear to the patient that he or she can refuse treatment at any time. Children and adolescents should also have a voice (*see action sheets 5 and 12*).
- Ask the patient if he or she would like to have a support person present at any time.[50]
- Be honest about complications related to the patient's medical problems and give realistic hope about future reproductive capabilities.

[50] Be careful, though, if you suspect someone might be trafficked and they are accompanied (*see action sheet 6*).

- Provide information on reproductive and sexual rights to inform women about their options to continue or terminate a pregnancy, in accordance with the legal context of the country.
- Take into account the possibility that a trafficked person may have had or may be having consensual, unprotected intercourse during or after the trafficking experience.
- Make clear and simple information on sexual and reproductive health matters available in the patient's own language.
- Health practitioners must be aware of local and cultural beliefs and practices related to sex and sexual health.
- Make referrals, with the patient's consent, to other services including social and emotional support, security, shelter, etc. (*see action sheet 10*). It is essential to know in advance where to refer victims of sexual violence safely.
- Communicate clearly and with sensitivity about sexual and reproductive health problems. Because of the intimate nature of sexual and reproductive health examinations and care, it is important keep the patient informed of procedures and diagnosis, and involved in the decision-making in ways that are sensitive to age, social and cultural backgrounds (*see action sheets 2, 5 and 16*). When treating children and adolescents, providers should be very clear about laws and practices pertaining to consent, emancipation, capacity and competency.

Examination

- Conduct the medical examination only with the patient's consent. The exam should be compassionate, confidential, systematic and complete, following a protocol that is explained before and during the exam. It is important to inform the patient about what you are doing **as** you are doing it.
- Every medical examination must involve a practitioner or chaperone of the gender preferred by the patient, and, if requested, an additional support person.
- Reassure patients that they are in control of the pace of the examination and that they have the right to refuse any aspect of it. Explain that the findings are confidential, but be honest about which results must or may be reported to comply with legal obligations.
- Conduct a complete pelvic examination on trafficked women and girls, because they may suffer from vaginal and perineal tears as well as other external and internal injuries due to

sexual abuse or unsafe abortions. Care should be taken to reduce as much pain and discomfort as possible.

- Conduct a complete genital and rectal examination of men and boys, who may also suffer trauma from sexual abuse.
- Evaluate pregnant women who have been trafficked for any risks to the woman and foetus; pregnant women should immediately have a complete antenatal exam. Arrange for a safe delivery and for post-natal follow-up.
- At the time of physical examination, normalize any somatic symptoms of panic or anxiety, such as dizziness, shortness of breath, palpitations and choking sensations that are medically unexplained (i.e., without organic cause). This means explaining in simple words that these bodily sensations are common in people who are very scared after having gone through a very frightening experience, and that they are not due to disease or injury; explain that they are part of experiencing strong emotions related to their trauma (*see action sheet 12*).
- **Collect minimum forensic evidence**
    - o Local legal requirements and laboratory capabilities determine if and what evidence should be collected for use in criminal prosecution, and by whom. If available, it is best to have forensic evidence collected by specially trained forensic professionals. Health workers should not collect evidence that cannot be processed or that will not be used.
    - o Provide accurate information about the purposes and use of evidence for criminal prosecution. Avoid making false promises or raising expectations unrealistically about criminal proceedings.
    - o Ensure the patient that the information will only be released to the authorities with their consent.
    - o Keep a careful written record of all findings of the medical examination of all cases of sexual violence; this may assist the management of care and any subsequent legal investigation. The medical chart may become part of the legal record and submitted as evidence in a court case.

Treatment

- Determine whether or not the patient is able to follow up with you for treatment (Are they still in the trafficking situation? Facing deportation? Being transferred to another centre?). If follow-up is unlikely, if possible complete or send along

(if multi-day therapy) treatment for any sexually transmitted infection or other problem.

- Where possible, see that care of sexual and reproductive health and gender-specific issues are followed up over the long term. These may include regular PAP smears, breast cancer screenings and tests for erectile dysfunction, etc.

Specific sexual and reproductive health issues

- Contraception
  - o Provide trafficked persons with information about contraception options. Choice of contraceptive should take into account their needs for family planning, prevention of sexually transmitted infections, as well as their ability to continue use of a preferred contraceptive in their current situation.
  - o Counselling should include the fact that contraception without condoms will not protect against sexually transmitted infections.
  - o Counsel every woman of reproductive age about which emergency contraceptive options are available locally; if appropriate, provide them with emergency contraception.
- Pregnancy tests and obstetric care
  - o Make pregnancy testing available to all women of reproductive age. Urine test strips are adequate.
  - o Determine the length of gestation for all women who are pregnant; counsel these women on their options to continue or terminate the pregnancy.
  - o For women who continue their pregnancies, discuss and provide antenatal care, delivery, and postpartum care using World Health Organization guidelines.
  - o Provide women who choose to terminate a pregnancy with information about safe, locally available options when termination is legal and possible.
- Prevention and treatment of HIV and sexually transmitted infections
  - o Identify in advance where patients can access voluntary HIV counselling, testing and treatment in a safe and confidential setting. Comprehensive access to HIV prevention, treatment, care and support is essential.
  - o Consider providing post-exposure prophylaxis for HIV for all who have had unprotected sexual contact within the prior 72 hours.

- o When managing and preventing sexually transmitted infections, keep in mind that trafficked persons may have been infected with a drug-resistant organism – or may have developed drug-resistance and infection through inappropriate treatment – and may not respond to first-line antibiotics. Where possible, provide follow-up visits to ensure effective treatment.

Sexual and reproductive health may be among the primary health concerns of those who have been sexually abused. Rapidly attending to a trafficked person's sexual and reproductive health can contribute to their overall health, particularly mental health (see *action sheet 12*).

REFERENCES AND RESOURCES

Hossain, M. et al.
2005 Recommendations for Reproductive and Sexual Health Care of Trafficked Women in Ukraine: Focus on STI/RTI care, First Edition, London School of Hygiene & Tropical Medicine and the International Organization for Migration, 2005.

Inter-Agency Standing Committee
2005 *Guidelines for Gender-Based Violence Interventions in Humanitarian Settings: Focusing on prevention of and response to sexual violence in emergencies*, IASC, Geneva, September 2005.

United Nations
1979 Convention on the Elimination of All Forms of Discrimination Against Women, adopted in 1979 by General Assembly Resolution 34/180, entry into force September 1981.

United Nations Population Fund
2002 *Trafficking in Women, Girls and Boys: Key issues for population and development programmes*, Report on the Consultative Meeting on Trafficking in Women and Children, Bratislava, Slovak Republic, 2-4 October 2002, UNFPA, New York, 2003.

United States Centers for Disease Control and Prevention
2006 "Sexually Transmitted Diseases Treatment Guidelines, 2006", *Morbidity and Mortality Weekly Report*, vol. 55, no. RR-1, 4 August 2006.

World Health Organization

2007 *Guidance on Global Scale-Up of the Prevention of Mother-to-Child Transmission of HIV: Towards universal access for women, infants and young children and eliminating HIV and AIDS among children*, WHO, Geneva, 2007.

2007 *WHO Recommended Interventions for Improving Maternal and Newborn Health*, Integrated Management of Pregnancy and Childbirth Guidelines, WHO, Geneva, 2007.

2007 *The WHO Strategic Approach to Strengthening Sexual and Reproductive Health Policies and Programmes*, WHO, Geneva, 2007.

2006 *Pregnancy, Childbirth, Postpartum and Newborn Care: A guide for essential practice*, Integrated Management of Pregnancy and Childbirth Guidelines WHO, Geneva, 2006.

2005 *Emergency Contraception*, Fact Sheet no. 244, WHO, Geneva, revised October 2005.

2005 *Sexually Transmitted and Other Reproductive Tract Infections: A guide to essential practice*, WHO, Geneva, 2005.

2005 *WHO Online Sex Work Toolkit: Targeted HIV/AIDS prevention and care in sex work settings*, web information available at « www.who.int/hiv/pub/prev_care/swtoolkit/en/ » WHO, Geneva, 2005.

2002 *Guidelines for medico-legal care for victims of sexual violence*, WHO, Geneva, 2003.

World Health Organization and International Labour Organization

2007 *Post-Exposure Prophylaxis to Prevent HIV Infection: Joint WHO/ILO guidelines on post-exposure prophylaxis (PEP) to prevent HIV infection*, WHO, Geneva, 2007.

World Health Organization Department of Reproductive Health and Research and Johns Hopkins Bloomberg School of Public Health Center for Communication Programs INFO Project

2008 *Family Planning: A global handbook for providers (2008 Edition)*, CCP and WHO, Baltimore and Geneva, 2008.

Zimmerman, C. et al.

2003 *The Health Risks and Consequences of Trafficking in Women and Adolescents: Findings from a European study*, London School of Hygiene and Tropical Medicine, London, 2003.

ACTION SHEET FOURTEEN

Action Sheet 14: Disability

Action Sheet 14: Disability

RATIONALE

Persons with disabilities may be vulnerable to being trafficked, and in some cases they may be targeted for exploitation because of their disability. Furthermore, some people may develop a disability as a result of their experiences in the trafficking situation. The current lack of statistical data on persons with disabilities who have been trafficked makes it difficult to determine the scope of the problem.

Nevertheless, health care providers need to recognize that trafficked persons may have disabilities, and they should be prepared to meet the particular health needs of trafficked persons with disabilities. Globally, there are 650 million people – around 10 per cent of the world's population – living with disabilities. In many parts of the world, disabled persons are among those trafficked persons who receive assistance.

There is some evidence to suggest that different forms of disability are linked with different forms of trafficking. In many parts of the world, traffickers target children and adults with physical disabilities for forced begging, because a visible disability may elicit public sympathy.[51] Amputees or persons disfigured by landmines have been found in situations of forced begging,[52] and in some countries persons trafficked for forced begging have been found to wear camouflage uniforms, to suggest they may be veterans

[51] See Kropiwnicki, Z. D., *Children Speak Out: Trafficking risk and resilience in southeast Europe (regional report),* Save the Children in Albania, Tirana, Albania, July 2007; *Reference Guide on Protecting the Rights of Child Victims of Trafficking in Europe*, UNICEF, 2006; United Nations Educational, Scientific and Cultural Organization, Human Trafficking in Nigeria: Root causes and recommendations, Policy Paper No. 14.2(E), UNESCO, Paris, 2006. Surtees, R., *Second Annual Report on Victims of Trafficking in South-Eastern Europe*, IOM, Geneva, 2005.

[52] U.S. Department of State, *Trafficking in Persons Report*, June 2007.

of armed conflict.[53] There are also reports of persons with physical, hearing or visual impairments being trafficked into forced begging in different parts of the world.[54] In addition, there are some reports of traffickers purposely mutilating their victims, particularly children, so they will be or appear disabled, or forcing them to sit in a wheelchair or to take drugs in order to appear disabled.[55] In other reports, children without disabilities have been forced to work in begging with disabled adults.[56] Traffickers may target persons with mental and intellectual impairments because of their limited capacity to gauge risk or escape trafficking; there is some evidence to suggest that persons with disabilities who have been trafficked are more often in situations of sexual exploitation, rather than forced begging.[57] However, persons with mental and intellectual impairments have been found in other forms of forced labour, including agricultural work[58] and brick making.[59]

Disability plays an important role in creating vulnerability to trafficking, particularly in countries with high levels of discrimination and abuse against persons with disabilities. Some newspapers have reported that children with disabilities face increased risk of being trafficked in some countries, because parents of children with disabilities may abandon their children or 'sell' them to traffickers.[60] Children with disabilities are particularly vulnerable and many face discrimination within their own cultures.[61] Trafficked persons who have disabilities may face multiple forms of discrimination and marginalization. Their disability has the potential to compound discrimination related to gender, ethnicity and immigration status; it can exacerbate the stigma of having been trafficked or the stigma associated with the work they were compelled to do while in the trafficking situation. Trafficked persons with disabilities have special needs, which means that the health care provider must consider a number of additional aspects of care. The health provider must also be prepared to advocate for specialized services for such persons, where required.

---

[53] Tiurukanova, E. V. and the Institute for Urban Economics, *Human Trafficking in the Russian Federation: Inventory and analysis of the current situation and responses*, United Nations Children's Fund, International Organization for Migration and Canadian International Development Agency, Moscow, March 2006.

[54] Ibid.

[55] Surtees, R., *Second Annual Report on Victims of Trafficking in South-Eastern Europe*, IOM, Geneva, 2005.

[56] Ibid.

[57] Surtees, R., *Second Annual Report on Victims of Trafficking in South-Eastern Europe*, IOM, Geneva, 2005; Zimmerman, C., *Stolen Smiles: The physical and psychological health consequences of women and adolescents trafficked in Europe*, London School of Hygiene and Tropical Medicine, London, 2006.

[58] International Organization for Migration, Trafficking in Persons: An analysis of Afghanistan, IOM, Kabul, Afghanistan, 2003.

[59] Hu, Y. "1,340 saved from forced labor", *China Daily*, 14 August 2007, p. 3.

[60] Ray, John, "Disabled children sold into slavery as beggars, Chinese racketeers living well by exploiting poverty, ignorance", newspaper article, in « guardian.co.uk » and in *The Observer*, 22 July 2007.

[61] West, A. "At the margins: street children in Asia and the Pacific", Asian Development Bank, Poverty and Social Development Papers, no. 8, October 2003.

REQUIRED ACTIONS

When working with trafficked persons who have physical or mental impairments, the standards of care described in other parts of this handbook should be applied. Persons who have been trafficked and who are also living with a disability have the same health needs as other trafficked persons and the same right to quality health care. For them to enjoy this right some accommodations may be necessary to remove barriers to access and promote non-discrimination, physical access to health care services and access to information. In particular, health care providers should adhere to the principle of non-discriminatory care. Persons with disabilities may also have specific health care needs related to their disability, in addition to needs stemming from the trafficking experience.

Commit to client-centred, rights-based care

- Persons with disabilities have human rights that are protected under international law, including the right to freedom from exploitation, violence and abuse[62] and the right to live independently, the right to be included in the community, and the right **not** to be obliged to live under a particular living arrangement.[63]
- It is important that trafficked persons who have disabilities are actively involved in making choices about their care plan and are able to voice concerns, either directly or through their chosen representative. That a person has a disability should not lead to an assumption of passivity or helplessness but to an assessment of the person's capacity to make decisions for themselves, and a commitment to the fulfilment of this capacity (*see action sheet 16*).
- In developing a health care plan, considerations should include whether standard care options need modification in order to accommodate the person's disability. They must also take into consideration any additional care concerns that need to be addressed, e.g., help with bathing, cooking, cleaning or shopping.
- As with all trafficked persons, carefully explain the purpose of consultations, interventions and services. Regardless of

[62] United Nations, Convention on the Rights of Persons with Disabilities, adopted on 13 December 2006 by General Assembly Resolution A/RES/61/106, opened for signature 30 March 2007, New York, Article 16, available at http://www.un.org/disabilities/convention/conventionfull.shtml

[63] Ibid., Article 19.

the patient's level of the disability, do not to talk down to or infantilize him or her; yet stay aware of any potential limitations of communication related to their disability. Interpreters should also be trained to work with persons with disabilities in a non-discriminatory manner (*see action sheet 3*).

- Non-discriminatory practices should be integrated into medical service protocols.

Acknowledge the disability when assessing health needs

- Acknowledge the patient's disability, and use the person's preferred language to talk about it.
- When assessing the health needs of a trafficked person who has a disability, take care that signs of abuse and exploitation are not mistakenly attributed to a disability and overlooked. Symptoms of trauma may manifest themselves differently depending upon whether a person has a disability (*see action sheets 1 and 12*). Abuse experienced in the trafficking situation **could exacerbate or mask** symptoms associated with a disability.
- If appropriate and available, explore care options related to individual impairments, for example surgery and speech and language therapy for patients with cleft palate, because previous access to health care for many trafficked persons has been limited. Health-related rehabilitation can contribute to a better quality of life and can help lower the risk of re-trafficking for some disabled persons.[64]

Consider the practicalities of providing services

- Depending on the nature of an individual's disability, it may be necessary to adapt standard therapeutic techniques or to have a greater number of sessions, which may also need to be shortened.
- Assess physical access to services for persons with a range of disabilities; also consider whether adaptive equipment is needed. Assistive devices such as wheelchairs, easy-to-understand language with many pictures, and aids for low vision and hearing impairment must be provided if appropriate.

---

[64] Marshall, P., "Globalization, migration and trafficking: some thoughts from the south-east Asian region", Occasional Paper No. 1, UN Inter-Agency Project on Trafficking in Women and Children in the Mekong Sub-region, paper to the Globalization Workshop in Kuala Lumpur, 8-10 May 2001, United Nations Office for Project Services, September 2001.

Be aware of appropriate referral services

- Be aware of suitable referral options (*see action sheet 10*), because typical post-trafficking service providers may be ill-equipped to meet the specific needs of a person who has been trafficked and who is also living with a disability. Organizations specializing in work with persons with disabilities who have been abused may be able to provide advice.
- When assessing whether a referral service is appropriate for a person with a disability, make sure there are no barriers to access:
  - o Assess possible **physical barriers** for persons with physical and sensory impairments including accommodation suitable to special needs (e.g., ramps); transport and accompaniment (if required); and safety precautions to meet special needs (e.g., emergency call buttons).
  - o Assess possible **communication and information barriers**, including whether information is in an appropriate format (plain text, large print, etc.) and provide assistance in applying for services when necessary.
  - o Assess possible **barriers related to attitudes**. Negative attitudes and stereotypes about persons with disabilities can lead to denial of appropriate health services or poor provision of services.

Be aware of issues around guardianship

- Depending on the individual circumstances of the patient, it may be necessary for a responsible parent, guardian or other adult to be present for consultations and treatment. This is to provide support to the patient and to provide legal consent where necessary (*see action sheet 16*).
- Where possible and safe, the family of the trafficked person should be included and encouraged to play an active role in the recovery and reintegration process. **Special note of caution regarding family and friends acting as representatives or advocates**: Family members may have been instrumental in the sale or exploitation of the patient. Family members may also have been a perpetrator of past abuse, such as physical violence, neglect or sexual abuse. Before involving any family members, friends or acquaintances in the care setting, the health provider must take great care to assess whether the potential guardian or representative has the individual's best

interest in mind. If the potential representative is not suitable, the health provider must make alternative care arrangements, e.g., apply for the appointment of an independent advocate (this may take place with the help of the trafficked person's legal advisor).

REFERENCES AND RESOURCES

Hu, Y.

2004 "1,340 saved from forced labor", *China Daily*, 14 August 2007, p. 3.

International Organization for Migration

2007 *The IOM Handbook on Direct Assistance for Victims of Trafficking*, IOM, Geneva, 2007.

2003 *Trafficking in Persons: An analysis of Afghanistan*, IOM, Kabul, Afghanistan, 2003.

Kropiwnicki, Z. D.

2007 *Children Speak Out: Trafficking risk and resilience in southeast Europe (regional report)*, Save the Children in Albania, Tirana, Albania, July 2007.

Marshall, P.

2001 "Globalization, migration and trafficking: some thoughts from the south-east Asian region", Occasional Paper No. 1, UN Inter-Agency Project on Trafficking in Women and Children in the Mekong Sub-region, paper to the Globalization Workshop in Kuala Lumpur, 8-10 May 2001, United Nations Office for Project Services, September 2001.

Ray, John

2007 "Disabled children sold into slavery as beggars, Chinese racketeers living well by exploiting poverty, ignorance", newspaper article, in « guardian.co.uk » and in *The Observer*, 22 July 2007.

Surtees, R.

2005 "Other forms of trafficking in minors: articulating victim profiles and conceptualizing interventions", paper originally presented at Childhoods Conference, Oslo, Norway, 29 June – 3 July 2005, NEXUS Institute to Combat Human Trafficking and International Organization for Migration.

2005 *Second Annual Report on Victims of Trafficking in South-Eastern Europe*, International Organization for Migration, Geneva, 2005.

Tiurukanova, E. V. and the Institute for Urban Economics

2006 *Human Trafficking in the Russian Federation: Inventory and analysis of the current situation and responses*, United Nations Children's Fund, International Organization for Migration and Canadian International Development Agency, Moscow, March 2006.

United Nations

2006 Convention on the Rights of Persons with Disabilities, adopted on 13 December 2006 by General Assembly Resolution A/RES/61/106, opened for signature 30 March 2007, New York.

United Nations Children's Fund

2004 *Let's Talk: Developing effective communication with child victims of abuse and human trafficking*, UNICEF, Pristina, Kosovo Office, Pristina, September 2004.

Trafficking in children and child involvement in beggary in Saudi Arabia, United Nations Children's Fund Gulf Area Office, Riyadh, Saudi Arabia, undated.

United Nations Educational, Scientific and Cultural Organization

2006 *Human Trafficking in Nigeria: Root causes and recommendations*, Policy Paper No. 14.2(E), UNESCO, Paris, 2006.

United Nations Population Fund

2007 *Sexual and Reproductive Health of Persons with Disabilities: Emerging UNFPA Issues*, UNFPA, New York, USA, 2007.

United States Department of State

2007 *Trafficking in Persons Report, June 2007*, Office of the Under Secretary for Democracy and Global Affairs and Bureau of Public Affairs, Washington, DC, USA, revised June 2007.

West, A.

2003 "At the margins: street children in Asia and the Pacific", Asian Development Bank, Poverty and Social Development Papers, no. 8, October 2003.

World Bank

Disability in Africa Region, web information/portal, available at « *http://web.worldbank.org/WBSITE/EXTERNAL/TOPICS/EXTSOCIALPROTECTION/EXTDISABILITY/0,,contentMDK:20183406~menuPK:417328~pagePK:148956~piPK:216618~theSitePK:282699,00.html*» World Bank, undated (accessed 4 January 2009).

World Health Organization

2001 *International Classification of Functioning, Disability and Health*, WHO, Geneva, 2001.

Zimmerman, C. et al.

2006 *Stolen Smiles: The physical and psychological health consequences of women and adolescents trafficked in Europe*, London School of Hygiene and Tropical Medicine, London, 2006.

Zimmerman, C. et al.

2003 *The Health Risks and Consequences of Trafficking in Women and Adolescents: Findings from a European study*, London School of Hygiene and Tropical Medicine, London, 2003.

Disability, Abuse and Personal Rights Project (resource directory on abuse and disability)
http://disability-abuse.com/

Respond (this is an example of an organization that supports persons who are disabled)
http://www.respond.org.uk

An example of a web resource for combating human trafficking
http://www.humantrafficking.org

An example of an online resource on dignity and rights of persons with disabilities
http://www.un.org/disabilities/default.asp?navid=11&pid=25

Action Sheet 15: Infectious diseases

ACTION SHEET FIFTEEN

Action Sheet 15: Infectious diseases

RATIONALE

Individuals who have been trafficked are at particular risk of exposure to infectious diseases at several stages of the trafficking process.

First, trafficked individuals may be predisposed to infections because of poor health status prior to having been trafficked (this may be associated with poverty, poor access to health care or residence in a country with a high burden of infectious disease). In this situation, risk for infection is similar to poor populations in the community of origin. Therefore, health care providers may need to familiarise themselves with infectious disease patterns in the person's place of origin. Trafficked persons may harbour an infectious disease that has either gone undiagnosed or been treated for an inadequate period. Tuberculosis is a notable example of this; interruption in an individual's treatment regime may predispose them to multi-drug resistant tuberculosis. Unfortunately, the health of someone who has been trafficked tends to worsen during the trafficking situation, especially if the individual cannot access health care because of fear, costs or concerns about stigmatization.

Second, individuals may acquire infectious diseases while in transit from point of origin to the trafficking destination. Particularly for those who travel clandestinely, travel may involve crowded, poorly ventilated, dangerous modes of transport that may be conducive to the spread of disease. Travelling or living in forests, deserts or squalid tenements, wading or swimming through contaminated waters, coming into contact with wild and domesticated animals – all may increase the risk of environmental pathogens and zoonoses (e.g., tick- or mosquito-borne illnesses). Trafficked persons, particularly women and children, may experience sexual abuse or may be raped as part of an initiation and submission process, which may expose them to sexually transmitted infections. Violence-related trauma makes mucosal surfaces of the body more vulnerable to secondary infections and lowers the body's natural ability to fight infection.

Third, people who have been trafficked may be exposed to infectious diseases in destination settings. Women who are trafficked for sexual exploitation may become infected by sexual contact with clients. Trafficked persons may be at risk of sexually transmitted infections from having unprotected sex. In certain settings, HIV transmission is of particular concern. It is therefore good practice to include STI testing in diagnostic procedures for trafficked persons, especially women and girls, regardless of their primary work at the trafficking destination.

Some trafficked persons may be exposed to infections through contact with other trafficked persons or from overcrowded housing and working in substandard conditions. Trafficking puts people at risk for trauma or occupational injuries, which if left unattended can become infected. Poor sanitation, malnutrition, and inability to access health care can increase both the susceptibility to and the severity of infectious diseases prevalent in a destination community.

As with anyone infected in the general population, trafficked persons may transmit these infections to those around them or in the larger community. Therefore, good public health practices require proper attention to prevention, control, and treatment of infectious diseases through a high level of suspicion for infection and transmission among trafficked persons.

This action sheet highlights the potential immediate and long-term problems of infectious diseases in trafficked persons. This information complements the information provided on the comprehensive health assessment in action sheets 4 and 5. Infectious disease concerns that relate to sexual and reproductive health are covered in action sheet 15.

Considerations for children and adolescents (under 18 years of age):

Infectious diseases in children require special consideration, whether they are trafficked themselves or are the children of trafficked persons. In either case, children may not have received standard preventive care or treatment because their caregiver has been absent, in transit, or cannot access or afford care. Children will be as (or possibly more) susceptible to infectious diseases as adults and are likely to have less ability to identify their symptoms, health needs or access care.

Because of their immature immune system, infants are more susceptible to infections than adults. Furthermore, children can present diseases differently from adults. It is therefore important to have a high degree of suspicion for certain infections among trafficked children, particularly those

commonly seen among other trafficked persons in the area (if known). Trafficked children also require special attention because they are likely to have missed routine immunizations for vaccine-preventable diseases; screenings for congenital and acquired health problems; work-ups for infectious diseases; and treatment of community-acquired infections. Health care providers should also be aware of vertical transmission of infectious diseases, particularly HIV, from mother to child; international protocols for prevention of mother-to-child transmission should be followed. (*See action sheet 5 for more comprehensive information on child and adolescent health.*)

Human immunodeficiency virus (HIV)

HIV deserves a special section because of the personal, medical, ethical, social and even political implications of this infection. Those providing health care to trafficked persons – during or after the trafficking situation – must pay special attention to HIV prevention, treatment, care and support, particularly in areas where HIV is prevalent.

Trafficked persons may be at increased risk of HIV infection because of the limited power they may have in negotiating safe sex, including using condoms; because they may be subjected to more repetitive and violent forms of sex, including rape, anal rape and sexual assault; and because many trafficked persons will not have access to information about HIV risk and safe sexual practices. Frequent vaginal or rectal abrasions from multiple forced episodes of intercourse, having multiple sex partners and the presence of other sexually transmitted infections can significantly increase the risk of HIV infection. Pre-pubertal girls and young teens may be particularly vulnerable to infection because of their immature physiology and sexual anatomy. Pregnancy complicates the treatment, care and support of trafficked women who are also HIV-positive (*see action sheet 13*). Non-sexual routes of transmission for HIV include injecting drug use, as well as blood transfusions. The risk of exposure to these non-sexual routes of transmission for individuals who have been trafficked is unknown.

REQUIRED ACTIONS

General

- Ensure that all examinations and testing is voluntary. Ensure all persons are informed of the purpose, procedures, results and implications of tests and that diagnostic testing is voluntary.
- Be aware that infectious diseases can be highly stigmatizing. Discuss such diseases sensitively: failure to do so may

compound the loss of confidence and self-esteem often experienced by people who have been trafficked. Use positive and accurate messages about prevention and treatment whenever possible.

- Be sure that the patient is adequately informed about potential communicability. Provide information on preventing transmission to others.
- Be informed about local prevalence rates for infectious diseases. Also be aware of infections endemic to the trafficked person's place of origin: this allows for a more informed examination and diagnosis.
- Follow protocols regarding paediatric diagnosis and treatment of infectious diseases, particularly HIV and tuberculosis in children. Children and adolescents often present diseases differently from adults. They may require special treatment protocols based on age, physical development and size.
- Compliance with confidentiality protocols is essential. Trauma-informed care, patient-centred care and informed consent are covered in action sheets 1 and 2.
- Identify resources for infectious disease diagnosis and investigation that may not be possible at your facility. Identify in advance options for patient referral, if necessary.
- Communicate accurate messages in positive ways. For example: "TB is treatable and curable". Be aware of drug-resistant TB, which requires longer duration of treatment with more expensive drugs that have greater side effects.
- Inform women of child-bearing age of the risks infectious diseases pose to maternal-child health.
- Be aware of local terms for sexually transmitted infections and their symptoms that may be used by or more easily understood by patients.

History-taking

- Cover key points including immunization status, travel history, prior infectious diseases and extent of treatment.
- In the case of children, where possible obtain information on birth and development facts, which may indicate infectious disease exposure. Also seek information on any clinical encounters for routine immunizations, health screenings and tuberculosis skin testing.

Physical exam

Consider specific diseases by system:

- Dermatological: herpes simplex, lice (body, hair or pubic), scabies, ringworm affecting body or scalp, chicken pox, buboes (plague), haemorrhagic fever, Hansen's disease, cellulitis; impetigo; molluscum contagiosum; soft-tissue injuries (including human bites) that are secondarily infected.
- Pulmonary: pneumonia, TB, other mycobacterial infections, viral respiratory syndromes; plague.
- Cardiovascular: viral cardiomyopathy, bacterial endocarditis (particularly with injecting drug use).
- Blood: HIV, hepatitis B, hepatitis C, dengue, malaria.
- Gastrointestinal: diarrhoeal disease, parasitism.
- Genito-urinary: gonorrhoea (local and disseminated), herpes simplex, syphilis, human papillomavirus (HPV), chlamydia, trichomonas, chancroid, condylomata. Candida and other yeast infections, bacterial vaginosis, endocervicitis and urinary tract infections are not sexually transmitted infections but can have adverse outcomes and complicate the clinical picture.
- Eye: conjunctivitis.
- Neurological: meningitis (including tuberculous meningitis), tetanus, encephalitis, brain abscesses.

Laboratory

Laboratory screening for infectious diseases requires a level of suspicion based on the patient's history and examination. In resource-poor settings, screening and testing should be offered depending on the availability of reliable diagnostic laboratory facilities. The patient should be adequately informed about the nature of the tests and the implications for treatment based on the results.

- Guided by the history and physical exam, baseline tests might include:
  - complete blood count with differential
  - HIV testing (rapid tests) following voluntary- or provider-initiated counselling
  - Serologic testing for hepatitis B, hepatitis C, syphilis (VDRL or RPR)
  - chest X-ray
  - stool ova and parasites
  - TB skin test (Mantoux/PPD)

  - PAP smear (human papillomavirus)
  - Cervical / vaginal swab (chlamydia, trichomonas)
  - Pharyngeal, high vaginal and rectal swabs (gonorrhoea)
- If the patient comes from or has transited through an endemic malarial zone, perform thick and thin blood smears.
- If TB symptoms are present, collect sputum specimens in accordance with national TB program guidelines
- Consider the need for skin or scalp scrapings, looking for skin and hair infestations of scabies or lice, infections with fungi or herpes, or needle aspirations for cellulitis or staphylococcal infections.
- If you suspect genitourinary infections, do a urinalysis including leukocyte esterase, and possibly a urine culture;
- If the patient has been raped, conduct other tests using a rape test kit, according to proper forensic exam technique.

HIV

- Offer voluntary and confidential HIV pre- and post-test counselling, with emotional support. If this is not available at your facility, identify where authorized HIV testing and counselling is available in the community so that you can provide access.
- Know ahead of time whether confidential treatment, including with antiretrovirals, is available. If antiretroviral medications are available, link with knowledgeable health care professionals to assure treatment adherence and an uninterrupted supply of drugs.
- In the case of pregnancy, follow international protocols to reduce likelihood of vertical transmission of HIV from mother to child. HIV-positive women should be fully informed about the risk of transmission to the foetus or the newborn and how to reduce this risk.
- Where labs are available and reliable, CD4 counts and viral loads, or at least total lymphocyte counts, will indicate the degree of immuno-suppression.
- If a patient is diagnosed with HIV or another sexually transmitted infection, consider the possibility of other sexually transmitted infections because of the high likelihood of concurrent infections. Treatment of one sexually transmitted infection will enhance the treatment of another.
- Discuss myths and truths about HIV infection, prevention, and treatment, because many trafficked people may not have much information – or may have incorrect information – about HIV.

- Provide information on correct and consistent condom use, recognizing that condoms are not 100 per cent protective and that the patient may not be able to insist on condom use. Where possible, provide condoms.
- Consider post-exposure prophylaxis (PEP) for those who present within 72 hours of a high-risk exposure.
- Look for opportunistic infections in anyone with poorly treated or untreated HIV, or in patients who fail to respond to normal therapeutic regimens. Serious and recurrent infections are also indicators of immuno-suppression.

Keep in mind that HIV-positive persons will have particular needs for ongoing care and support. Knowing your community resources is invaluable for patient referral.

Treatment plan

- Immunize patients of all ages according to the expanded protocol for immunizations in the country.
- Provide immediate anti-bacterial, anti-viral, anti-parasitic, anti-malarial or anti-fungal treatment, when indicated.
- If STI testing is not available, the syndromic approach to treating STI is adequate.
- Assure treatment adherence to anti-TB drugs. Involve public health authorities knowledgeable about DOTS and who can provide ongoing instruction and guarantee an uninterrupted supply of drugs.
- Nutritional rehabilitation is vital to the treatment of infectious diseases. Appropriate nutrition should be provided, including correction of vitamin and mineral deficiencies.
- Consider prophylaxis for patients exposed to diseases where there is potential for prevention. For example, post-exposure prophylaxis for HIV; hepatitis B immunoglobulin (HBIG) for hepatitis B; and tetanus toxoid for tetanus.
- Teach patients about personal hygiene and offer soap, hand sanitizers and similar items. Be aware that basic knowledge regarding hygiene and transmission of disease may not be well understood.
- Persons who have recently left a trafficking situation may still be vulnerable to some infectious diseases as a result of the physical conditions in which they are living (e.g., if they are living in temporary housing, in camps or in detention). You are responsible for treating those diseases to which the patient

may be vulnerable, and for providing information about prevention, risk reduction and mitigation of the negative health impact of living in such circumstances.

Public health

- Practice universal precautions at all times to protect staff and self.
- Establish plans for infection control and possible quarantine, which may include awareness of local laws and a relationship with the local health authorities and infectious disease specialists.

Trafficked persons should not be viewed as 'vectors of disease'. Individuals are often trafficked into circumstances that may make them more vulnerable to contracting infections and less able to access treatment. Health care providers are responsible for offering patient-centred approaches to diagnosis and treatment to protect the health of individuals and public health.

REFERENCES AND RESOURCES

Grant, B. and C. L. Hudlin (Eds.)
2007 *Faith Alliance Hands that Heal: International curriculum to train caregivers of trafficking survivors*, Faith Alliance Against Slavery and Trafficking, Alexandria, Virginia, USA, 12 September 2007.

Heymann, D. L. (Ed.)
2004 *Control of Communicable Diseases Manual, 19th Edition*, American Public Health Association Press, Washington, DC, USA, December 2008.

International Organization for Migration
2007 *The IOM Handbook on Direct Assistance for Victims of Trafficking*, IOM, Geneva, 2007.

Pickering, L.K. (Ed.)
2006 *The Red Book: 2006 Report of the Committee on Infectious Diseases, 27th Edition*, American Academy of Pediatrics, Oak Grove, Illinois, USA, 2006.

Silverman, J. et al.

2007 "HIV prevalence and prediction of infection in sex-trafficked Nepalese girls and women", *The Journal of the American Medical Association*, vol. 298, no. 5, pp. 536–542.

United States Centers for Disease Control and Prevention, et al.

2008 *Guidelines for Prevention and Treatment of Opportunistic Infections among HIV-Exposed and HIV-Infected Children*, Centers for Disease Control and Prevention, National Institutes of Health, Infectious Disease Association of America, Pediatric Infectious Disease Society and American Academy of Pediatrics, USA, 20 June 2008.

World Health Organization

2001 *Recommended Strategies for Prevention and Control of Communicable Diseases*, WHO, Geneva, 2001.

2006 "Sexually Transmitted Diseases Treatment Guidelines, 2006", *Morbidity and Mortality Weekly Report*, vol. 55, no. RR-1, 4 August 2006.

World Health Organization and International Labour Organization

2007 *Post-Exposure Prophylaxis to Prevent HIV Infection: Joint WHO/ILO guidelines on post-exposure prophylaxis (PEP) to prevent HIV infection*, WHO, Geneva, 2007.

Action Sheet 16: Medico-legal considerations

ACTION SHEET SIXTEEN

Action Sheet 16: Medico-legal considerations

RATIONALE

Trafficking in human beings is a criminal activity and therefore health assistance to trafficked persons is very often linked to legal rights, obligations and judicial procedures. Within the clinical setting, there are medico-legal obligations for the provider to adhere to and rights of the patient that must be communicated and observed (*see action sheet 17 for more on coordination with law enforcement*).

All medical staff involved in the care of trafficked persons must be careful to learn about their obligations and limitations according to local legislation. Sometimes legal issues may be complex, for example, regarding mandatory reporting requirements or care provision in special circumstances. For instance, trafficked persons requiring care may be in detention centres or prison settings. Legislative and regulatory frameworks may help or hinder health services or may dictate how they must be carried out, recorded or reported. It is therefore very important for providers to be well-informed – or at the very least – know where to seek information for themselves and for their patients. It is also essential to know where to refer your patient for legal counselling (*see action sheet 10*).

REQUIRED ACTIONS

Health workers and forensic evidence associated with trafficking

Trafficked persons should be informed of their right to a forensic medical examination where local legal requirements permit forensic evidence and capable laboratory facilities are available. Forensic exams should be offered upon the first contact with a health provider because timing is essential for gathering medical evidence. A forensic medical examination is an examination provided to a victim of a crime, carried out by a forensic

professional trained to gather evidence in a manner suitable for use in a court of law (*see actions sheets 4, 5 and 13*).

- Recruit or refer your patient to an external forensic expert to collect medical evidence (with the consent of the trafficked person), if you do not wish to present testimony or appear as an expert witness in court, or if you do not have the necessary training in forensic medicine. It is important to have contact details of recognised forensic professionals (*see action sheet 10*).
- Make sure your patient is informed (by you or by a legal professional) how medical records or information may be used in court. In some circumstances, services provider staff can be ordered to provide such evidence to official investigators working for the prosecution or defence of the alleged trafficker, and patients should be aware of this (*see action sheet 9*).
- Keep all evidence obtained from medical records (e.g., medical history, examination notes, diagnostic results and treatment records) confidential and only provide evidence to law enforcement authorities with the permission of the trafficked person or at the direction of a court (*see action sheet 9*). Ensure you are aware of any local laws that dictate who has and who does not have a right to access these files.
- Ensure that health workers assisting trafficked persons have basic knowledge and training in forensic medicine, so that if they see the individual before a forensic specialist, they understand basic preliminary procedures. For example, staff should be aware of the importance of clothing as evidence and of noting carefully details from the victim's first reporting of past events and symptoms.

The right of trafficked persons to their health records

- Inform patients of their right to copies of all their medical records. Individuals have a right to a copy of a forensic medical examination in order to pursue criminal or civil legal claims against a trafficker or others who have abused or exploited them.
- Use available translating resources to ensure the individual has a copy of a summary medical report in her or her original language.

Informed consent

Obtaining informed consent is particularly important for patient-provider procedures that may become part of a legal case. The individual's consent must be given based upon her or his clear appreciation and understanding of the facts and implications of health care services and procedures. The individual needs to be in possession of relevant facts and reasoning faculties. It is not reasonable to gain informed consent from someone who may have impaired judgement at the time consent is requested. Such impairments might include illness, intoxication, insufficient sleep, pain or other health problems.

When a person who has been trafficked is deemed unable to give informed consent, another person can be authorized to give consent on their behalf. Examples of this include the parents or legal guardians of a child or a state child welfare officer, or caregivers for the severely mentally ill. In cases where an individual is provided with limited facts or has a limited understanding of information provided to them, serious ethical and legal issues may arise.

- Obtain informed consent in writing, through a professional interpreter or guardian if needed, before undertaking any medical procedures. Ensure the patient understands that he or she may decline or accept some or all of the proposed procedures. Potential implications for the refusal of treatment should be explained as well. Information needs to be provided in a neutral, non-judgemental, and non-coercive way. If the person refuses to sign but gives verbal authorization, the provider should document that verbal consent was provided.

Consent may be overridden in certain circumstances. Be aware of local regulations and laws as these circumstances differ by jurisdiction and depending on local legislation. Examples might include:

- Notifying authorities of suspected child abuse or domestic violence, in the absence of consent of the victim.
- Enforcing treatment in those who are psychiatrically ill and who have been involuntarily hospitalized.
- A court ordering examination of a patient records, generally as part of the investigation into criminal offences. Here the patient may either be a 'victim' or a suspect.
- Life-saving procedures

Competence, capacity, and guardianship[65]

There is a tendency to use the terms 'capacity' and 'competence' interchangeably though they are not the same. *Capacity* refers specifically to the presence of mental abilities to make decisions or engage in a course of action while *competence* refers to the legal consequences of not having the mental capacity. Thus capacity is a health concept whereas competence is a legal concept. When persons are unable to make important decisions about their own health care, most legislative systems allow someone to be appointed to act on their behalf as a legal *guardian*. Such decisions should always be made within the context of the rights of persons to have as much control over their own lives as possible.

Capacity

Assessing a patient's capacity to make a specific decision means evaluating the following: 1) whether the person has a general understanding of the decision they need to make and why they need to make it; 2) whether the person has a general understanding of the likely consequences of making – or not making – this decision; 3) whether the person is able to understand, retain, use and evaluate the information relevant to this decision over a period of at least a few minutes; and 4) whether the person can communicate his or her decision. In assessing capacity, consider the following:

- Assess the patient's mental capacity to make a decision if you suspect that capacity may be lacking and if you are considering making an intervention in someone's 'best interest'.
- Assume that an adult (usually aged 18 or over though the legal definition of adult varies by country) has full capacity to make decisions for his or herself (the right to autonomy), unless it is shown that the person lacks capacity to make their own decision at the time the decision needs to be made. A person may lack mental capacity (i.e., the ability to make a decision) if they have an impairment or disturbance of the mind or brain and this impairment or disturbance means that the person is unable to make the decision in question at the time it needs to be made. Disability does not imply in and of itself a lack of capacity (see action sheet 14). A lack of capacity could be the result of a severe learning disability, severe mental health problems, a brain injury or impaired consciousness due to alcohol or drug misuse or to such causes as delirium.

---

[65] *WHO Resource Book on Mental Health, Human Rights, and Legislation*, WHO, Geneva, 2005, pages 39–43.

- In some emergency situations, treatment cannot be delayed while a person gets support to make a decision. For non-emergency situations, ensure that the person is supported to make his or her own decision as far it is possible to do so. A few examples of such support include: making sure that the patient is in an environment in which he or she is comfortable; using a different form of communication, e.g., non-verbal communication; providing information in a more accessible form, e.g., drawings; or treating a medical condition which may be affecting the person's capacity.
- Do not determine that someone lacks capacity to make a decision simply because they make an unwise decision.
- Ensure that the patient has all the information needed to make a particular decision, including information about possible alternatives.
- Encourage individuals to ask questions, request information be repeated and gain clarification about issues that seem complicated.
- Be aware of cultural, ethnic or religious factors that shape a person's way of thinking, behaviour or communication.

If your assessment suggests that the person lacks the capacity to comprehend and process the information provided, you may wish to seek a specialist opinion. If you wish to act or make a decision on behalf of the person you believe lacks the capacity you should ensure this is in the person's best interests and in accordance with national legislation. Any act done for, or any decision made on behalf of, someone who lacks capacity should be an option that promotes and protects their basic rights and freedoms and is in their best interests (*see box*).

Legal guardians representing a child or adolescent (under 18 years of age)

Children and adolescents have a right to health services appropriate to their age. Age-appropriate care is essential for their survival and well-being and is a fundamental human right (*see action sheets 5 and 12*). The United Nations *Convention on the Rights of the Child* states that the best interest of the child shall be a primary consideration. Requirements around guardianship and consent for minors are based on national legislation. Providers must consult relevant local authorities to arrange for appropriate legal guardianship and consent for an unaccompanied minor (*see box*).

In some cases, a child's legal guardian may be an abusive parent or relative, or the individual that sold the child to the trafficker. When there is reason to believe a child was or will be abused by parents of relatives, it is crucial to seek legal advice and have national authorities assign a legal guardian who can make decisions on behalf of the child and act in his or her best interest.

- Obtain appropriate consent from a legal guardian for any medical examination or procedures, except in cases where the child's life is in immediate danger.
- Do not assume automatic guardianship as a health provider and make decisions on behalf of a child, even if you think this is in the best interest of the child.
- Lack of genuine identity documents can be an obstacle to health care and emergency identification papers may have to be obtained.

There are many complex ethical and administrative issues related to laws governing health and health care. Different countries will have differing legislation and regulations. These laws may also change over time. Because of the diversity of individuals who are trafficked (including children, undocumented migrants, persons with disabilities, and persons who may not speak the local language) it is imperative for medical professionals to consult national resources including legal experts to learn about their professional obligations. It is also important that health providers know where to refer their patients when they are in need of specialized legal support (*see action sheet 10*).

REFERENCES AND RESOURCES

Hossain, M. et al.
2005 *Recommendations for Reproductive and Sexual Health Care of Trafficked Women in Ukraine: Focus on STI/RTI care, First Edition*, London School of Hygiene & Tropical Medicine and the International Organization for Migration, 2005.

International Organization for Migration
2007 *The IOM Handbook on Direct Assistance for Victims of Trafficking*, IOM, Geneva, 2007.

Physicians for Human Rights
2001 *Examining Asylum Seekers: A health professional's guide to medical and psychological evaluation of torture*, PHR, Cambridge, Massachusetts, USA, August 2001.

United Nations Children's Fund

2003 *Guidelines for the Protection of the Rights of Children Victims of Trafficking in South Eastern Europe*. Unpublished draft.

2006 *Action to Prevent Child Trafficking in South Eastern Europe*, preliminary assessment, UNICEF and Terre des homes Foundation, Geneva, June 2006.

United Nations Office of the High Commissioner for Human Rights

2002 *Recommended Principles and Guidelines on Human Rights and Human Trafficking*, Report of the United Nations High Commissioner for Human Rights to the Economic and Social Council (E/2002/68/Add.1), United Nations Economic and Social Council, New York, 20 May 2002.

1989 Convention on the Rights of the Child, Adopted and Open for ratification by General Assembly Resolution 44/25 of 20 November 1989, entry into force 20 September 1990. See in particular Articles 3 and 24.

World Health Organization

2005 *WHO Resource Book on Mental Health, Human Rights, and Legislation*, WHO, Geneva, 2005.

2002 *Guidelines for medico-legal care for victims of sexual violence*, WHO, Geneva, 2003.

1999 *Ethical Practice in Laboratory Medicine and Forensic Pathology*, WHO Regional Publications, Eastern Mediterranean Series, no. 20, WHO Regional Office for the Eastern Mediterranean, 1999.

World Health Organization and United Nations High Commissioner for Refugees

2004 *Clinical Management of Rape Survivors: Developing protocols for use with refugees and internally displaced persons, revised edition*, WHO and UNHCR, Geneva, 2004.

Action Sheet 17: Interactions with law enforcement

Action Sheet 17: Interactions with law enforcement

RATIONALE

Human trafficking involves various criminal acts that may include assault, abduction, blackmail, extortion, false imprisonment, immigration crimes, pimping, rape and rape of a minor. A trafficked person may come into contact with the criminal justice system as a victim or witness of a crime. Unfortunately, some victims are also mistakenly identified and prosecuted for such criminal violations as illegal immigration, fraud or prostitution. It may be necessary to seek support from local human rights or legal aid organizations to help defend such persons (*see action sheet 10*).

At some point while providing care for trafficked persons, health care practitioners may come into contact with law enforcement staff such as police or immigration officials. This contact may happen directly or indirectly:

- Trafficked persons are referred to health care following a police raid or an immigration procedure.
- A health provider is treating persons who are participating in a criminal prosecution.
- A health provider is asked to provide medical reports or expert testimony for police investigations, trials or asylum applications.
- A health provider is providing psychological support when a patient who has been trafficked participates in a criminal investigation or trial.

Health providers may also be in contact with immigration or policing services under less positive, more 'hostile' circumstances, for example:

- A health provider treating trafficked persons who are held in such confined facilities as immigration detention centres, prisons or under the watch of law enforcement officials.
- A health provider treating individuals who may be the target

of an investigation into trafficking (e.g., the patients is suspected of having collaborated in trafficking cases).
- A health provider treating individuals who are living covertly (e.g., avoiding deportation, maintaining irregular or illegal employment).
- A health provider interacting with police or immigration officials who are corrupt or who have an agenda that is not in the trafficked person's best interest.

Many of these interactions may be stressful to trafficked persons and to health care providers. Some may pose challenging dilemmas about legal requirements and protection obligations.

REQUIRED ACTIONS

You must be absolutely clear about your role and ethical obligations as a health provider in cases where victims of trafficking are in contact with law enforcement. Rules about medical ethics and, in particular, patient safety and confidentiality are paramount (*see chapter 3*). Adhering to these principles can be challenging when they appear to be in conflict with what is wanted or needed by law enforcement or immigration officials.

Maintain confidentiality

You may sometimes feel pressure from – or, in some cases, camaraderie with – authorities. When put in the position between the obligations to the patient and requests from officials who may need information for an investigation or an immigration case, the medical ethics code of conduct requires that the practitioner respect the confidentiality and decisions of the patient. Patient communications and files are private and cannot be transferred to authorities without the express permission of the patient or a judicial order (*see action sheet 9*).

Similarly, even when you are attempting to 'protect' trafficked persons, do not pressure them to withhold or release evidence or to avoid or collaborate with investigators. Explain options clearly and neutrally to your patients, and let them make their choices freely.

Express objectivity and compassion

Behave impartially and compassionately when you are in a position of being an intermediary between a trafficked person and a government authority. It is not uncommon for those providing health services to trafficked

persons to have a professional or funding relationship (e.g., protection by police, access to detention centres or funding from social services) with officials seeking information about the trafficked person. These relationships should not affect your professionalism and objectivity, particularly when drafting written reports or offering testimony about a trafficked person.

Do not express a 'legal opinion', provide referral to legal counsel

Health care providers should not try to offer legal advice to persons who have been trafficked. Providers should be able to facilitate a referral to legal professionals who are able to provide accurate advice and assistance (*see action sheet 10*). It is important to make this referral because in most settings, trafficked persons have the right, for example, to participate or refuse to participate in a prosecution as witness against the trafficker(s) and in many locations, they may also seek compensation for the crimes committed against them.

Do not offer personal involvement or assistance

Trafficked persons very often seem alone and sometimes in extreme danger and in need of urgent care. You may feel the need to respond by offering such personal assistance as shelter or personal contact details. This type of non-professional support may put you in danger, create expectations that cannot be met and foster an unsustainable and ultimately destructive relationship. Instead, you should make every effort to make safe and well-supported referrals and seek peer group support when available (*see action sheets 8 and 10*).

Consult with trafficked persons before reporting a crime or seeking law enforcement assistance or protection on their behalf

If you suspect or are informed that an individual in your care has been trafficked, you should **not** contact the police or other authorities (e.g., consular staff) without the express permission of the trafficked person.[66] Although you may be tempted to seek help or protection for individuals in your care by contacting authorities, this decision is one that should not be taken without consulting your patient. This is particularly important because, in some locations, officials may be corrupt or in collusion with traffickers or others who are exploiting the individual. Some individuals may assess that it is not safe for them or family members to leave the trafficking situation at that time. Given adequate information and the opportunity to discuss their options, trafficked persons are generally in the best position to assess the

---

[66] Please see action sheet 16 for special considerations related to competence, capacity, and guardianship.

risks and benefits of contact with authorities. Similarly, reporting information offered by trafficked persons to law enforcement should take place only with the consent of the trafficked person (*see action sheet 6*).

Offer a fair, well-considered, assessment of a patient's ability to participate in a criminal or judicial proceeding

There are times when health care providers, particularly psychologists and psychiatrists, will be given the opportunity to offer their judgement about the physical or psychological capacity of a trafficked person to participate in such legal proceedings as an interrogation, court testimony or immigration appeals proceeding. It is particularly important for specialists in mental health to highlight to those involved in legal proceedings the ways that post-trafficking reactions may impair memory, recall and cognition. In their role as a medical professional, practitioners may assess the ability of the individual to participate and the potential harm that might be caused as a result of the level of participation requested. Whenever possible, providers should be available to support the individual's psychological reactions to these proceedings.

Prepare a forensic report

Be aware of the value of forensic evidence. Forensic reports can play an important role in criminal investigations and asylum claims. Local legal requirements and laboratory capabilities determine if and what evidence should be collected for use in criminal prosecution, and by whom. If available, it is best to have forensic evidence collected by specially trained forensic professionals. Health workers should not collect evidence that cannot be processed or that will not be used. Before a forensic exam can be carried out, it is essential to gain an individual's informed consent; explain the purpose of the exam to the patient before undertaking any exam. Exams should be conducted with great sensitivity to ensure that the individual is not further traumatized and is treated as an individual who may have experienced painful or life-threatening events.

Raise awareness about the health implications of trafficking and post-trafficking symptoms

If you become familiar with the health risks and characteristics of trafficked persons, post-trafficking reactions, or the effects of severe or chronic trauma, try to create links with other services and agencies working on trafficking. The health consequences of trafficking and related care needs are relatively overlooked or ignored aspects of human trafficking – even

though the health impacts of trafficking can have a significant influence on prevention, prosecution and protection activities.

Trying to care for a patient when there are legal proceedings involved, demands from law enforcement or pressures related to the patient's immigration status can be complicated –sometimes even stressful or frustrating. It can be helpful for providers in these situations to gain legal counsel about their rights and obligations, as well as those of their patient.

REFERENCES AND RESOURCES

International Centre for Migration Policy Development

2006 *Anti-trafficking training material for judges and prosecutors in EU member states and accession and candidate countries (handbook).* Vienna: ICMPD, 2006.

International Organization for Migration

2007 *The IOM Handbook on Direct Assistance for Victims of Trafficking*, IOM, Geneva, 2007.

2008 *Handbook on Performance Indicators for Counter-Trafficking Projects: A handbook for project managers, developers, implementers, evaluators and donors working to counter trafficking in persons*, IOM, Geneva, 2008.

OSCE Office for Democratic Institutions and Human Rights

2004 *National Referral Mechanisms Joining Efforts to Protect the Rights of Trafficked Persons: A practical handbook*, Organization for Security and Co-operation in Europe, Warsaw, 2004.

United Nations Office on Drugs and Crime

2008 *Toolkit to Combat Trafficking in Persons*, UNODC Global Programme against Trafficking in Human Beings, Vienna, 2008.

Conclusion

Conclusion

People who are trafficked are daughters and sons, mothers, brothers, fathers and sisters. Most often, they are individuals who believed they were being given an opportunity to earn money to improve their future and that of their loved ones. Once in a trafficking situation, most come to believe that their hopes have been ruined. For many survivors, consequent health problems may further hamper their ability to care for themselves and their family. For those most severely abused, those violated at the youngest ages or those most vulnerable to mental health problems, the psychological burden may prevent them from moving beyond the trafficking experience, and may even make them at risk of re-trafficking or other forms of abuse.

This handbook is a ‘guide’, not a prescriptive text, for health care providers to consider ways in which they might assist trafficked and other exploited individuals to regain their health, independence and hope for a better future. There are numerous guidance materials on responding to other forms of abuse (e.g., domestic violence, sexual abuse, or child abuse) or caring for vulnerable populations (e.g., minors, migrants, or disabled persons) that have been developed over the past decade. Many of these have been cited in this text. We urge you to consider these essential supplements to this handbook and to consult these resources as needed.

As noted early in this handbook, there is currently very little evidence on the health of trafficked persons. Health has been a neglected area of study. We are in urgent need of a greater knowledge-base on the health needs of individuals who are exploited in different sectors and in various ways. We are in even greater need of evidence on the best ways to return health and well-being to those who have been harmed by traffickers, who unscrupulously take advantage of people’s hopes and determination for a better life. As this evidence emerges, we intend to revise this guidance based on what has been learned by the health community of practitioners.

Finally, we want to emphasise that, despite the obvious health implications of being trafficked, the health care community is very frequently left out of the dialogue, planning and resource allocation for trafficked persons. We wish to urge greater participation. Especially for those of you who are seeing numbers of trafficked persons or persons you suspect may be have been trafficked, it is important to find ways to participate in both policy discussions and local service networks.

While some trafficked persons may simply need a medical assessment and reassurance that they are healthy, others may require intensive or long-term support. In either case, as health care providers, we must all continue to remind governments and others planning assistance for trafficked persons that health care is an essential component of any support package and we must be prepared to offer the best possible treatment for persons who are trafficked. We hope you find this guidance document useful in caring for trafficked persons.

BIBLIOGRAPHY

Bibliography

Adams, R. E. et al.
2006 "Compassion fatigue and psychological distress among social workers: a validation study", *American Journal of Orthopsychiatry*, vol. 76, no. 1, January 2006, pp. 103–108.

Anderson, B. and B. Rogaly
2005 *Forced Labour and Migration to the UK*, Oxford: Centre for Migration, Policy and Society (COMPAS), in association with the Trades Union Congress, TUC, London, 2005.

Anti-Slavery International
2006 *Trafficking in Women, Forced Labour and Domestic Work in the Context of the Middle East and Gulf*, working paper, Anti-Slavery International, London, 2006.

Anti Slavery International and International Confederation of Free Trade Unions (ICTFU)
2001 *Forced Labour in the 21st Century*, Anti-Slavery International, London, 2001.

Basoğlu, M. and S. Mineka
1992 "The role of uncontrollable and unpredictable stress in post-traumatic stress responses in torture survivors" in *Torture and Its Consequences: Current Treatment Approaches*, M. Basoğlu, Ed., Cambridge University Press, New York, 1992.

Beyrer, C.
2004 "Is trafficking a health issue?" *The Lancet,* vol. 363, no. 9408, 14 February 2004, p. 564.

Brach, C. and I. Fraser
2000 "Can cultural competency reduce racial and ethnic disparities? A review and conceptual model", *Medical Care Research and Review*, vol. 57, no. 4 suppl., December 2000, pp. 181–217.

Canadian Centre for Occupational Health and Safety
2008 "Extreme hot or cold temperature conditions", web information, available at « http://www.ccohs.ca/oshanswers/phys_agents/hot_cold.html » CCOHS, Hamilton, Ontario, Canada, page last updated 20 October 2008 (accessed 2 January 2009).

Carrillo, J. E. et al.
1999 "Cross-cultural primary care: a patient-based approach", *Annals of Internal Medicine*, vol. 130, no. 10, 18 May 1999, pp. 829–834.

Chang, J. C. et al.
2005 "Asking about intimate partner violence: advice from female survivors to health care providers", *Patient Education and Counseling*, vol. 59, no. 2, November 2005, pp. 141–147.

Clark, H. and A. Power
2005 "Women, co-occurring disorders, and violence study: a case for trauma-informed care", *Journal of Substance Abuse Treatment*, vol. 28, no. 2, March 2005, pp. 145–146.

Council of Europe
2005 Council of Europe Convention on Action Against Trafficking in Human Beings, treaty open for signature by the member states, the non-member states which have participated in its elaboration, and by the European Community, and for accession by other non-member states, CETS no. 197, Warsaw, Poland, 16 May 2005.

Culhane-Pera, K.A. et al.
1997 "A curriculum for multicultural education in family medicine", *Family Medicine*, vol. 29, no. 10, November-December 1997, pp. 719–723.

Davis, D.A. et al.
1995 "Changing physician performance: a systematic review of the effect of continuing medical education strategies", *Journal of the American Medical Association*, vol. 274, no. 9, 6 September 1995, pp. 700–705.

Day, J.H. et al.
2006 *Risking Connection in Faith Communities: A training curriculum for faith leaders supporting trauma survivors*, Sidran Institute Press, Baltimore, Maryland, USA, 2006.

Delaney, S. and C. Coterrill
2005 *The Psychosocial Rehabilitation of Children who have been Commercially Sexually Exploited: A training guide*, ECPAT International, Bangkok, Thailand, 2005.

Denoba, D. L. et al.
1998 "Reducing health disparities through cultural competence", *American Journal of Health Education*, vol. 29 (5 Suppl.), pp. S47–S58.

European Parliament and the Council of the European Union
1995 "Directive 95/46/EC of the European Parliament and of the Council of 24 October 1995, on the protection of individuals with regard to the processing of personal data and on the free movement of such data", *Official Journal of the European Communities*, no. L 231/81, 23 November 1995.

Elliott, D. et al.
2005 "Trauma-informed or trauma-denied: principles and implementation of trauma-informed services for women ", *Journal of Community Psychology*, vol. 33, no. 4 (special issue on 'Serving the needs of women with co-occurring disorders and a history of trauma'), July 2005, pp. 461–477.

EuroSOCAP Project
2005 European Standards on Confidentiality and Privacy in Health Care, EuroSOCAP and Queen's University, Belfast, November 2005.

Figley, C.R. (Ed.)
2002 *Treating Compassion Fatigue*, part of Psychological Stress Series, Brunner-Routledge Press, New York, NY, USA, 2002.

Grant, B. and C. L. Hudlin (Eds.)
2007 *Faith Alliance Hands that Heal: International curriculum to train caregivers of trafficking survivors*, Faith Alliance Against Slavery and Trafficking, Alexandria, Virginia, USA, 12 September 2007.

Harris, M. and R.D. Fallot

2001 "Envisioning a trauma-informed service system: a vital paradigm shift", *New Directions for Mental Health Services,* vol. 89, Spring 2001, pp. 3–22.

Heymann, D. L. (Ed.)

2004 *Control of Communicable Diseases Manual, 19t[h] Edition*, American Public Health Association Press, Washington, DC, USA, December 2008.

Hjermov, B.

2004 *Cultural Mediation at the Workplace – an Introduction*, 2004.

Hossain, M. et al.

2005 *Recommendations for Reproductive and Sexual Health Care of Trafficked Women in Ukraine: Focus on STI/RTI care, First Edition*, London School of Hygiene & Tropical Medicine and the International Organization for Migration, 2005.

Hu, Y.

2007 "1,340 saved from forced labor", *China Daily*, 14 August 2007, p. 3.

Human Rights Watch

2006 "Swept under the rug: abuses against domestic workers around the world", *Human Rights Watch*, vol. 18, no. 7(C), Spring 2006, pp. 1–95.

Huntington, N. et al.

2005 "Developing and implementing a comprehensive approach to serving women with co-occurring disorders and histories of trauma", *Journal of Community Psychology,* vol. 33, no. 4 (special issue on 'Serving the needs of women with co-occurring disorders and a history of trauma'), July 2005, pp. 395–410.

Inter-Agency Standing Committee

2007 *IASC Guidelines on Mental Health and Psychosocial Support in Emergency Settings*, IASC, Geneva, 2007.

2005 *Guidelines for Gender-Based Violence Interventions in Humanitarian Settings: Focusing on prevention of and response to sexual violence in emergencies*, IASC, Geneva, September 2005.

International Centre for Migration Policy Development

2006 *Anti-trafficking training material for judges and prosecutors in EU member states and accession and candidate countries (handbook).* Vienna: ICMPD, 2006.

International Labour Organization

2005 *A Global Alliance Against Forced Labour: Global report under the follow-up to the ILO Declaration on Fundamental Principles and Rights at Work,* International Labour Conference, 93rd Session 2005, Report I (B), International Labour Office, Geneva, 2005.

International Organization for Migration

2008 Data Protection Principles: Information Bulletin IB/00047

2008 *Handbook on Performance Indicators for Counter-Trafficking Projects: A handbook for project managers, developers, implementers, evaluators and donors working to counter trafficking in persons*, IOM, Geneva, 2008.

2007 *The IOM Handbook on Direct Assistance for Victims of Trafficking*, IOM, Geneva, 2007.

2006 *Breaking the Cycle of Vulnerability: Responding to the health needs of trafficked women in east and southern africa*, IOM, Pretoria, South Africa, September 2006.

2005 *IOM Counter-Trafficking Training Modules: Return and reintegration*, IOM, Geneva, 2005.

2004 *The Mental Health Aspects of Trafficking in Human Beings: Training manual*, IOM, Budapest, Hungary, 2004.

2004 *The Mental Health Aspects of Trafficking in Human Beings: A set of minimum standards*, IOM, Budapest, Hungary, 2004.

2003 *Trafficking in Persons: An analysis of Afghanistan*, IOM, Kabul, Afghanistan, 2003.

2001 *Medical Manual, 2001 Edition*, IOM, Geneva, 2001.

Joos, S. K. et al.

1996 "Effects of a physician communication intervention on patient care outcomes", *Journal of General Internal Medicine,* vol. 11, no. 3, pp. 147–155.

Kropiwnicki, Z. D.
2007 *Children Speak Out: Trafficking risk and resilience in southeast Europe (regional report)*, Save the Children in Albania, Tirana, Albania, July 2007.

Marshall, P.
2001 "Globalization, migration and trafficking: some thoughts from the south-east Asian region", Occasional Paper No. 1, UN Inter-Agency Project on Trafficking in Women and Children in the Mekong Sub-region, paper to the Globalization Workshop in Kuala Lumpur, 8–10 May 2001, United Nations Office for Project Services, September 2001.

Morrissey, J.P. et al.
2005 "Twelve-month outcomes of trauma-informed interventions for women with co-occurring disorders", *Psychiatric Services*, vol. 56, no. 10, October 2005, pp. 1213–1222.

Nikapota, A. and D. Samarasinghe
1991 *Manual for helping children traumatized by* conflict, UNICEF, Sri Lanka, 1991.

OSCE Office for Democratic Institutions and Human Rights
2004 *National Referral Mechanisms Joining Efforts to Protect the Rights of Trafficked Persons: A practical handbook*, Organization for Security and Co-operation in Europe, Warsaw, 2004.

Pan American Health Organization, Women, Health and Development Program and Organization for American States, Inter-American Commission on Women
2001 "Trafficking for Sexual Exploitation", Fact Sheet of the Program on Women, Health and Development, Washington, DC, July 2001.

Patel, V.
2003 *Where There is No Psychiatrist: A mental health care manual*, Gaskell, London, 2003.

Physicians for Human Rights
2001 *Examining Asylum Seekers: A health professional's guide to medical and psychological evaluation of torture*, PHR, Cambridge, Massachusetts, USA, August 2001.

Pickering, L.K. (Ed.)
2006 *The Red Book: 2006 report of the committee on infectious diseases, 27th Edition*, American Academy of Pediatrics, Oak Grove, Illinois, USA, 2006.

Raval, H.
2005 "Being heard and understood in the context of seeking asylum and refuge: communicating with the help of bilingual co-workers", *Clinical Child Psychology and Psychiatry*, vol. 10, no. 2, 1 April 2005, pp. 197–216.

Ray, John
2007 "Disabled children sold into slavery as beggars, Chinese racketeers living well by exploiting poverty, ignorance", newspaper article, in « guardian.co.uk » and in *The Observer*, 22 July 2007.

Regional Conference on Public Health and Trafficking in Human Beings in Central, Eastern and Southeast Europe
2003 Budapest Declaration on Public Health & Trafficking in Human Beings, Budapest, Hungary, March 2003.

Rende Taylor, L.
2008 Guide to ethics and human rights in counter-trafficking. Ethical standards for counter-trafficking research and programming. United Nations Inter-agency Project on Human Trafficking.

Salasin, S.
2005 "Evolution of women's trauma-integrated services at the Substance Abuse and Mental Health Services Administration", *Journal of Community Psychology*, vol. 3, no. 4 (special issue on 'Serving the needs of women with co-occurring disorders and a history of trauma'), July 2005, pp. 379–393.

Silverman, J. et al.
2007 "HIV prevalence and prediction of infection in sex-trafficked Nepalese girls and women", *The Journal of the American Medical Association*, vol. 298, no. 5, pp. 536–542.

Smith P. et al.
1999 *A Manual for Teaching Survival Techniques to Child Survivors of Wars and Major Disasters*, Children and War Foundation, Bergen, Norway, 1999.

Stellman, J. M. (Editor-in-chief)
1998 *Encyclopaedia of Occupational Health and Safety, Fourth Edition*, International Labour Organization, Geneva, 1998.

Surtees, R.
2005 *Second Annual Report on Victims of Trafficking in South-Eastern Europe*, International Organization for Migration, Geneva, 2005.
2005 "Other forms of trafficking in minors: articulating victim profiles and conceptualizing interventions", paper originally presented at Childhoods Conference, Oslo, Norway, 29 June – 3 July 2005, NEXUS Institute to Combat Human Trafficking and International Organization for Migration.

Tiurukanova, E. V. and the Institute for Urban Economics
2006 *Human Trafficking in the Russian Federation: Inventory and analysis of the current situation and responses*, United Nations Children's Fund, International Organization for Migration and Canadian International Development Agency, Moscow, March 2006.

Tribe, R. and H. Raval (Eds.)
2003 *Working with Interpreters in Mental Health*, Brunner-Routledge, Hove, United Kingdom and New York, NY, USA, 2003.

Joint United Nations Programme on HIV/AIDS (UNAIDS)
2007 *Guidelines on Protecting the Confidentiality and Security of HIV Information: Proceedings from a workshop 15–17 May 2006 Geneva, Switzerland, interim guidelines*, UNAIDS, Geneva, 15 May 2007.

United Nations
2006 Convention on the Rights of Persons with Disabilities, adopted on 13 December 2006 by General Assembly Resolution A/RES/61/106, opened for signature 30 March 2007, New York.
2000 Protocol to Prevent, Suppress and Punish Trafficking in Persons, Especially Women and Children, Supplementing the United Nations Convention against Transnational Organized Crime, United Nations, New York, 2000.
1989 Convention on the Rights of the Child, adopted on 20 November 1989 by General Assembly Resolution 44/25, entry into force 2 September 1990.

1979 Convention on the Elimination of All Forms of Discrimination Against Women, adopted in 1979 by General Assembly Resolution 34/180, entry into force September 1981.

United Nations Children's Fund

2006 *Guidelines on the Protection of Child Victims of Trafficking,* UNICEF Technical Notes, provisional version 2.1, UNICEF, New York, September 2006.

2006 *Reference Guide on Protecting the Rights of Child Victims of Trafficking in Europe*, UNICEF, 2006.

2005 *Manual for Medical Officers Dealing with Child Victims of Trafficking and Commercial Sexual Exploitation (Manual for Medical Officers Dealing with Medico-Legal Cases of Victims of Trafficking for Commercial Sexual Exploitation and Child Sexual Abuse),* UNICEF and the Department of Women and Child Development, Government of India, New Delhi, 2005.

2003 *Guidelines for the Protection of the Rights of Children Victims of Trafficking in South Eastern Europe*. Unpublished draft.

2006 *Action to Prevent Child Trafficking in South Eastern Europe,* preliminary assessment, UNICEF and Terre des homes Foundation, Geneva, June 2006.

Trafficking in children and child involvement in beggary in Saudi Arabia, United Nations Children's Fund Gulf Area Office, Riyadh, Saudi Arabia, undated.

United Nations Educational, Scientific and Cultural Organization

2006 *Human Trafficking in Nigeria: Root Causes and Recommendations,* Policy Paper No. 14.2(E), UNESCO, Paris, 2006.

United Nations High Commissioner for Refugees

2003 *Sexual and Gender-Based Violence against Refugees, Returnees, and Internally Displaced Persons: Guidelines for prevention and response*, UNHCR, Geneva, May 2003.

1999 *Reproductive Health in Refugee Situations: An inter-agency field manual*, UNHCR, Geneva, 1999.

United Nations Office on Drugs and Crime

2008 *Toolkit to Combat Trafficking in Persons*, UNODC Global Programme against Trafficking in Human Beings, Vienna, 2008.

United Nations Office of the High Commissioner for Human Rights

2002 *Recommended Principles and Guidelines on Human Rights and Human Trafficking*, Report of the United Nations High Commissioner for Human Rights to the Economic and Social Council (E/2002/68/Add.1), United Nations Economic and Social Council, New York, 20 May 2002.

1990 Guidelines for the Regulation of Computerized Personal Data Files, adopted by General Assembly Resolution 45/95 of 14 December 1990.

1989 Convention on the Rights of the Child, Adopted and Open for ratification by General Assembly Resolution 44/25 of 20 November 1989, entry into force 20 September 1990.

United Nations Population Fund

2008 *Mental, Sexual & Reproductive Health: UNFPA Emerging Issues*, UNFPA, New York, USA, 2008.

2007 *Sexual and Reproductive Health of Persons with Disabilities: Emerging UNFPA Issues*, UNFPA, New York, USA, 2007.

2002 *Trafficking in Women, Girls and Boys: Key issues for population and development programmes*, Report on the Consultative Meeting on Trafficking in Women and Children, Bratislava, Slovak Republic, 2-4 October 2002, UNFPA, New York, 2003.

United States Centers for Disease Control and Prevention

2006 "Sexually Transmitted Diseases Treatment Guidelines, 2006", *Morbidity and Mortality Weekly Report*, vol. 55, no. RR-1, 4 August 2006.

United States Centers for Disease Control and Prevention et al.

2008 *Guidelines for Prevention and Treatment of Opportunistic Infections among HIV-Exposed and HIV-Infected Children*, Centers for Disease Control and Prevention, National Institutes of Health, Infectious Disease Association of America, Pediatric Infectious Disease Society and American Academy of Pediatrics, USA, 20 June 2008.

United States Department of Health & Human Services

2008 *Fact Sheet: Human Trafficking*, United States Department of Health and Human Services Administration of Children & Families, Campaign to Rescue and Restore Victims of Human Trafficking, Washington, DC, USA, January 2008.

2007 *Common Health Issues Seen in Victims of Human Trafficking*, web information available at « *http://www.acf.hhs.gov/trafficking/campaign_kits/tool_kit_health/health_problems.html* » United States Department of Health and Human Services Administration of Children & Families, Campaign to Rescue and Restore Victims of Human Trafficking, Washington, DC, Campaign to Rescue and Restore Victims of Human Trafficking, October 2007.

United States Department of State
2007 *Trafficking in Persons Report, June 2007*, Office of the Under Secretary for Democracy and Global Affairs and Bureau of Public Affairs, Washington, DC, USA, revised June 2007.
2007 *Health Consequences of Trafficking in Persons*, fact sheet, Department of State Office to Monitor and Combat Trafficking in Persons, Washington, DC, USA, 8 August 2007.

University of California at Davis
"A guide to agricultural heat stress", newsletter, Agricultural Personnel Management Program, Davis, California, USA, undated.

West, A.
2003 "At the margins: street children in Asia and the Pacific", Asian Development Bank, Poverty and Social Development Papers, no. 8, October 2003.

Willis, B. and B. Levy
2002 "Child prostitution: global health burden, research needs, and interventions", *The Lancet,* vol. 359, no. 9315, 20 April 2002, pp. 1417–1422.

Wolffers, I. et al.
2003 "Migration, human rights, and health", *The Lancet*, vol. 362, no. 9400, 13 December 2003, pp. 2019–2020.

World Bank
Disability in Africa Region, web information/portal, available at « *http://web.worldbank.org/WBSITE/EXTERNAL/TOPICS/EXTSOCIALPROTECTION/EXTDISABILITY/0,,contentMDK:20183406~menuPK:417328~pagePK:148956~piPK:216618~theSitePK:282699,00.html*» World Bank, undated (accessed 4 January 2009).

World Health Organization

2007 *Guidance on Global Scale-Up of the Prevention of Mother-to-Child Transmission of HIV: Towards universal access for women, infants and young children and eliminating HIV and AIDS among children*, WHO, Geneva, 2007.

2007 *The WHO Strategic Approach to strengthening sexual and reproductive health policies and programmes*, WHO, Geneva, 2007.

2007 *WHO Recommended Interventions for Improving Maternal and Newborn Health*, Integrated Management of Pregnancy and Childbirth Guidelines, WHO, Geneva, 2007.

2006 *Pregnancy, Childbirth, Postpartum and Newborn Care: A guide for essential practice*, Integrated Management of Pregnancy and Childbirth Guidelines WHO, Geneva, 2006.

2006 *Standards for Maternal and Neonatal Care*, Integrated Management of Pregnancy and Childbirth Guidelines, WHO, Geneva, 2006.

2005 *Child and Adolescent Mental Health Policies and Plans*, mental health policy and service guidance package, WHO, Geneva, 2005.

2005 *Emergency Contraception*, Fact Sheet no. 244, WHO, Geneva, revised October 2005.

2005 *Mental Health Atlas, Revised* Edition, WHO, Geneva, 2005.

2005 *WHO Resource Book on Mental Health, Human Rights, and Legislation*, WHO, Geneva, 2005.

2005 *Sexually Transmitted and Other Reproductive Tract Infections: A guide to essential practice*, WHO, Geneva, 2005.

2005 *WHO Online Sex Work Toolkit: Targeted HIV/AIDS prevention and care in sex work settings*, web information available at « www.who.int/hiv/pub/prev_care/swtoolkit/en/ » WHO, Geneva, 2005.

2004 *Antiretroviral Drugs for Treating Pregnant Women and Preventing HIV Infection in Infants: Guidelines on care, treatment and support for women living with HIV/AIDS and their children in resource-constrained settings*, WHO, Geneva, 2004.

2003 *International Migration, Health & Human Rights*, Health and Human Rights Publication Series, no. 4, December 2003.

2002 *Guidelines for medico-legal care for victims of sexual violence*, WHO, Geneva, 2003.

2001 *International Classification of Functioning, Disability and Health*, WHO, Geneva, 2001.

2001 *Recommended Strategies for Prevention and Control of Communicable Diseases*, WHO, Geneva, 2001.

1999 *Ethical Practice in Laboratory Medicine and Forensic Pathology*, WHO Regional Publications, Eastern Mediterranean Series, no. 20, WHO Regional Office for the Eastern Mediterranean, 1999.

1998 *Mental Disorders in Primary* Care, A WHO Educational Package, WHO, Geneva, 1998.

1997 *Syndromic Case Management of Sexually Transmitted Diseases: A guide for decision-makers, health care workers and communicators*, WHO Regional Office for the Western Pacific, Manila, 1997.

"Screening and brief intervention for alcohol problems in primary health care", web information available at « *www.who.int/substance_abuse/activities/sbi/en/index.html* » WHO, Geneva, undated, accessed 3 January 2009.

World Health Organization Department of Reproductive Health and Research and Johns Hopkins Bloomberg School of Public Health Center for Communication Programs INFO Project

2008 *Family Planning: A global handbook for providers (2008 Edition)*, CCP and WHO, Baltimore and Geneva, 2008.

World Health Organization and International Labour Organization

2007 Post-Exposure Prophylaxis to Prevent HIV Infection: Joint WHO/ILO guidelines on post-exposure prophylaxis (PEP) to prevent HIV infection, WHO, Geneva, 2007.

World Health Organization and United Nations High Commissioner for Refugees

2004 *Clinical Management of Rape Survivors: Developing protocols for use with refugees and internally displaced persons, revised edition*, WHO and UNHCR, Geneva, 2004.

Zimmerman, C. and C. Watts,

2003 *WHO Ethical and Safety Recommendations for Interviewing Trafficked Women*, World Health Organization, Geneva 2003.

Zimmerman, C. et al.

2008 "The health of trafficked women: A survey of women entering posttrafficking services in Europe", *American Journal of Public Health*, vol. 98, no. 1, January 2008, pp. 55–59.

Zimmerman, C. et al.

2006 *Stolen Smiles: The physical and psychological health consequences of women and adolescents trafficked in Europe*, London School of Hygiene and Tropical Medicine, London, 2006.

Zimmerman, C. et al.
2003 *The Health Risks and Consequences of Trafficking in Women and Adolescents: Findings from a European study*, London School of Hygiene and Tropical Medicine, London, 2003.

Other resources

Centres, institutions, organizations and web sites

United Nations Global Initiative to Fight Trafficking in Persons (UN.GIFT)
http://www.ungift.org

United States Centers for Disease Control and Prevention
http://www.cdc.gov/

United States Centers for Disease Control and Prevention
Electronic library of construction occupational safety and health
http://www.cdc.gov/elcosh/

Gender Violence & and Health Centre, London School of Hygiene & Tropical Medicine
http://www.lshtm.ac.uk/genderviolence/recent.htm

The National Child Traumatic Stress Network
www.nctsn.org

Disability, Abuse and Personal Rights Project (resource directory on abuse and disability)
http://disability-abuse.com/

Respond (this is an example of an organization that supports persons who are disabled)
http://www.respond.org.uk

An example of a web resource for combating human trafficking
http://www.humantrafficking.org

An example of an online resource on dignity and rights of persons with disabilities
http://www.un.org/disabilities/default.asp?navid=11&pid=25

An example of an online resource related to child trafficking
http://www.childtrafficking.com

Many thanks to the individuals from the following organizations who provided technical input and peer review for this document:

Animus Foundation

Fundación Social Fénix

Ghana Health Services

Global Health Promise

Harvard School of Public Health

International Labour Organization

International Organization for Migration

The Joint United Nations Programme on HIV/AIDS

Centre for Anxiety, Disorders and Trauma Maudsely Hospital

Kings College London Institute of Psychiatry

London School for Hygiene and Tropical Medicine

Mahidol University Institute for Population and Social Research

Office of the United Nations High Commissioner for Human Rights

Pan American Health Organization

UC Davis School of Medicine

UK-Sri Lanka Trauma Group

United Nations Population Fund

US Agency for International Development (Bureau for Global Health)

Victorian Institute of Forensic Medicine

World Health Organization

17 route des Morillons
1211 Geneva 19
Switzerland
Tel: +41.22.717 91 11 | Fax: +41.22.798 61 50
E-mail: hq@iom.int | Internet: http://www.iom.int
