# Supplementary material for: Health Care Providers and Human Trafficking: What do They Know, What do They Need to Know? Findings from the Middle East, the Caribbean, and Central America
Source: Front Public Health. 2015 Jan 29;3:6. doi: 10.3389/fpubh.2015.00006 (PMC4310216; doi:10.3389/fpubh.2015.00006)
Supplement: Supplementary file 1 [file Presentation_1.ZIP › IOM_LSHTM_CTP Training Guide_2012.pdf]

CARING FOR TRAFFICKED PERSONS

GUIDANCE FOR HEALTH PROVIDERS

TRAINING

FACILITATOR'S GUIDE

International Organization for Migration (IOM)

LONDON SCHOOL *of* HYGIENE &TROPICAL MEDICINE

The opinions expressed in this guide are those of the authors and do not necessarily reflect the views of the International Organization for Migration (IOM). The designations employed and the presentation of the material throughout the guide do not imply the expression of any opinion whatsoever on the part of IOM concerning the legal status of any country, territory, city or area, or of its authorities, or concerning its frontiers or boundaries.

IOM is committed to the principle that humane and orderly migration benefits migrants and society. As an intergovernmental organization, IOM acts with its partners in the international community to: assist in meeting the operational challenges of migration; advance understanding of migration issues; encourage social and economic development through migration; and uphold the human dignity and well-being of migrants.

Editors: Rosilyne Borland
International Organization for Migration
Migrant Assistance Division

Cathy Zimmerman
London School of Hygiene & Tropical Medicine
Gender Violence & Health Centre

Publisher: International Organization for Migration
17 route des Morillons
1211 Geneva 19
Switzerland
Tel.: + 41 22 717 91 11
Fax: + 41 22 798 61 50
E-mail: hq@iom.int
Internet: www.iom.int

This publication was made possible through support provided by the United States Department of State, under the terms of Award No. S-SGTIP-10-GR-0026. The opinions expressed herein are those of the authors and do not necessarily reflect the views of the United States Department of State.

ISBN 978-92-9068-655-2

© 2012 International Organization for Migration (IOM)
© 2012 London School of Hygiene & Tropical Medicine (LSHTM)

All rights reserved. No part of this publication may be reproduced, stored in a retrieval system, or transmitted in any form or by any means, electronic, mechanical, photocopying, recording, or otherwise without the prior written permission of the publisher.

46_12

CARING FOR TRAFFICKED PERSONS

GUIDANCE FOR HEALTH PROVIDERS

TRAINING

FACILITATOR'S GUIDE

International Organization for Migration (IOM)

LONDON SCHOOL of HYGIENE & TROPICAL MEDICINE

Acknowledgements

This *Facilitator's Guide* was made possible through the generous support of the United States Department of State Office to Monitor and Combat Trafficking in Persons, grant number S-SGTIP-10-GR-0026. The training materials are based on the *Caring for Trafficked Persons: Guidance for Health Providers* handbook published in 2009 with the support of the United Nations Global Initiative to Fight Trafficking in Persons (UN.GIFT). The development of the training materials was coordinated by the International Organization for Migration (IOM) and the Gender Violence & Health Centre of the London School of Hygiene & Tropical Medicine (LSHTM).

We were privileged to have the support and participation of a network of IOM counter-trafficking and health experts throughout this project. The training package was developed and improved during pilot trainings in English, Arabic and Spanish with health providers in the following countries: Antigua and Barbuda, Belize, Costa Rica, Egypt, El Salvador, Guyana, and Jordan. Principal trainers and contributors included Rosilyne Borland, Sarah Boutros, Sarah Craggs, Kristen Dadey, Poonam Dhavan, Ana Hidalgo, Maria Moreiane, Chissey Mueller, Marija Nikolovska, Mohamed Refaat, Patience Sizani, Shaiban Taqa, Carlos Van Der Laat, Haley West, Tea Zakaria, and Cathy Zimmerman. Thank you to Oxford Change Management for assistance in developing the early stages of the training package.

This guide was developed with the support of Dr. Davide Mosca, Director of the Migration Health Division and Laurence Hart, Director of the Migrant Assistance Division of the International Organization for Migration.

Table of Contents

Acknowledgements i

Introduction 3

Part 1: Preparing for the Training 5
- Prepare Your Training Plan 7
- Know the Local Context 9
- Prepare Your Materials 11

Part 2: Core Training 13
- Core Training Overview 16
- Session Guide 1: What is Trafficking in Persons? 18
- Session 1 Handouts 28
- Session Guide 2: Health Consequences 32
- Session 2 Handouts 49
- Session Guide 3: Trauma-informed Care 52
- Session Guide 4: Role of the Health Care Provider 64
- Session 4 Handouts 77
- Session Guide 5: Guiding Principles 82
- Session 5 Handouts 90
- Session Guide 6: Local Context and Next Steps (optional) 94
- Session 6 Handouts 95

Conclusion 101

Annexes 103
- Annex 1: General Training Preparations 105
- Annex 2: Additional Suggested Topics 110

Introduction

Introduction

Welcome to the *Caring for Trafficked Persons: Guidance for Health Providers* Training!

For health care providers, trafficking in persons is best understood as a serious health risk because as with other forms of violence, it is associated with physical and psychological harm. The informed and attentive health care provider can play an important role in assisting and treating individuals who may have suffered unspeakable and repeated abuse. For health practitioners, diagnosing and treating trafficked persons can pose a range of new challenges related to care provision. In recognition of the need for global guidance, the International Organization for Migration (IOM) and the Gender Violence & Health Centre of the London School of Hygiene & Tropical Medicine (LSHTM) developed a global handbook in 2009 through an expert group initiative, funded by the United Nations Global Initiative to Fight Human Trafficking (UN.GIFT). *Caring for Trafficked Persons: Guidance for Health Providers* combines research, field experience and good practice into a practical tool for those who provide health services to trafficked persons, whether identified victims or populations who may include unidentified victims or other exploited persons.

In 2010, IOM and LSHTM began phase two of the project, focused on building the capacity of health providers through the development of a training package to complement the release of the handbook in multiple languages. The training package is based on the handbook and is initially being released in English, Spanish and Arabic, thanks to the generous support of the United States Department of State Office to Combat and Monitor Trafficking in Persons.

This training package is composed of six Sessions, which together make up the Core Training for health providers, based on the *Caring for Trafficked Persons* handbook. The Core Training takes approximately two days, and offers some flexibility depending on the activities used and whether additional topics from the handbook are added.

This *Facilitator's Guide* and accompanying materials have been developed for individuals who wish to carry out training for health providers. The training is designed for all types and levels of health providers (e.g. nurses, medical technicians, doctors, counsellors, etc.), particularly those actively providing services.

This *Facilitator's Guide* contains two parts:

Part 1 contains basic information for you, the facilitator, on how to prepare before the training takes place. Additional general information about facilitating training events can be found in Annex 1.

Part 2 contains the information that you need to facilitate the *Caring for Trafficked Persons* Core Training. For each Session of the Core Training, you will find:

- An overview of the Session including: objectives and timetable
- Facilitator notes giving detailed instructions on how to facilitate each part of the Session, including activities
- PowerPoint slides (you can copy these onto flip charts if you do not have access to a projector or computer)
- Handouts related to the Session

Information on additional topics and related learning objectives based on content in the handbook not covered in the Core Training is included in Annex 2. Facilitators may wish to consider including these more specific topics in longer trainings or for future trainings with the same participants.

The full training package is also available online at the IOM Bookstore (http://publications.iom.int/bookstore) and at the LSHTM website (http://genderviolence.lshtm.ac.uk/category/reports/).

We hope that you find the *Caring for Trafficked Persons Training* useful!

PART ONE

Preparing for the Training

Prepare your Training Plan

It is absolutely essential that you, the facilitator, prepare yourself for the training in advance. This section provides specific guidance on preparing your training plan, necessary preparations for training on health and human trafficking, as well as other relevant information to conduct a successful training.

Before you can prepare your training materials you need to decide whether any changes should be made to the training plan itself. Remember, the training is based on the assumption that, as a minimum, the full two-day Core Training content will be delivered, with small adaptations to ensure the specific context and examples fit the needs of your participants and your context.

SEE PAGE 16 Core Training Timetable Overview

There are a number of factors to consider as you prepare your training plan:

Expectations

- What are the requirements of your training audience / participants?
- What are the local needs driving the desire for training and are these needs met by the content included here?
- What is the impact or change in provider practice you hope to achieve and will you achieve your goals with this training package as designed?

REMEMBER This training is designed for *health providers* who are providing services for: 1) identified trafficked persons and 2) vulnerable populations that may include trafficked persons who have not yet been identified. While the content may be useful for non-health service participants, keep in mind that the training is most effective when the participants are mostly health providers. The training is designed for all types and levels of health providers (e.g. nurses, medical technicians, doctors, counsellors, etc.), particularly those actively providing services.

CONSIDER If not already part of the training team, it may be useful to include a representative from the local counter-trafficking coalition or another local counter-trafficking service provider as a participant, to further enrich the discussion. While the majority of participants should be health providers, including a participant with local counter-trafficking experience can add depth and practical knowledge about resources and referral to the discussions and activities.

Timing

- How much time can you and your participants reasonably set aside for this training?
- What schedule is acceptable in your context? For example, in some countries it is preferable to begin and end early, while in others a later start is expected.
- How much time should you schedule for lunch and breaks?

CONSIDER In many settings it is common to have an opening session or ceremony to welcome participants. This session can be informal or formal, and may include higher level representatives of organizations and institutions supporting the training. Be sure to plan for this time if appropriate and to allow time for high-level guests to leave after the opening remarks.

REMEMBER

The training is designed for two days but can be shortened or lengthened depending on the activities included. Local expectations should be taken into account when adapting the suggested agenda included in this training package.

CONSIDER

Health providers often have inflexible work schedules or service obligations and might require training timing that differs from other typical training schedules in your context. Especially if providers' schedules are officially mandated by the public health system, the training may need to take place around that schedule.

Training team

- How many trainers will you need to deliver the training? Who will facilitate each Session?
- Do you need the support of a specialist for parts of the training (e.g. local counter-trafficking specialist, local health specialist, etc.)?

IMPORTANT

It is essential to have someone involved in the training who is familiar with human trafficking, ideally as a member of the training team. If you do not have experience with this topic, it is highly recommended that you invite a local counter-trafficking partner to participate as part of the training team.

REMEMBER

Even if you are experienced in counter-trafficking, if you are not from the city / country where you are training, it is important to involve someone from the local context to make the information relevant and who can answer context-specific questions. If appropriate, you might invite them to deliver one Session of the training.

CONSIDER

It is very useful to have more than one person facilitate this training. For example, if one person on the training team has more experience with human trafficking, it would make sense for that person to facilitate Session 1. A trainer with more direct assistance and health experience would likely be most comfortable with Session 2.

CONSIDER

While we recommend the training be done in one language, in some settings it may be important to provide handouts in alternative languages to some participants or to consider simultaneous translation for some trainers. This will require additional time and must be considered in the training plan.

The training assumes a total of up to 25 participants in order to allow small group work of no more than five or six people per group, as well as the possibility for productive full group discussions. Depending on the number of participants, you will need to adjust the estimated timing for each section appropriately.

Once you've finalized your training plan, you will need to carefully prepare for the training by preparing yourself, your materials, and the training room.

Know the Local Context

These training materials have been developed to provide a flexible resource for use in many settings. As a result they may not always reflect the specific local context exactly. We recommend that you, as the facilitator, *always* investigate local issues, resources and examples prior to commencing any training to be sure the training reflects the participants' context (e.g. context-specific examples).

IMPORTANT

Make sure you know as much as you can about human trafficking in the local context.

CHECKLIST

In particular we suggest you try to have basic information on the following:

- ✓ Is there a local anti-trafficking law in this country? Does it follow the Palermo Protocol definition of human trafficking and related recommendations?
- ✓ Be familiar with other related legislation (e.g. domestic violence / gender-based violence, etc.).
- ✓ Have there been investigations and prosecutions of traffickers in this country?
- ✓ Is there a national counter-trafficking committee, taskforce or collation? Is the health sector involved?
- ✓ What are the migration flows (international and internal) related to this country and region?
- ✓ What are the major work sectors where exploitation and abuse are thought to take place?
- ✓ Is there a shelter for trafficked persons in this country? Does it accept men, women, boys and girls? Are there other shelters available (e.g. domestic violence)?
- ✓ Is there a specialized non-governmental organization working on human trafficking?
- ✓ Is there a hotline or other number to call related to human trafficking? Are there other relevant helplines to social services?
- ✓ Is there a formal referral system or other standard procedures related to the protection of trafficked persons when they are identified?
- ✓ Have any studies related to trafficking in persons been done that include this context? Did they include health?
- ✓ Have any major events or conferences taken place recently on this topic?
- ✓ Has trafficking in persons been in the news recently? Are there any specific myths or stereotypes that are included in this media coverage?
- ✓ Are numbers or estimates of trafficking victims assisted available?
- ✓ What are the local forensic procedures (e.g. who is contacted to carry out forensic exams, a rape crisis centre? A specialized police team? Specialized emergency room staff?).
- ✓ What are the medical reporting requirements (e.g. when is it mandatory to report child abuse, child sexual abuse, rape, etc.?).

REMEMBER

While it may be difficult to find all of the above information, it is important to know as much as possible. Participants may ask some of these questions and some may also know the answers.

IMPORTANT

Be sure you understand the definition of human trafficking as described in the Palermo Protocol (see Session 1). This is especially important during the first session, but questions may arise throughout the training.

CONSIDER

You may want to use some of the following resources to find information on trafficking in persons in your context, such as:

- IOM website (http://www.iom.int/jahia/Jahia/activities/by-theme/regulating-migration/counter-trafficking)
- LSHTM website (http://genderviolence.lshtm.ac.uk/category/reports/)
- US Department of *State Trafficking in Persons Report* (http://www.state.gov/j/tip/)
- UN.GIFT.HUB (http://www.ungift.org/knowledgehub/)
- International Labour Organization (http://www.ilo.org/global/topics/forced-labour/lang--en/index.htm)
- Child trafficking digital library (http://childtrafficking.com/)

SEE PAGE 89

Referral Mapping Form

CHECKLIST

In addition to learning the local context, you should prepare yourself to use the training materials:

- ✓ Read through the *Facilitator's Guide* and handbook carefully and make sure you understand the content and can answer questions.
- ✓ Make sure you know how the Sessions work – what do you need to do, in what order? Are you clear on the instructions for the activities and when they should happen?
- ✓ Practice your Sessions – particularly any presentations that you will be making. You will feel – and appear – much more confident if you have done a presentation before.

SEE PAGE 15

Part 2: Core Training

Prepare Your Materials

If you will be copying slides or instructions onto flip charts, do this well in advance: flip charts written in a hurry are hard to read!

Make copies of any handouts you will need.

CONSIDER

It is often useful to have a sign-in sheet prepared to gather basic contact information of the participants, and as a record of who has attended.

CONSIDER

Many facilitators like to bring a printout of the training session slides to have on hand, just in case the projector malfunctions. This can allow a facilitator to continue the session despite unexpected problems.

Make sure you have sufficient copies of the *Caring for Trafficked Persons* handbook and any other reference materials you plan to make available to participants during the training.

IMPORTANT

One of the most important pieces of information you should have available is who participants can call in the local context if they suspect someone is a victim of trafficking. This may be a local hotline, a shelter or non-governmental organization, or an international organization like IOM. Check *before* the training and have this information on hand, ideally as a handout that participants can take with them.

IMPORTANT

The *Caring for Trafficked Persons* handbook contains much more detailed information than what is included in the training. Participants should receive a copy during the training if possible, as some people prefer to refer to reference materials throughout the training. The handbook is available online for free download or can be shipped upon request (see http://publications.iom.int/bookstore/index.php?main_page=product_info&products_id=510).

Check that any equipment (e.g. projector, computer, microphones) are working and that you have the necessary electronic adaptors, extension cords, speakers, tape, scissors, markers, or any other materials you need well in advance. Many trainers bring extra supplies just in case they need them.

Please see Annex 1 for additional general information on successful training preparations, including preparing your training room, facilitating case studies and role plays, facilitating group discussions, adult learning, and interactive presentations.

PART

Core Training

TWO

The following pages present the Core Training based on the *Caring for Trafficked Persons: Guidance for Health Providers* handbook.

LEARNING OBJECTIVES

By the end of this two-day training, participants will be able to:

- Understand what is human trafficking
- Identify some of the major health consequences of trafficking
- Recognize key features of trauma-informed care
- Recognize techniques for provider and patient safety
- Understand the benefits of incorporating specialized care approaches for trafficked persons
- Identify the possibilities and limitations of role of health care providers in the human trafficking cycle

The training is based on the assumption that, as a minimum, all participants will do the Core Training, estimated to take two days. While experienced trainers may be able to shorten the Core Training to 1 ½ days, depending on the activities they choose to include, it is recommended that the material in the Core Training be the basis of any *Caring for Trafficked Persons* training.

SEE PAGE 103

Annex 2: Additional Suggested Topics. The handbook contains much more detailed information and additional content can be added to the Core Training if time allows, or in future trainings with the same participants.

The Core Training contains six Sessions. This part of the *Facilitator's Guide* contains detailed information about each of the training sessions in detail in order to give you, the facilitator, clear guidance on how to run each session.

REMEMBER

Before you begin reviewing in detail the content of the training sessions, you should first prepare yourself and your overall training plan (see Part 1 of this guide).

Core Training Overview

TIMETABLE OVERVIEW

Core Training (internal agenda for facilitator)

The following is a breakdown of the suggested timetable for the two-day Core Training. This is an *internal* version of the agenda, to give you a sense of what is included. An external version is available in the handouts section and on the CD included with this guide.

**Day One**	
9:00 – 9:30	**Arrival of Participants and Registration**
9:30 – 10:00	**Opening Remarks**<br>• Welcome on behalf of training organization, authorities
10:00 – 10:30	**Session 1: What is Human Trafficking?**<br>• Introduction of training team<br>• Icebreaker (hand-raising)<br>• Introductions of participants / expectations<br>• Introduction of *Caring for Trafficked Persons handbook* / participants receive handbook
10:30 – 11:00	*Coffee Break*
11:00 – 12:30	**Session 1: What is Human Trafficking? Continued**<br>• PPT Pack 1 – What is TIP?<br>• Poll Quiz: Myths and Realities<br>• VIDEO or story
12:30 – 13:30	*Lunch*
13:30 – 15:00	**Session 2: Health Consequences**<br>• PPT Pack 2 – Health Consequences<br>• Cases illustrate health consequences (in the slides)<br>• EXERCISE: brainstorm health consequences in each category based on cases
15:00 – 15:30	*Coffee Break*
15:30 – 16:30	**Session 3: Trauma-informed Care**<br>• PPT Pack 3 – Trauma informed care
16:30 – 17:00	**Reflection and Wrap Up**<br>• EXERCISE Individual: reflection on what you would do differently based on today's learning (no reporting – bring back tomorrow)<br>• Wrap up by facilitator

IMPORTANT

The content for day one has less flexibility than the content for day two. The first day of the Core training provides essential information on trafficking in persons, health consequences, and the trauma-informed care approach. The second day of training is designed to reinforce some of the main points from day one, to allow participants to practice some of the skills, and to create an opportunity for a final session to complement on-going counter-trafficking work in the local context. Alternatively, some trainers may prefer to expand the content of the first day by allowing more time for small group activities, or to end the second day earlier and to cut some of the suggested activities – these options are included in the Session Guides that follow.

REMEMBER

This timetable is meant as a suggestion, based on successful trainings in multiple regions and languages. However, facilitators should adapt content as needed to better reflect their setting and participants, as described in Part 1.

**Day Two**	
9:00 – 9:30	**Arrival of Participants and Welcome Back**<br>• Ice-breaker<br>• Expectations Review
9:30 – 10:30	**Session 4: Role of the Health Provider**<br>• PPT Pack 4 – Role of Health Provider
10:30 – 11:00	*Coffee Break*
11:00 – 12:30	**Role of the Health Provider Continued**<br>• Role play: Practicing trauma-informed care
12:30 – 13:30	*Lunch*
13:30 – 14:00	**Session 5: Guiding Principles**<br>• PT Pack 5 – Guiding Principles
14:00 – 15:00	**Local Context and Next Steps (optional session)**<br>• Referral networking session / Presentation by local partner
15:00 – 15:30	*Coffee Break*
15:30 – 16:30	**Conclusions and Wrap-up**

SEE PAGE 28

Session 1 Handouts

Detailed notes on each session are included in Session Guides in the following pages. Each Session Guide includes:

- Timetable overview
- Session learning objectives
- Preparation and materials required
- Session guidance (slide by slide notes and commentary)
- Session handouts (e.g. external agenda)

Please also see the CD included with this *Facilitator's Guide*, which contains the PowerPoint presentations and other materials in modifiable formats.

Session Guide 1: What is Trafficking in Persons?

Session 1: TIMETABLE OVERVIEW

This session is estimated to take 2-3 hours, including the time for introductions and overview of the training. In most settings, the training event may also include an opening session as well as a coffee break. This session is therefore anticipated to take the morning of the first day of the Core Training.

Session 1: LEARNING OBJECTIVE

By the end of this session, participants will be able to:

- Understand what is human trafficking

CONSIDER

It is recommended to build extra time into your morning session to allow for participants or opening guest speakers to arrive late.

IMPORTANT

If adjusting the training schedule (start time, breaks, etc.) be sure to do this before the training (see Part 1).

PREPARATION & MATERIALS REQUIRED

- Be sure to have your sign-in sheet for participants ready, as well as copies of the external agenda and other handouts.
- You will need to be well-acquainted with the slides for this module, especially those you will use to help participants understand the elements and definition of human trafficking.
- If you don't have access to PowerPoint, a computer and a projector in the training room, prepare flip charts in advance of the training with the slide content.
- Materials you need include flip charts and markers, as well as something to stick the paper to the walls.

SEE PAGE 28

Session 1 Handouts

REMEMBER

The UN Protocol to Prevent, Suppress and Punish Trafficking in Persons (herein after referred to as the Palermo Protocol because it was developed in Palermo, Italy) should be used as the basis for understanding the definition of human trafficking (see handouts for Session 1), regardless of the local context. Review it carefully before this session.

IMPORTANT

Be sure you are aware of the local counter-trafficking context before this session (see Part 1).

Slide 1

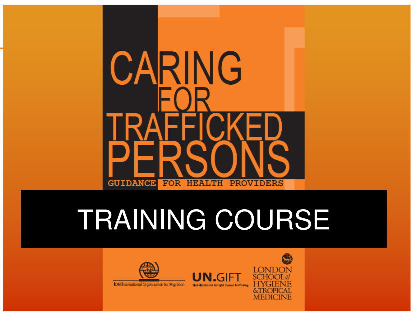

CONSIDER

This introductory slide can be up on the screen as participants arrive and during any opening remarks, if appropriate.

NOTE

It may be appropriate to plan an opening session which includes welcome remarks on behalf of trainers, participating organizations and possibly authorities from the health system. Be sure to introduce yourself and other members of the training team. It is useful to provide some background about why the training is taking place and the experience of the facilitator / organization leading the training.

ACTIVITY

Ice-breaker and participant introductions (10 minutes). Go around the room and ask participants to share their name and any other relevant information (e.g. where they work, what kind of health provider they are, what country or community they are from).

ACTIVITY

Assessing participants' level of experience with topics related to trafficking in persons (5 minutes). It is important for you the facilitator to know a bit more about the relevant experience of participants in the room. One way to quickly have this information is to conduct a poll. Ask participants to raise their hands in response to the following questions:

- How many of you have ever worked with trafficked persons?
- How many have ever worked with victims of domestic violence? Sexual violence and abuse?
- How many have worked with migrants?
- How many think that trafficking is a problem in this country?
- How many of you have been part of a training on trafficking in persons before? A training on health and trafficking in persons?

Feel free to ask follow up questions as needed, but keep an eye on the time. This poll will give everyone a sense of the experience in the room, and you as the facilitator a sense of who might contribute usefully to different discussions.

ALTERNATE ACTIVITY

Expectations (10 minutes). If time permits, it can also be useful to go around the room and ask participants to share their expectations for the training. Write these on a flip charts. This helps you get a sense of what your participants hope to achieve during the training, and can reinforce the learning objectives. As a facilitator, you can also refer back to these during the training as useful to measure progress. It may be useful to save this flip charts or to paste it on a wall for use later in the training.

CONSIDER

You may want to ask participants to:

- Switch off their mobile phones or put them on silent
- Respect the confidentiality of any discussions that take place during the training
- Respect each other by listening to one another and allowing all members of the group to contribute
- Take comfort breaks as required

IMPORTANT

It can be useful to tell participants that this is a *non-clinical* training specifically designed for health providers. They are the experts on how to provide health services. This training is to help them learn about how to adapt specialized approaches with trafficked persons.

Slide 2

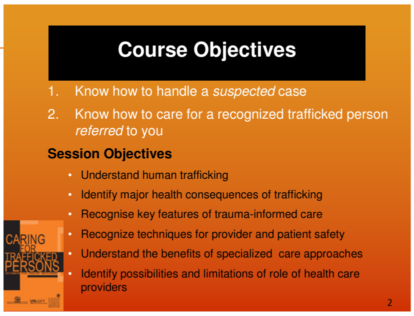

NOTE

This slide appears at the beginning of every session, to remind participants of the main objectives of the course: 1) know how to handle a suspected case and 2) know how to care for a recognized trafficked person. It is useful to remind participants of the overall structure of the course, and session objectives.

IMPORTANT

Emphasize that there are generally two situations in which a health provider might encounter a person who has been trafficked:

1. They may *suspect* someone is a victim of trafficking.
2. They may be caring for someone who is referred to them and is already *identified* as a trafficked person.

The guidance in this training and in the *Caring for Trafficked Persons* handbook is useful for both situations.

Slide 3

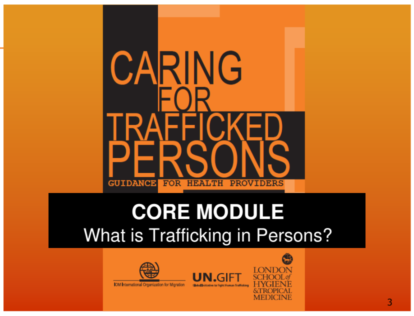

NOTE

This slide begins Session 1: What is Trafficking in Persons?

This is a good moment to provide a brief overview of the agenda for the day, to let participants know that you will be spending the morning on this topic.

Slide 4

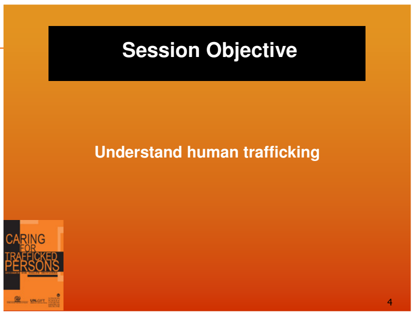

LEARNING OBJECTIVES

By the end of this session, you will be able to understand what is human trafficking.

This learning objective is the focus of the current session.

Slide 5

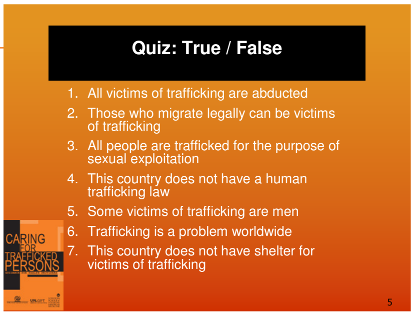

ACTIVITY

Poll / Quiz: Myths and Realities (10 minutes). Before you discuss human trafficking, it is useful to find out whether there are misperceptions among participants. Ask participants if they think the statsements are true or false. You can have a participant read a statement and then ask the group to raise their hands if they feel it is "true", and then if they feel it is "false".

IMPORTANT

Do not give the answers at this stage! Tell participants we will come back to this later in the session.

ALTERNATE ACTIVITY

Action voting: Myths and Realities (15 minutes). In some contexts, participants will respond well to activities that include movement. Ask participants to stand up and to move to an open part of the training room. Pick two people and have them stand at each end of the open space (person 1 is "true" and person 2 is "false"). Read out the first statement and tell participants that if they think it is true they should go stand by person 1 and if they think it is false they should stand by person 2. Once everyone has moved, get their attention and read the second statement. Again, tell them to move to "true" or "false" depending on whether they think the statement is true or false. Do not give them the answers; just let them see whether the group is divided or in agreement. Once you've finished the list, ask everyone to go back to their seats and tell them we will come back to this later in the session.

ALTERNATE ACTIVITY

Small group work: Myths and Realities (20 minutes). Another way to carry out this exercise is to break participants into small groups and to give them the myths and realities handout to discuss for 10 minutes. Then discuss the answers briefly in a full group. If you do small group work, it is suggested you do not confirm or clarify their answers, but only allow them to be discussed. Even if there is disagreement in the room, let the participants know we will come back to this later in the session.

Slide 6

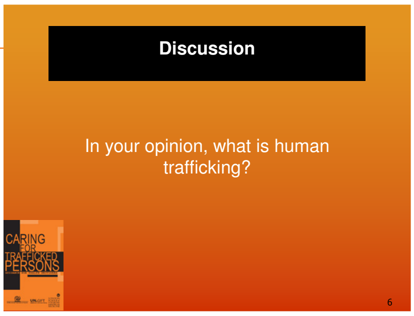

NOTE

Trafficking in persons is a complex phenomenon and many people may have their own impressions and ideas about it. This slide allows some participants to quickly voice their ideas.

Ask the full group what they think human trafficking is. If you like, you can write down some of what is said on a flip chart, but this is not necessary. Primarily this activity is useful to stimulate thought, discussion and to hear what people have to say, whether right or wrong.

IMPORTANT

Don't worry about misconceptions or comments that are not correct. At this stage, you want only to allow some ideas to be voiced. The rest of the presentation will help correct any misconceptions.

Slide 7

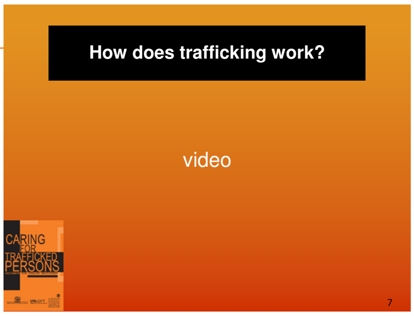

ACTIVITY

Video (10 minutes). Included on the CD are several short videos related to human trafficking. The purpose of this short video is to introduce the participants to the elements of human trafficking through a story.

CONSIDER

You may want to consider which video is most appropriate for your participants. The animated video from IOM Southern Africa (included on the CD that accompanies this guide) is a basic and easy-to-follow video which can be used in most settings. You may, however, want instead to show a video testimony of a survivor, or another human trafficking video, produced in the region or country where you are training. Check with your local counter-trafficking partners for examples from your region.

NOTE If it is not possible to show a video due to the technology available, find an alternative way to tell the story of a typical trafficking experience. Reading quotes from trafficked persons aloud (from published reports or other publicly available sources) can also help introduce the topic to your participants.

IMPORTANT Also be sure to protect confidential information about trafficked persons. If you adapt a story of a real victim from a non-public source, be sure to change the identifying details to protect their safety. For more information on safe management of trafficking in persons data, see the *IOM Data Protection Manual* available at http://publications.iom.int/bookstore/index.php?main_page=product_info&cPath=47&products_id=759.

Slide 8

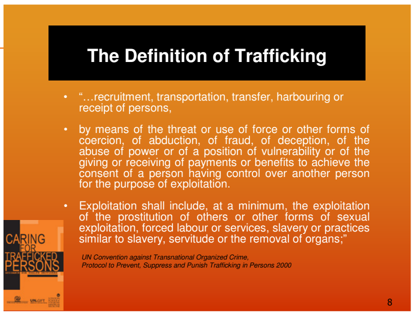

NOTE This slide is the first in a series about the definition of trafficking in persons, as defined by the UN Convention against Transnational Organized Crime and the UN Protocol to Prevent, Suppress and Punish Trafficking in Persons. The next few slides will help you explain this definition in more detail.

CONSIDER Some participants might want to study this definition more closely. Consider referring them to page 7 of the *Caring for Trafficked Persons* handbook so that they can continue to review the definition as you change slides. You can also provide them with the handout included in this *Facilitator's Guide*.

IMPORTANT While national legislation will differ, the definition from the UN Protocol is the global standard when defining human trafficking. In settings where there are differences, it is recommended to present the global definition first, even while noting that there may be differences within local legislation in a particular setting. It may also be useful to refer participants to concepts related to forced labour.

Slide 9

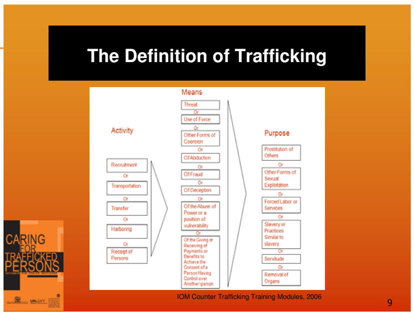

NOTE One way to understand the definition of trafficking in persons is to break it down into three elements that must be present in order for trafficking to have occurred, and to explain what these might include. This diagram, from an internal IOM training tool, is one visual way to help participants understand the key elements of the definition.

IMPORTANT

Be sure to emphasize that only one element from *each* of three categories needs to be present.

CONSIDER

It can be useful to refer to examples, especially if they have already been brought up in discussions. For example, you could refer back to the video which was shown earlier in the session, to point out specific elements. Or if participants have shared stories, you could use elements from those stories as you explain this slide. There will also be further examples in the next few slides.

REMEMBER

Health providers do not need to be specialists in understanding the definition of human trafficking or the laws and legal implications. Providers will not be responsible for determining whether someone is a victim of trafficking, but rather for providing care in appropriate and safe ways. You might want to remind your participants that we will be discussing referral and support systems for victims of trafficking and the specific role of health providers later in the training.

Slide 10

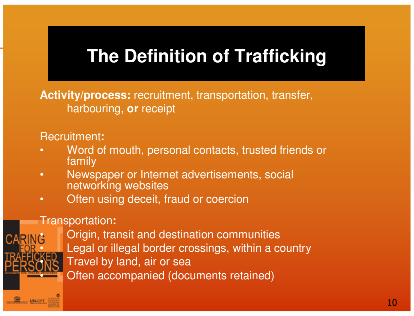

NOTE

This slide is useful in giving more detailed examples of how the "activity" element of human trafficking may take place.

CONSIDER

If possible, use examples of newspaper advertisements that seem "too good to be true" to share with participants. These are fairly easy to find in many contexts, and can generate interesting discussions.

Slide 11

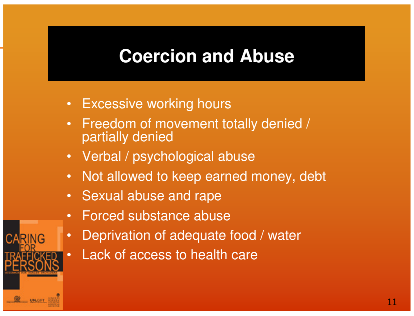

NOTE

The coercion and abuse that often accompanies trafficking in persons is an important element to emphasize, given the potential health consequences. In the next session, participants will discuss in more depth the health consequences of trafficking in persons. At this stage it is important for them to begin to understand what a trafficked person might experience in a trafficking situation.

Slide 12

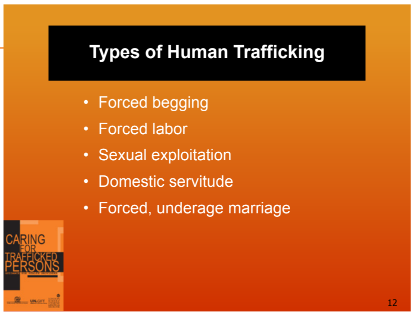

NOTE

Human trafficking is often associated only with sexual exploitation and forced prostitution. This slide reminds participants that trafficking may take place in a range of ways, and that the forms of exploitations are diverse, including labour exploitation, sexual exploitation and a range of other forms of exploitation.

Slide 13

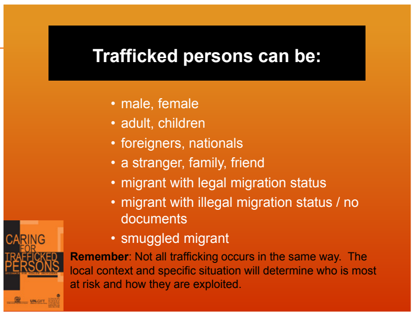

NOTE

This slide reminds participants that human trafficking can happen to anyone, regardless of the person's specific profile.

CONSIDER

Traffickers look for people they can exploit, such as migrants looking to improve their lives, or workers in a particularly unregulated sector.

Slide 14

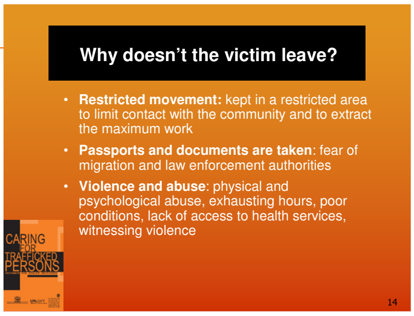

NOTE

This slide again emphasizes the control and coercion that are often part of the "means" element of human trafficking (the middle category in slide 9).

IMPORTANT Be aware that many participants will imagine situations where victims are locked in rooms or chained to sewing machines. While this does happen, in many cases, physical and especially psychological abuse and violence are used by traffickers to control victims. One example seen in many cases assisted by IOM is when violence is witnessed by the group. By harming or even killing one person in front of the others, the rest of the group will effectively be controlled through fear. It is important to note that for victims of trafficking, the threat of violence is very real. Other factors which may affect the ability of a trafficked person to leave include debt, unfamiliarity with the local language, fear of authorities, and not knowing where they are and how to get home.

Slide 15

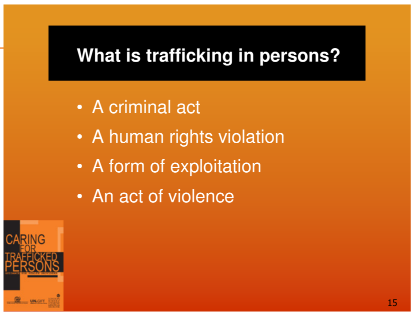

NOTE This slide summarizes the session, and the definition of trafficking in persons.

REMEMBER For health providers, the violence and exploitation inherent in the trafficking experience are important, as they result in potential health consequences.

Slide 16

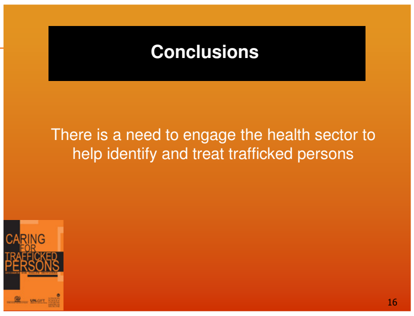

NOTE Based on the content of the session, it is clear that someone who has suffered a trafficking experience will need support from health providers to recover. In our next session we will begin to explore health consequences in more depth.

Slide 17

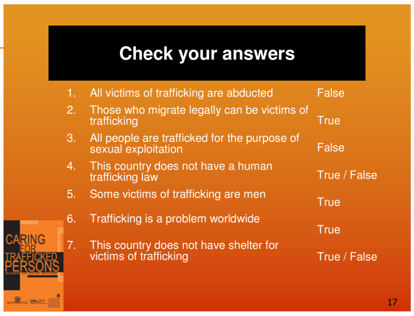

ACTIVITY

Poll / Quiz: Myths and Realities – Answers. As a wrap-up of the session, show participants the statements and the answers.

IMPORTANT

Questions 4 and 7 will depend on the context. You should know the answers to these questions before the session. Keep in mind that in some cases the answer may be a mix of true and false. For example, a shelter may exist, but it may not allow men and boys.

CONSIDER

If it has been impossible to locate this information before the session, participants in the room may be able to answer questions 4 and 7 for each other, depending on their experience in the topic. Always keep this in mind and allow participants the space to add information to the training based on their experience.

Slide 18

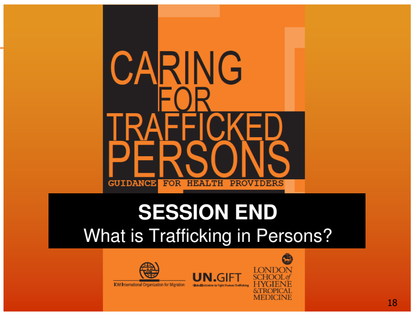

NOTE

This slide ends Session 1: What is Trafficking in Persons?

If you are following the suggested timeline, Session 1 will have been completed during the morning of the first day of training, and a lunch break should take place before Session 2.

SEE PAGE 16

Core Training Overview.

Session 1 Handouts

The following handouts are included for Session 1:

- Preliminary Agenda for participants
- Myths and realities handout for small group work
- Definition of Trafficking according to the international standard established in the Palermo Protocol

Capacity-Building for Health Providers on *Caring for Trafficked Persons*

Preliminary Agenda

**Day One**	
9:00 – 9:30	Arrival of Participants and Registration
9:30 – 10:00	Opening Remarks
10:00 – 10:30	What is Human Trafficking?
10:30 – 11:00	*Coffee Break*
11:00 – 12:30	What is Human Trafficking? Continued
12:30 – 13:30	*Lunch*
13:30 – 15:00	Health Consequences
15:00 – 15:30	*Coffee Break*
15:30 – 16:30	Trauma-informed Care
16:30 – 17:00	Reflection and Wrap Up

**Day Two**	
9:00 – 9:30	Arrival of Participants and Welcome Back
9:30 – 10:30	Role of the Health Provider
10:30 – 11:00	*Coffee Break*
11:00 – 12:30	Role of the Health Provider Continued
12:30 – 13:30	*Lunch*
13:30 – 14:00	Guiding Principles
14:00 – 15:00	Local Context and Next Steps
15:00 – 15:30	*Coffee Break*
15:30 – 16:30	Conclusions and Wrap up

QUIZ: True / False

In your small group, discuss the following statements and whether you feel they are true or false. Be prepared to share your answers with the full group after 10 minutes.

	True	False
1. All victims of trafficking are abducted.		
2. Those who migrate legally can be victims of trafficking.		
3. All people are trafficked for the purpose of sexual exploitation.		
4. This country does not have a human trafficking law.		
5. Some victims of trafficking are men.		
6. Trafficking is a problem worldwide.		
7. This country does not have shelters for victims of trafficking.		

PROTOCOL TO PREVENT, SUPPRESS AND PUNISH TRAFFICKING IN PERSONS, ESPECIALLY WOMEN AND CHILDREN, SUPPLEMENTING THE UNITED NATIONS CONVENTION AGAINST TRANSNATIONAL ORGANIZED CRIME

United Nations 2000

Excerpt from the full Protocol which is available online at http://www2.ohchr.org/english/law/protocoltraffic.htm:

Article 3
Use of terms

For the purposes of this Protocol:

(a) "Trafficking in persons" shall mean the recruitment, transportation, transfer, harbouring or receipt of persons, by means of the threat or use of force or other forms of coercion, of abduction, of fraud, of deception, of the abuse of power or of a position of vulnerability or of the giving or receiving of payments or benefits to achieve the consent of a person having control over another person, for the purpose of exploitation. Exploitation shall include, at a minimum, the exploitation of the prostitution of others or other forms of sexual exploitation, forced labour or services, slavery or practices similar to slavery, servitude or the removal of organs;

(b The consent of a victim of trafficking in persons to the intended exploitation set forth in subparagraph (a) of this article shall be irrelevant where any of the means set forth in subparagraph (a) have been used;

(c) The recruitment, transportation, transfer, harbouring or receipt of a child for the purpose of exploitation shall be considered "trafficking in persons" even if this does not involve any of the means set forth in subparagraph (a) of this article;

(d) "Child" shall mean any person under eighteen years of age.

Session Guide 2: Health Consequences

Session 2: TIMETABLE OVERVIEW

This session is estimated to take 1 ½ to 2 hours, depending on which activities are included. It is recommended that participants complete Session 1 prior to this session. This session is recommended for the afternoon of the first day of the Core Training.

SEE PAGE 16 Core Training Overview.

Session 2: LEARNING OBJECTIVE

By the end of this session, participants will be able to:

- Identify some of the major health consequences of trafficking.

CONSIDER

If appropriate, consider showing another short video or public service announcement on trafficking in persons at the start of the session. This can help focus participants after lunch.

IMPORTANT

Be sure to adjust the suggested agenda as needed, including start and end times and breaks, *before* the training (see Part 1).

PREPARATION & MATERIALS REQUIRED

- Be sure to have copies of the external agenda and other handouts.
- If you don't have access to PowerPoint, a computer, and a projector in the training room prepare flip charts in advance of the training with the slide content.
- Materials you need include flip charts and markers, as well as something to stick the paper to the walls.

IMPORTANT

It is essential that you are aware of the local counter-trafficking context *before* this session (see Part 1). For this session it is particularly useful to have information about local forms of exploitation, and if possible, examples from local service providers on the types of health problems they have seen among victims.

Slide 1

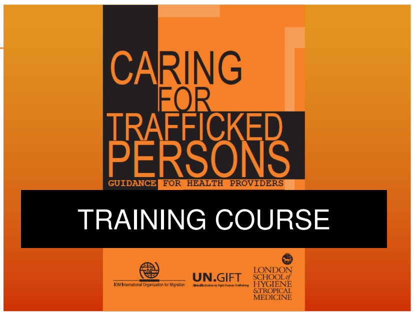

CONSIDER

This slide can be up on the screen as participants arrive after lunch, and during any announcements or remarks, if appropriate.

Slide 2

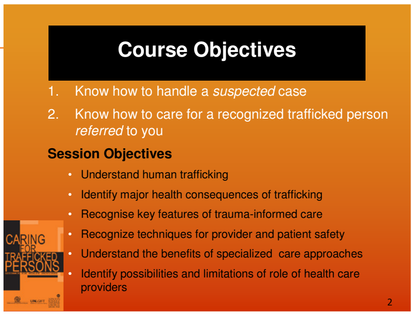

NOTE

This slide appears at the beginning of every session, to remind participants of the main objectives of the course: 1) know how to handle a suspected case and 2) know how to care for a recognized trafficked person. It is useful to remind participants of the overall structure of the course and session objectives.

IMPORTANT

Emphasize the two situations that a health provider might find themselves in related to trafficking in persons:

1. They may *suspect* someone is a victim of trafficking.
2. They may be caring for someone who is referred to them and is already *identified* as a trafficked person.

The guidance in this training and in the *Caring for Trafficked Persons* handbook is useful for both situations.

Slide 3

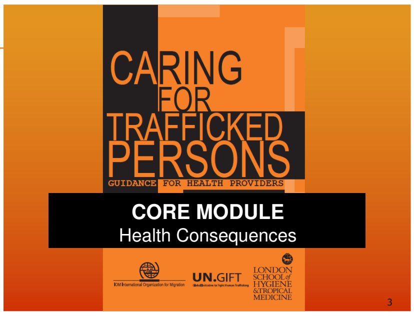

NOTE

This slide begins Session 2: Health Consequences

This is a good moment to remind participants that we are now going to focus more specifically on the health risks and consequences of human trafficking.

REMEMBER

Your participants will engage with the training if it is relevant to them as health providers. Remind them why the training is taking place and that we are all working on this topic because human trafficking hurts people, so they need health care.

Slide 4

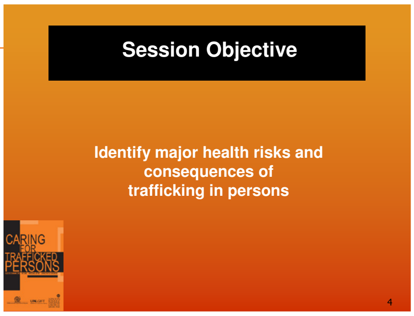

LEARNING OBJECTIVES

By the end of this session, you will be able to identify major health risks and consequences of trafficking in persons.

This learning objective is the focus of the current session.

IMPORTANT

Remind participants that you are talking about health as defined by the World Health Organization, "physical, social and mental well-being".

Slide 5

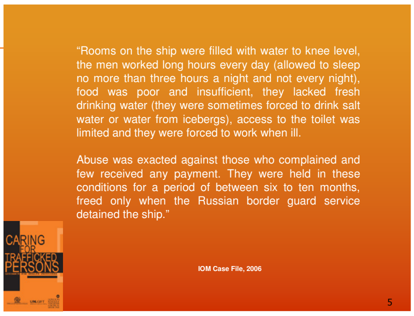

NOTE

The next few slides include stories of real human trafficking cases, to help illustrate the kinds of health risks trafficked persons are exposed to.

NOTE

See publications on trafficking in persons by IOM and by the LSHTM for more examples of stories (available online at www.lshtm.ac.uk/genderviolence see REPORTS).

ACTIVITY

Identifying health risks (during presentation). As you go through slides 5-8, ask participants to identify health risks in the stories. This can be done as a full group.

ALTERNATE ACTIVITY

Identifying health risks (20 minutes). If you feel it is appropriate to spend more time on this, you can have participants pair off or form small groups and do this exercise together. In this case, give each small group one of the stories listed in slides 5-8 and have them identify a list of health consequences. Then allow time for each group to present their case and the health risks they have identified to the full group.

REMEMBER

If you do the alternate activity you should estimate a longer time for this session.

CONSIDER

These case studies are included to illustrate a range of trafficking situations with different forms of exploitation and potentially different health risks. If possible, adapt the presentation or the activity to include examples from the local context. These may be available in country or local research reports, for example. Alternatively, you might be able to get case studies from local service providers or organizations like IOM. Be sure to carefully protect the anonymity of victims if you base the stories on real cases, by changing some of the details and not including any identifiable or personal information about real trafficked persons. If examples include identifying details, such as photos or names, do not include these in the training.

Slide 6

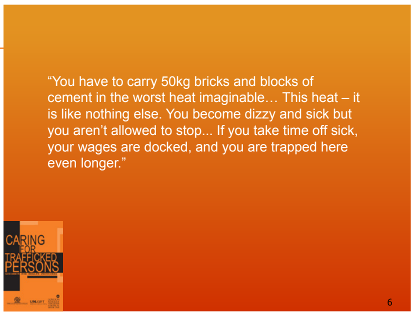

NOTE

See instructions on slide 5.

Slide 7

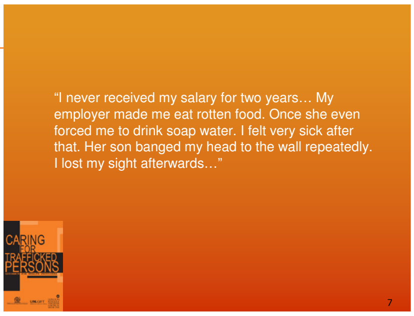

NOTE

See instructions on slide 5.

Slide 8

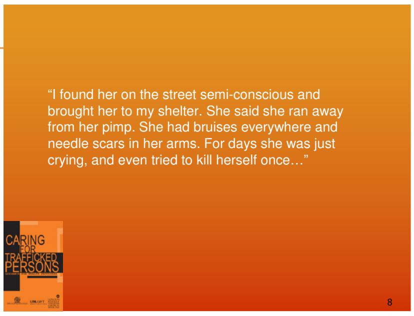

NOTE

See instructions on slide 5.

NOTE

In this example the quote is from a service provider.

Slide 9

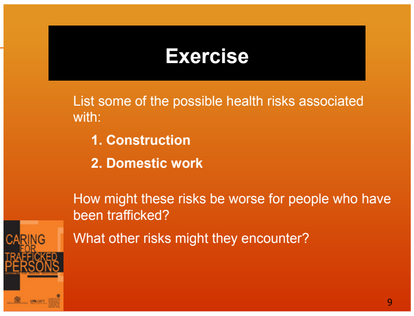

NOTE

This slide aims to help participants think about why trafficked persons may have specific health needs and require specialized approaches. During the first part of the discussion, participants should identify typical health risks faced by anyone working in that sector. In the second part of the discussion, they should begin to identify problems faced by trafficked persons that may cause them to face additional and increased health risks. These can include things such as extremely long hours / inability to stop working, little rest or sleep, extremely poor conditions, no protective equipment, poor training or training that is not in their language, accidents and abuse without any medical treatment, etc.

ACTIVITY

Health risks for trafficked persons (15 minutes). Ask participants what health risks they would associate with working in the sectors listed on the slide (e.g. construction). Ask participants to take a few minutes to list health risks, and then take some time in the full group to explore their answers. You can note these on a flip chart if you like. Then ask participants how the risks might be worse or different for people who have been trafficked (full group discussion).

CONSIDER

If useful during the second part of the discussion, you can flip back to the cases in slides 5-8.

IMPORTANT

Don't worry about being comprehensive – this slide is only to get participants thinking about how the health risks for trafficked persons may be different than for other patients. The next slide will allow them to work on this more comprehensively.

Slide 10

Health risks	Potential consequences
Physical abuse, deprivation	
Threats, intimidation, abuse	
Sexual abuse	
Substance misuse	
Social restrictions , manipulation and emotional abuse	
Economic exploitation	
Legal insecurity	
Occupational hazard	
Marginalization	

NOTE

This slide now begins to move from health risks to health consequences. Often, some consequences will have already been mentioned in discussion of risks.

ACTIVITY

Health risks and consequences (10 minutes). Viewing the table of health risks and potential consequences, working together in a full group, ask participants to call out potential consequences for each risk listed in the chart. You can repeat these back to them without filling out the chart, as this is in the handbook or can be given as a handout at the end of the activity. See handout included at the end of this Session.

ALTERNATE ACTIVITY

Health risks and consequences (20 minutes). Break the participants into small groups and give each group a copy of the Health Risks and Consequences handout. Tell them they have 10 minutes to fill out the potential consequences and then each group will be asked to provide some of the answers. After 10 minutes, bring the full group back together and have each group provide some of the answers. Once the activity is finished, either provide the participants with the summary of health risks and consequences handout, or direct them to their handbooks.

IMPORTANT

Be sure to ask each group for only some of the answers. This will save time, but also keep the activity from being repetitious. After you've completed the exercise, you can ask the full group if they have anything to add to what the other groups said (if time permits).

Slide 11

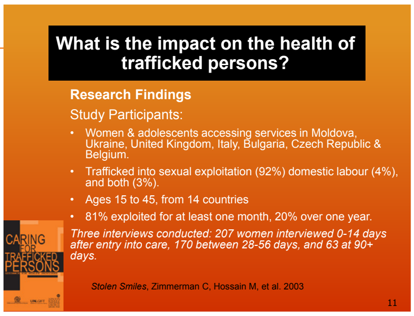

NOTE

This slide and the next few slides introduce the information that is available in peer-reviewed research about the health impact of trafficking in persons.

IMPORTANT

It is important to note that despite the importance of trafficking in many countries in the past 10 years, there is very little information from peer-reviewed research on the health impact of this crime. A recent systematic review (Oram, et. Al, 2012) found only 19 studies on health and trafficking that met their criteria, and they were all focused only on women and girls in sexual exploitation (see www.plosmedicine.org/article/info%3Adoi%2F10.1371%2Fjournal.pmed.1001224). Despite the need for additional research (especially on labour exploitation), some important information has been documented about the health impacts of trafficking in persons.

NOTE

Tell participants that you will be drawing on findings from one study in particular to illustrate common health patterns among trafficked persons. Remind them that while this study only focused on women and girls in sexual exploitation in Europe, the information is still useful for health providers to understand the impact on the health on trafficked persons regardless of the form of exploitation.

IMPORTANT

One of the reasons this study is particularly interesting, is that it included interviews at three different points in time after the women and girls were out of the trafficking situation, and this revealed some interesting information about the changes of symptom patters over time, which we will look at more closely in this session.

CONSIDER

If appropriate, you can provide participants with more details about the methodology of the study. Two hundred and seven women were included in the study. All study participants were receiving post-trafficking services. The study aimed to interview women three times after their contact with post-trafficking services: within 14 days, 28–56 days, and at 90 or more days, in order to understand how their health needs might have changed over time.

Slide 12

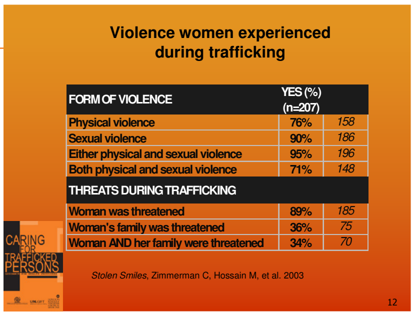

NOTE

This slide shows how often women in the study experienced violence during the trafficking situation. In addition this shows that trafficked persons face very real threats of violence and for this reason may not try to escape a trafficking situation. This also implies that their physical and psychological health is seriously in danger when they are in trafficking situations, and great caution should be taken when approaching someone who may still be in a trafficking situation, to avoid causing greater harm.

CONSIDER

Some quotes from this study are included in the training materials, but if you feel it is appropriate, you can also share more information from the *Stolen Smiles* study, available online (see http://genderviolence.lshtm.ac.uk/files/Stolen-Smiles-Summary.pdf).

CONSIDER

At the time of the publication of this training package, several studies on health and trafficking for labour exploitation were underway by IOM and LSHTM. It is highly recommended that you search for any new peer-reviewed research available when you prepare for the training, to complement the information here.

Slide 13

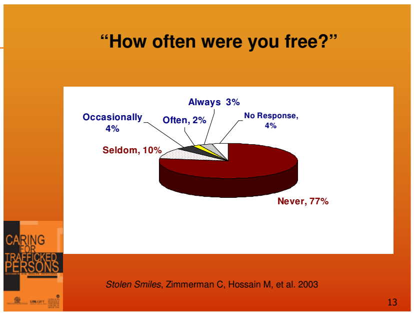

NOTE

This slide shows the results of the question "How often were you free to do as you wished or go where you wanted to go?". The coercion and control present in many trafficking situations translates for trafficked persons into an inability to control who does what to their bodies, and when, if at all, they are able to seek help for the pain, injuries and infections inflicted. This in turn has health consequences.

REMEMBER

Studies have shown that even when trafficked for other forms of exploitation (e.g. domestic servitude), sexual violence is often used to control trafficked women.

Slide 14

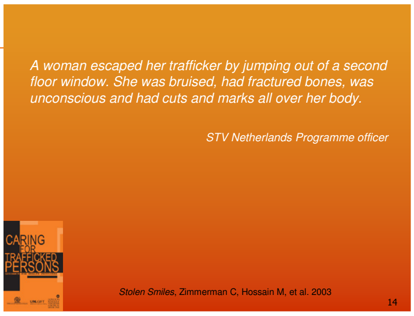

NOTE

This is a quote from a participant in the study, a real case. Remind participants that this is not unusual and that unfortunately, health providers in some settings come into contact with trafficked persons in hospital emergency rooms after they've jumped from windows to escape their situations.

Slide 15

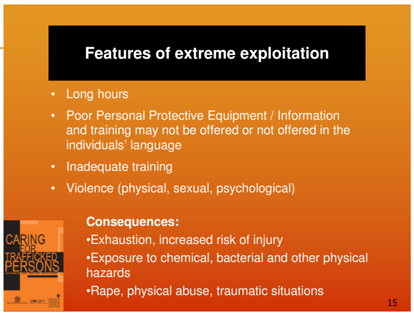

NOTE

This slide reminds participants of the kinds of health risks and consequences faced by many trafficked persons.

CONSIDER

Referring back to cases mentioned earlier in the training.

Slide 16

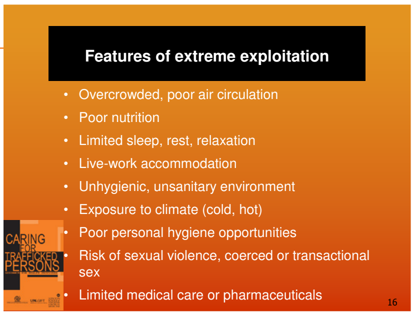

NOTE

This slide reminds participants of the kinds of health risks and consequences faced by many trafficked persons.

CONSIDER

Referring back to cases mentioned earlier in the training.

Slide 17

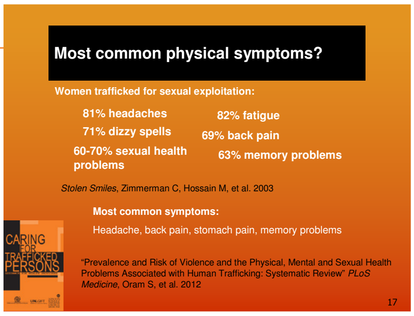

Slide 18

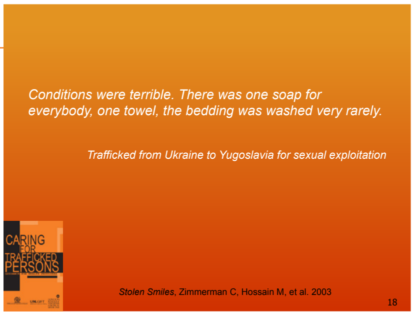

NOTE

This quote highlights that very often people are in daily living conditions, as well as working conditions, that are difficult, dangerous or stressful. This quote is from a participant in the study, a real case.

ACTIVITY

Most common physical symptoms (during presentation). As a full group, ask participants to guess the most common physical symptoms reported by trafficked persons. Then make them appear on the slide.

NOTE

A systematic review of health problems (Oram, et al., 2012) found very similar symptoms among trafficked persons, validating the earlier study. However the literature is focused on sexual exploitation, and more research is needed on labour exploitation.

Slide 19

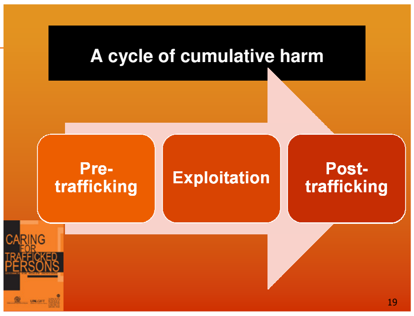

NOTE

This slide introduces the importance of thinking about the health profile of the trafficked person in a holistic way, taking into account their experiences before being trafficked, during the exploitation and post-trafficking. The next two slides will discuss experiences before trafficking in particular.

IMPORTANT

The impact of trafficking can be long-term. For example, some trafficked persons use drugs or alcohol during their exploitation, either to cope or because they are forced to do so. This can lead to long-term problems with addiction.

Slide 20

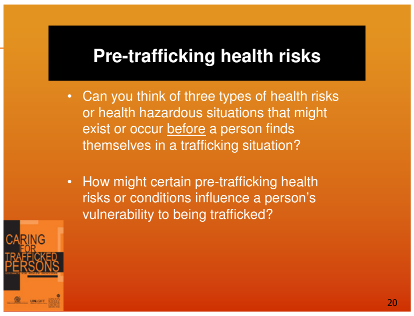

ACTIVITY

Pre-trafficking health risks (during presentation). As a full group, ask participants to think of possible health risks faced by trafficked persons *before* they were in the trafficking situation. Examples may include chronic health conditions (e.g. diabetes, high blood pressure), previous sexual abuse or harm, health problems linked to poverty, pre-existing psychological problems, etc.)

Slide 21

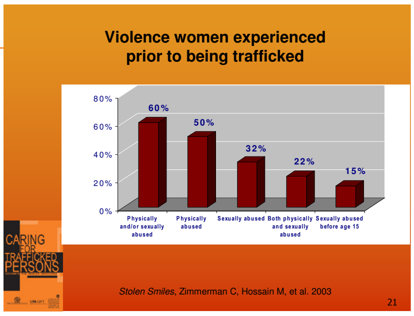

NOTE

This slide shows high levels of violence experienced by women prior to being trafficked, based on findings in the *Stolen Smiles* study. Health conditions and experiences prior to trafficking can increase the vulnerability of a person to being trafficked. Another example is disability, where people are targeted by traffickers because of their disability (e.g. for forced begging).

CONSIDER

If there are violence specialists in the room (e.g. domestic violence or gender-based violence), they may be able to provide additional comments about how previous violence can create vulnerability to future violence.

Slide 22

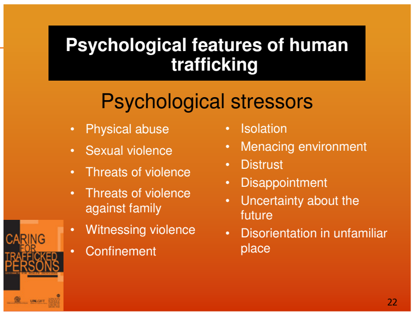

NOTE

The next few slides are related to the psychological impacts of trafficking. Tell participants that we have looked at some physical risks and impacts, and now we will look a bit more at psychological risks and impacts.

ACTIVITY

Psychological stressors (during presentation). As a full group, ask participants what elements of a trafficking experience might affect a victim's mental health? Then make the bullets appear to share some examples.

Slide 23

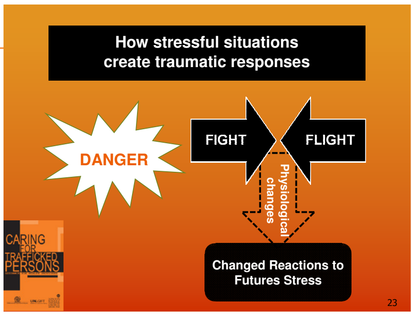

NOTE

It is important to keep in mind that traumatic experiences during a trafficking situation often influence the way people will react to stress in the future. This slide shows the "typical" response of anyone to danger –fight or flight. A trafficked person has this reaction over and over again during their experience, and this can lead to physiological changes that cause them to have this extreme reaction to minor stressors in the future. So they can seem agitated or over-reactive. Or, in some cases, the opposite can also occur: people may have become numbed to stressful events and have "turned off" their biological alarm and safety mechanisms, so they may be less able to perceive danger and protect themselves. A health provider needs to keep this in mind when interacting with a trafficked person.

CONSIDER

You can ask participants what they might feel if a fire cracker suddenly exploded in the room, or a wall suddenly burst into flames. The intense adrenaline reaction that everyone would have is a good example of the fight or flight response. Imagine having that same response to small, everyday occurrences.

Slide 24

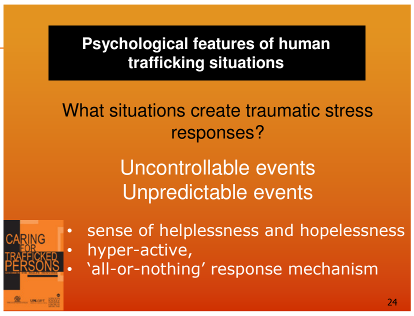

NOTE

Studies of trafficked persons and research on torture victims show that they have similar experiences psychologically. These experiences are uncontrollable and unpredictable. How 'unpredictable' and 'uncontrollable' appear to be the two main factors that cause very poor mental health after a traumatic situation has ended. That is, people in both trafficking and torture situations are likely to have strong post-trauma reactions because they had very little ability to control or predict what was happening to them while they were in the trafficking situation. They are also unable to find relief from dangers and stress while in trafficking situations, such as you might be able to find ways to reduce your stress in your work or in your home. Remember that control and coercion are common means used by traffickers, and that many trafficked persons do not feel "free" to do what they want at any time. Imagine not being able to control when you rest, when you eat, or when you interact with family and friends. Trafficked persons may be threatened or abused or told to do something by their traffickers at any moment of the day or night and have little ability to predict when this situation might end (unpredictable). The combination of these factors has many negative mental health consequences, including the reactions shown on this slide.

Slide 25

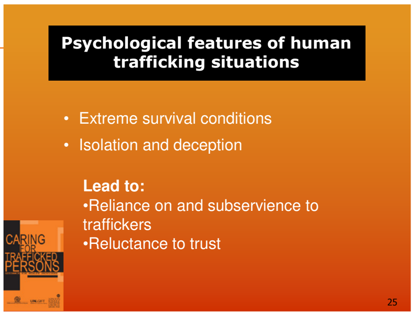

NOTE

This slide shows that in addition to the uncontrollability and unpredictability of the trafficking situation, extreme survival conditions (e.g. you do not receive food unless the trafficker decides to give it to you, you cannot keep warm enough, clean enough), isolation and deception all lead to reliance and subservience to traffickers and a reluctance to trust.

REMEMBER

Health providers will relate to the content of the training if it is relevant to them. Ask them how this might affect the way they interact with their patient. Ask them whether a patient might act differently with their provider on the first visit versus the seventh (e.g., trust-building, providing clear information, giving options and control over decisions). Trafficked persons have learned how to survive, but some of these survival mechanisms can impact the interaction they have with a health provider.

Slide 26

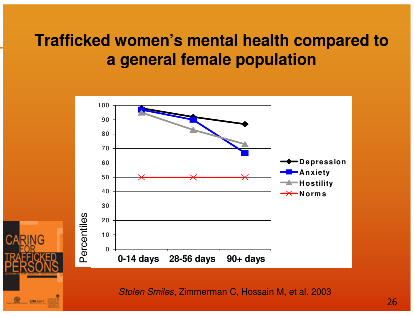

NOTE

This slide compares the symptom levels of depression, anxiety and hostility of trafficked women in the Stolen Smiles study to an average female population. You can see that all three symptoms improve over time while in contact with support services. However, the red line shows the "norm" (from a sample of US women). So, you can see that while symptoms appear to improve, the trafficked persons still have levels of depression, anxiety and hostility much higher than the population norms – even after 90 or more days in contact with support services. It is also notable that depression symptoms reduce the least, suggesting that depression is likely to be a longer-lasting mental health problem.

Slide 27

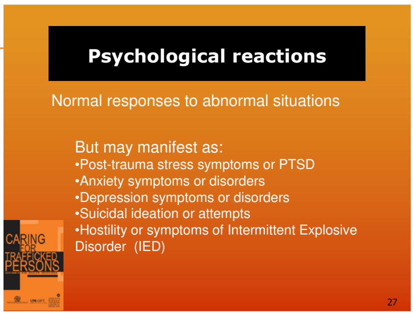

NOTE

This slide reminds participants that the psychological responses that trafficked persons have are *normal* responses to what they have experienced. However, health providers should be aware that these normal responses can manifest themselves as a range of health problems.

REMEMBER

Attitudes and opinions about mental health may vary dramatically from country to country. It is important to emphasize that a trafficking situation is not a normal, everyday occurrence, and it is very appropriate and normal to have these reactions. Many of these reactions comprise our biological survival tactics. Later in the training we'll talk more about how to care for someone who has been through these experiences.

Slide 28

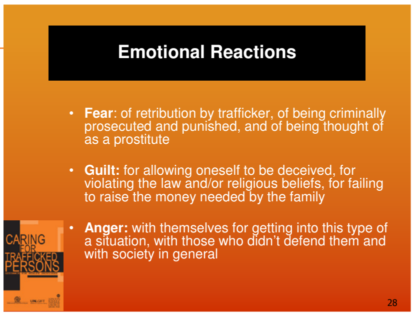

NOTE

The next two slides give examples of the kinds of emotional reactions that a trafficked person may have.

Slide 29

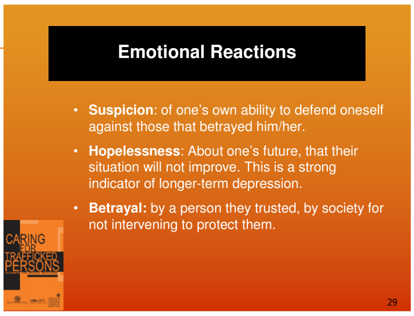

Slide 30

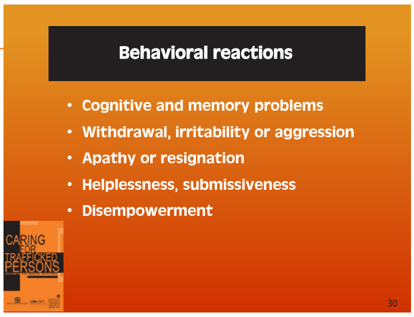

NOTE The next two slides give examples of the kinds of behavioural reactions that a trafficked person may have.

Slide 31

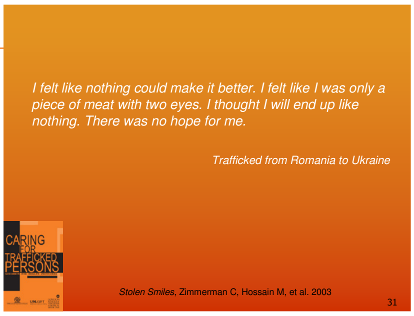

NOTE This is a quote from a participant in the study, a real case.

Slide 32

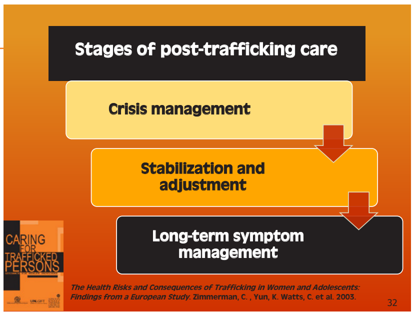

NOTE

We've discussed how some symptoms can persist over time. These are the phases of post-trafficking care. Health providers are involved in all of these stages.

Slide 33

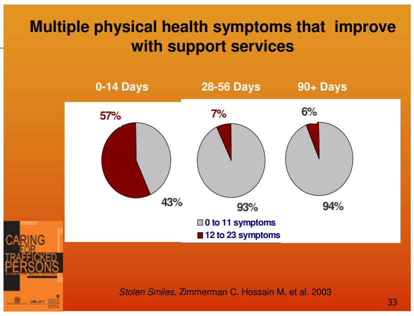

NOTE

This slide shows the changes in physical health symptoms over time (in the *Stolen Smiles* study). You can see how poorly a trafficked person might be feeling when they first get out of a trafficking situation and the importance of providing immediate health care. For health care practitioners, this suggests the complexity of symptoms and the diagnostic challenges in trying to interpret this range of symptoms.

Slide 34

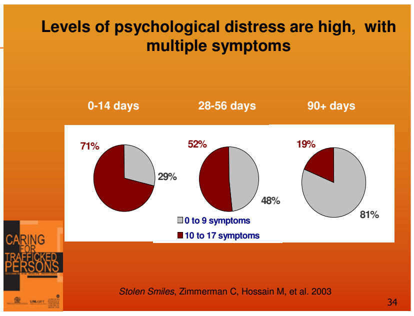

NOTE

This slide shows the changes in psychological health symptoms over time (from the study). There is still improvement over time, but much less quickly than with physical symptoms. It is not until 90 or more days that a real reduction of symptoms is seen.

CONSIDER

If appropriate, discuss with participants the implications of slides 34 and 35, both for health providers but also for other actors. A good example is police, who often try to interview victims immediately after they come out of a trafficking situation. Imagine being interviewed with this level of pain and discomfort. In the United Kingdom, the results of this study were used to help police consider when they would undertake full interviews to allow victims time to receive support and health care and begin to feel better.

Slide 35

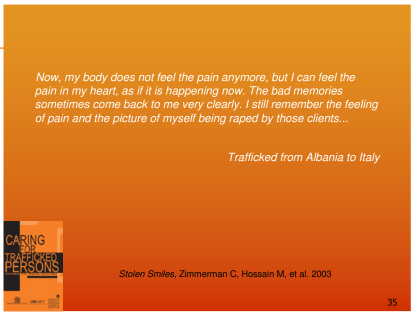

NOTE

This is a quote from a participant in the study, a real case.

Slide 36

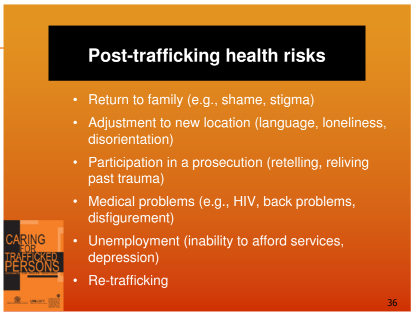

NOTE

So we discussed the possible violence and other health risks that may exist before a person is trafficked, and we discussed the impacts of the trafficking itself on their health. Now let's look at post-trafficking health risks.

ACTIVITY

Post-trafficking health risks (during presentation). As a full group, ask participants what health risks might exist once a person is out of the trafficking situation. Then make the bullets appear to share examples.

REMEMBER

Some health consequences will be life-long. Imagine the case of a domestic worker who set out to improve her life and fell into a trafficking situation, and from the repetitive work or jumping from a multistory window to escape, now has lifelong back problems or disability. Or imagine a trafficked person who becomes infected with HIV and must learn to live with this chronic disease.

Slide 37

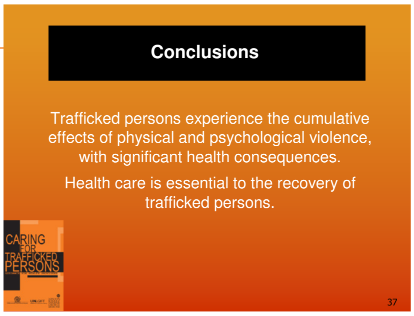

NOTE

Based on the content of the session, it is clear that trafficked persons experience the cumulative effects of physical and psychological risks and abuse (including before, during, and after the actual trafficking experience) that are likely to have acute and longer-term health consequences. Health care is essential to their recovery. In the next session we will begin to explore specialized care approaches for trafficked persons.

Slide 38

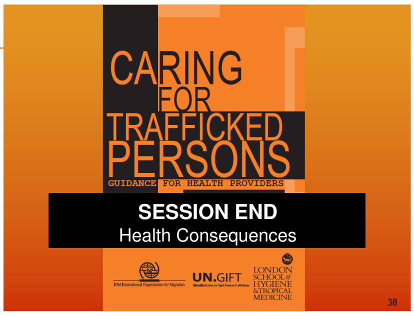

NOTE

This slide ends Session 2: Health Consequences.

If you are following the suggested timeline, this session will have been completed during the afternoon of the first day.

SEE PAGE 16

Core Training Overview.

Session 2 Handouts

The following handouts are included for Session 2:

- Health risks and consequences handout
- Summary of health risks and consequences handout

HEALTH RISKS & CONSEQUENCES OF BEING TRAFFICKED

Health risks	Potential consequences
Physical abuse, deprivation	
Threats, intimidation, abuse	
Sexual abuse	
Substance misuse<br>Drugs (legal and illegal), alcohol	
Social restrictions and manipulation & emotional abuse	
Economic exploitation<br>Debt bondage, deceptive accounting	
Legal insecurity<br>Forced illegal activities, confiscation of documents	
Occupational hazards<br>Dangerous working conditions, poor training or equipment , exposure to chemical, bacterial or physical dangers	
Marginalization<br>Structural and social barriers, including isolation, discrimination, linguistic and cultural barriers, difficult logistics, for example: transport systems, administrative procedures.	

SUMMARY OF THE HEALTH RISKS & CONSEQUENCES OF BEING TRAFFICKED[7]

Health risks	Potential consequences
**Physical abuse, deprivation**	Physical health problems, including death, contusions, cuts, burns, broken bones
**Threats, intimidation, abuse**	Mental health problems including suicidal ideation and attempts, depression, anxiety, hostility, flashbacks and re-experiencing symptoms
**Sexual abuse**	Sexually transmitted infections (including HIV), pelvic inflammatory disease, infertility, vaginal fistula, unwanted pregnancy, unsafe abortion, poor reproductive health
**Substance misuse**<br>Drugs (legal & illegal), alcohol	Overdose, drug or alcohol addiction
**Social restrictions & manipulation & emotional abuse**	Psychological distress, inability to access care
**Economic exploitation**<br>Debt bondage, deceptive accounting	Insufficient food or liquid, climate control, poor hygiene, risk-taking to repay debts, insufficient funds to pay for care
**Legal insecurity**<br>Forced illegal activities, confiscation of documents	Restriction from or hesitancy to access services resulting in deterioration of health and exacerbation of conditions
**Occupational hazards**<br>Dangerous working conditions, poor training or equipment, exposure to chemical, bacterial or physical dangers	Dehydration, physical injury, bacterial infections, heat or cold overexposure, cut or amputated limbs
**Marginalization**<br>Structural and social barriers, including isolation, discrimination, linguistic and cultural barriers, difficult logistics, e.g. transport systems, administrative procedures	Unattended injuries or infections, debilitating conditions, psycho-social health problems

7 IOM, UN.GIFT and London School of Hygiene and Tropical Medicine, *Caring for Trafficked Persons: Guidance for Health Providers*. Geneva, 2009. pp. 17.

Session Guide 3: Trauma-informed Care

Session 3: TIMETABLE OVERVIEW

This session is estimated to take 1 hour, but can be longer if additional activities are included. It is recommended that participants complete Sessions 1 and 2 prior to this session. This session is recommended as the final session on the afternoon of the first day of the Core Training.

SEE PAGE 16 Core Training Overview.

Session 3: LEARNING OBJECTIVES

By the end of this session, participants will be able to:

- Recognize key features of trauma-informed care
- Recognize techniques for provider and patient safety
- Understand the benefits of specialized care approaches for trafficked persons

IMPORTANT Be sure to adjust the suggested agenda, including start and end times and breaks, before the training (see Part 1).

PREPARATION & MATERIALS REQUIRED

- Be sure to have copies of the external agenda and other handouts.
- If you don't have access to PowerPoint, a computer, and a projector in the training room prepare flip charts in advance of the training with the slide content.
- Materials you need include flip charts and markers, as well as something to stick the paper to the walls.

IMPORTANT It is essential that you are aware of the local counter-trafficking context before this session (see Part 1). For this session it is particularly useful to have information about the local health system, such as local requirements for forensic exams (i.e. who carries these out in this context) and local reporting requirements for health providers (i.e. when are they required by law to report something to the authorities).

CONSIDER Having health providers with experience caring for trafficked persons in the room can be helpful for this session. If this is not possible, health providers who work routinely with victims of sexual violence or domestic violence can also contribute greatly to the conversation when discussing issues such as cooperation with law enforcement, forensic exams, informed consent and reporting requirements.

What is trauma-informed care? Trauma-informed care is an approach used by health providers through which providers explicitly acknowledge that violence may have occurred and may be affecting the patient's physical and psychological symptoms and how the provider will offer care. It suggests that the provider should be informed about, and sensitive to, trauma-related issues present in violence survivors.

REMEMBER

"Trauma-informed care" is an emerging concept that may be new to many of your participants. Keep in mind that those who work with victims of violence (whether trafficking in persons or other forms of violence, such as domestic violence or rape) will likely relate to the content based on their own experience.

Slide 1

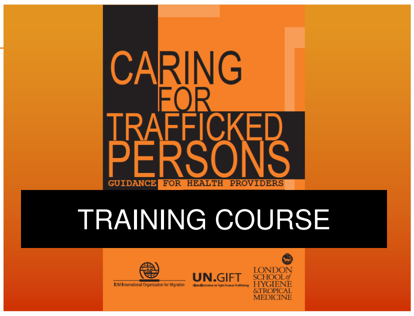

CONSIDER

This slide can be up on the screen as participants return from a coffee break or if there are changes in facilitators between this session and the previous session.

Slide 2

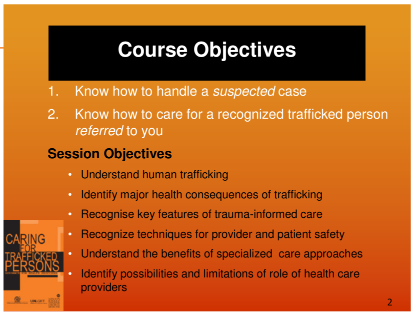

NOTE

This slide appears at the beginning of every session, to remind participants of the main objectives of the course: 1) know how to handle a suspected case and 2) know how to care for a recognized trafficked person. It is useful to remind participants of the overall structure of the course, and session objectives.

IMPORTANT

Emphasize the two situations that a health provider might find themselves in related to trafficking in persons:

1. They may *suspect* someone is a victim of trafficking.
2. They may be caring for someone who is referred to them and is already *identified* as a trafficked person.

The guidance in this training and in the *Caring for Trafficked Persons* handbook is useful for both situations.

Slide 3

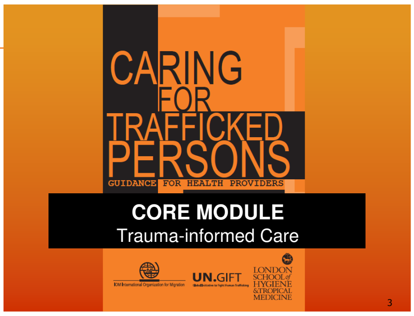

NOTE

This slide begins Session 3: Trauma-informed Care.

This is a good moment to remind participants that we are now going to begin to focus on how to interact with trafficked persons in specialized ways.

Slide 4

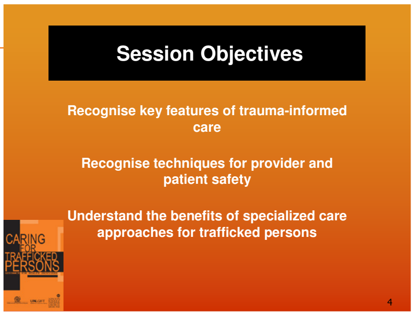

LEARNING OBJECTIVES

By the end of this session, you will be able to:

- Recognize key features of trauma-informed care
- Recognize techniques for provider and patient safety
- Understand the benefits of specialized care approaches for trafficked persons

These learning objectives are the focus of the current session and the rest of day one of the training.

Slide 5

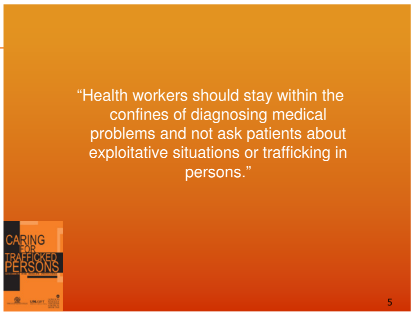

NOTE

This slide begins to explore the role of the health provider, particularly as related to violence. The idea of this slide is first to elicit reactions from the health providers about their own feelings in response to the statement, and second, to emphasize that violence has health consequences so health providers need to find ways to talk about it as part of their job. Trauma-informed care is based on the concept that service providers recognize the effects of violence on survivors and respond to people's special care needs.

ACTIVITY

Role of the health provider (during presentation). As a full group, ask participants what they think of this statement. Is it true? False?

IMPORTANT

There is no wrong answer to this statement. If health providers agree with the statement, you as the facilitator can respond by being empathetic as to how difficult it may be in their setting to talk about these sorts of topics. But gently make the point that because of the health consequences of violence (such as trafficking), it is important to acknowledge what they have experienced and consider how these experiences might affect their health and the health providers' approach to care.

Slide 6

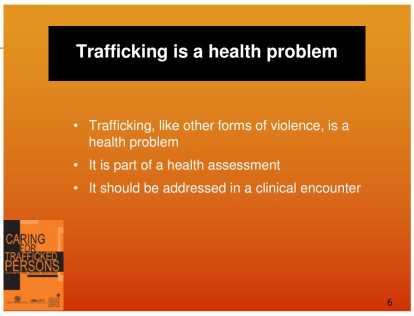

NOTE

This slide reinforces the message from the previous slide, that trafficking has health implications and should be addressed in a clinical encounter. Just as we ask about other health issues (e.g. do you smoke?) we need to ask about experiences that may be affecting their health. The way we do this is key.

IMPORTANT

Though in some trafficking cases the health risks are extreme, the symptoms are not always obvious, especially if the health provider sees the trafficked person after the signs of physical injuries have disappeared. Remind participants that even if they don't see signs of trauma (physical or psychological) that doesn't mean people are not suffering post-trauma reactions. Think back to the lists of symptoms we looked at in Session 2.

Slide 7

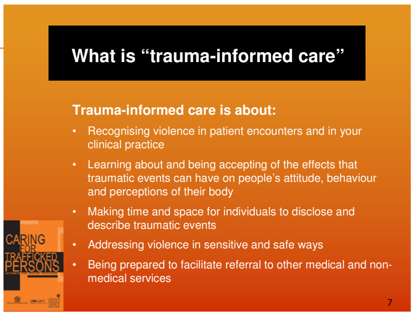

NOTE

This slide introduces the concept and definition of trauma-informed care.

CONSIDER

You might want to remind participants of the information in Session 2, which showed why people who have been trafficked might act distant, untrusting, or hostile. It is a natural reaction for a health provider to have an emotional response when a patient acts in these ways. Part of a trauma-informed approach is recognizing that these actions are related to what the person has experienced.

CONSIDER

You may also want to tell participants that they will need to be prepared when working with trafficked persons to ask about and to hear about violence and highly stressful and emotional experiences. Hearing about violence also impacts the health provider, so it is important to try to be prepared.

Slide 8

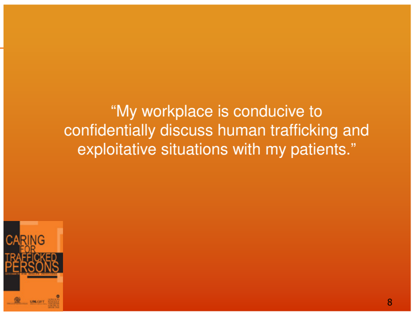

NOTE

This slide begins to explore the importance of creating a "clinical safe space" to trauma-informed care. Creating a "clinical safe space" means ensuring that your patient feels not only physically safe, but also adopting an approach that makes your patient feel at ease to disclose difficult subjects, talk about current health problems, and that he or she knows that the information disclosed will be kept confidential. Creating a clinical safe space will be discussed later in the session.

ACTIVITY

Clinical safe space (during presentation). As a full group, ask participants what they think of this statement. Ask them how many agree. If someone is in agreement, ask them to explain why / how the workplace is conducive.

Slide 9

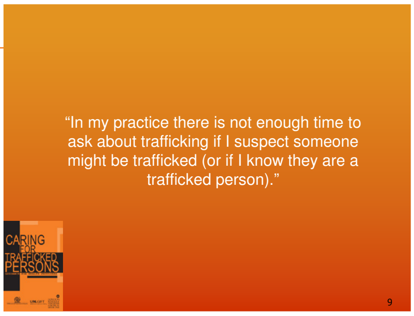

NOTE

This slide recognizes that many health providers already feel overwhelmed and lack time for their jobs, even without trying to implement specialized approaches for certain patients.

ACTIVITY

Constraints (during presentation). As a full group, ask participants what they think of this statement. Ask them how many agree.

IMPORTANT

This a very real challenge faced in many settings, and not something that a health care provider can necessarily change. However, you can suggest that there are some small things that can be done *in the time they have*, to recognize the experience of the trafficked person.

Slide 10

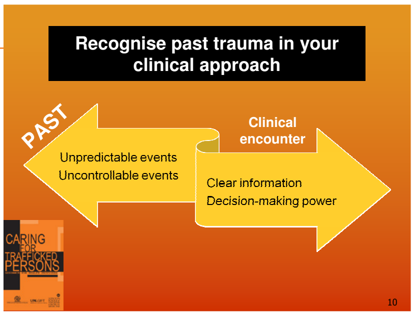

NOTE

This slide builds on the information in the previous session when you discussed the similarity between the psychological reactions of trafficked persons and those of torture victims. Anything that can be done during the clinical encounter to remove "unpredictability" and "uncontrollability" your patient feels will go a long way to reducing his or her stress and to beginning their move towards independence and a sense of safety. Two clear ways to do this during a clinical encounter are by: 1) providing clear information; and 2) empowering trafficked persons to make decisions about their own care.

ACTIVITY

Predictability and Controllability (during presentation). As a full group, ask participants what could be done to make a clinical encounter more predictable and to help the person feel they are not helpless and out of control. One example is to explain everything step-by-step, before and as it happens.

Slide 11

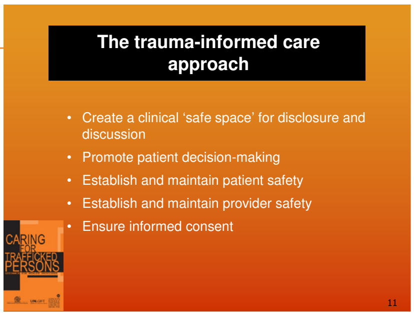

NOTE

This slide breaks the specialized approach into five clear areas, which are explained in more detail in the next few slides. The purpose of this slide is to just introduce these five areas, without going into detail.

CONSIDER

Slides 13-16 each contain elements of trauma-informed care, and all contain a great deal of information. Several activities are suggested as alternatives for how to present these slides. Be aware of the dynamics of your group and the time you have available. Some possibilities include:

- Presenting all four slides in a full group (fastest alternative)
- Breaking into small groups for all four slides (longest alternative)
- Some mix of the above

Keep in mind that during the second day of the training, participants can practice some of these skills during a role-play activity.

Slide 12

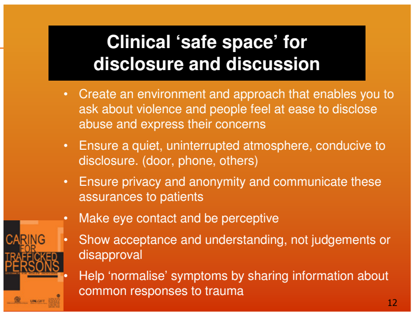

NOTE

This slide contains elements of how to create a clinical safe space (psychologically and physically safe) space for disclosure and discussion.

ACTIVITY

Clinical safe space (during presentation). As a full group, ask participants what they think a "clinical safe space" may be. Be sure to tell them that safety is both psychological and physical. After a short discussion, make the bullets appear.

ALTERNATE ACTIVITY

Clinical safe space (20 minutes). Divide participants into small groups and ask each to brainstorm examples of what might help make a "clinical safe space". Be sure to tell them that safety is both psychological and physical. After 10 minutes, ask each group to share their ideas. Then make the bullets appear.

CONSIDER

To save time if doing the alternative activity, ask each group to only report back on things that weren't already shared by other groups.

NOTE

You may want to remind participants that people who have experienced trafficking have often been tricked, deceived and abused by people they trusted, people who may have offered to help them. They have very real reasons not to trust you. Remind participants of the "fight or flight" reaction that some patients continue to feel even when not in danger. The health provider may know that the setting is safe or that the room is private. But it is important to communicate this clearly to the trafficked person (i.e. "you are safe here and no one will come in this room while we are talking").

CONSIDER

Some health providers may be accustomed to hearing about violence or abuse, while others are not. Remind your participants that it is important not to be judgemental or moralistic towards their patient. Many trafficked persons already feel ashamed and responsible for the violence they experienced.

IMPORTANT

Remind your participants of the information from Session 2 about normal reactions to abnormal experiences. Providers can help put trafficked people at ease by telling patients who have been through a trafficking experience and are suffering a range of difficult symptoms that their reactions (e.g. inability to sleep, flashbacks, and fearfulness) are *normal* reactions to what they've been through.

Slide 13

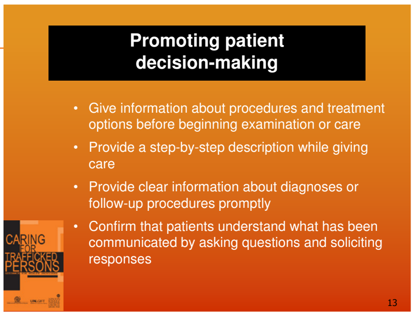

NOTE

This slide contains elements of how to promote and empower trafficked persons to participate in the decision-making about their care.

ACTIVITY

Decision-making (during presentation). As a full group, ask participants how they think patients could be encouraged to participate in decisions about their care. After a short discussion, make the bullets appear (two slides).

ALTERNATE ACTIVITY

Decision-making (20 minutes). Divide participants into small groups and ask each to brainstorm examples of what might help encourage patients to participate in decisions about their care. After 10 minutes, ask each group to share their ideas. Then make the bullets appear.

CONSIDER

To save time if doing the alternative activity, ask each group to only report back on things that weren't already shared by other groups.

NOTE

While many counselling approaches use "open-ended" questions that allow an individual to describe their experiences or feelings, this may not be a good approach for a trafficked person. When a clinician first meets with a person who has been trafficked, it is often the case that the person will be more at ease answering closed questions (questions with a "yes" or "no" answer) or with questions that have several clear choices. For most people, asking someone specific questions about their health is much more manageable than inquiring "so, tell me what happened."

Slide 14

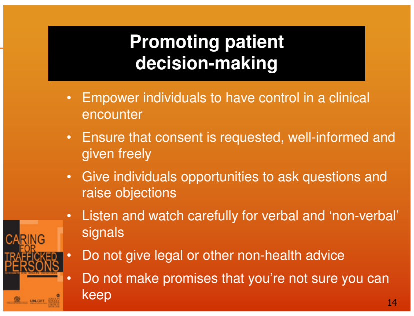

NOTE

This slide contains more elements of how to promote patient decision-making.

Slide 15

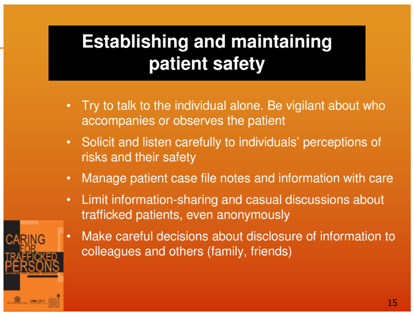

NOTE

This slide contains elements of how to establish and maintain patient safety.

ACTIVITY

Patient safety (during presentation). As a full group, ask participants how they think patient safety can be established and maintained. After a short discussion, make the bullets appear.

ALTERNATE ACTIVITY

Decision-making (20 minutes). Divide participants into small groups and ask each to brainstorm how to establish and maintain patient safety. After 10 minutes, ask each group to share their ideas. Then make the bullets appear.

CONSIDER

To save time if doing the alternative activity, ask each group to only report back on things that weren't already shared by other groups.

Slide 16

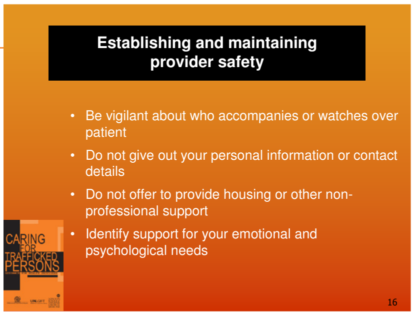

NOTE

This slide contains elements of how to establish and maintain *provider* safety.

ACTIVITY

Provider safety (during presentation). As a full group, ask participants how they think provider safety can be established and maintained. After a short discussion, make the bullets appear.

ALTERNATE ACTIVITY

Decision-making (20 minutes). Divide participants into small groups and ask each to brainstorm how to establish and maintain provider safety. After 10 minutes, ask each group to share their ideas. Then make the bullets appear.

CONSIDER

To save time if doing the alternative activity, ask each group to only report back on things that weren't already shared by other groups.

IMPORTANT

In some settings trafficking in persons is associated with organized crime, while in others with small groups of individuals. While in most situations a health provider will not be in danger by providing care for a trafficked person, it is important to emphasize that it is good practice to always assume danger is possible, and take measures to keep everyone safe. The purpose of these safety rules is not to worry health providers, but instead to remind them to take basic safety precautions. It may be useful to refer to domestic violence as a similar example – in most cases the abuser will not come after the health provider or the victim during a health exam, but by adopting certain measures, it is possible to ensure the safety of both the provider and the patient. Similarly, in caring for people who have been trafficked, it is always best to assume it is possible that a trafficker has accompanied the victim, is within the vicinity of the clinic or is looking for the victim and may be dangerous, even if that is not always the case. Remind health care providers that the most important thing they can do to protect themselves and their patient is to respect confidentiality.

Slide 17

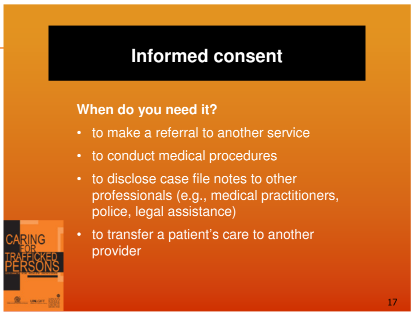

NOTE

This slide reminds participants of the importance of informed consent. While informed consent is a normal part of providing health services, it is especially important for trafficked persons (i.e. to help them feel safe, to involve them in decisions about their care, etc.).

ACTIVITY

Informed consent (during presentation). As a full group, ask participants when informed consent is necessary. After a short discussion, make the bullets appear.

Slide 18

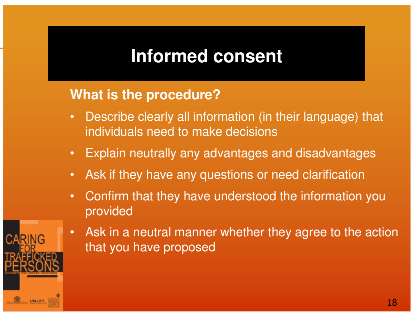

NOTE

This slide continues to discuss informed consent.

IMPORTANT

Remind participants that the “informed” part of informed consent is extremely important. It may be useful to remind participants that they themselves would like to have information about their condition, treatment options, etc. before making decision about or agreeing to care. The patient must understand what they are consenting to, why it is happening, and what will happen next.

CONSIDER

You may want to ask participants what situations or issues might impede a patient’s understanding. This could include language, culture, a disability, or education level, for example.

Slide 19

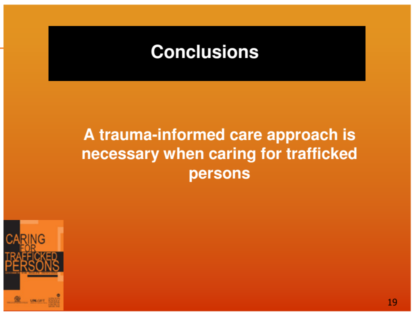

NOTE

Based on the content of the session, it is clear that a trauma-informed care approach is necessary when caring for trafficked persons. In day two of the training, we will begin to practice some of these skills.

Slide 20

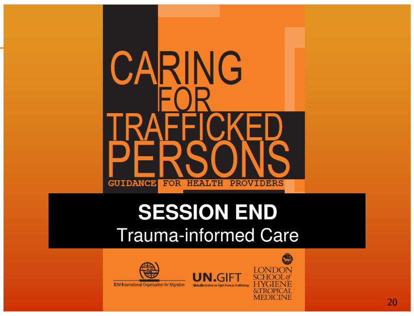

NOTE

This slide ends Session 3: Trauma-informed Care.

ACTIVITY

Reflection and wrap-up. As this is the final session in day one of the Core Training, it is recommended to undertake one final activity. Ask participants to think about the following questions and to write down their answers:

1. What one thing will you do differently in your work in the future based on today’s training?
2. What questions do you have based on today’s training?

Ask participants to save the answers and bring them back for day two of the training.

CONSIDER

It's usually a good idea to remind participants what time the training will start on day two.

If you are following the suggested timeline, this session will be the last session of the first day.

SEE PAGE 16

Core Training Overview.

Session Guide 4: Role of the Health Care Provider

Session 4: TIMETABLE OVERVIEW

This session is estimated to take 2 1/2 hours, including a one-hour presentation and an activity, though it can be longer depending on the activities included. It is recommended that participants complete Sessions 1, 2 and 3 prior to this session. This session is recommended as the first session on the second day of the Core Training.

SEE PAGE 16 Core Training Overview.

Session 4: LEARNING OBJECTIVES

By the end of this session, participants will be able to:

- Identify the possibilities and limitations of the role of the health care provider

IMPORTANT

Be sure to adjust the suggested agenda, including start and end times and breaks, *before* the training (see Part 1).

PREPARATION & MATERIALS REQUIRED

- Be sure to have copies of the external agenda and other handouts.
- If you don't have access to PowerPoint, a computer, and a projector in the training room prepare flip charts in advance of the training with the slide content.
- Materials you need include flip charts and markers, as well as something to stick the paper to the walls.

IMPORTANT

It is essential that you prepare yourself in particular for the role-play activity that is suggested as part of this session. Handouts and instructions must be clear in order for this activity to be successful (see below for more details).

CONSIDER

It is recommended to build extra time into your morning sessions, to allow for participants to arrive late. This will vary by context, but in general it is smart to have some extra time built into the planned agenda.

Slide 1

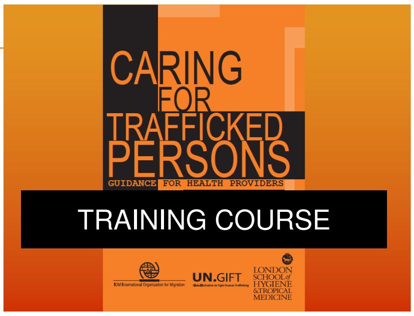

CONSIDER

This slide can be up on the screen as participants arrive in the morning and during any announcements at the start of day two.

ACTIVITY

Ice-breaker day two (15 minutes). Welcome participants back to the second day of training. Ask them to take out the answers to the "reflection" questions you asked at the end of the training yesterday:

1. What one thing will you do differently in your work based on the training so far?
2. What questions do you have based on the training so far?

You may want to consider having these questions written on a flip chart ahead of time. In a full group, ask if any participants would like to share their answers. After several people have shared, move into the content of the session.

CONSIDER

As participants share their questions, note which ones will be addressed during day two of the training, and which ones might be related to more details within the handbook. Some facilitators like to write questions and topics that are brought up by participants but that will *not* be addressed specifically in the training on a separate flip charts (sometimes called the "parking lot" as ideas are "parked" there). This can validate the person and show you are listening, but also sets a limit about what will be discussed further in the context of the specific training.

ALTERNATE ACTIVITY

Review of Expectations (10 minutes). As an introduction to day two, it can be useful to look back at the expectations generated on day one of the training. Point out things which have already been covered and others that will be addressed today.

NOTE

This is a good moment to provide a brief overview of the agenda for the day. Let participants know that today we will be practicing some of the ideas presented yesterday, and discussing the local context in more detail.

Slide 2

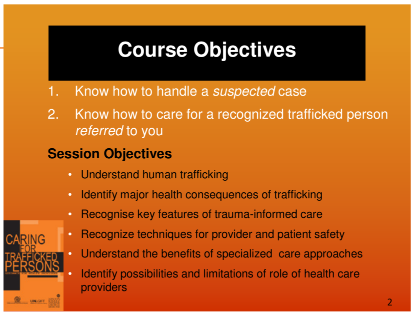

NOTE

This slide appears at the beginning of every session, to remind participants of the main objectives of the course: 1) know how to handle a suspected case and 2) know how to care for a recognized trafficked person. It is useful to remind participants of the overall structure of the course, and session objectives.

IMPORTANT

Emphasize the two situations that a health provider might find themselves in related to trafficking in persons:

1. They may suspect someone is a victim of trafficking.
2. They may be caring for someone who is referred to them and is already identified as a trafficked person.

The guidance in this training and in the *Caring for Trafficked Persons* handbook is useful for both situations.

Slide 3

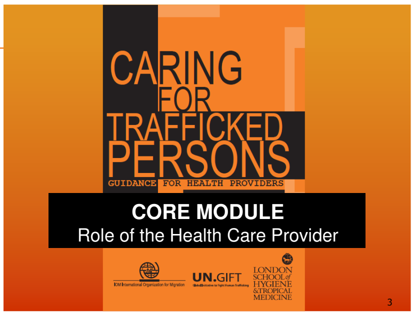

NOTE

This slide begins Session 4: Role of the Health Care Provider.

Slide 4

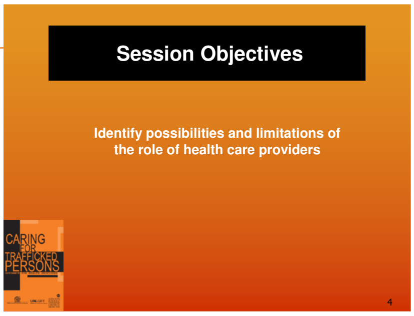

LEARNING OBJECTIVES

By the end of this session, you will be able to:

- Identify possibilities and limitations of the role of health care providers

This learning objective is the focus of the current session.

NOTE

Yesterday during Session 3, participants began to explore their role, during discussions about using the trauma-informed care approach. Today we will explore this a bit further, including some specific recommendations about what a health care provider should and should not do in caring for trafficked persons.

Slide 5

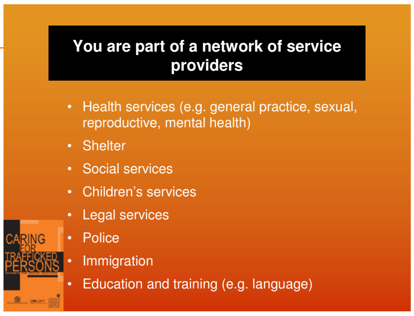

NOTE

This slide is to remind participants that they are part of a broad network of service providers. Trafficked persons have a range of needs, and health care is only one. Health providers should remember they are part of a broader network and cannot meet the needs of a trafficked person alone.

ACTIVITY

Referral Network Introduction (during presentation). As a full group, ask participants what types of needs a trafficked person might have, in addition to health needs. Following a short brain-storming, make the bullets appear and discuss briefly.

Slide 6

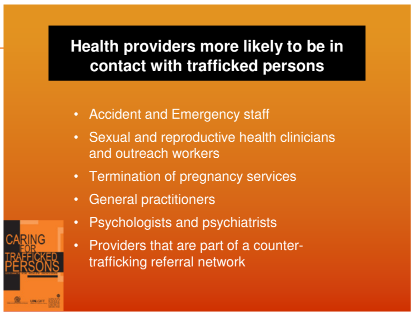

NOTE

This slide explains that some health providers are more likely to come into contact with trafficked persons than others. These include health providers that handle emergencies and violence response, health providers that work with vulnerable populations, and health providers who are already part of existing counter-trafficking referral networks. However, any health care provider could potentially come into contact with a trafficked person, and should be prepared.

IMPORTANT

Remind health care providers that in general, traffickers tend to isolate their victims and to limit their contact with others. However, if a health problem is interfering in the exploitation activity (i.e. making the trafficked person less able to work and therefore less profitable to the trafficker) they may bring a trafficked person to a health structure for care. Another scenario that is common is that a trafficker abandons a trafficked person because they are sick or injured, and then they are brought to health services.

CONSIDER

Health providers are therefore very important partners in the identification of possible victims of trafficking. If appropriate, mention that later in the day, we'll be looking more closely at the local context and who to call if you suspect someone is a victim of trafficking.

Slide 7

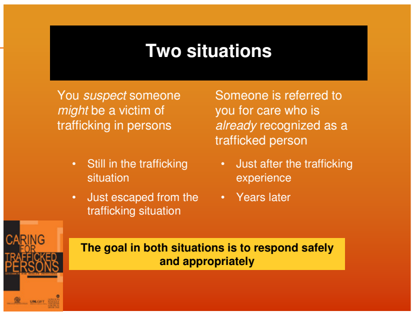

NOTE

The next few slides discuss what to do in the two situations we've been discussing throughout the training. Remind participants of these two situations, and that the goal is to respond safely and appropriately. Let them know we'll look first at a situation where the health care provider suspects someone might be a victim of trafficking.

Slide 8

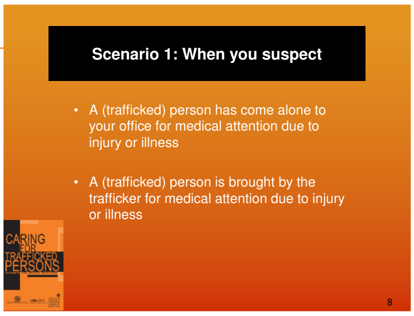

NOTE

These are two examples of situations where a health provider might become suspicious that their patient might be a victim of trafficking.

REMEMBER

The health provider would not know that the person accompanying the patient is a trafficker. But they might notice that something seems unusual or "abnormal" about the interaction.

IMPORTANT

Remind participants that it is not their job to make any legal determination about whether someone is a victim of trafficking or not. Someone else, such as a member of the local counter-trafficking coalition, makes that determination. What is important is that they recognize the red flags and know what to do if they suspect.

Slide 9

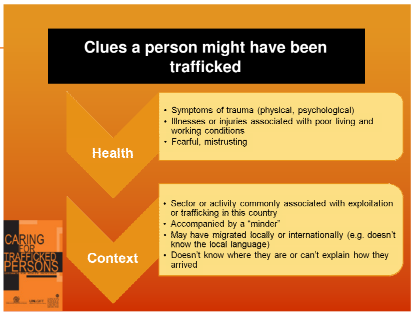

NOTE

This slide illustrates some clues that might indicate a possible trafficking situation. Again, this is not a training on how to do a formal screening and determination in cases of trafficking in persons. These are just some clues that might help formulate a viable suspicion.

IMPORTANT

Emphasize to the participants that every trafficking case is different. There is no single set of signs, no protocol that will enable health providers to detect possible trafficking situations in all cases.

Slide 10

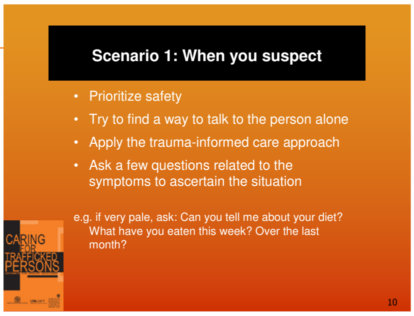

NOTE

This slide reviews what to do you when you *suspect* a patient might be a victim of trafficking. Even if you are not sure, it is always good to be very careful, and to err on the side of precaution. This means not doing anything that can put the person in danger or cause them harm.

REMEMBER

In addition to applying a trauma-informed care approach and being very careful, focusing on health symptoms in a non-threatening way can help a health provider learn more about the situation of the person.

Slide 11

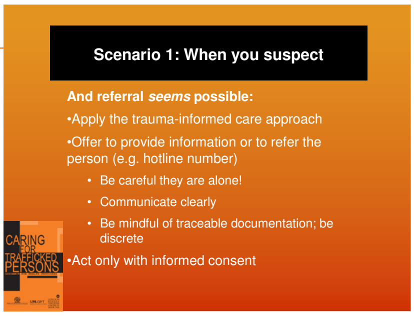

NOTE

There are two possible situations when you *suspect* a patient might be a victim of trafficking. The first is when a referral seems possible. In this case, the health provider can discretely offer to call someone *if the patient agrees*. It is important to have this information available and ready to use *before* this situation happens. Later today we'll be talking about this context.

IMPORTANT

Making a referral should always be done by applying informed consent procedures. That is, explaining the options to the possible victims and inquiring their preferred action – which may be to "do nothing for the moment". Victims of trafficking might still be in the situation. As an outsider, you cannot know what threats have been made, whether they have a child still with the trafficker or whether they have made other plans to leave the trafficking situation. Only the victim knows the range of dangers the situation may pose and what might make the circumstances better or worse. So a health provider should attempt to find safe ways to share information about possible options and get consent to make a referral – *before* taking any actions.

Slide 12

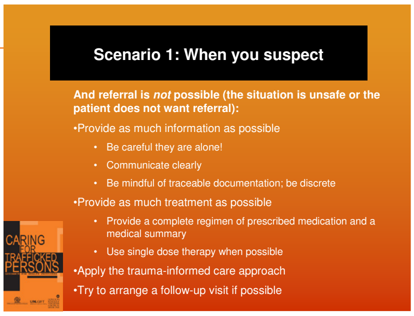

NOTE

The other situation when you *suspect* a patient might be a victim of trafficking is that referral does *not* seem possible. In this case, the health provider can try to make the most of the encounter by maximizing the information they share and maximizing the treatment they provide, and attempting to make a follow-up appointment to see the person again.

ACTIVITY

Referral not possible (during presentation). In a full group, ask participants for examples when referral might not be possible. Some examples include:

- When the patient does not give their consent
- When the patient is accompanied by a possible "minder"
- When the patient is going to be deported, or jailed

IMPORTANT

In this situation, any information shared with a possible trafficked person should be done *very discreetly*; otherwise it could put the person in further danger. Examples might include a small piece of paper with the hotline number on it, something that could be tucked away inside the clothes without being detected. In some countries such "bra cards" have been used to disseminate a hotline number to women who may have been trafficked. It is also not recommended that any personal information (e.g. name of the hospital or health provider) be included.

IMPORTANT

Remind health providers that it is not their job to *rescue* someone. Not only could this put them at risk, it could put the person they want to help at further risk.

Slide 13

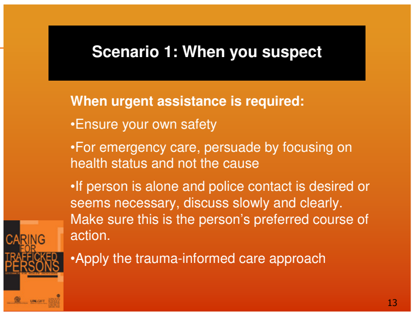

NOTE

In situations where urgent help is needed, health providers must ensure their own safety first. As a general rule, it is always important to try to get the consent of the person *before* calling the police. In non-emergency situations, and unless required by law, the police should not be called without the person's consent to this type of assistance.

IMPORTANT

Remind participants that no one can make a better risk assessment than the victims themselves. If a victim does not feel it is safe enough to call the police, it probably isn't. Think about domestic violence response, where often social services work to make sure the situation is stable enough to involve the police, before they do so.

Slide 14

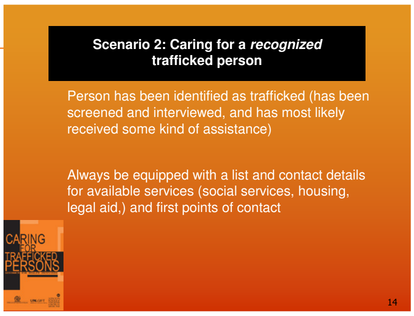

NOTE

The much more common situation is that a health provider will receive a patient that is no longer in the situation, who has been referred to them for care. This may be someone who just escaped the trafficking situation, or could be someone who years later is seeking help for something that happened in the past that is now affecting their health.

IMPORTANT

As in the previous situations, health care providers should have information about other services available *before* the patient arrives. Let participants know that later today we'll be discussing the local context in more detail.

Slide 15

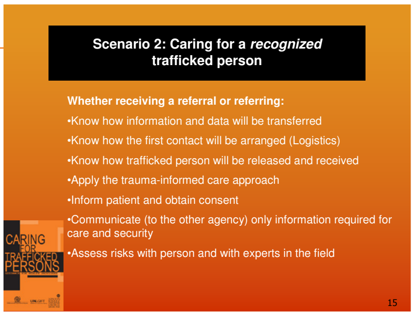

NOTE

This slide provides some basic information about referrals and trafficked persons. Whether the health provider is receiving the patient or referring them to another service provider, these steps can help ensure a more positive experience for the trafficked person.

IMPORTANT

Remind participants about the "uncontrollable" and "unpredictable" elements of trafficking that were discussed in a previous session. A referral that is handled badly can contribute to high levels of stress and make a trafficked person feel worse rather than better. On the other hand, a referral that is well-planned and carefully explained to the person in advance and throughout, can be empowering, encouraging and help contribute to the person's recovery.

Slide 16

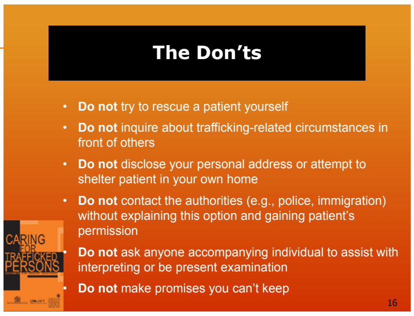

NOTE

This slide summarizes the things a health care provider should *not* do when caring for a trafficked person.

CONSIDER

Refer participants to the handbook, where more details are included on things to do and not to do.

Slide 17

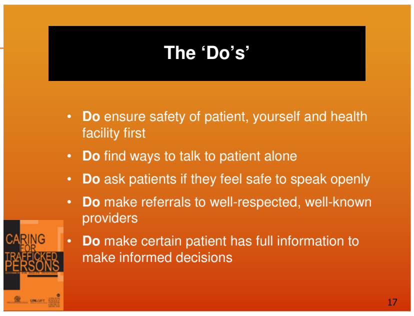

NOTE

This slide summarizes the things a health care provider *should* do when caring for a trafficked person.

NOTE

One way health providers can try to speak to the patient alone is to suggest that privacy is required for health reasons.

Slide 18

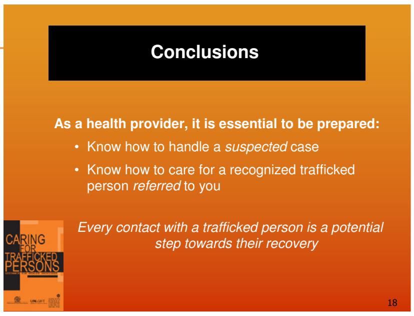

NOTE

Based on the content of this session, it is clear that health providers should know how to handle situations with trafficked persons (whether recognized or suspected), to contribute to their recovery.

CONSIDER

This session ends with a role-play activity, which is anticipated to take 1 ½ - 2 hours. It is likely a break will take place after this slide and before the activity. Depending on the timing, you may choose to explain the activity before the break.

Slide 19

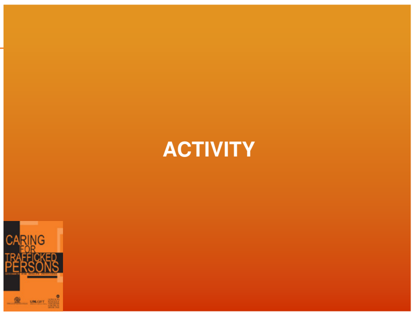

NOTE

An activity is suggested at the end of this session, to allow participants to practice some of the skills we've been discussing over the past two days. There are two ways to carry out this activity:

1. Simultaneous role plays among small groups
2. "Fish-bowl" role plays in front of the full group

It is suggested you carefully read the description and instructions for each (below) and determine which is most appropriate.

IMPORTANT

Be well prepared for this activity in advance. A well-planned role play can contribute greatly to a training.

SEE PAGE 99

Annex 1: General Training Preparations

SEE PAGE 75

Session 4: Handouts

CONSIDER

To complicate this role play and emphasize the issues related to communication and working with interpreters, consider casting a "victim" and "trafficker / minder" who speak another language than the group, so they can speak with each other but the health provider cannot understand. It can be helpful to add another role, an administrator or someone else in the health structure who speaks both languages and can assist the health provider to communicate.

ACTIVITY

Simultaneous Role play (1 ½ – 2 hours). Simultaneous role play allows several small groups to carry out the role play at the same time. The benefits of this method include:

- More participants are able to play an active role
- Participants may feel less intimidated in a small group
- Different groups may have different experiences, which can be shared in the full group discussion afterwards

It is suggested that you give instructions to the full group *before* you break them into their small groups and assign roles:

1. Explain to the full group how the role play will take place. Do *not* tell them any details about the roles, only that three people will act out a scenario while everyone else observes, and then we will discuss what happened.
2. Break the participants into small groups.
3. Assign roles in each group (every group needs to have someone play role 1, 2, 3, and all others will be role 4). Give people the handout that goes with their role.

IMPORTANT

Be sure to tell everyone to keep the details of their roles secret! The activity will not work if they start to look at each other's roles before the role play.

4. Speak separately with everyone who has role number 1, then role number 2, then 3, then 4. Explain to them their role and answer any questions they might have. Remind them not to discuss it with anyone when they go back to their group. Remind them they should try to "act" the role during the role play.

CONSIDER

If you have more than one facilitator, it is best to do step 4 simultaneously to help prevent the participants from sharing secret information about their roles.

5. Once everyone is back in their small groups, tell them to start the role play and that they have 20 minutes to act out the scenario.

IMPORTANT

It may take a few minutes for some of the groups to get started. It can help to give them each a couple of props, like chairs. Make sure each group is able to get started.

6. After 20 minutes, check to see if the groups are finished or if they need more time. When possible tell the groups to stop the role play and to spend 20 minutes discussing the activity. In particular, observers should share their observations.

CONSIDER

You can try to structure the discussion in the small groups (e.g. 10 minutes comments from observers, 10 minutes discussion by the actors themselves, but in most cases the discussion will happen naturally.

7. Finally, hold a full group discussion about the activity for 20 minutes.

CONSIDER

One way to structure this full group discussion is to first ask the "victims" how they felt, what comments they have, and then ask the "traffickers / minders" and then the "health care providers". This can be useful to get the discussion going.

IMPORTANT

The full group discussion is both to allow the participants to share their emotional reactions to the activity and to begin to reflect on the challenges and benefits of applying a "trauma-informed care" approach. Often in role plays the participants become very involved and have lots to say after the activity. When you can, illustrate points from the training that came up in the role play.

ACTIVITY

"Fish-bowl" Role play (1 ½ - 2 hours). "Fish-bowl" role play allows the full group to watch the role play take place and then discuss it together. The benefits of this method include:

- The facilitator is able to see the entire role play and then can use elements of what happened in the full group discussion afterwards.
- Some participants enjoy the intensity of acting out a role in front of the full group.
- It can take less time if only one group acts out the role play.

1. Explain to the full group how the role play will take place. Do not tell them any details about the roles, only that three people will act out a scenario while everyone else observes, and then we will discuss what happened.
2. Assign the 3 key roles. Give these people the handout that goes with their role.

CONSIDER

As the success of the fish-bowl role play depends very much on the participants chosen to act it out, some facilitators like to approach participants they think might be willing to play a role before the activity, to ask them in private. This can happen during a coffee break, for example.

IMPORTANT

Be sure to tell the "actors" to keep the details of their roles secret! The activity will not work if they start to look at each other's roles before the role play.

3. Speak separately with each person, away from the others. Explain to them their role and answer any questions they might have. Remind them not to discuss it with anyone when they go back to the group. Remind them they should try to "act" the role during the role play.

CONSIDER

If you have more than one facilitator, it is best to do step 3 simultaneously to help prevent the actors from sharing secret information about their roles on accident.

4. Tell them to start the role play and that they have 20 minutes to act out the scenario.

CONSIDER

It can help to set the scene for a fish-bowl role play, such as setting up an area with chairs for the doctor's office, for example. Keep in mind that all participants need to be able to hear and to see the role play acted out.

5. After 20 minutes, decide whether they need more time. Once complete, stop the role play.

6. Hold a full-group discussion about the activity for 20 minutes.

CONSIDER

You can try to structure the discussion by asking first for feedback from the actors themselves, and then from the full-group. In most cases the discussion will happen naturally.

IMPORTANT

The full group discussion is both to allow the participants to share their emotional reactions to the activity and to begin to reflect on how difficult it was to try to apply a "trauma-informed care" approach. Often in role plays the participants become very involved and have lots to say after the activity. When you can, illustrate points from the training that came up in the role play.

CONSIDER

Depending on the time available, you might consider holding two separate fish-bowl role plays. The diversity of the actors can make this interesting for the group.

Slide 20

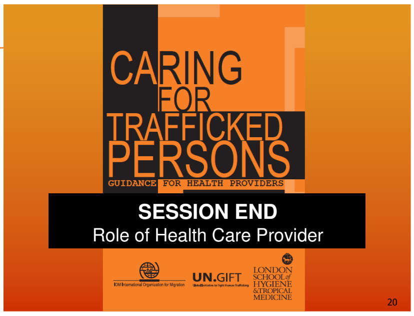

NOTE

This slide ends Session 4: Role of the Health Care Provider.

If you are following the suggested timeline, this session ends the morning of the second day. In some cases, depending on the time allotted the role play, it may extend into the afternoon session.

SEE PAGE 16

Core Training Overview.

Session 4 Handouts

The following handouts are included for Session 4:

- Role Number One: Victim
- Role Number Two: Health Care Provider
- Role Number Three: Trafficker / Minder
- All Other Participants: Observer

ROLE NUMBER ONE: VICTIM

Instructions: Carefully read the description of your role below. Do not share the details of your character with anyone else (only you know the details). Try to put yourself in the place of the person and do what they would do.

You are a 19 year-old woman, who has been a victim of trafficking for the past nine months. You are currently forced to work in a bar in Country X, and in addition to cleaning and cooking you are sexually exploited. Four weeks ago you began to get a red rash and sores on your hands. They brought you a cream that you pretended to use but really threw away. Your hands are worse, and since you can't cook and some of your "clients" are disgusted by your hands, the boss has decided to take you to a health clinic. She has made it clear that if you say anything you shouldn't or do anything she doesn't like while at the clinic, she will order your family killed. You are feeling desperate and don't think you can survive much longer.

You arrive at the clinic after a journey, and you have no identification. The boss pretends she is your family, and she fills out all the papers at the reception desk. When the doctor calls you the boss goes with you and she sits beside you. You keep quiet.

If the health worker is able to speak to you alone, without the boss, you will try to explain, very carefully, your situation. Even if the boss is not there you will be careful, since you are not sure that you can trust the health worker and you do not want to make your situation worse.

If the boss never leaves, you will eventually cooperate with the health worker in front of the boss, but very, very carefully. You might try to find ways to explain the situation or to drop hints to the health worker in a way that the boss doesn't understand what you are doing.

Either way, you hope they keep you at the clinic and send the boss away. You are very careful because you know she could have your family killed.

ROLE NUMBER TWO: HEALTH CARE WORKER

Instructions: Carefully read the description of your role below. Do not share the details of your character with anyone else (only you know the details). Try to put yourself in the place of the person and do what they would do.

You are a health care worker who works in a clinic. It has been a busy day, like usual, and there are many people waiting to be seen. You call for the next patient. A shy young woman, between 15-25 years old, walks in with an older family member who is also a business owner and seems very concerned with the health of the young woman.

The patient's hands are covered with blisters and a rash, and you begin to try to assess her health.

ROLE NUMBER THREE: TRAFFICKER / MINDER

Instructions: Carefully read the description of your role below. Do not share the details of your character with anyone else (only you know the details). Try to put yourself in the place of the person and do what they would do.

You are a 41-year old woman who is the boss of a network of traffickers working in Country X. You've had some complaints from clients about the hands of one of your girls, and the cream you bought for her in a pharmacy hasn't worked. You decide you will take her to a clinic, but not the one nearby, so they can fix her hands and she can get back to work.

Before you go to the clinic, you make sure she understands you won't tolerate her acting out or trying to talk to the doctor, and you'll have her family killed if she tries anything. You also decide to take her during the busy time at the clinic, to make sure they don't spend too much time with her.

You have her documents and must keep control of the situation to be sure there is no suspicion that anything criminal is going on. You dress well, are confident, and pretend to care about the health of the young woman.

You will say that you don't know why she has these sores on her hands, but they appeared about a week ago and they seem to be getting worse, so that is why you are at the clinic.

You will say whatever you need to, lie as much as necessary, so that they do not suspect anything. Your goal is that they fix her hands quickly and you can get out of the clinic and put her back to work.

ALL OTHER PARTICIPANTS: OBSERVER

Instructions: Carefully read the description of your role below. Do not share the details of your character with anyone else (only you know the details). Try to put yourself in the place of the person and do what they would do.

Watch the interaction during the role play. Do not participate, only observe. Take notes so that later you can tell them what you observed.

Keep in mind the following:

- The health care workers should try to provide personalized, individualized care, especially if he or she suspects this might be a trafficked person.
- The health care worker should make sure to do no harm in the situation, and not to put the person in danger or at risk.
- The health care worker must seek consent from the patient before saying anything to authorities.

Ask yourself:

- What did the health care worker do well?
- What could have been done differently?

Session Guide 5: Guiding Principles

Session 5: TIMETABLE OVERVIEW

This session is estimated to take 1/2 hour. It is recommended that participants complete Sessions 1, 2, 3 and 4 prior to this session. This session is recommended for the afternoon of the second day of the Core Training.

SEE PAGE 16 Core Training Overview.

Session 5: LEARNING OBJECTIVES

As this is the final presentation, the Guiding Principles session will review elements of all the other sessions:

- Understand what is human trafficking
- Identify some of the major health consequences of trafficking
- Recognize key features of trauma-informed care
- Recognize techniques for provider and patient safety
- Understand the benefits of incorporating specialized care approaches for trafficked persons
- Identify the possibilities and limitations of role of health care providers related to human trafficking

PREPARATION & MATERIALS REQUIRED

- Be sure to have copies of the external agenda and other handouts.
- If you don't have access to PowerPoint, a computer, and a projector in the training room prepare flip charts in advance of the training with the slide content.
- Materials you need include flip charts and markers, as well as something to stick the paper to the walls.

Slide 1

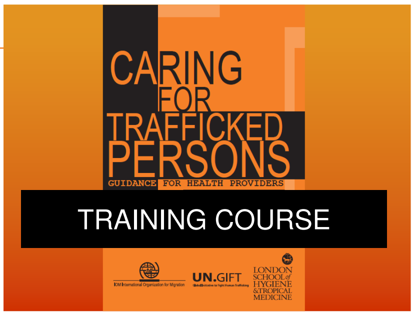

CONSIDER

This slide can be up on the screen as participants return from breaks or during changes of facilitators.

Slide 2

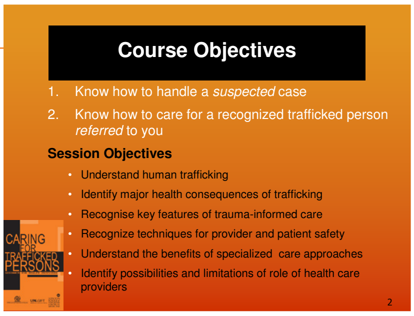

NOTE

As the final presentation, the Guiding Principles session will review elements of all the other sessions. Tell participants that we have now worked through all of the session objectives listed here.

Slide 3

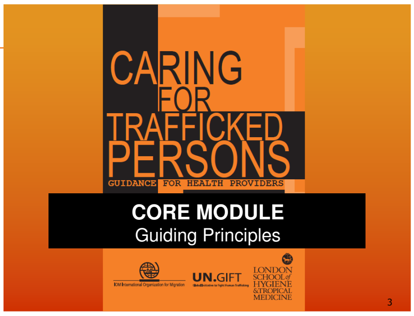

NOTE

This slide begins Session 5: Guiding principles.

NOTE

The next few slides will review some of the most important ideas from the Core Training.

Slide 4

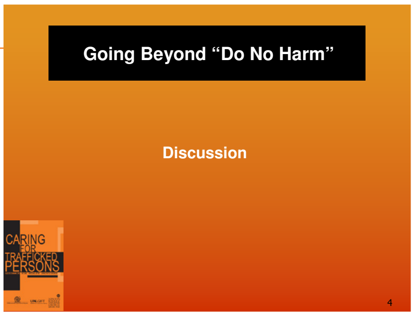

ACTIVITY

Do no harm (during presentation). As a full group, ask participants what they think this may mean. If as health care providers they always work to do no harm, why would we talk about going "beyond" this for trafficked persons?

NOTE

For trafficking, "going beyond doing no harm" means remembering that health providers can contribute to the health and further recovery of trafficked persons. It also means recognizing that a "typical" approach to doing no harm is often not enough in these cases – a trauma-informed care approach is needed.

Slide 5

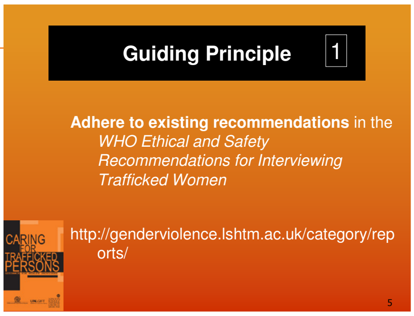

NOTE

This slide shares an important resource, the *WHO Ethical and Safety Recommendations for Interviewing Trafficked Women*. This resource provides detailed advice on how to ethically and safely interview trafficked persons. Though published in 2003 to assist trafficked women, the principles apply to all possible trafficked persons.

CONSIDER

You may want to have this resource on hand to give to participants. Consider referring them to the Recommendations section on page five of the WHO document.

Slide 6

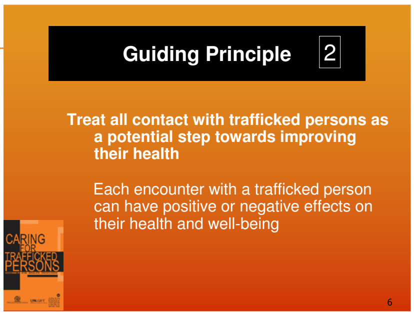

NOTE

This slide reinforces the idea that small changes in how health care providers interact with trafficked persons can support their recovery. Case workers and psychologists have found that steps to empower trafficked persons, to build their confidence and participation in care decisions related to their recovery and to reduce the unpredictability or uncontrollability of the clinical encounter can make a big difference in their recovery.

CONSIDER

You may want to remind participants that trafficked persons have experienced a situation where they felt helpless and hopeless, where they felt they had no control over their own lives. Helping them make small accomplishments can be a very important part of their recovery. Similarly, if the health provider does not pay attention to these approaches, they can have an unintended negative impact on the trafficked person's health and well-being, making the person feel uninformed and disempowered.

Slide 7

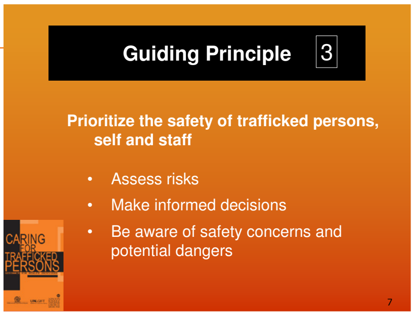

NOTE

This slide reminds health providers to always pay attention to the safety of themselves and their patient. As a good practice, health providers should be alert and aware of potential trafficking-related risks and related security options (e.g. protecting the confidentiality of the patient).

IMPORTANT

Be sure to tell your participants that this does not mean that all encounters with a trafficked person are likely to put them at risk. In fact, in most cases where someone is referred into your care, there are limited risks. But in order to protect the patient and the provider from any potentially dangerous situation, it is essential to always assume it is possible that a trafficker could be searching for a former victim, and to take precautions.

CONSIDER

You can refer participants to their handbooks and the action sheets on protection and security as well as safe referrals.

CONSIDER

For health providers who expect to work on a regular basis with trafficked persons, for example those that will be the one receiving referrals from the police or other partners, it is especially important to pay attention to the protection of victims, including security and safety, as well as confidentiality.

Slide 8

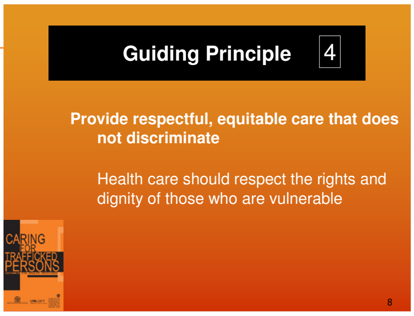

NOTE

This slide recognizes that in many cases, individuals who are trafficked may be from marginalized or stigmatized populations, such as informal migrant workers. While principles of non-discrimination are important in all health care settings, with trafficked persons respectful treatment is particularly important, and health providers should reflect on their own stereotypes and possible prejudices.

CONSIDER

Prejudice can lead to misconceptions and lead to mistakes and misinformation. Health providers should take care to communicate clearly and respectfully, to be sure trafficked persons understand and participate in their own care decisions.

Slide 9

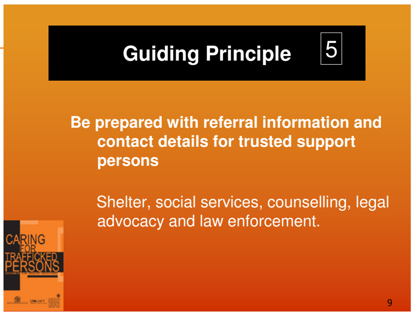

NOTE

This idea was reinforced throughout the training. It is extremely important to have this information on-hand.

Slide 10

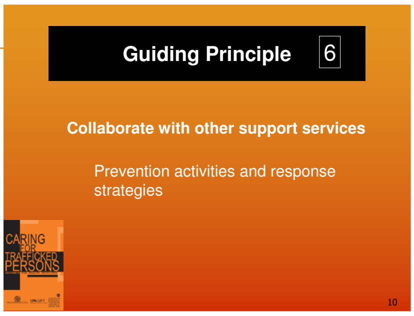

NOTE

This slide reinforces the idea that health providers will need to collaborate with other service providers to meet the needs of trafficked persons.

CONSIDER

Prevention efforts might include posting hotline information on the walls of your clinic.

Slide 11

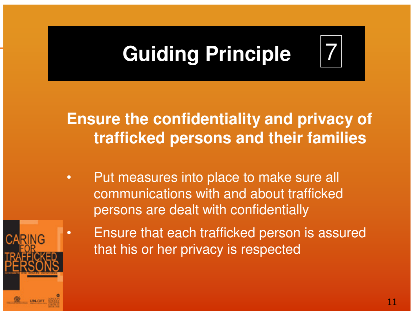

NOTE

This slide emphasizes the importance of confidentiality and privacy (remind them of the “clinical safe space”).

CONSIDER

Invite participants to think about their own work setting, and what they might need to change or adapt to be able to ensure this privacy and confidentiality.

CONSIDER

You might ask participants to think about someone who was offered a job as a waitress, but then was sexually exploited, and whose friends and family may not know the truth about what happened to them. Confidentiality is important also to protect trafficked persons from stigma and discrimination.

Slide 12

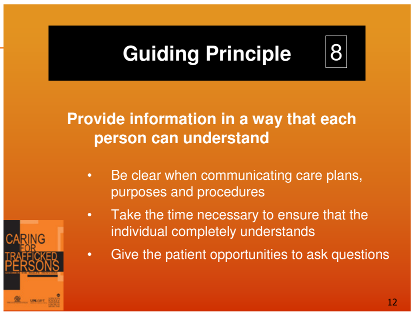

NOTE

This slide emphasizes the importance of communication. Given what we've discussed over these two days, it is clear that there can be numerous challenges associated with discussing health risks and consequences associated with trafficking in persons.

Slide 13

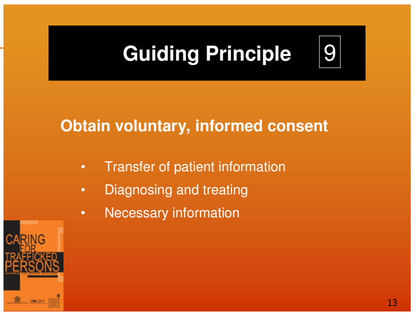

NOTE

This slide again emphasizes the importance of informed consent.

IMPORTANT

In general health care providers should share only necessary information when referring.

CONSIDER

You might want to refer participants to page 19 of the *WHO Ethical and Safety Recommendations* mentioned earlier in this session.

Slide 14

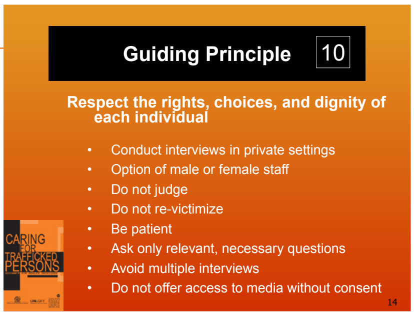

NOTE

Paying attention to details about the clinical encounter, such as the gender of the provider, the level of privacy, turning off phones, avoiding unnecessary interruptions and the way interviews are conducted can contribute to respecting the individual.

IMPORTANT

It is extremely important to only ask questions about what is relevant for you to know. Do not ask for information out of curiosity and do not ask the person to tell or repeat their story unnecessarily.

CONSIDER

It is considered good practice to ask the person whether they'd like a male or female health provider when possible, and not to assume whether talking to or being examined by a man or a woman provider would make them most comfortable.

Slide 15

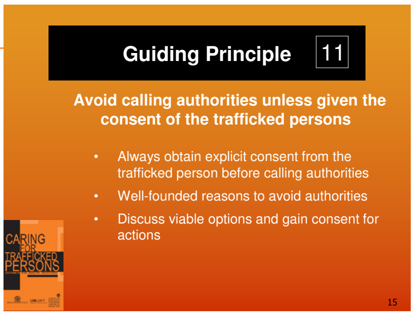

NOTE

This idea was discussed in detail during the training, but it is useful to reinforce the importance of asking individuals if they would like to have the police involved *before* calling the authorities. It is not a good idea to contact police without the consent of the person.

Slide 16

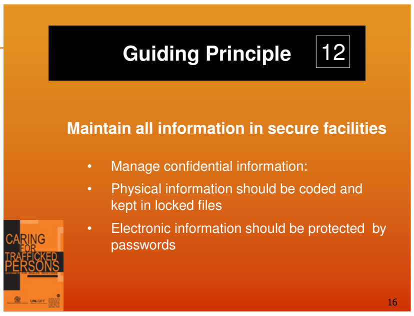

NOTE

This slide relates to the protection and safety of the trafficked person as well as the health provider. Confidentiality is essential. This may include data and case files, physical archives, electronic archives.

CONSIDER

You may want to refer participants to the handbook for more information on data security.

Slide 17

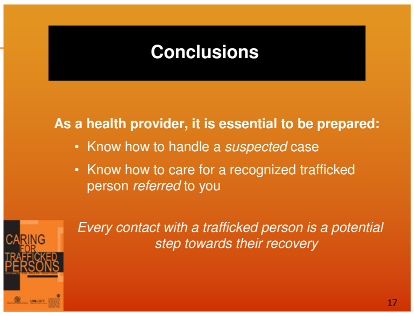

NOTE

Based on all of the content in this session and the previous sessions, it is clear that health providers must be prepared and know how to care for trafficked persons.

Slide 18

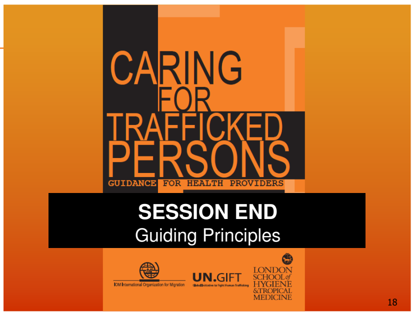

NOTE

This slide ends Session 5: Guiding principles.

If you are following the suggested timeline, this will take place during the afternoon of the second day. You may choose to end the training here, or to proceed to Session 6.

SEE PAGE 16

Core Training Overview.

Session 5 Handouts

The following handouts are included for Session 5:

- 12 Guiding Principles handout
- 10 Guiding Principles for Ethical and Safe Interviews

12 GUIDING PRINCIPLES

Listed below are the 12 guiding principles[8] for all professionals involved with persons who have been trafficked:

1. Adhere to existing recommendations in the *WHO Ethical and Safety Recommendations for Interviewing Trafficked Women* [9].

2. Treat all contact with trafficked persons as a potential step towards improving their health. Each encounter with a trafficked person can have positive or negative effects on their health and well-being.

3. Prioritize the safety of trafficked persons, self and staff by assessing risks and making consultative and well-informed decisions. Be aware of the safety concerns of trafficked persons and potential dangers to them or their family members.

4. Provide respectful, equitable care that does not discriminate based on gender, age, social class, religion, race or ethnicity. Health care should respect the rights and dignity of those who are vulnerable, particularly women, children, the poor and minorities.

5. Be prepared with referral information and contact details for trusted support persons for a range of assistance, including shelter, social services, counselling, legal advocacy and law enforcement. If providing information to persons who are suspected or known victims who may still be in contact with traffickers, this must be done discretely, e.g. with small pieces of paper that can be hidden.

6. Collaborate with other support services to implement prevention activities and response strategies that are cooperative and appropriate to the differing needs of trafficked persons.

7. Ensure the confidentiality and privacy of trafficked persons and their families. Put measures into place to make sure all communications with and about trafficked persons are dealt with confidentially and that each trafficked person is assured that his or her privacy will be respected.

8. Provide information in a way that each trafficked person can understand. Communicate care plans, purposes and procedures with linguistically and age-appropriate descriptions, taking the time necessary to be sure that each individual understands what is being said and has the opportunity to ask questions. This is an essential step prior to requesting informed consent.

9. Obtain voluntary, informed consent. Before sharing or transferring information about patients, and before beginning procedures to diagnose, treat or make referrals, it is necessary to obtain the patient's voluntary informed consent. If an individual agrees that information about them or others may be shared, provide only that which is necessary to assist the individual (e.g. when making a referral to another service) or to assist others (e.g. other trafficked persons).

10. Respect the rights, choices, and dignity of each individual by:

---

8 IOM, UN.GIFT and London School of Hygiene and Tropical Medicine, *Caring for Trafficked Persons: Guidance for Health Providers*. Geneva, 2009. Page 27-29.

9 Zimmerman, C. and C. Watts, *WHO Ethical and Safety Recommendations for Interviewing Trafficked Women*, World Health Organization, Geneva, 2003.

a. Conducting interviews in private settings.
b. Offering the patient the option of interacting with male or female staff or interpreters. For interviews and clinical examinations of trafficked women and girls, it is of particular importance to make certain female staff and interpreters are available.
c. Maintaining a non-judgmental and sympathetic manner and showing respect for and acceptance of each individual and his or her culture and situation.
d. Showing patience. Do not press for information if individuals do not appear ready or willing to speak about their situation or experience.
e. Asking only relevant questions that are necessary for the assistance being provided. Do not ask questions out of simple curiosity, e.g. about the person's virginity, money paid or earned, etc.
f. Avoiding repeated requests for the same information through multiple interviews. When possible, ask for the individual's consent to transfer necessary information to other key service providers.
g. Do not offer access to media, journalists or others seeking interviews with trafficked persons without their express permission. Do not coerce individuals to participate. Individuals in 'fragile' health conditions or risky circumstances should be warned against participating.

11. Avoid calling authorities, such as police or immigration services, unless given the explicit consent of the trafficked person. Trafficked persons may have well-founded reasons to avoid authorities; these should not be ignored, even in a perceived attempt to protect a patient.

12. Maintain all information about trafficked persons in secure facilities. Data and case files on trafficked persons should be coded whenever possible and kept in locked files. Electronic information should be protected by passwords.

10 GUIDING PRINCIPLES FOR ETHICAL AND SAFE INTERVIEWS[10]

1. Do no harm.
2. Know your subject and assess the risks.
3. Prepare referral information – do not make promises that you cannot fulfil.
4. Adequately select and prepare interpreters and co-workers.
5. Ensure anonymity and confidentiality.
6. Get informed consent.
7. Listen to and respect each person's assessment of their situation and risks to their safety.
8. Do not re-traumatize individuals.
9. Be prepared for emergency intervention.
10. Put information collected to good use.

10 Adapted from Zimmerman, C. and C. Watts, *WHO Ethical and Safety Recommendations for Interviewing Trafficked Women*, World Health Organization, Geneva 2003.

Session Guide 6: Local Context and Next Steps (Optional)

Session 6: TIMETABLE OVERVIEW

This is an optional session that focuses on promoting linkages between services, which can be included during the afternoon of the second day of the Core Training. It is estimated to take ½ hour to 1 hour, depending on whether the activity is included. It is recommended that participants complete all other Sessions prior to this optional session.

SEE PAGE 16

Core Training Overview.

CONSIDER

The final activity can be modified depending on the resources available in the context. However, in most contexts, the referral mapping exercise is useful in sharing practical information among participants.

EXTERNAL PRESENTATION

Presentation by local partner (1/2 hour). It is highly recommended that you invite a local counter-trafficking partner to present the local counter-trafficking response. In particular, ask them to include information about who to call when the participant suspects someone may have been trafficked. It is recommended that you allow time for questions and answers.

CONSIDER

Depending on the availability of the local context presenter, you might want to include this presentation elsewhere during the training (e.g. at the end of the first day or the beginning of the second day). If appropriate, you might invite them to attend the entire training.

ACTIVITY

Referral mapping (1 hour). Break the participants into small groups. Give participants a copy of the referral mapping form. Ask each group to work on completing this form with information based on the local context (name, contact information). After 30-40 minutes, invite the groups to present their answers. As with previous activities, ask each group reporting back to only add new information.

Session 6 Handouts

The following handouts are included for Session 6:

- Referral Mapping Form

REFERRAL MAPPING FORM [11]

To map out referral networks for trafficked persons.

Service	Contact details
**Local counter-trafficking organizations**	
**Telephone hotlines**	
Counter-trafficking hotline	
Family violence hotline	
Child services hotline	
Suicide hotline	
Missing persons hotline	
**Shelters & housing services**[12]	
Counter-trafficking shelter	
Domestic violence shelter	
Children & adolescent shelter	
Migrant & refugee shelter	
Homeless shelter	
Shelters run by religious or community-based organizations	
**Health services**	
Sexual health clinics and outreach services	
Reproductive health services, including (where legal) pregnancy termination services	
General practitioners	
Alcohol or drug clinics	
Mobile clinics or outreach services	
Free health services	

11 IOM, UN.GIFT and London School of Hygiene and Tropical Medicine, *Caring for Trafficked Persons: Guidance for Health Providers*.IOM, Geneva, 2009. Page 123-124

12 Shelters are sometimes managed by the government; other times they are managed by local or international organizations.

**Mental health and counselling services**	
Psychologists or therapists	
Specialists in violence-related counselling	
Mental health/psychiatric clinics	
**Non-governmental and community organizations**[13]	
Counter-trafficking	
Family violence	
Rights organizations (e.g. human rights, women's or children's rights, labour)	
Refugee or immigrant services	
Social support services	
Religious or community-based organizations	
**Legal services**	
Independent lawyers (immigration and criminal)	
Community legal aid services	
**Police, law enforcement services**[14]	
Local police contacts	
Sexual and domestic violence focal point	
Children's focal point	
**Local government contacts**	
National anti-trafficking centre	
Children's offices or services	
Women's offices or services	
Immigration services	
Housing and social services	
**Embassy and consular offices**	
Embassies & consular services for most common migrant or trafficked populations	

13 These can include local community organizations as well as international non-governmental organizations. See the UN.GIFT (Global Initiative to Fight Human Trafficking) directory of civil society partners for links to some large coalitions of organizations working in counter-trafficking: http://www.ungift.org/ungift/en/partners/civil.html.

14 Officials, including police, may be involved in criminal networks that traffic human beings. Whenever possible, identify specific focal persons who work closely with and are trusted by others working on human trafficking.

**International Organizations**	
International Organization for Migration	
International Labour Organization	
Office of the High Commissioner for Refugees	
Office of the High Commissioner for Human Rights	
United Nations Children's Fund	
United Nations Office on Drugs and Crime	
United Nations Population Fund	
World Health Organization	
Other international agencies	
**Non-governmental organizations in other countries**	
Counter-trafficking organizations based in common countries of origin	
**Interpreters**[15]	
List likely languages required	

15 It can also be helpful to note whether trusted colleagues speak a particular language, because formal interpreters are not always available. Use caution when selecting interpreters (see action sheet 3).

CON

Conclusion

CLU

SION

Conclusion

You have completed the *Caring for Trafficked Persons: Guidance for Health Providers Training Facilitator's Guide*!

Remember, the corresponding handbook, *Caring for Trafficked Persons: Guidance for Health Providers*, contains a wealth of additional information and resources, and it is highly recommended that you use it to support you during the Core Training.

For more experienced facilitators, you may want to consider including some of the other topics that are included in the handbook. For your consideration, some examples of additional topics and learning objectives are included as an annex to this guide.

> *The health provider who encounters a trafficked person or other exploited individual has a unique opportunity to provide essential medical care and vital referral options that may be the first step towards safety and recovery.*
>
> *– Caring for Trafficked Persons page 4.*

AN-

Annexes

NEXES

Annex 1: General Training Preparations

Prepare the training room

If you can, get into the training room the day before the training. If this is not possible, be sure to arrive at least an hour early in case something needs to be adjusted.

REMEMBER

Have on hand the phone number of the person to contact if something goes wrong in the training room (e.g. such as a malfunction of the equipment).

Check the layout. The training is designed to include some activities which take place in small groups, so be sure you have space for this and have thought where the groups will meet. You may want to group participants around small tables or have extra space where they can move chairs or sit apart.

Check the equipment now that you are in the room. Do you have flip chart stands? Enough paper? Markers? Is the projector working and in focus? Do you know how to adjust the lighting if you want to show a video? Is the presentation visible from the back of the room?

CONSIDER

It can be very helpful for a facilitator to know the names of participants. Depending on your context, consider name cards, badges, or name stickers (or have the participant list handy).

Ensure that the temperature in the room is acceptable (remember, it will heat up with people in it). If there is air-conditioning or heating, check that you know how to make adjustments if necessary. It is always a good idea to make sure water is on hand for participants and for the training team. Ensure that arrangements have been made to allow any participants with limited mobility to access the room.

Case studies and role plays

Case studies are written descriptions of realistic situations containing a set of problems. Often, there are no absolutely 'right' answers to these problems, but a variety of possible solutions. Case studies allow people to think about ideas in the context of real situations, and to decide how they would resolve the situation. They allow for analysis and discussion and provide a way to explore real issues rather than talking about theory.

In this training, case studies are introduced as a special type of small group discussion. Rather than asking a question to the groups, you instead ask them to read the case study or scenario, and then answer specific questions about the situation presented.

Role plays similarly present realistic situations but, instead of just describing them, role plays give participants the opportunity to act-out the situation, thus more closely 'experiencing' the feelings associated with the situation and allowing for participants to practice the use of skills and tools in a 'safe' setting. As with case studies, role plays do not present the 'right' answers. Instead role plays act as a space for exploration, reflection, and discussion. As such they can be unpredictable (participants contribute to the development of the story) and, as with case studies, they are not comprehensive. No one role play or case study can reflect all the different ways human trafficking takes place, for example.

When you are presenting a case study or role play, you should:

- Be sure to allocate enough time. In general, a case study takes 5 minutes to read, 10 minutes to discuss in a small group, 10 minutes for the small group to answer questions or complete the task, and 15-30 minutes in plenary.
- Introduce the case study or role play; briefly explain how it relates to the topic you are discussing.
- Remind participants it represents one example of the topic under consideration – there are many other possibilities.
- Outline the process: For case studies, participants will read the scenario, discuss specific questions in small groups, and then each small group will present the key elements of their conversation. For role plays, participants will read only their allocated role, interact based on their "role" as instructed by the facilitator, and then receive feedback from observers.
- Be clear as to how much time participants have for each element of the exercise (reading, discussion, and presentation/observation).
- Put the discussion questions or observation points on a flip charts or PowerPoint slide so participants can refer to them during the activity.
- Walk around during group discussions to show that you are available to answer questions, and help small groups as needed.

Group discussions

Group discussions – asking a question to the group and then allowing them to discuss it for themselves and summarize their conclusions – are a good way to get participants engaged and thinking about a topic. Small group discussions are particularly useful, because:

- they split the group into smaller sets and more people are able to speak, and;
- participants who may be uncomfortable speaking in front of 20 people often find it easier to speak in a smaller group.

In this training course you will find a number of suggested large group (plenary) and small group discussions. In order for participants to get the most out of these discussions, we suggest that you:

- Arrange the seating to encourage interaction – the best seating is probably 'cafe style' – with several small tables – as this allows both plenary and small group discussions.
- Encourage people to work with those they do not know; if participants are from various health professions and at different levels then ensure small groups are diverse (e.g. mix of seniority and experience).
- Be clear about the topic of discussion. It can help to write the topic or question on a flip charts.
- Be clear about the amount of time that the participants have for discussion.
- If you want small groups to summarize their discussions, say so before they begin talking. Tell them how long they will have to summarize, ask them to put the main points on a flip charts, and suggest they identify someone to present the summary.
- Participate in the small group discussion only when necessary. Ideally, participants should be talking to one another, not to you. You might find that this is most easily achieved by sitting down, or by moving to the side of the room.
- In plenary (whole group) discussions, intervene only to:
    - o summarize key points (and write them on a flip charts);
    - o ensure that no individual dominates the discussion: "Thank you for that point. I think it would be interesting to hear what others think. Would anyone like to give another point of view?";
    - o conclude the discussion, paying attention to time.
- In small group discussions, intervene only when a group asks you to explain something, or when it is time to end the activity.

How adults learn

As you explore the Core Training, you will notice that it involves a mix of presentations and activities. This is to help engage different participants and to limit the time spent giving presentations. In any group of people, you will probably have four different learning 'styles' represented:

- Visual: people who learn best by seeing things. Visual learners will typically remember pictures, colours and diagrams.
- Auditory: people who learn by hearing. Auditory learners will typically remember what is said to them, particularly if it is said with variations in volume, tone and rhythm.
- Read/Write: people who learn through the written word. These learners benefit from flip charts, PowerPoint's and the ability to take notes (even if they already have the notes in front of them!).
- Kinaesthetic: people who learn by doing and by physical action. These are people who learn best from experience, or from role plays. They may want to walk around, or fiddle with pencils or pens.

It is important to remember that adults learn in a different way from children. In particular:

- Adults seldom learn 'for fun': they want to know why they need to learn something new. If there is not a powerful motivation to learn, they probably won't.
- Adults are people with a lot of experience and knowledge. They relate new knowledge and information to previously learned knowledge and experience. They often need to ask a lot of questions in order to make these links.
- Adults expect to be treated as equals, and like to be able to challenge both content and process.

What does this mean for you as you help health care providers to learn about caring for trafficked persons and other exploited migrants? Generally:

- You should try to keep your own talking to a minimum.
- You should do your best to ensure that everyone is engaged, and able to discuss and ask questions.
- You should ask participants to talk about their own experiences, in the context of the course.
- You should explain why the course is useful, and ask the participants to consider and discuss how the course will help them in their work.
- You should try to use a variety of visual aids, provide written handouts and space to write, and use changes in rhythm and volume when you speak. This will help visual, auditory and read / write learners.

Interactive Presentations

What makes a good presentation? There are three golden rules to a good presentation:

Rule 1: You should be 100 per cent comfortable with the presentation. This means being well prepared.

For more on this, see the preparation sections above.

Rule 2: The PowerPoint slide, or flip chart, is just a visual aid to help you. YOU are the centre of the presentation.

The main instrument you have as a presenter is not the PowerPoint, it is your voice. The tone and sound of your voice communicates at least as much as the words that you say. So use your voice well. Remember, in particular, the importance of variety, vocabulary, and volume:

Variety: Vary your tone, volume and speaking speed. Increasing volume and speed will build excitement and enthusiasm. Slowing speed, and decreasing volume – will make people concentrate on what you are saying. Pausing will make people hang on your next word, prepare for the next point.

Volume: The general volume should be one that can be heard comfortably at the back of the room. Pay attention to whether your participants can hear you.

Vocabulary: Use clear language. Use participants' names, as this connects the participants to the presentation.

Another important instrument is your posture and 'body language'. If you appear confident and open, participants will be more open to what you have to say:

- Stand up! You should always present on your feet. Only sit down when you want the group to ignore you (when they are conducting small group exercises, for example).
- Face the audience. Make sure you have set the room up so that you can change PowerPoint slides, write on flip charts, etc., and still be facing the group.
- Maintain eye contact by using the 'lighthouse technique'. Sweep the group with your eyes, like a lighthouse beam, as you are talking, to include everyone.
- Use movement to your advantage: the human eye is very attuned to movement, and you can use this: move when there is a reason to do so – to emphasize a point, or to catch attention. When you are not moving, find one comfortable space and anchor yourself there. Go back to this space when you have finished moving.
- Use open body language. Try not to 'shield' your body with hands and arms – leave your body as open as possible. Also – leave your hands open. Only point, or make a fist, for extra emphasis.

Rule 3: Allow space for participants – either during or after the presentation – to ask questions and interact with the ideas

Remember that people need to ask questions to learn. Also, it is a general rule of thumb that most people cannot concentrate for more than ten minutes or so. Questions and interaction break the presentation up, and allow people to 'refocus' their concentration.

So, if the presentation is scheduled to last for more than ten minutes, plan on breaking it up to ask some questions. When asking questions, it helps to:

- Ask questions that begin with 'what; why; how; when' – these are called open questions, and cannot be answered by a simple 'yes' or 'no' response. This forces participants to think more about the question.
- Show that you are listening carefully to the answers that participants give, and thank them for their responses.
- Paraphrase the answers you are given back in your own words – this shows that you have understood the answer, and allows participants who may not have heard the answer to hear it.
- Ask: "does anyone have anything to add?"
- Before you begin the next part of the presentation, summarize all of the answers and add any extra ideas that did not come up from the participants.

If the presentation is fairly short, ask participants to wait until the end of the presentation, and then open a discussion. If participants do not have questions, you can ask:

- What was new or surprising in this information?
- What do you think was the most important thing in that presentation?
- How could you apply this to a work situation?

Annex 2: Additional Suggested Topics

These additional training topics and suggested learning objectives are based on the content of the handbook *Caring for Trafficked Persons: Guidance for Health Providers* and are included here for the consideration of more experienced facilitators who may want to include additional information in their trainings.

Focus on communication
Learning objectives:

1. Apply basic techniques for effective communication using interpreters
   a. Clarity on the role and limitations of the interpreter
   b. Proper selection and monitoring of an interpreter
   c. Ensuring clinical safe space during consultation
2. Identify beneficial approaches for communicating with individuals from varying backgrounds and with different expressions of illness and wellness.
   a. Explanatory model
   b. Culturally responsive – language, literacy, etc.
   c. Provider-patient negotiation / empowerment

Self-care
Learning objectives:

1. Recognize signs of provider fatigue or burnout and appropriate responses for self and colleagues.
   a. Signs of fatigue or burn out
   b. Ways to prevent / cope
   c. Compassion fatigue scale (tool)

Medico-legal issues
Learning objectives:

1. Identify fundamental rights and responsibilities related to forensic examinations.
   a. Know who should perform forensic examinations and proper timing
   b. Never collect unusable samples
   c. Informed consent / rights-based
   d. Trauma-informed / process
2. Identify appropriate methods for assessing the capacity and need for legal guardianship of patients in your care.
   a. Culturally responsive communication
   b. Non-judgemental / empowerment
   c. Rights-based / best interest / legal requirements
   d. Assessment by specialist
   e. Box on competence (tool)
3. Describe key features of 'informed consent' and apply procedures for informed consent.
   a. Informed – appropriate communication
   b. Impaired judgement / distress and symptoms
   c. In writing
   d. Legal requirements

4. Recognize responsible approaches to sharing information with authorities, including law enforcement, courts and immigration services.
    a. Crime – trafficking prosecution / prosecution of other crimes / criminalization of the victim (e.g. illegal exploitation activity or migration status)
    b. Security / protection / safe network (e.g. requests for information from authorities)
    c. Information shared only with informed consent or judicial order
    d. Don't provide legal counsel / opinions
    e. Don't involve the police unless the person wants you to

Sub-speciality: Mental health
Learning objectives:

1. Identify the complex set of past stressors and repetitive exposures beyond trafficking-related events that may be contributing to poor mental health or distress.
    a. Pre-existing mental health conditions
    b. Cumulative / chronic violence
2. Consider current or future events that may be affecting individuals' mental health.
    a. Non-health stressors related to legal situation / criminal prosecution / shelter and basic needs / hostile environments / etc.
    b. Identify a range of basic non-clinical responses to foster reduced stress levels.
    c. Establish routines
    d. Talk and ask about common psychological symptoms of trafficked / exploited persons
    e. Non-drug approach
    f. Trauma-informed/ empowering / patient-centred
    g. Be prepared to listen to details but do not push for "debriefing"

Sub-speciality: Sexual and reproductive health
Learning objectives:

1. Implement sensitive approaches to conducting clinical exams with people who have been exposed to traumatic events or high-risk situations.
    a. Patient-centred, culturally appropriate communication
    b. Trauma-informed, process, informed consent
2. Recognize fundamental responsibilities and options regarding the collection of forensic evidence.
3. Consider the specific sexual and reproductive health information needs of trafficked persons.
    a. Non-judgemental, rights-based approach, menu of options and information (access to contraceptives, STI prevention and care, pregnancy tests and obstetric care, etc.)
    b. Patients set their own priorities

Sub-speciality: General practice and urgent care
Learning objectives:

1. Consider and document a range of health risk factors (e.g. occupational, environmental, abuse) that may be affecting an individual's health;
    a. Consider additional history questions in the assessment
2. Have a greater appreciation of potential links between current symptoms and past exposures, including epidemiological prevalence zones for communicable diseases.
    a. Consider mobility, migration – health profile of migrants (where people come from)
    b. Be aware of common symptoms of distress in trafficked / exploited persons and how it may affect their communication with you
    c. Consider effects of cumulative violence on communication during urgent assessments

3. Identify procedures to respond to emergency care needs and make safe referrals.
    a. Consider potential life-threatening injuries, conditions common in trafficked persons
    b. Cumulative health problems and lack of medical attention over time
    c. Consider a toxicology screen (withdrawal, forced drug use, drug use for coping)

Sub-speciality: Paediatrics and adolescent care
Learning objectives:

1. Consider issues related to consent and guardianship related to trafficked youth.
    a. Be aware that family members / guardians may have been involved in the trafficking / exploitation
    b. Be aware that children in work settings may be considered exploited / trafficked even if they seem to consent they cannot by law in many settings
    c. Be aware of local legal requirements on reporting (e.g. child abuse, particular diseases in children) and guardianship
2. Identify some features of developmental effects associated with trafficking abuses.
    a. Be aware of developmental effects as a result of malnutrition, sexual abuse and exploitation, violence, exposure to infectious diseases, substance abuse
    b. Cognitive and developmental delays, regression, behavioural problems
3. Have a greater appreciation for age-appropriate approaches to care.
    a. Tailor approaches to the abuse and neglect experience of child (e.g. malnutrition, lack of vaccines, lack of dental care)
    b. Child-appropriate and trauma informed assessment / exams
    c. Special needs of orphaned / separated children
    d. Consistent care box

International Organization for Migration (IOM)

17 route des Morillons 1211 Geneva 19 Switzerland
Tel.: + 41 22 717 91 11 • Fax: + 41 22 798 61 50
E-mail: hq@iom.int • Internet: www.iom.int
