# Supplementary material for: Health Care Providers and Human Trafficking: What do They Know, What do They Need to Know? Findings from the Middle East, the Caribbean, and Central America
Source: Front Public Health. 2015 Jan 29;3:6. doi: 10.3389/fpubh.2015.00006 (PMC4310216; doi:10.3389/fpubh.2015.00006)
Supplement: Supplementary file 1 [file Presentation_1.ZIP › Caring for Trafficked Persons Training Session 1.pptx]

## Slide 1
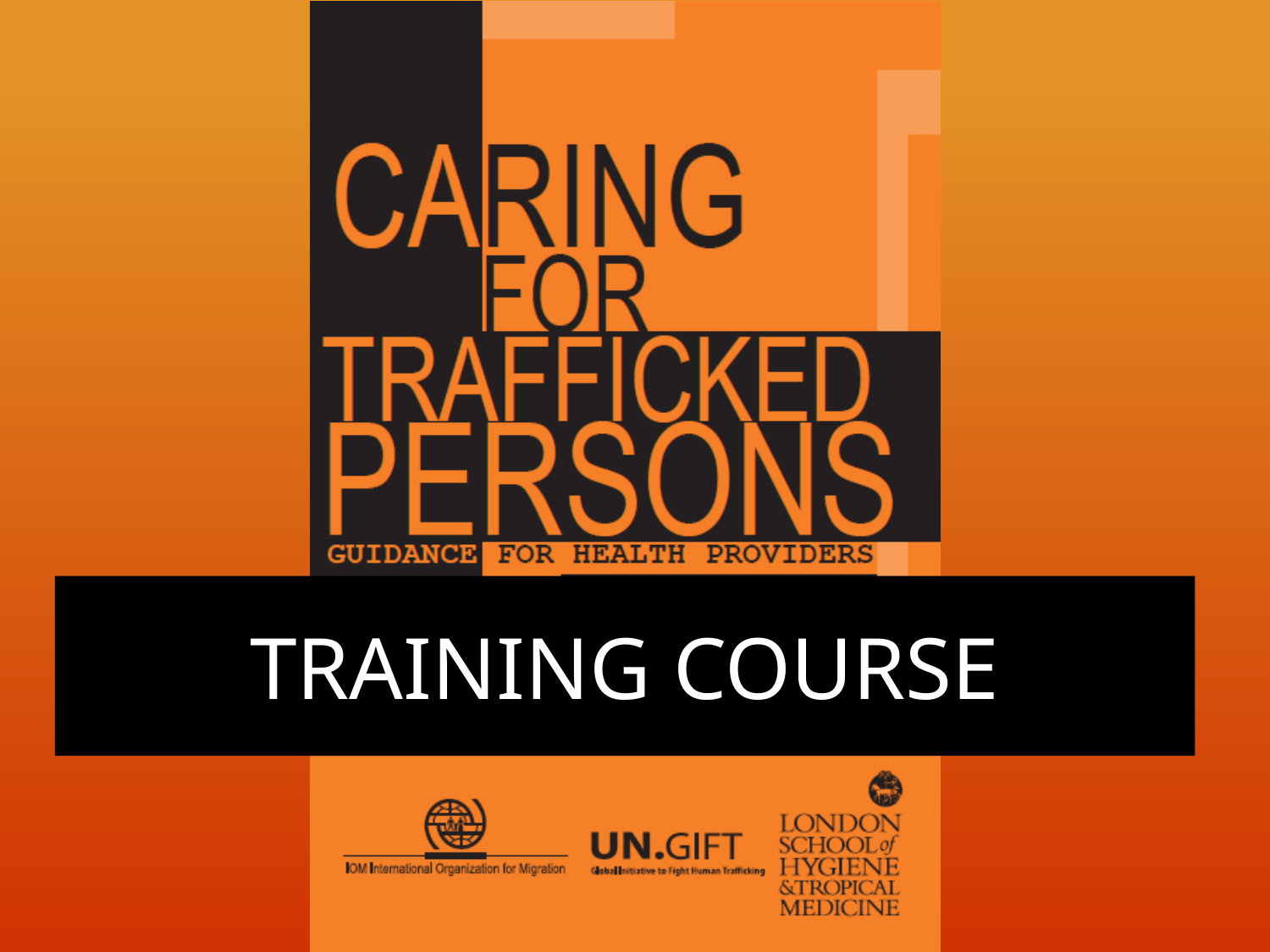

## Slide 2
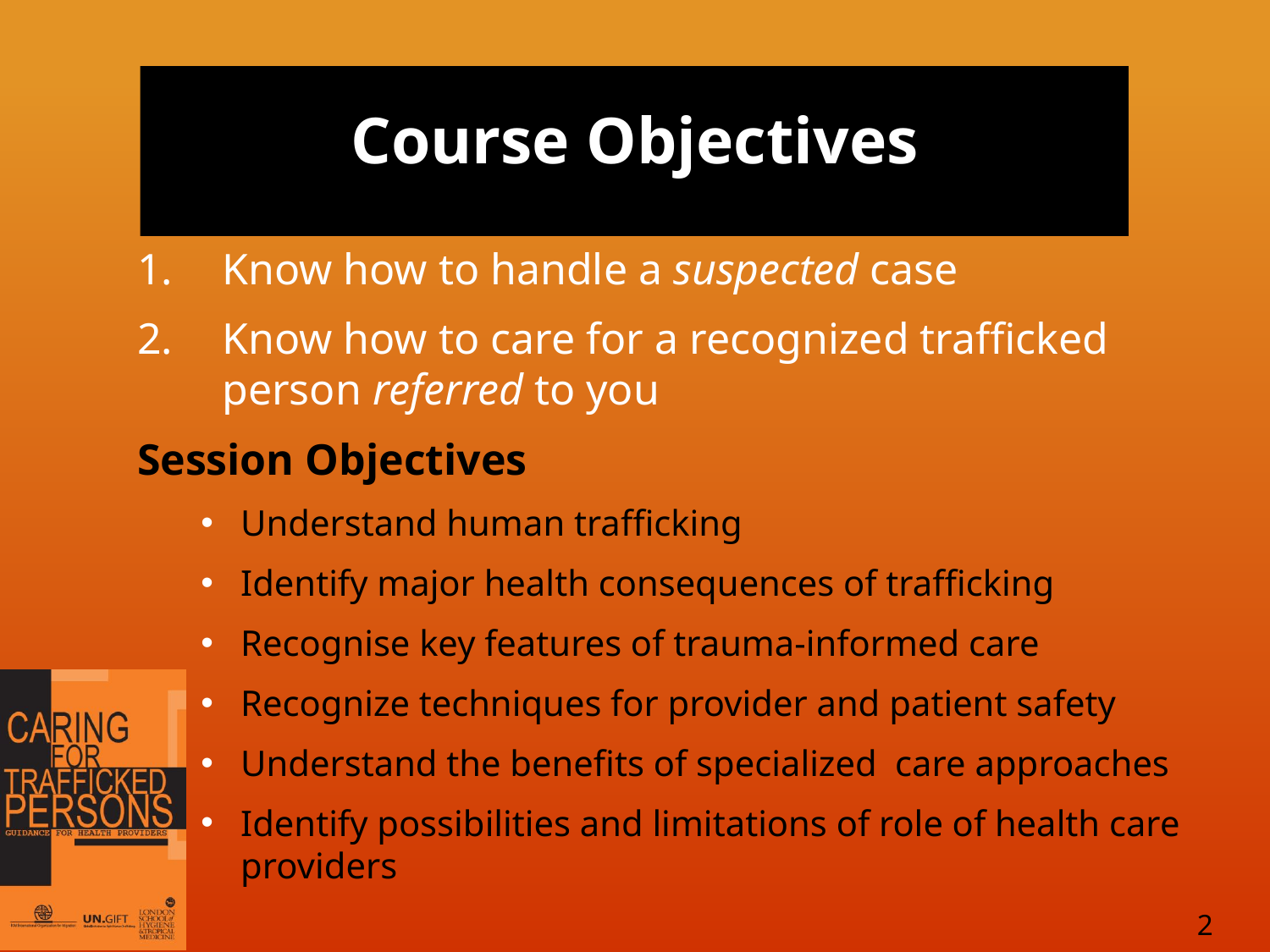

# Course Objectives
Know how to handle a suspected case
Know how to care for a recognized trafficked person referred to you
Session Objectives
Understand human trafficking
Identify major health consequences of trafficking
Recognise key features of trauma-informed care
Recognize techniques for provider and patient safety
Understand the benefits of specialized care approaches
Identify possibilities and limitations of role of health care providers
2

## Slide 3
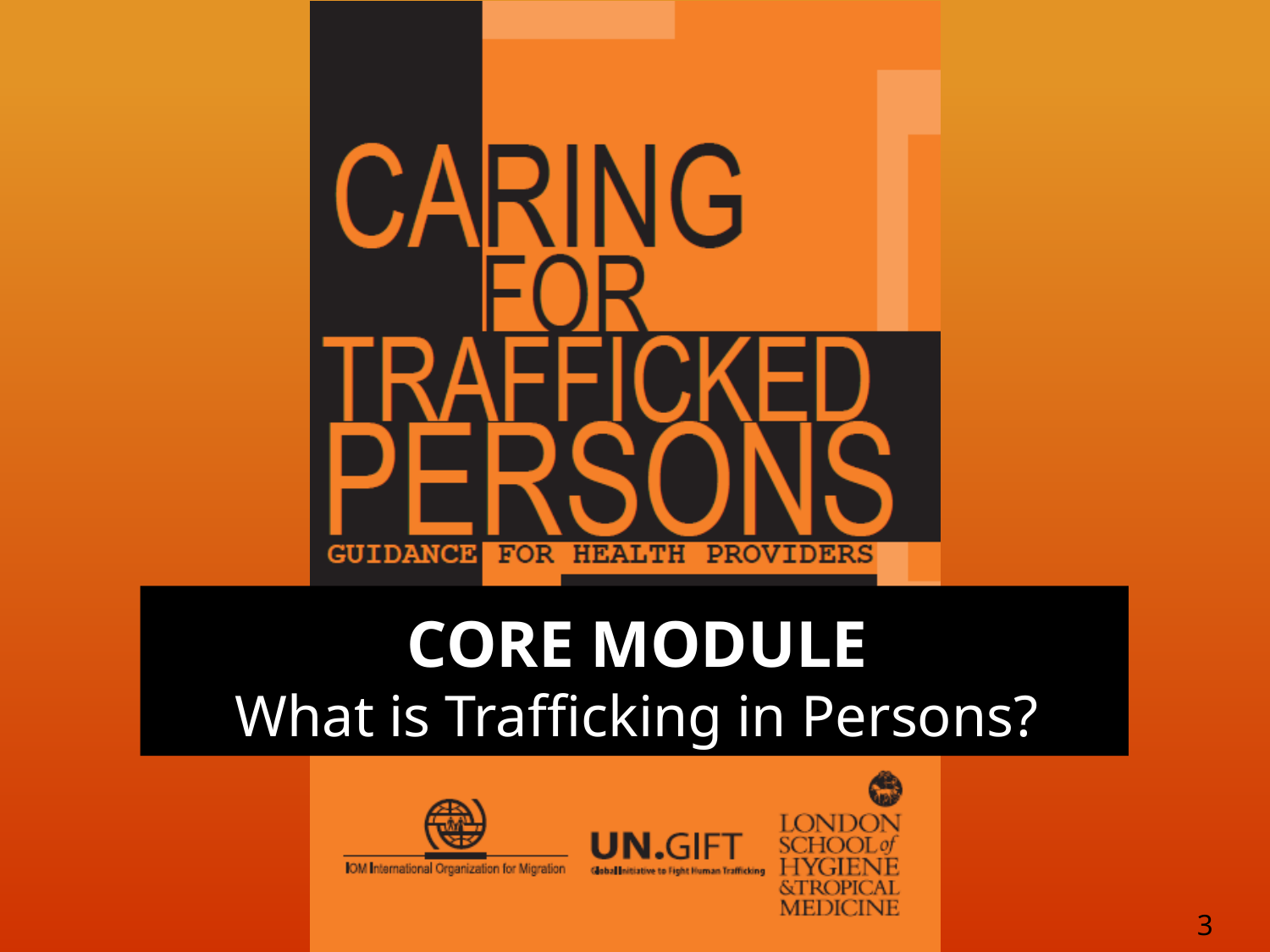

CORE MODULE
What is Trafficking in Persons?
3

## Slide 4
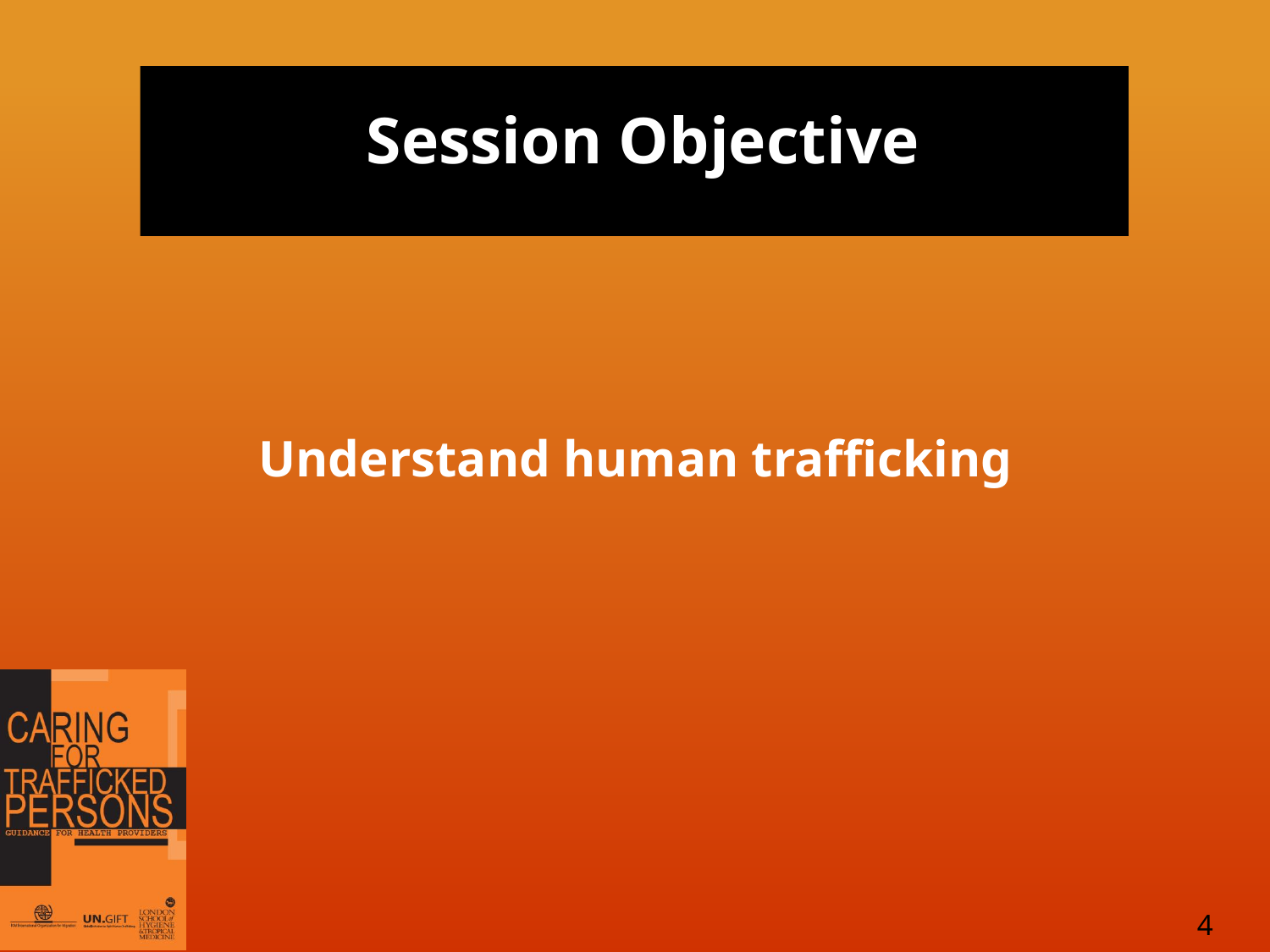

# Session Objective
Understand human trafficking
4

## Slide 5
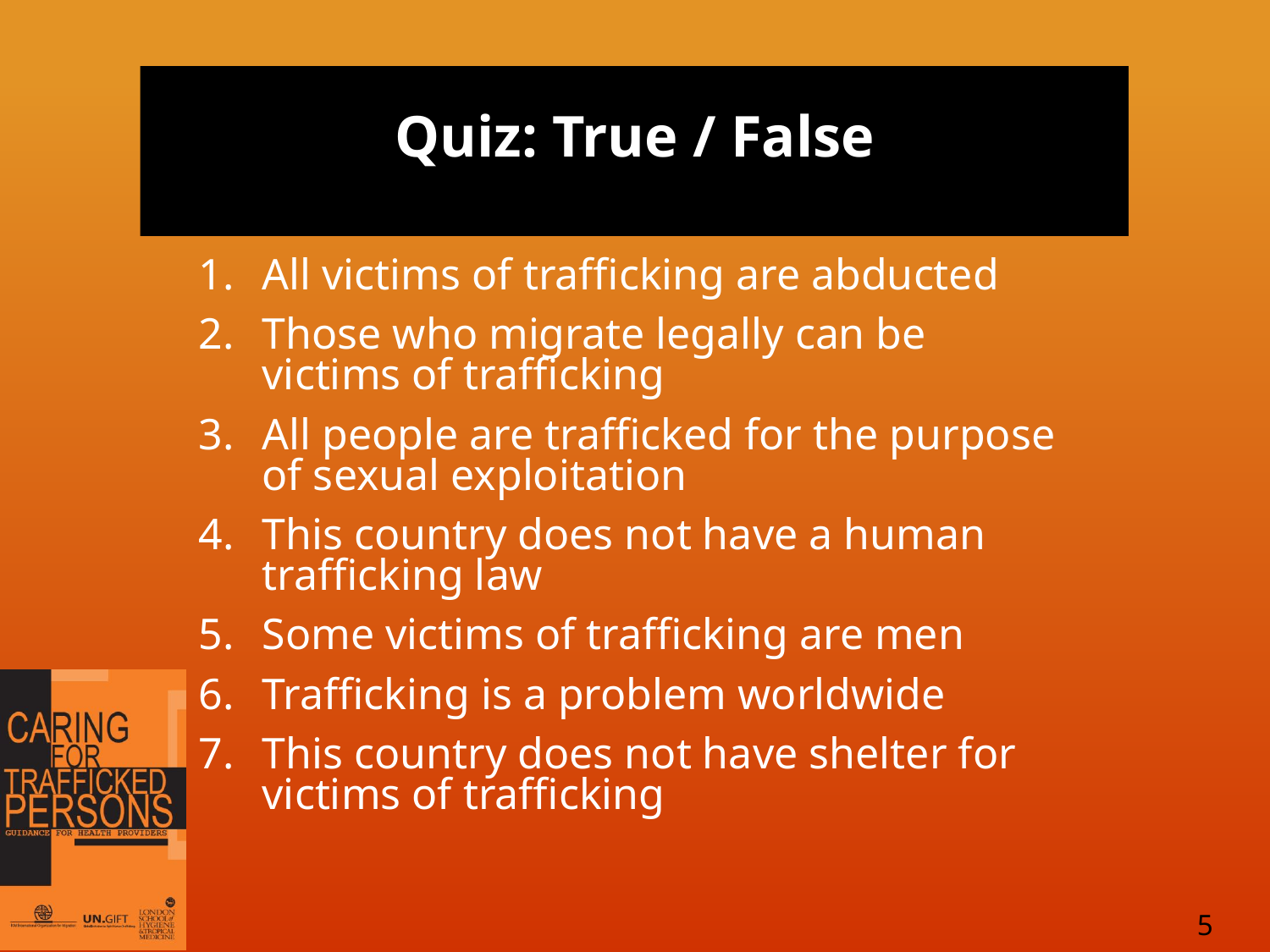

# Quiz: True / False
All victims of trafficking are abducted
Those who migrate legally can be victims of trafficking
All people are trafficked for the purpose of sexual exploitation
This country does not have a human trafficking law
Some victims of trafficking are men
Trafficking is a problem worldwide
This country does not have shelter for victims of trafficking
5

## Slide 6
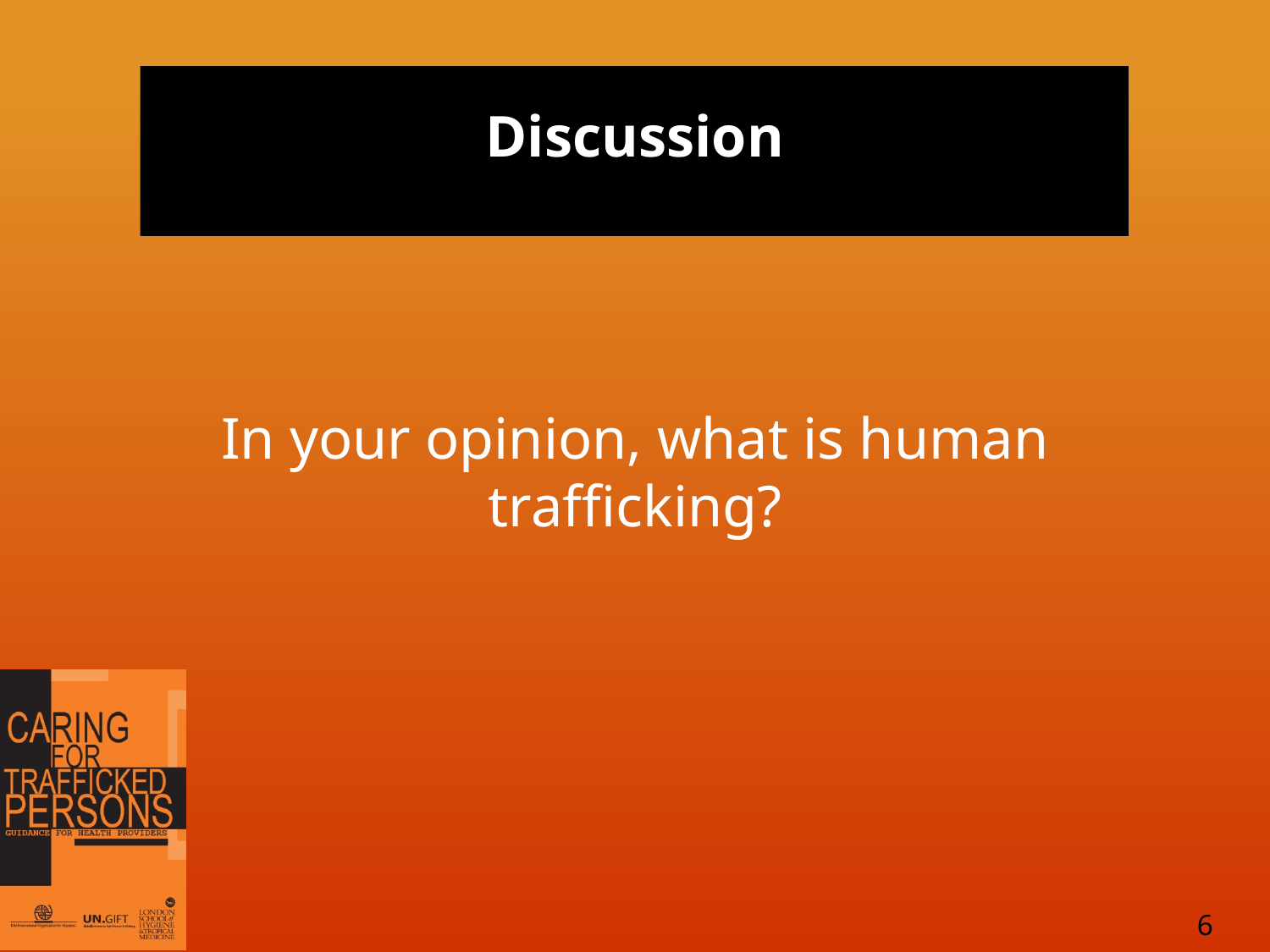

# Discussion
In your opinion, what is human trafficking?
6

## Slide 7
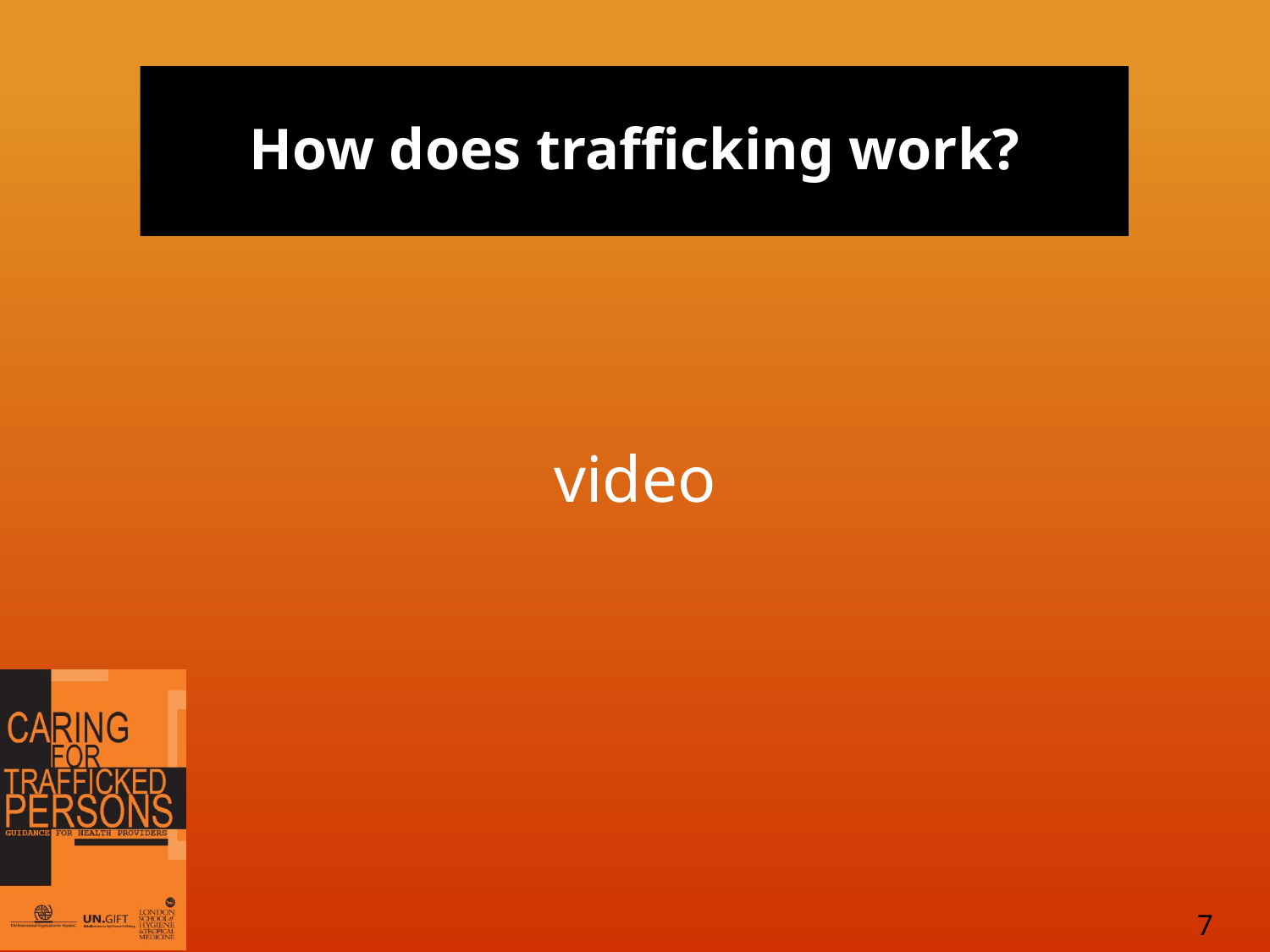

# How does trafficking work?
video
7

## Slide 8
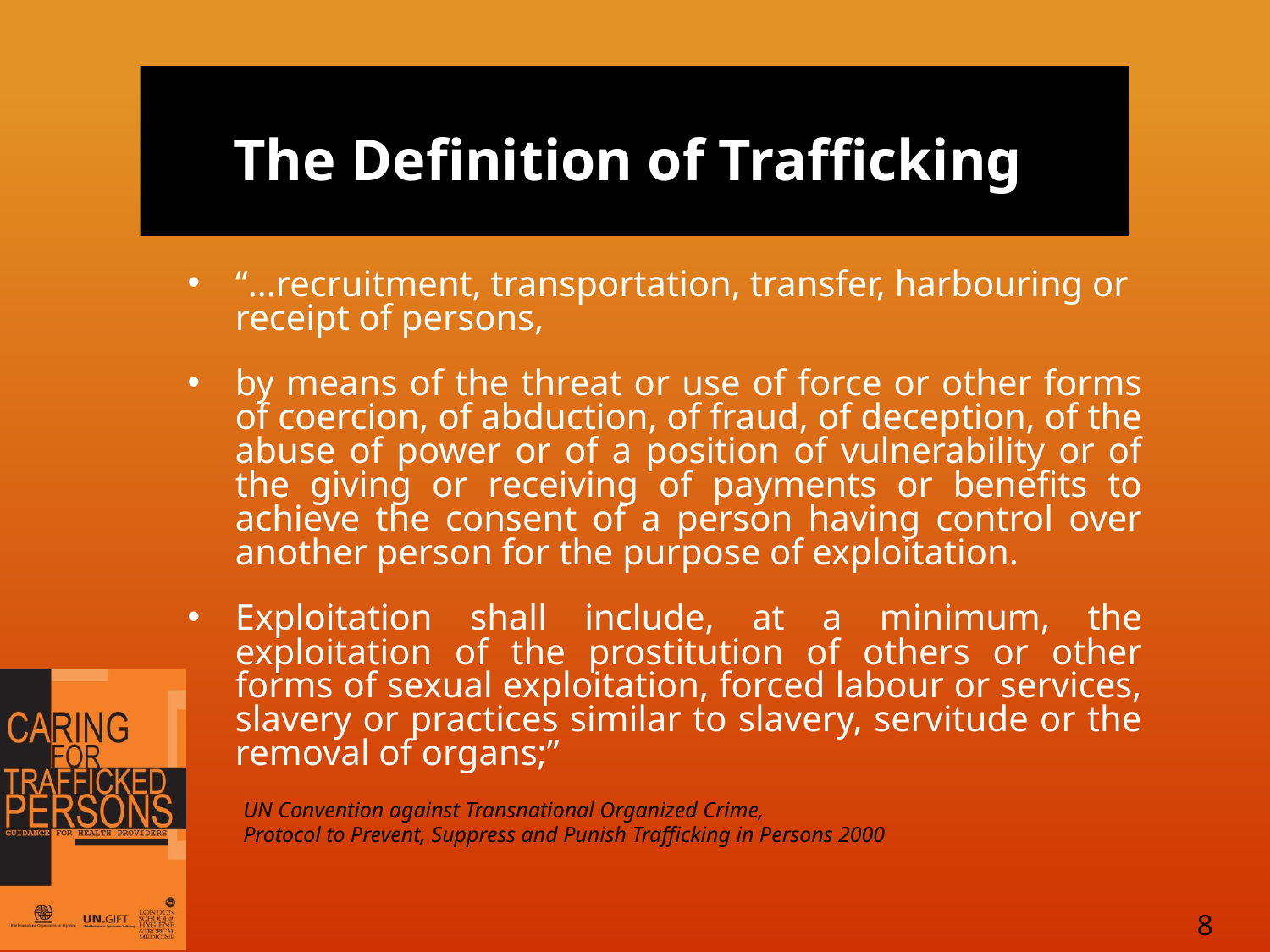

# The Definition of Trafficking
“…recruitment, transportation, transfer, harbouring or receipt of persons,
by means of the threat or use of force or other forms of coercion, of abduction, of fraud, of deception, of the abuse of power or of a position of vulnerability or of the giving or receiving of payments or benefits to achieve the consent of a person having control over another person for the purpose of exploitation.
Exploitation shall include, at a minimum, the exploitation of the prostitution of others or other forms of sexual exploitation, forced labour or services, slavery or practices similar to slavery, servitude or the removal of organs;”
UN Convention against Transnational Organized Crime,
Protocol to Prevent, Suppress and Punish Trafficking in Persons 2000
8

## Slide 9
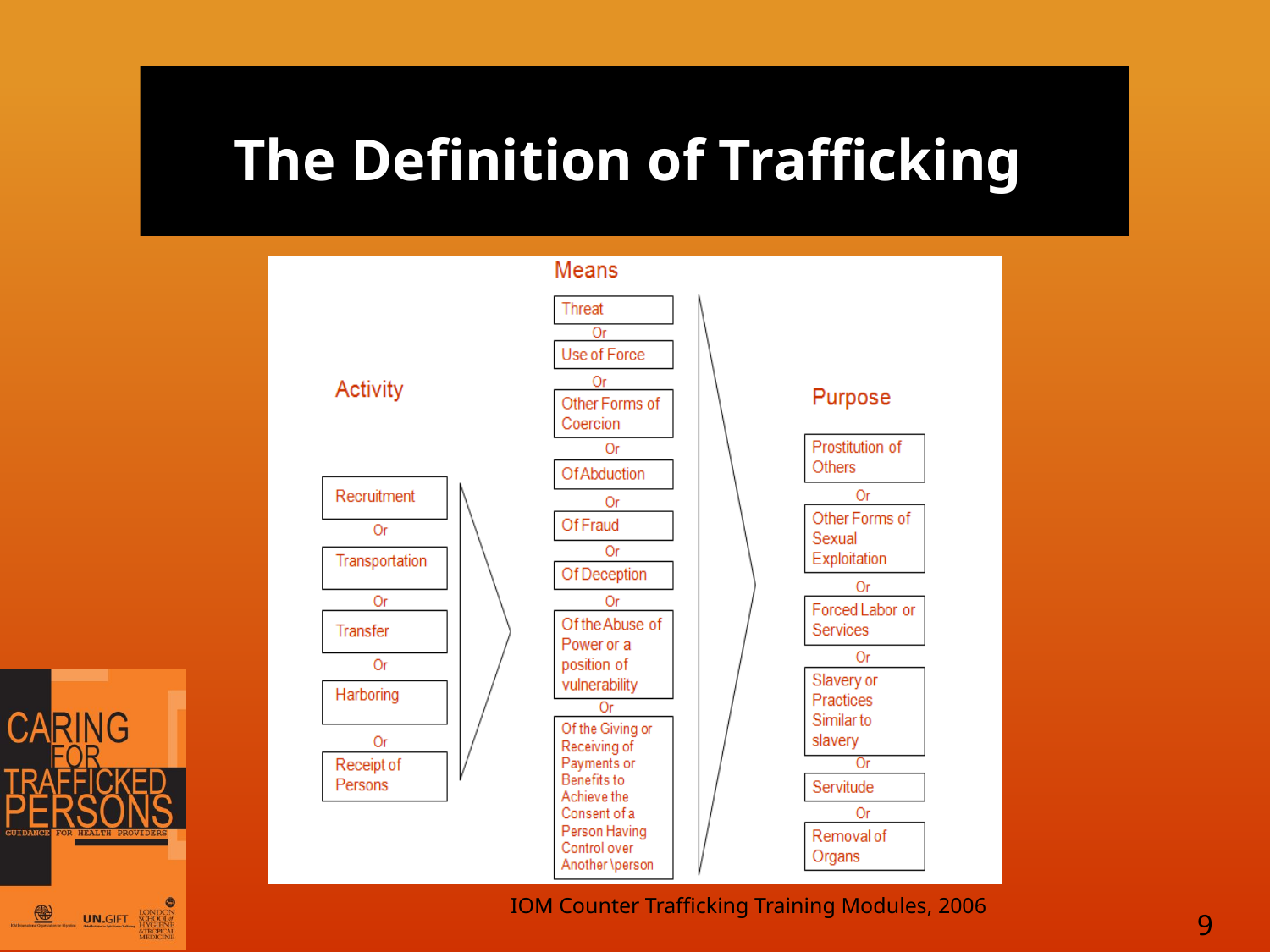

# The Definition of Trafficking
IOM Counter Trafficking Training Modules, 2006
9

## Slide 10
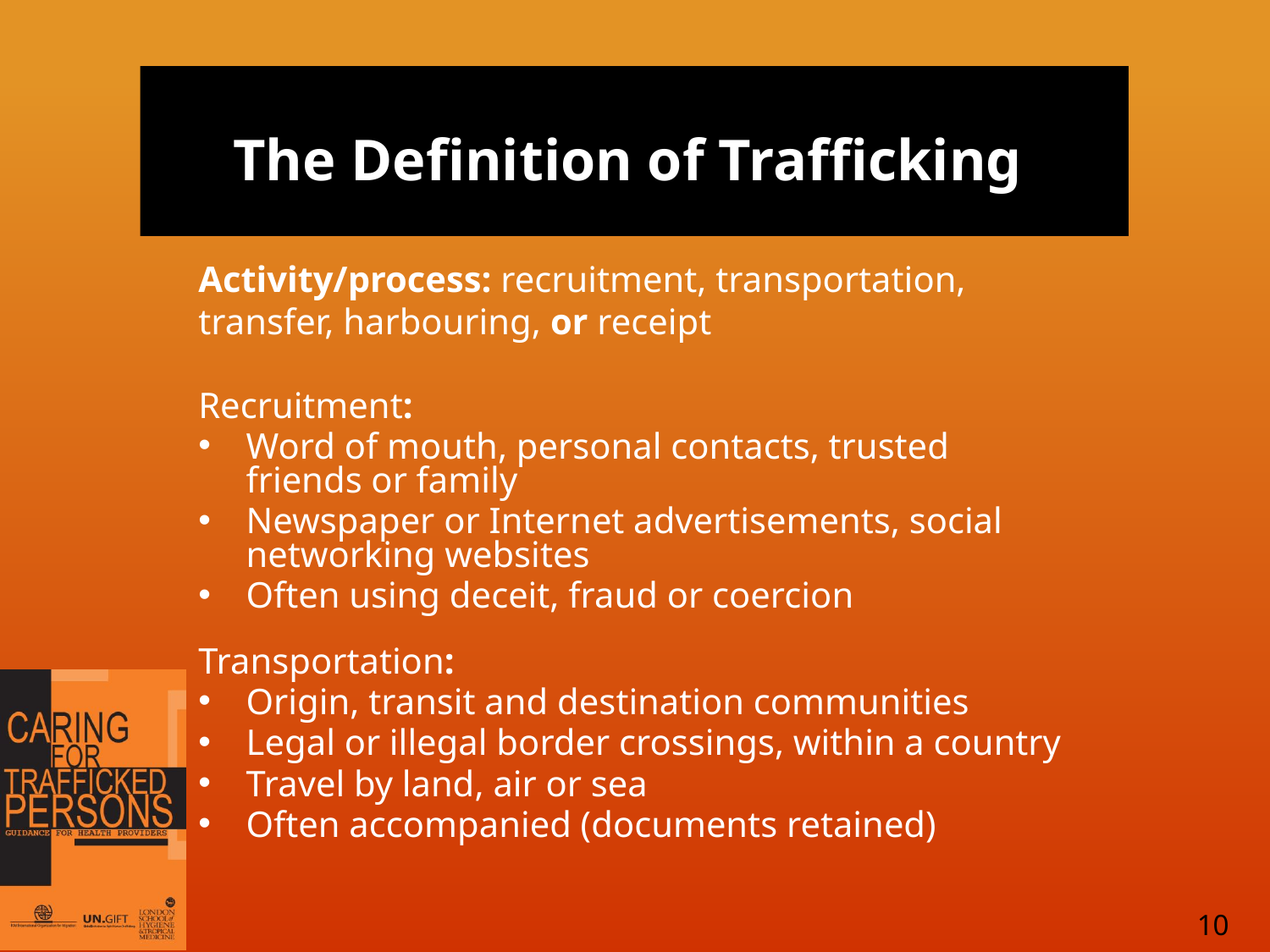

# The Definition of Trafficking
Activity/process: recruitment, transportation, transfer, harbouring, or receipt
Recruitment:
Word of mouth, personal contacts, trusted friends or family
Newspaper or Internet advertisements, social networking websites
Often using deceit, fraud or coercion
Transportation:
Origin, transit and destination communities
Legal or illegal border crossings, within a country
Travel by land, air or sea
Often accompanied (documents retained)
10

## Slide 11
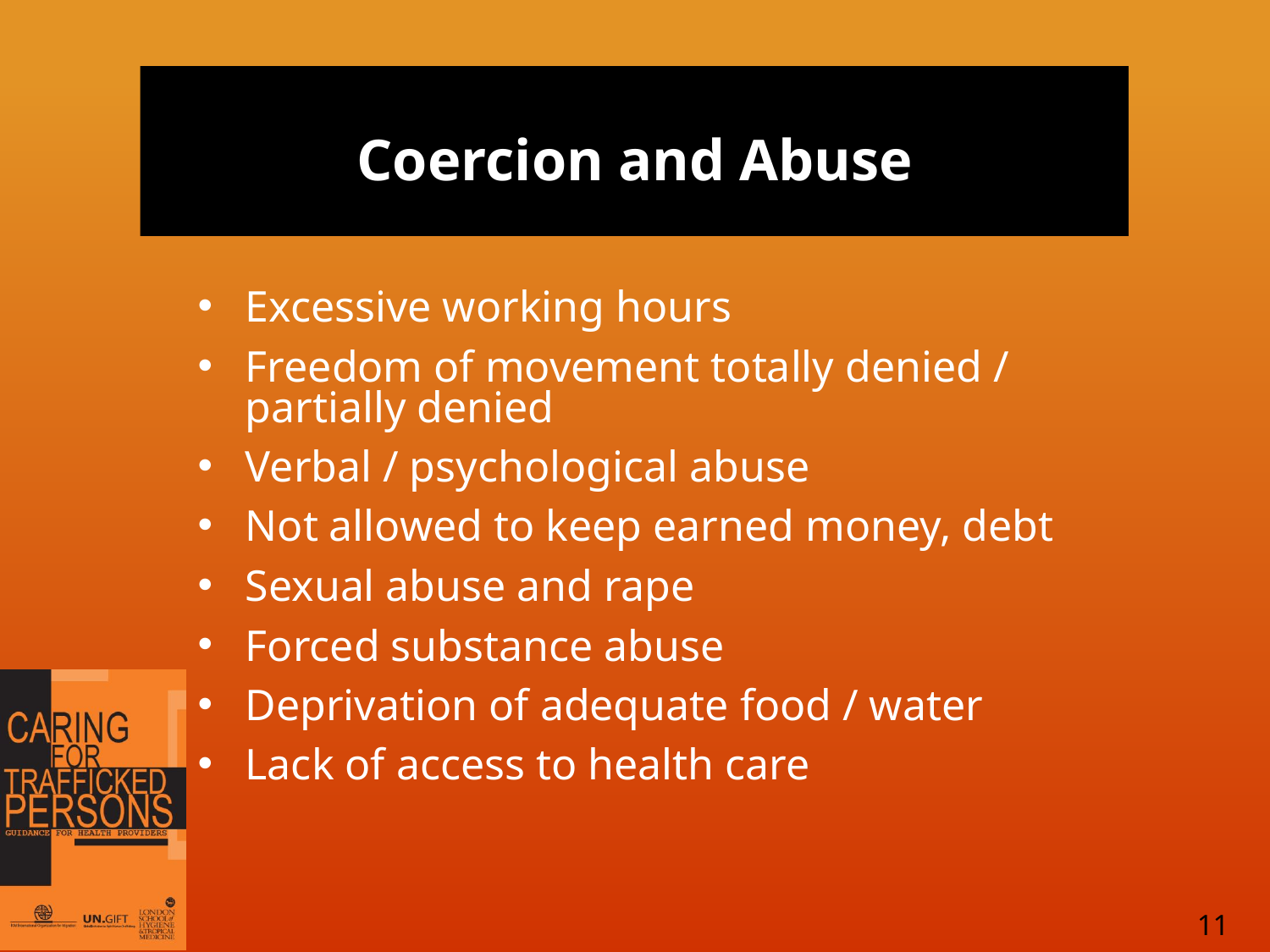

# Coercion and Abuse
Excessive working hours
Freedom of movement totally denied / partially denied
Verbal / psychological abuse
Not allowed to keep earned money, debt
Sexual abuse and rape
Forced substance abuse
Deprivation of adequate food / water
Lack of access to health care
11

## Slide 12
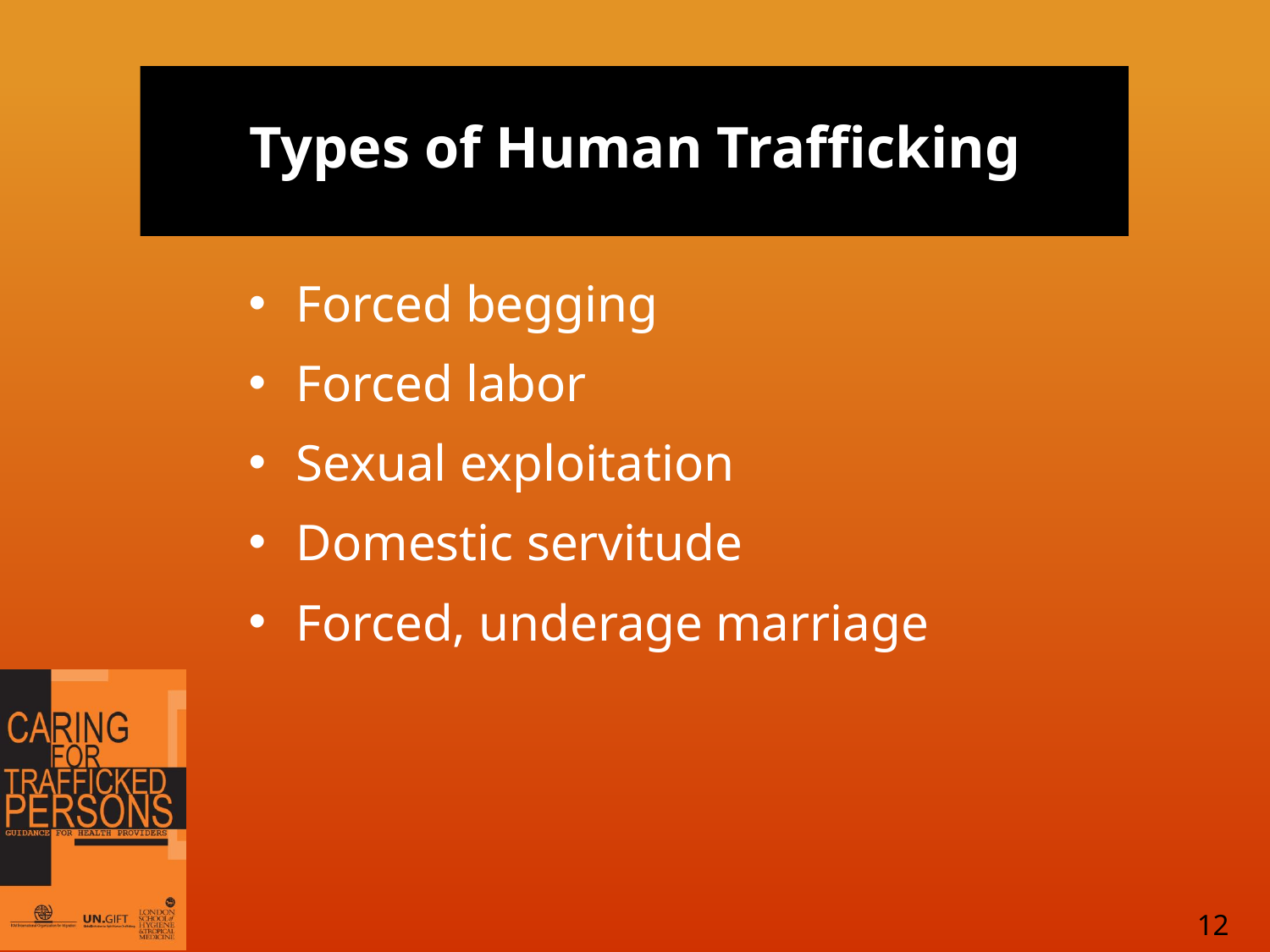

# Types of Human Trafficking
Forced begging
Forced labor
Sexual exploitation
Domestic servitude
Forced, underage marriage
12

## Slide 13
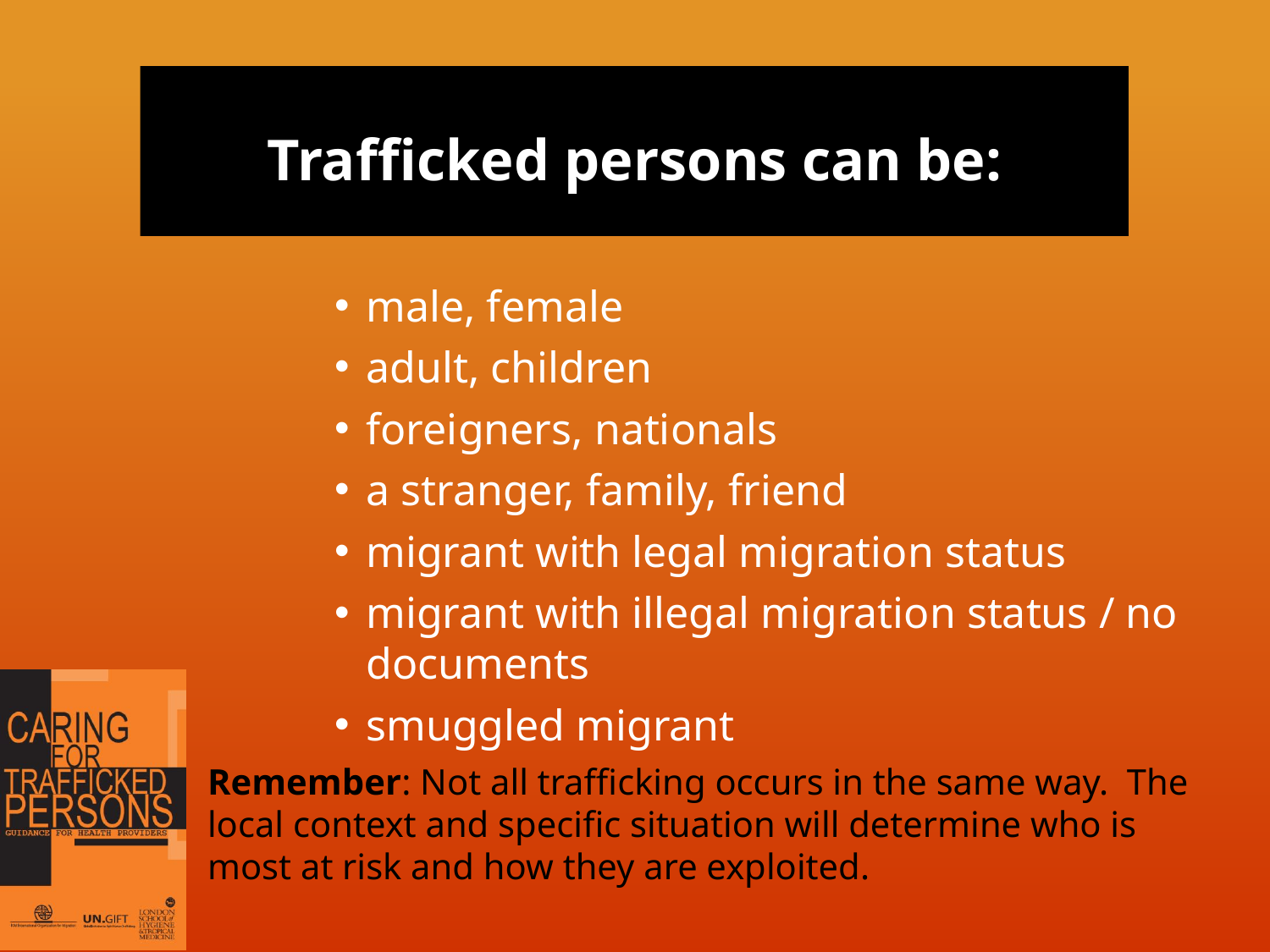

# Trafficked persons can be:
male, female
adult, children
foreigners, nationals
a stranger, family, friend
migrant with legal migration status
migrant with illegal migration status / no documents
smuggled migrant
Remember: Not all trafficking occurs in the same way. The local context and specific situation will determine who is most at risk and how they are exploited.

## Slide 14
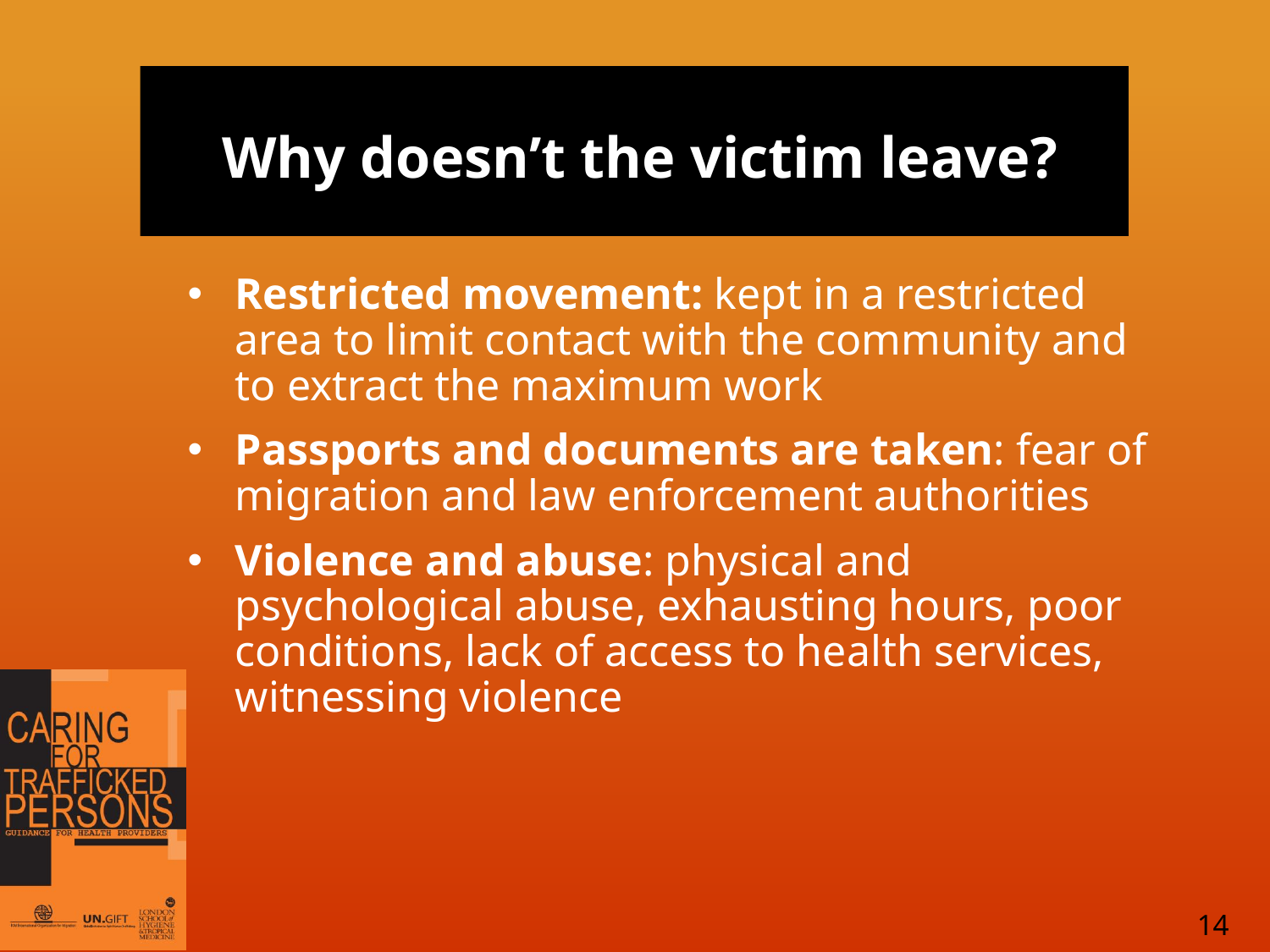

# Why doesn’t the victim leave?
Restricted movement: kept in a restricted area to limit contact with the community and to extract the maximum work
Passports and documents are taken: fear of migration and law enforcement authorities
Violence and abuse: physical and psychological abuse, exhausting hours, poor conditions, lack of access to health services, witnessing violence
14

## Slide 15
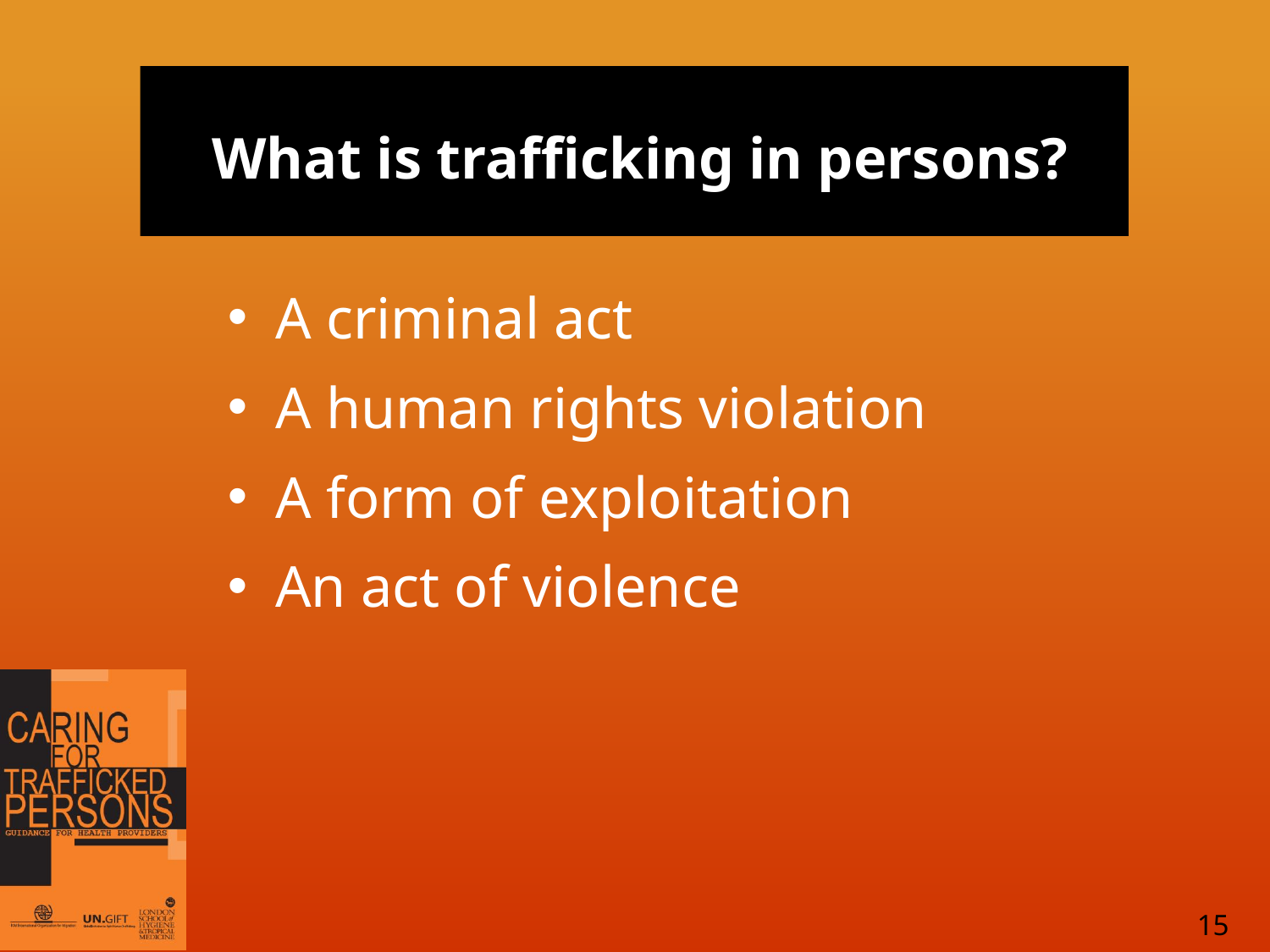

# What is trafficking in persons?
A criminal act
A human rights violation
A form of exploitation
An act of violence
15

## Slide 16
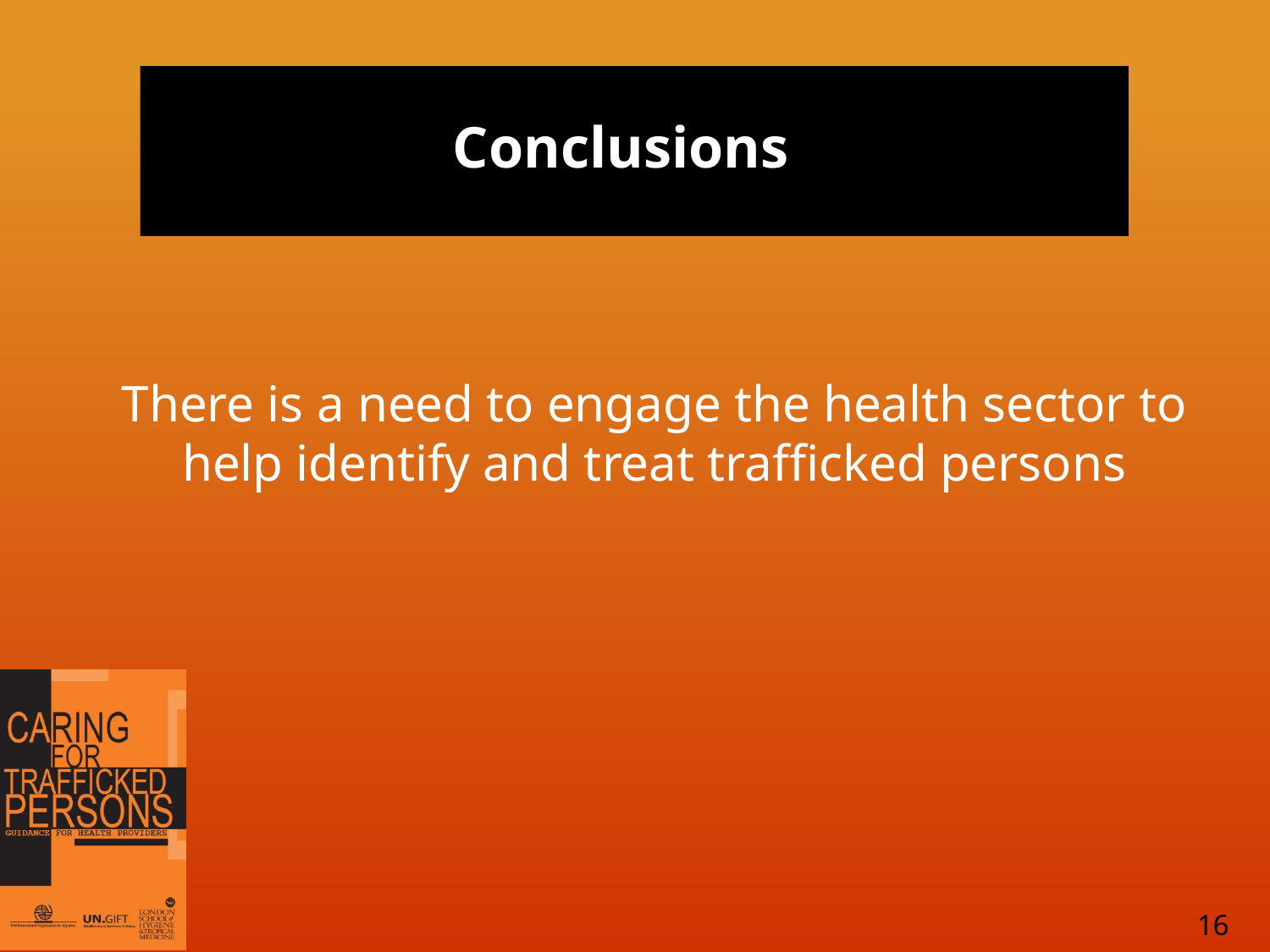

# Conclusions
There is a need to engage the health sector to help identify and treat trafficked persons
16

## Slide 17
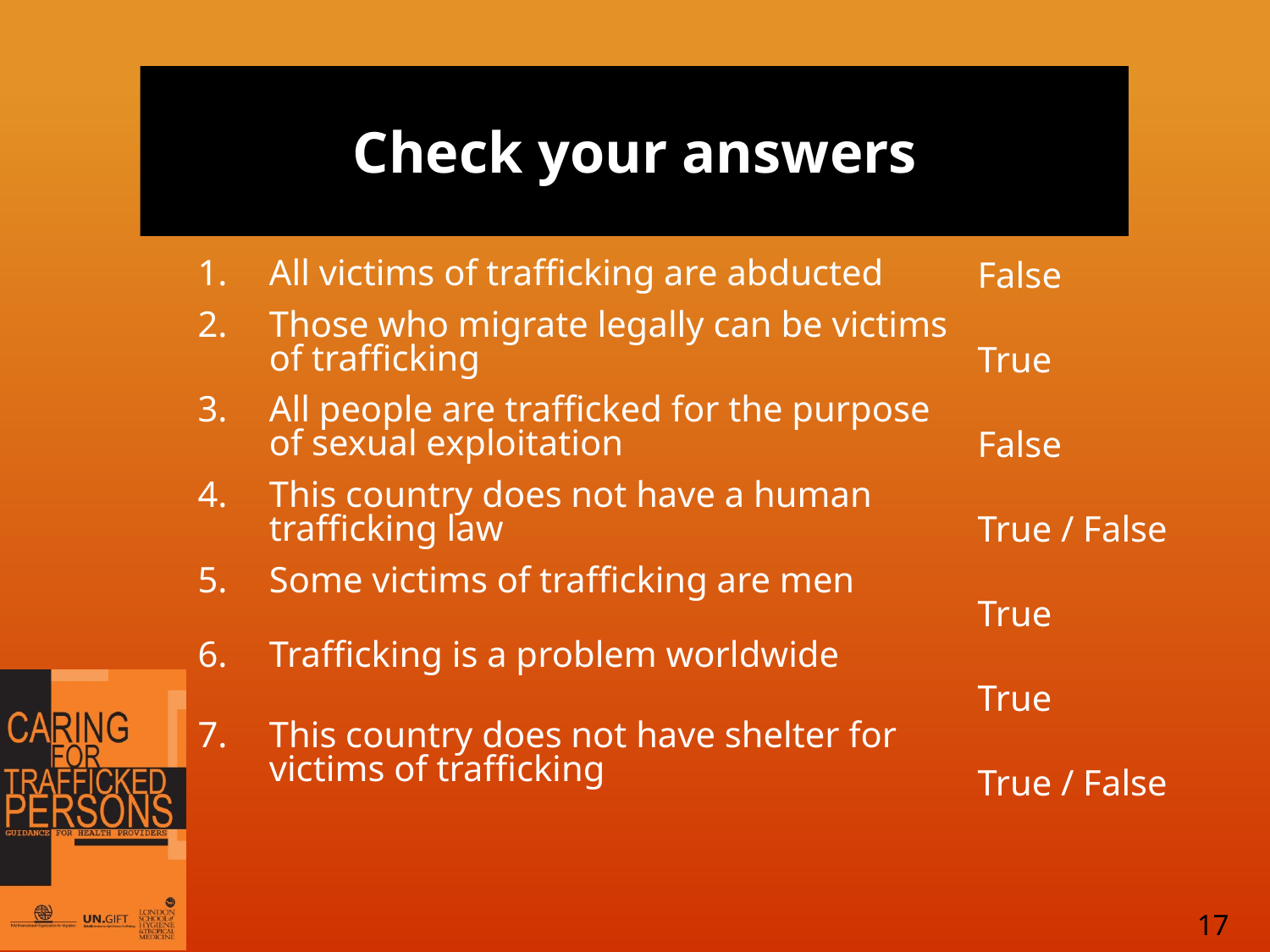

# Check your answers
False
True
False
True / False
True
True
True / False
All victims of trafficking are abducted
Those who migrate legally can be victims of trafficking
All people are trafficked for the purpose of sexual exploitation
This country does not have a human trafficking law
Some victims of trafficking are men
Trafficking is a problem worldwide
This country does not have shelter for victims of trafficking
17

## Slide 18
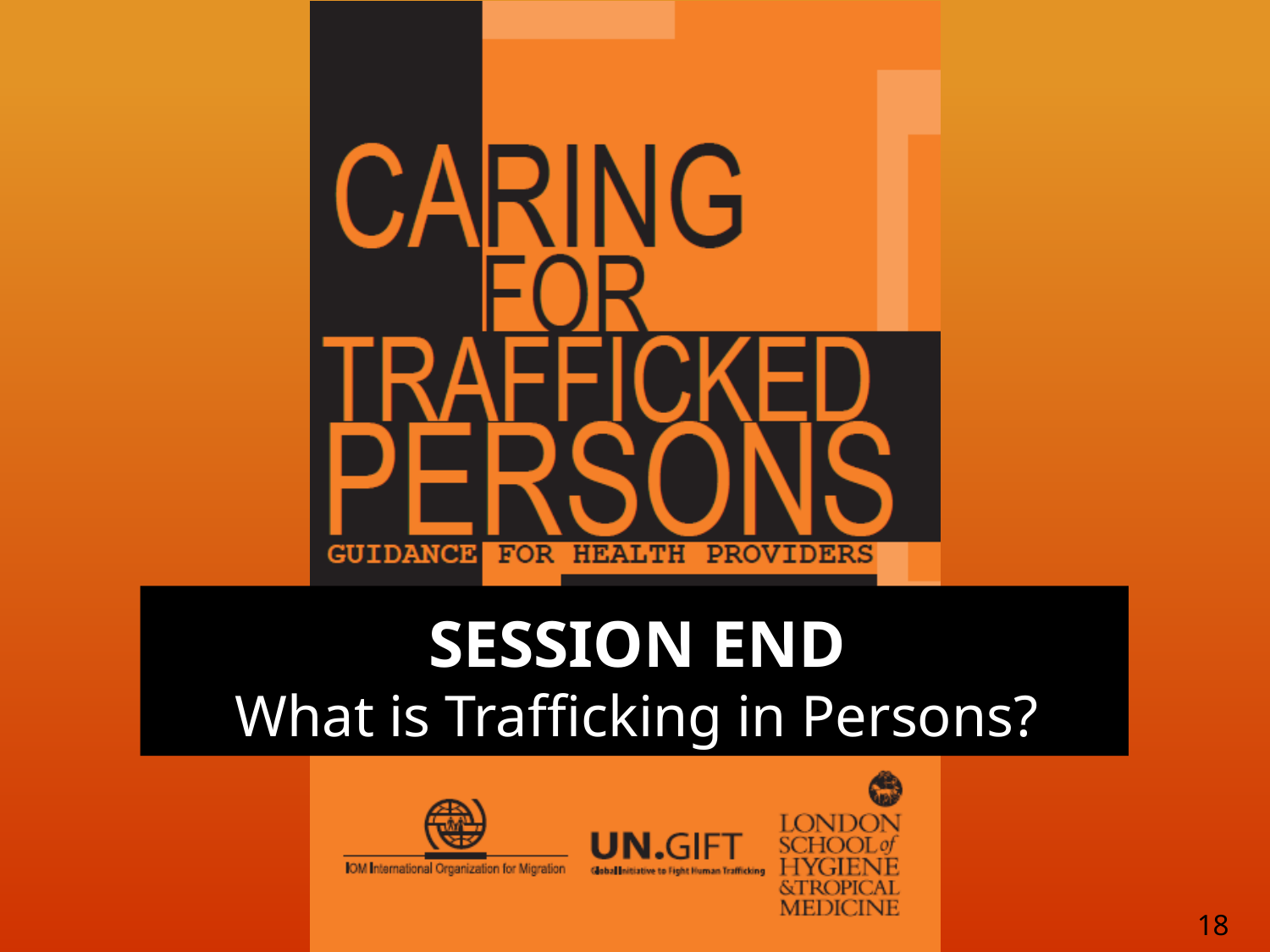

SESSION END
What is Trafficking in Persons?
18
