# Supplementary material for: Health Care Providers and Human Trafficking: What do They Know, What do They Need to Know? Findings from the Middle East, the Caribbean, and Central America
Source: Front Public Health. 2015 Jan 29;3:6. doi: 10.3389/fpubh.2015.00006 (PMC4310216; doi:10.3389/fpubh.2015.00006)
Supplement: Supplementary file 1 [file Presentation_1.ZIP › Caring for Trafficked Persons Training Session 2.pptx]

## Slide 1
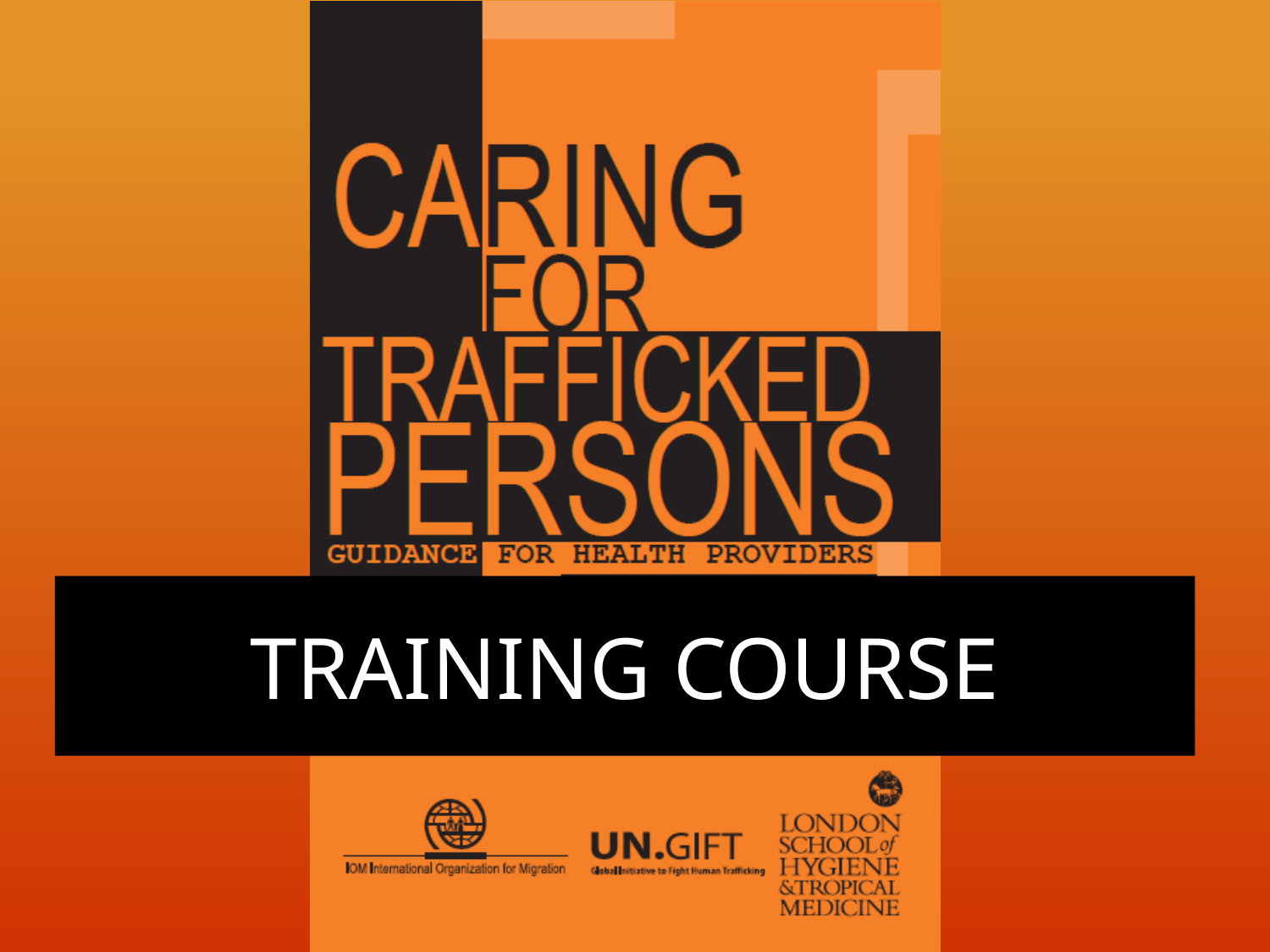

## Slide 2
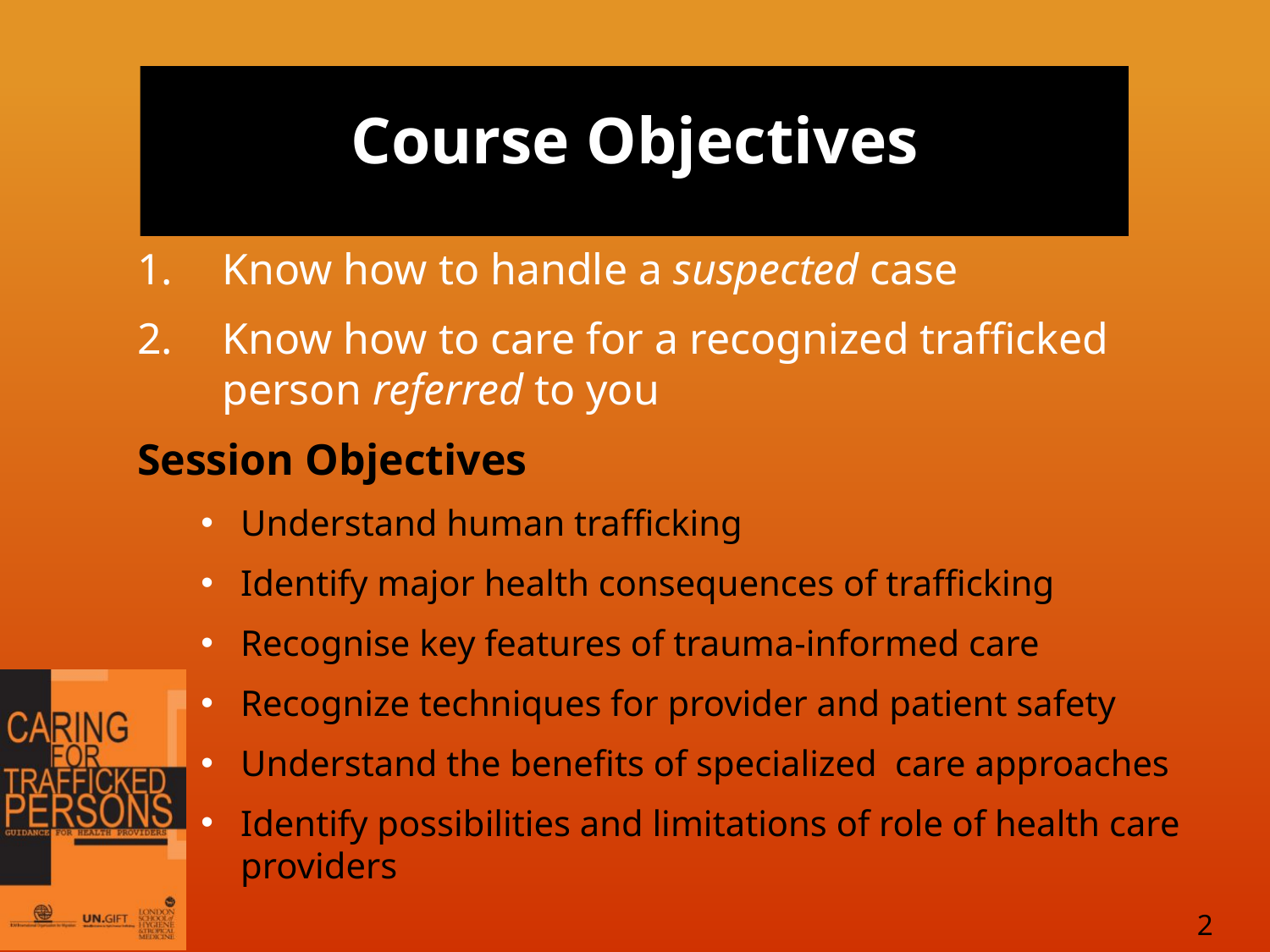

# Course Objectives
Know how to handle a suspected case
Know how to care for a recognized trafficked person referred to you
Session Objectives
Understand human trafficking
Identify major health consequences of trafficking
Recognise key features of trauma-informed care
Recognize techniques for provider and patient safety
Understand the benefits of specialized care approaches
Identify possibilities and limitations of role of health care providers
2

## Slide 3
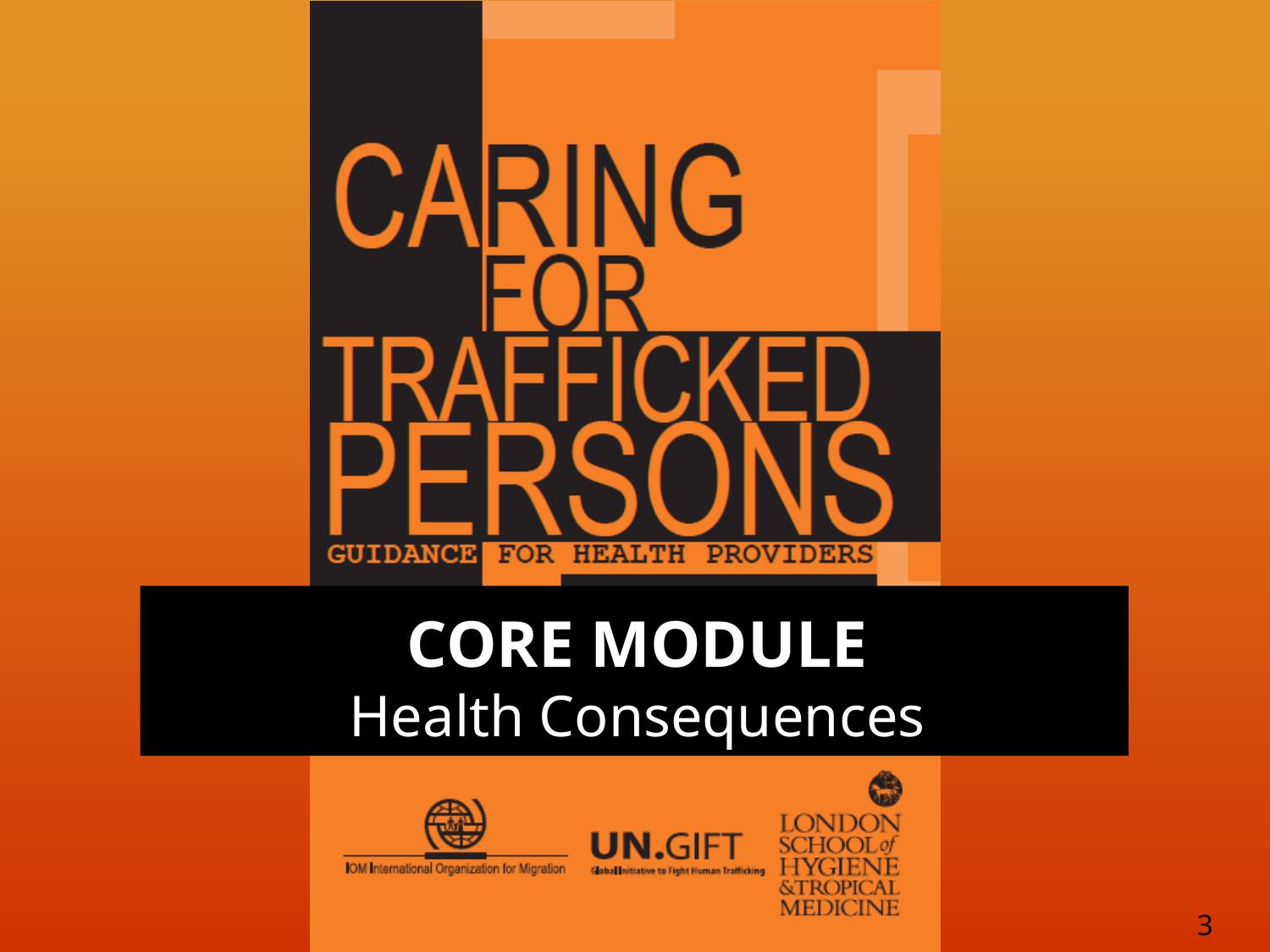

CORE MODULE
Health Consequences
3

## Slide 4
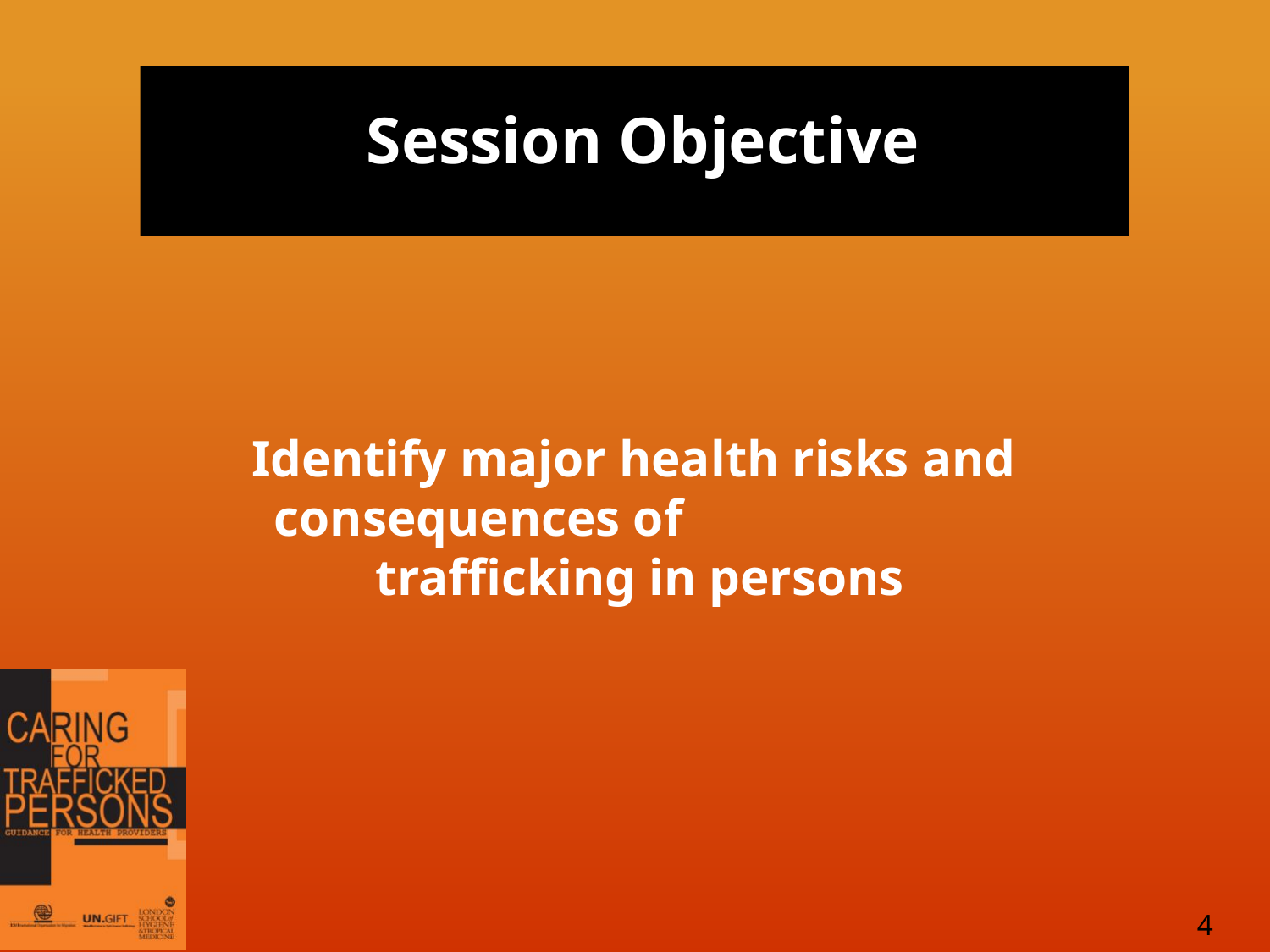

# Session Objective
Identify major health risks and consequences of trafficking in persons
4

## Slide 5
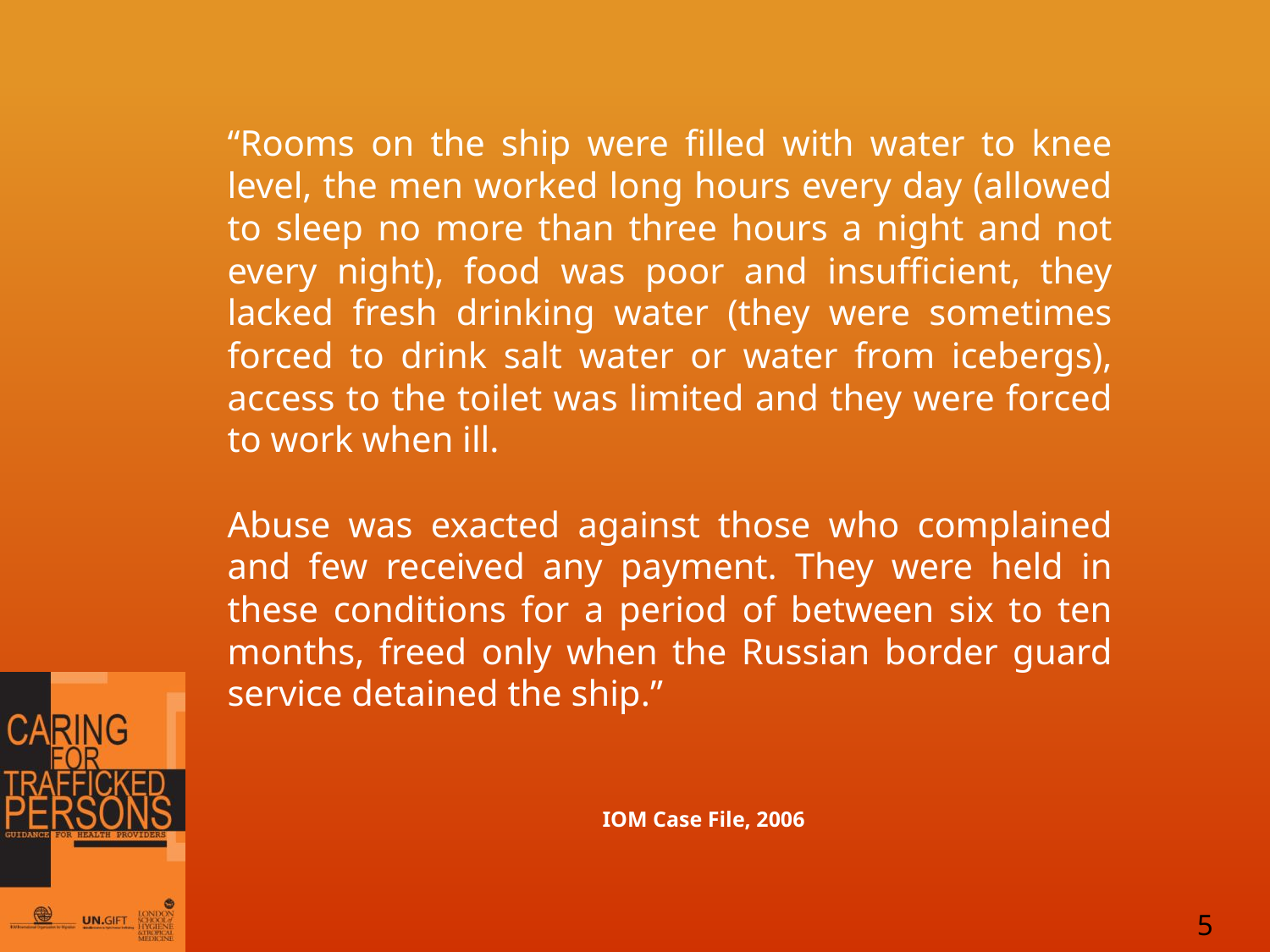

“Rooms on the ship were filled with water to knee level, the men worked long hours every day (allowed to sleep no more than three hours a night and not every night), food was poor and insufficient, they lacked fresh drinking water (they were sometimes forced to drink salt water or water from icebergs), access to the toilet was limited and they were forced to work when ill.
Abuse was exacted against those who complained and few received any payment. They were held in these conditions for a period of between six to ten months, freed only when the Russian border guard service detained the ship.”
IOM Case File, 2006
5

## Slide 6
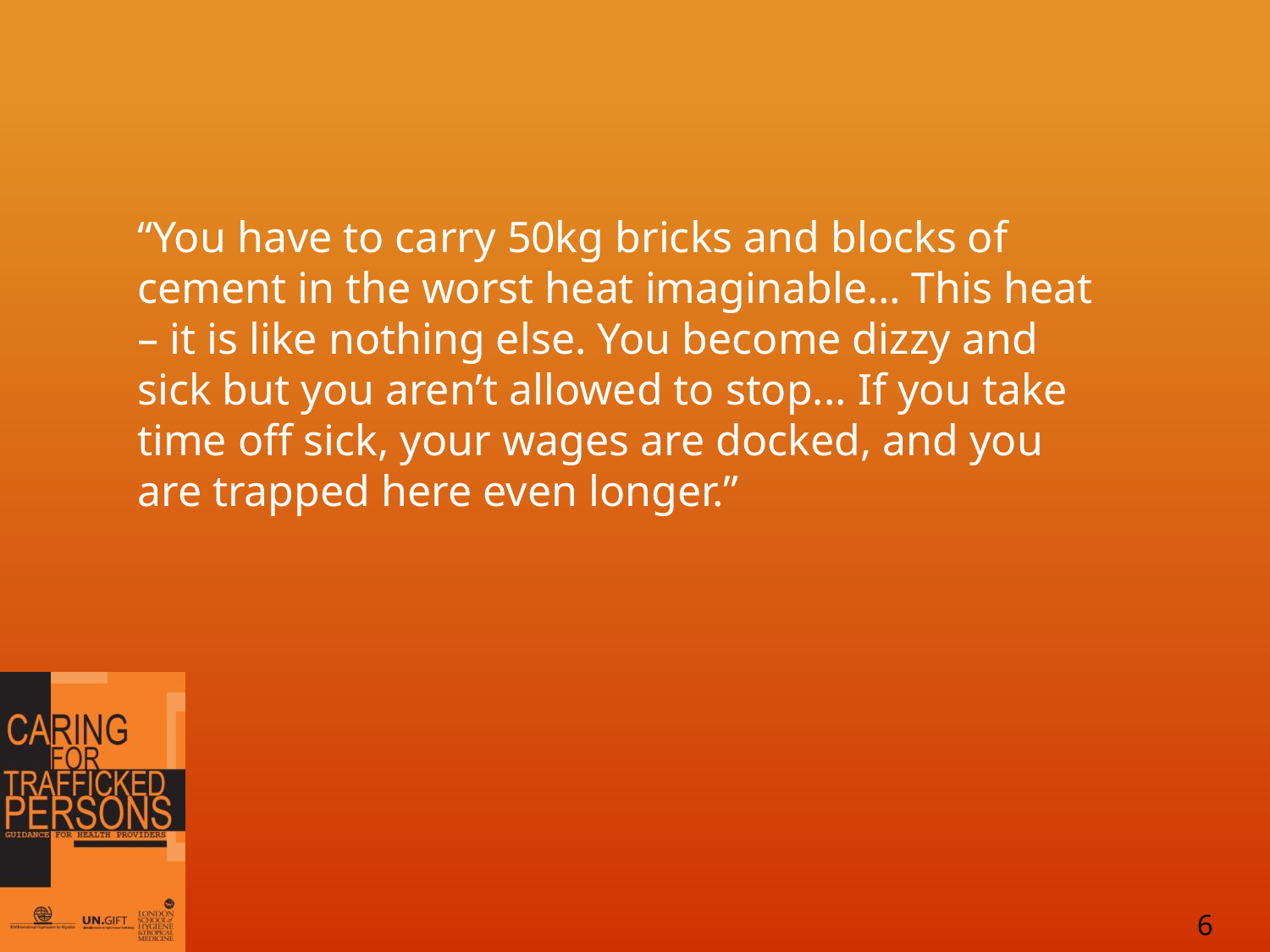

“You have to carry 50kg bricks and blocks of cement in the worst heat imaginable… This heat – it is like nothing else. You become dizzy and sick but you aren’t allowed to stop... If you take time off sick, your wages are docked, and you are trapped here even longer.”
6

## Slide 7
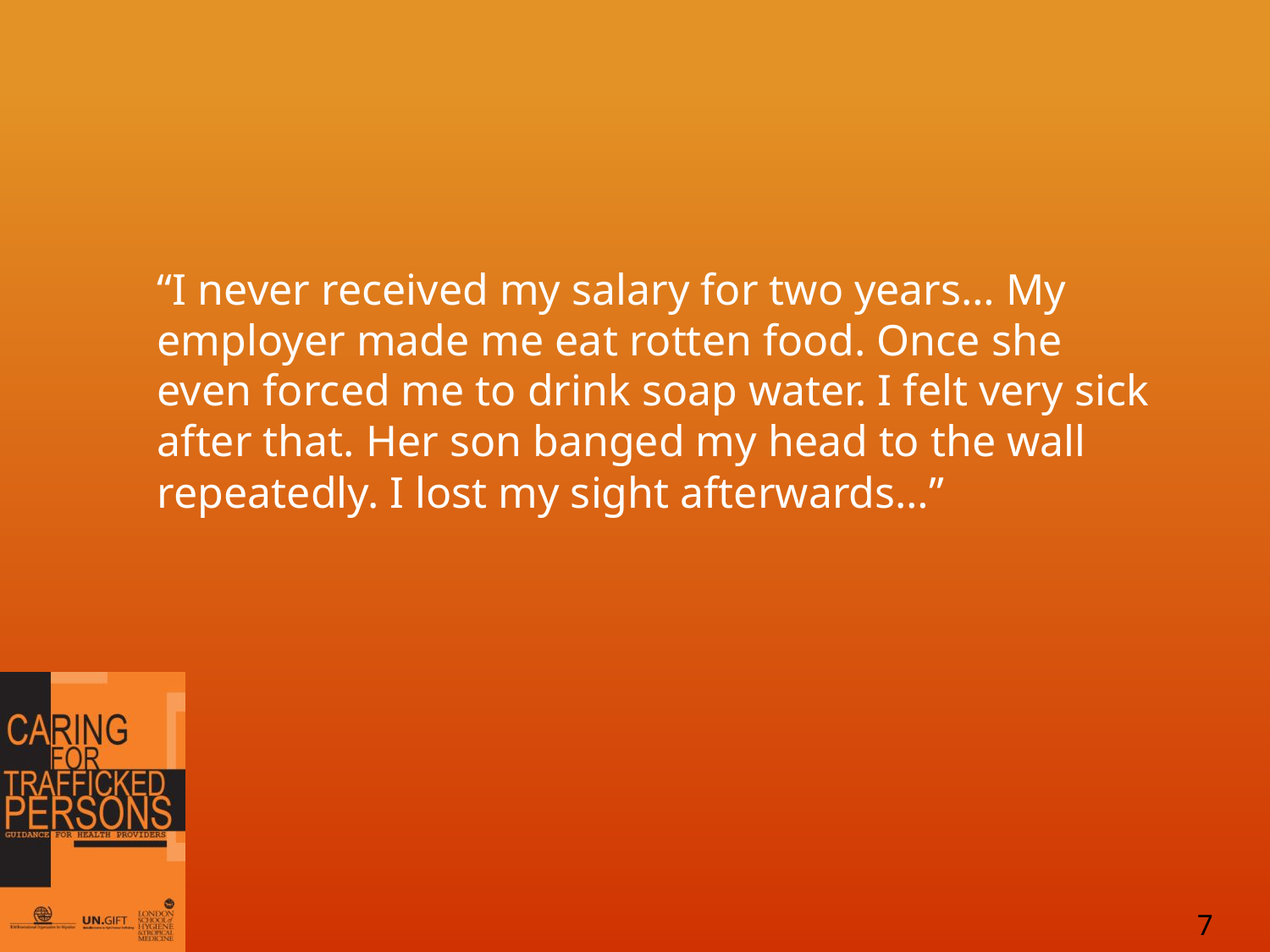

“I never received my salary for two years… My employer made me eat rotten food. Once she even forced me to drink soap water. I felt very sick after that. Her son banged my head to the wall repeatedly. I lost my sight afterwards…”
7

## Slide 8
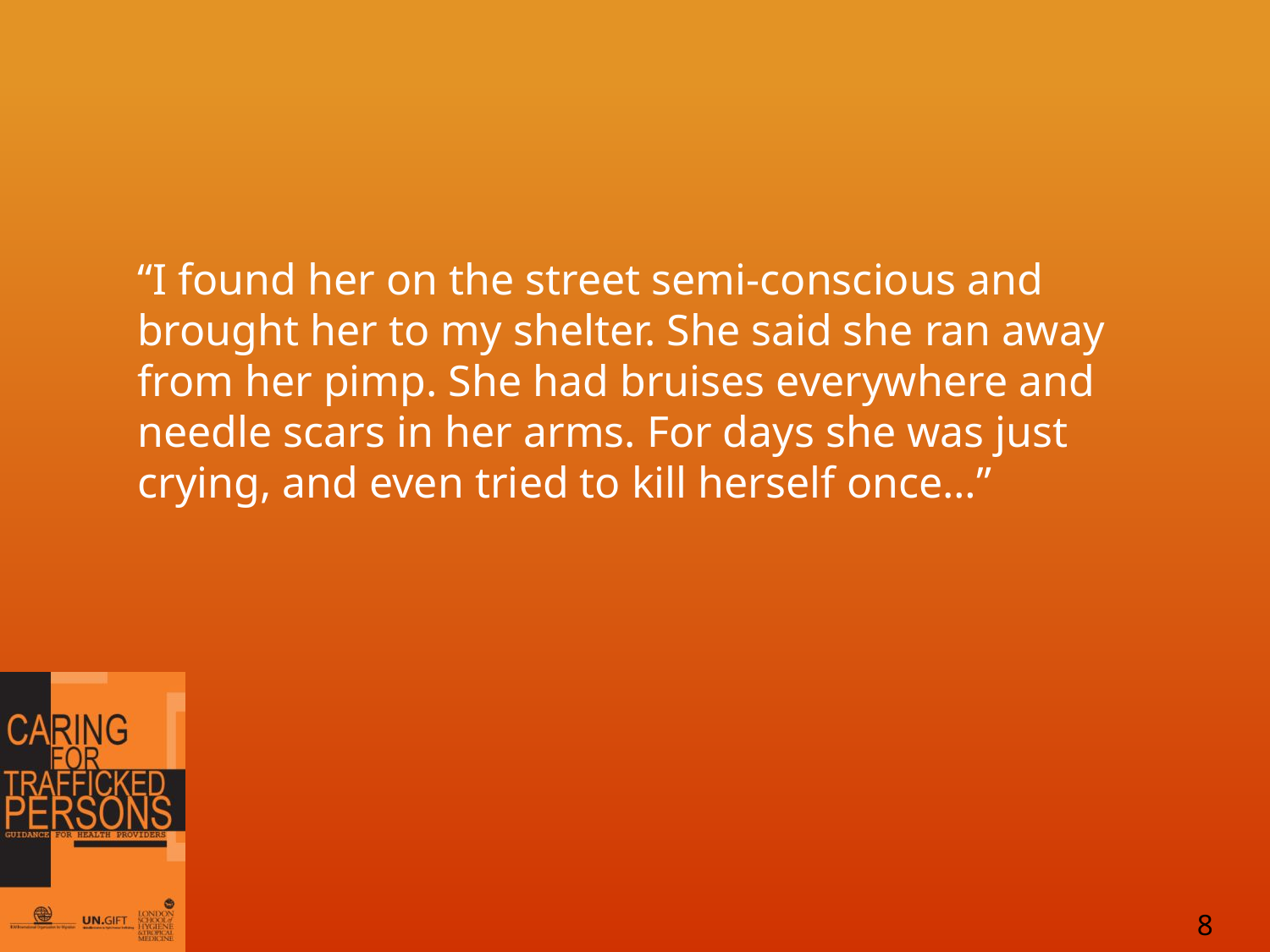

“I found her on the street semi-conscious and brought her to my shelter. She said she ran away from her pimp. She had bruises everywhere and needle scars in her arms. For days she was just crying, and even tried to kill herself once…”
8

## Slide 9
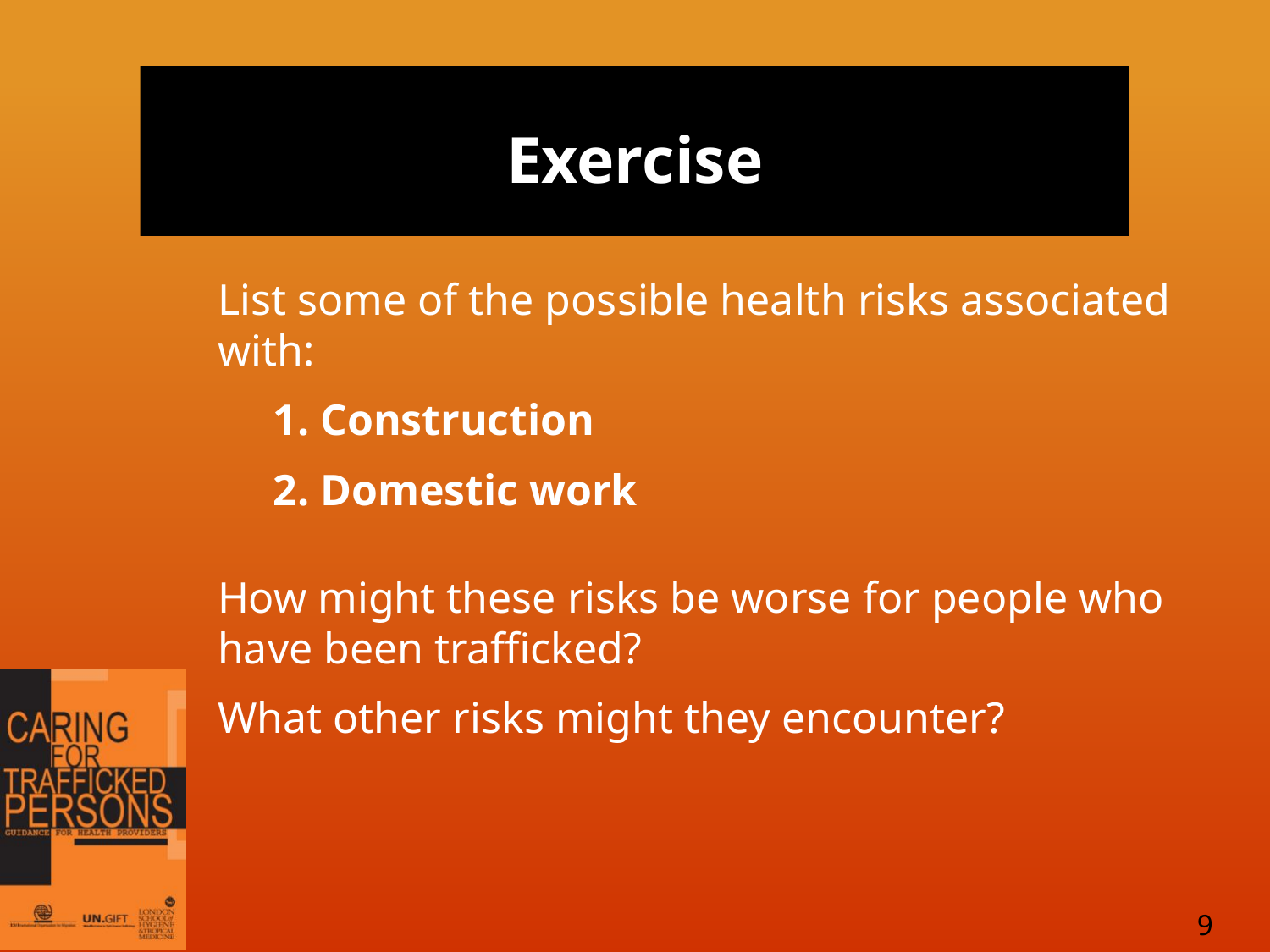

# Exercise
List some of the possible health risks associated with:
1. Construction
2. Domestic work
How might these risks be worse for people who have been trafficked?
What other risks might they encounter?
9

## Slide 10
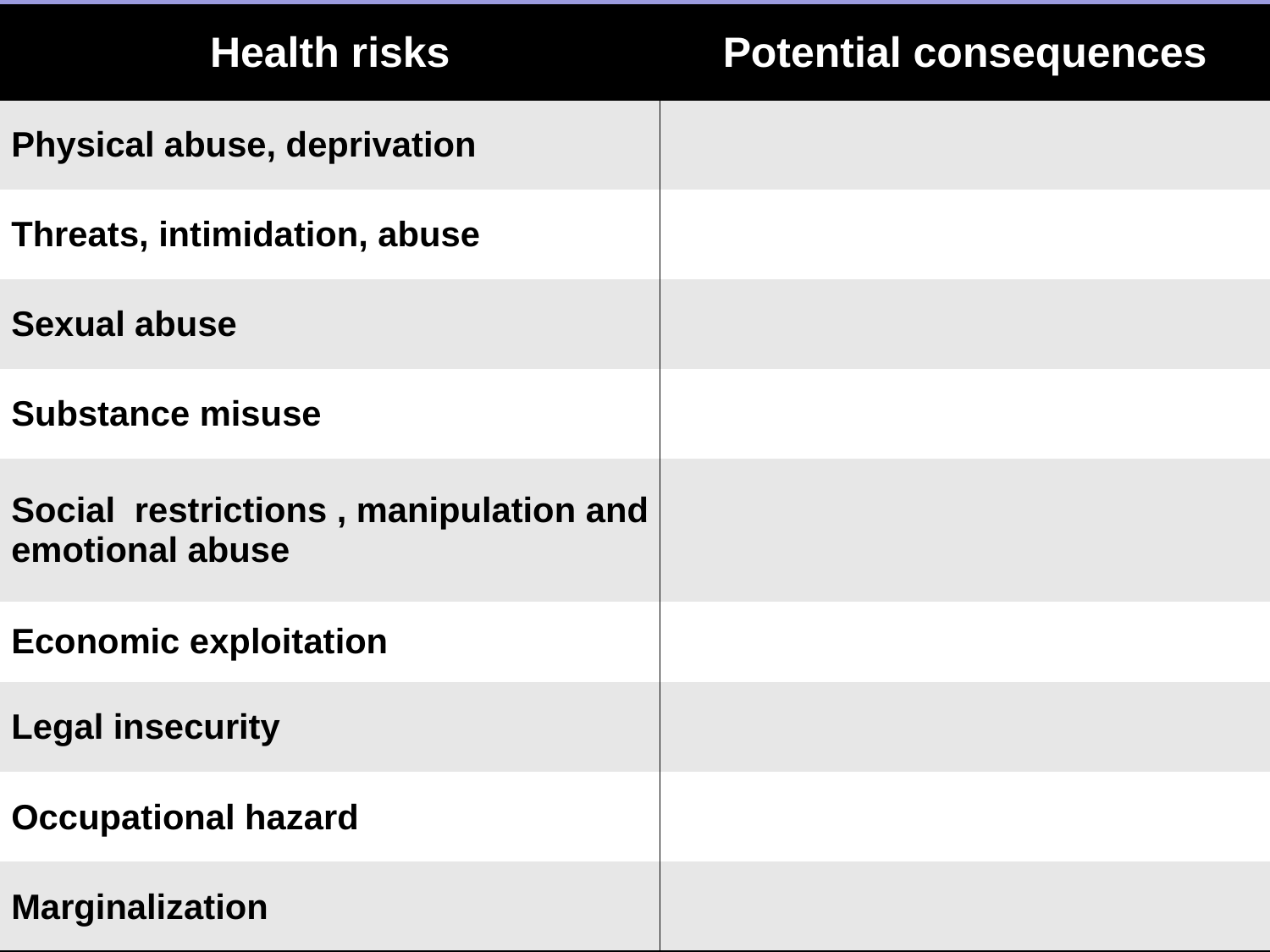

| Health risks | Potential consequences |
| --- | --- |
| Physical abuse, deprivation | |
| Threats, intimidation, abuse | |
| Sexual abuse | |
| Substance misuse | |
| Social restrictions , manipulation and emotional abuse | |
| Economic exploitation | |
| Legal insecurity | |
| Occupational hazard | |
| Marginalization | |
10

## Slide 11
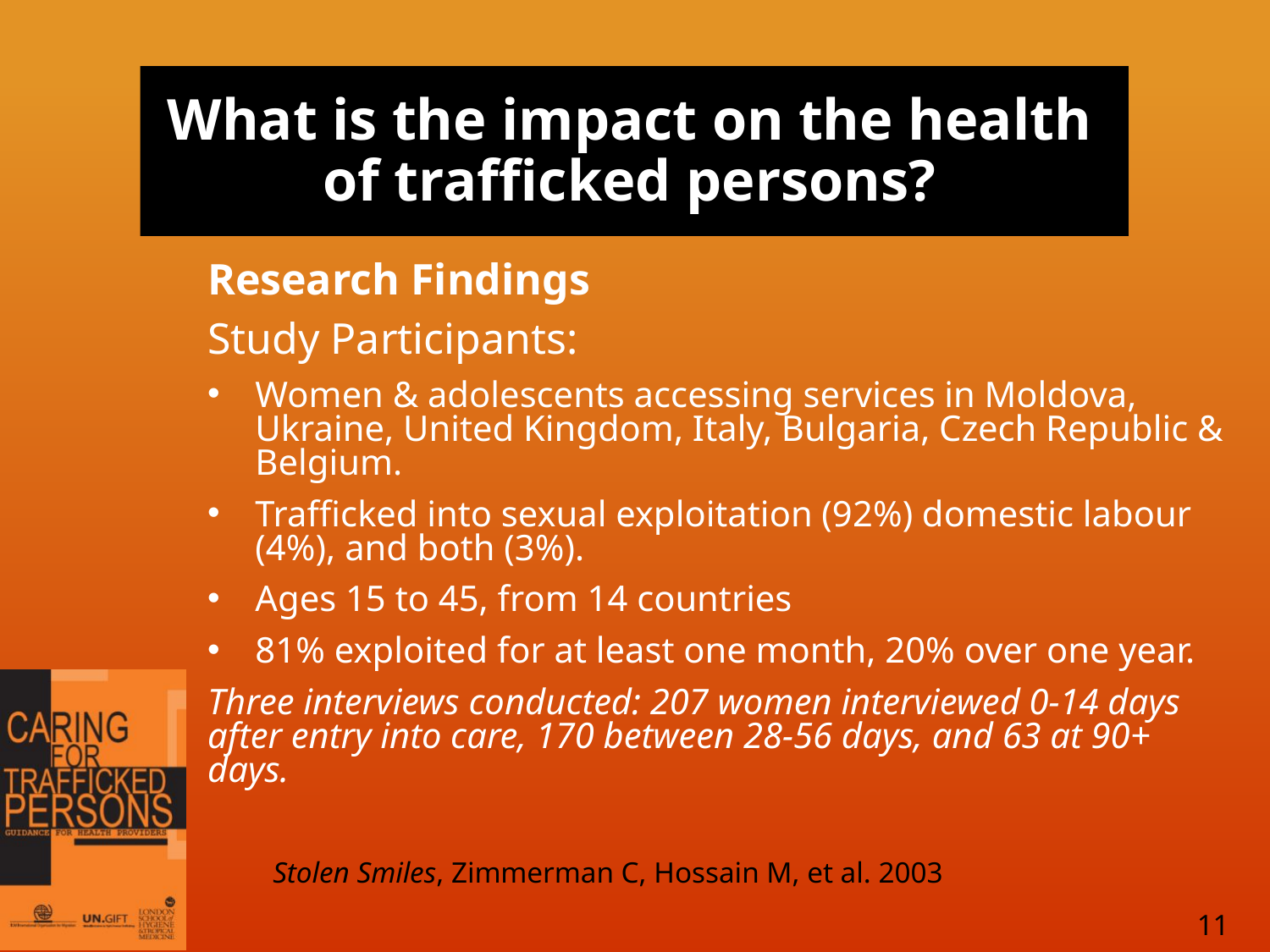

# What is the impact on the health of trafficked persons?
Research Findings
Study Participants:
Women & adolescents accessing services in Moldova, Ukraine, United Kingdom, Italy, Bulgaria, Czech Republic & Belgium.
Trafficked into sexual exploitation (92%) domestic labour (4%), and both (3%).
Ages 15 to 45, from 14 countries
81% exploited for at least one month, 20% over one year.
Three interviews conducted: 207 women interviewed 0-14 days after entry into care, 170 between 28-56 days, and 63 at 90+ days.
Stolen Smiles, Zimmerman C, Hossain M, et al. 2003
11

## Slide 12
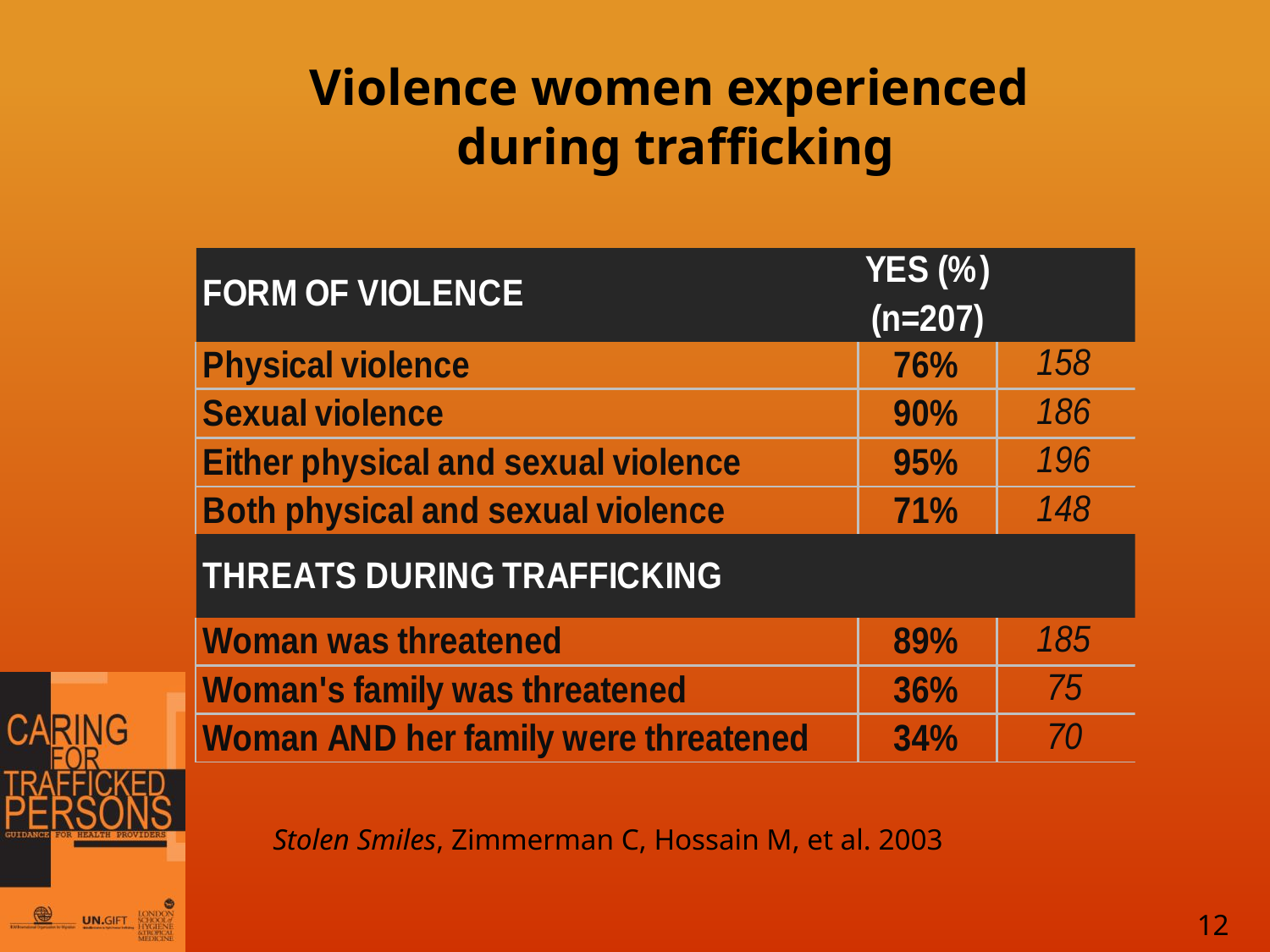

Violence women experienced
during trafficking
Stolen Smiles, Zimmerman C, Hossain M, et al. 2003
12

## Slide 13
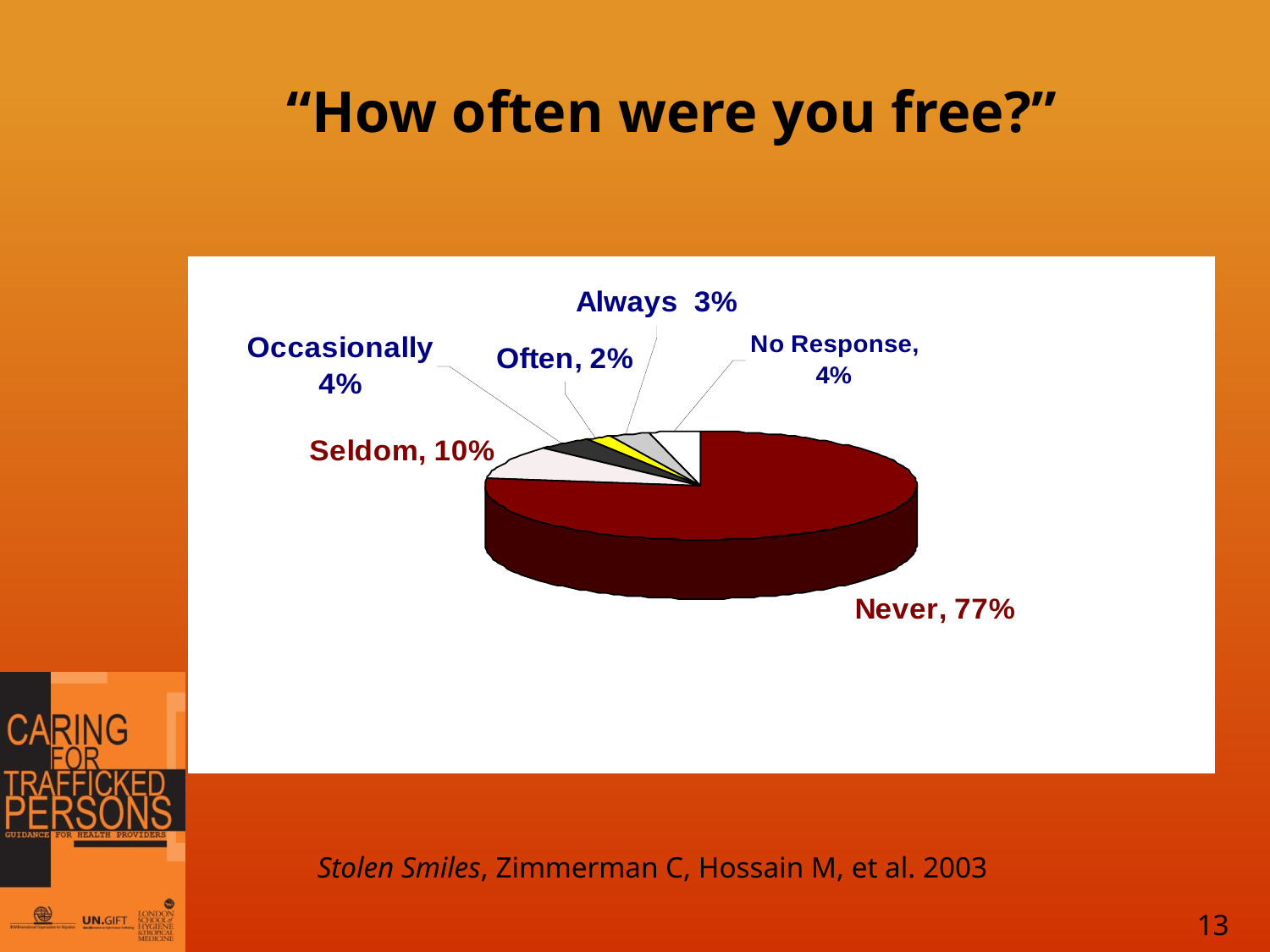

“How often were you free?”
Stolen Smiles, Zimmerman C, Hossain M, et al. 2003
13

## Slide 14
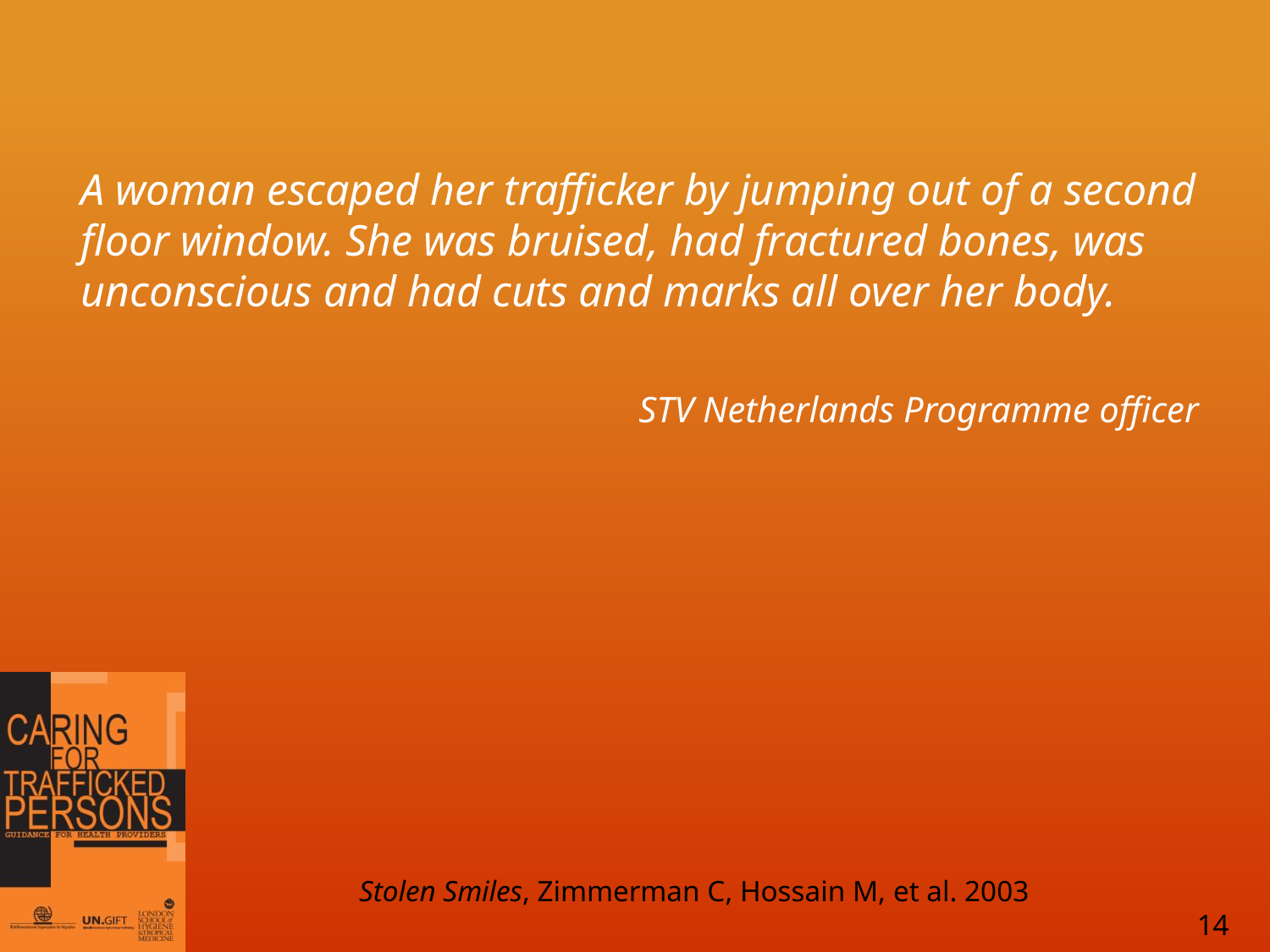

A woman escaped her trafficker by jumping out of a second floor window. She was bruised, had fractured bones, was unconscious and had cuts and marks all over her body.
STV Netherlands Programme officer
Stolen Smiles, Zimmerman C, Hossain M, et al. 2003
14

## Slide 15
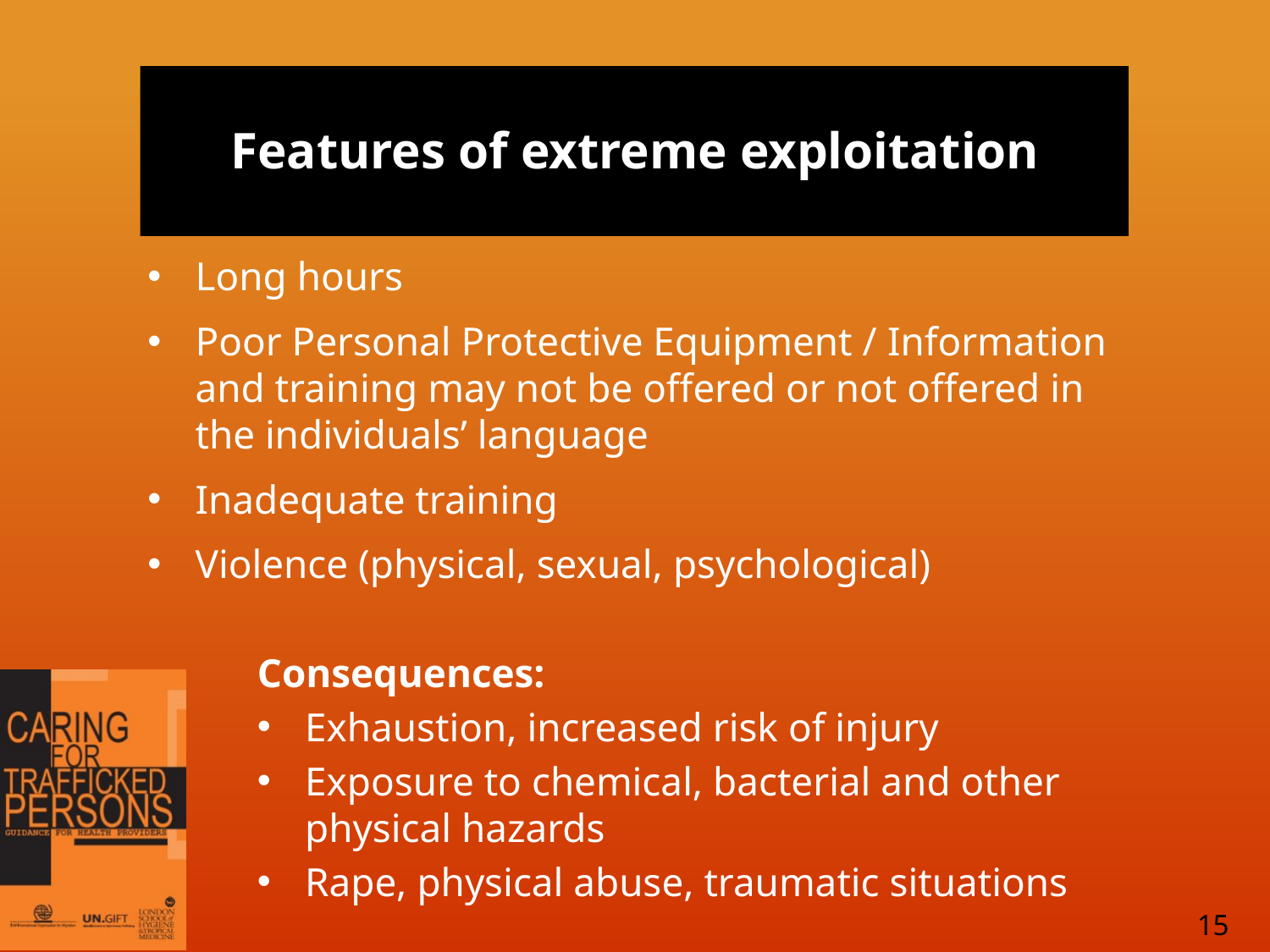

# Features of extreme exploitation
Long hours
Poor Personal Protective Equipment / Information and training may not be offered or not offered in the individuals’ language
Inadequate training
Violence (physical, sexual, psychological)
Consequences:
Exhaustion, increased risk of injury
Exposure to chemical, bacterial and other physical hazards
Rape, physical abuse, traumatic situations
15

## Slide 16
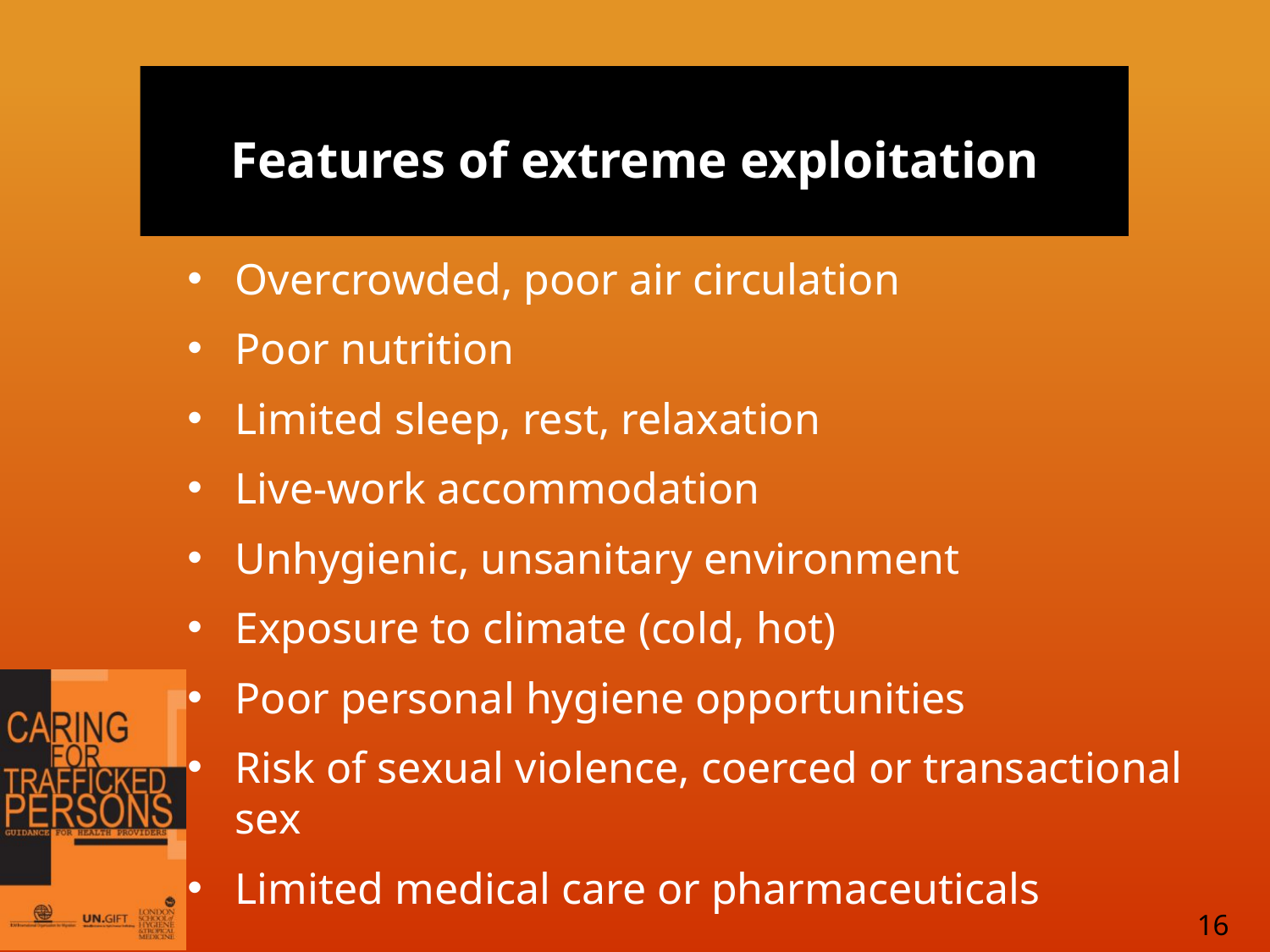

# Features of extreme exploitation
Overcrowded, poor air circulation
Poor nutrition
Limited sleep, rest, relaxation
Live-work accommodation
Unhygienic, unsanitary environment
Exposure to climate (cold, hot)
Poor personal hygiene opportunities
Risk of sexual violence, coerced or transactional sex
Limited medical care or pharmaceuticals
16

## Slide 17
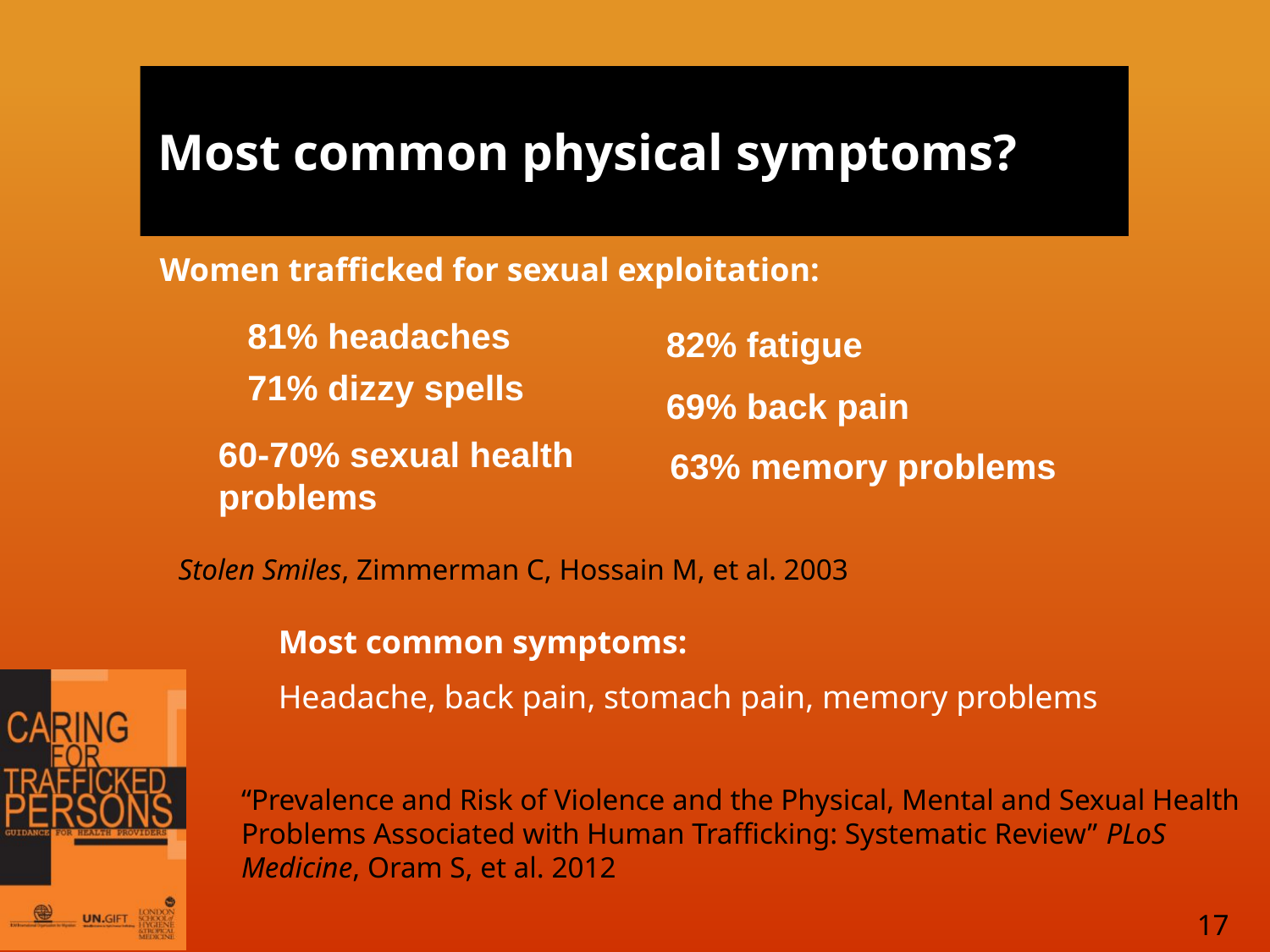

# Most common physical symptoms?
Women trafficked for sexual exploitation:
81% headaches
71% dizzy spells
82% fatigue
69% back pain
60-70% sexual health problems
63% memory problems
Stolen Smiles, Zimmerman C, Hossain M, et al. 2003
Most common symptoms:
Headache, back pain, stomach pain, memory problems
“Prevalence and Risk of Violence and the Physical, Mental and Sexual Health Problems Associated with Human Trafficking: Systematic Review” PLoS Medicine, Oram S, et al. 2012
17

## Slide 18
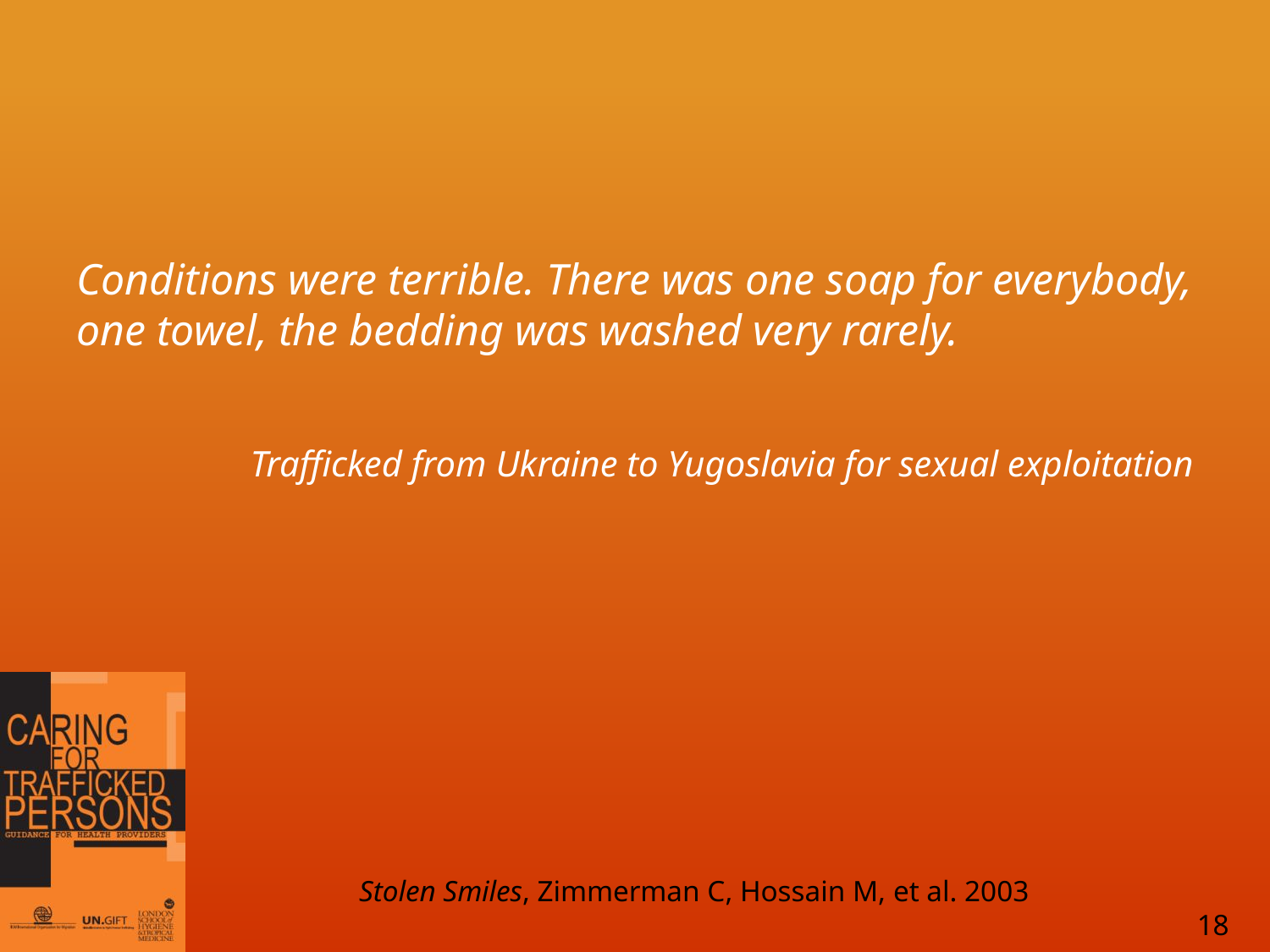

Conditions were terrible. There was one soap for everybody, one towel, the bedding was washed very rarely.
Trafficked from Ukraine to Yugoslavia for sexual exploitation
Stolen Smiles, Zimmerman C, Hossain M, et al. 2003
18

## Slide 19
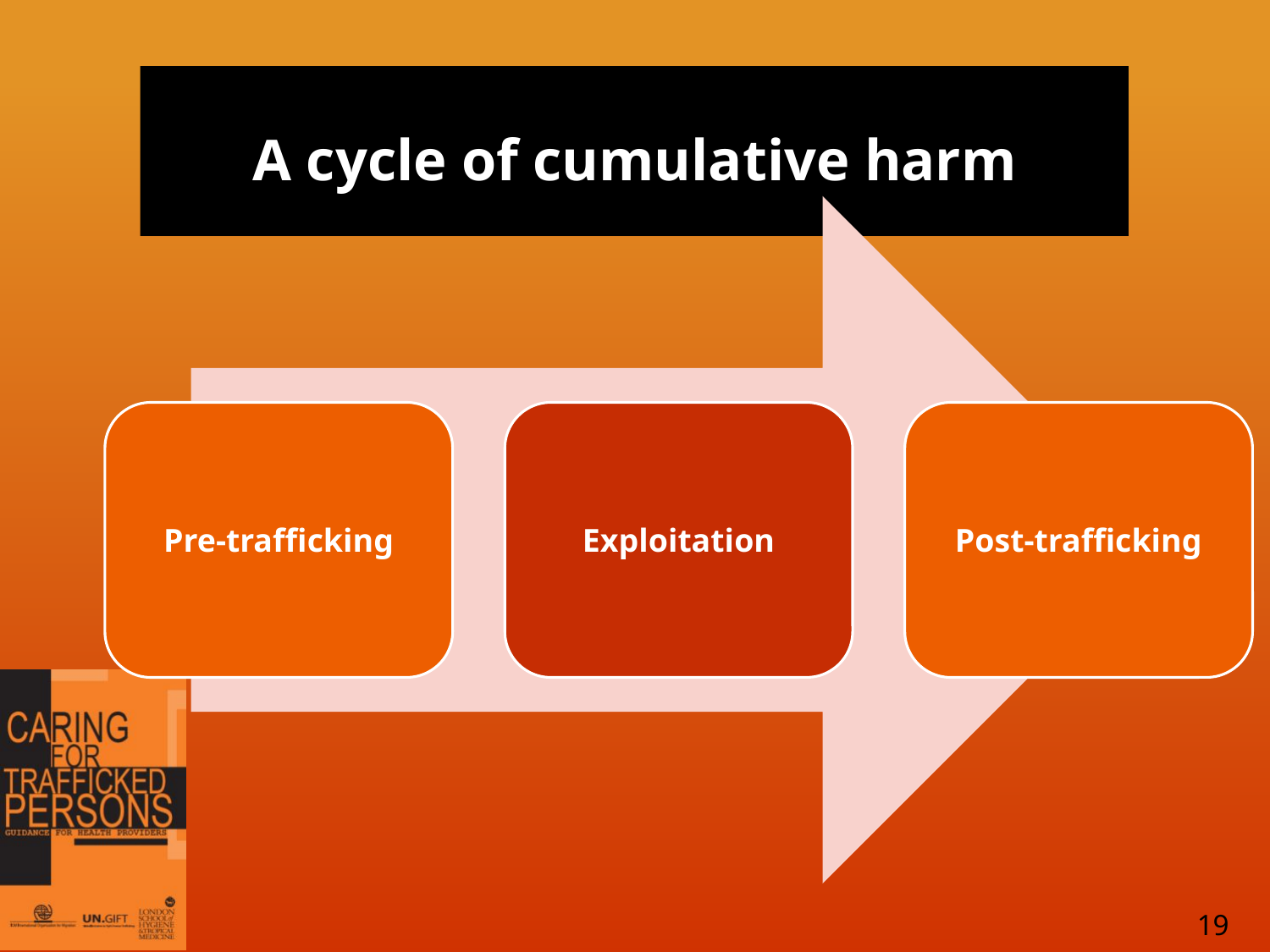

# A cycle of cumulative harm
19

## Slide 20
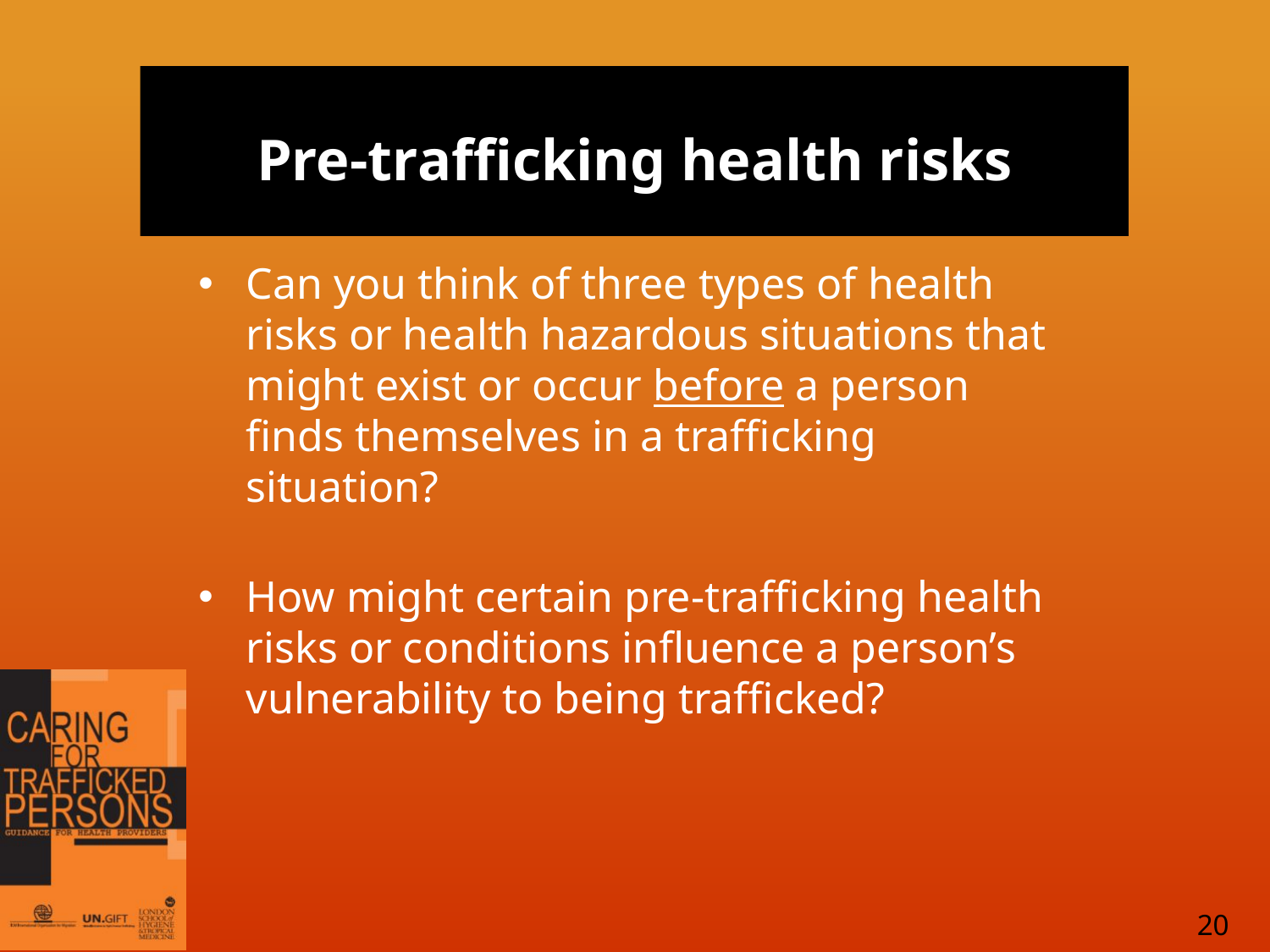

# Pre-trafficking health risks
Can you think of three types of health risks or health hazardous situations that might exist or occur before a person finds themselves in a trafficking situation?
How might certain pre-trafficking health risks or conditions influence a person’s vulnerability to being trafficked?
20

## Slide 21
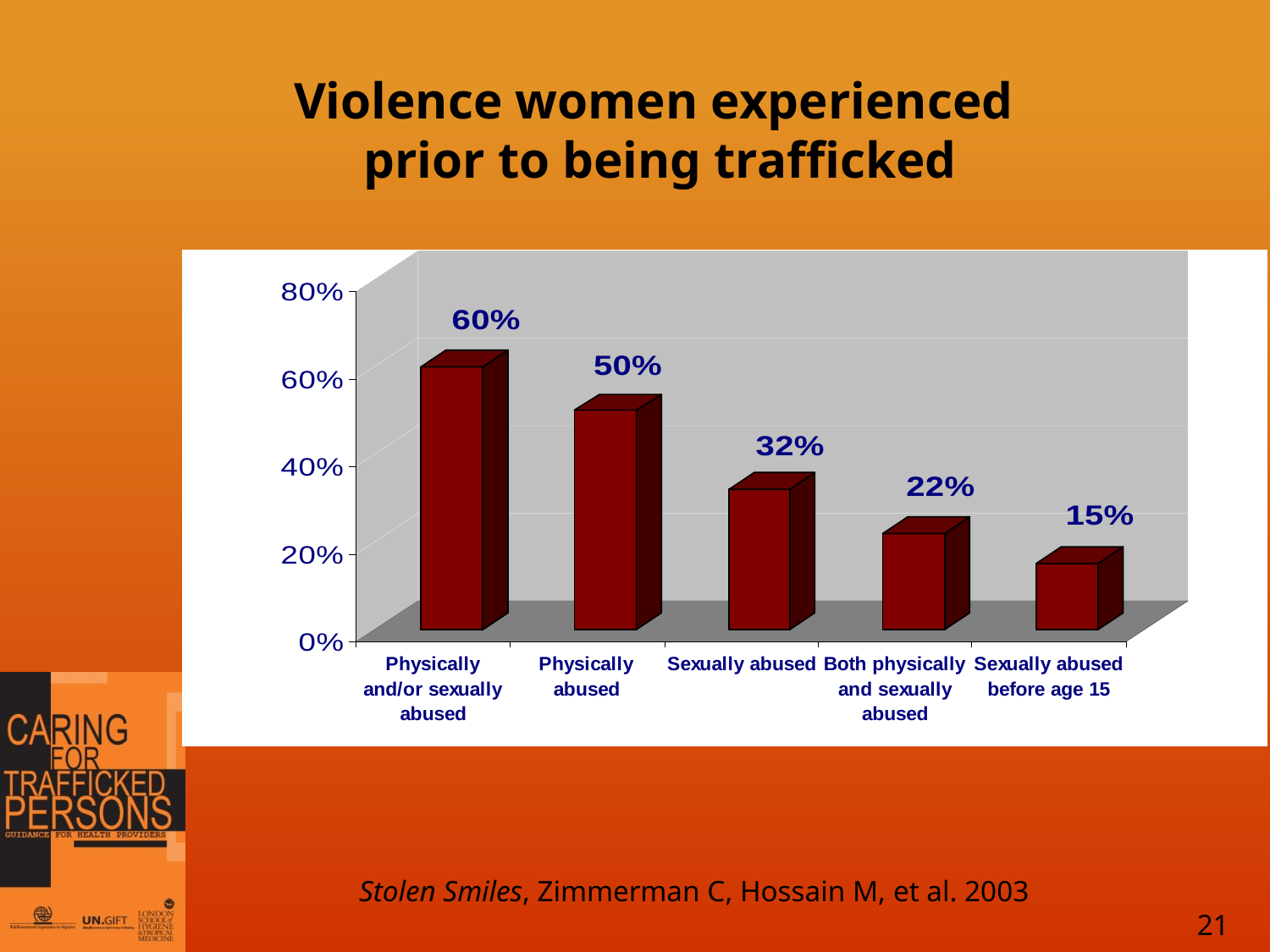

Violence women experienced
prior to being trafficked
Stolen Smiles, Zimmerman C, Hossain M, et al. 2003
21

## Slide 22
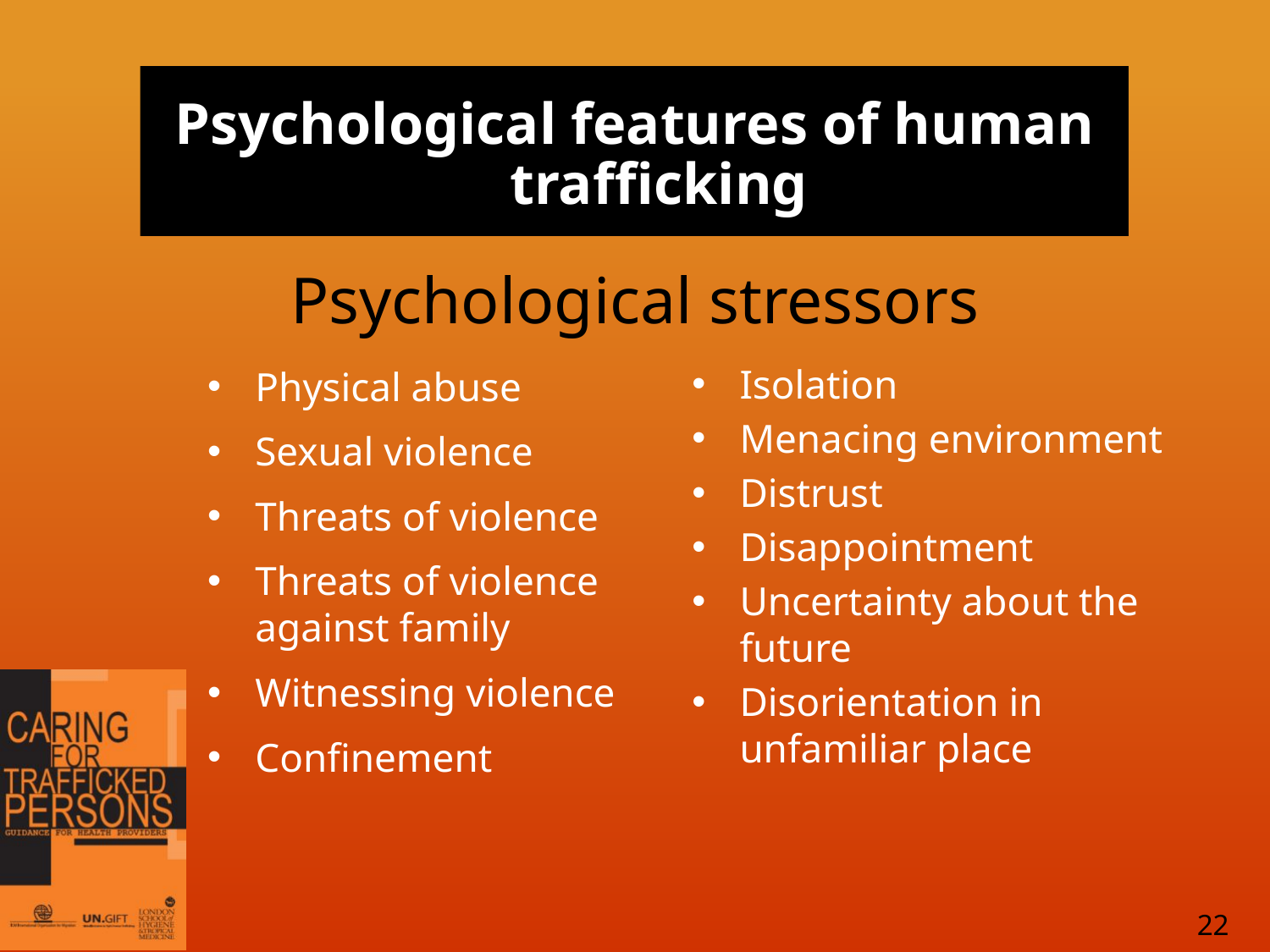

Psychological features of human trafficking
# Psychological stressors
Isolation
Menacing environment
Distrust
Disappointment
Uncertainty about the future
Disorientation in unfamiliar place
Physical abuse
Sexual violence
Threats of violence
Threats of violence against family
Witnessing violence
Confinement
22

## Slide 23
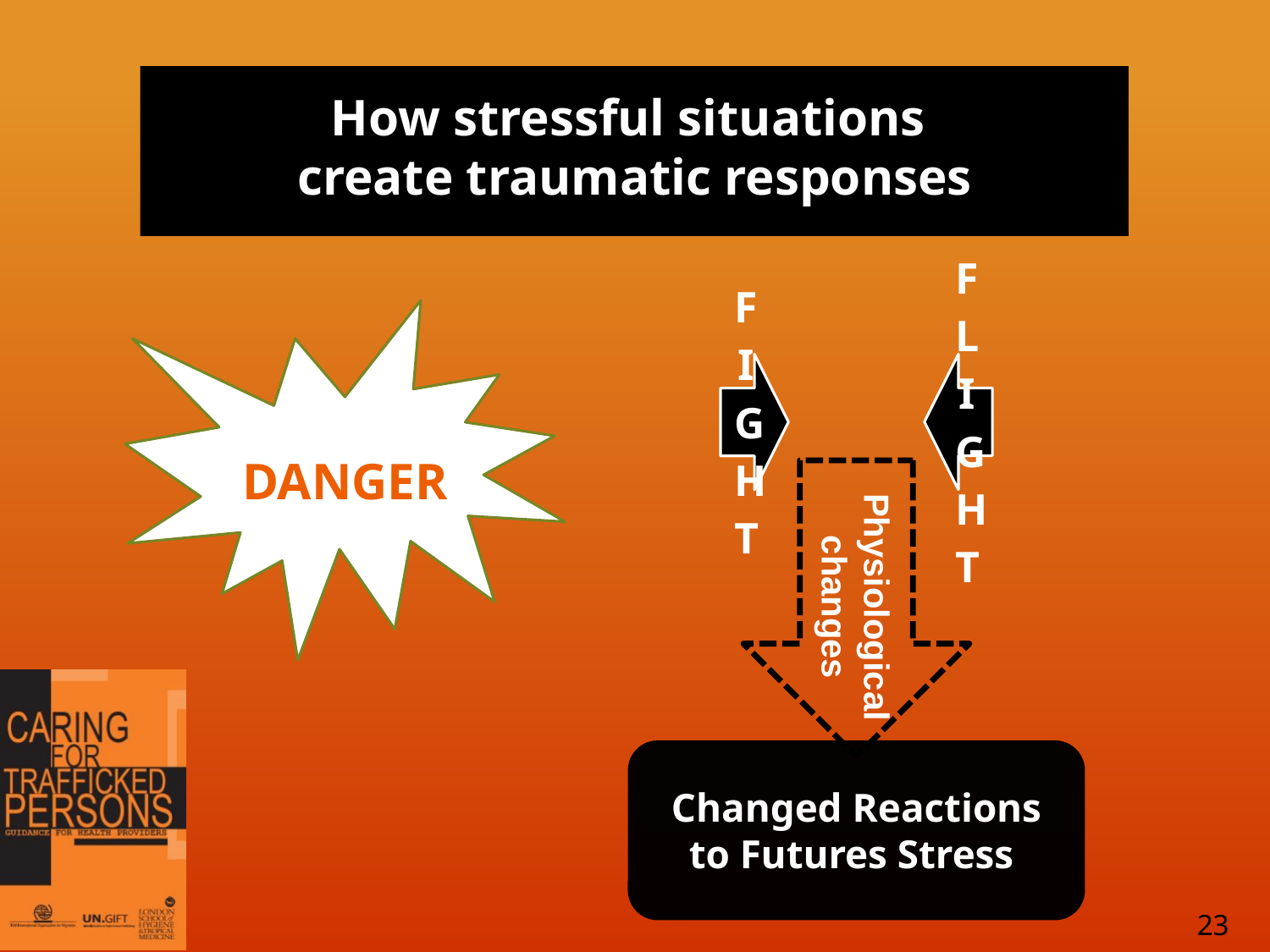

# How stressful situations create traumatic responses
DANGER
Physiological changes
Changed Reactions to Futures Stress
23

## Slide 24
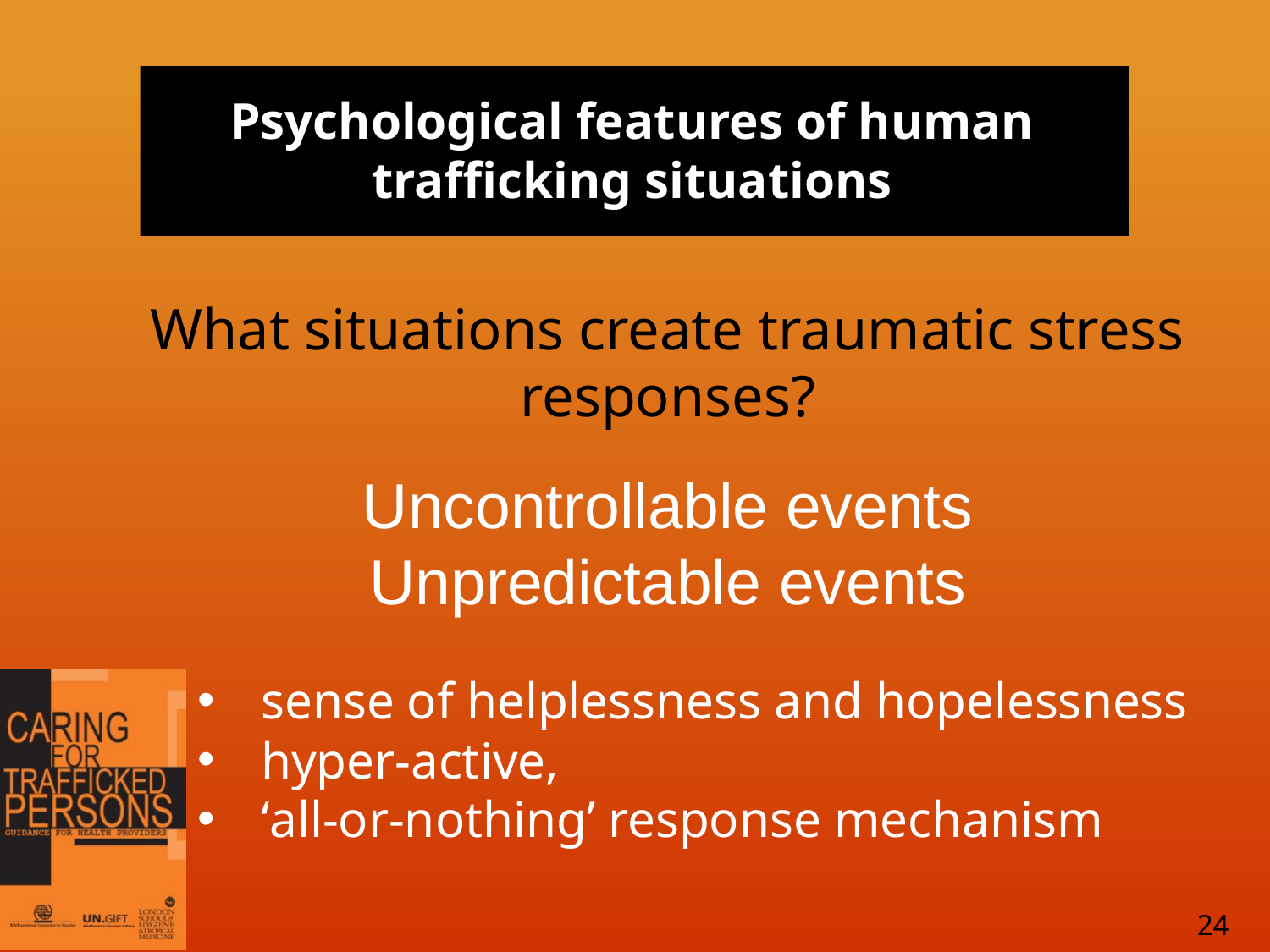

# Psychological features of human trafficking situations
What situations create traumatic stress responses?
Uncontrollable events
Unpredictable events
sense of helplessness and hopelessness
hyper-active,
‘all-or-nothing’ response mechanism
24

## Slide 25
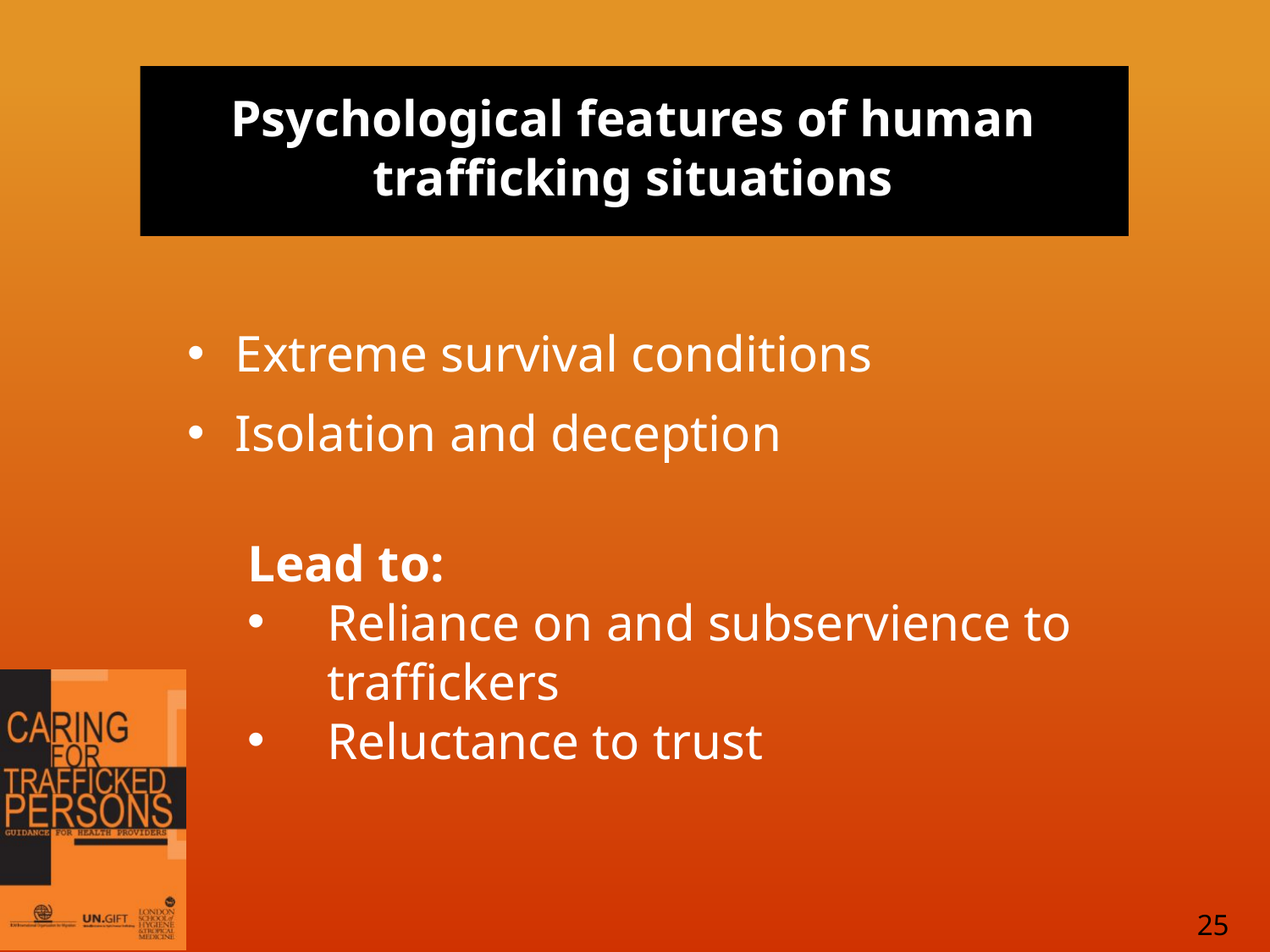

# Psychological features of human trafficking situations
Extreme survival conditions
Isolation and deception
Lead to:
Reliance on and subservience to traffickers
Reluctance to trust
25

## Slide 26
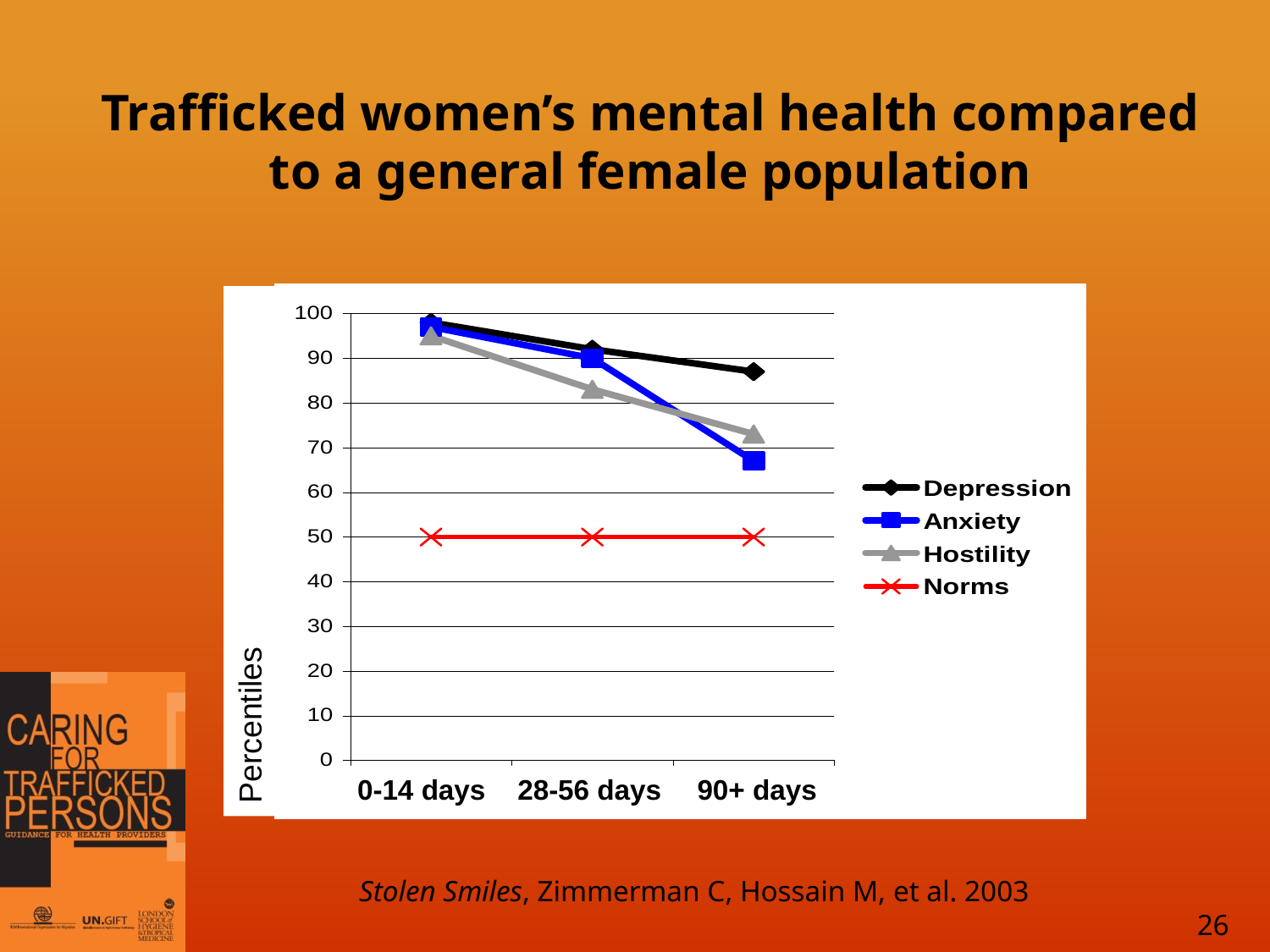

Trafficked women’s mental health compared to a general female population
Percentiles
0-14 days
28-56 days
90+ days
Stolen Smiles, Zimmerman C, Hossain M, et al. 2003
26

## Slide 27
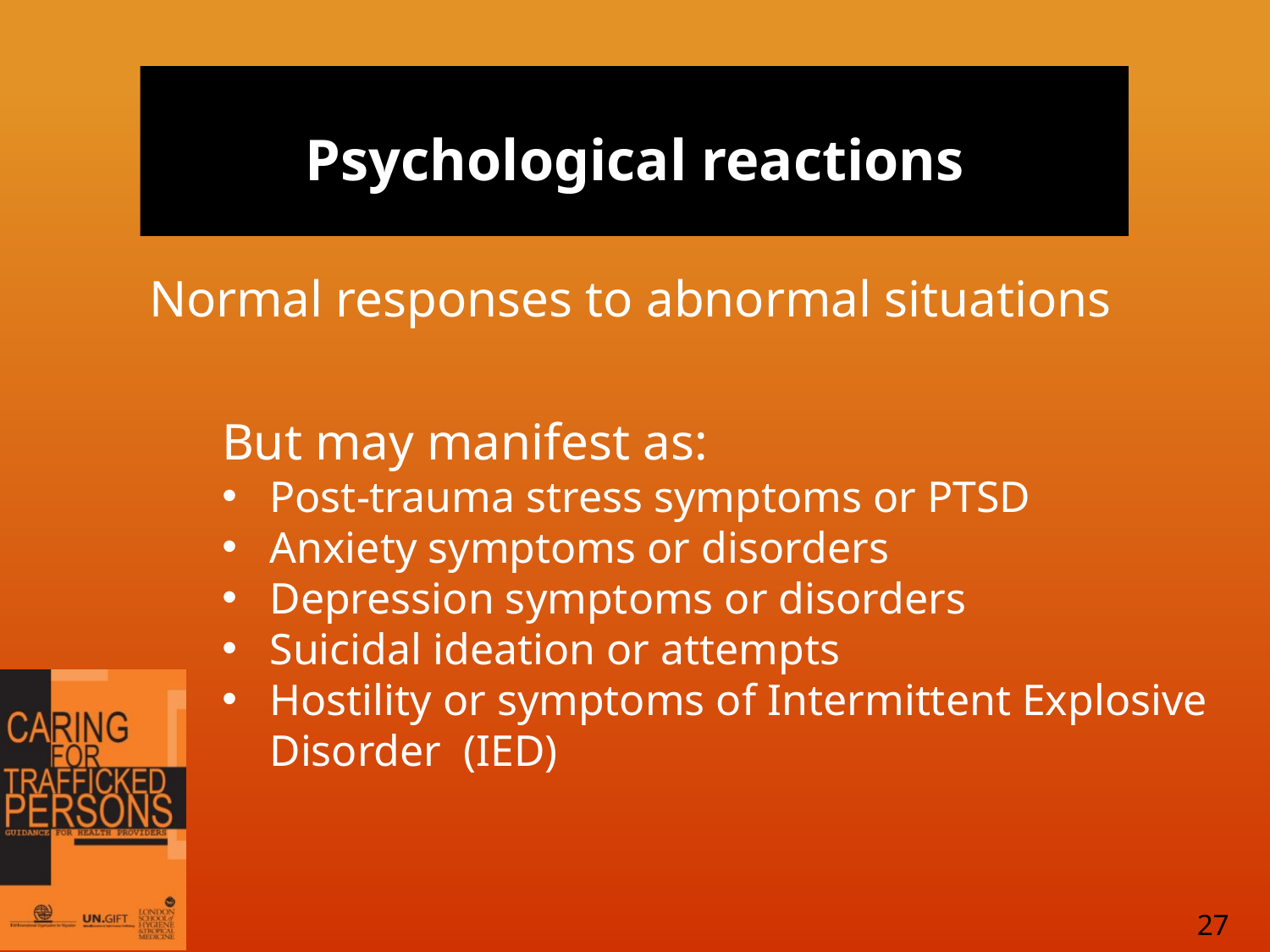

# Psychological reactions
Normal responses to abnormal situations
But may manifest as:
Post-trauma stress symptoms or PTSD
Anxiety symptoms or disorders
Depression symptoms or disorders
Suicidal ideation or attempts
Hostility or symptoms of Intermittent Explosive Disorder (IED)
27

## Slide 28
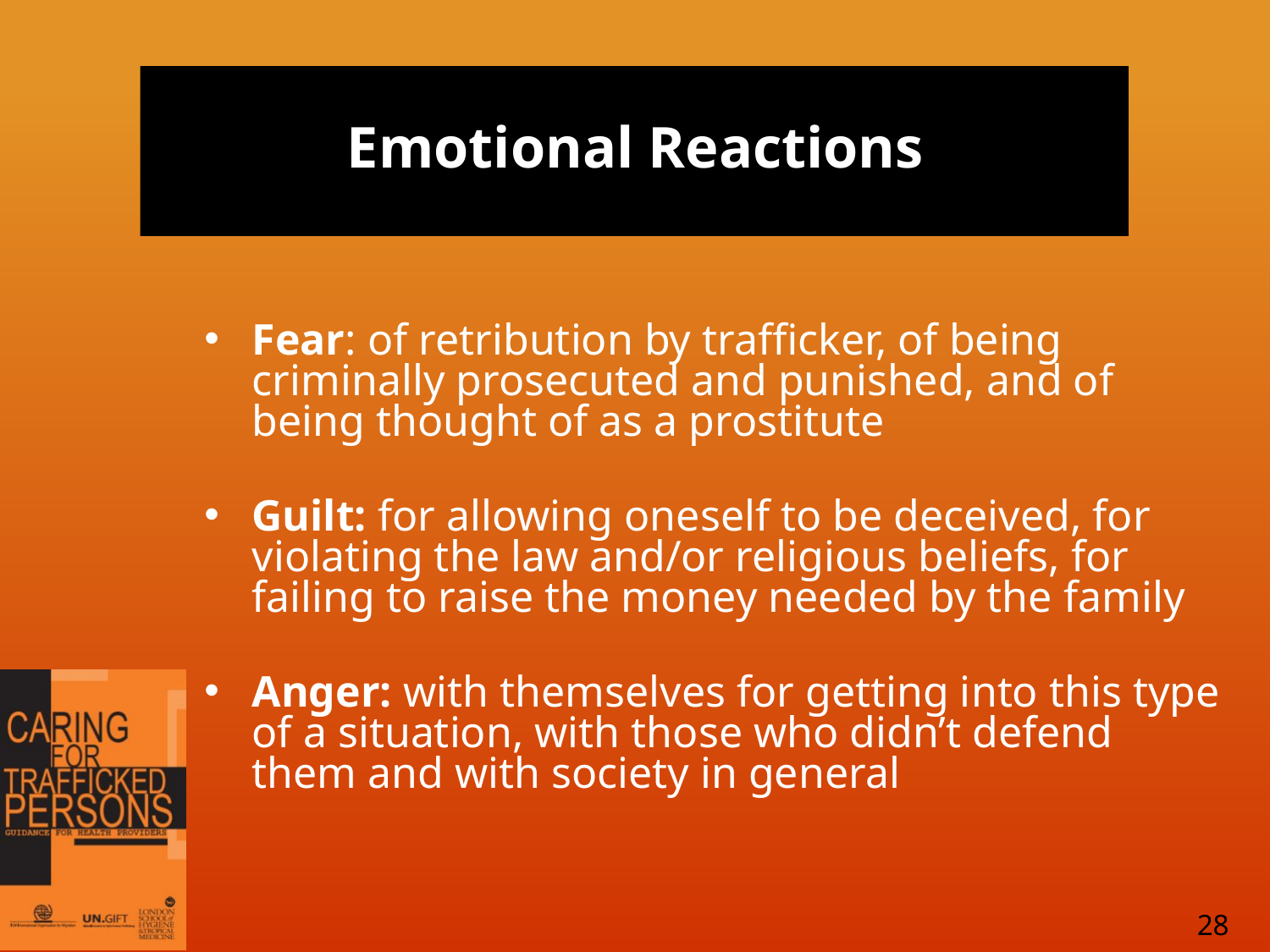

# Emotional Reactions
Fear: of retribution by trafficker, of being criminally prosecuted and punished, and of being thought of as a prostitute
Guilt: for allowing oneself to be deceived, for violating the law and/or religious beliefs, for failing to raise the money needed by the family
Anger: with themselves for getting into this type of a situation, with those who didn’t defend them and with society in general
28

## Slide 29
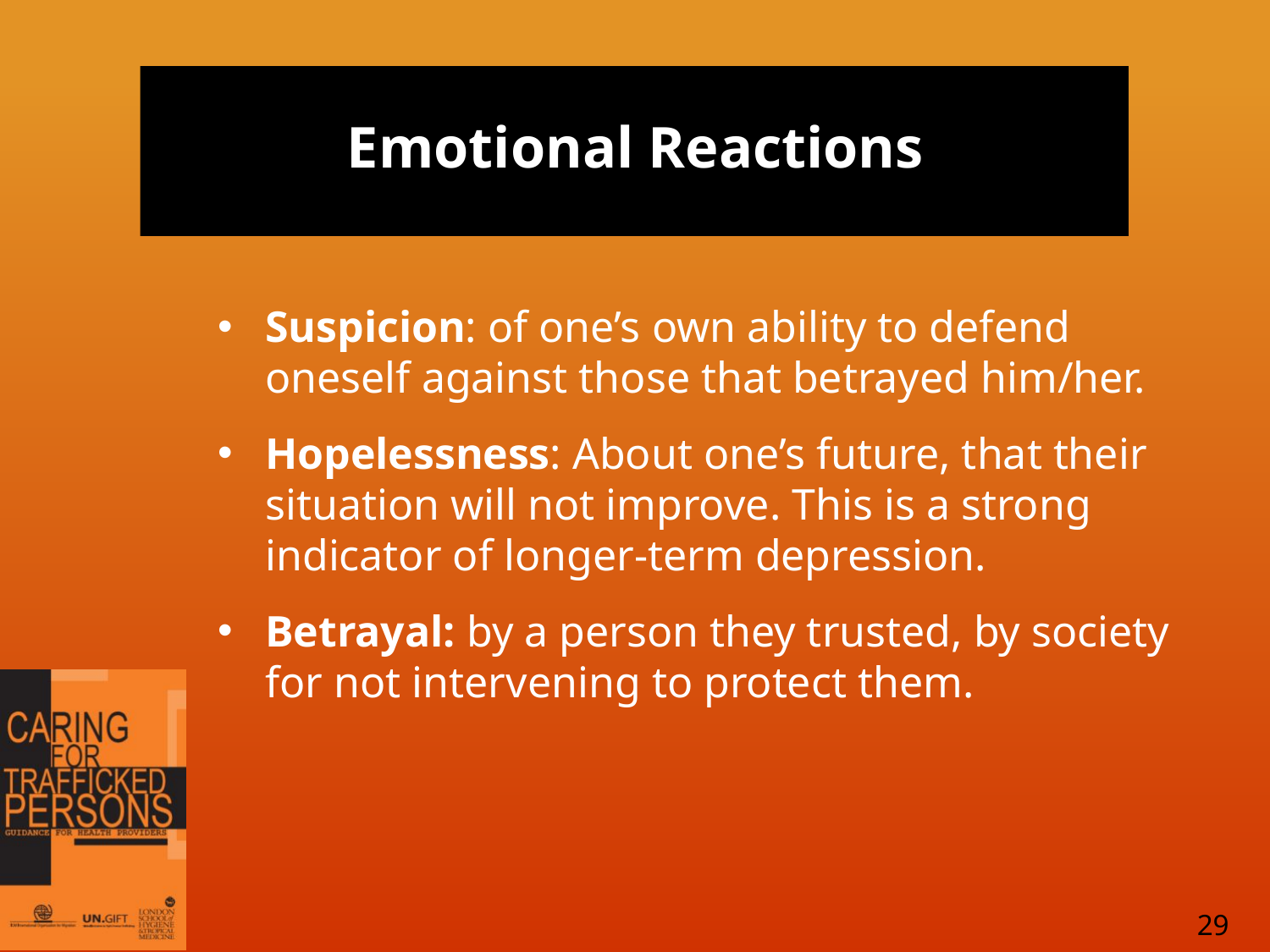

# Emotional Reactions
Suspicion: of one’s own ability to defend oneself against those that betrayed him/her.
Hopelessness: About one’s future, that their situation will not improve. This is a strong indicator of longer-term depression.
Betrayal: by a person they trusted, by society for not intervening to protect them.
29

## Slide 30
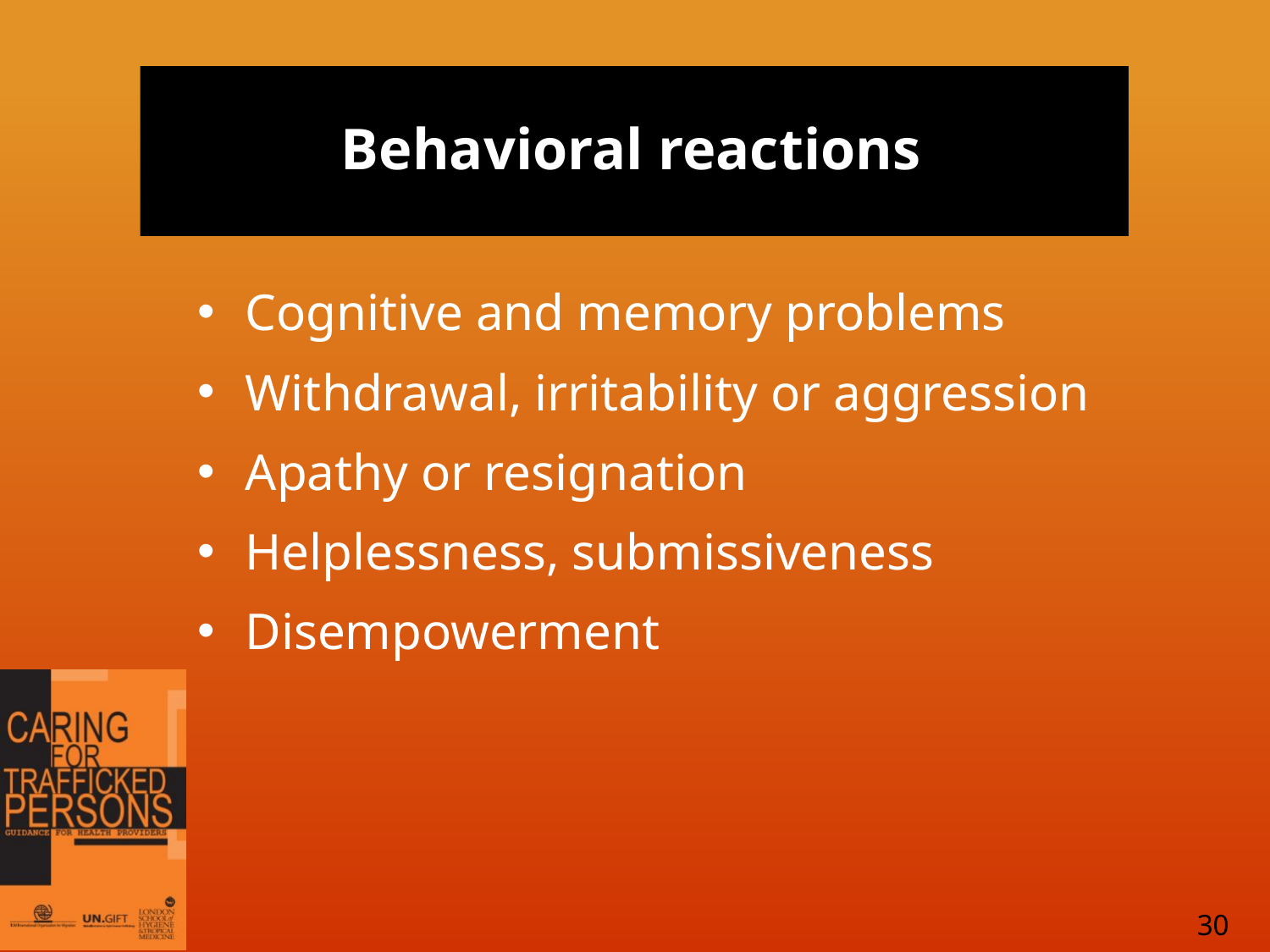

# Behavioral reactions
Cognitive and memory problems
Withdrawal, irritability or aggression
Apathy or resignation
Helplessness, submissiveness
Disempowerment
30

## Slide 31
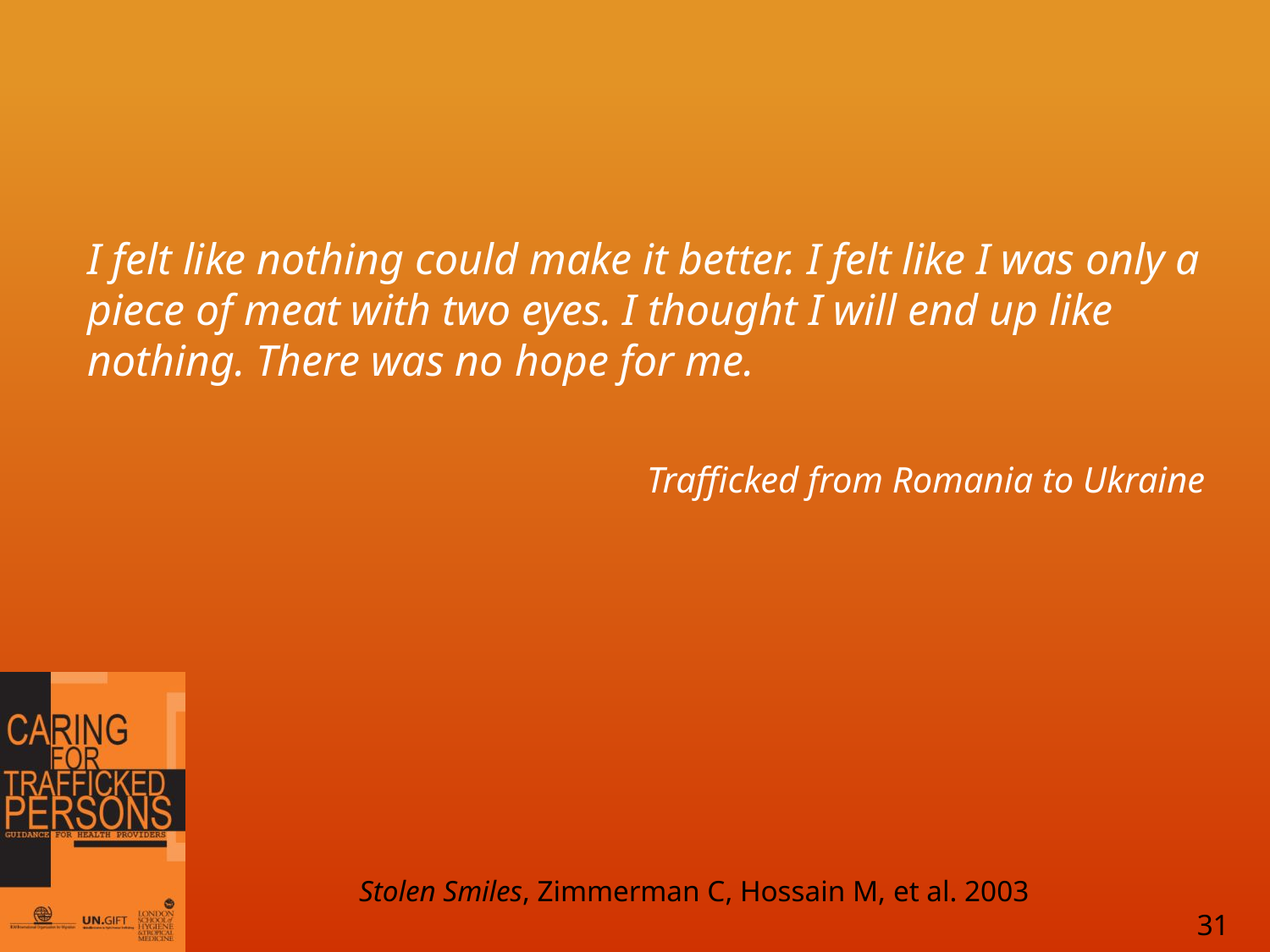

I felt like nothing could make it better. I felt like I was only a piece of meat with two eyes. I thought I will end up like nothing. There was no hope for me.
Trafficked from Romania to Ukraine
Stolen Smiles, Zimmerman C, Hossain M, et al. 2003
31

## Slide 32
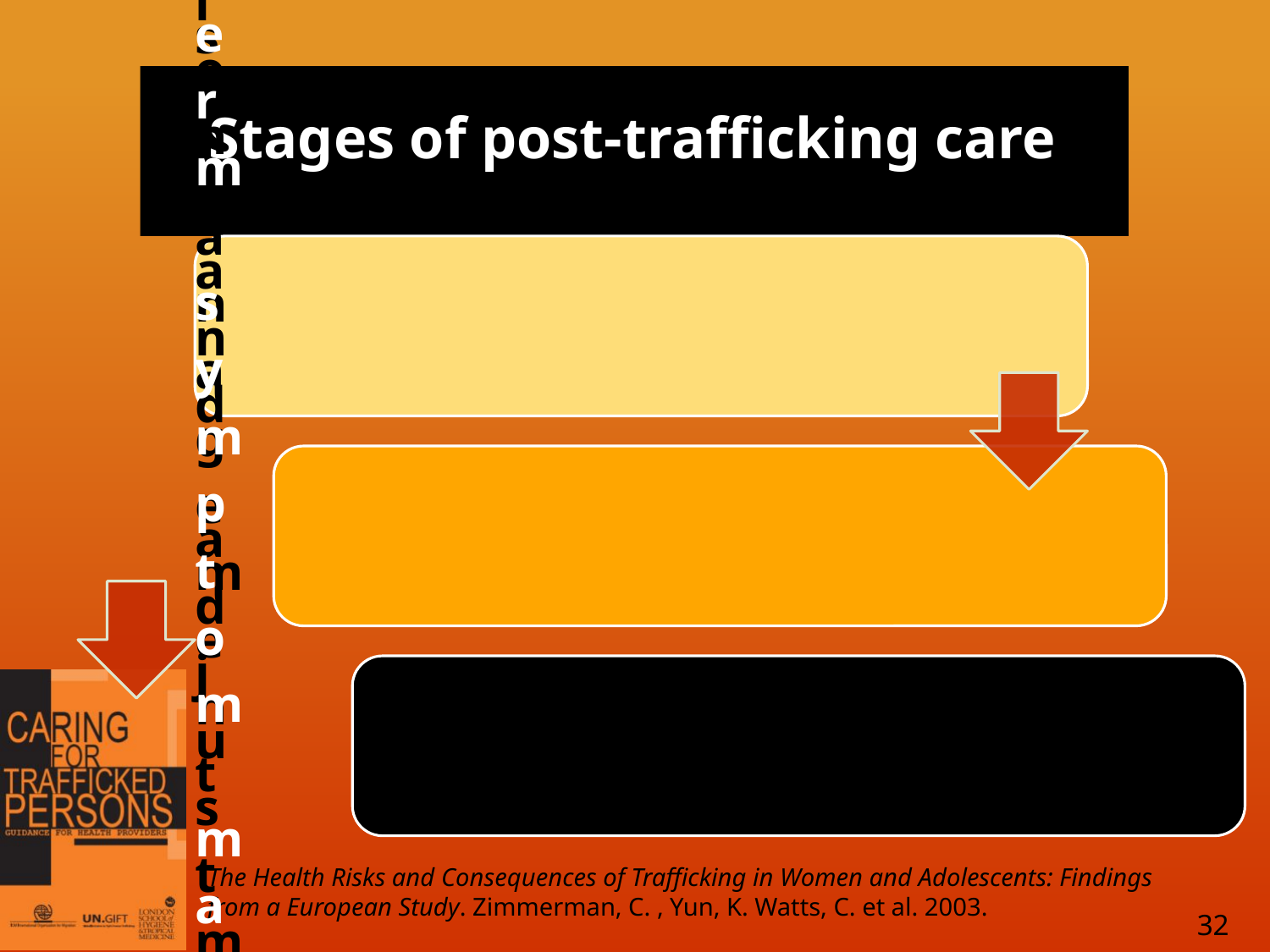

# Stages of post-trafficking care
The Health Risks and Consequences of Trafficking in Women and Adolescents: Findings from a European Study. Zimmerman, C. , Yun, K. Watts, C. et al. 2003.
32

## Slide 33
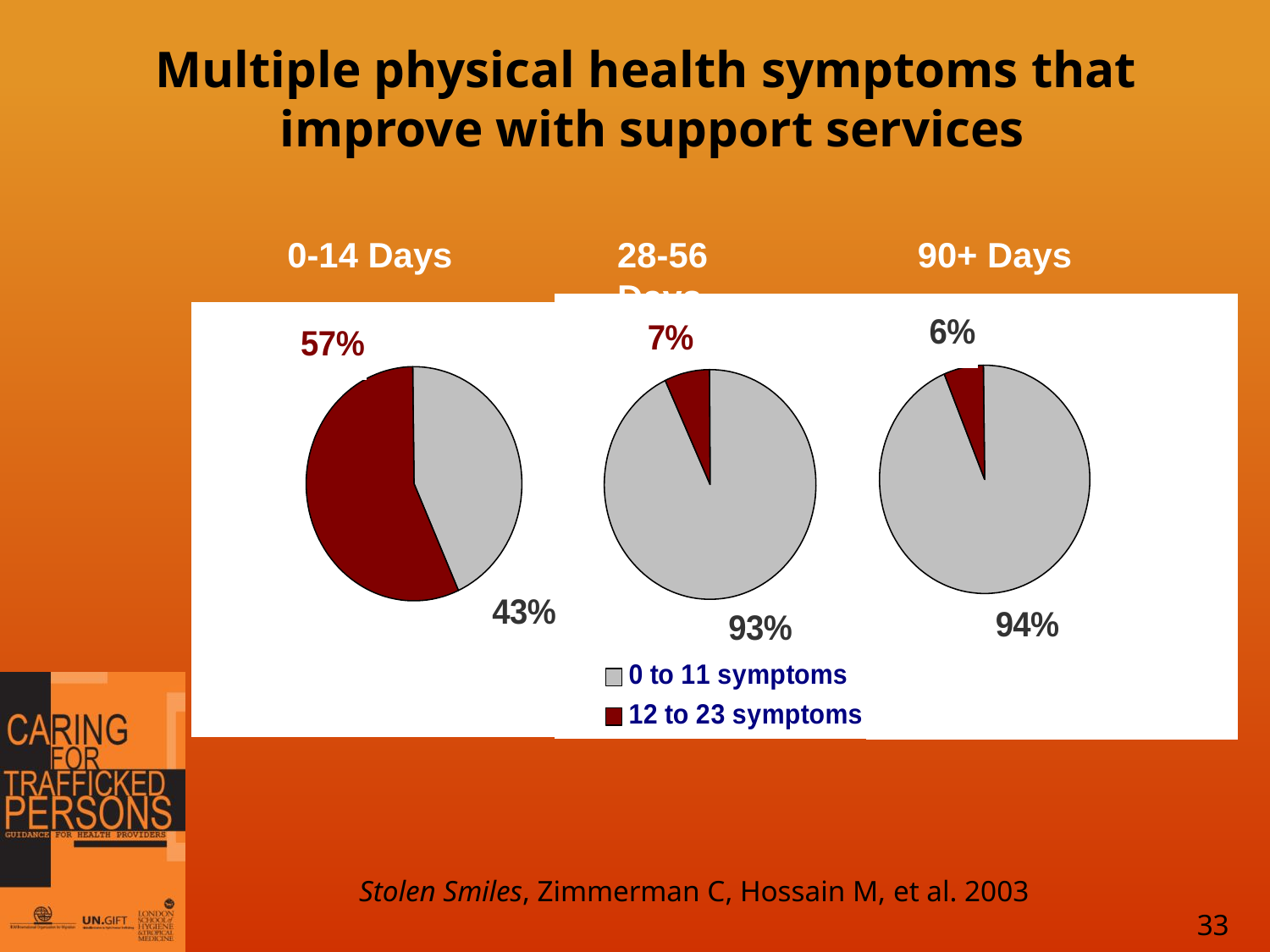

Multiple physical health symptoms that improve with support services
0-14 Days
28-56 Days
90+ Days
Stolen Smiles, Zimmerman C, Hossain M, et al. 2003
33

## Slide 34
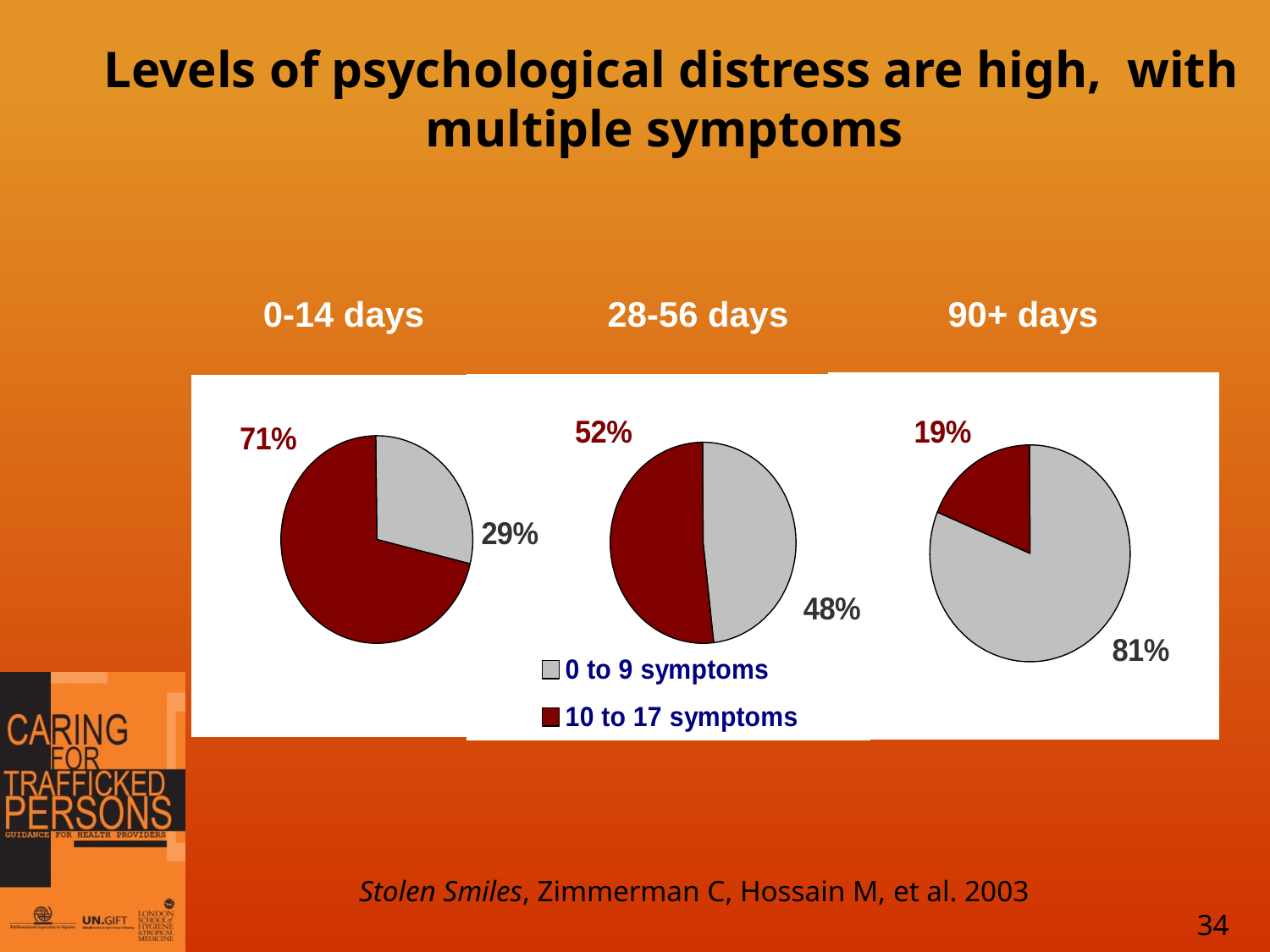

Levels of psychological distress are high, with multiple symptoms
28-56 days
90+ days
0-14 days
Stolen Smiles, Zimmerman C, Hossain M, et al. 2003
34

## Slide 35
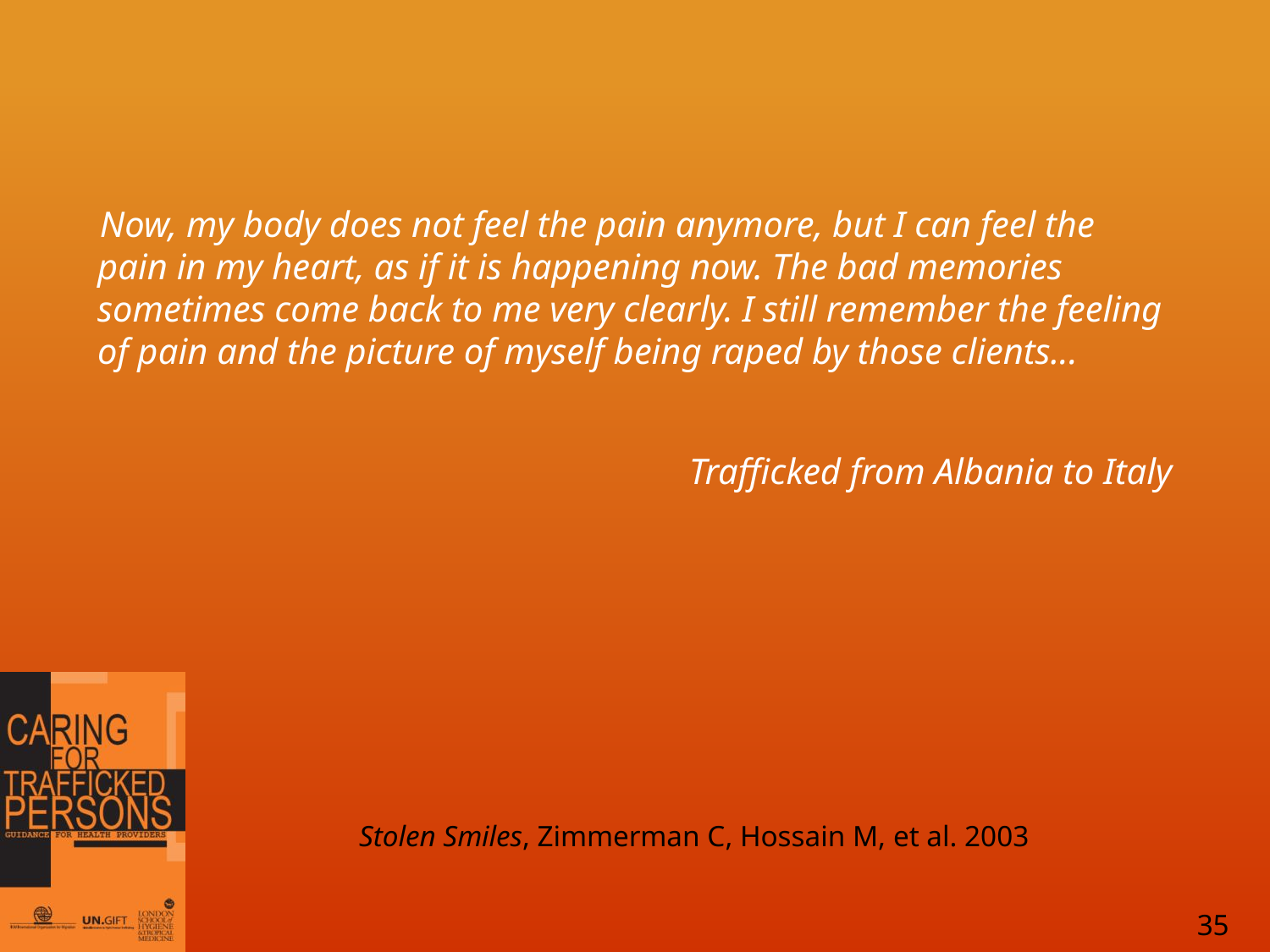

Now, my body does not feel the pain anymore, but I can feel the pain in my heart, as if it is happening now. The bad memories sometimes come back to me very clearly. I still remember the feeling of pain and the picture of myself being raped by those clients...
Trafficked from Albania to Italy
Stolen Smiles, Zimmerman C, Hossain M, et al. 2003
35

## Slide 36
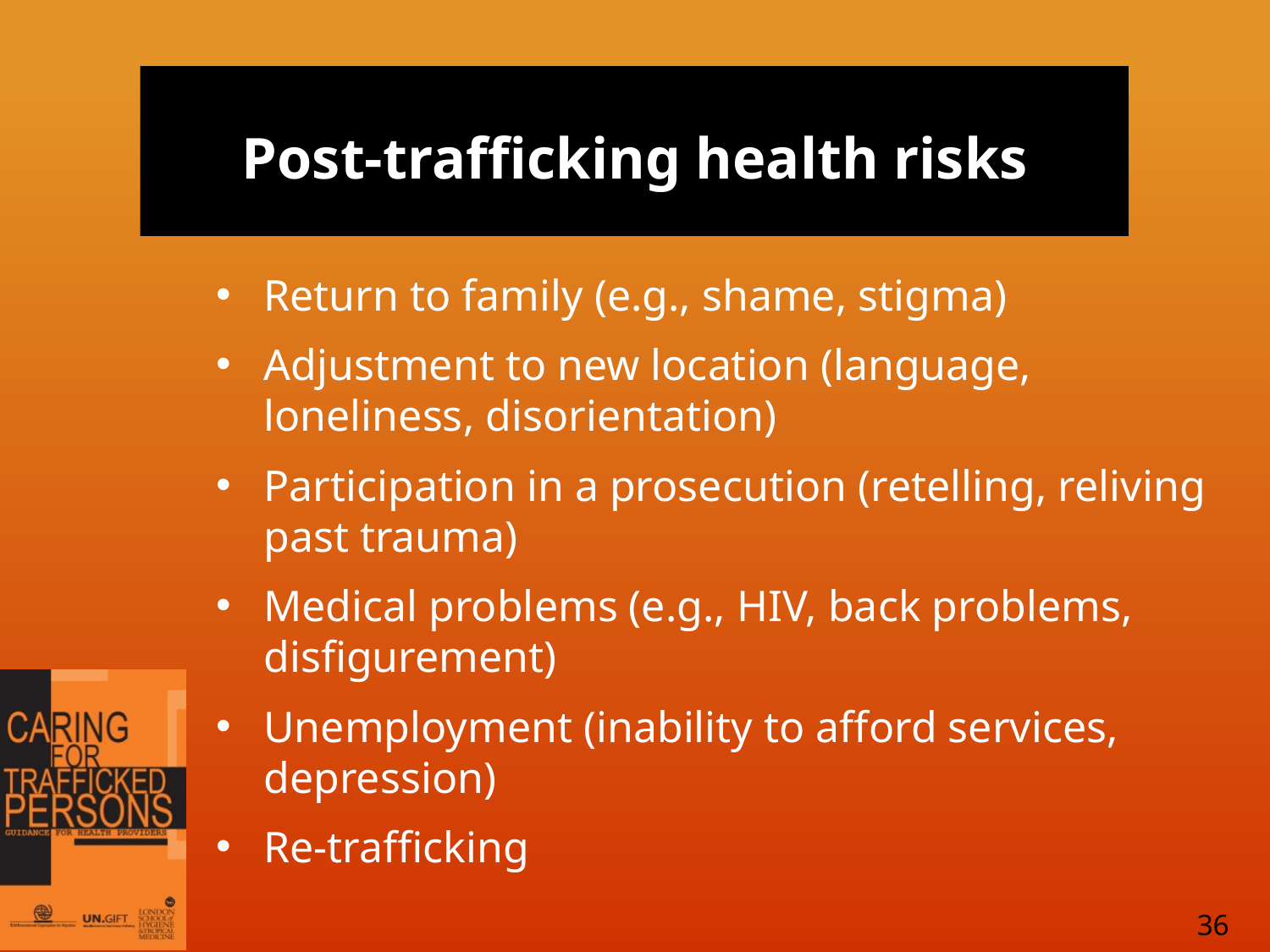

# Post-trafficking health risks
Return to family (e.g., shame, stigma)
Adjustment to new location (language, loneliness, disorientation)
Participation in a prosecution (retelling, reliving past trauma)
Medical problems (e.g., HIV, back problems, disfigurement)
Unemployment (inability to afford services, depression)
Re-trafficking
36

## Slide 37
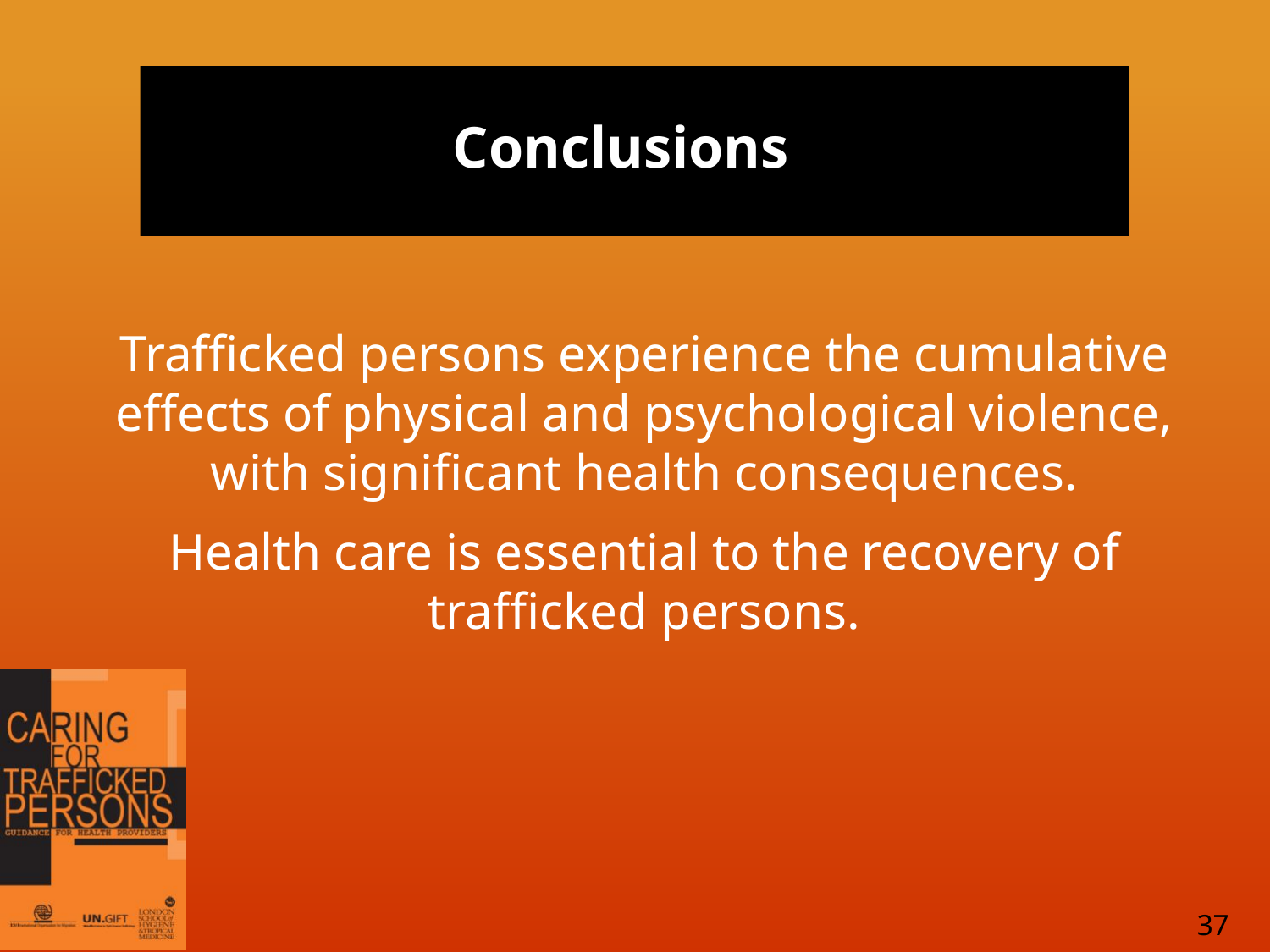

# Conclusions
Trafficked persons experience the cumulative effects of physical and psychological violence, with significant health consequences.
Health care is essential to the recovery of trafficked persons.
37

## Slide 38
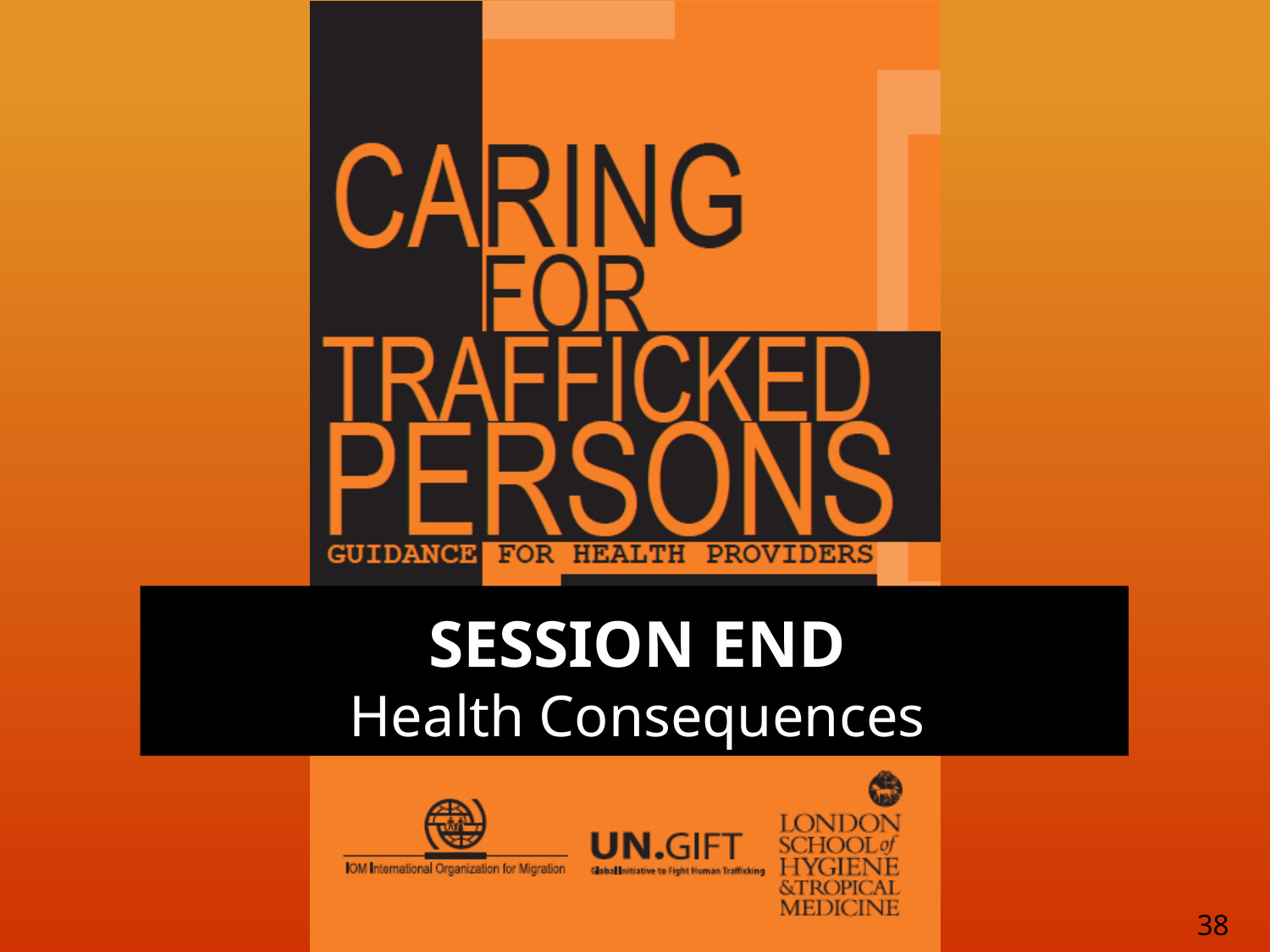

SESSION END
Health Consequences
38
