# Supplementary material for: Health Care Providers and Human Trafficking: What do They Know, What do They Need to Know? Findings from the Middle East, the Caribbean, and Central America
Source: Front Public Health. 2015 Jan 29;3:6. doi: 10.3389/fpubh.2015.00006 (PMC4310216; doi:10.3389/fpubh.2015.00006)
Supplement: Supplementary file 1 [file Presentation_1.ZIP › Caring for Trafficked Persons Training Session 3.pptx]

## Slide 1
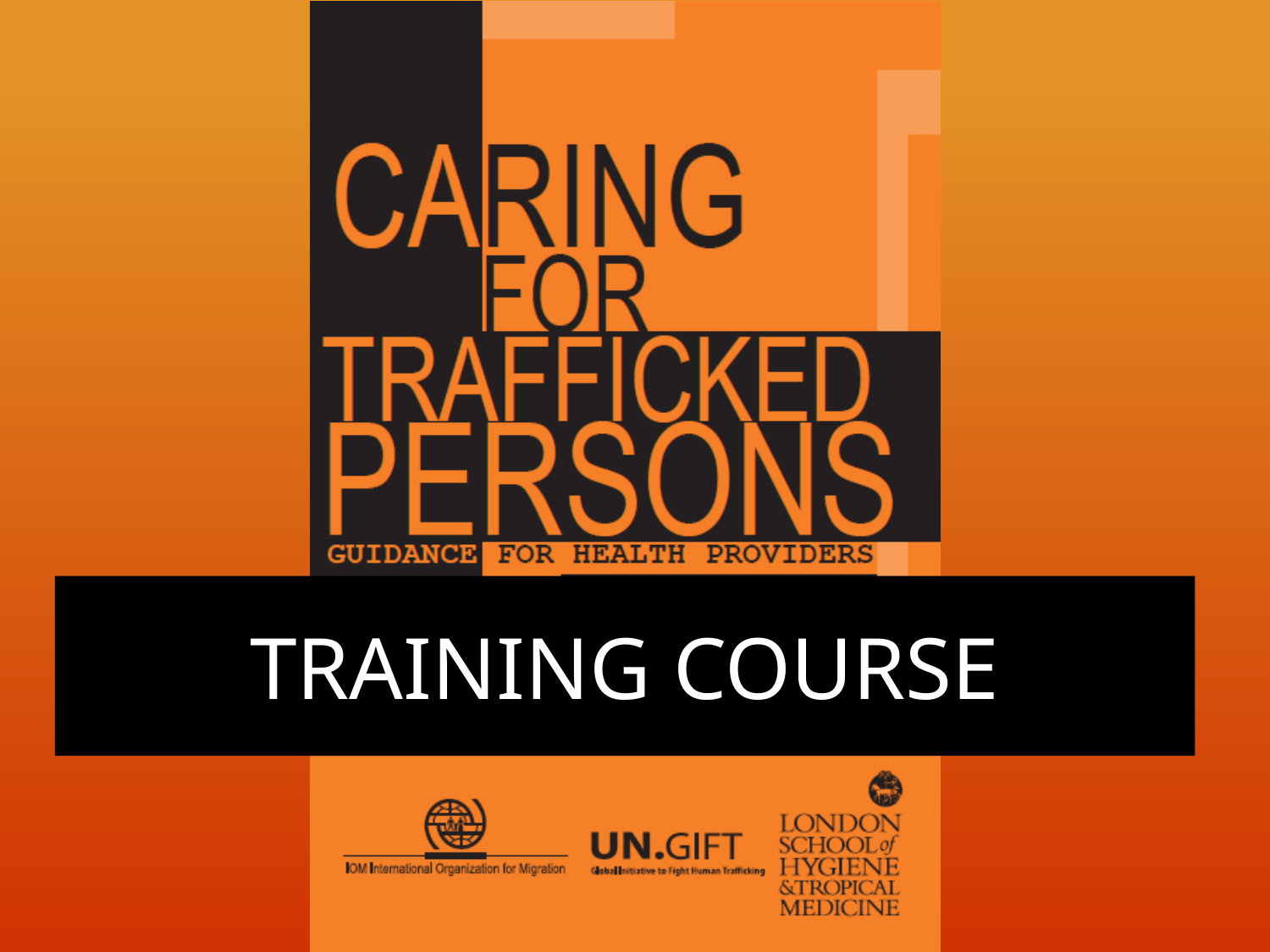

## Slide 2
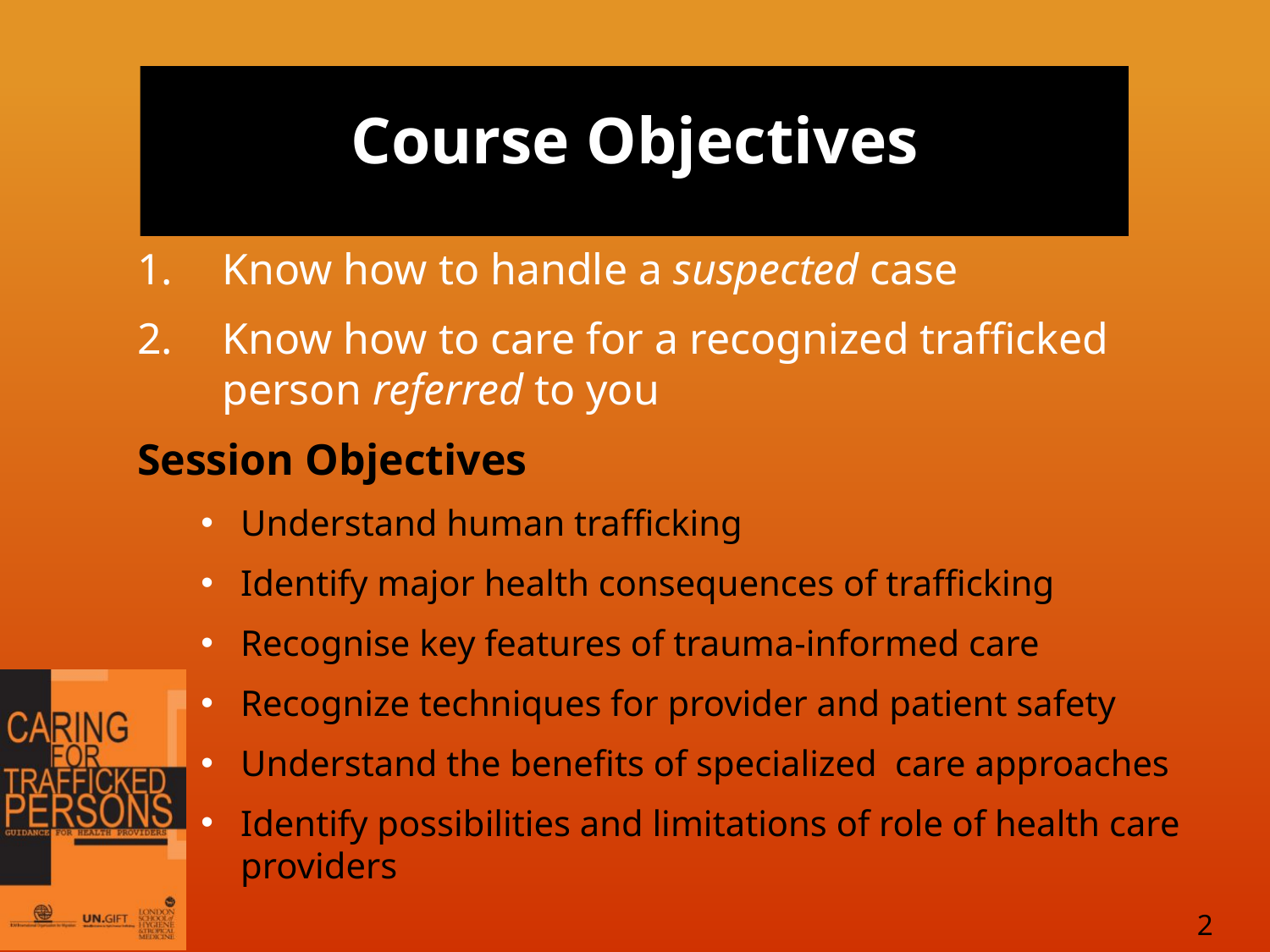

# Course Objectives
Know how to handle a suspected case
Know how to care for a recognized trafficked person referred to you
Session Objectives
Understand human trafficking
Identify major health consequences of trafficking
Recognise key features of trauma-informed care
Recognize techniques for provider and patient safety
Understand the benefits of specialized care approaches
Identify possibilities and limitations of role of health care providers
2

## Slide 3
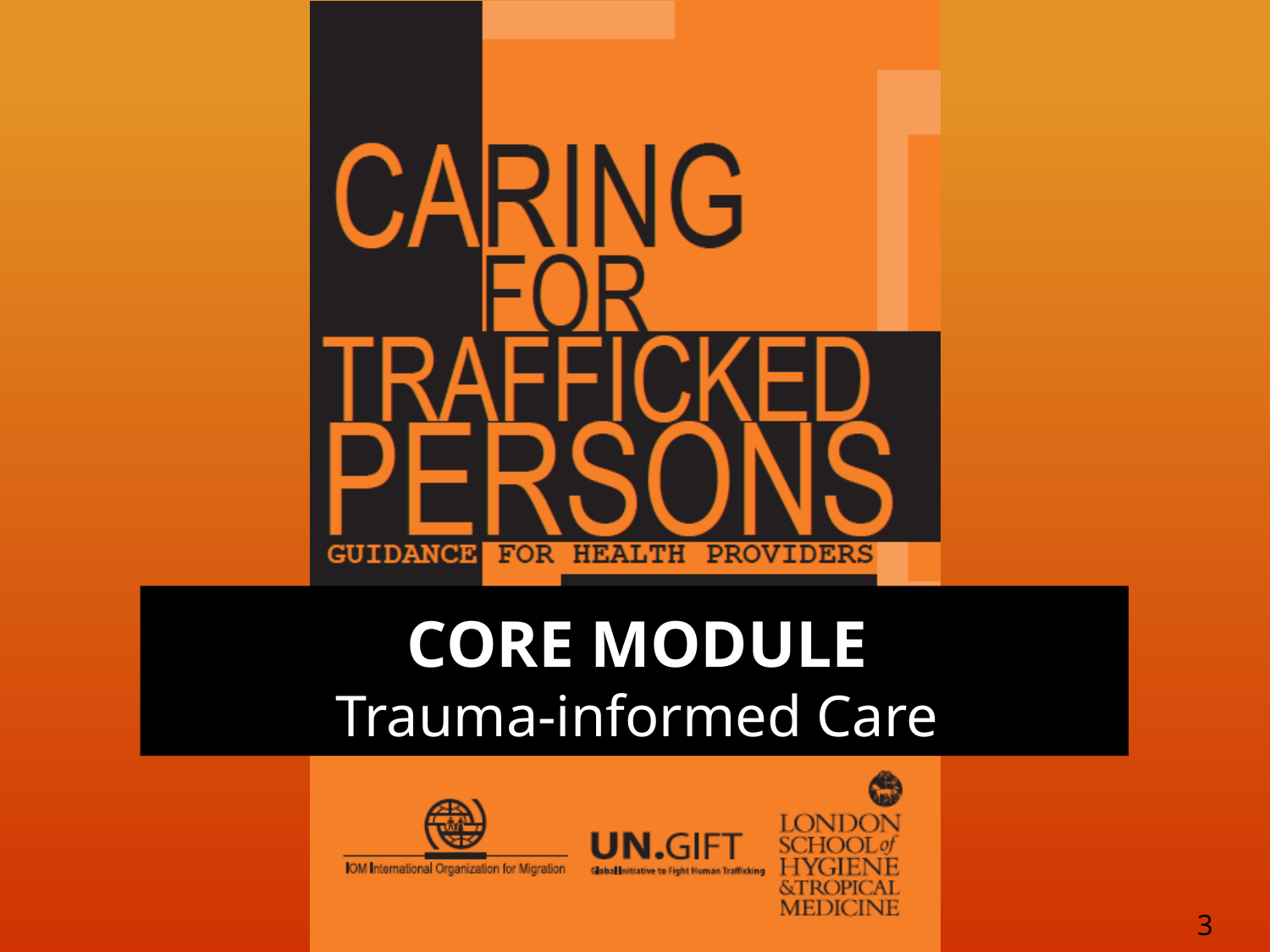

CORE MODULE
Trauma-informed Care
3

## Slide 4
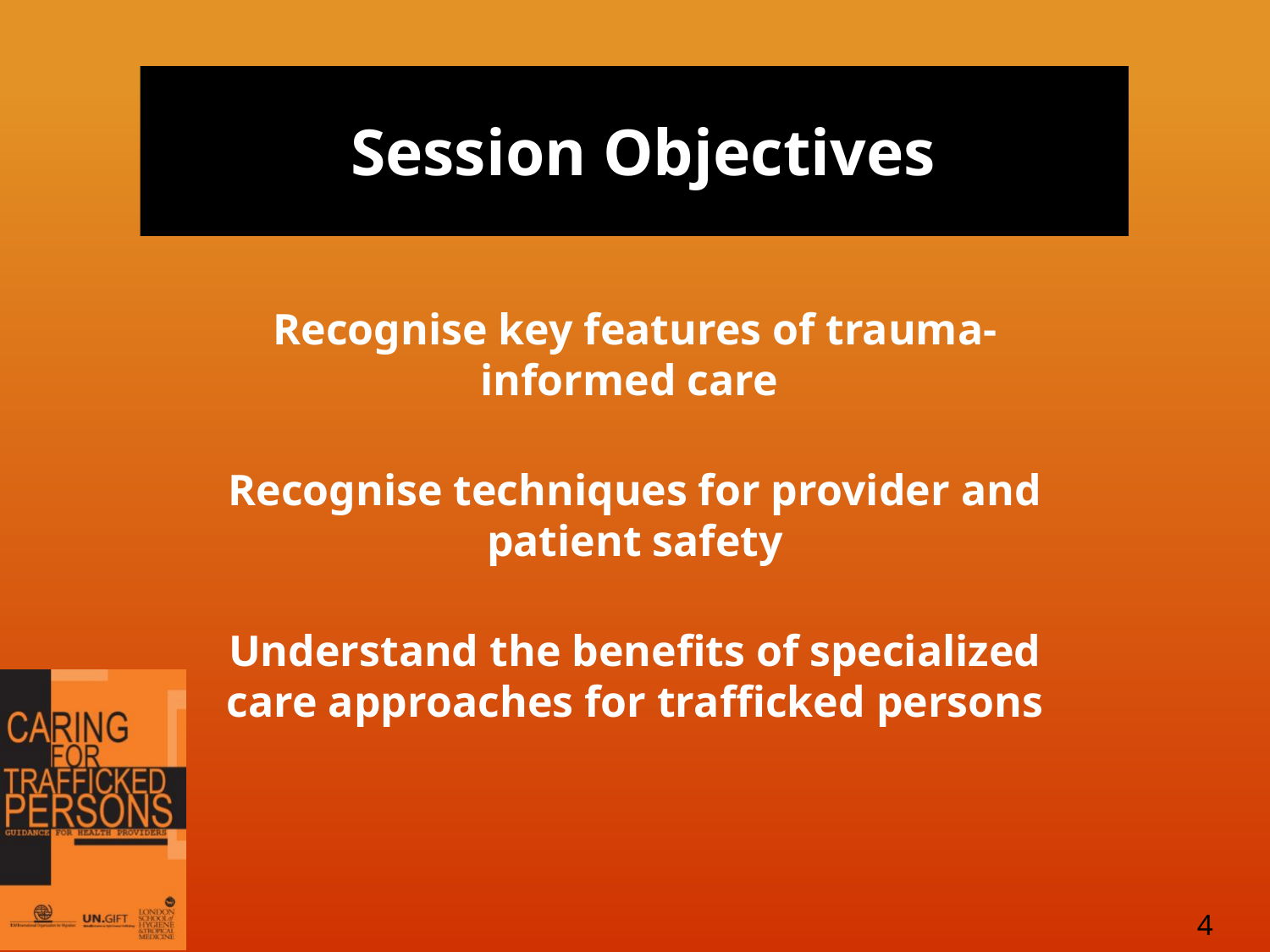

# Session Objectives
Recognise key features of trauma-informed care
Recognise techniques for provider and patient safety
Understand the benefits of specialized care approaches for trafficked persons
4

## Slide 5
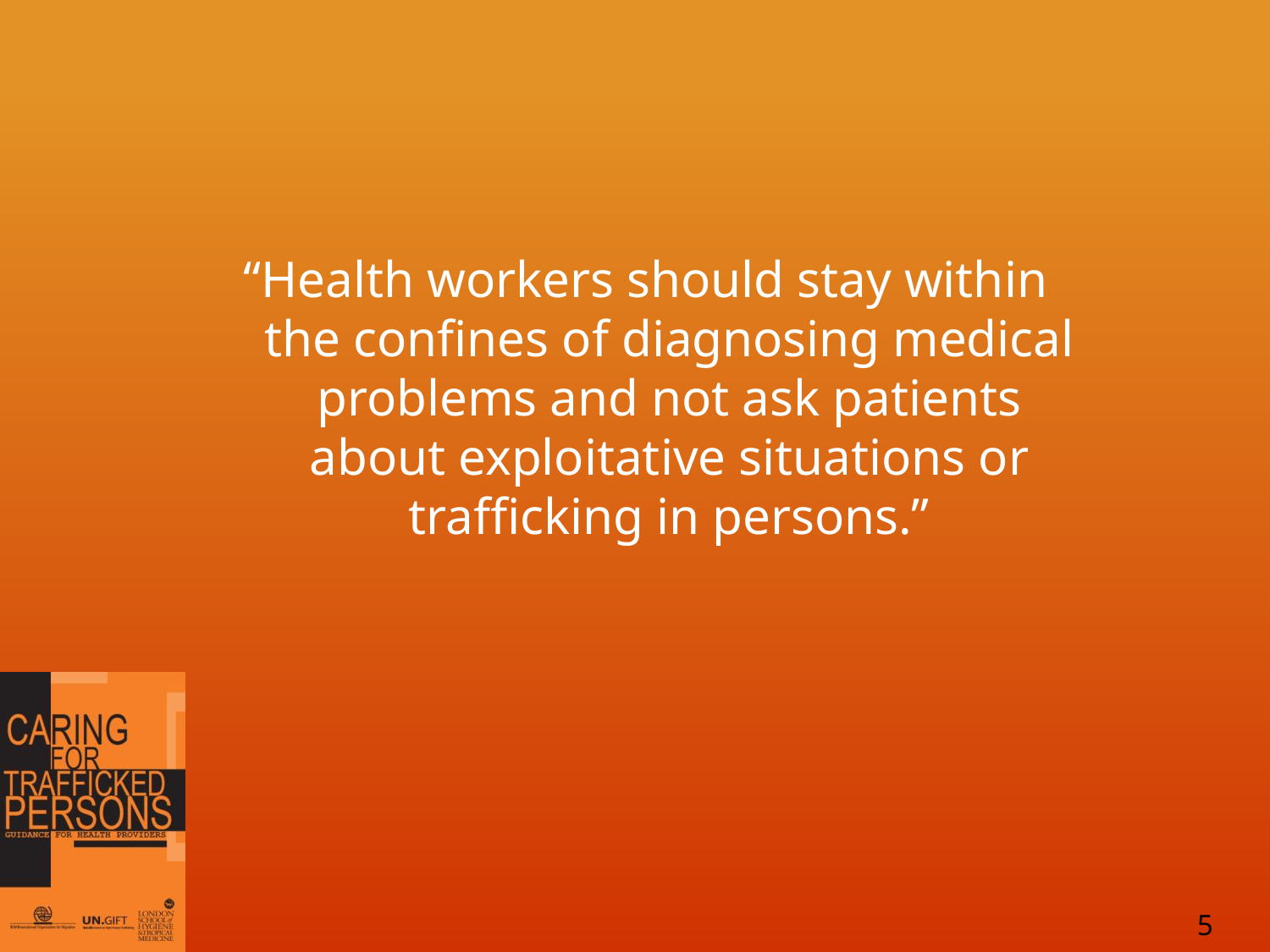

“Health workers should stay within the confines of diagnosing medical problems and not ask patients about exploitative situations or trafficking in persons.”
5

## Slide 6
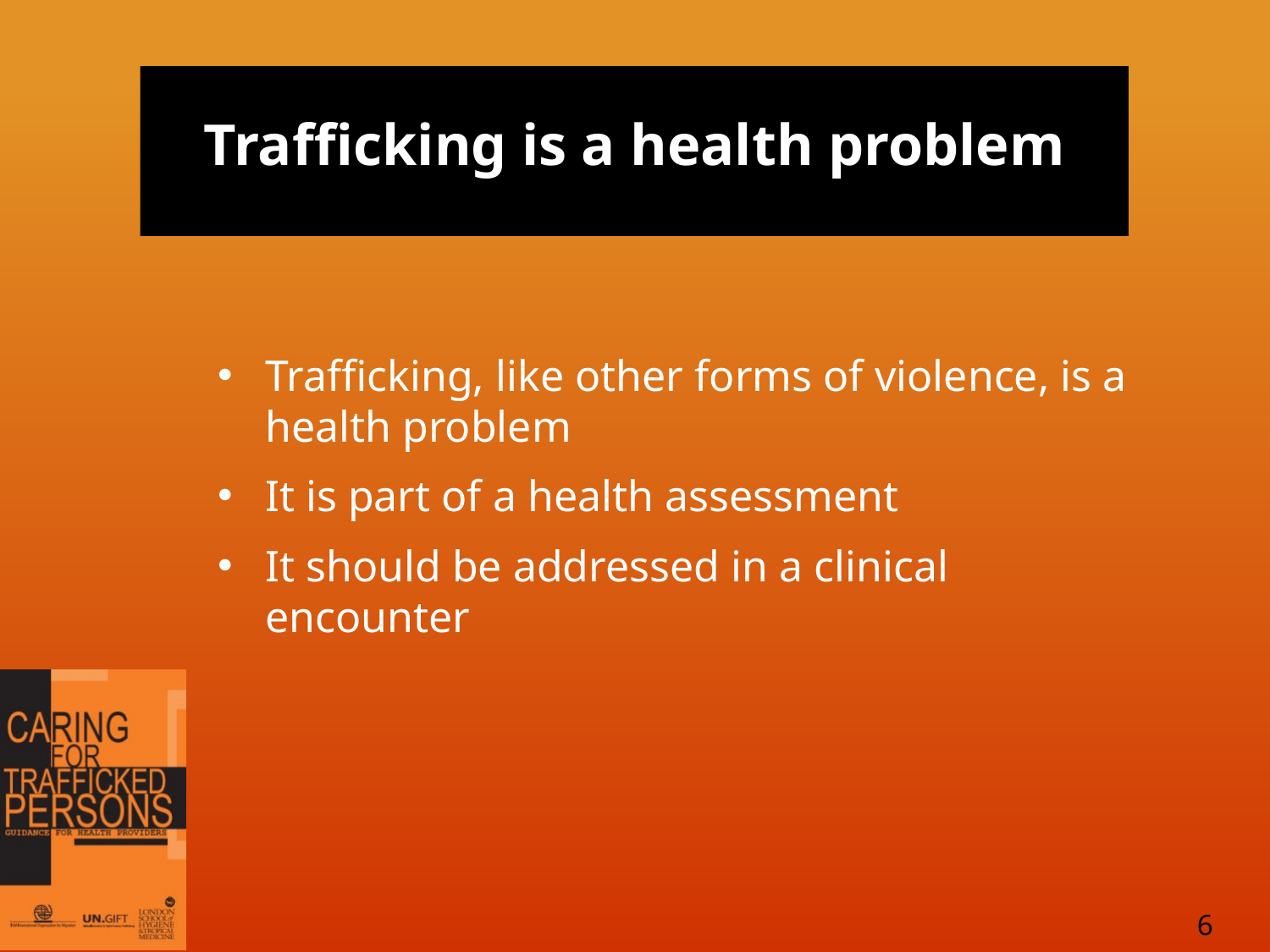

Trafficking is a health problem
Trafficking, like other forms of violence, is a health problem
It is part of a health assessment
It should be addressed in a clinical encounter
6

## Slide 7
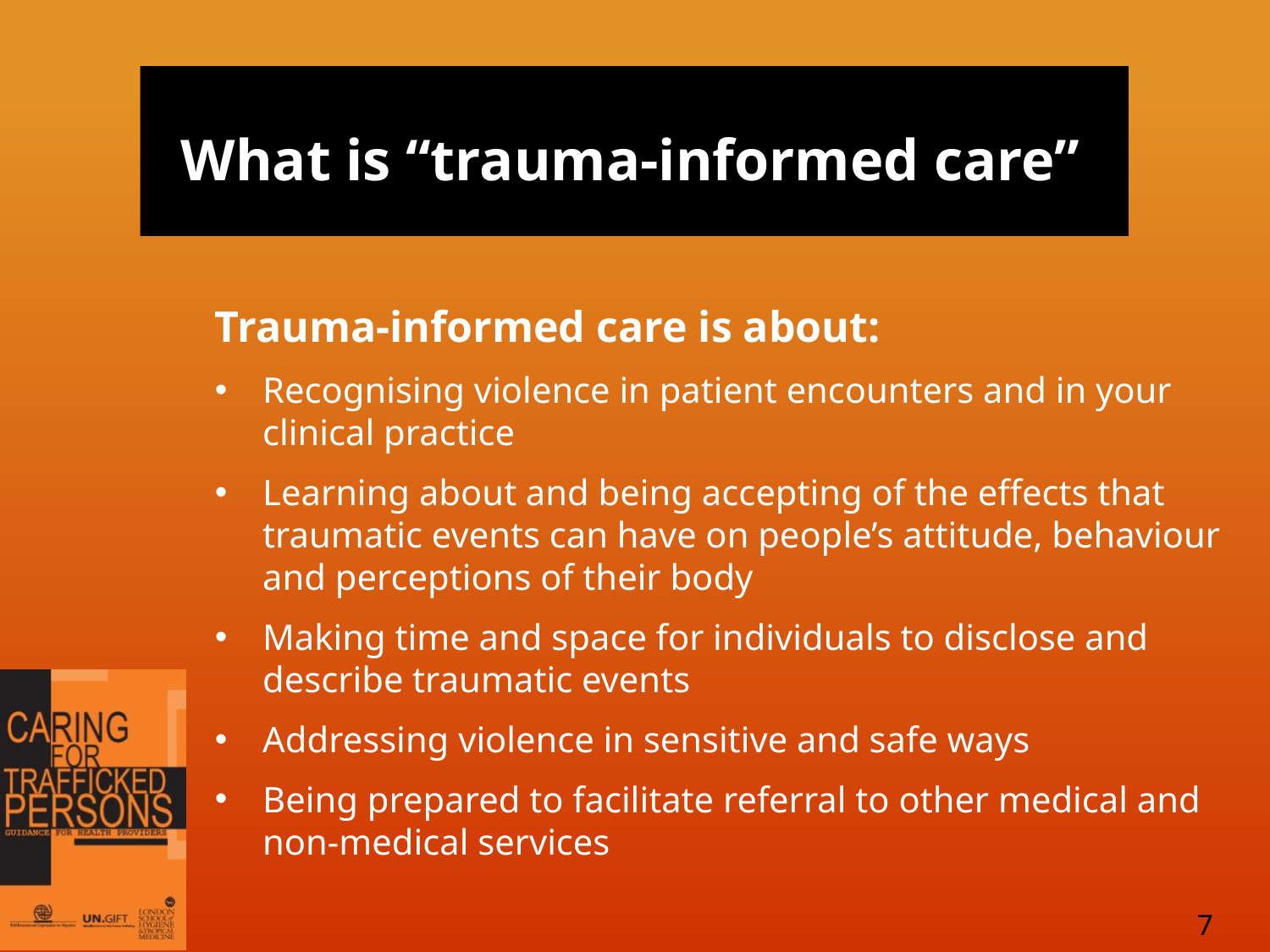

# What is “trauma-informed care”
Trauma-informed care is about:
Recognising violence in patient encounters and in your clinical practice
Learning about and being accepting of the effects that traumatic events can have on people’s attitude, behaviour and perceptions of their body
Making time and space for individuals to disclose and describe traumatic events
Addressing violence in sensitive and safe ways
Being prepared to facilitate referral to other medical and non-medical services
7

## Slide 8
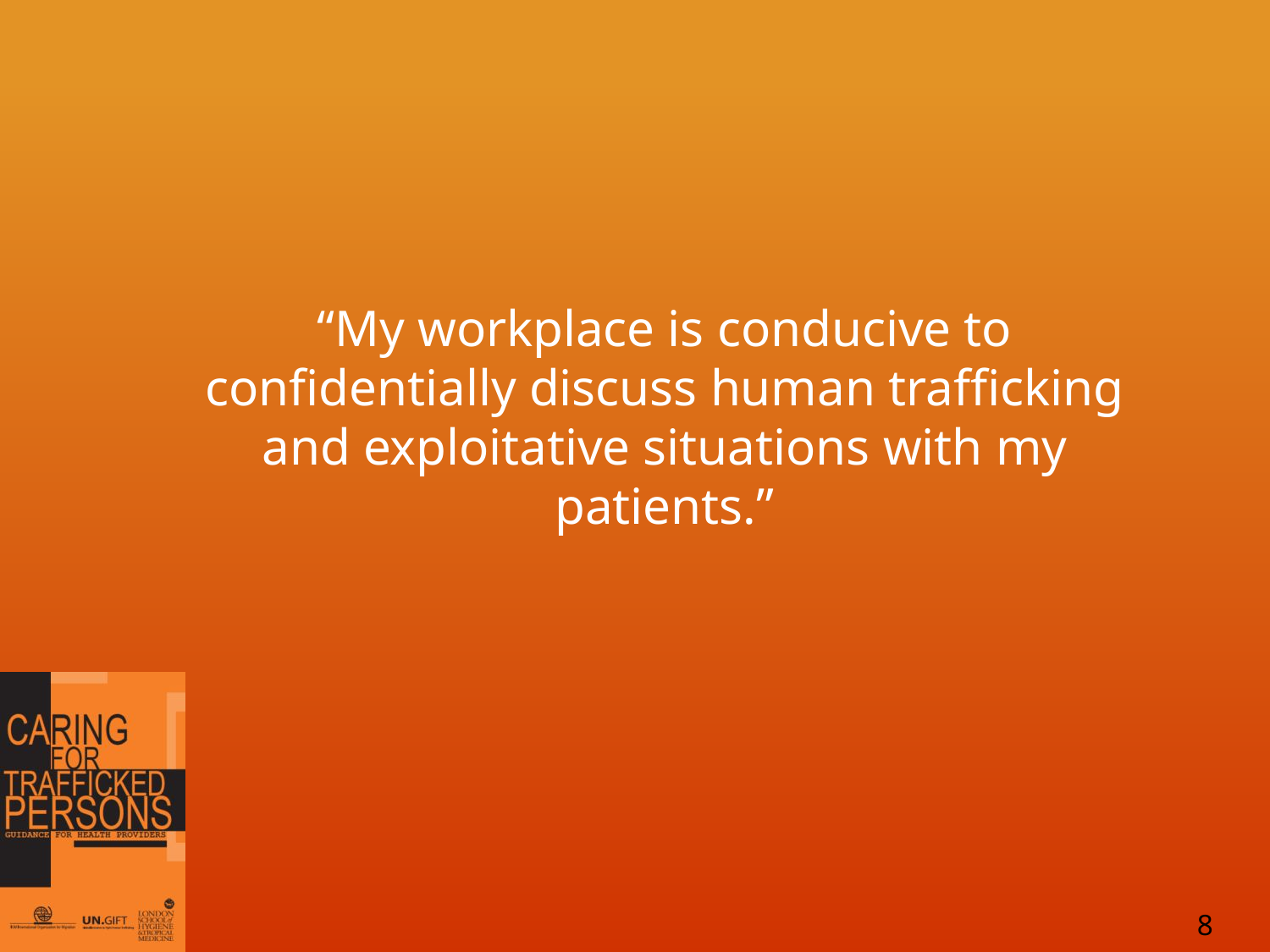

“My workplace is conducive to confidentially discuss human trafficking and exploitative situations with my patients.”
8

## Slide 9
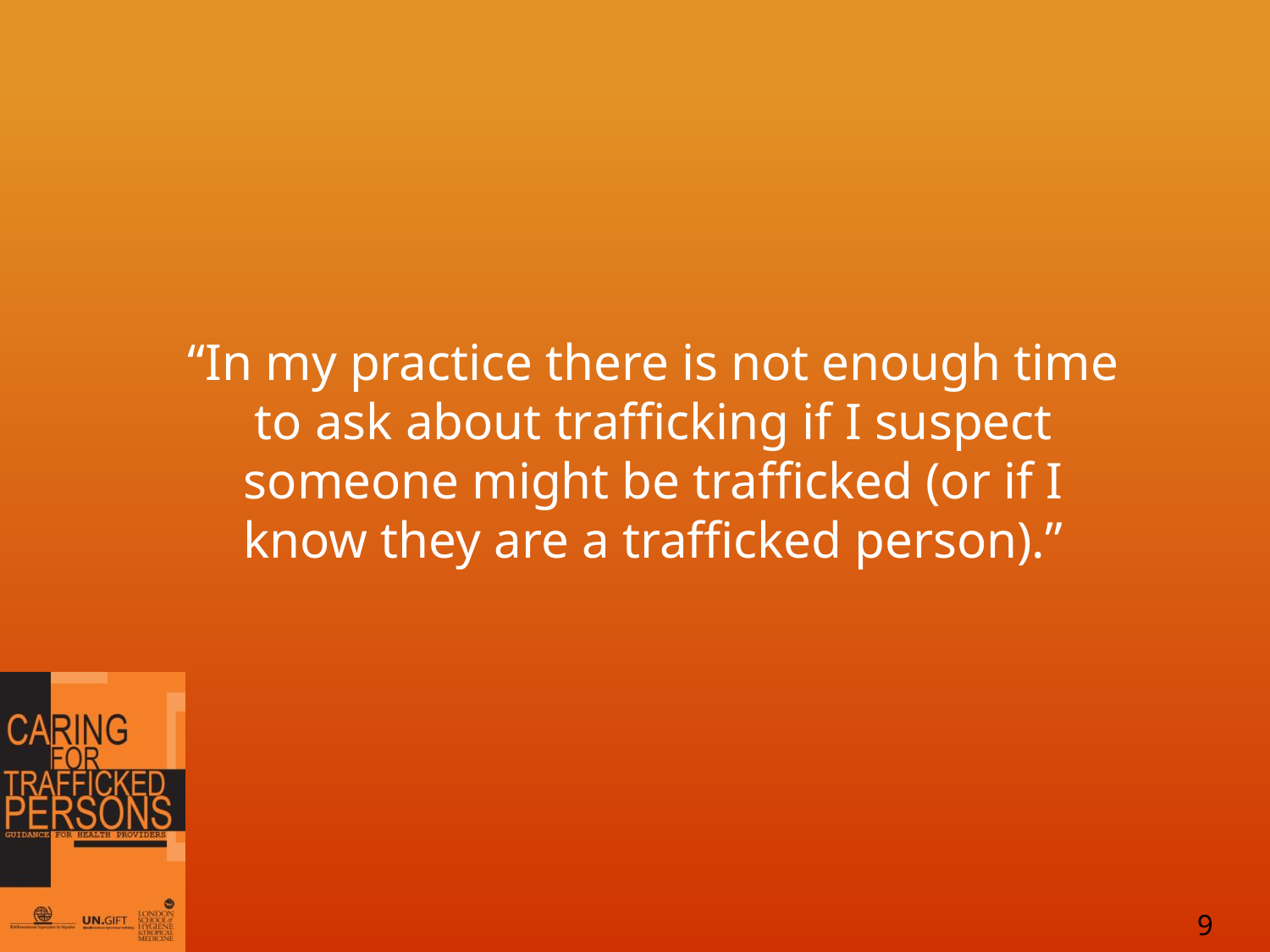

“In my practice there is not enough time to ask about trafficking if I suspect someone might be trafficked (or if I know they are a trafficked person).”
9

## Slide 10
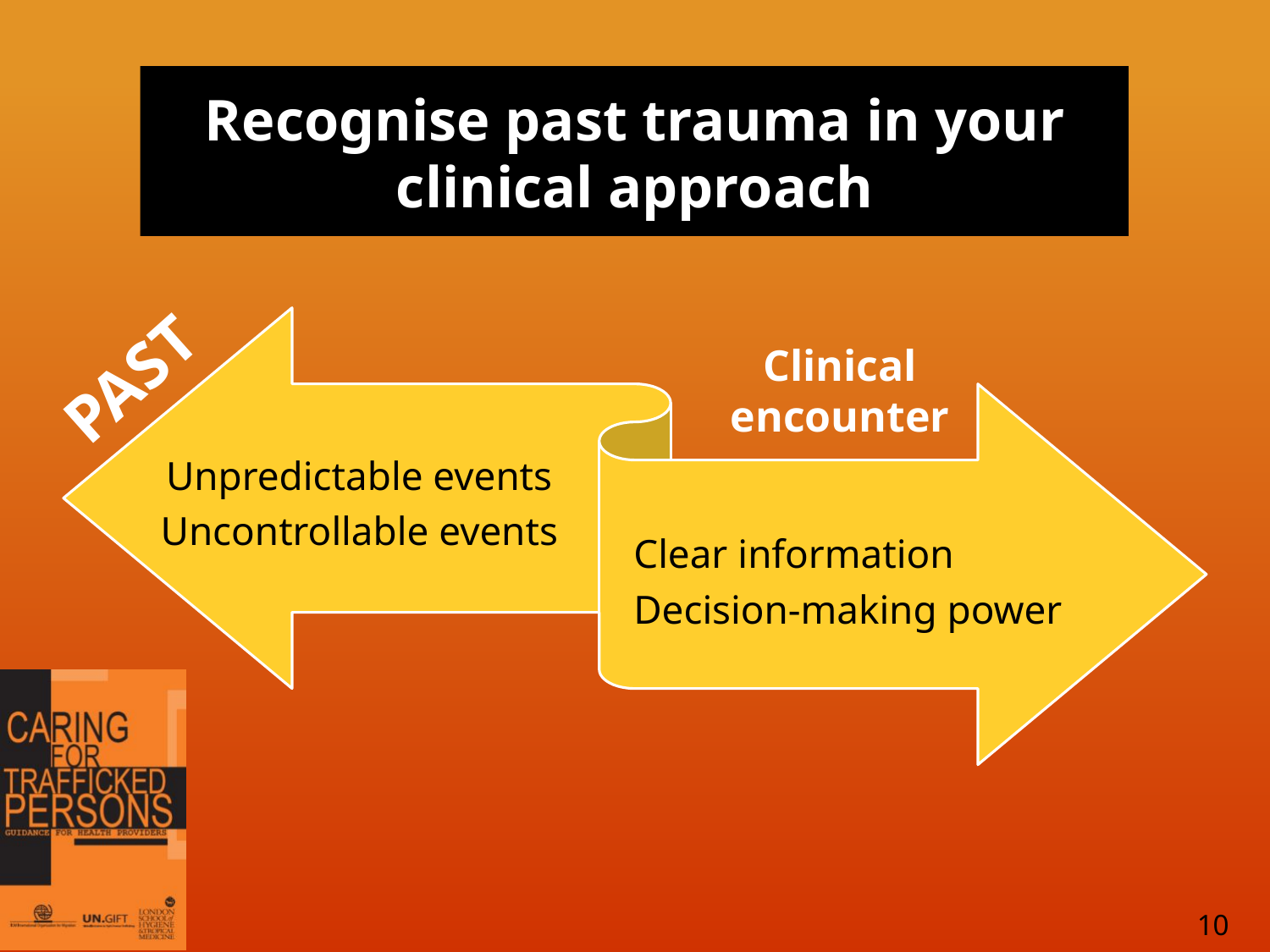

# Recognise past trauma in your clinical approach
Clinical encounter
PAST
10

## Slide 11
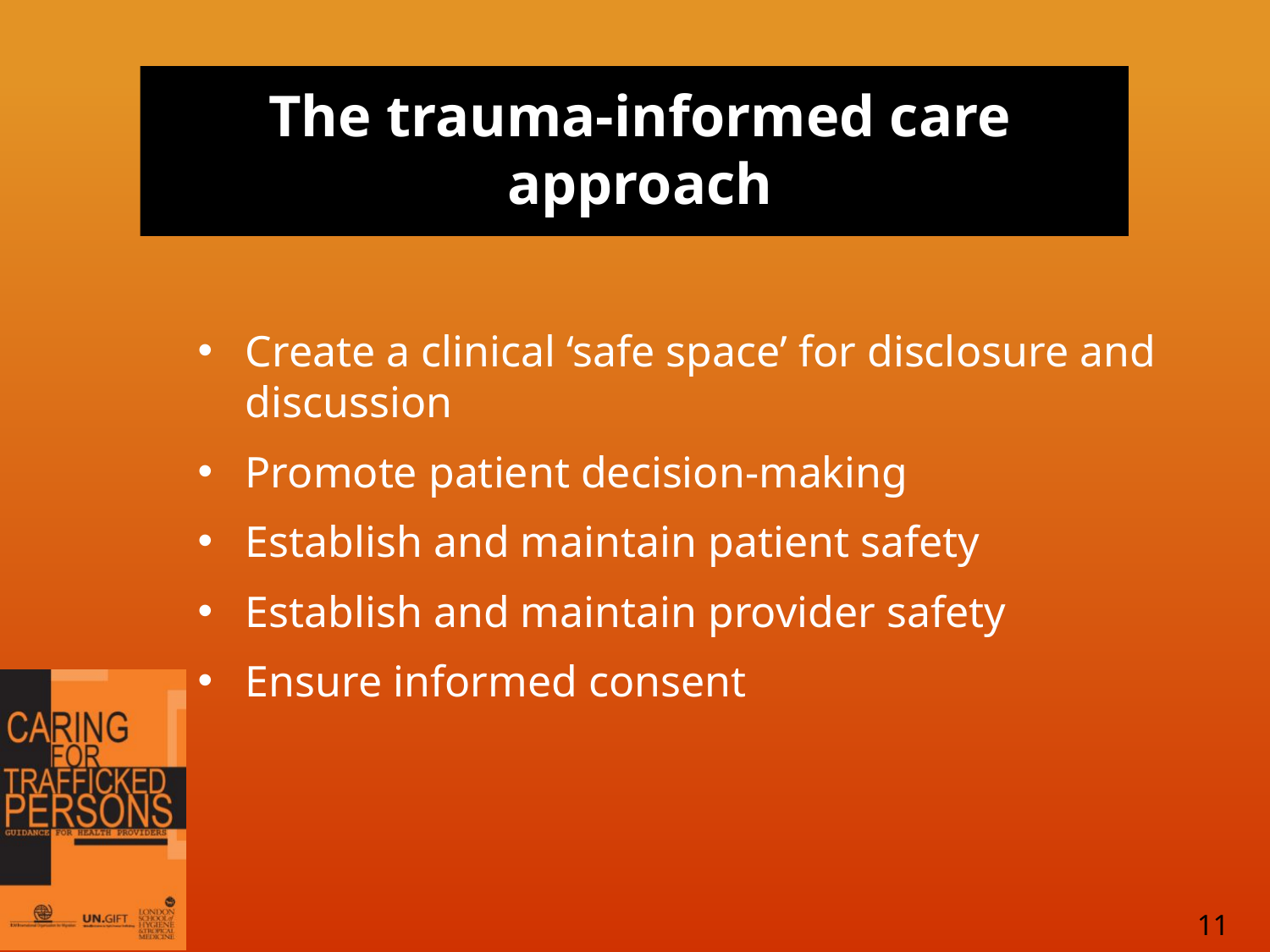

The trauma-informed care approach
Create a clinical ‘safe space’ for disclosure and discussion
Promote patient decision-making
Establish and maintain patient safety
Establish and maintain provider safety
Ensure informed consent
11

## Slide 12
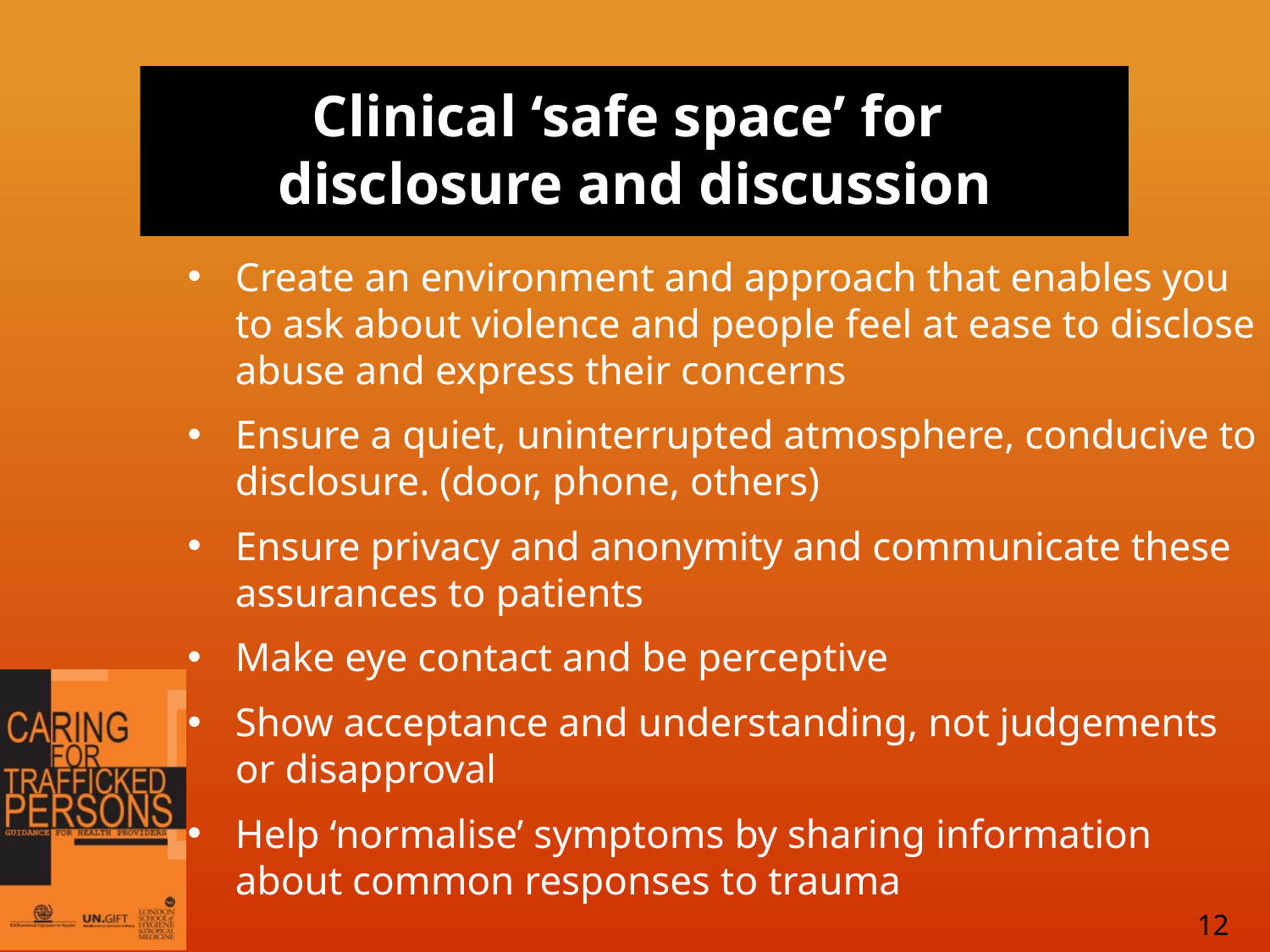

# Clinical ‘safe space’ for disclosure and discussion
Create an environment and approach that enables you to ask about violence and people feel at ease to disclose abuse and express their concerns
Ensure a quiet, uninterrupted atmosphere, conducive to disclosure. (door, phone, others)
Ensure privacy and anonymity and communicate these assurances to patients
Make eye contact and be perceptive
Show acceptance and understanding, not judgements or disapproval
Help ‘normalise’ symptoms by sharing information about common responses to trauma
12

## Slide 13
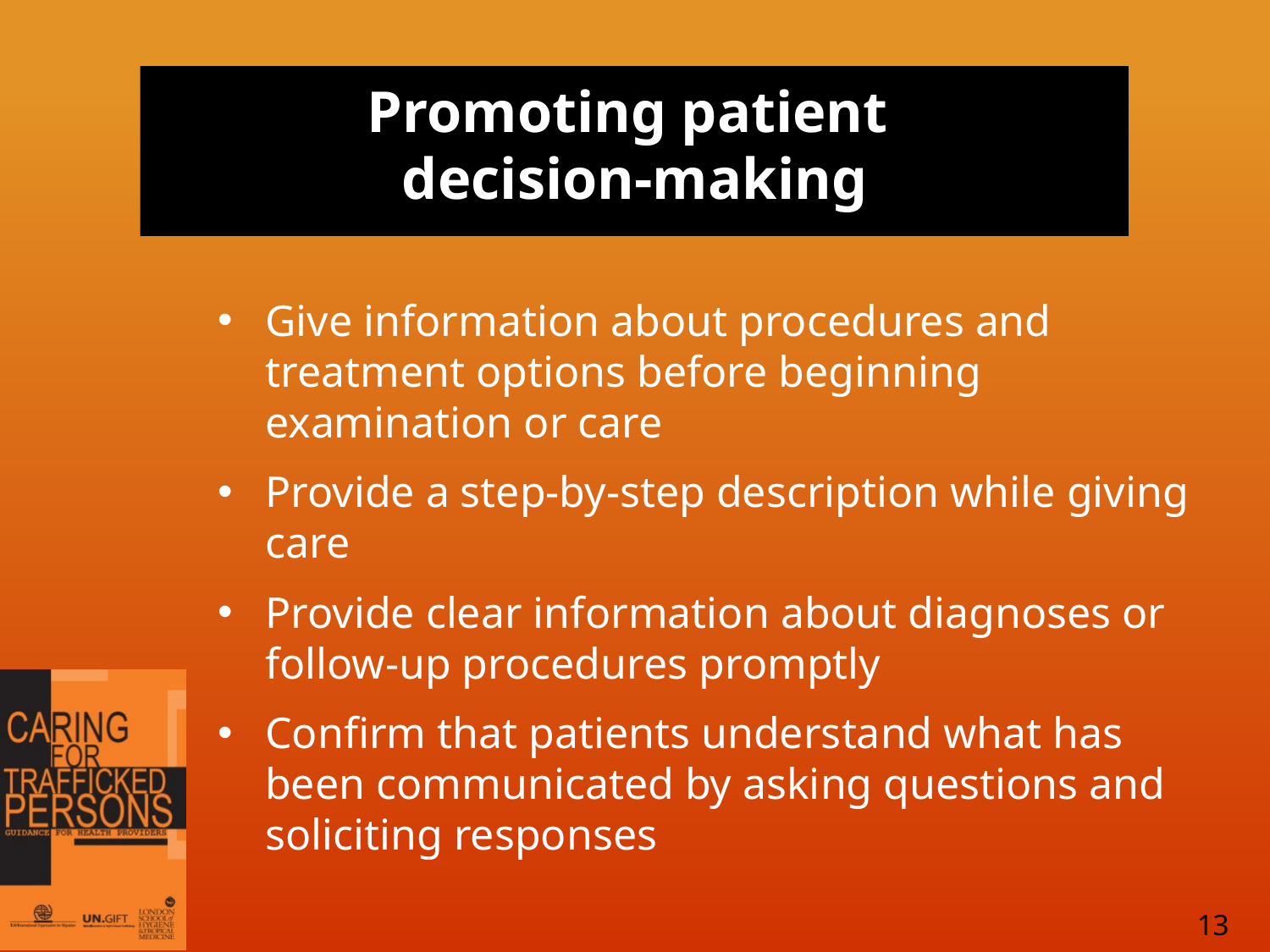

# Promoting patient decision-making
Give information about procedures and treatment options before beginning examination or care
Provide a step-by-step description while giving care
Provide clear information about diagnoses or follow-up procedures promptly
Confirm that patients understand what has been communicated by asking questions and soliciting responses
13

## Slide 14
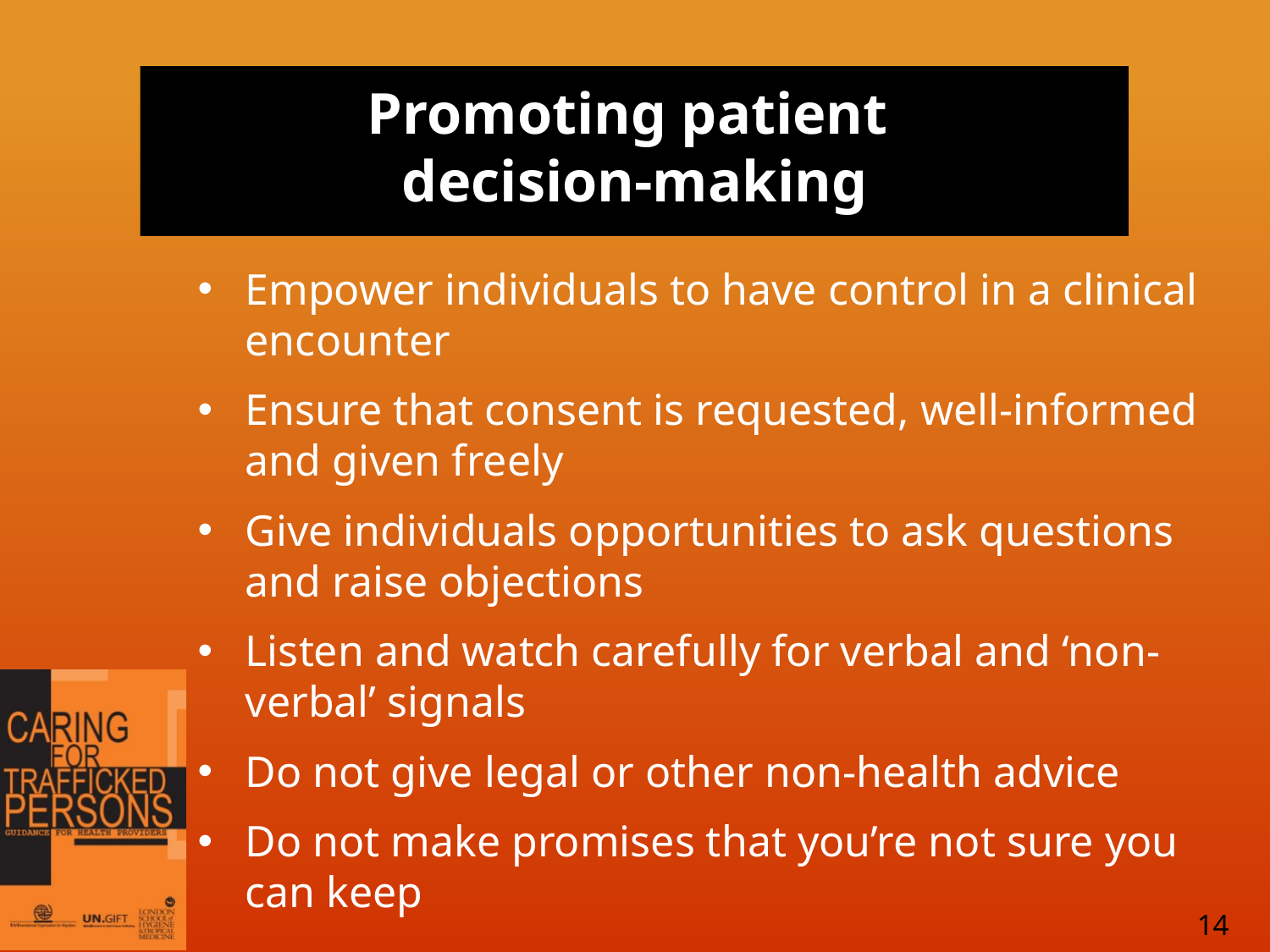

# Promoting patient decision-making
Empower individuals to have control in a clinical encounter
Ensure that consent is requested, well-informed and given freely
Give individuals opportunities to ask questions and raise objections
Listen and watch carefully for verbal and ‘non-verbal’ signals
Do not give legal or other non-health advice
Do not make promises that you’re not sure you can keep
14

## Slide 15
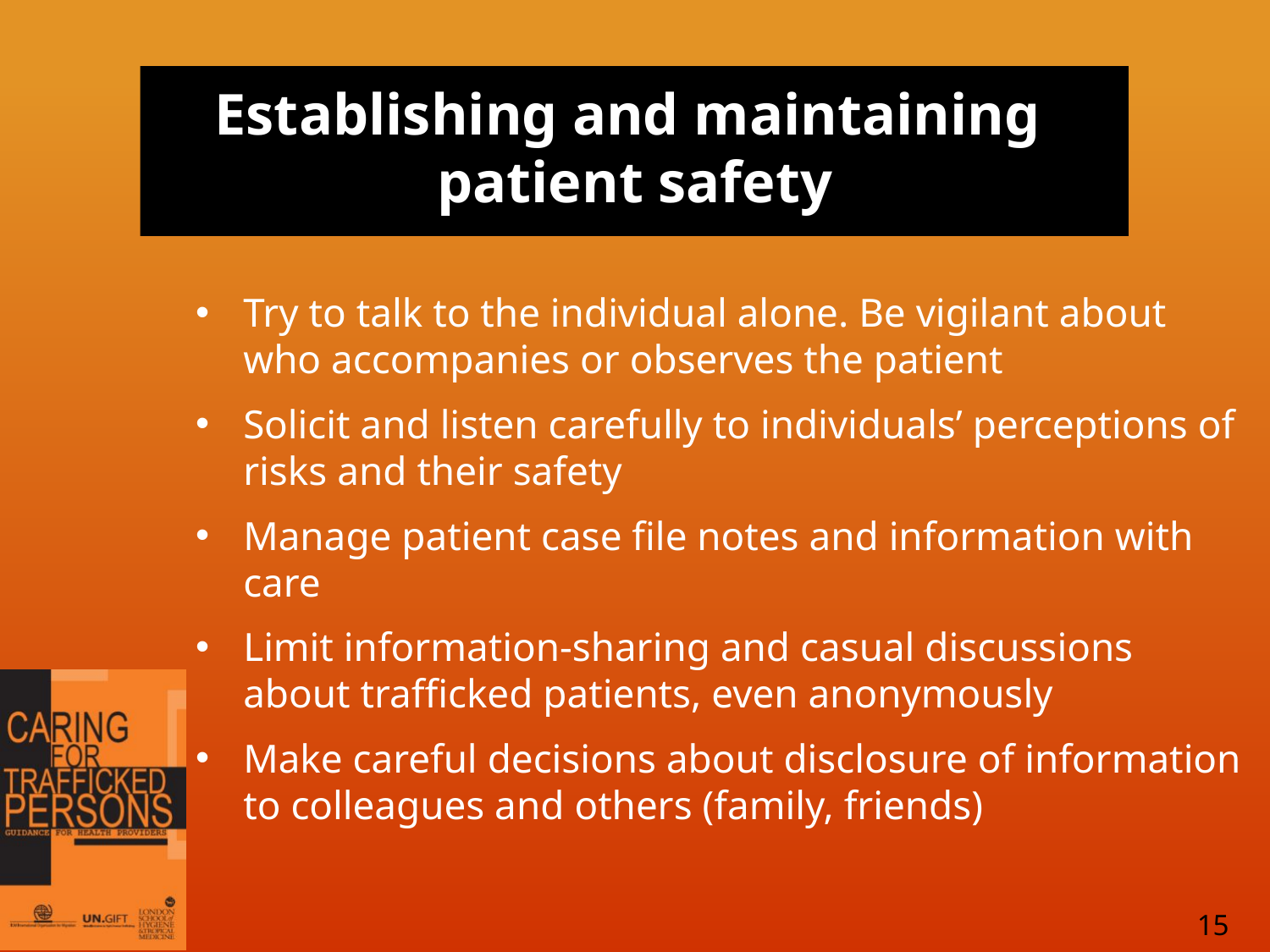

# Establishing and maintaining patient safety
Try to talk to the individual alone. Be vigilant about who accompanies or observes the patient
Solicit and listen carefully to individuals’ perceptions of risks and their safety
Manage patient case file notes and information with care
Limit information-sharing and casual discussions about trafficked patients, even anonymously
Make careful decisions about disclosure of information to colleagues and others (family, friends)
15

## Slide 16
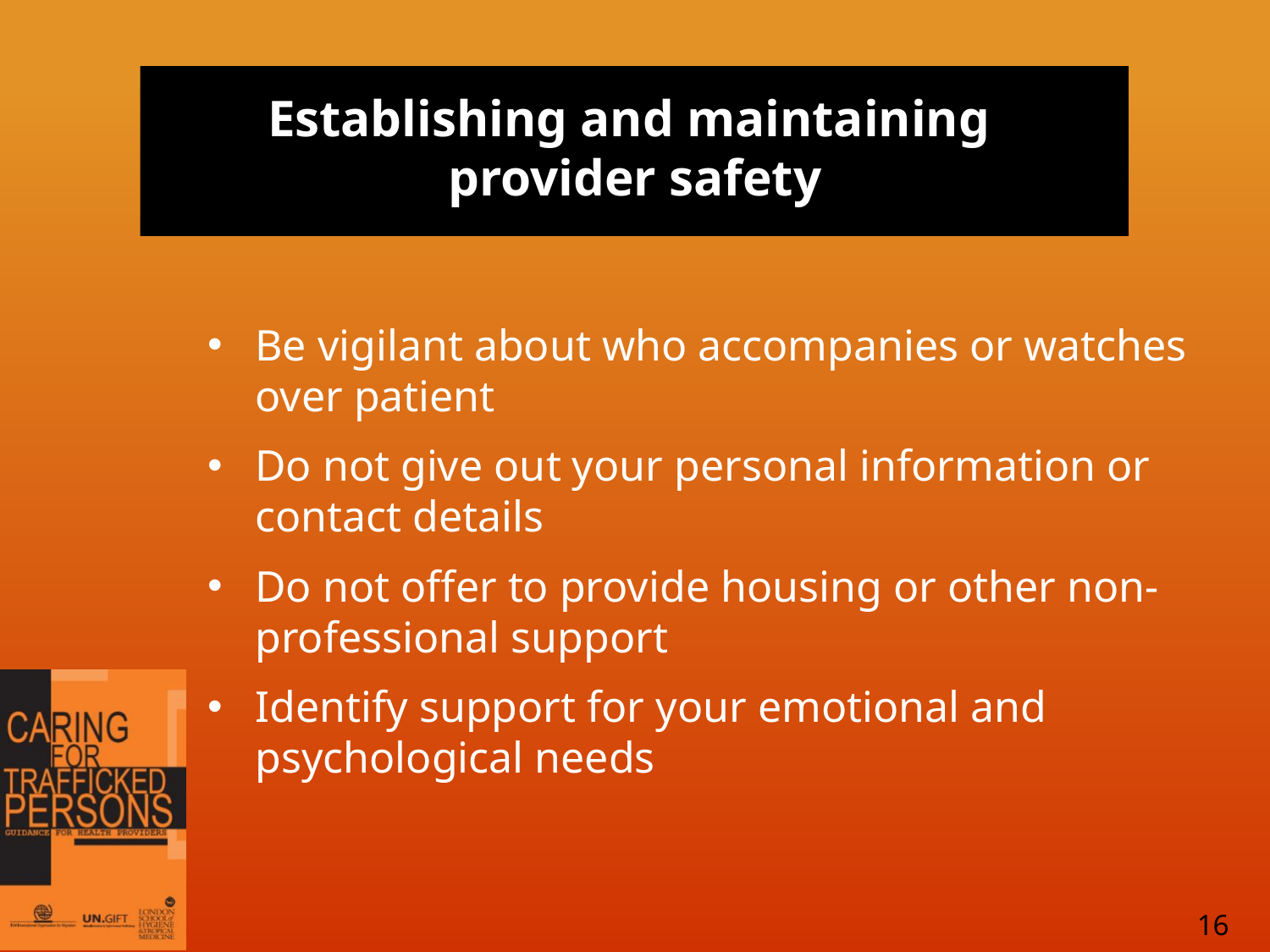

# Establishing and maintaining provider safety
Be vigilant about who accompanies or watches over patient
Do not give out your personal information or contact details
Do not offer to provide housing or other non-professional support
Identify support for your emotional and psychological needs
16

## Slide 17
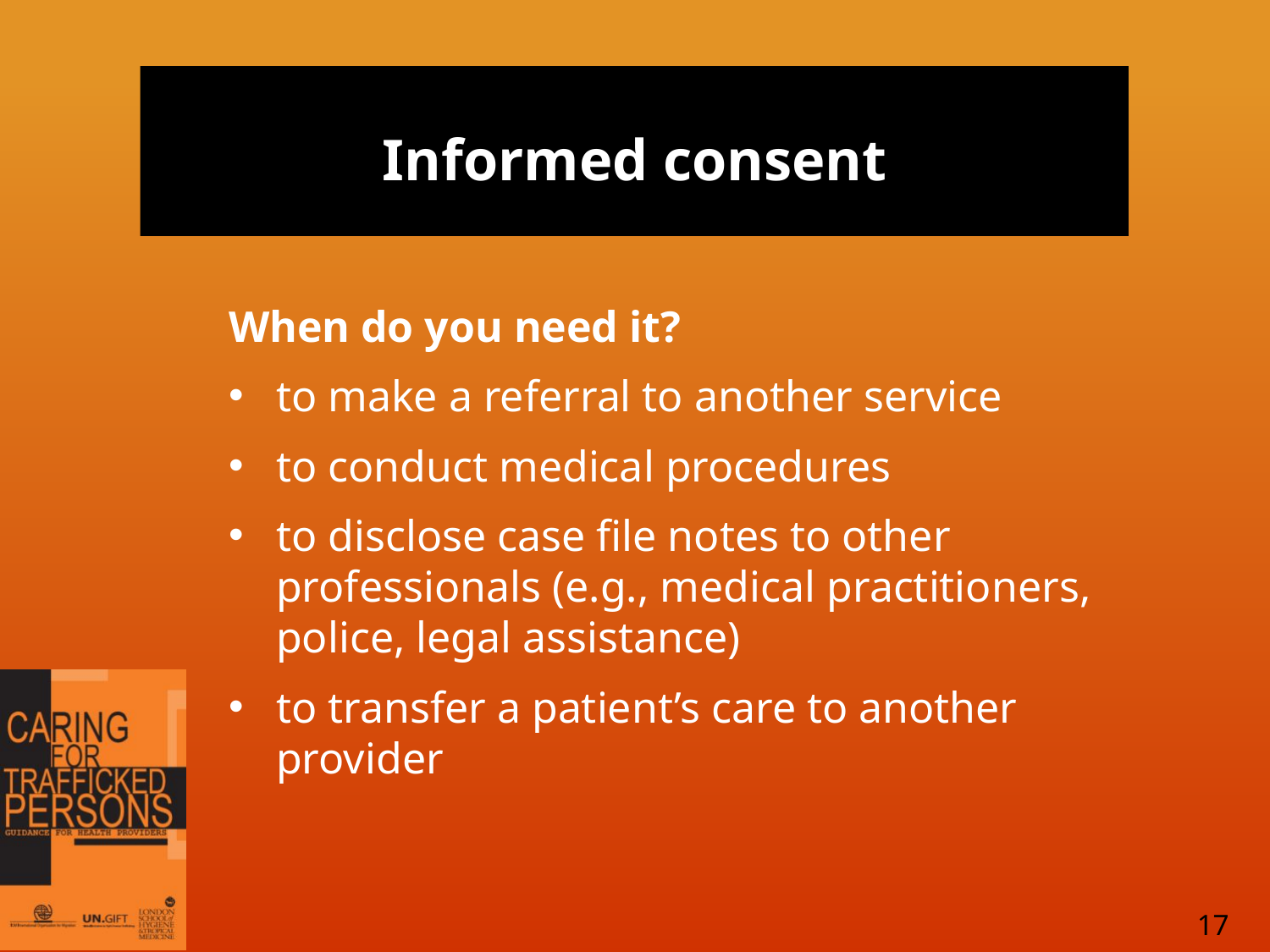

# Informed consent
When do you need it?
to make a referral to another service
to conduct medical procedures
to disclose case file notes to other professionals (e.g., medical practitioners, police, legal assistance)
to transfer a patient’s care to another provider
17

## Slide 18
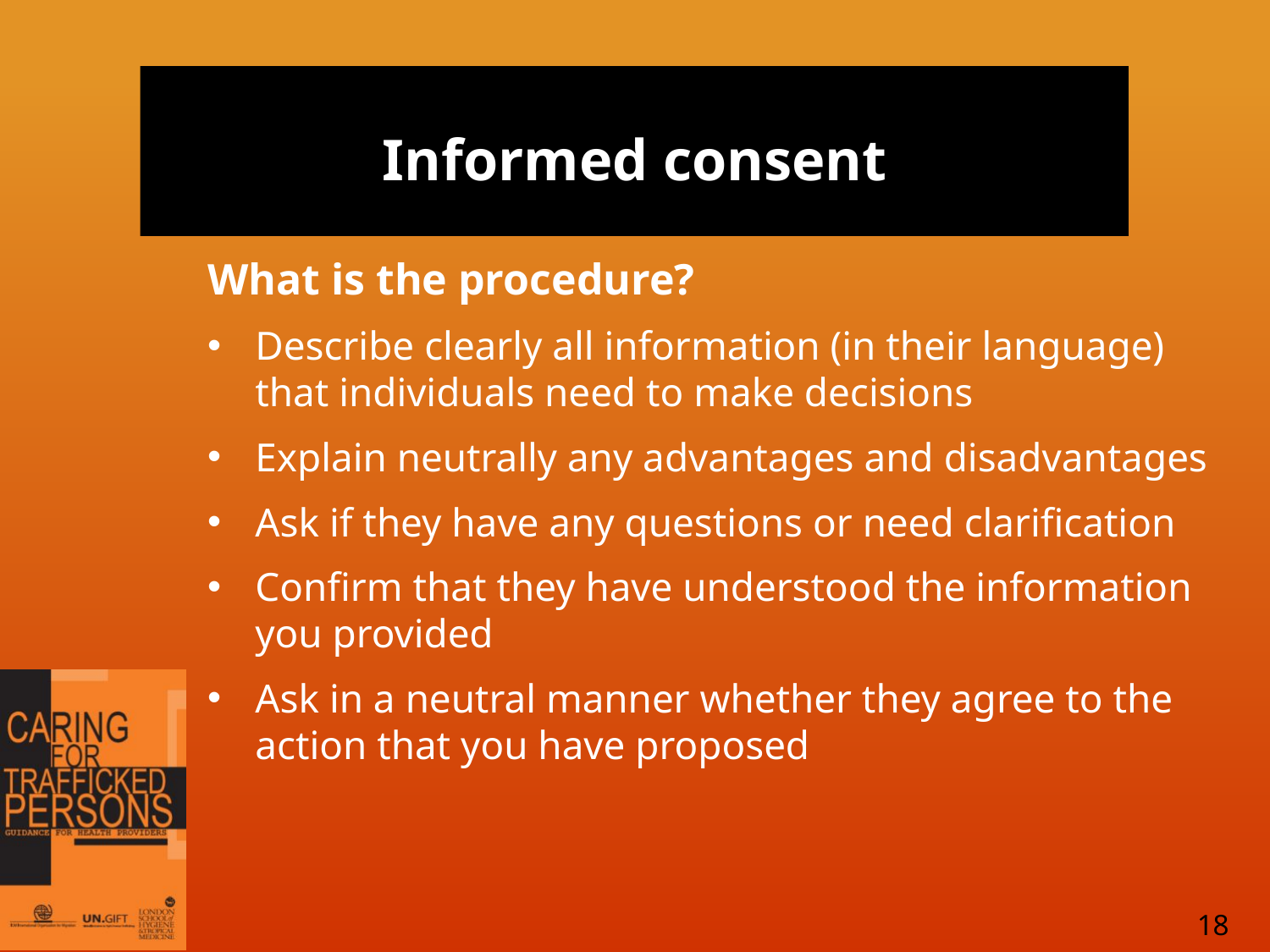

# Informed consent
What is the procedure?
Describe clearly all information (in their language) that individuals need to make decisions
Explain neutrally any advantages and disadvantages
Ask if they have any questions or need clarification
Confirm that they have understood the information you provided
Ask in a neutral manner whether they agree to the action that you have proposed
18

## Slide 19
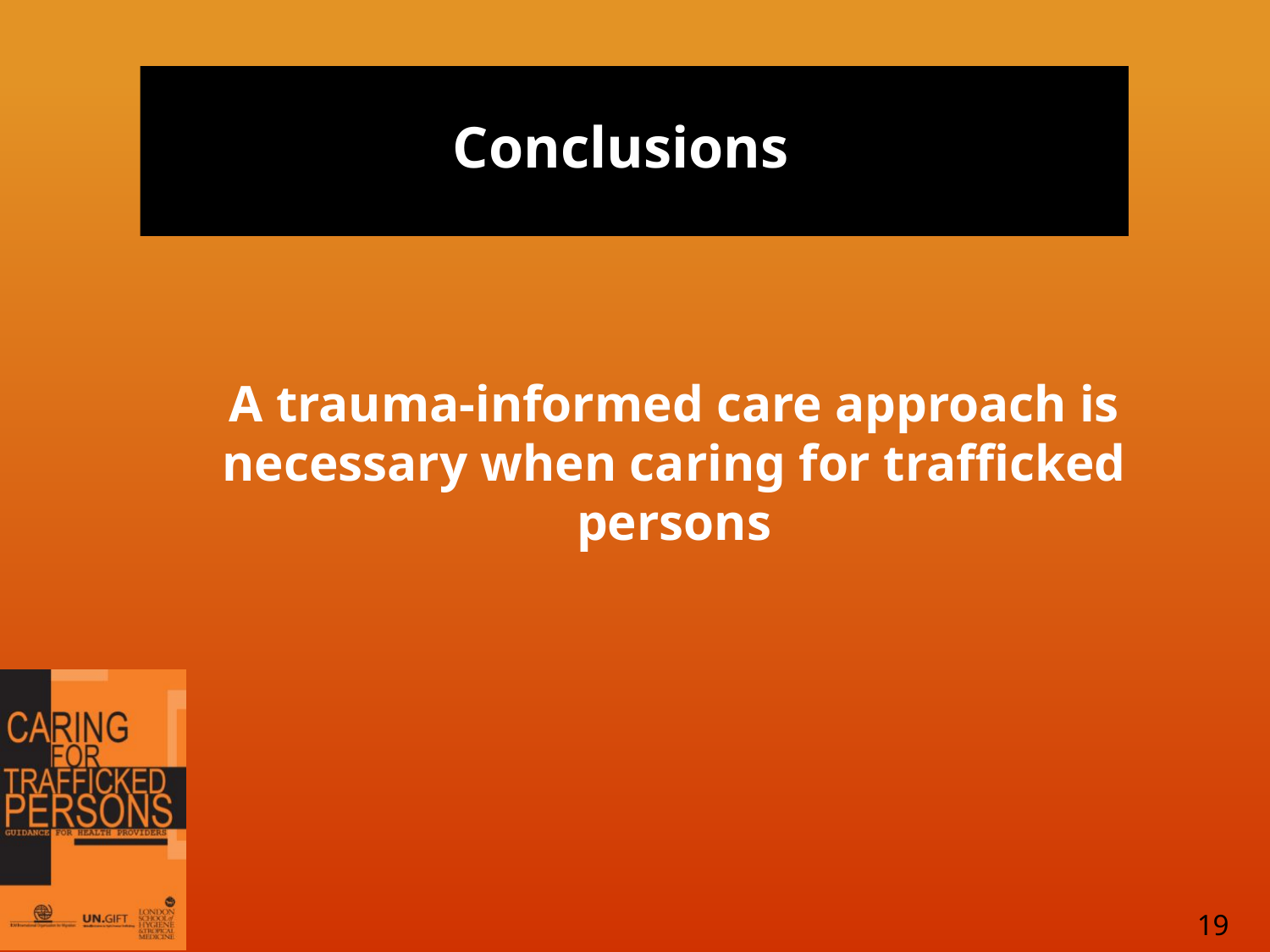

# Conclusions
A trauma-informed care approach is necessary when caring for trafficked persons
19

## Slide 20
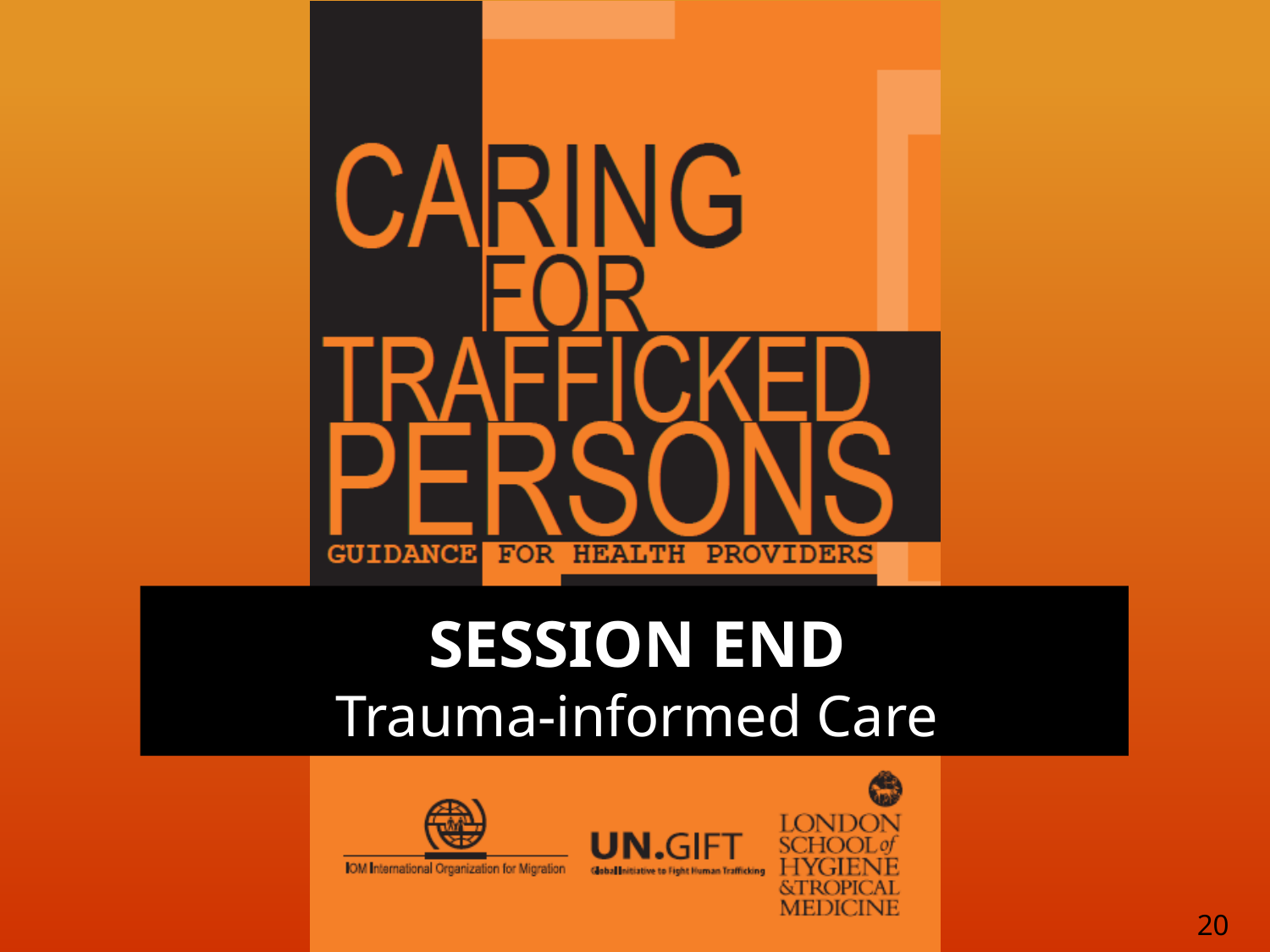

SESSION END
Trauma-informed Care
20
