# Supplementary material for: Health Care Providers and Human Trafficking: What do They Know, What do They Need to Know? Findings from the Middle East, the Caribbean, and Central America
Source: Front Public Health. 2015 Jan 29;3:6. doi: 10.3389/fpubh.2015.00006 (PMC4310216; doi:10.3389/fpubh.2015.00006)
Supplement: Supplementary file 1 [file Presentation_1.ZIP › Caring for Trafficked Persons Training Session 4.pptx]

## Slide 1
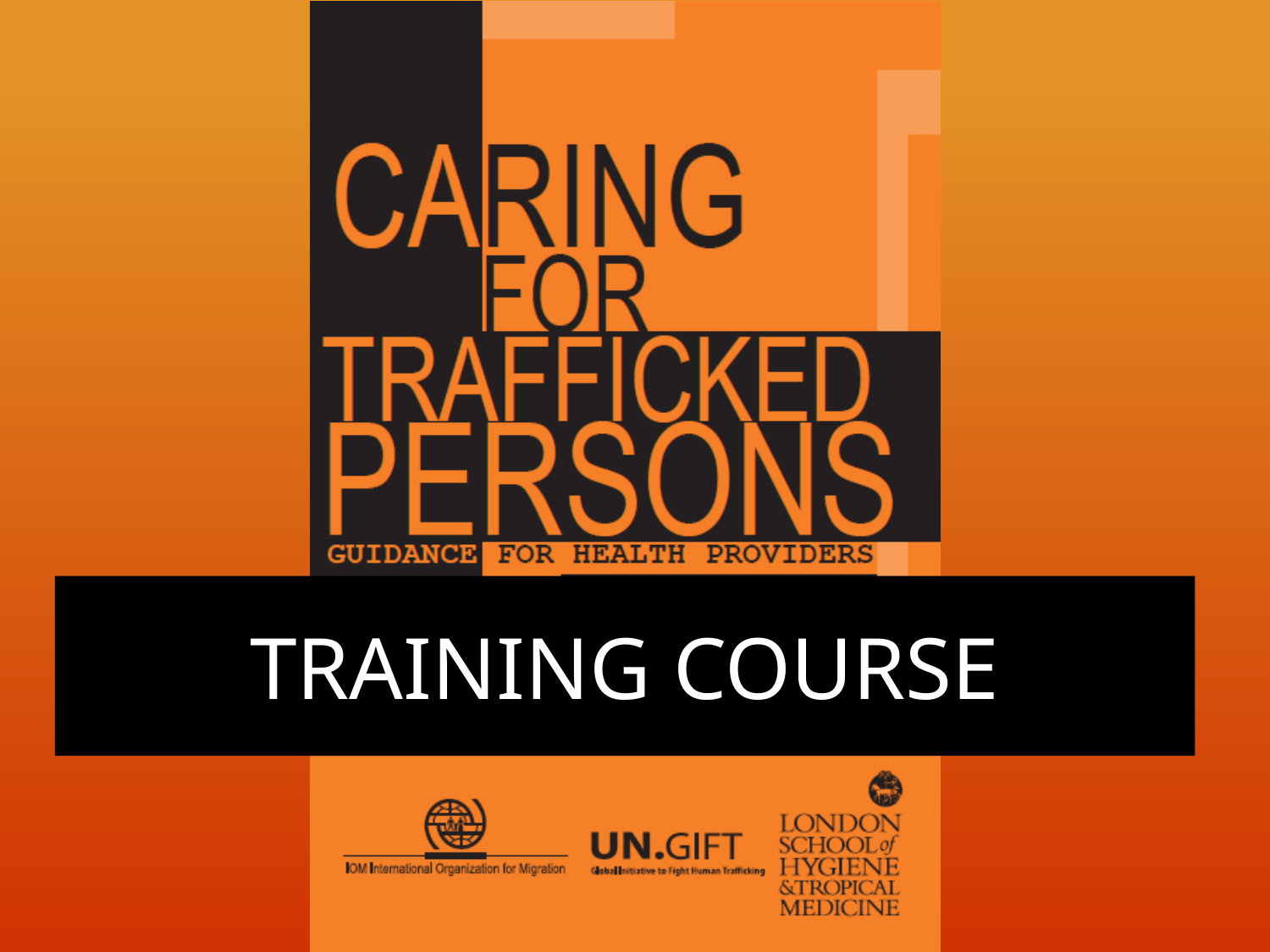

## Slide 2
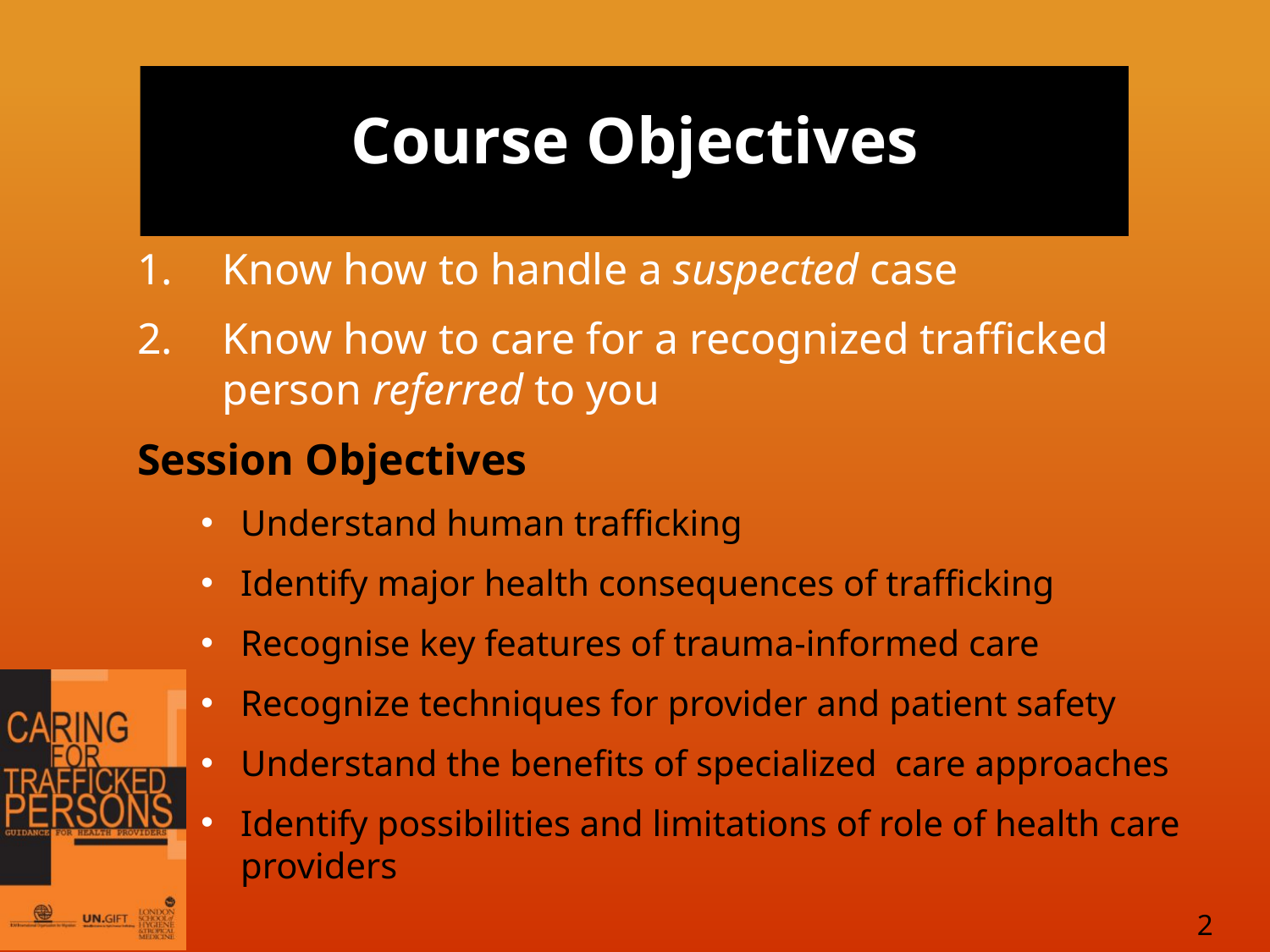

# Course Objectives
Know how to handle a suspected case
Know how to care for a recognized trafficked person referred to you
Session Objectives
Understand human trafficking
Identify major health consequences of trafficking
Recognise key features of trauma-informed care
Recognize techniques for provider and patient safety
Understand the benefits of specialized care approaches
Identify possibilities and limitations of role of health care providers
2

## Slide 3
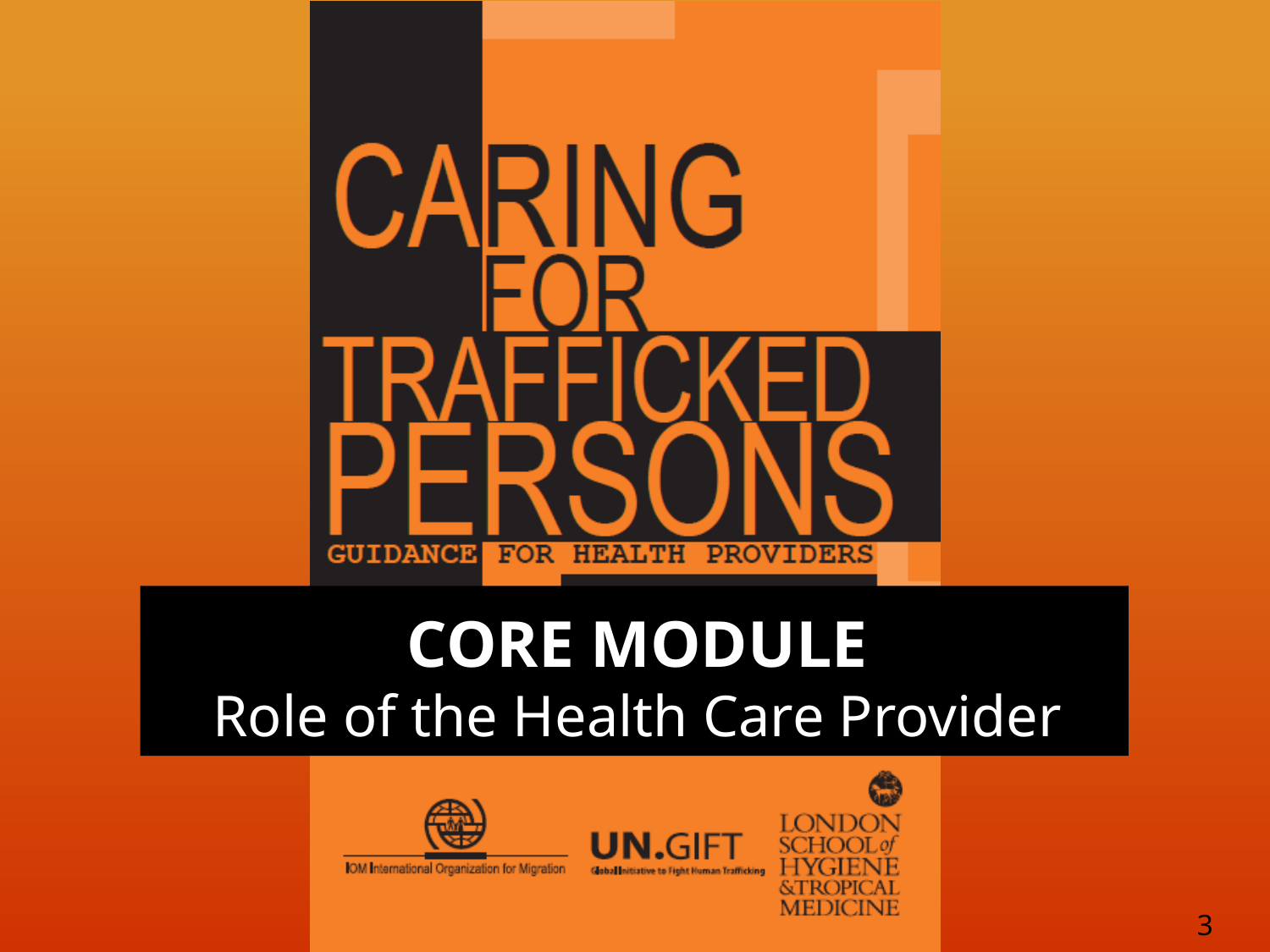

CORE MODULE
Role of the Health Care Provider
3

## Slide 4
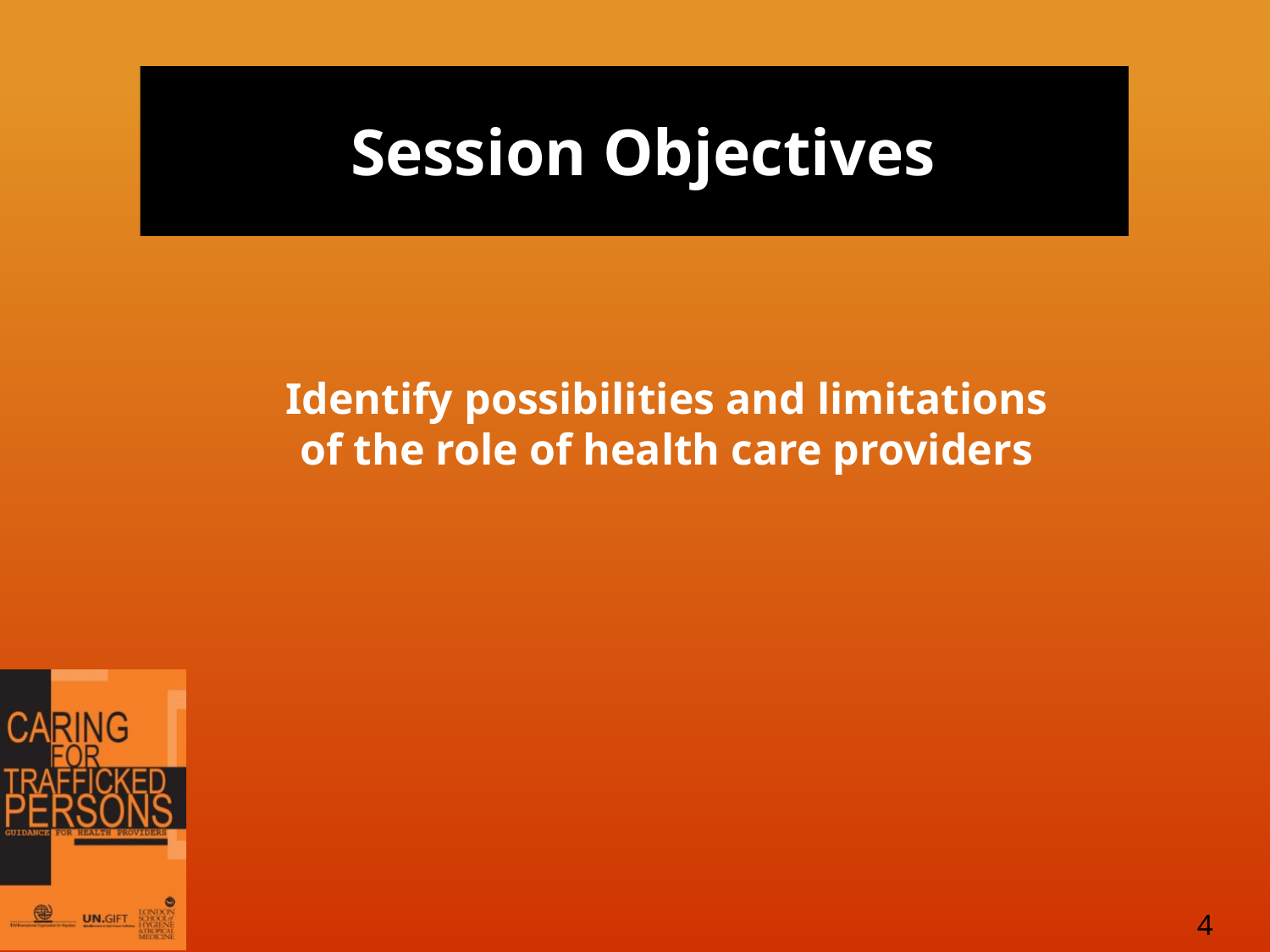

# Session Objectives
Identify possibilities and limitations of the role of health care providers
4

## Slide 5
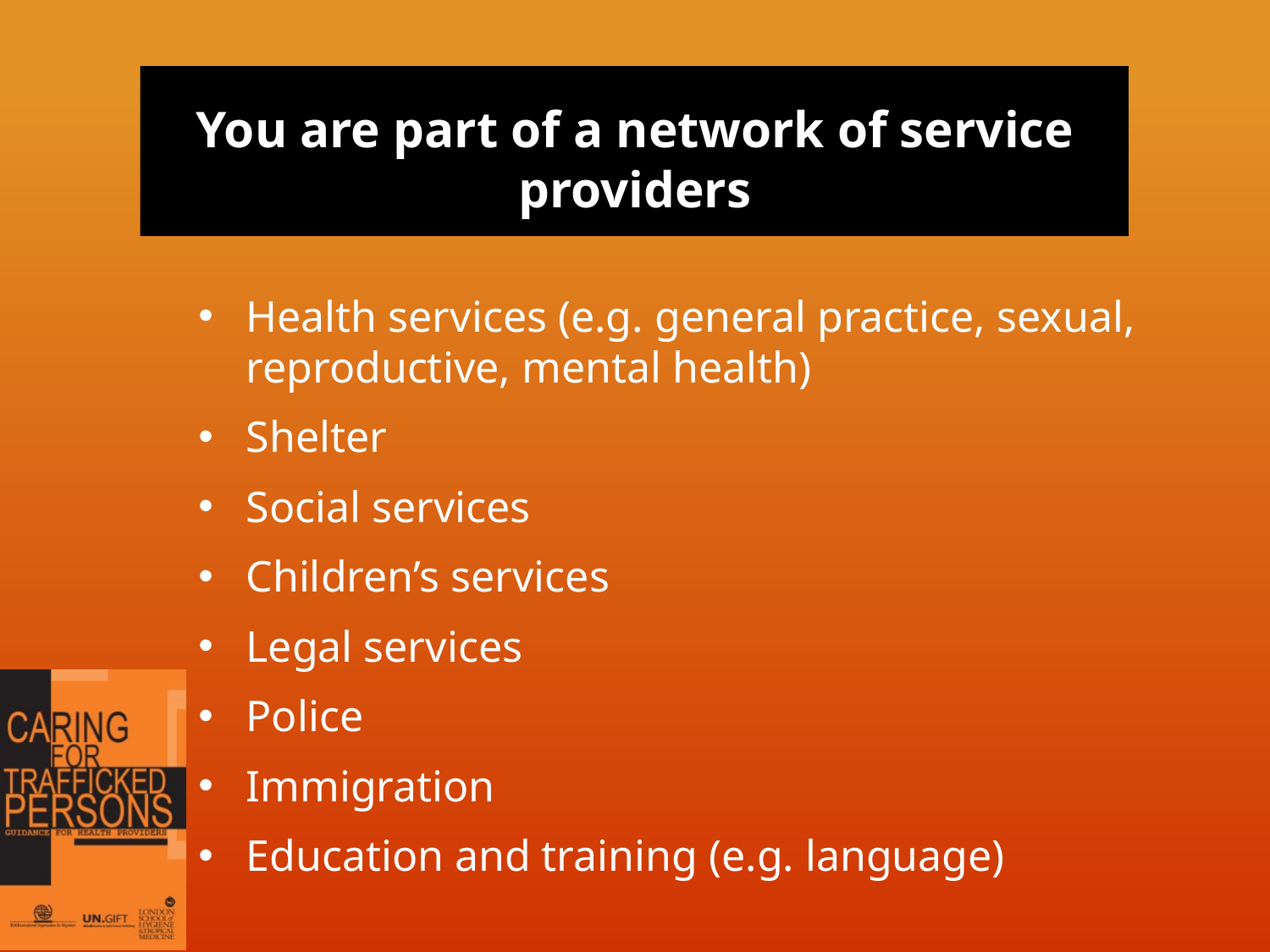

# You are part of a network of service providers
Health services (e.g. general practice, sexual, reproductive, mental health)
Shelter
Social services
Children’s services
Legal services
Police
Immigration
Education and training (e.g. language)

## Slide 6
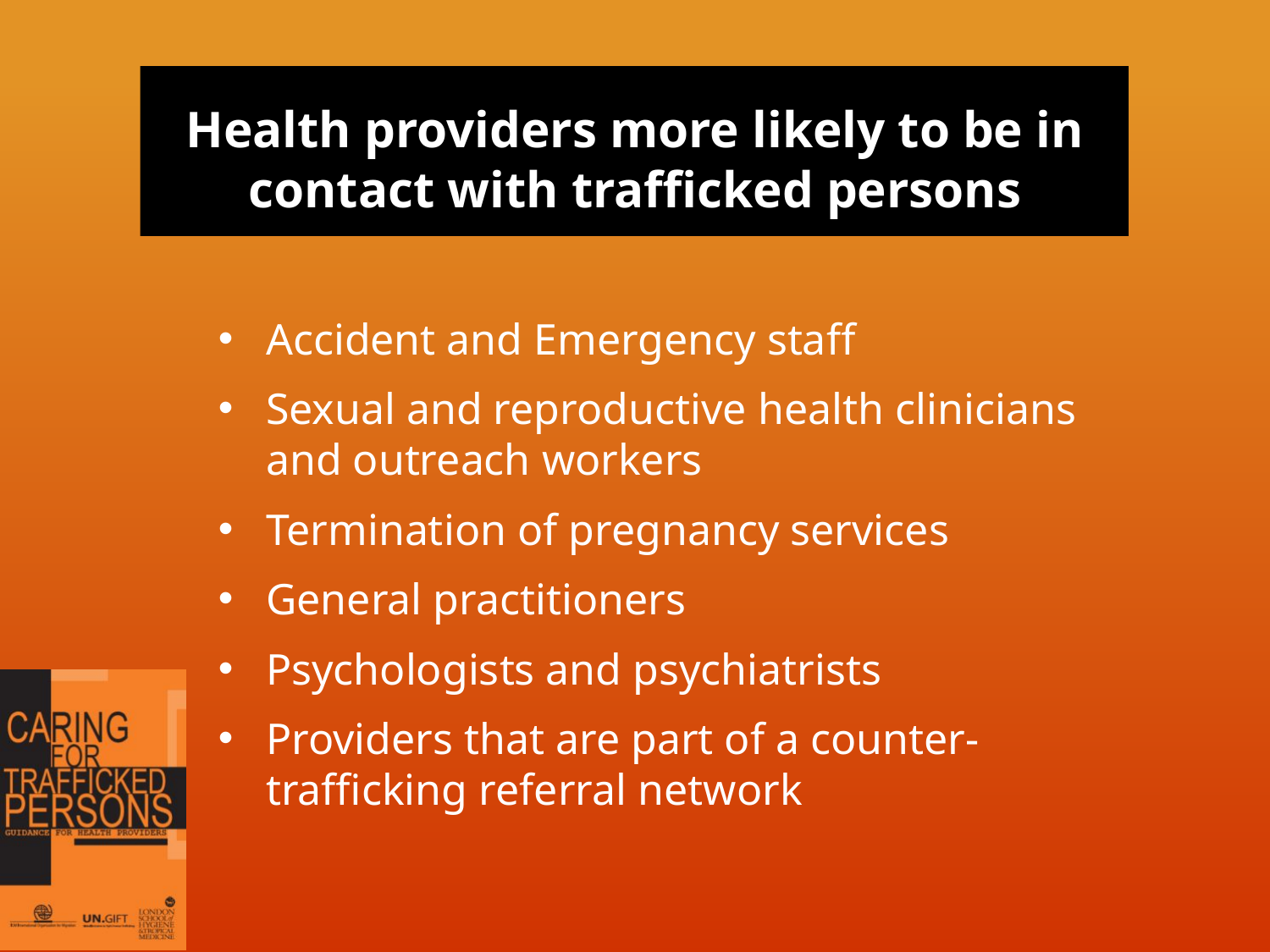

# Health providers more likely to be in contact with trafficked persons
Accident and Emergency staff
Sexual and reproductive health clinicians and outreach workers
Termination of pregnancy services
General practitioners
Psychologists and psychiatrists
Providers that are part of a counter-trafficking referral network

## Slide 7
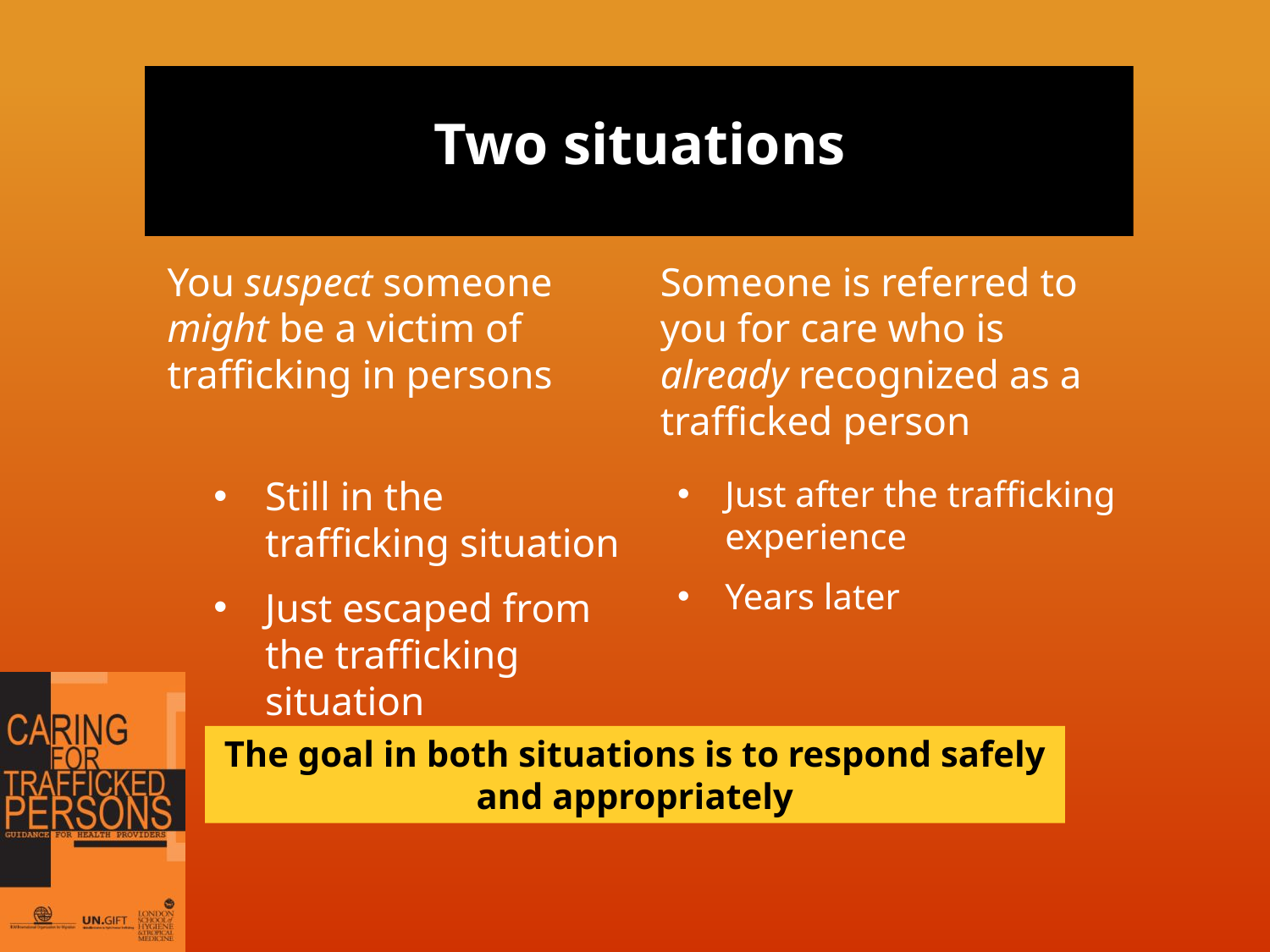

Two situations
You suspect someone might be a victim of trafficking in persons
Someone is referred to you for care who is already recognized as a trafficked person
Still in the trafficking situation
Just escaped from the trafficking situation
Just after the trafficking experience
Years later
The goal in both situations is to respond safely and appropriately

## Slide 8
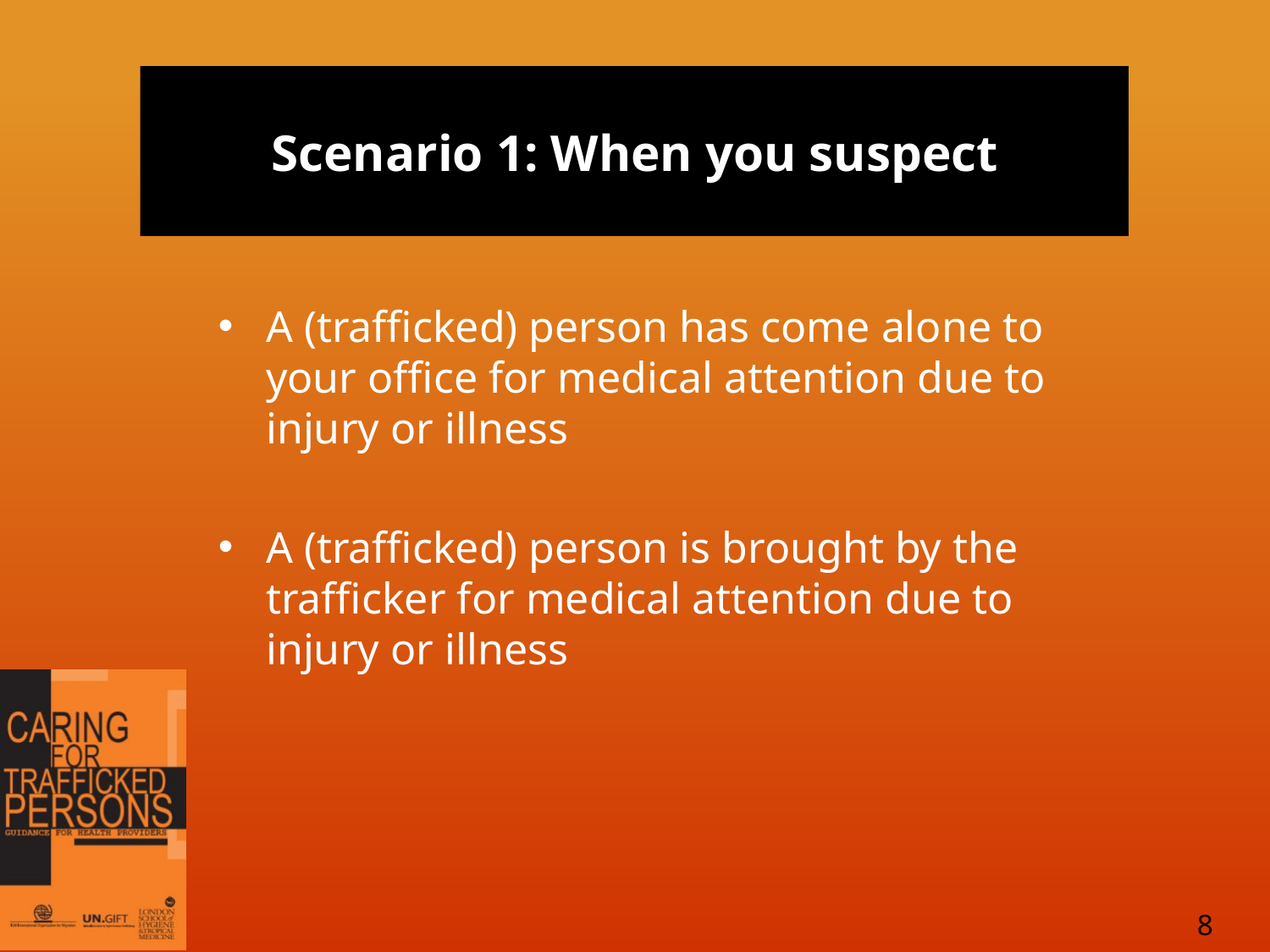

# Scenario 1: When you suspect
A (trafficked) person has come alone to your office for medical attention due to injury or illness
A (trafficked) person is brought by the trafficker for medical attention due to injury or illness
8

## Slide 9
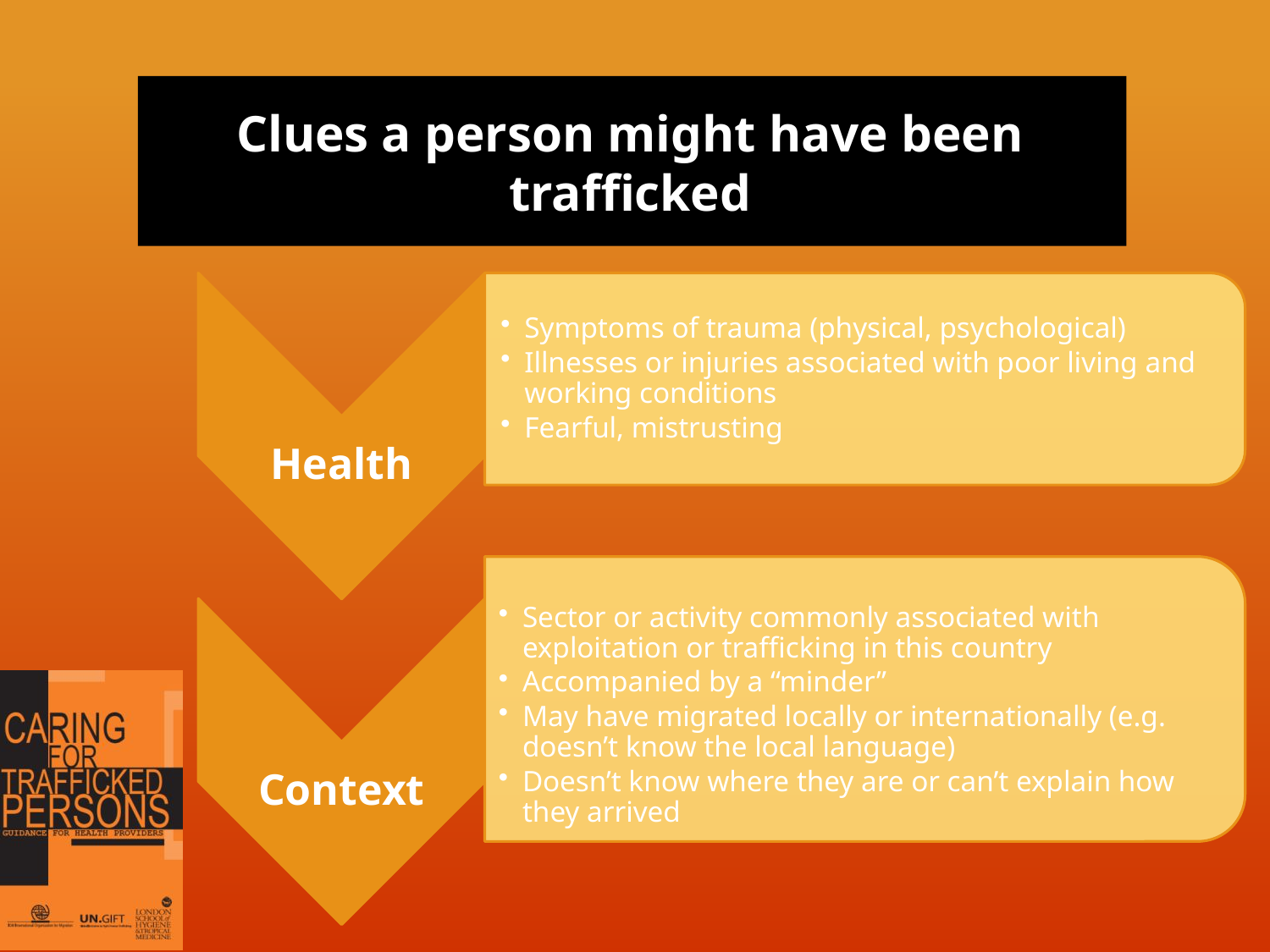

Clues a person might have been trafficked

## Slide 10
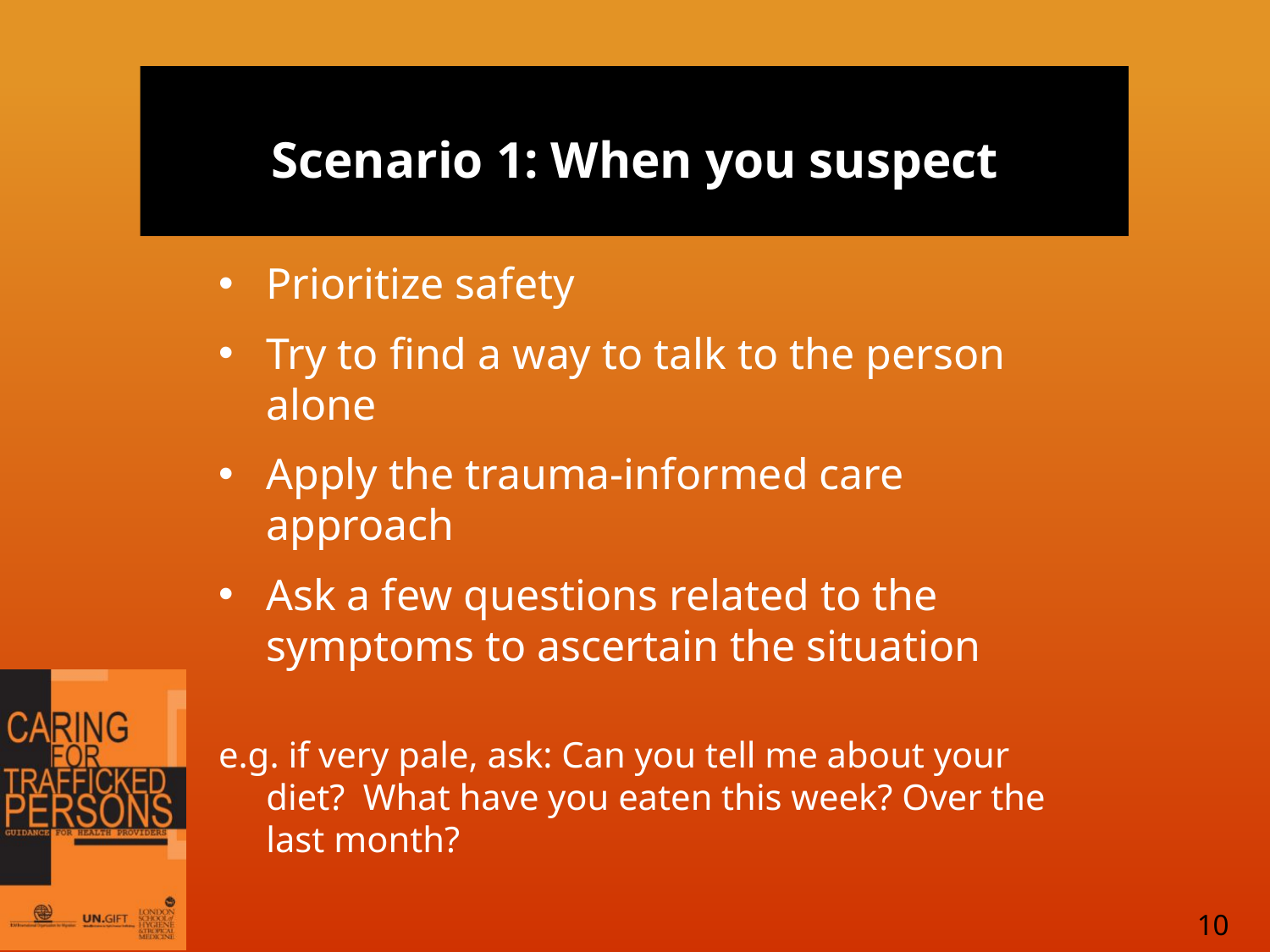

# Scenario 1: When you suspect
Prioritize safety
Try to find a way to talk to the person alone
Apply the trauma-informed care approach
Ask a few questions related to the symptoms to ascertain the situation
e.g. if very pale, ask: Can you tell me about your diet? What have you eaten this week? Over the last month?
10

## Slide 11
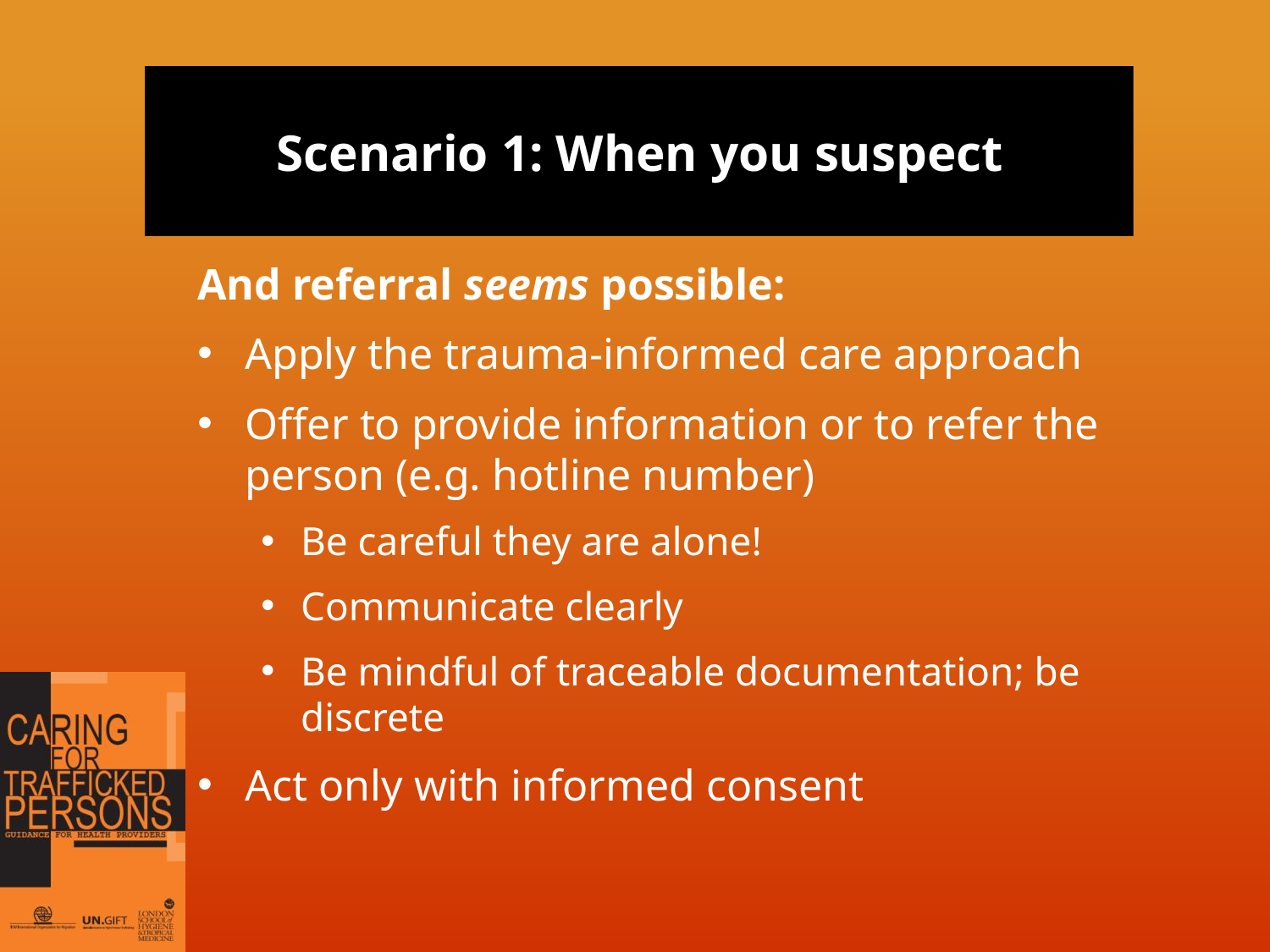

Scenario 1: When you suspect
And referral seems possible:
Apply the trauma-informed care approach
Offer to provide information or to refer the person (e.g. hotline number)
Be careful they are alone!
Communicate clearly
Be mindful of traceable documentation; be discrete
Act only with informed consent

## Slide 12
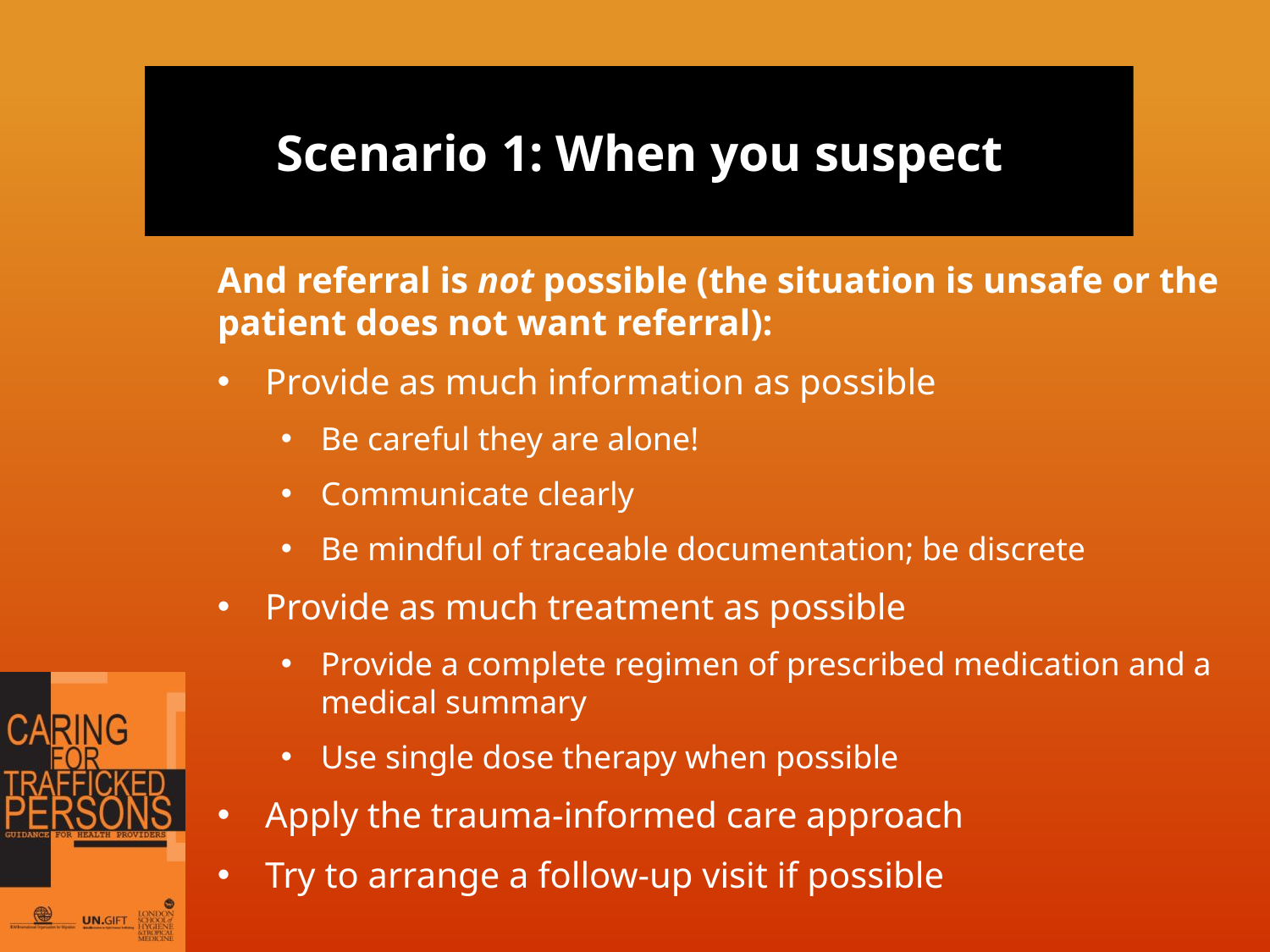

Scenario 1: When you suspect
And referral is not possible (the situation is unsafe or the patient does not want referral):
Provide as much information as possible
Be careful they are alone!
Communicate clearly
Be mindful of traceable documentation; be discrete
Provide as much treatment as possible
Provide a complete regimen of prescribed medication and a medical summary
Use single dose therapy when possible
Apply the trauma-informed care approach
Try to arrange a follow-up visit if possible

## Slide 13
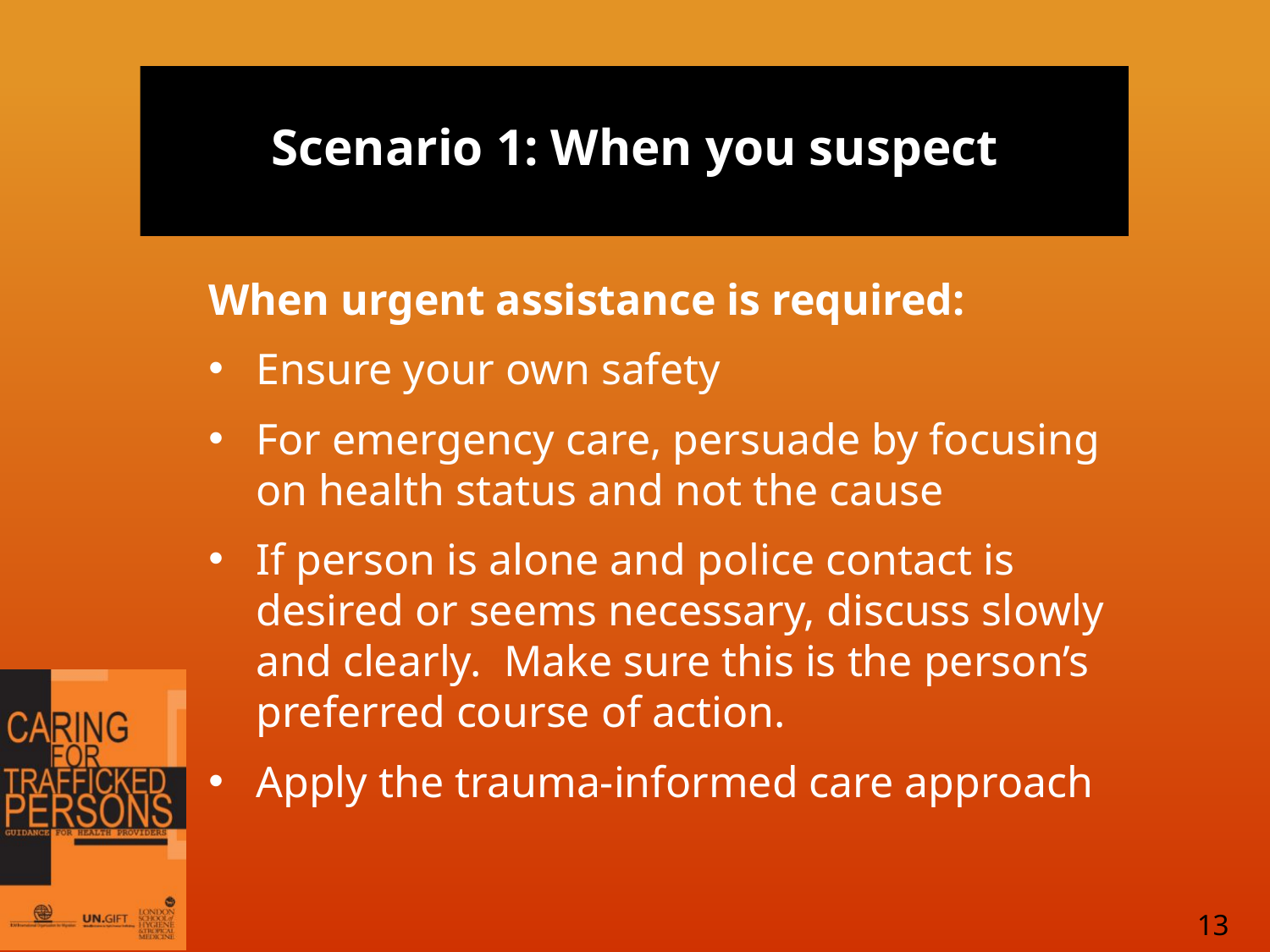

# Scenario 1: When you suspect
When urgent assistance is required:
Ensure your own safety
For emergency care, persuade by focusing on health status and not the cause
If person is alone and police contact is desired or seems necessary, discuss slowly and clearly. Make sure this is the person’s preferred course of action.
Apply the trauma-informed care approach
13

## Slide 14
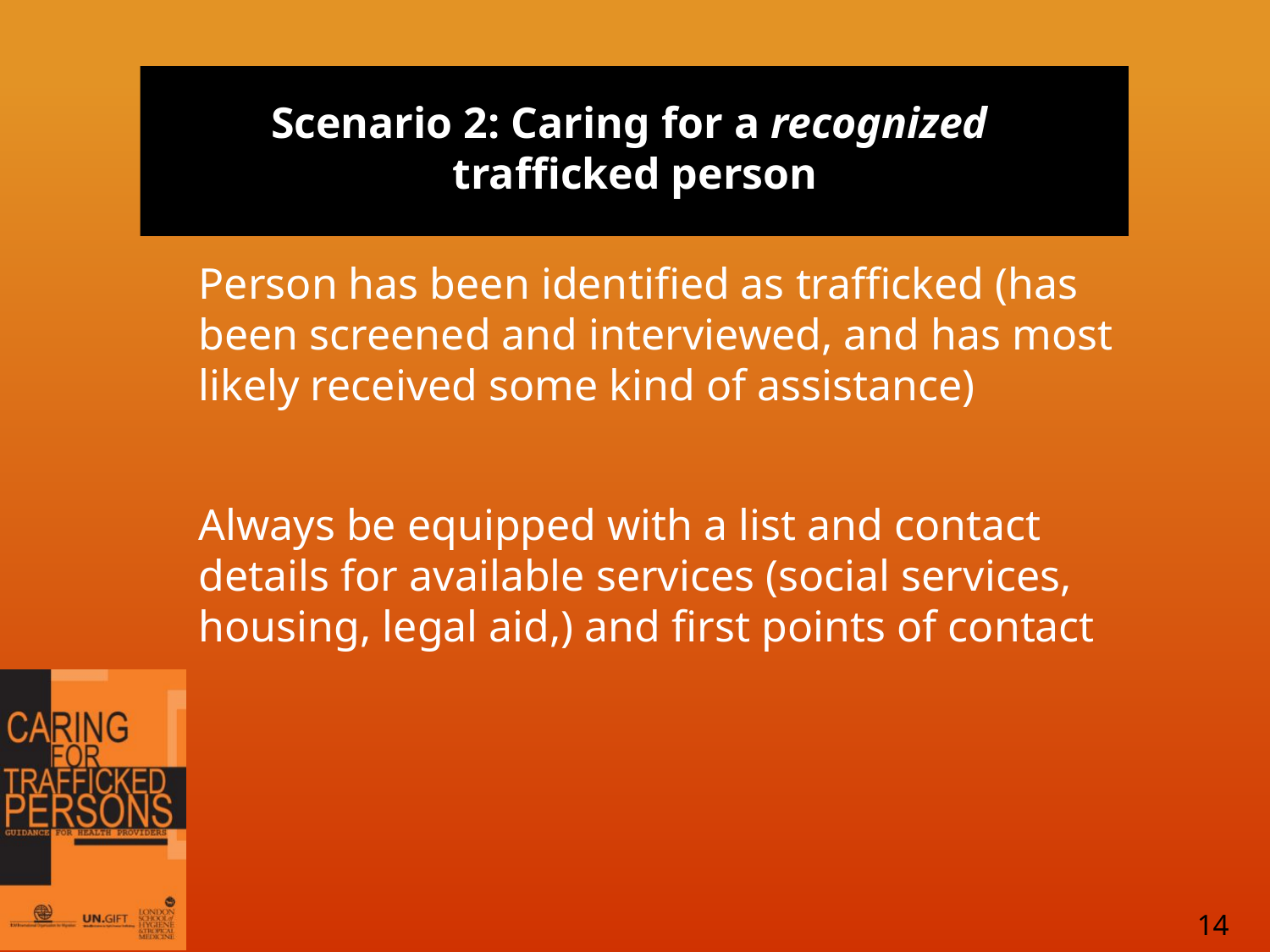

# Scenario 2: Caring for a recognized trafficked person
Person has been identified as trafficked (has been screened and interviewed, and has most likely received some kind of assistance)
Always be equipped with a list and contact details for available services (social services, housing, legal aid,) and first points of contact
14

## Slide 15
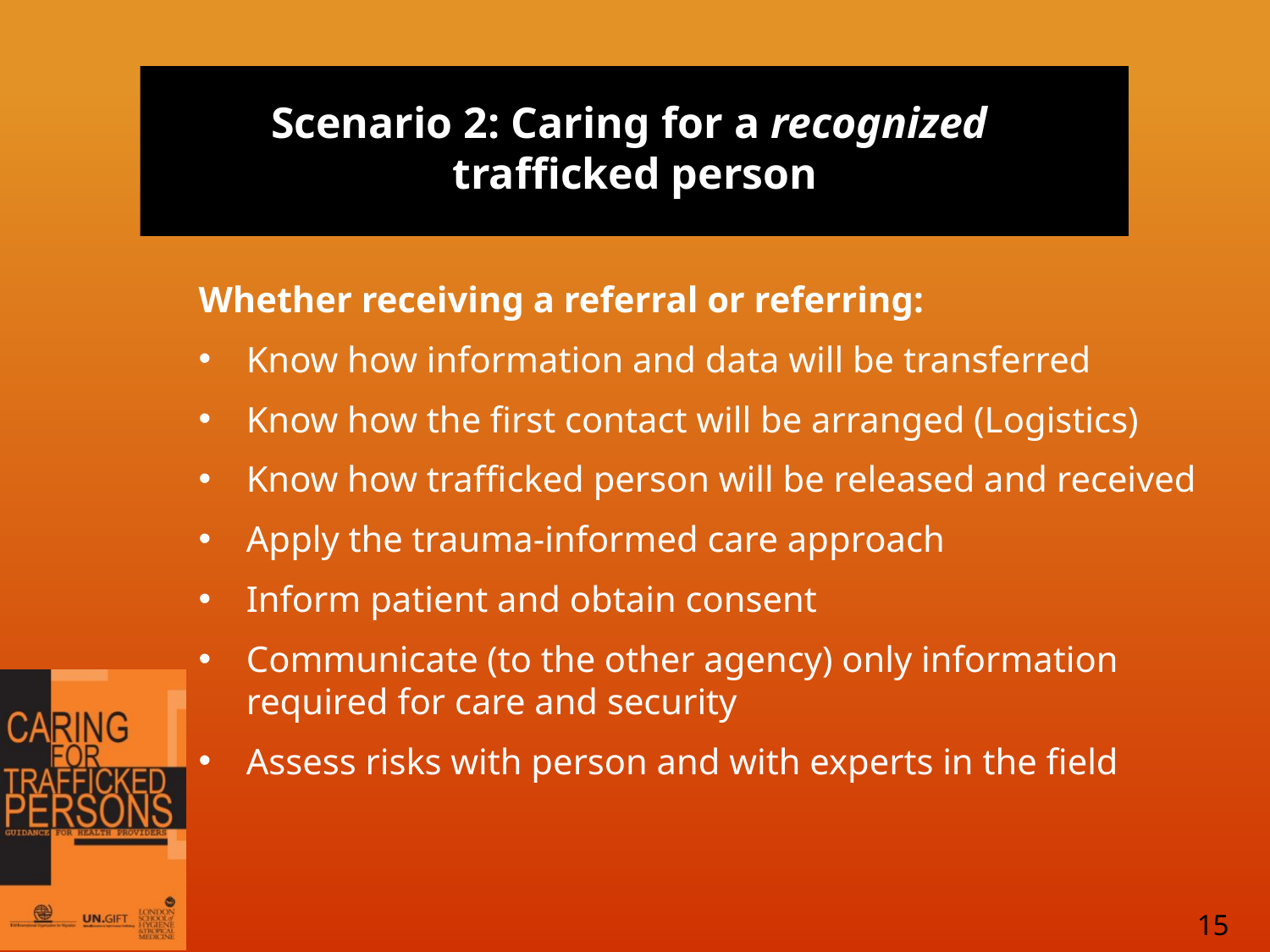

# Scenario 2: Caring for a recognized trafficked person
Whether receiving a referral or referring:
Know how information and data will be transferred
Know how the first contact will be arranged (Logistics)
Know how trafficked person will be released and received
Apply the trauma-informed care approach
Inform patient and obtain consent
Communicate (to the other agency) only information required for care and security
Assess risks with person and with experts in the field
15

## Slide 16
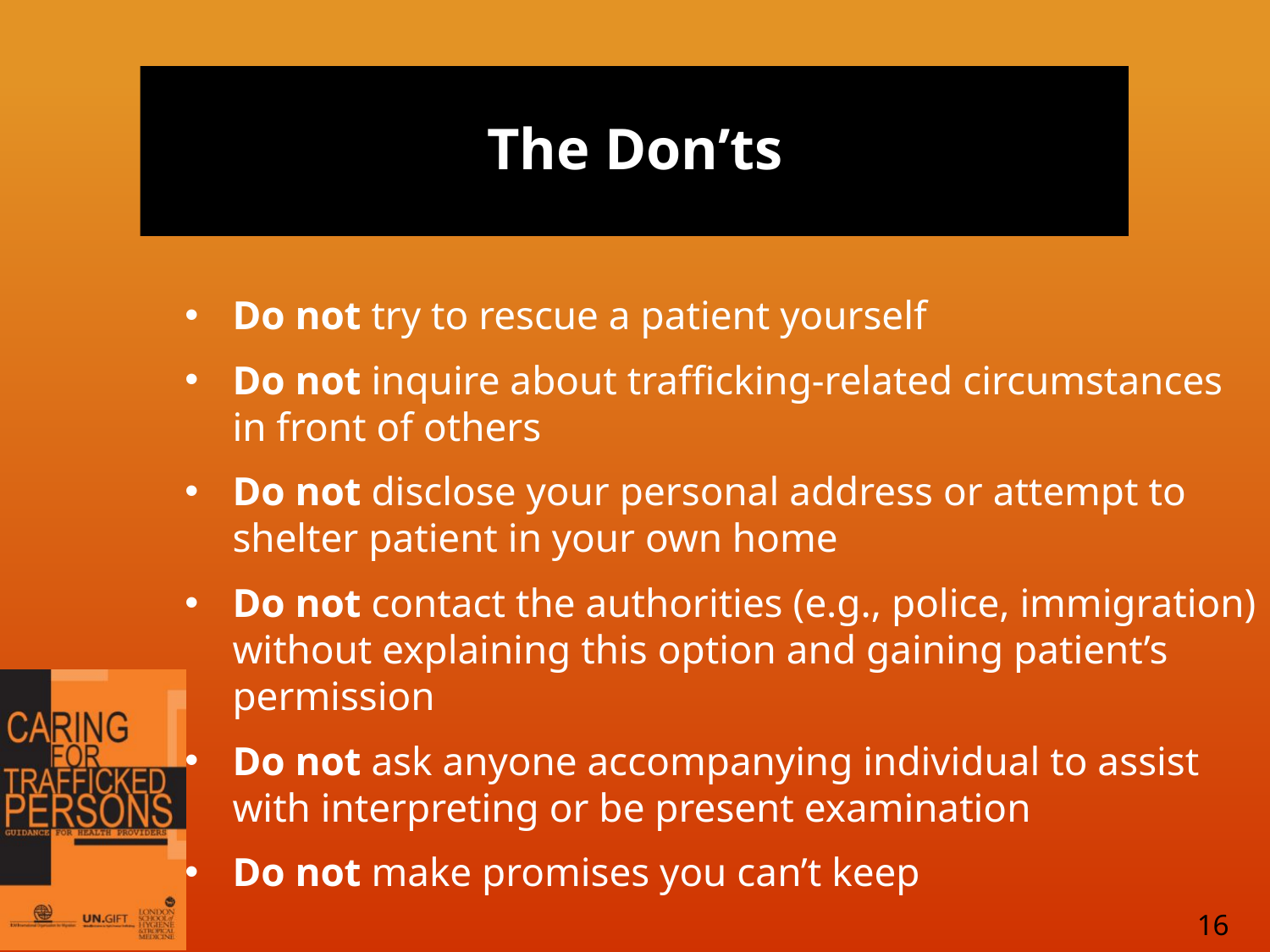

# The Don’ts
Do not try to rescue a patient yourself
Do not inquire about trafficking-related circumstances in front of others
Do not disclose your personal address or attempt to shelter patient in your own home
Do not contact the authorities (e.g., police, immigration) without explaining this option and gaining patient’s permission
Do not ask anyone accompanying individual to assist with interpreting or be present examination
Do not make promises you can’t keep
16

## Slide 17
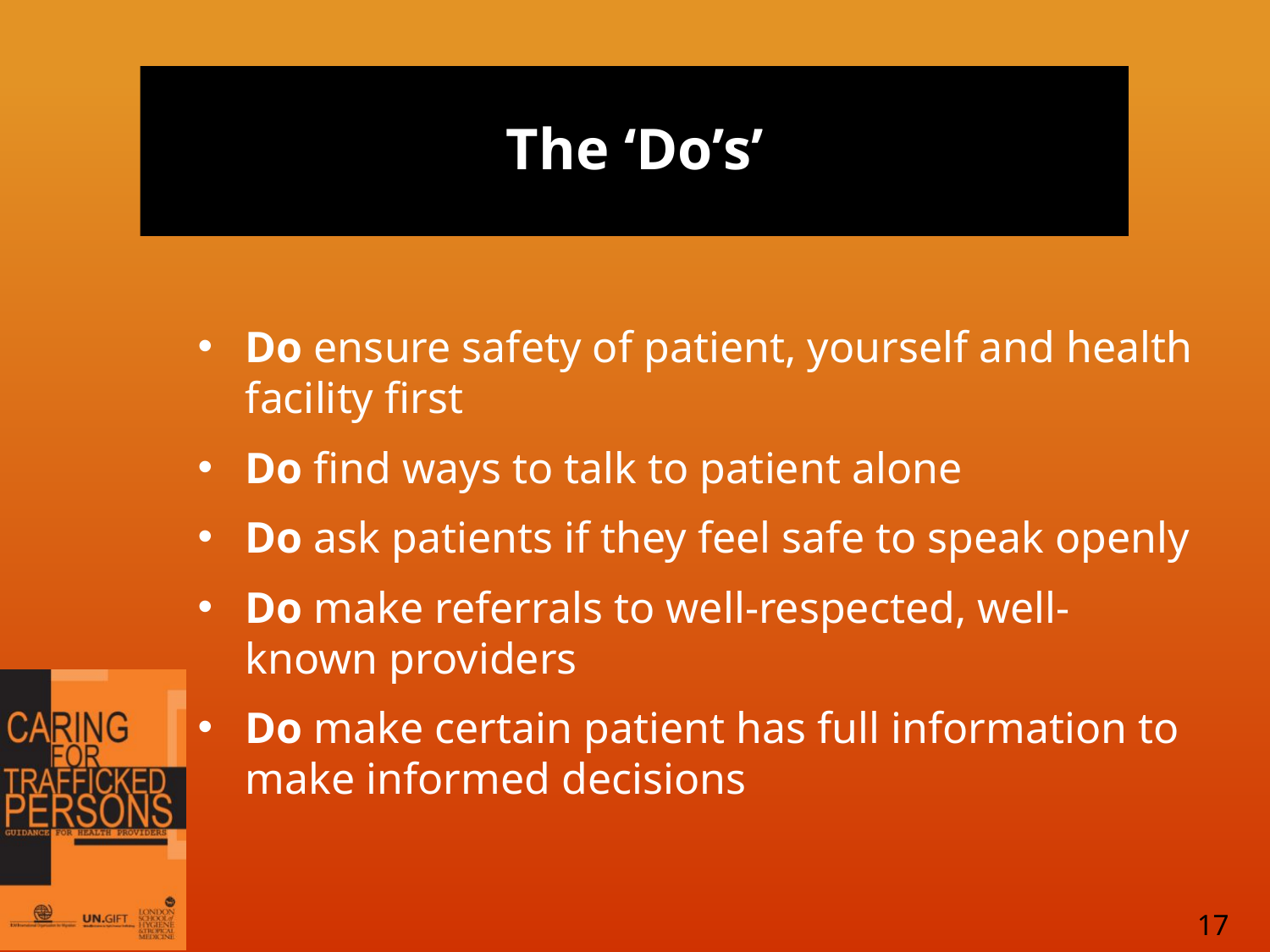

# The ‘Do’s’
Do ensure safety of patient, yourself and health facility first
Do find ways to talk to patient alone
Do ask patients if they feel safe to speak openly
Do make referrals to well-respected, well-known providers
Do make certain patient has full information to make informed decisions
17

## Slide 18
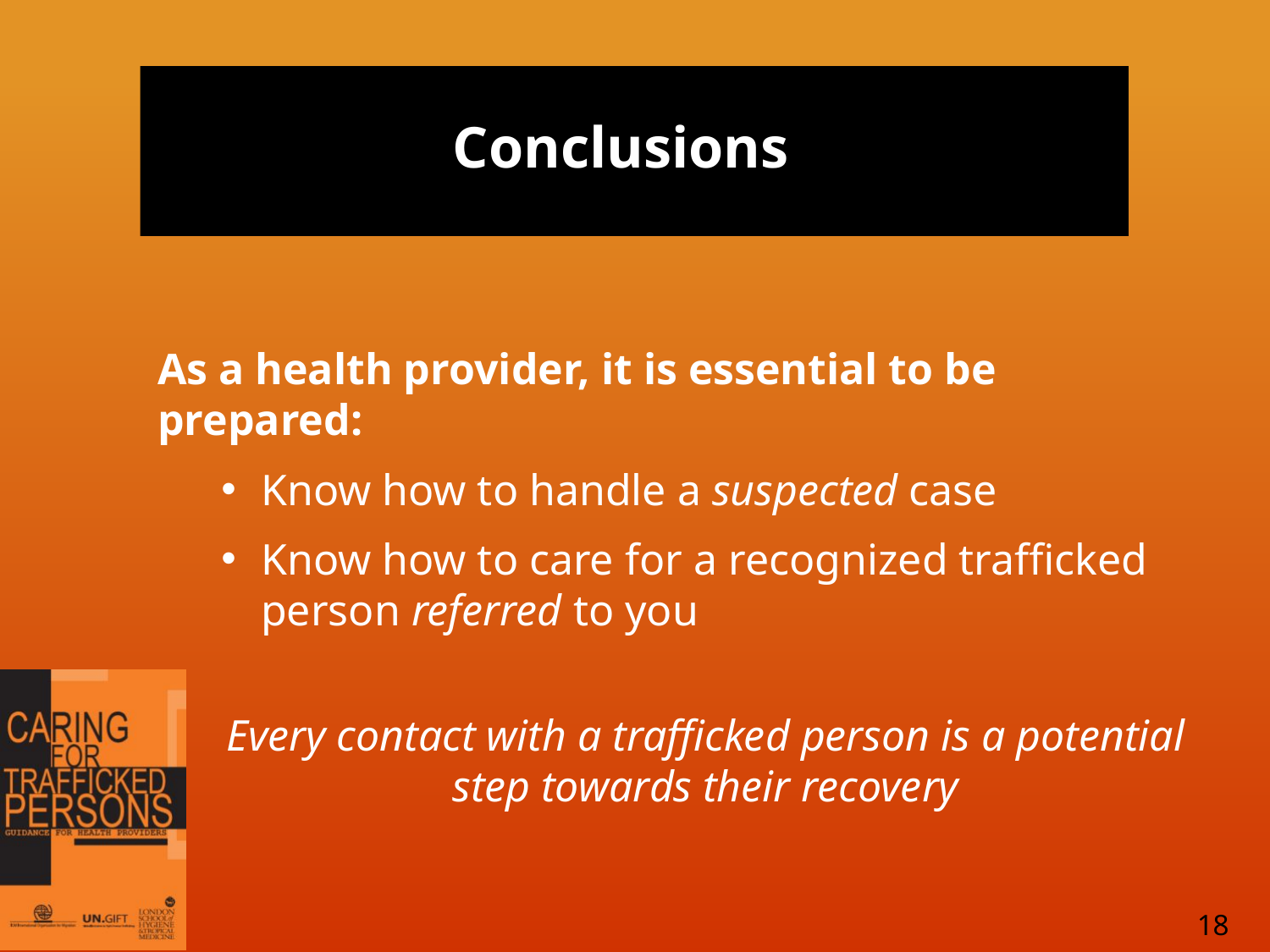

# Conclusions
As a health provider, it is essential to be prepared:
Know how to handle a suspected case
Know how to care for a recognized trafficked person referred to you
Every contact with a trafficked person is a potential step towards their recovery
18

## Slide 19
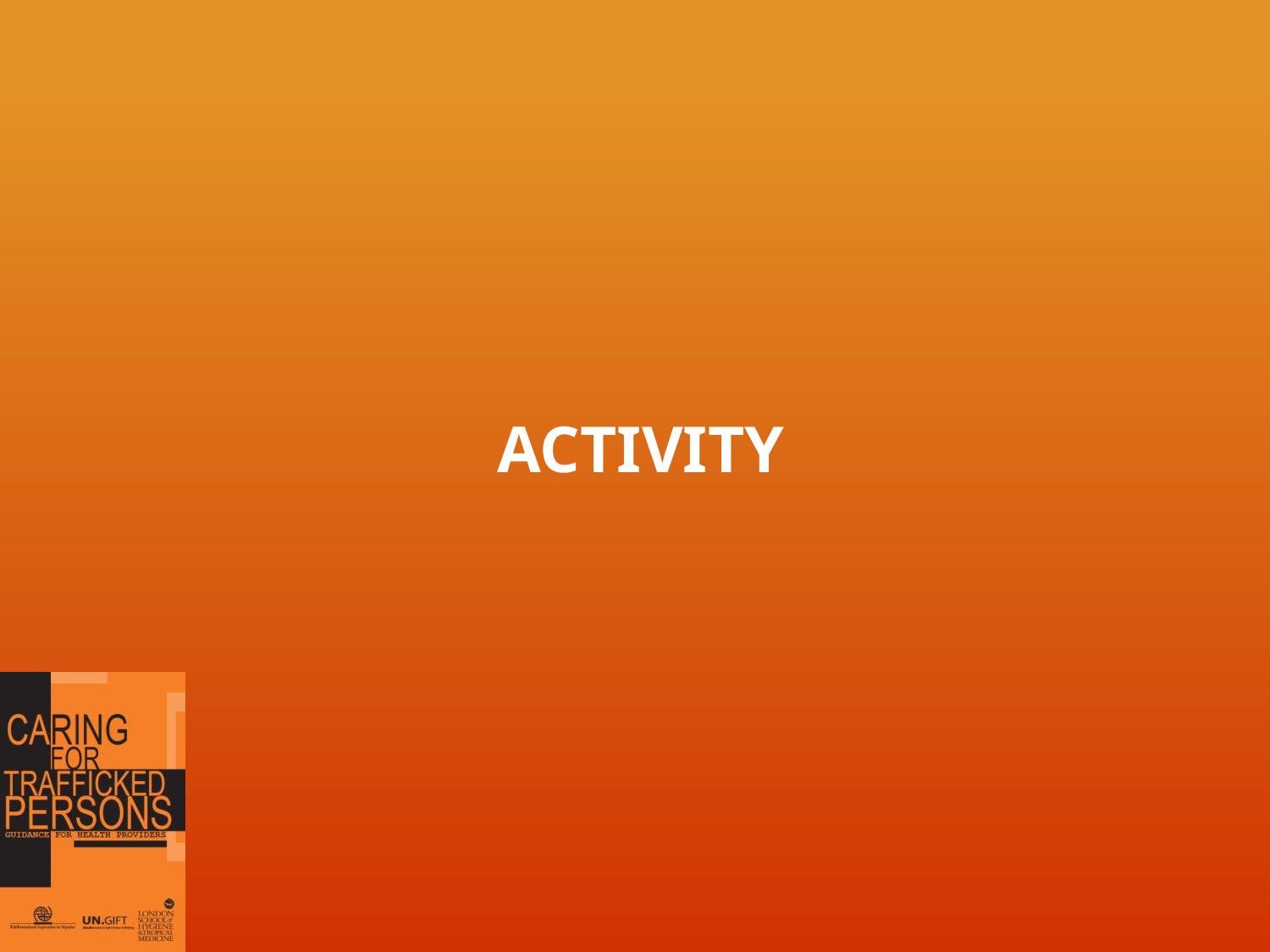

ACTIVITY

## Slide 20
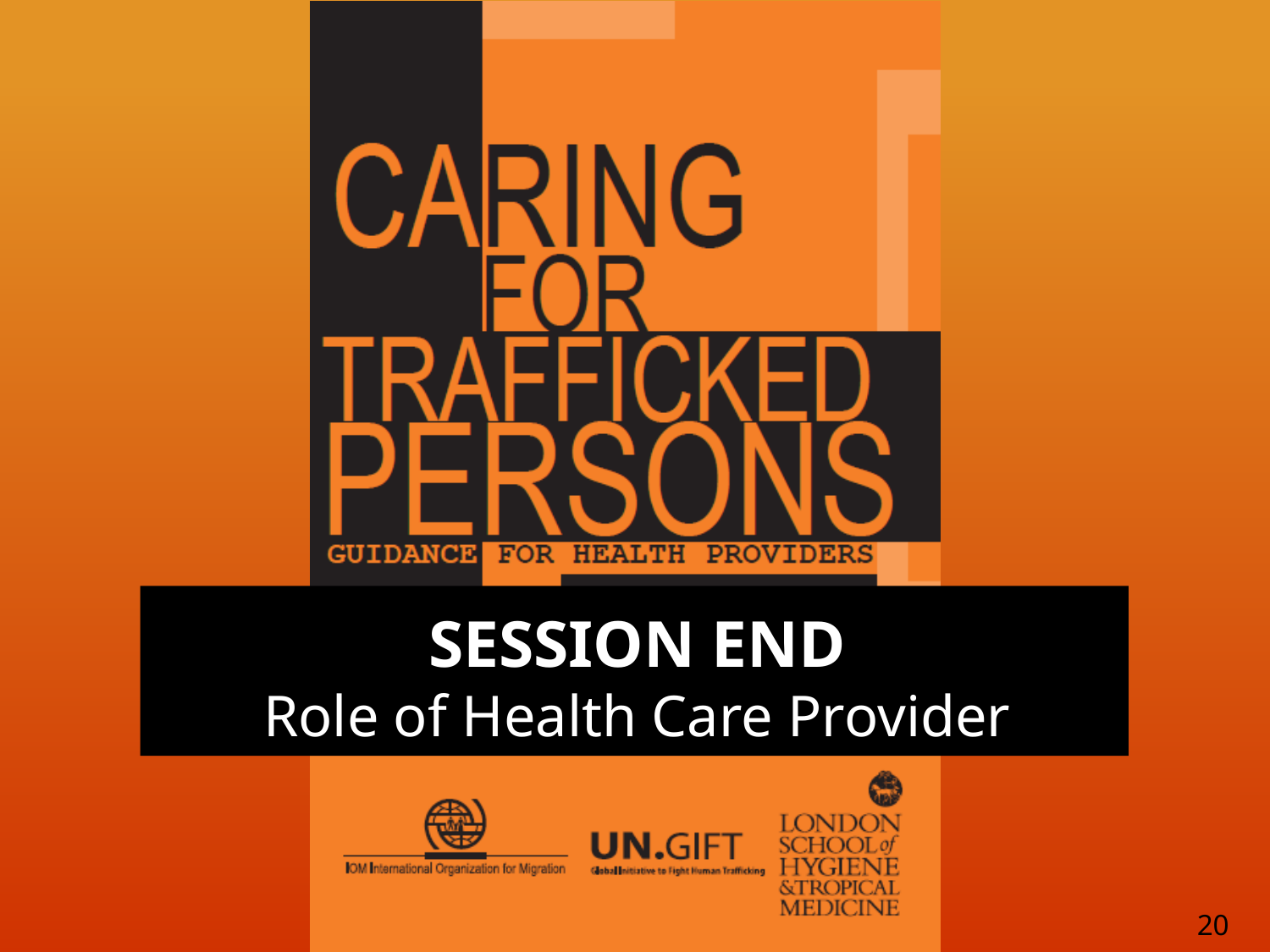

SESSION END
Role of Health Care Provider
20
