# Supplementary material for: Health Care Providers and Human Trafficking: What do They Know, What do They Need to Know? Findings from the Middle East, the Caribbean, and Central America
Source: Front Public Health. 2015 Jan 29;3:6. doi: 10.3389/fpubh.2015.00006 (PMC4310216; doi:10.3389/fpubh.2015.00006)
Supplement: Supplementary file 1 [file Presentation_1.ZIP › Caring for Trafficked Persons Training Session 5.pptx]

## Slide 1
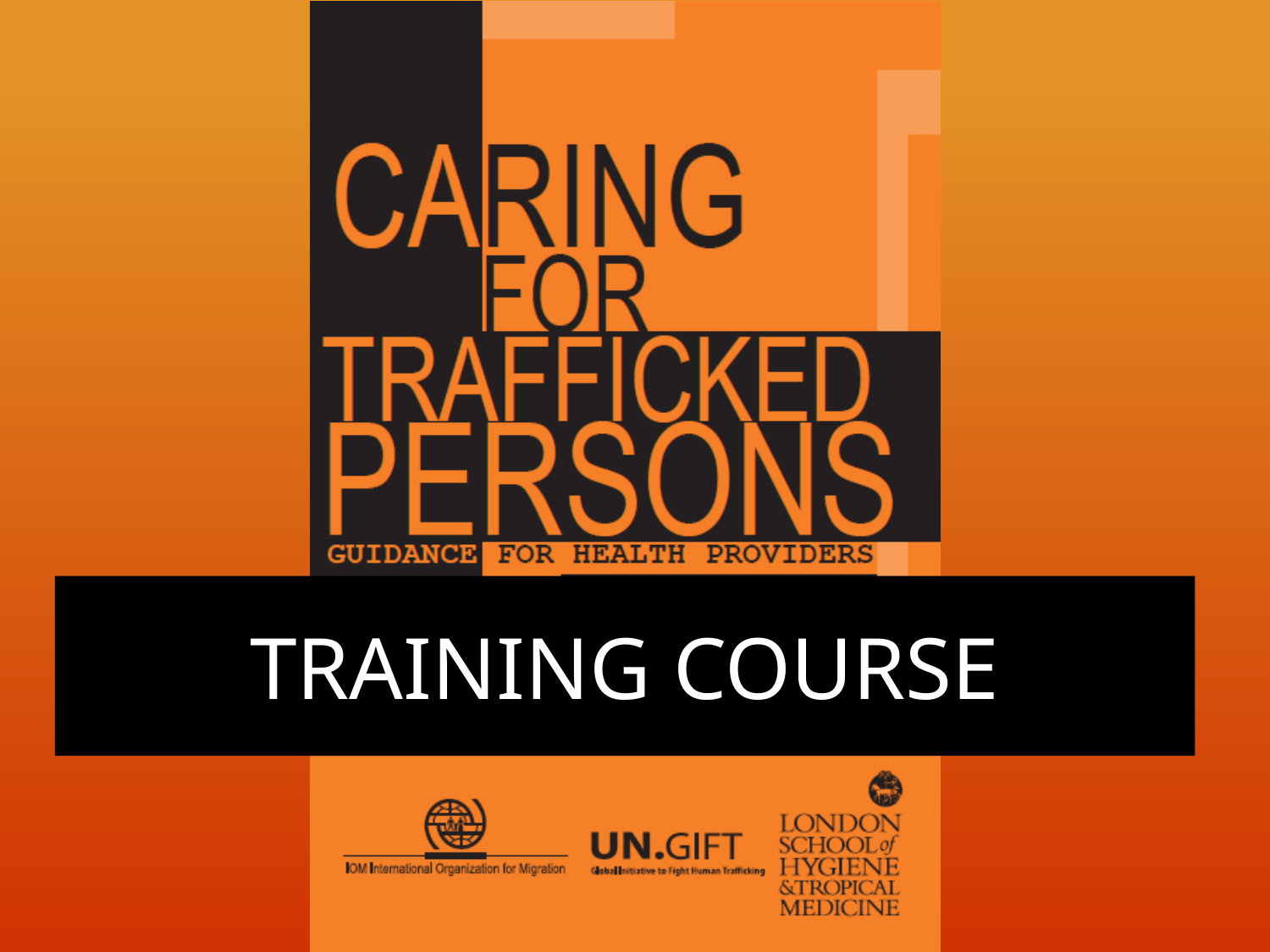

## Slide 2
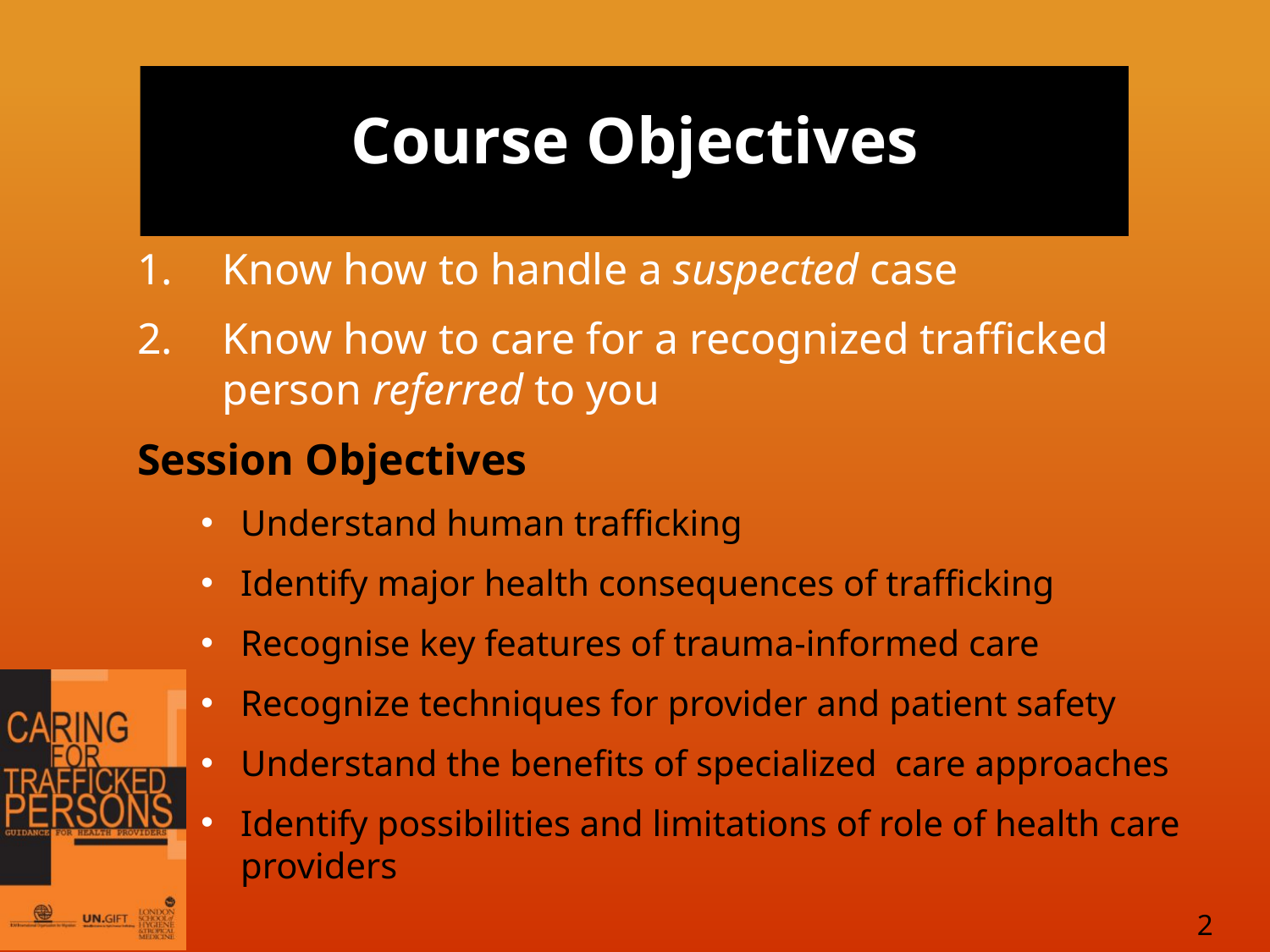

# Course Objectives
Know how to handle a suspected case
Know how to care for a recognized trafficked person referred to you
Session Objectives
Understand human trafficking
Identify major health consequences of trafficking
Recognise key features of trauma-informed care
Recognize techniques for provider and patient safety
Understand the benefits of specialized care approaches
Identify possibilities and limitations of role of health care providers
2

## Slide 3
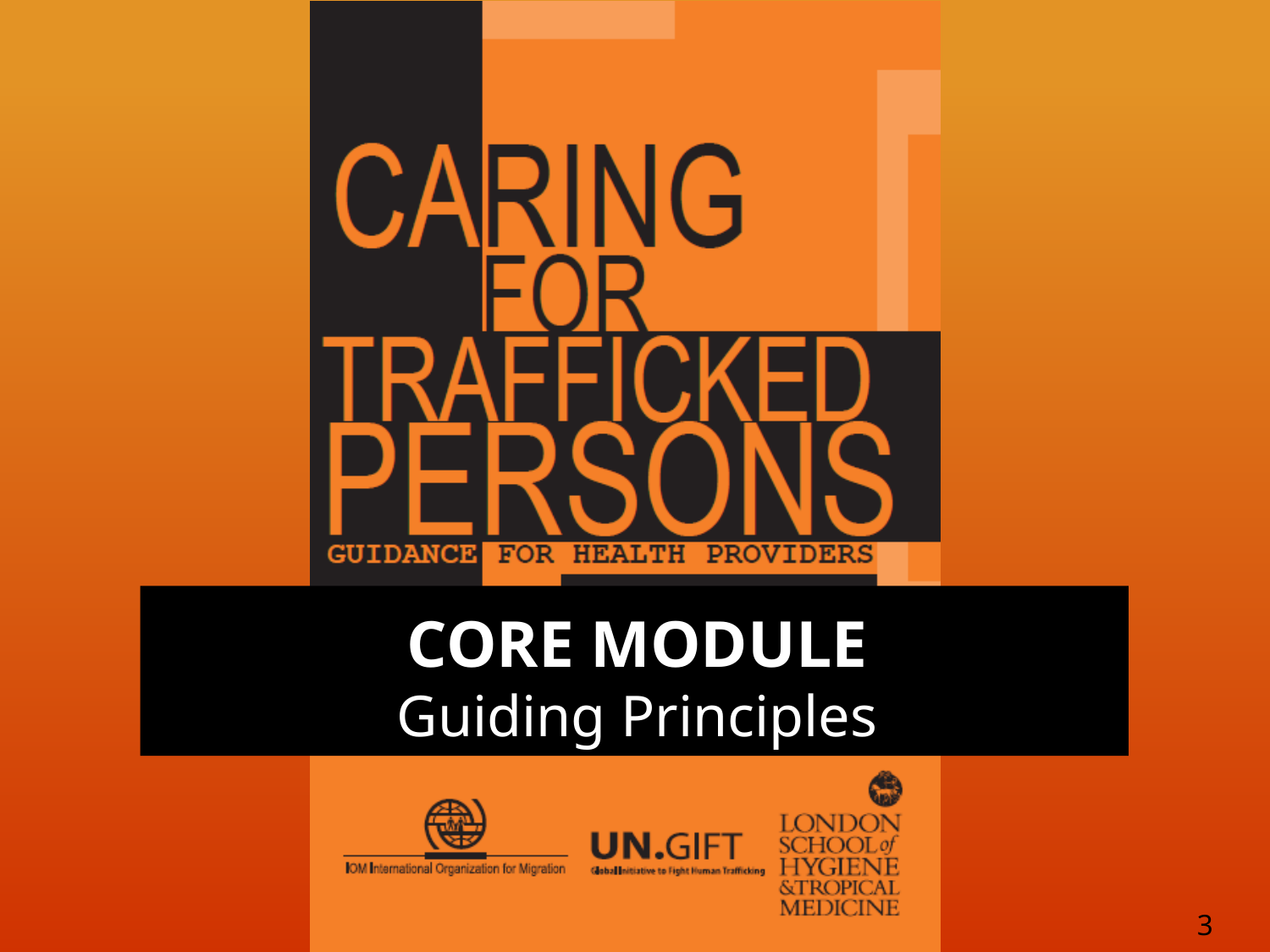

CORE MODULE
Guiding Principles
3

## Slide 4
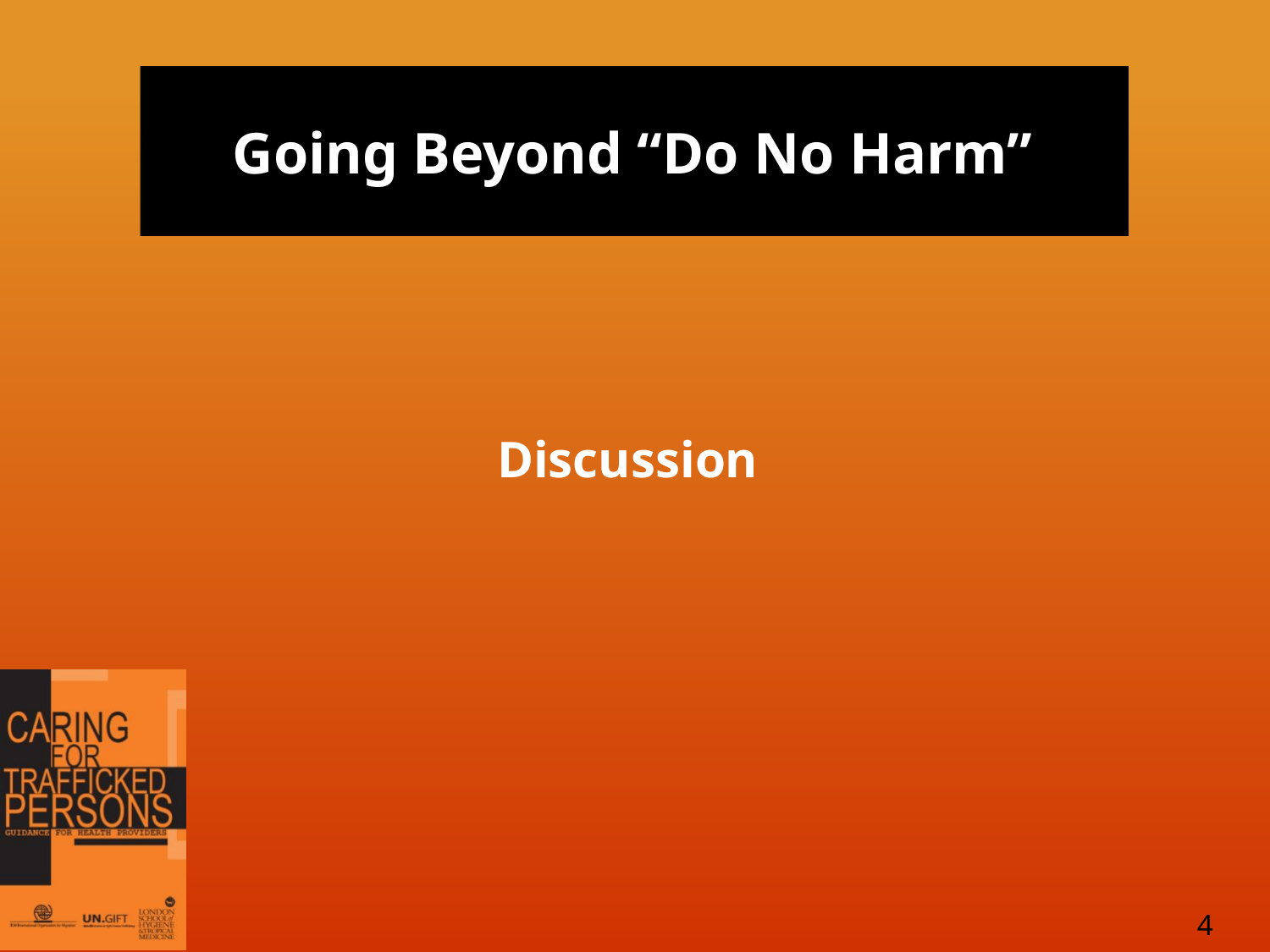

Going Beyond “Do No Harm”
Discussion
4

## Slide 5
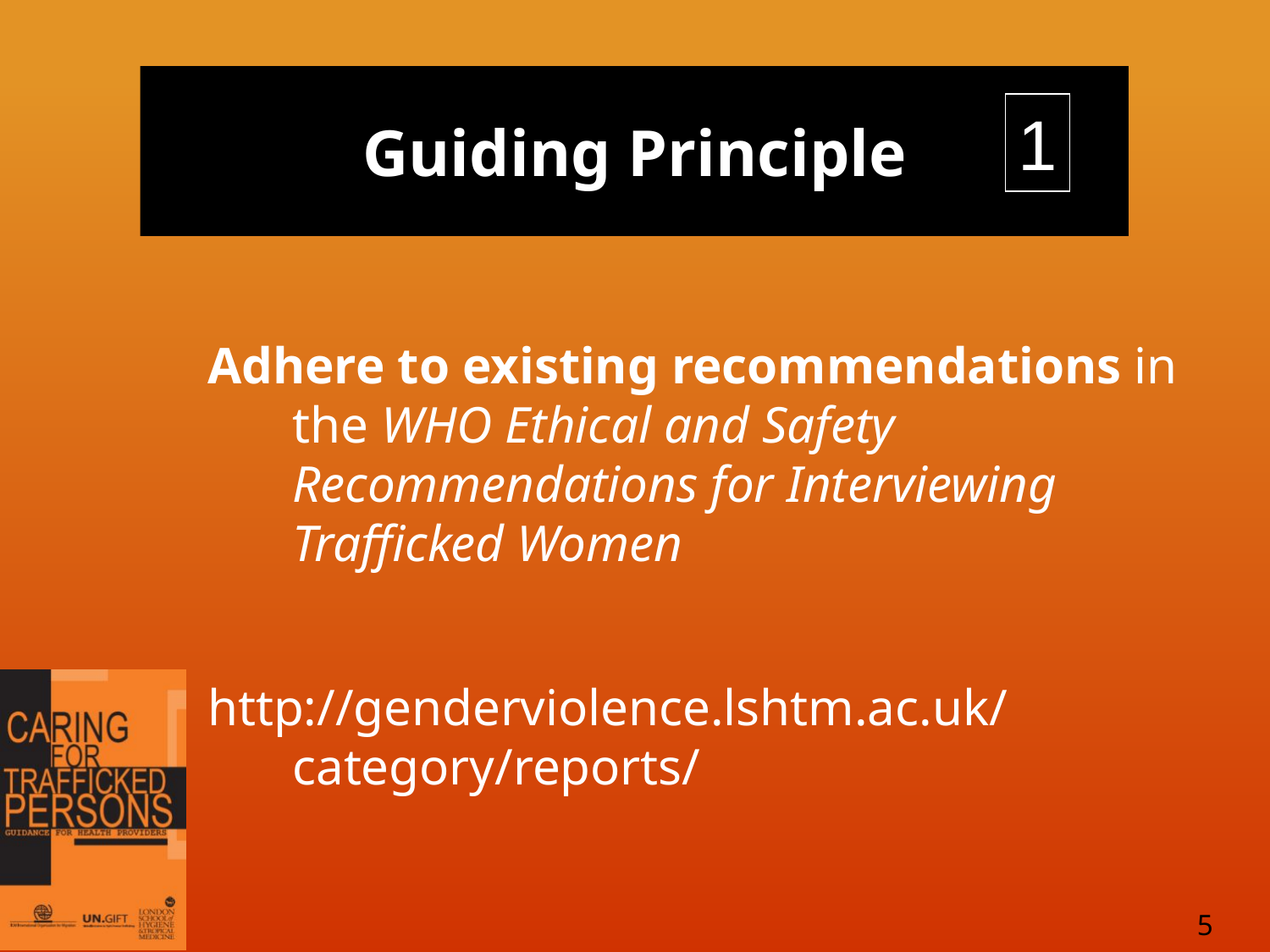

1
Guiding Principle
Adhere to existing recommendations in the WHO Ethical and Safety Recommendations for Interviewing Trafficked Women
http://genderviolence.lshtm.ac.uk/category/reports/
5

## Slide 6
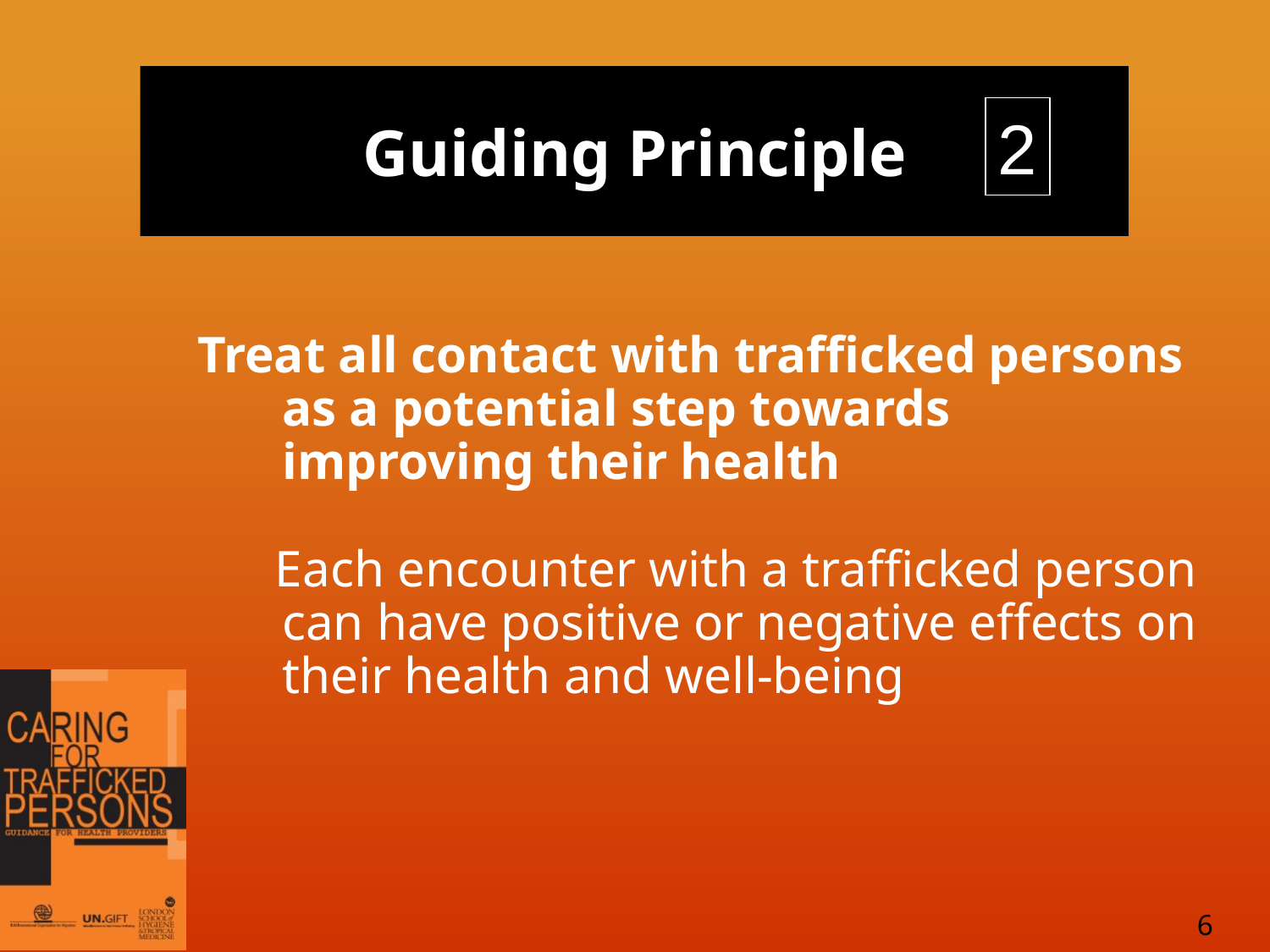

2
Guiding Principle
Treat all contact with trafficked persons as a potential step towards improving their health
 Each encounter with a trafficked person can have positive or negative effects on their health and well-being
6

## Slide 7
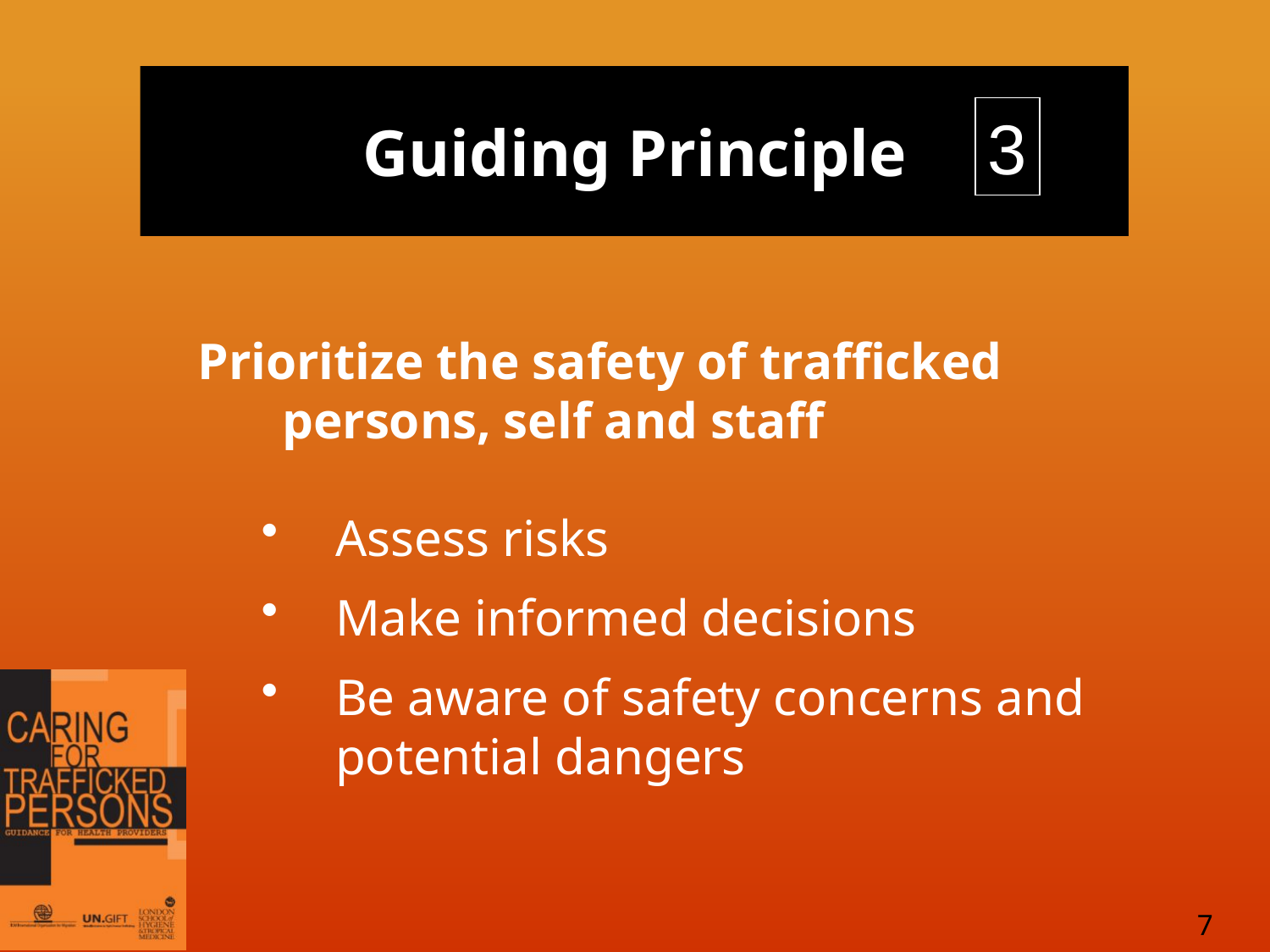

3
Guiding Principle
Prioritize the safety of trafficked persons, self and staff
Assess risks
Make informed decisions
Be aware of safety concerns and potential dangers
7

## Slide 8
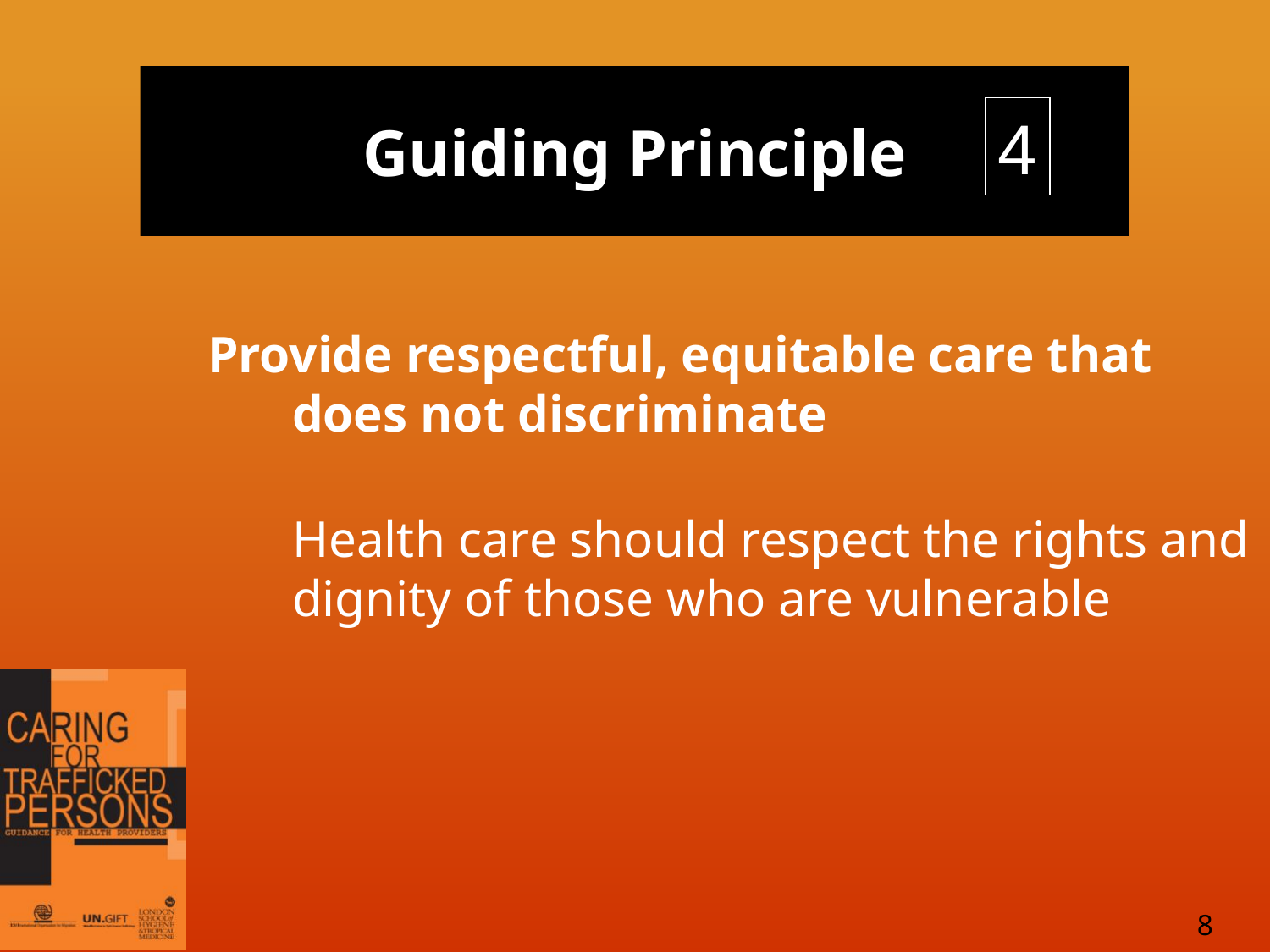

4
Guiding Principle
Provide respectful, equitable care that does not discriminate
	Health care should respect the rights and dignity of those who are vulnerable
8

## Slide 9
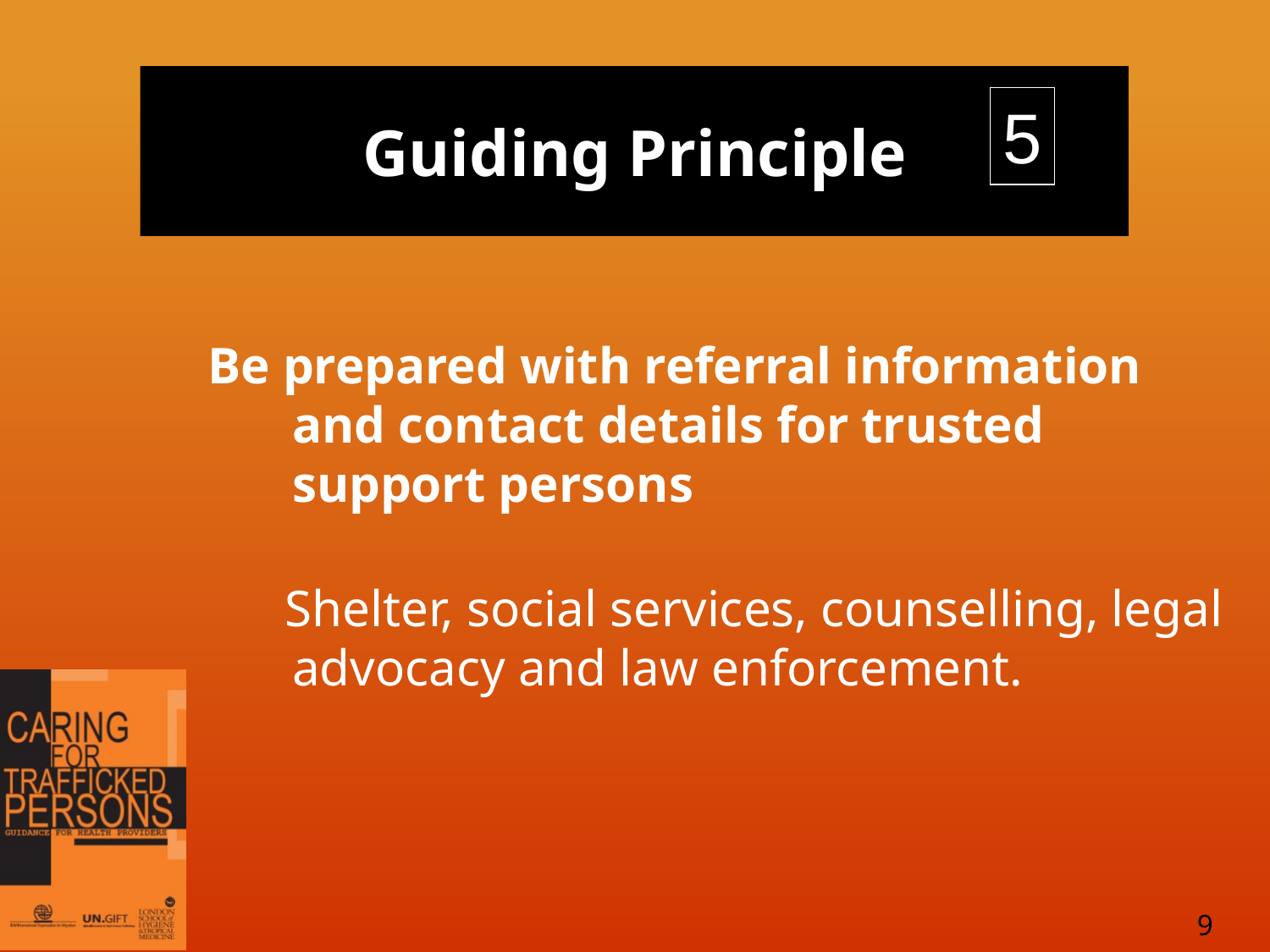

5
Guiding Principle
Be prepared with referral information and contact details for trusted support persons
 Shelter, social services, counselling, legal advocacy and law enforcement.
9

## Slide 10
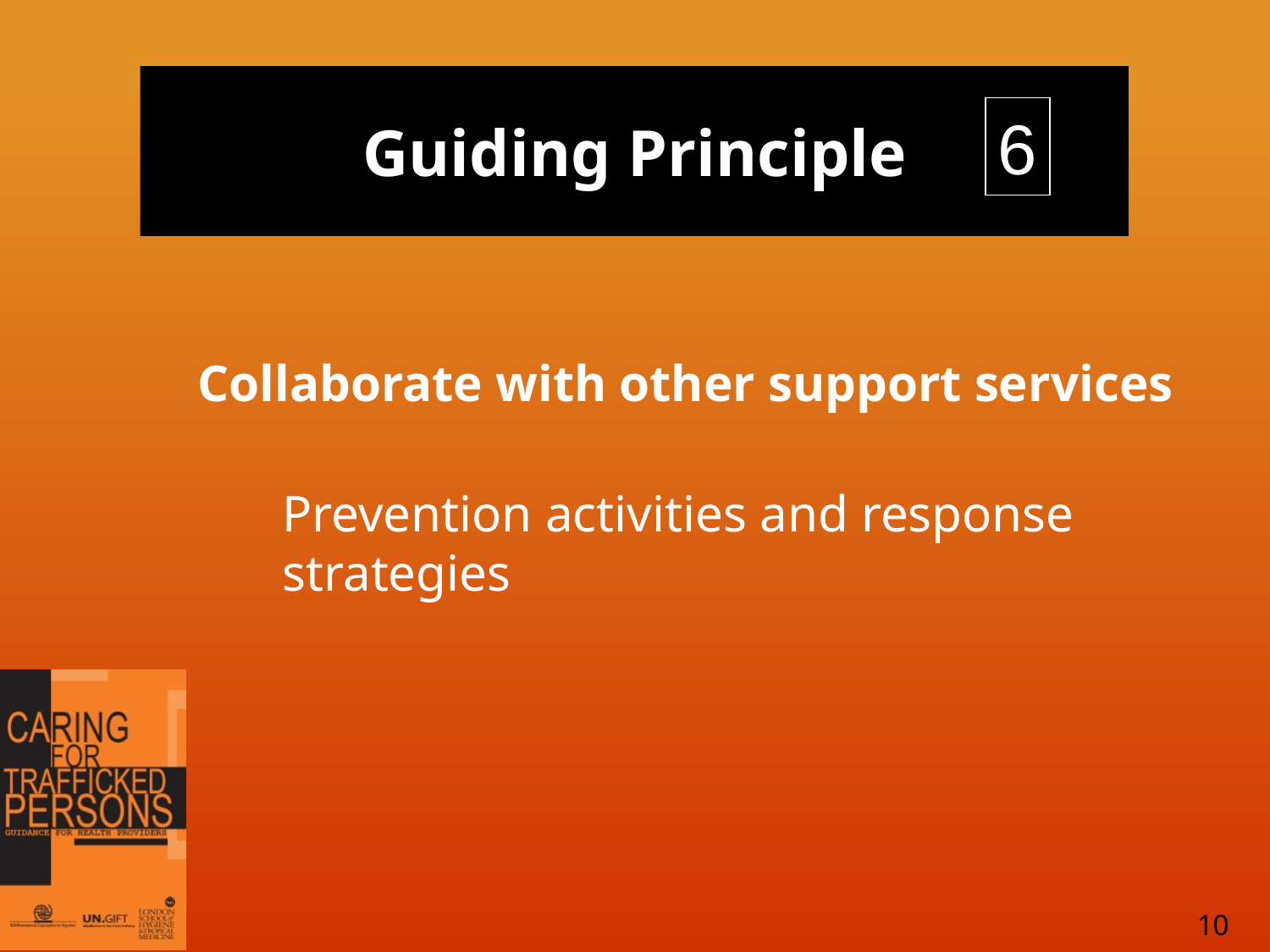

6
Guiding Principle
Collaborate with other support services
	Prevention activities and response strategies
10

## Slide 11
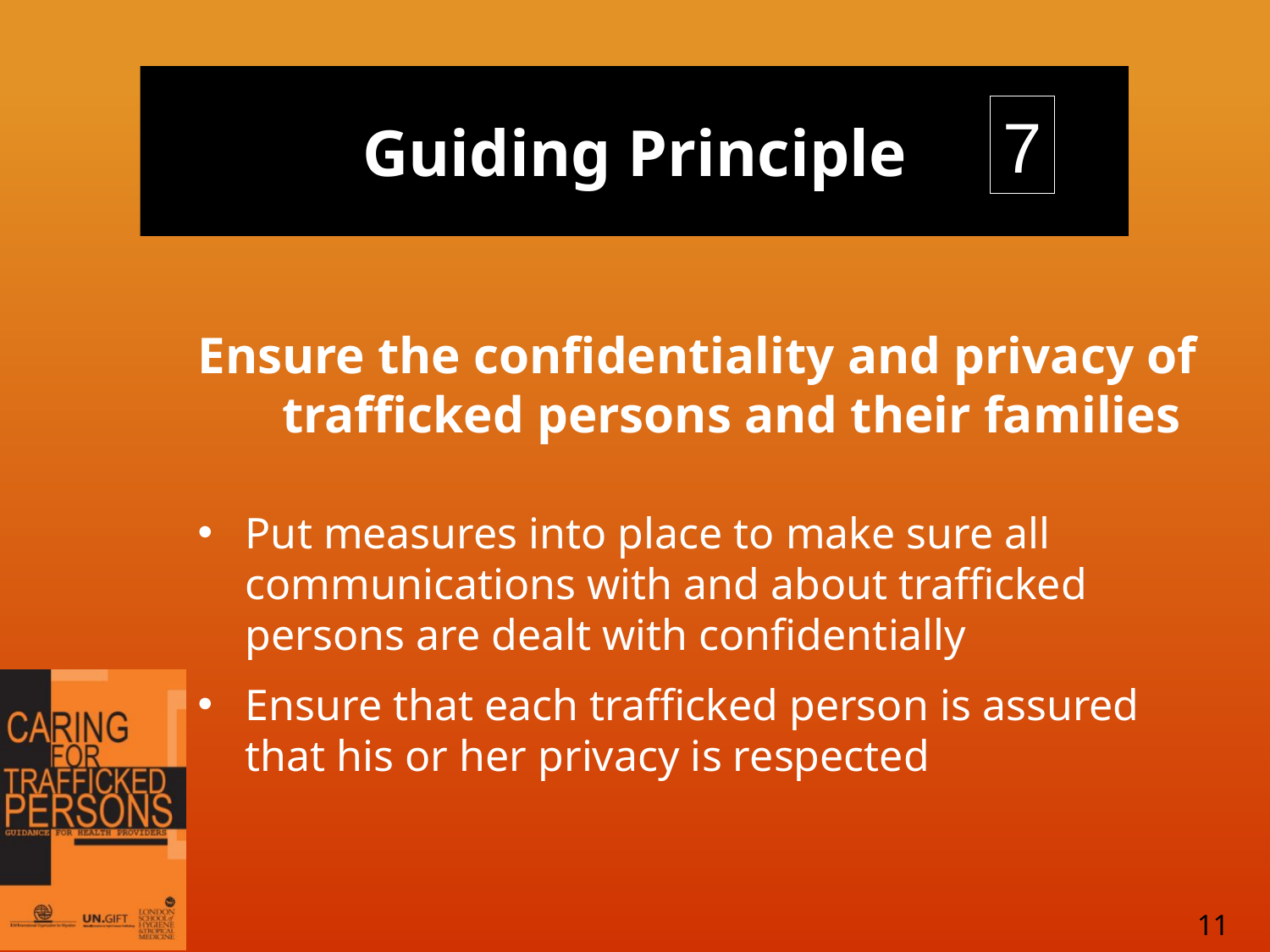

7
Guiding Principle
Ensure the confidentiality and privacy of trafficked persons and their families
Put measures into place to make sure all communications with and about trafficked persons are dealt with confidentially
Ensure that each trafficked person is assured that his or her privacy is respected
11

## Slide 12
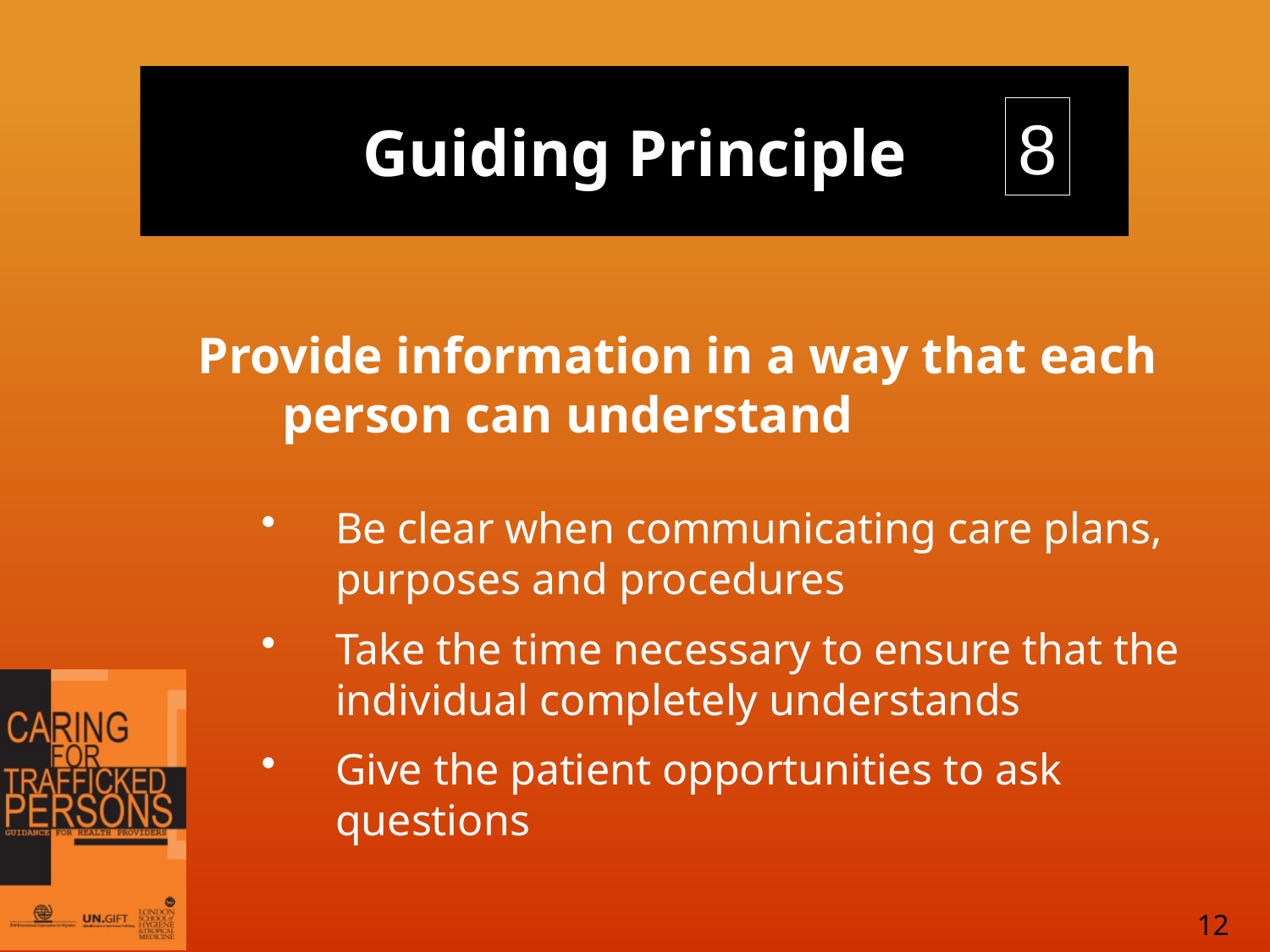

8
Guiding Principle
Provide information in a way that each person can understand
Be clear when communicating care plans, purposes and procedures
Take the time necessary to ensure that the individual completely understands
Give the patient opportunities to ask questions
12

## Slide 13
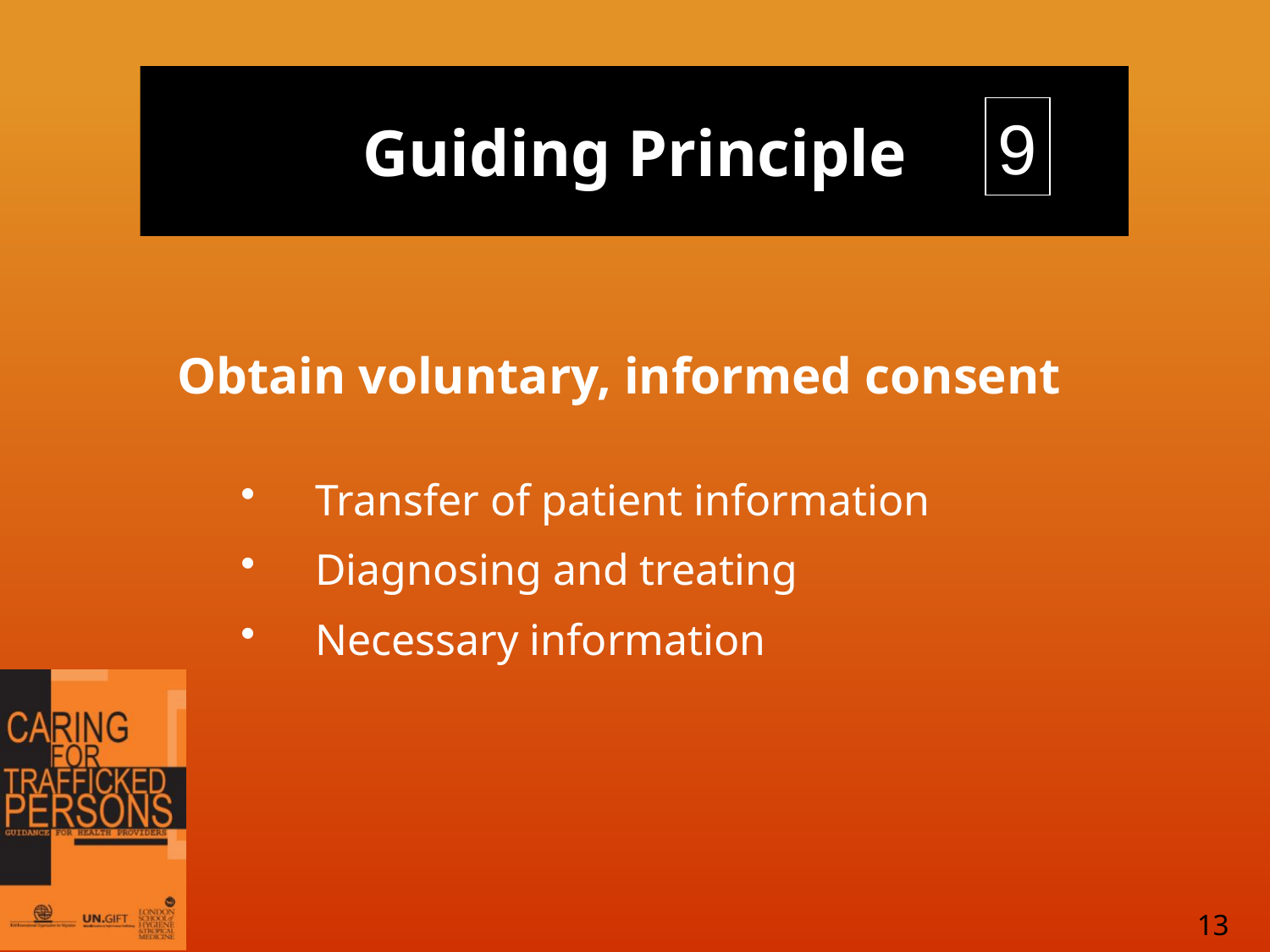

9
Guiding Principle
Obtain voluntary, informed consent
Transfer of patient information
Diagnosing and treating
Necessary information
13

## Slide 14
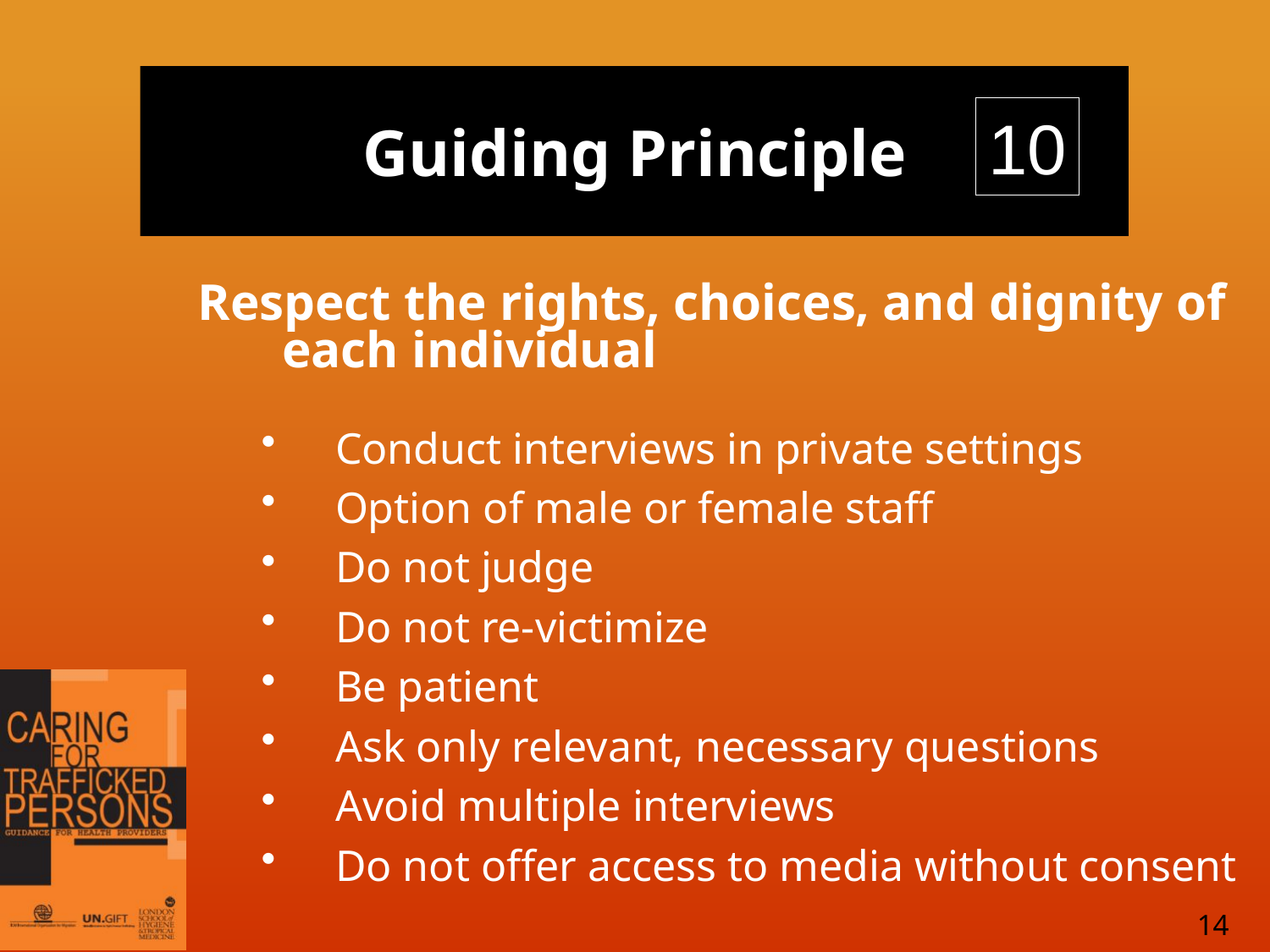

10
Guiding Principle
Respect the rights, choices, and dignity of each individual
Conduct interviews in private settings
Option of male or female staff
Do not judge
Do not re-victimize
Be patient
Ask only relevant, necessary questions
Avoid multiple interviews
Do not offer access to media without consent
14

## Slide 15
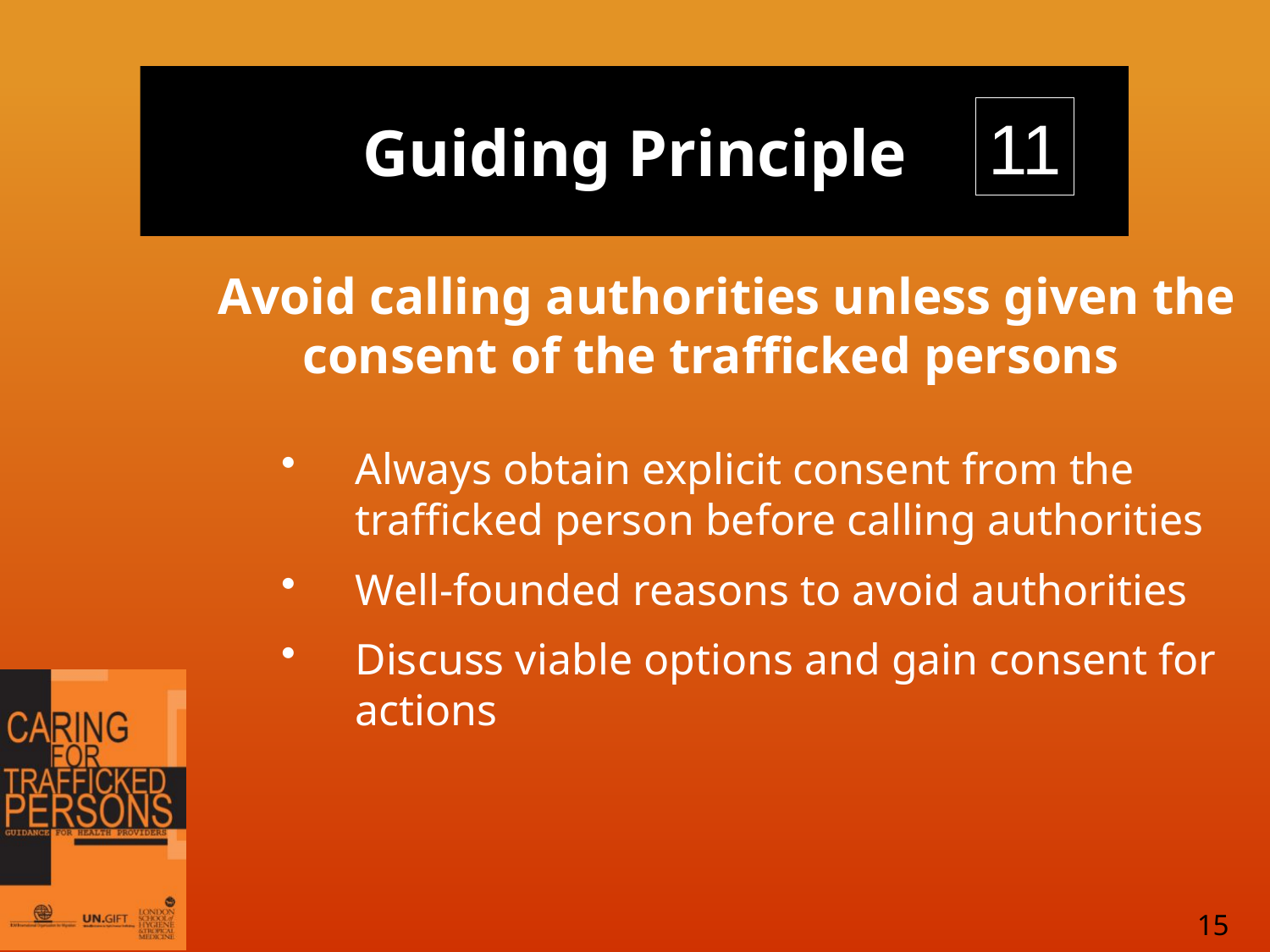

11
Guiding Principle
Avoid calling authorities unless given the consent of the trafficked persons
Always obtain explicit consent from the trafficked person before calling authorities
Well-founded reasons to avoid authorities
Discuss viable options and gain consent for actions
15

## Slide 16
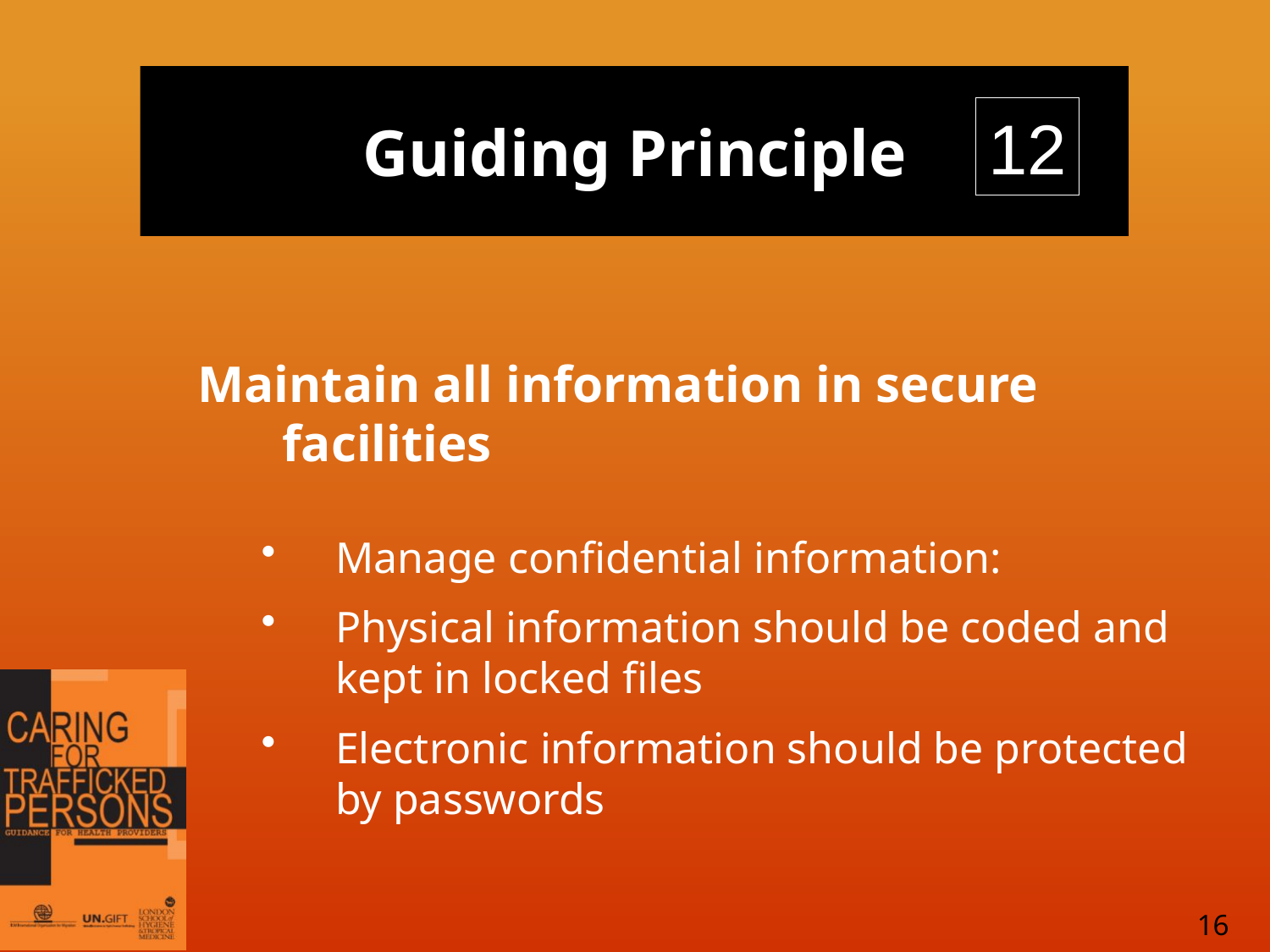

12
Guiding Principle
Maintain all information in secure facilities
Manage confidential information:
Physical information should be coded and kept in locked files
Electronic information should be protected by passwords
16

## Slide 17
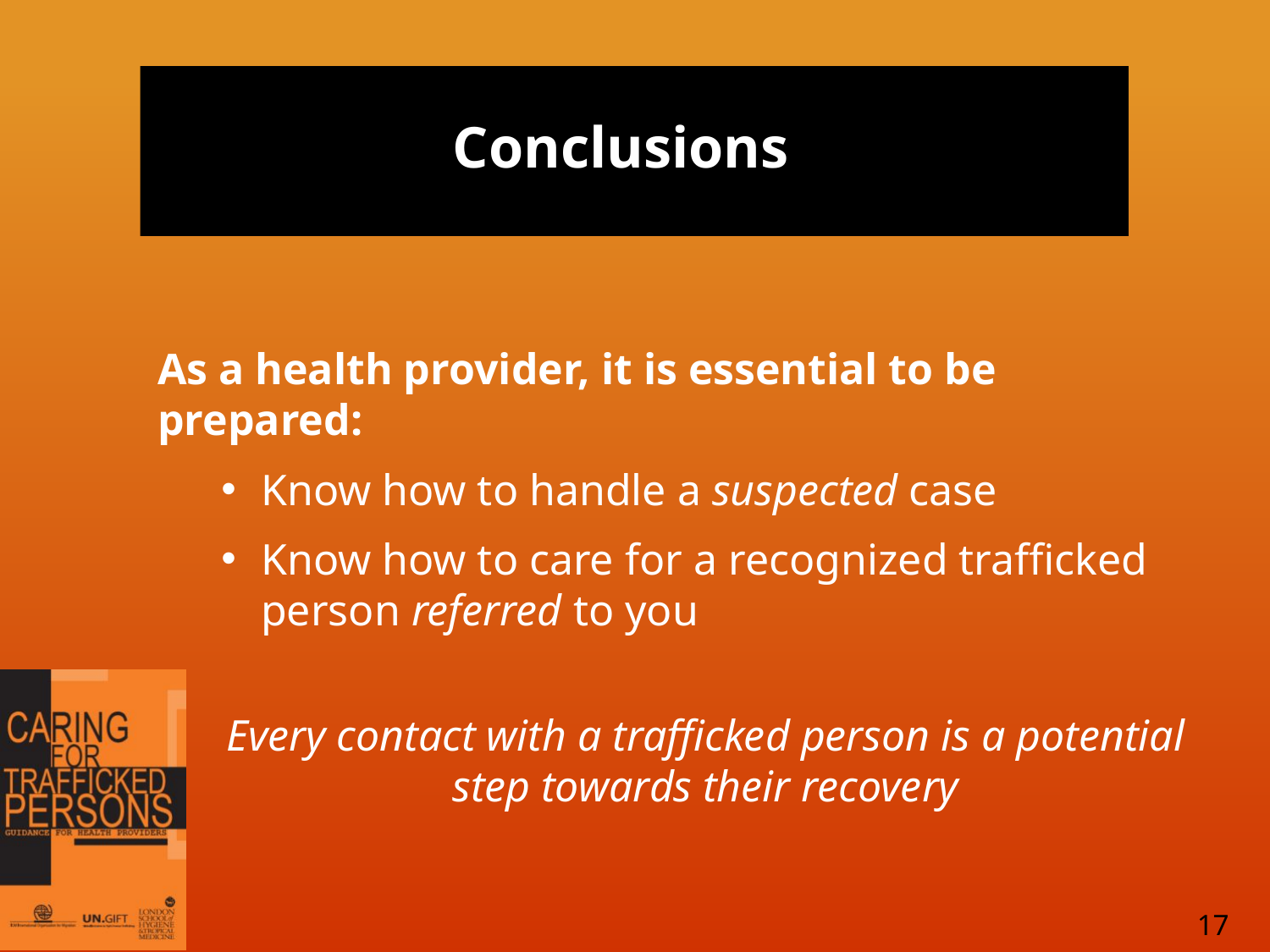

# Conclusions
As a health provider, it is essential to be prepared:
Know how to handle a suspected case
Know how to care for a recognized trafficked person referred to you
Every contact with a trafficked person is a potential step towards their recovery
17

## Slide 18
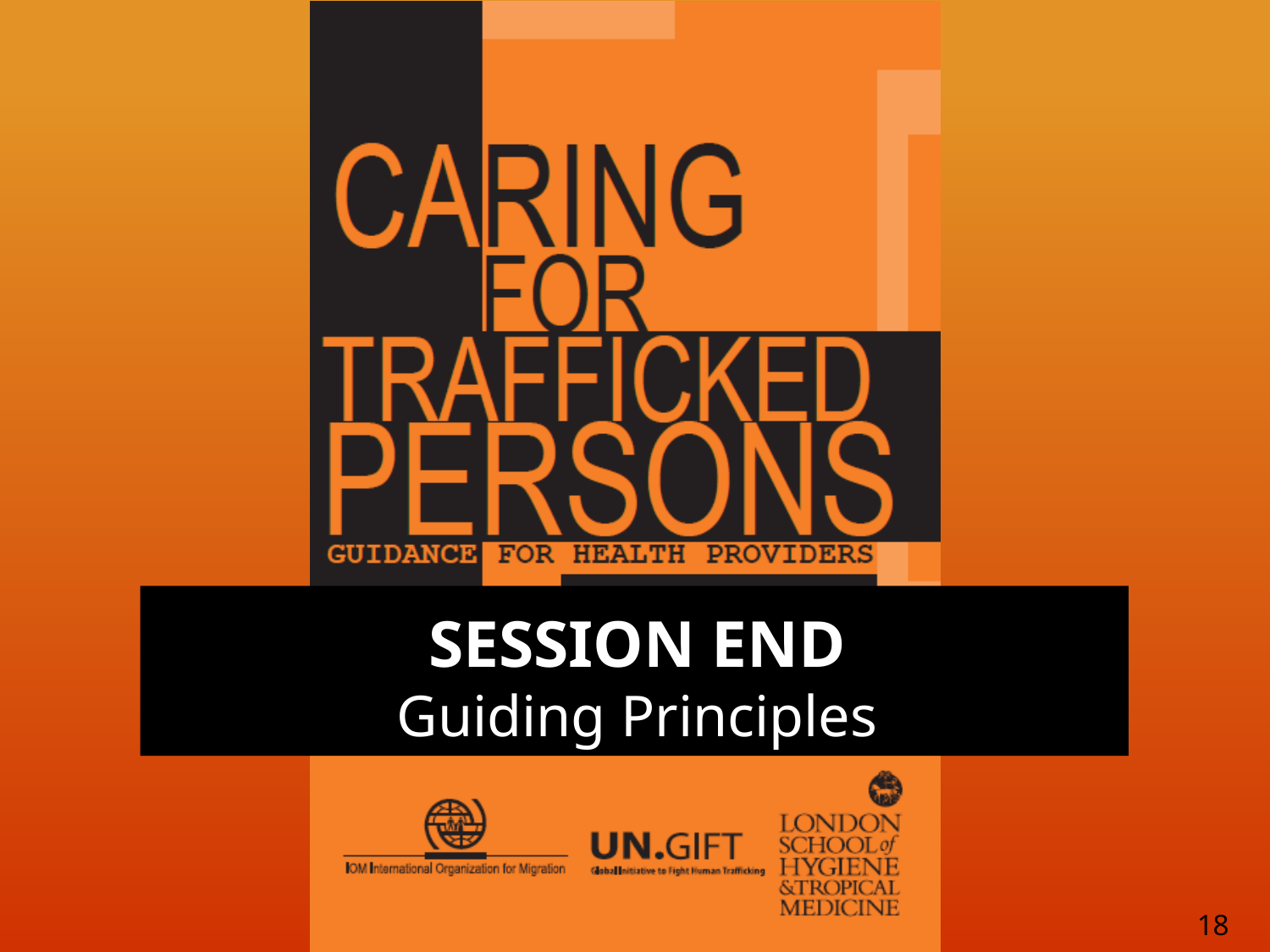

SESSION END
Guiding Principles
18
